# Generation of potent antibacterial compounds through enzymatic and chemical modifications of the *trans*-δ-viniferin scaffold

**DOI:** 10.1038/s41598-023-43000-5

**Published:** 2023-09-25

**Authors:** Robin Huber, Laurence Marcourt, Margaux Héritier, Alexandre Luscher, Laurie Guebey, Sylvain Schnee, Emilie Michellod, Stéphane Guerrier, Jean-Luc Wolfender, Leonardo Scapozza, Thilo Köhler, Katia Gindro, Emerson Ferreira Queiroz

**Affiliations:** 1 School of Pharmaceutical Sciences, University of Geneva, CMU, Geneva, Switzerland; 2 Institute of Pharmaceutical Sciences of Western Switzerland, University of Geneva, CMU, Geneva, Switzerland; 3 Department of Microbiology and Molecular Medicine, University of Geneva, Rue Michel-Servet 1, 1211 Genève 4, Switzerland; 4 Agroscope, Plant Protection Research Division, Mycology Group, Route de Duillier 50, P.O. Box 1012, 1260 Nyon, Switzerland; 5 Geneva School of Economics and Management, University of Geneva, 1205 Geneva, Switzerland

**Keywords:** Drug discovery, Natural product synthesis, Structure elucidation, Antibiotics

## Abstract

Stilbene dimers are well-known for their diverse biological activities. In particular, previous studies have demonstrated the high antibacterial potential of a series of *trans*-δ-viniferin-related compounds against gram-positive bacteria such as *Staphylococcus aureus*. The *trans*-δ-viniferin scaffold has multiple chemical functions and can therefore be modified in various ways to generate derivatives. Here we report the synthesis of 40 derivatives obtained by light isomerization, *O*-methylation, halogenation and dimerization of other stilbene monomers. The antibacterial activities of all generated *trans*-δ-viniferin derivatives were evaluated against *S. aureus* and information on their structure–activity relationships (SAR) was obtained using a linear regression model. Our results show how several parameters, such as the *O*-methylation pattern and the presence of halogen atoms at specific positions, can determine the antibacterial activity. Taken together, these results can serve as a starting point for further SAR investigations.

## Introduction

The first viniferin (ε-viniferin) was isolated by Langcake and Pryce from the vine leaves of *Vitis vinifera* L. (Vitaceae family) that had been infected with the necrotrophic fungus *Botrytis cinerea* Pers.^1^. As already pointed out by Beaumont et al., the term “viniferin” could be a source of confusion, since δ-, ε-, and ω-viniferin are resveratrol dimers, α-viniferin is a resveratrol trimer, and r2- and r-viniferin (also known as Vitisin A and Vitisin B, respectively) are resveratrol tetramers^2^. Viniferin are found in a large range of plant genera (*Vitis*, *Gnetum*, *Shorea*, *Rheum*, *Parthenocissus*, *Paeonia*, *Iris*, *Hopea*, *Dryobalanops*, *Dipterocarpus*, *Cyphostemma*, *Cayratia*, *Carex*, *Caragana*, *Bombax*, *Astilbe*)^3^ but δ-viniferin (also known as Maximol A) has only been described in the *Vitis*, *Rheum* and *Gnetum* genera^3^.

Viniferins are commonly biosynthesized by regioselective oxidative coupling of two, three or four units of stilbene monomers such as resveratrol^4,5^. They have been described with a wide range of biological properties. In plants, these compounds are produced in response to aggressive attacks from pathogens and herbivores or to protect against damage from UV radiation^2^. In addition, their involvement in the inhibition of bacterial or fungal growth has been highlighted in several studies^3,6–10^.

In previous work, a large number of stilbene dimers have been obtained by chemoenzymatic dimerization of stilbene units such as resveratrol and pterostilbene. These reactions were carried out with an enzyme-enriched fraction from the fungus *Botrytis cinerea* (“fungal secretome”) and resulted in the generation of more than 70 compounds^11^. The dimers obtained were classified into five structural families, namely *trans*-δ-viniferin, pallidol, leachianol, restrytisol, and acyclic dimer. The *trans*-δ-viniferin family, and in particular two *O*-methylated derivatives have shown remarkable biological activities against methicillin- and vancomycin-resistant strains of *Staphylococcus aureus*^12^. The formation of all dimers is thought to occur through phenoxy radical couplings generated by laccases present in the enzymatic secretome. Although enzymes are involved in this process, the resulting compounds are mixtures of enantiomers. Indeed, laccases generate planar phenoxy radicals, which are then randomly combined in solution with no enantioselectivity^5^.

The *trans*-δ-viniferin and its derivatives have two chiral centers, at C-7′ and C-8′ positions on the dihydrofuran ring, giving two potential enantiomers (the relative configuration between these positions is always *trans* in this scaffold). In our previous study, targeted chiral isolation of the enantiomers allowed their bioactivity to be assessed independently, and no significant differences were found compared to the racemic mixtures. This suggests that further work can be done on the racemic mixture^13^.

In parallel, considerable work has been done on the *trans*-δ-viniferin family by synthesizing numerous derivatives to improve their antibacterial activity^8,9^. Simplified molecules have also been prepared by removing the aromatic moieties to elucidate the structural requirements for the activity^10^.

To further explore the *trans*-δ-viniferin scaffold, we synthesized a series of closely related derivatives of *trans*-δ-viniferin with the aim of (1) gaining a better understanding of the features responsible for their antimicrobial properties and (2) obtaining more potent compounds with enhanced activity. The stereochemistry of the double bond was light-modified to obtain the *cis*-isomers of given *trans*-δ-viniferin derivatives. The role of the *O*-methylation pattern was investigated by generating all possible mono- and di-*O*-methylated derivatives. A methoxylated derivative of *trans*-δ-viniferin was obtained by enzymatic dimerization of isorhapontigenin, a less common stilbene. Oxidized derivatives containing a benzofuran moiety, whose activity against *S. aureus* was enhanced in the case of *trans*-δ-viniferin, were synthesized^8^. Finally, halogenated derivatives (chlorinated, brominated, iodinated) were synthesized. The antibacterial activity of these 40 *trans*-δ-viniferin derivatives was evaluated against *S. aureus*.

## Results and discussion

### Synthesis, isolation and characterization of *trans*-δ-viniferin derivatives

**Figure 1:**
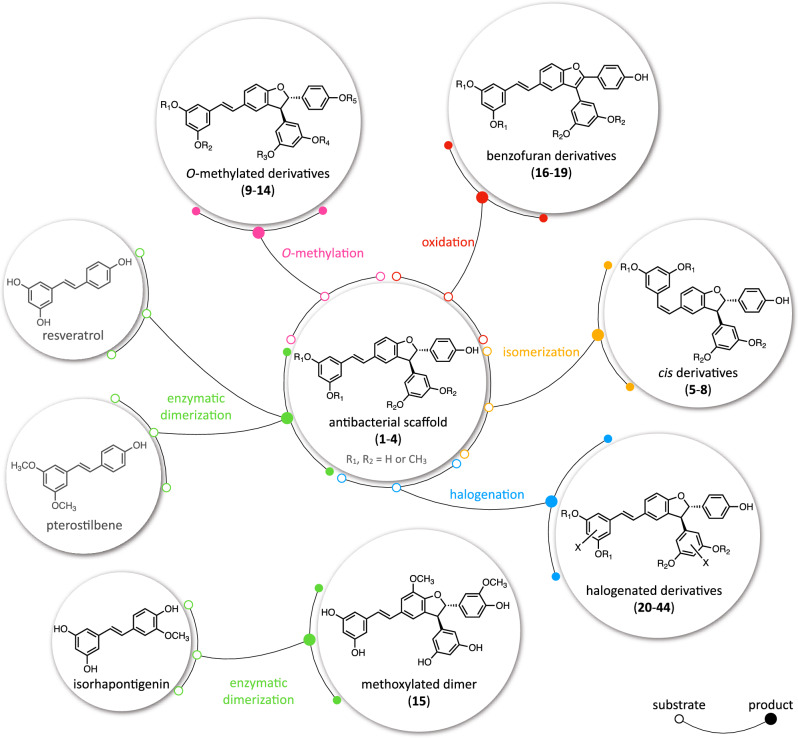
Overview of the *trans*-δ-viniferin derivatives obtained by chemical and chemoenzymatic methods (4 *cis* derivatives, 6 *O*-methylated derivatives, 1 methoxylated derivative, 4 benzofuran derivatives and 25 halogenated derivatives).

*Trans*-δ-viniferin derivatives (**1**–**4**) were first obtained in large quantities by chemoenzymatic synthesis using the enzymatic secretome of *B. cinerea* according to the previously developed protocol (see experimental section)^12^. The compounds were obtained in high purity using a high-resolution preparative chromatography separation procedure. The structures were confirmed using conventional spectroscopic methods. These four compounds (**1**–**4**) were used to generate a library of 40 derivatives (**5**–**44**) using chemical and chemoenzymatic methods described below (Fig. 1). In each case, a purification workflow based on ultra-high performance liquid chromatography (UHPLC) or high-performance liquid chromatography (HPLC) optimization followed by gradient transfer to semi-preparative HPLC was applied^14^, allowing the efficient collection of the target compounds. Samples were introduced by dry load according to a protocol recently developed in our laboratory^15^. These compounds were fully characterized by high-resolution mass spectrometry (HRMS), nuclear magnetic resonance (NMR) and ultraviolet spectroscopy (UV).

**Figure 2:**
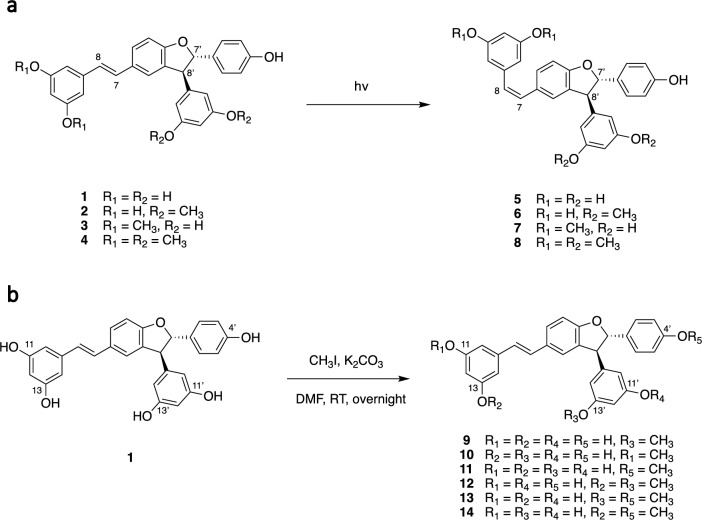
(**a**) Light isomerization of *trans*-δ-viniferin derivatives to *cis*-δ-viniferin derivatives. (**b**) Synthesis of the *O*-methylated *trans*-δ-viniferin derivatives.

First, the *trans* double bond was light isomerized to give the corresponding *cis* isomers. This type of isomerization is well-known for stilbenes and significantly alters the overall molecular shape, which can help in understanding the structure–activity relationships (SAR). Compounds **1**–**4** were dissolved in DMSO and exposed to white light for 7 days. Samples were taken at different times and profiled by UHPLC-PDA-ELSD-MS (data not shown). The ratio of *trans* to *cis* isomers evolved to a 1:1 ratio by day 5 and was stable thereafter. The samples were dried after 7 days. To remove the remaining *trans* isomers, each sample was purified according to the workflow described in Fig. S1. The *cis* and *trans* isomers were easily distinguished by comparing their UV absorptions at 280 and 320 nm. This allowed the targeted isolation of the *cis* compounds **5**–**8** (Fig. 2a).

The isomerization of the C-7/C-8 double bond was confirmed by NMR from the ^3^*J*_H7-H8_ coupling constant, which was about 16 Hz in the *trans*-δ-viniferin and 12 Hz in the *cis*-δ-viniferin derivatives. The structures of the 4 *cis*-δ-viniferin were confirmed by 2D NMR experiments (see experimental section).

Our previous results indicated that the *O*-methylation pattern of *trans*-δ-viniferin was key for antibacterial activity^12^. Indeed, the previously generated compounds **2** and **3** with two *O*-methyl groups had the lowest MICs against *S. aureus* of the series (MIC of 4 and 2 µM, respectively). In contrast, compound **4** with four *O*-methyl groups completely lost its activity (MIC > 250 µM), while **1** had an intermediate effect (MIC 35 µM)^12^. To better understand this behavior, all possible mono- and di-*O*-methylated derivatives of *trans*-δ-viniferin were chemically synthesized. The protocol was based on that of Peñalver et al*.* for the resveratrol alkylation^16^. It uses the simple reagent iodomethane and a weak base, starting, in our case, from compound **1**. The main challenge was to control the reaction to avoid poly-*O*-methylation, which was achieved by controlling the amount of iodomethane and base as well as the reaction time and temperature. The advantage of this method is that it produces all the targeted isomers in a single reaction. A clear readout was obtained by monitoring the reaction on UHPLC-PDA-ELSD-MS, with MS detection allowing easy interpretation of the presence of mono-, di- or poly-*O*-methylated derivatives based on their *m/z* ratios (Fig. S2a). Once the best conditions for obtaining mono- and di-*O*-methylated derivatives were found (see experimental section), the reaction was performed on a larger scale (50 mg of **1**). The separation of the mixture containing several *O*-methylated derivatives was carried out as described in Fig. S2b. This yielded the series of mono-*O*-methylated compounds **9**, **10**, and **11** and di-*O*-methylated compounds **12**, **13** and **14** (Fig. 2b).

Study of the ^1^H NMR spectra in the aromatic region allowed the identification of the various *O*-methylation patterns. The chemical shifts of the protons of the two resorcinol groups of the fully hydroxylated form (*trans*-δ-viniferin) were: 6.03 (2H, d, *J* = 2.1 Hz, H-10′, H-14′), 6.10 (1H, t, *J* = 2.1 Hz, H-12′), 6.11 (1H, t, *J* = 2.2 Hz, H-12), and 6.37 (2H, d, *J* = 2.2 Hz, H-10, H-14). For compound **9**, the H-10, H-12 and H-14 protons appear at almost the same chemical shift (δ_H_ 6.37 for H-10 and H-14 and 6.11 for H-12), while the other aromatic system was split into 3 triplets, indicating that the *O*-methylation took place in C-11. For compound **10**, the protons H-10′, H-12′ and H-14′ were observed at almost the same chemical shift as in *trans*-δ-viniferin (δ_H_ 6.04 for H-10′ and H-14′ and 6.10 for H-12′), while the other aromatic system was split into 3 triplets, indicating the *O*-methylation in C-11′. For **11**, both resorcinol systems were conserved at δ_H_ 6.04 (2H, d, *J* = 2.2 Hz, H-10′, H-14′), 6.11 (2H, 2xt, *J* = 2.2 Hz, H-12, H-12′), and 6.37 (2H, d, *J* = 2.2 Hz, H-10, H-14), but the H-2′/H-6′ and H-3′/H-5′ systems were shifted downfield from 6.76 (2H, d, *J* = 8.6 Hz, H-3′, H-5′), and 7.18 (2H, d, *J* = 8.6 Hz, H-2′, H-6′) for the *trans*-δ-viniferin to 6.94 (2H, d, *J* = 8.6 Hz, H-3′, H-5′), and 7.30 (2H, d, *J* = 8.6 Hz, H-2′, H-6′) for **11**. The *O*-methylation was therefore expected to be at C-4′. For **13**, the *O*-methylation was assumed to be in C-4′ and C-11′ based on the chemical shift values of H-3′/H-5′ and H-2′/H-6′ at δ_H_ 6.94 and 7.31, respectively as in **11** and those of H-10/H-14 and H-12 at δ_H_ 6.37 and 6.11, respectively, as in *trans*-δ-viniferin. For **14**, the downfielded chemical shift values of H-3′/H-5′ and H-2′/H-6′ as in **11** and the conserved chemical shifts of H-10′/H-14′ and H-12′ indicated C-4′ and C-11 *O*-methylation. For **12**, the two resorcinol systems were modified for three triplets each showing a C-11 and C-11′ *O*-methylation. All these structures were confirmed by 2D NMR analysis (see experimental section).

**Figure 3:**
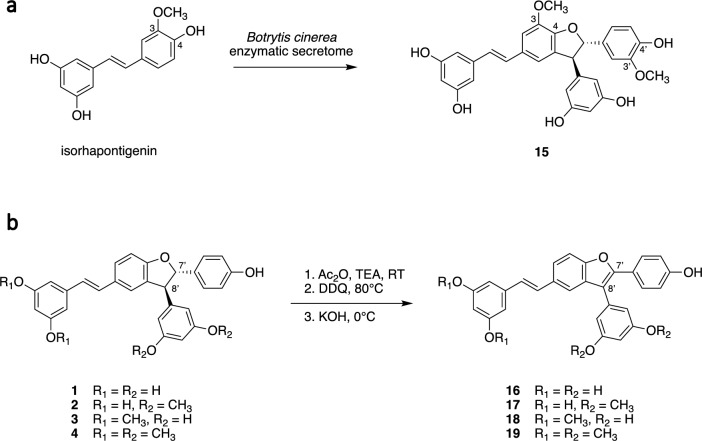
(**a**) Chemoenzymatic synthesis of a given methoxylated derivative of *trans*-δ-viniferin starting from isorhapontigenin, using the enzymatic secretome of *Botrytis cinerea*. (**b**) Synthesis of benzofuran derivatives of *trans*-δ-viniferin.

It is worth mentioning that compound **12** could also be obtained more efficiently by chemoenzymatic synthesis, starting from pinostilbene and using the enzymatic secretome of *B. cinerea* (Fig. S3, and see experimental section on isorhapontigenin). Compound **15** was produced exclusively by chemoenzymatic synthesis (Fig. 3a). Its structure consists of a *trans*-δ-viniferin backbone, methoxylated at C-3 and C-3′. It was obtained by dimerization of a stilbenoid called isorhapontigenin, found for example in several species of the genus *Gnetum*^17,18^. The procedure was similar to that used to obtain compounds **1**–**4** and is described in detail in the experimental section. The reaction generated multiple products, and **15** was the major one, as indicated by the semi-quantitative ELSD detector (Fig. S4a and b) This compound was subsequently isolated (Fig. S4c and d). The ^1^H NMR spectrum of **15** is consistent with a methoxylated *trans*-δ-viniferin at C-3 and C-3′: two resorcinol systems at δ_H_ 6.04 (2H, d, *J* = 2.2 Hz, H-10′, H-14′), 6.09 (1H, t, *J* = 2.2 Hz, H-12′) and 6.11 (1H, t, *J* = 2.1 Hz, H-12), 6.37 (2H, d, *J* = 2.1 Hz, H-10, H-14); two ethylenic protons with a *trans* configuration at δ_H_ 6.87 (1H, d, *J* = 16.3 Hz, H-8), 6.97 (1H, d, *J* = 16.3 Hz, H-7); two *meta* coupled aromatic protons at δ_H_ 6.80 (1H, d, *J* = 1.4 Hz, H-6), 7.17 (1H, d, *J* = 1.4 Hz, H-2); three aromatic protons at δ_H_ 6.76 (2H, m, H-5′, H-6′), 6.95 (1H, s, H-2′); two methines with a *trans* configuration at δ_H_ 4.52 (1H, d, *J* = 8.4 Hz, H-8′), 5.37 (1H, d, *J* = 8.4 Hz, 7′) and two methoxy groups at δ_H_ 3.75 (3H, s, CH_3_O-3′), 3.87 (3H, s, CH_3_O-3). The ROESY correlations from H-2 to CH_3_O-3 and from H-2′ to CH_3_O-3′ confirmed the position of the methoxy groups.

Another series of derivatives was obtained based on the work of Mattio et al*.*^8^. In the case of **1**, they showed that oxidation of the dihydrofuran ring to form a double bond significantly increased the antibacterial activity against *S. aureus* (from an MIC of 35 µM to 4.5 µM for the benzofuran derivative). Since compounds **2** and **3** were shown to have lower MICs than **1** against the same target, it was decided to prepare the corresponding derivatives. The inactive **4** was also included in the experiment in order to obtain more information on the SAR (Fig. 3b).

The procedure was carried out in 3 steps. The first step was a protection step of the phenolic functions. The second was the oxidation, which was performed with DDQ. This step required some optimization, as initial tests in DCM at room temperature did not yield the expected products. Other references in the literature described the reaction in toluene, but with different conditions for similar substrates: from 70 °C, 3 h, 1.2 equivalents of DDQ^19^ to 110 °C, 90 h, 9.5 equivalents of DDQ^20^. The optimal conditions in our case were intermediate: 80 °C, 20 h and 10 equivalents of DDQ. The formation of the target compounds was followed by MS (*m/z* ratio 2 Da lower than that of the starting compounds due to the loss of 2 hydrogen atoms). The final step was a simple hydrolysis to remove the protecting groups on the phenolic moieties, resulting in the target compounds **16**–**19**, which were obtained without further purification. All intermediates were characterized by HRMS and ^1^H-NMR, while compounds **16**–**19** were also further analyzed by 2D NMR and UV. The chromatographic analysis of the different steps is shown in Figs. S5 to S8. The ^1^H NMR spectra of compounds **16**–**19** showed the same spin systems as their corresponding *trans*-δ-viniferin derivatives (**1**–**4**), except that the two methine protons H-7′ and H-8′ from the dihydrofuran ring of **1**–**4** were absent in **16**–**19**. Due to the presence of a double bond between C-7′ and C-8′, some aromatic protons were shifted at lower field in the benzofuran derivatives. For example, H-5 was shifted from δ_H_ 6.89 in **1** to 7.59 in **16**, H-2 from δ_H_ 7.23 to 7.53, H-2′/H-6′ from δ_H_ 7.19 to 7.47, and H-10′/H-14′ from δ_H_ 6.04 to 6.32.

The last set of derivatives was obtained by halogenation of compounds **1**–**4**. The introduction of a carbon-halogen bond is a widely used tool in medicinal chemistry^21^. 25% of small molecule drugs contain at least one halogen atom^22^. Chlorine is the most common halogen, followed by fluorine, bromine and iodine^21^. The introduction of such carbon-halogen bonds can have several effects. The two most important are: (1) an increase in thermal and oxidative stability, which means less susceptibility to oxidation by P450 enzymes, and (2) an increased penetration across biological membranes^23^. The introduction of halogen atoms has the greatest impact, when added to (hetero)aromatic or olefinic moieties. This leads to steric and electronic effects, that can alter the interaction with the amino acids close to the drug target^24^. This approach has been used to improve the biological activity of some natural products^25–30^. In particular, bromination of resveratrol at a specific position resulted in improved activity against *S. aureus*^31^.

The oxidative halogenation performed here was carried out using a sodium halide salt (sodium bromide, sodium chloride and sodium iodide), hydrogen peroxide and acetic acid in an acetonitrile/water solution at 40 °C^26^. Due to the high electronegativity and reactivity of fluorine, fluorination was not possible using this halogenation method.

The halogenation reactions were first optimized on a small scale using only compound **1,** as the four selected compounds were expected to have close reactivities. Several reaction parameters could be varied, such as (1) reaction time, (2) temperature, or (3) amount of halide salt. The temperature and the amount of halide salt were set according to previous work, therefore, the reaction time was defined as the variable parameter. In practice, the bromination, chlorination and iodination reactions were carried out on 4 mg of compound **1** with 1, 100 and 0.5 eq of sodium halide at 40 °C, respectively. The reactions were monitored by UHPLC-PDA-ELSD-MS and MS identified the newly halogenated based on their characteristic isotopic patterns. The aim was to obtain mono- or di-halogenated derivatives, since polyhalogenated compounds are generally described as toxic^32^.

Once satisfactory conditions were obtained for each halogenation reaction, they were carried out with 10 mg of **1, 2, 3** and** 4** using the previously defined reaction times. The 12 reaction mixtures obtained were monitored by UHPLC-PDA-ELSD-MS and their separation was performed using two different stationary phases: C_18_ and phenyl, which allowed the isolation of the 23 pure compounds (Figs. S9 to S11).

**Figure 4:**
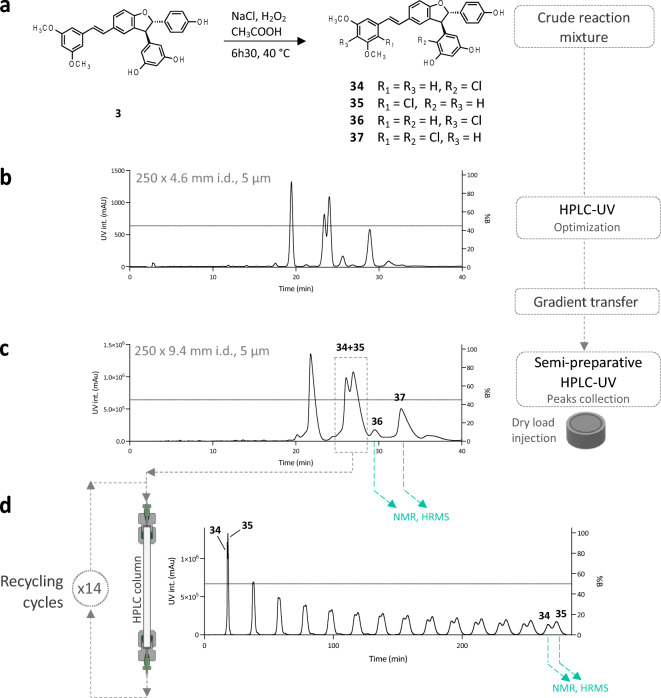
Synthesis and isolation of chlorinated derivatives of *trans*-δ-viniferin derivative **3**. (**a**) Chlorination of compound **3** under optimized conditions yields a crude reaction mixture containing mono- and di-chlorinated derivatives. (**b**) HPLC–UV optimization on a phenyl stationary phase. (**c**) Semi-preparative HPLC–UV run using the conditions optimized at the analytical level. Collection of pure compounds **36** and **37**. (**d**) Semi-preparative HPLC–UV using a recycling valve module to separate compounds **34** and **35**.

**Figure 5:**
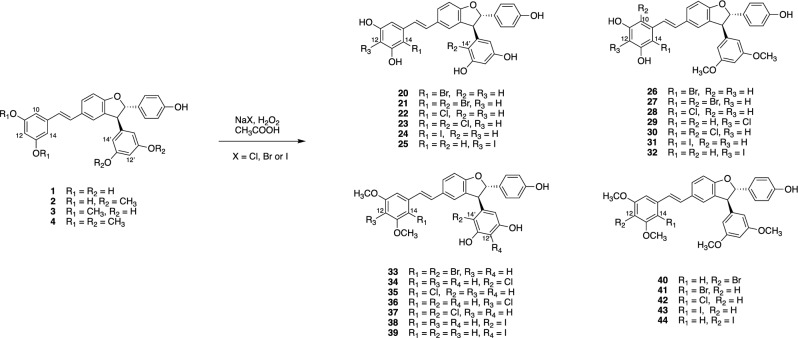
Synthesis of halogenated derivatives of *trans*-δ-viniferin.

A notable case worth mentioning was the chlorination of compound **3**, which gave, among other compounds, two monochlorinated derivatives (**34** and **35**) which were not separated on the C_18_ or phenyl stationary phases (data not shown). After unsuccessful trials on a pentafluorophenyl (PFP) HPLC column, the two compounds were finally separated by semi-preparative HPLC instrument using a recycling valve module^33^. In this technique, the sample can be passed through the same column several times using a connected valve. These “recycle cycles” can be performed repeatedly without consuming solvent or increasing pressure, thus mimicking a longer column. In this case, 14 cycles were used, corresponding to a column length of 3.5 m. After these cycles, the 2 compounds were collected with sufficient purity for structure elucidation and biological testing (Fig. 4). A total of 25 mono- and di-halogenated derivatives of **1, 2, 3** and** 4** were obtained (Fig. 5).

The position of halogenation was deduced from the ^1^H NMR spectra. If the halogenation is at C-14 (equivalent to C-10) or C-14′ (equivalent to C-10′), the resorcinol system consisting of one doublet of 2 protons and a triplet of 1 proton (*J* = 2 Hz) is replaced by two doublets (*J* = 2 Hz). The position of the halogen at C-14 or C-14′ is determined by comparing the chemical shifts of the remaining resorcinol group with those of *trans*-δ-viniferin. For example, in **20**, the chemical shifts of the remaining doublet were observed at δ_H_ 6.04 and those of the triplet at δ_H_ 6.10, corresponding to H-10′/H-14′ and H-12′, respectively in *trans*-δ-viniferin. **20** was therefore identified as 14-bromo-*trans*-δ-viniferin with H-10 and H-12 observed as two doublets at δ_H_ 6.38 and 6.61 (*J* = 2.6 Hz), respectively. When halogenation occurred at C-12, a singlet appeared integrating for 2 protons (H-10/H-14). In the ^13^C or HMBC spectrum, the halogenated carbon was deduced at δ_C_ between 98.2 and 103.6 for bromine, between 107.6 and 112.3 for chlorine, and between 74.1 and 81.9 for iodine.

### Biological screening of the compounds generated

All the generated compounds were tested against a methicillin-susceptible *Staphylococcus aureus* (MSSA) strain (Table 1), except **27**, **36** and **38**, which were not obtained in sufficient quantities. The four starting compounds (**1**–**4**) showed activities against MSSA and methicillin-resistant (MRSA) *S. aureus* strains similar to those reported previously^12,13^. The di-*O*-methylated derivatives **2** and **3** were the most active with a minimum inhibitory concentration (MIC) of about 4 µM.

**Table 1:** Antibacterial activity of the generated compounds, ranked by activity, against the methicillin-susceptible strain of *Staphylococcus aureus* (MSSA) Newman.

Compound	MIC *S. aureus* (µM)	Compound	MIC *S. aureus* (µM)
**27**	ND	**30**	16
**36**	ND	**6**	8–16
**38**	ND	**7**	8–16
**4**	> 64	**9**	8–16
**8**	> 64	**10**	8–16
**19**	> 64	**31**	8–16
**33**	> 64	**11**	8
**40**	> 64	**12**	8
**41**	> 64	**13**	8
**42**	> 64	**14**	8
**43**	> 64	**16**	8
**44**	> 64	**18**	8
**5**	32–64	**17**	4–8
**15**	32–64	**25**	4–8
**1**	16–32	**28**	4–8
**20**	16–32	**2**	4
**21**	16–32	**3**	4
**22**	16–32	**39**	4
**23**	16–32	**37**	2–4
**24**	16–32	**35**	2
**26**	16–32	**34**	1–2
**29**	16–32	Vancomycin^a^	1
**32**	16–32		

^a^Reference compound; *ND *not determined.

As mentioned, the two chemical modifications that most significantly affected the overall shape of the molecule are the *cis* isomerization of the double bond and the oxidation of the furan to a benzofuran ring. *Cis* isomerization of the double bond changes the orientation of one of the aromatic rings in space, while oxidation to benzofuran flattens the structure. *Cis* isomerization kept compound **4** inactive at the highest concentration tested (64 µM), but led to a slight decrease in activity for **1**, **2**, and **3**. As reported by Mattio et al*.*^8^, the benzofuran derivative of *trans*-δ-viniferin (**16**, 8 µM) had a lower MIC than the parent compound (**1**, 16–32 µM). The other benzofuran compounds (**17**, **18**, **19**) showed similar or slightly weaker activities.

The influence of the *O*-methylation pattern was then investigated using compounds **1**–**4**, and **9**–**14**. Compounds with no *O*-methyl group showed the lowest activities (**1**, 16–32 µM), whereas the addition of a single *O*-methyl group led to an increase in activity (8 µM or 8–16 µM, **9**–**11**), regardless of the position. The presence of a second *O*-methyl group further confirmed this tendency [8 µM (**12**–**14**) and 4 µM (**2** and **3**)]. In this case, the presence of the two *O*-methyl groups on the same phenolic ring (**2** and **3**) gave the highest activities. Finally, the presence of four *O*-methyl groups completely eliminated any activity (**4**). According to these results, both the number and the position of the *O*-methyl groups tend to influence the antibacterial activity. Moderate hydrophobicity seems to be the key to activity and could help to cross biological membranes, thus improving the permeation of the compounds, while maintaining a reasonable solubility. The influence of *O*-methyl groups on antibacterial activity has also been previously highlighted for monomeric stilbenes^34^.

Finally, the effect of halogenation was studied. Halogenation of compound **1** led to compounds with the same MIC (16–32 µM, **20**–**24**), regardless of the type of halogen atom (Cl, Br, I) and the number of halogens introduced (one or two). Compound **25**, with an iodine between two *meta*-hydroxy groups, was an exception with a lower MIC against *S. aureus* of 4–8 µM. For compound **2**, halogenation led to a decrease in activity (more or less pronounced) for all the compounds obtained (**26**, **28**–**32)**. Halogenation of **3** led to the most active compounds in the series. Three chlorinated compounds had MICs of 1–2, 2 and 2–4 µM, respectively (**34**, **35** and **37**). Surprisingly, the position of the substitution and the number of chlorine atoms (one or two) did not significantly affect the MIC. It should be noted that replacing the two chlorine atoms of **37** with two bromine atoms (**33**) led to a complete loss of activity. The iodine derivative **39** also had an MIC of 4 µM, the iodine atom being located between two *meta*-hydroxy groups as in compound **25**. Finally, all the halogenated derivatives of compound **4** were still inactive at the concentration tested.

In general, the effect of halogenation on compounds **1**–**4** was very variable, leading to either an increase or a decrease in activity. In contrast to the *O*-methylated derivatives, the activities here were clearly not correlated with polarity and steric and/or electronic effects must be involved, as previously reported^24^. The main conclusion to be drawn from these results is that chlorination allows for the generation of derivatives of compound **3** with slightly enhanced activities.

**Figure 6:**
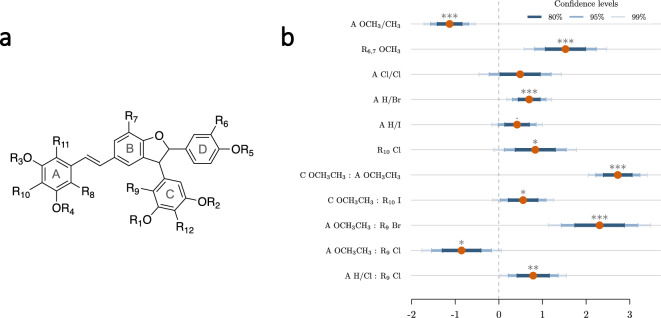
QSAR analysis. (**a**) Maximum common substructure and R_1_ to R_12_ positions. (**b**) Coefficients for each variable of the model and their 95% confidence intervals. The symbols ***, **, * and · correspond to *p* values in the following intervals [0, 0.1%), [0.1%, 1%), [1%, 5%) and [5%, 10%), respectively. For the complete model see Table S1 of supplementary material.

To further investigate the structure–activity relationships (SAR) of the series of analogs, another approach based on a linear regression model was explored. First, the maximum common substructure was first identified, with all selected compounds sharing a common core with a total of 12 positions that can be further substituted (Fig. 6a). Compounds **5**–**8** and **16**–**19** are not included in the analysis as they do not share the same common substructure. To understand which substitutions significantly contribute to activity, we considered a penalized linear regression approach using the log transformation of the MIC as the response variable. Due to the symmetry present in the R groups of rings A and C (Fig. 6a), we considered a single variable to describe R_3_ and R_4_, denoted “A OX” where X is the combination of all possible substituents (CH_3_ and H). Following the same logic, we encoded “C OX” for R_1_ and R_2_ and “A X” for R_8_ and R_11_. The model that best describes the set (R2 = 0.84) includes six R-groups and five second-order interactions between R-groups (Fig. 6b).

This approach suggests that a methyl group at positions R_3_ and R_4_ (A OCH_3_/CH_3_) significantly reduces the log (MIC). This can be seen directly for compounds **1** and **3**, where the sole addition of methyl groups at R_3_ and R_4_ reduces the MIC from 16–32 to 4 µM, respectively. This effect is abolished when R_1_ and R_2_ are also methylated, as shown by the highly significant interaction A OCH_3_/CH_3_:C OCH_3_/CH_3_ and when R_9_ is substituted with a bromine (A OCH_3_/CH_3_:R_9_ Br interaction). It should be noted that the latter is based only on a dibrominated compound (**33**), since the monobrominated compound was not obtained. On the other hand, the simultaneous substitution of R_3_ and R_4_ with a CH_3_ and R_9_ with a chlorine atom tends to decrease the log (MIC) (A OCH_3_/CH_3_:R_9_ Cl), as seen with compound **34** (MIC 1–2 µM), the most active compound obtained.

Positions R_6_ and R_7_ cannot be considered separately from a statistical point of view because both positions are substituted in the only compound (**15**) where these positions have a substituent other than hydrogen. It is interesting to note that the methoxy groups have a negative effect on the log(MIC) (R_6,7_ OCH_3_). This trend can be seen for compounds **1** and **15** where the MIC goes from 16–32 to 32–64 µM. Concerning positions R_8_ or R_11_ of ring A, a single bromine significantly increases the log (MIC), although the effect is minimal. Similarly, a single iodine or double chlorine substitution tends to increase the log (MIC). A single chlorine substitution on both ring A and in position R_9_ also increases the log (MIC) (A H/Cl:R_9_ Cl). Finally, the last significant individual feature is a chlorine atom at position R_10_, which also leads to a decrease in activity.

Taken together, these results emphasize the importance of considering the R-groups together rather than independently. The statistical interactions concern positions that are in spatially distinct locations: they indicate that the correct orientation and filling of the space is necessary for the whole molecule to be active. This statistical model provides the basis for further improvement of the derivatives. For example, the R_3_ and R_4_ positions could be explored with other hydrophobic and/or bulkier groups, as a methyl positively affects the affinity. The effect of halogenation strongly depends on the position but also on the nature of the halogen atom. In this respect, the qualitative observations described above are unfortunately difficult to validate with the model. Further work is needed to generate new derivatives based on the preliminary trends described in this article.

To further validate the interest of the compounds, a subset containing the most active compounds was selected. These compounds were tested against the MRSA strain COL and against a clinical isolate of *Staphylococcus epidermidis* (Table 2). The compounds were equally active against these staphylococcal strains, suggesting a similar mode of action in these gram-positive bacteria.

**Table 2:** Antibacterial activity of a subset of compounds against a methicillin-susceptible (MSSA, Newman) and a methicillin-resistant (MRSA, COL) strain of *Staphylococcus aureus* and a clinical isolate of *Staphylococcus epidermidis*.

Compound	MIC (µM)		
	*S. aureus* Newman (MSSA)	*S. aureus* COL (MRSA)	*S. epidermidis*
**14**	8	4–8	4–8
**17**	4–8	2–4	2–4
**2**	4	4	4–8
**3**	4	4	4
**39**	4	4	2
**37**	2–4	1–2	2
**35**	2	2	1–2
Vancomycin^a^	1	1–2	1

^a^Reference compound.

## Conclusion

The generation of a library of 40 *trans*-δ-viniferin analogues allowed an in-depth study of the modulation of antibacterial activity against *S. aureus* and gave a better understanding of the structure–activity relationships. The high-resolution semi-preparative HPLC–UV methods used provided these compounds with a high degree of purity for biological studies. To our knowledge, this is the first time that the importance of the degree of *O*-methylation for antibacterial activity has been systematically investigated on this scaffold. As expected, the addition of halogen atoms influenced the biological activity. Among the compounds obtained, we highlight the three chlorinated derivatives **34**, **35** and **37**, which showed improved antibacterial activities compared to the parent compound **3**, reaching activities similar to vancomycin. Overall, these results can serve as a starting point for further SAR studies. In addition, the mechanism of action of the most potent compounds is currently being investigated.

## Experimental section

### General experiment procedures

UV and NMR analyses were carried out on the same instruments and under the same conditions as those described in Huber et al. 2022^11^. The UV spectra were recorded on a JASCO J-815 spectrometer (Loveland, CO, USA) in MeOH, using a 1 cm cell. The scan speed was set at 200 nm/min in continuous mode between 500 and 190 nm, with a band width of 1 nm, a data pitch of 0.1 nm and 2 accumulations. NMR spectroscopic data were recorded on a Bruker Avance Neo 600 MHz NMR spectrometer equipped with a QCI 5 mm cryoprobe and a SampleJet automated sample changer (Bruker BioSpin, Rheinstetten, Germany). 1D and 2D NMR experiments (COSY, ROESY, HMBC and HSQC) were recorded in DMSO-*d*_*6*_. Chemical shifts are reported in parts per million (δ) and coupling constants (*J*) in Hz. The residual DMSO-*d*_*6*_ signal (δ_H_ 2.50; δ_C_ 39.5) were used as internal standards for ^1^H and ^13^C NMR, respectively. Iodomethane (CH_3_I), potassium carbonate (K_2_CO_3_), potassium hydroxyde (KOH), acetic anhydride (Ac_2_O), triethylamine (TEA), 2,3-dichloro-5,6-dicyano-p-benzoquinone (DDQ), sodium chloride (NaCl), sodium bromide (NaBr), sodium iodide (NaI), hydrogen peroxide (H_2_O_2_) and acetic acid (CH_3_COOH) were purchased from Sigma-Aldrich (Saint-Louis, MO, USA). Resveratrol, pterostilbene, isorhapontigenin and pinostilbene were purchased from Biopurify (Chengdu, Sichuan, China). The yield for each product is given as an indication, but it is important to note that the strategy followed was to maximise compound diversity rather than yield.

### UHPLC-PDA-ELSD-MS analysis

UHPLC analyses were carried out on the same instrument and under the same conditions as those described in Huber et al. 2022^11^. Analysis of the crude reaction mixtures were carried out on an Ultra-High-Performance Liquid Chromatography system equipped with a PhotoDiode Array, an Evaporative Light-Scattering Detector, and a single quadrupole Mass Spectrometer detector using heated electrospray ionization (UHPLC-PDA-ELSD-MS) (Waters, Milford, MA, USA). The ESI parameters were the following: capillary voltage 800 V, cone voltage 15 V, source temperature 120 °C, and probe temperature 600 °C. Acquisition was done in positive or negative ionization mode with an *m/z* range of 150–1000 Da. The chromatographic separation was performed on an Acquity UPLC BEH C_18_ column (50 × 2.1 mm i.d., 1.7 μm; Waters, Milford, MA, USA) at 0.6 mL/min, 40 °C with H_2_O (A) and MeCN (B) both containing 0.1% formic acid as solvents. The gradient was carried out as follows: 5–100% B in 7 min, 1 min at 100% B, and a re-equilibration step at 5% B for 2 min. The ELSD temperature was fixed at 45 °C, with a gain of 9. The PDA data were acquired from 190 to 500 nm, with a resolution of 1.2 nm. The sampling rate was set at 20 points/s. Every data was processed with the MassLynx software (Waters, Milford, MA, USA).

### UHPLC-PDA-CAD-HRMS analysis

UHPLC-PDA-CAD-HRMS analyses were carried out on the same instrument and under the same conditions as those described in Huber et al. 2022^11^. The pure compounds were analyzed on a Waters Acquity UHPLC system equipped with a Q-Exactive Focus mass spectrometer (Thermo Scientific, Bremen, Germany), using heated electrospray ionization source (HESI-II). The chromatographic separation was carried out on an Acquity UPLC BEH C_18_ column (50 × 2.1 mm i.d., 1.7 μm; Waters) at 0.6 mL/min, 40 °C with H_2_O (A) and MeCN (B) both containing 0.1% formic acid as solvents. The gradient was carried out as follows: 5%–100% B in 7 min, 1 min at 100% B, and a reequilibration step at 5% B in 2 min. The ionization parameters were the same as used in (Rutz et al., 2019).

### Chemoenzymatic synthesis of compounds **1** to **4**

Compound **1** to **4** were obtained by chemoenzymatic synthesis starting from resveratrol and pterostilbene, using the enzymatic secretome of *Botrytis cinerea*, as described in previous articles^11^.

### Light isomerization (compounds **6**–**9**)

Compounds **1**, **2**, **3** and **4** (15 mg each) were placed in solution in DMSO (10 mL) in 50 mL flasks. The flasks were placed under a white light (Fluora T8, L58 W/77, Osram, Munich, Germany). Samples were taken after 15 min, 4, 7, 24, 30, 48 h, 3, 4, 5, 6 and 7 days. Each sample was analyzed by UHPLC-PDA-ELSD-MS and the samples after 7 days were dried for NMR analysis and further purification. Optimizations in view of the purifications were performed on the UHPLC-PDA-ELSD-MS instrument with an Acquity UPLC BEH C_18_ column (100 × 2.1 mm i.d., 1.7 μm; Waters) at 0.5 mL/min, 40 °C with H_2_O (A) and MeCN (B) both containing 0.1% formic acid as solvents. The optimized gradient conditions for each reaction were geometrically transferred by gradient transfer to the HPLC scale (see Table S2, for each chromatographic condition). Semi-preparative injections were run on a Shimadzu system equipped with an LC-20 A module pumps, an SPD-20 A UV/VIS, a 7725I Rheodyne® valve, and an FRC-40 fraction collector (Shimadzu, Kyoto, Japan). The separation was performed on a XBridge C_18_ column (250 mm × 19 mm i.d., 5 μm; Waters) equipped with a C_18_ pre-column cartridge holder (10 mm × 19 mm i.d., 5 μm; Waters) at 17 mL/min, with H_2_O (A) and MeCN (B) both containing 0.1% formic acid as solvents. The UV detection was set at 280 and 320 nm. The mixtures were injected on the semi-preparative HPLC column using a dry load^15^. Fractions were collected, analyzed by UHPLC-PDA-ELSD-MS and combined according to their composition. Compounds were obtained as follows: **5** (1.2 mg, 24% yield, *t*_R_ = 27 min), **6** (2.8 mg, 40% yield, *t*_R_ = 29 min), **7** (4.5 mg, 45% yield, *t*_R_ = 34 min), and **8** (4.6 mg, 51% yield, *t*_R_ = 44 min).

### *O*-methylation (compounds **9**–**14**)

*O*-methylation protocol was adapted from the article published by Delgado and co-workers^16^. 50 mg of compound **1** was solubilized in 10 mL of DMF in a round-bottom flask. 60 mg of K_2_CO_3_ (4 eq) and 62 mg of iodomethane (4 eq) were added under agitation. The mixture was stirred at room temperature overnight. The solvent was removed by rotatory evaporation. The dry deposit was solubilized in MeOH and dried under nitrogen. The crude reaction mixture was analyzed by UHPLC-PDA-ELSD-MS. The optimization in view of the purifications was performed on the UHPLC-PDA-ELSD-MS instrument with an Acquity UPLC BEH C_18_ column (100 × 2.1 mm i.d., 1.7 μm; Waters) at 0.5 mL/min, 40 °C with H_2_O (A) and MeCN (B) both containing 0.1% formic acid as solvents. The optimized gradient condition was found to be an isocratic run at 34% of MeCN. The same isocratic conditions were used on the semi-preparative instrument, by adapting only the flow-rate. The semi-preparative injection was run on a Shimadzu system equipped with an LC-20 A module pumps, an SPD-20 A UV/VIS, a 7725I Rheodyne® valve, and an FRC-40 fraction collector (Shimadzu, Kyoto, Japan). The separation was performed on a XBridge C_18_ column (250 mm × 19 mm i.d., 5 μm; Waters) equipped with a C_18_ pre-column cartridge holder (10 mm × 19 mm i.d., 5 μm; Waters) at 17 mL/min, with H_2_O (A) and MeCN (B) both containing 0.1% formic acid as solvents. The UV detection was set at 280 and 320 nm. The mixtures were injected on the semi-preparative HPLC column using a dry load^15^. The isolation procedures were carried out using the same strategy as described in Huber et al. 2022^11^. Fractions were collected, analyzed by UHPLC-PDA-ELSD-MS and combined according to their composition. Compounds were obtained as follows: **9** (1.4 mg, 3% yield, *t*_R_ = 81 min), **10** (1.2 mg, 2% yield, *t*_R_ = 89 min), **11** (2.4 mg, 5% yield, *t*_R_ = 93 min), **12** (0.7 mg, 1% yield, *t*_R_ = 114 min), **13** (1.0 mg, 2% yield, *t*_R_ = 122 min) and **14** (1.0 mg, 2% yield *t*_R_ = 125 min).

### Chemoenzymatic synthesis starting from isorhapontigenin or pinostilbene (compounds **15** and **12**)

Isorhapontigenin (10 mg) was incubated with the enzymatic secretome of *B. cinerea* (200 µL, 2%), in 8.8 mL of water (88%) and 1 mL of acetone (10%) for 24 h under gentle agitation. The solvent was removed by rotatory evaporation. The dry deposit was solubilized in MeOH and dried under nitrogen. The crude reaction mixture was analyzed by UHPLC-PDA-ELSD-MS. The optimization in view of the purifications was performed on the UHPLC-PDA-ELSD-MS instrument with an Acquity UPLC BEH C_18_ column (100 × 2.1 mm i.d., 1.7 μm; Waters) at 0.5 mL/min, 40 °C with H_2_O (A) and MeCN (B) both containing 0.1% formic acid as solvents. The optimized gradient condition was from 20 to 50% MeCN in 10 min. This was geometrically transferred by gradient transfer to the semi-preparative HPLC scale. The semi-preparative injection was run on a Shimadzu system equipped with an LC-20 A module pumps, an SPD-20 A UV/VIS, a 7725I Rheodyne® valve, and an FRC-40 fraction collector (Shimadzu, Kyoto, Japan). The separation was performed on a XBridge C_18_ column (250 mm × 19 mm i.d., 5 μm; Waters) equipped with a C_18_ pre-column cartridge holder (10 mm × 19 mm i.d., 5 μm; Waters) at 17 mL/min, with H_2_O(A) and MeCN (B) both containing 0.1% formic acid as solvents. The UV detection was set at 210 and 320 nm. The mixtures were injected on the semi-preparative HPLC column using a dry load^15^. The isolation procedures were carried out using the same strategy as described in Huber et al. 2022^11^. Fractions were collected, analyzed by UHPLC-PDA-ELSD-MS and combined according to their composition, leading to compound **15** (1.6 mg, 16% yield, *t*_R_ = 32 min). The same procedure was used with pinostilbene as an alternative method to generate compound **12** (Fig. S3).

### Benzofuran derivatives synthesis (compounds **16**–**19**)

Benzofuran derivative synthesis was performed in 3 steps for each *trans*-δ-viniferin derivative (**1**, **2**, **3** and **4**): (1) protection of the phenolic groups by acetylation (**X-Ac** derivatives), (2) oxidation to benzofuran with DDQ (**X-Ac-Ox** derivatives) and (3) deprotection of the phenolic groups with KOH, leading to compounds **16** (3.9 mg, 23% yield), **17** (5.6 mg, 33% yield), **18** (7.2 mg, 43% yield) and **19** (3.4 mg, 17% yield). The protocol used was based on the work of Mattio et al.^8^. The detailed procedures can be found in the supplementary material.

### Generic halogenation procedure (optimization)

*Trans*-δ-viniferin **1** (4 mg, 0.088 mmol, 1 eq) was solubilized in 1 mL of a MeCN:H_2_O 50:50 mixture in a 5 mL round-bottom flask. The mixture was placed at 40 °C and stirred. 0.5 eq. (NaI), 1 eq. (NaBr) or 100 eq. (NaCl) of halide salt were added, followed by 1 mL of CH_3_COOH and 21 eq. of H_2_O_2_ added dropwise. Samples were regularly collected between 1 and 24 h and analyzed by UHPLC-PDA-ELSD-MS. The detailed procedures for the bromination (compounds **20, 21, 26, 27, 33, 40** and **41**), chlorination (compounds **22**, **23**, **28**, **29**, **30**, **34**, **35 36**, **37** and **42**) and iodination (compounds **24**, **25**, **31**, **32**, **38**, **39**, **43** and **44**) scale-up and isolation can be found in the supplementary material. Supplementary Table S3 summarizes each chromatographic condition.

### Description of the isolated compounds

*trans*-δ-viniferin (**1**): UV (MeCN) λ_max_ (log ε) 227 (sh) (4.46), 285 (4.08), 312 (4.32), 333 (4.127), 350 (sh) (3.99) nm. ^1^H NMR (DMSO-*d*_6_, 600 MHz) δ 4.44 (1H, d, *J* = 7.8 Hz, H-8′), 5.39 (1H, d, *J* = 7.8 Hz, H-7′), 6.03 (2H, d, *J* = 2.1 Hz, H-10′, H-14′), 6.10 (1H, t, *J* = 2.1 Hz, H-12′), 6.11 (1H, t, *J* = 2.2 Hz, H-12), 6.37 (2H, d, *J* = 2.2 Hz, H-10, H-14), 6.76 (2H, d, *J* = 8.6 Hz, H-3′, H-5′), 6.83 (1H, d, *J* = 16.3 Hz, H-8), 6.89 (1H, d, *J* = 8.3 Hz, H-5), 6.98 (1H, d, *J* = 16.3 Hz, H-7), 7.18 (2H, d, *J* = 8.6 Hz, H-2′, H-6′), 7.23 (1H, d, *J* = 1.9 Hz, H-2), 7.42 (1H, dd, *J* = 8.3, 1.9 Hz, H-6), 9.17 (2H, s), 9.21 (2H, s), 9.53 (1H, s, 4′OH). HR-ESI/MS analysis: *m/z* 453.1348 [M-H]^−^, (calcd for C_28_H_21_O_6_^−^, 453.1338, ∆ = 2.2 ppm). MS/MS spectrum: CCMSLIB00009918864. SMILES: OC1=CC=C([C@H](O2)[C@H](C3=CC(O)=CC(O)=C3)C4=C2C=CC(/C=C/C5=CC(O)=CC(O)=C5)=C4)C=C1.

11′,13′-di-*O*-methyl-*trans*-δ-viniferin (**2**): UV (MeCN) λ_max_ (log ε) 227 (sh) (4.45), 285 (4.06), 312 (4.30), 333 (4.25), 350 (sh) (3.97) nm. ^1^H NMR (DMSO-*d*_6_, 600 MHz) δ 3.70 (6H, s, CH_3_O-11′, CH_3_O-13′), 4.58 (1H, d, *J* = 8.1 Hz, H-8′), 5.56 (1H, d, *J* = 8.1 Hz, H-7′), 6.11 (1H, t, *J* = 2.2 Hz, H-12), 6.36 (4H, 2d, *J* = 2.3 Hz, H-10, H-10′, H-14, H-14′), 6.43 (1H, t, *J* = 2.3 Hz, H-12′), 6.76 (2H, d, *J* = 8.7 Hz, H-3′, H-5′), 6.82 (1H, d, *J* = 16.3 Hz, H-8), 6.90 (1H, d, *J* = 8.3 Hz, H-5), 6.97 (1H, d, *J* = 16.3 Hz, H-7), 7.19 (2H, d, *J* = 8.7 Hz, H-2′, H-6′), 7.21 (1H, d, *J* = 1.9 Hz, H-2), 7.43 (1H, dd, *J* = 8.3, 1.9 Hz, H-6), 9.17 (2H, s, 11OH, 13OH), 9.53 (1H, s, 4′OH). HR-ESI/MS analysis: *m/z* 481.1652 [M-H]^−^, (calcd for C_30_H_25_O_6_,^−^ 481.1651, ∆ = 0.2 ppm). MS/MS spectrum: CCMSLIB00009918871. SMILES: OC1=CC=C([C@H](O2)[C@H](C3=CC(OC)=CC(OC)=C3)C4=C2C=CC(/C=C/C5=CC(O)=CC(O)=C5)=C4)C=C1.

11,13-di-*O*-methyl-*trans*-δ-viniferin (**3**): UV (MeCN) λ_max_ (log ε) 227 (sh) (4.48), 285 (4.10), 312 (4.34), 333 (4.30), 350 (sh) (4.00) nm. ^1^H NMR (DMSO-*d*_6_, 600 MHz) δ 3.75 (6H, s, CH_3_O-11, CH_3_O-13), 4.46 (1H, d, *J* = 8.3 Hz, H-8′), 5.41 (1H, d, *J* = 8.3 Hz, H-7′), 6.05 (2H, d, *J* = 2.1 Hz, H-10′, H-14′), 6.11 (1H, t, *J* = 2.2 Hz, H-12′), 6.35 (1H, t, *J* = 2.2 Hz, H-12), 6.73 (2H, d, *J* = 2.2 Hz, H-10, H-14), 6.76 (2H, d, *J* = 8.7 Hz, H-3′, H-5′), 6.90 (1H, d, *J* = 8.3 Hz, H-5), 6.96 (1H, d, *J* = 16.4 Hz, H-8), 7.20 (2H, d, *J* = 8.7 Hz, H-2′, H-6′), 7.22 (1H, d, *J* = 16.4 Hz, H-7), 7.24 (1H, d, *J* = 1.8 Hz, H-2), 7.44 (1H, dd, *J* = 8.3, 1.8 Hz, H-6), 9.22 (2H, s, 11′OH, 13′OH), 9.53 (1H, s, 4′OH). HR-ESI/MS analysis: *m/z* 481.1654 [M-H]^−^, (calcd for C_30_H_25_O_6_,^−^ 481.1651, ∆ = 0.6 ppm). MS/MS spectrum: CCMSLIB00009918873. SMILES: OC(C=C1)=CC=C1[C@@H](O2)[C@@H](C3=CC(O)=CC(O)=C3)C4=C2C=CC(/C=C/C5=CC(OC)=CC(OC)=C5)=C4.

11,11′,13,13′-tetra-*O*-methyl-*trans*-δ-viniferin (**4**): UV (MeCN) λ_max_ (log ε) 227 (sh) (4.56), 285 (4.17), 311 (4.41), 333 (4.37), 350 (sh) (4.12) nm. ^1^H NMR (DMSO-*d*_6_, 600 MHz) δ 3.63 (6H, s, CH_3_O-11′, CH_3_O-13′), 3.67 (6H, s, CH_3_O-11, CH_3_O-13), 4.54 (1H, d, *J* = 8.5 Hz, H-8′), 5.52 (1H, d, *J* = 8.5 Hz, H-7′), 6.28 (1H, t, *J* = 2.3 Hz, H-12), 6.31 (2H, d, *J* = 2.3 Hz, H-10′, H-14′), 6.36 (1H, t, *J* = 2.3 Hz, H-12′), 6.65 (2H, d, *J* = 2.3 Hz, H-10, H-14), 6.69 (2H, d, *J* = 8.6 Hz, H-3′, H-5′), 6.85 (1H, d, *J* = 8.3 Hz, H-5), 6.88 (1H, d, *J* = 16.4 Hz, H-8), 7.12 (1H, d, *J* = 1.8 Hz, H-2), 7.13 (2H, d, *J* = 8.6 Hz, H-2′, H-6′), 7.14 (1H, d, *J* = 16.4 Hz, H-7), 7.38 (1H, dd, *J* = 8.3, 1.8 Hz, H-6), 9.49 (1H, s, 4′OH). HR-ESI/MS analysis: *m/z* 509.1968 [M-H]^−^, (calcd for C_32_H_29_O_6_^−^, 509.1964, ∆ = 0.8 ppm). MS/MS spectrum: CCMSLIB00009918877. SMILES: OC(C=C1)=CC=C1[C@@H](O2)[C@@H](C3=CC(OC)=CC(OC)=C3)C4=C2C=CC(/C=C/C5=CC(OC)=CC(OC)=C5)=C4.

*cis*-δ-viniferin (**5**): UV (MeOH) λ_max_ (log ε) 229 (sh) (4.53), 287 (4.14), 300 (sh) (4.09) nm; ^1^H NMR (DMSO-*d*_6_, 600 MHz) δ 4.38 (1H, d, *J* = 8.5 Hz, H-8′), 5.35 (1H, d, *J* = 8.5 Hz, H-7′), 5.99 (2H, d, *J* = 2.4 Hz, H-10′, H-14′), 6.06 (1H, d, *J* = 2.5 Hz, H-12), 6.08 (1H, d, *J* = 2.4 Hz, H-12′), 6.13 (2H, d, *J* = 2.5 Hz, H-10, H-14), 6.28 (1H, d, *J* = 12.4 Hz, H-8), 6.39 (1H, d, *J* = 12.4 Hz, H-7), 6.74 (1H, d, *J* = 8.3 Hz, H-5), 6.75 (2H, d, *J* = 8.8 Hz, H-3′, H-5′), 6.88 (1H, s, H-2), 7.13 (1H, dd, *J* = 8.3, 1.9 Hz, H-6), 7.17 (2H, d, *J* = 8.8 Hz, H-2′, H-6′), 9.13 (2H, s, 11OH, 13OH), 9.19 (2H, s, 11′OH, 13′OH), 9.53 (1H, s, 4′OH); ^13^C NMR (DMSO-*d*_6_, 151 MHz) δ 55.7 (C-8′), 92.4 (C-7′), 101.3 (C-12′), 101.6 (C-12), 105.9 (C-10′, C-14′), 106.3 (C-10, C-14), 108.8 (C-5), 115.3 (C-3′, C-5′), 126.0 (C-2), 127.9 (C-2′, C-6′), 128.4 (C-8), 128.8 (C-6), 129.3 (C-7), 129.6 (C-1), 130.2 (C-1′), 130.4 (C-3), 138.9 (C-9), 143.4 (C-9′), 157.5 (C-4′), 158.2 (C-4), 158.3 (C-11, C-13), 158.6 (C-11′, C-13′). HR-ESI/MS analysis: *m/z* 453.1356 [M-H]^−^, (calcd for C_28_H_21_O_6_^−^, 453.1344 , ∆ = 2.7 ppm). MS/MS spectrum: CCMSLIB00010129236. SMILES: OC(C=C1)=CC=C1[C@@H](O2)[C@@H](C3=CC(O)=CC(O)=C3)C4=C2C=CC(/C=C\C5=CC(O)=CC(O)=C5)=C4.

11′,13′-di-*O*-methyl-*cis*-δ-viniferin (**6**): UV (MeOH) λ_max_ (log ε) 227 (sh) (4.55), 284 (4.17), 298 (sh) (4.12) nm; ^1^H NMR (DMSO-*d*_6_, 600 MHz) δ 3.68 (6H, s, CH_3_O-11′, CH_3_O-13′), 4.52 (1H, d, *J* = 8.8 Hz, H-8′), 5.54 (1H, d, *J* = 8.8 Hz, H-7′), 6.04 (1H, t, *J* = 2.2 Hz, H-12), 6.09 (2H, d, *J* = 2.2 Hz, H-10, H-14), 6.28 (1H, d, *J* = 12.3 Hz, H-8), 6.30 (2H, d, *J* = 2.3 Hz, H-10′, H-14′), 6.39 (1H, t, *J* = 2.3 Hz, H-12′), 6.39 (1H, d, *J* = 12.3 Hz, H-7), 6.75 (3H, d, *J* = 8.5 Hz, H-3′, H-5′), 6.77 (1H, d, *J* = 8.4 Hz, H-5), 6.85 (1H, t, *J* = 1.4 Hz, H-2), 7.12 (1H, dd, *J* = 8.4, 1.6 Hz, H-6), 7.19 (2H, d, *J* = 8.5 Hz, H-2′, H-6′), 9.09 (2H, s, 11OH, 13OH), 9.53 (1H, s, 4′OH); ^13^C NMR (DMSO-*d*_6_, 151 MHz) δ 55.1 (CH_3_O-11′, CH_3_O-13′), 55.7 (C-8′), 91.7 (C-7′), 98.6 (C-12′), 101.5 (C-12), 106.1 (C-10′, C-14′), 106.3 (C-10, C-14), 109.0 (C-5), 115.3 (C-3′, C-5′), 125.5 (C-2), 127.9 (C-2′, C-6′), 128.6 (C-8), 129.1 (C-6), 129.3 (C-7), 129.8 (C-1), 129.9 (C-1′), 130.5 (C-3), 138.8 (C-9), 143.2 (C-9′), 157.6 (C-4′), 158.1 (C-4), 158.3 (C-11, C-13), 160.7 (C-11′, C-13′); HR-ESI/MS analysis: *m/z* 481.1665 [M-H]^−^, (calcd for C_30_H_25_O_6_^−^, 481.1657 , ∆ = 1.7 ppm). MS/MS spectrum: CCMSLIB00010129237. SMILES: OC(C=C1)=CC=C1[C@@H](O2)[C@@H](C3=CC(OC)=CC(OC)=C3)C4=C2C=CC(/C=C\C5=CC(O)=CC(O)=C5)=C4.

11,13-di-*O*-methyl-*cis*-δ-viniferin (**7**): UV (MeOH) λ_max_ (log ε) 227 (sh) (4.55), 285 (4.15), 300 (4.09) nm; ^1^H NMR (DMSO-*d*_6_, 600 MHz) δ 3.60 (6H, s, CH_3_O-11, CH_3_O-13), 4.34 (1H, d, *J* = 7.7 Hz, H-8′), 5.39 (1H, d, *J* = 7.7 Hz, H-7′), 5.98 (2H, d, *J* = 2.2 Hz, H-10′, H-14′), 6.08 (1H, t, *J* = 2.2 Hz, H-12′), 6.31 (1H, t, *J* = 2.3 Hz, H-12), 6.38 (3H, d, *J* = 2.3 Hz, H-10, H-14), 6.40 (1H, d, *J* = 12.2 Hz, H-8), 6.52 (1H, d, *J* = 12.2 Hz, H-7), 6.74 (2H, d, *J* = 8.6 Hz, H-3′, H-5′), 6.80 (1H, d, *J* = 8.4 Hz, H-5), 6.91 (1H, d, *J* = 1.8 Hz, H-2), 7.11 (1H, dd, *J* = 8.4, 1.8 Hz, H-6), 7.15 (2H, d, *J* = 8.6 Hz, H-2′, H-6′), 9.18 (2H, s, 11′OH, 13′OH), 9.52 (1H, s, 4′OH); ^13^C NMR (DMSO-*d*_6_, 151 MHz) δ 54.9 (CH_3_O-11, CH_3_O-13), 55.7 (C-8′), 92.2 (C-7′), 99.5 (C-12), 101.3 (C-12′), 105.8 (C-10′, C-14′), 106.3 (C-10, C-14), 108.9 (C-5), 115.3 (C-3′, C-5′), 125.5 (C-2), 127.7 (C-2′, C-6′), 128.2 (C-8), 129.3 (C-6), 129.5 (C-1), 130.3 (C-7), 130.4 (C-1′), 130.7 (C-3), 138.8 (C-9), 143.6 (C-9′), 157.5 (C-4′), 158.4 (C-4), 158.6 (C-11′, C-13′), 160.2 (C-11, C-13); HR-ESI/MS analysis: *m/z* 481.1666 [M-H]^−^, (calcd C_30_H_25_O_6_^−^, 481.1657 , ∆ = 1.9 ppm). MS/MS spectrum: CCMSLIB00010129238. SMILES: OC(C=C1)=CC=C1[C@@H](O2)[C@@H](C3=CC(O)=CC(O)=C3)C4=C2C=CC(/C=C\C5=CC(OC)=CC(OC)=C5)=C4.

11,11′,13,13′-tetra-*O*-methyl-*cis*-δ-viniferin (**8**): UV (MeOH) λ_max_ (log ε) 227 (sh) (4.57), 285 (4.17), 300 (4.11) nm; ^1^H NMR (DMSO-*d*_6_, 600 MHz) δ 3.58 (6H, s, CH_3_O-11, CH_3_O-13), 3.67 (6H, s, CH_3_O-11′, CH_3_O-13′), 4.48 (1H, d, *J* = 8.0 Hz, H-8′), 5.57 (1H, d, *J* = 8.0 Hz, H-7′), 6.29 (3H, d, *J* = 2.2 Hz, H-10′, H-12, H-14′), 6.33 (2H, d, *J* = 2.2 Hz, H-10, H-14), 6.38 (1H, t, *J* = 2.2 Hz, H-12′), 6.40 (1H, d, *J* = 12.1 Hz, H-8), 6.52 (1H, d, *J* = 12.1 Hz, H-7), 6.74 (2H, d, *J* = 8.2 Hz, H-3′, H-5′), 6.82 (1H, d, *J* = 8.2 Hz, H-5), 6.86 (1H, d, *J* = 1.8 Hz, H-2), 7.10 (1H, dd, *J* = 8.2, 1.8 Hz, H-6), 7.16 (2H, d, *J* = 8.2 Hz, H-2′, H-6′), 9.53 (1H, s, 4′OH); ^13^C NMR (DMSO-*d*_6_, 151 MHz) δ 54.9 (CH_3_O-11, CH_3_O-13), 55.1 (CH_3_O-11′, CH_3_O-13′), 55.5 (C-8′), 91.5 (C-7′), 98.5 (C-12′), 99.3 (C-12), 105.9 (C-10′, C-14′), 106.2 (C-10, C-14), 109.0 (C-5), 115.2 (C-3′, C-5′), 125.1 (C-2), 127.7 (C-2′, C-6′), 128.2 (C-8), 129.5 (C-6), 129.6 (C-1), 130.2 (C-1′), 130.2 (C-7), 130.7 (C-3), 138.8 (C-9), 143.5 (C-9′), 157.5 (C-4′), 158.1 (C-4), 160.2 (C-11, C-13), 160.6 (C-11′, C-13′); HR-ESI/MS analysis: *m/z* 509.1982 [M-H]^−^, (calcd for C_32_H_29_O_6_^−^, 509.1970 , ∆ = 2.4 ppm). MS/MS spectrum: CCMSLIB00010129239. SMILES: OC(C=C1)=CC=C1[C@@H](O2)[C@@H](C3=CC(OC)=CC(OC)=C3)C4=C2C=CC(/C=C\C5=CC(OC)=CC(OC)=C5)=C4.

11′-*O*-methyl-*trans*-δ-viniferin (**9**): UV (MeOH) λ_max_ (log ε) 228 (sh) (4.50), 285 (4.09), 310 (4.27), 330 (4.23) nm; ^1^H NMR (DMSO-*d*_6_, 600 MHz) δ 3.65 (3H, s, CH_3_O-11′), 4.51 (1H, d, *J* = 7.9 Hz, H-8′), 5.45 (1H, d, *J* = 7.9 Hz, H-7′), 6.11 (1H, t, *J* = 2.2 Hz, H-12), 6.18 (1H, t, *J* = 1.8 Hz, H-14′), 6.20 (1H, t, *J* = 1.8 Hz, H-10′), 6.24 (1H, t, *J* = 1.8 Hz, H-12′), 6.37 (2H, d, *J* = 2.2 Hz, H-10, H-14), 6.76 (2H, d, *J* = 8.6 Hz, H-3′, H-5′), 6.83 (1H, d, *J* = 16.3 Hz, H-8), 6.89 (1H, d, *J* = 8.3 Hz, H-5), 6.98 (1H, d, *J* = 16.3 Hz, H-7), 7.18 (2H, d, *J* = 8.6 Hz, H-2′, H-6′), 7.22 (1H, d, *J* = 1.9 Hz, H-2), 7.42 (1H, dd, *J* = 8.3, 1.9 Hz, H-6), 9.17 (2H, s, 11OH, 13OH), 9.43 (1H, s, 13′OH), 9.53 (1H, s, 4′OH); ^13^C NMR (DMSO-*d*_6_, 151 MHz) δ 54.9 (CH_3_O-11′), 55.8 (C-8′), 92.4 (C-7′), 99.8 (C-12′), 101.9 (C-12), 104.5 (C-10, C-14), 105.0 (C-10′), 107.1 (C-14′), 109.4 (C-5), 115.2 (C-3′, C-5′), 122.8 (C-2), 126.3 (C-8), 127.7 (C-6), 127.8 (C-7), 127.9 (C-2′, C-6′), 130.4 (C-1, C-1′), 131.0 (C-3), 139.1 (C-9), 143.9 (C-9′), 157.6 (C-4′), 158.5 (C-11, C-13), 158.9 (C-4, C-13′), 160.6 (C-11′); HR-ESI/MS analysis: 467.1498 m*/z* [M-H]^−^, (calcd for C_29_H_23_O_6_^−^, 467.1500 , ∆ = 0.5 ppm). MS/MS spectrum: CCMSLIB00010129240. SMILES: OC(C=C1)=CC=C1[C@@H](O2)[C@@H](C3=CC(O)=CC(OC)=C3)C4=C2C=CC(/C=C/C5=CC(O)=CC(O)=C5)=C4.

11-*O*-methyl-*trans*-δ-viniferin (**10**): UV (MeOH) λ_max_ (log ε) 227 (sh) (4.44), 288 (4.05), 310 (4.20), 330 (4.17) nm; ^1^H NMR (DMSO-*d*_6_, 600 MHz) δ 3.70 (3H, s, CH_3_O-11), 4.45 (1H, d, *J* = 8.1 Hz, 8′), 5.40 (1H, d, *J* = 8.1 Hz, 7′), 6.04 (2H, d, *J* = 2.2 Hz, 10′, 14′), 6.10 (1H, t, *J* = 2.2 Hz, 12′), 6.20 (1H, t, *J* = 2.0 Hz, H-12), 6.52 (1H, t, *J* = 2.0 Hz, H-14), 6.59 (1H, t, *J* = 2.0 Hz, H-10), 6.76 (2H, d, *J* = 8.6 Hz, 3′, 5′), 6.89 (1H, d, *J* = 8.3 Hz, H-5), 6.90 (1H, d, *J* = 16.4 Hz, H-8), 7.10 (1H, d, *J* = 16.4 Hz, H-7), 7.19 (2H, d, *J* = 8.6 Hz, 2′, 6′), 7.23 (1H, d, *J* = 1.8 Hz, H-2), 7.43 (1H, dd, *J* = 8.3, 1.8 Hz, H-6), 9.21 (2H, s, 11′OH, 13′OH), 9.37 (1H, s, 13OH), 9.53 (1H, s, 4′OH); ^13^C NMR (DMSO-*d*_6_, 151 MHz) δ 54.9 (CH_3_O-11), 55.8 (C-8′), 92.7 (C-7′), 100.6 (C-12), 101.3 (C-12′), 102.6 (C-10), 105.9 (C-14), 106.1 (C-10′, C-14′), 109.3 (C-5), 115.2 (C-3′, C-5′), 123.0 (C-2), 126.0 (C-8), 127.7 (C-6), 127.9 (C-2′, C-6′), 128.4 (C-7), 130.4 (C-1, C-1′), 131.3 (C-3), 139.4 (C-9), 143.6 (C-9′), 157.6 (C-4′), 158.5 (C-13), 158.7 (C-11′, C-13′), 158.9 (C-4), 160.5 (C-11); HR-ESI/MS analysis: *m/z* 467.1507 [M-H]^−^, (calcd for C_29_H_23_O_6_^−^, 467.1500, ∆ = 1.5 ppm). MS/MS spectrum: CCMSLIB00010129241. SMILES: OC(C=C1)=CC=C1[C@@H](O2)[C@@H](C3=CC(O)=CC(O)=C3)C4=C2C=CC(/C=C/C5=CC(O)=CC(OC)=C5)=C4.

4′-*O*-methyl-*trans*-δ-viniferin (**11**): UV (MeOH) λ_max_ (log ε) 226 (sh) (4.48), 286 (4.09), 310 (4.29), 329 (4.26) nm; ^1^H NMR (DMSO-*d*_6_, 600 MHz) δ 3.76 (3H, s, CH_3_O-4′), 4.45 (1H, d, *J* = 7.9 Hz, H-8′), 5.45 (1H, d, *J* = 7.9 Hz, H-7′), 6.04 (2H, d, *J* = 2.2 Hz, H-10′, H-14′), 6.11 (2H, 2xt, *J* = 2.2 Hz, H-12, H-12′), 6.37 (2H, d, *J* = 2.2 Hz, H-10, H-14), 6.83 (1H, d, *J* = 16.3 Hz, H-8), 6.91 (1H, d, *J* = 8.3 Hz, H-5), 6.94 (2H, d, *J* = 8.6 Hz, H-3′, H-5′), 6.98 (1H, d, *J* = 16.3 Hz, H-7), 7.23 (1H, t, *J* = 1.4 Hz, H-2), 7.30 (2H, d, *J* = 8.6 Hz, H-2′, H-6′), 7.43 (1H, dd, *J* = 8.3, 1.9 Hz, H-6), 9.17 (2H, s, 11OH, 13OH), 9.22 (2H, s, 11′OH, 13′OH); ^13^C NMR (DMSO-*d*_6_, 151 MHz) δ 55.1 (CH_3_O-4′), 55.9 (C-8′), 92.3 (C-7′), 101.4 (C-12′), 101.9 (C-12), 104.4 (C-10, C-14), 106.0 (C-10′, C-14′), 109.4 (C-5), 114.0 (C-3′, C-5′), 122.9 (C-2), 126.4 (C-8), 127.7 (C-6 or C-7), 127.8 (C-7 or C-6), 127.8 (C-2′, C-6′), 130.5 (C-1), 131.1 (C-3), 132.2 (C-1′), 139.1 (C-9), 143.6 (C-9′), 158.4 (C-11, C-13), 158.7 (C-11′, C-13′), 158.9 (C-4), 159.3 (C-4′); HR-ESI/MS analysis: *m/z* 467.1509 [M-H]^−^, (calcd for C_29_H_23_O_6_^−^, 467.1500, ∆ = 1.9 ppm). MS/MS spectrum: CCMSLIB00010129242. SMILES: OC1=CC([C@H]([C@@H](C2=CC=C(OC)C=C2)O3)C4=C3C=CC(/C=C/C5=CC(O)=CC(O)=C5)=C4)=CC(O)=C1.

11,11′-di-*O*-methyl-*trans*-δ-viniferin (**12**): UV (MeOH) λ_max_ (log ε) 226 (sh) (4.57), 285 (4.18), 312 (4.42), 330 (4.40) nm; ^1^H NMR (DMSO-*d*_6_, 600 MHz) δ 3.65 (3H, s, CH_3_O-11′), 3.70 (3H, s, CH_3_O-11), 4.52 (1H, d, *J* = 8.1 Hz, H-8′), 5.47 (1H, d, *J* = 8.1 Hz, H-7′), 6.19 (1H, t, *J* = 2.0 Hz, H-14′), 6.20 (1H, t, *J* = 2.0 Hz, H-12), 6.21 (1H, t, *J* = 2.0 Hz, H-10′), 6.25 (1H, t, *J* = 2.0 Hz, H-12′), 6.52 (1H, t, *J* = 2.0 Hz, H-14), 6.58 (1H, t, *J* = 2.0 Hz, H-10), 6.76 (2H, d, *J* = 8.7 Hz, H-3′, H-5′), 6.90 (1H, d, *J* = 16.3 Hz, H-8), 6.90 (1H, d, *J* = 8.4 Hz, H-5), 7.10 (1H, d, *J* = 16.3 Hz, H-7), 7.19 (2H, d, *J* = 8.7 Hz, H-2′, H-6′), 7.22 (1H, t, *J* = 1.4 Hz, H-2), 7.44 (1H, dd, *J* = 8.4, 1.9 Hz, H-6), 9.37 (1H, s, 13OH), 9.43 (1H, s, 13′OH), 9.53 (1H, s, 4′OH); ^13^C NMR (DMSO-*d*_6_, 151 MHz) δ 54.9 (CH_3_O-11′), 54.9 (CH_3_O-11), 55.7 (C-8′), 92.4 (C-7′), 99.7 (C-12′), 100.6 (C-12), 102.5 (C-10), 105.1 (C-10′), 106.0 (C-14), 107.2 (C-14′), 109.4 (C-5), 115.3 (C-3′, C-5′), 122.9 (C-2), 126.0 (C-8), 127.7 (C-6), 127.9 (C-2′, C-6′), 128.3 (C-7), 130.2 (C-1′), 130.4 (C-1), 131.3 (C-3), 139.3 (C-9), 143.8 (C-9′), 157.6 (C-4′), 158.5 (C-13), 158.8 (C-13′), 158.9 (C-4), 160.6 (C-11), 160.6 (C-11′); HR-ESI/MS analysis: *m/z* 481.1662 [M-H]^−^, (calcd for C_30_H_25_O_6_^−^, 481.1657, ∆ = 1.1 ppm). MS/MS spectrum: CCMSLIB00010129245. SMILES: OC(C=C1)=CC=C1[C@@H](O2)[C@@H](C3=CC(O)=CC(OC)=C3)C4=C2C=CC(/C=C/C5=CC(OC)=CC(O)=C5)=C4.

4′,11′-di-*O*-methyl-*trans*-δ-viniferin (**13**): UV (MeOH) λ_max_ (log ε) 228 (sh) (4.54), 285 (4.10), 310 (4.28), 331 (4.22) nm; ^1^H NMR (DMSO-*d*_6_, 600 MHz) δ 3.66 (3H, s, CH_3_O-11′), 3.76 (3H, s, CH_3_O-4′), 4.52 (1H, d, *J* = 7.9 Hz, H-8′), 5.52 (1H, d, *J* = 7.9 Hz, H-7′), 6.11 (1H, t, *J* = 2.2 Hz, H-12), 6.19 (1H, t, *J* = 2.0 Hz, H-14′), 6.21 (1H, t, *J* = 2.0 Hz, H-10′), 6.25 (1H, t, *J* = 2.0 Hz, H-12′), 6.37 (2H, d, *J* = 2.2 Hz, H-10, H-14), 6.83 (1H, d, *J* = 16.4 Hz, H-8), 6.92 (1H, d, *J* = 8.3 Hz, H-5), 6.94 (2H, d, *J* = 8.7 Hz, H-3′, H-5′), 6.98 (1H, d, *J* = 16.4 Hz, H-7), 7.22 (1H, t, *J* = 1.5 Hz, H-2), 7.31 (2H, d, *J* = 8.7 Hz, H-2′, H-6′), 7.43 (1H, dd, *J* = 8.3, 1.9 Hz, H-6), 9.17 (2H, s, 11OH, 13OH), 9.43 (1H, s, 13′OH); ^13^C NMR (DMSO-*d*_6_, 151 MHz) δ 54.9 (CH_3_O-11′), 55.1 (CH_3_O-4′), 55.9 (C-8′), 92.0 (C-7′), 99.7 (C-12′), 101.9 (C-12), 104.4 (C-10, C-14), 105.0 (C-10′), 107.2 (C-14′), 109.4 (C-5), 114.0 (C-3′, C-5′), 122.9 (C-2), 126.4 (C-8), 127.7 (C-2′, C-6, C-6′, C-7), 130.5 (C-1), 131.1 (C-3), 132.1 (C-1′), 139.1 (C-9), 143.7 (C-9′), 158.4 (C-11, C-13), 158.8 (C-13′), 158.8 (C-4), 159.3 (C-4′), 160.6 (C-11′). HR-ESI/MS analysis: *m/z* 481.1662 [M-H]^−^, (calcd for C_30_H_25_O_6_^−^, 481.1657 , ∆ = 1.1 ppm). MS/MS spectrum: CCMSLIB00010129243. SMILES: OC1=CC(OC)=CC([C@H]([C@@H](C2=CC=C(OC)C=C2)O3)C4=C3C=CC(/C=C/C5=CC(O)=CC(O)=C5)=C4)=C1.

4′,11-di-*O*-methyl-*trans*-δ-viniferin (**14**): UV (MeOH) λ_max_ (log ε) 228 (sh) (4.65), 285 (4.21), 310 (4.39), 329 (4.36) nm; ^1^H NMR (DMSO-*d*_6_, 600 MHz) δ 3.70 (3H, s, CH_3_O-11), 3.76 (3H, s, CH_3_O-4′), 4.46 (1H, d, *J* = 8.1 Hz, H-8′), 5.46 (1H, d, *J* = 8.1 Hz, H-7′), 6.04 (2H, d, *J* = 2.2 Hz, H-10′, H-14′), 6.11 (1H, t, *J* = 2.2 Hz, H-12′), 6.20 (1H, t, *J* = 2.0 Hz, H-12), 6.52 (1H, t, *J* = 2.0 Hz, H-14), 6.59 (1H, t, *J* = 2.0 Hz, H-10), 6.90 (1H, d, *J* = 16.4 Hz, H-8), 6.92 (1H, d, *J* = 8.3 Hz, H-5), 6.95 (2H, d, *J* = 8.7 Hz, H-3′, H-5′), 7.10 (1H, d, *J* = 16.4 Hz, H-7), 7.24 (1H, t, *J* = 1.4 Hz, H-2), 7.31 (2H, d, *J* = 8.7 Hz, H-2′, H-6′), 7.44 (1H, dd, *J* = 8.3, 1.9 Hz, H-6), 9.22 (2H, s, 11′OH, 13′OH), 9.37 (1H, s, 13OH); ^13^C NMR (DMSO-*d*_6_, 151 MHz) δ 54.9 (CH_3_O-11), 55.1 (CH_3_O-4′), 55.9 (C-8′), 92.3 (C-7′), 100.6 (C-12), 101.4 (C-12′), 102.5 (C-10), 105.9 (C-14), 106.0 (C-10′, C-14′), 109.4 (C-5), 114.0 (C-3′, C-5′), 123.0 (C-2), 126.0 (C-8), 127.7 (C-6), 127.8 (C-2′, C-6′), 128.3 (C-7), 130.4 (C-1), 131.2 (C-3), 132.0 (C-1′), 139.3 (C-9), 143.5 (C-9′), 158.5 (C-13), 158.7 (C-11′, C-13′), 158.9 (C-4), 159.3 (C-4′), 160.6 (C-11); HR-ESI/MS analysis: *m/z* 481.1646 [M-H]^−^, (calcd for C_30_H_25_O_6_^−^, 481.1657, ∆ = 2.2 ppm). MS/MS spectrum: CCMSLIB00010129244. SMILES: OC1=CC([C@H]([C@@H](C2=CC=C(OC)C=C2)O3)C4=C3C=CC(/C=C/C5=CC(OC)=CC(O)=C5)=C4)=CC(O)=C1.

5,5′-dimethoxy-*trans*-δ-viniferin (**15**): UV (MeOH) λ_max_ (log ε) 229 (sh) (4.44), 288 (4.07), 312 (4.21), 332 (4.24), 348 (sh) (4.05) nm; ^1^H NMR (DMSO-*d*_6_, 600 MHz) δ 3.75 (3H, s, CH_3_O-3′), 3.87 (3H, s, CH_3_O-3), 4.52 (1H, d, *J* = 8.4 Hz, H-8′), 5.37 (1H, d, *J* = 8.4 Hz, 7′), 6.04 (2H, d, *J* = 2.2 Hz, H-10′, H-14′), 6.09 (1H, t, *J* = 2.2 Hz, H-12′), 6.11 (1H, t, *J* = 2.1 Hz, H-12), 6.37 (2H, d, *J* = 2.1 Hz, H-10, H-14), 6.76 (2H, m, H-5′, H-6′), 6.80 (1H, d, *J* = 1.4 Hz, H-6), 6.87 (1H, d, *J* = 16.3 Hz, H-8), 6.95 (1H, s, H-2′), 6.97 (1H, d, *J* = 16.3 Hz, H-7), 7.17 (1H, d, *J* = 1.4 Hz, H-2), 9.09 (1H, s, 4′OH), 9.17 (2H, s, 11OH, 13OH), 9.20 (2H, s, 11′OH, 13′OH); ^13^C NMR (DMSO-*d*_6_, 151 MHz) δ 55.6 (CH_3_O-3′), 55.8 (CH_3_O-3), 55.9 (C-8′), 93.1 (C-7′), 101.3 (C-12′), 101.9 (C-12), 104.4 (C-10, C-14), 106.1 (C-10′, C-14′), 110.3 (C-2), 110.8 (C-2′), 115.3 (C-5′), 115.6 (C-6), 119.4 (C-6′), 126.6 (C-8), 128.1 (C-7), 130.5 (C-1′), 131.2 (C-1), 131.8 (C-5), 139.2 (C-9), 143.5 (C-9′), 144.0 (C-3), 146.8 (C-4′), 147.4 (C-4), 147.6 (C-3′), 158.4 (C-11, C-13), 158.6 (C-11′, C-13′); HR-ESI/MS analysis: *m/z* 513.1548 [M-H]^−^, (calcd for C_30_H_25_O_8_^−^, 513.1555 , ∆ = 1.3 ppm). MS/MS spectrum: CCMSLIB00010129246. SMILES: OC(C=C1)=C(OC)C=C1[C@@H](O2)[C@@H](C3=CC(O)=CC(O)=C3)C4=C2C(OC)=CC(/C=C/C5=CC(O)=CC(O)=C5)=C4.

dehydro-*trans*-δ-viniferin (**16**): UV (MeOH) λ_max_ (log ε) 307 (4.34) nm; ^1^H NMR (DMSO-*d*_6_, 600 MHz) δ 6.14 (1H, t, *J* = 2.1 Hz, H-12), 6.27 (1H, t, *J* = 2.2 Hz, H-12′), 6.32 (2H, d, *J* = 2.2 Hz, H-10′, H-14′), 6.44 (2H, d, *J* = 2.1 Hz, H-10, H-14), 6.79 (2H, d, *J* = 8.7 Hz, H-3′, H-5′), 6.98 (1H, d, *J* = 16.3 Hz, H-8), 7.16 (1H, d, *J* = 16.3 Hz, H-7), 7.47 (2H, d, *J* = 8.7 Hz, H-2′, H-6′), 7.53 (1H, s, H-2), 7.59 (2H, m, H-5, H-6), 9.22 (2H, s, 11OH, 13OH), 9.41 (2H, s, 11′OH, 13′OH), 9.86 (1H, s, 4′OH); ^13^C NMR (DMSO, 151 MHz) δ 102.1 (C-12, C-12′), 104.6 (C-10, C-14), 107.3 (C-10′, C-14′), 111.2 (C-5), 115.3 (C-8′), 115.6 (C-3′, C-5′), 117.5 (C-2), 120.7 (C-1′), 123.0 (C-6), 127.9 (C-8), 128.2 (C-7), 128.3 (C-2′, C-6′), 130.2 (C-3), 132.5 (C-1), 133.7 (C-9′), 139.0 (C-9), 150.9 (C-7′), 152.7 (C-4), 158.1 (C-4′), 158.5 (C-11, C-13), 159.0 (C-11′, C-13′); HR-ESI/MS analysis: *m/z* 451.1175 [M-H]^−^, (calcd for C_28_H_19_O_6_^−^, 451.1187 , ∆ = 2.7 ppm). MS/MS spectrum: CCMSLIB00010129247. SMILES: OC1=CC=C(C(O2)=C(C3=CC(O)=CC(O)=C3)C4=C2C=CC(/C=C/C5=CC(O)=CC(O)=C5)=C4)C=C1.

11′,13′-di-*O*-methyl-dehydro-*trans*-δ-viniferin (**17**): UV (MeOH) λ_max_ (log ε) 306 (4.51) nm; ^1^H NMR (DMSO-*d*_6_, 600 MHz) δ 3.76 (6H, s, CH_3_O-11′, CH_3_O-13′), 6.15 (1H, t, *J* = 2.1 Hz, H-12), 6.44 (2H, d, *J* = 2.1 Hz, H-10, H-14), 6.59 (1H, t, *J* = 2.3 Hz, H-12′), 6.61 (2H, d, *J* = 2.3 Hz, H-10′, H-14′), 6.79 (2H, d, *J* = 8.7 Hz, H-3′, H-5′), 7.00 (1H, d, *J* = 16.3 Hz, H-8), 7.18 (1H, d, *J* = 16.3 Hz, H-7), 7.45 (2H, d, *J* = 8.7 Hz, H-2′, H-6′), 7.57 (1H, d, *J* = 1.6 Hz, H-2), 7.61 (1H, d, *J* = 8.6 Hz, H-5), 7.63 (1H, dd, *J* = 8.6, 1.6 Hz, H-6), 9.22 (2H, s, 11OH, 13OH), 9.89 (1H, s, 4′OH); ^13^C NMR (DMSO-*d*_6_, 151 MHz) δ 55.3 (CH_3_O-11′, CH_3_O-13′), 99.8 (C-12′), 102.1 (C-12), 104.7 (C-10, C-14), 107.4 (C-10′, C-14′), 111.2 (C-5), 115.0 (C-8′), 115.6 (C-3′, C-5′), 117.7 (C-2), 120.5 (C-1′), 122.9 (C-6), 127.9 (C-8), 128.3 (C-7), 128.4 (C-2′, C-6′), 130.0 (C-3), 132.7 (C-1), 134.1 (C-9′), 139.0 (C-9), 151.3 (C-7′), 152.7 (C-4), 158.2 (C-4′), 158.5 (C-11, C-13), 161.0 (C-11′, C-13′); HR-ESI/MS analysis: *m/z* 479.1490 [M-H]^−^, (calcd for C_30_H_23_O_6_^−^, 479.1500 , ∆ = 2.1 ppm). MS/MS spectrum: CCMSLIB00010129248. SMILES: OC1=CC=C(C(O2)=C(C3=CC(OC)=CC(OC)=C3)C4=C2C=CC(/C=C/C5=CC(O)=CC(O)=C5)=C4)C=C1.

11,13-di-*O*-methyl-dehydro-*trans*-δ-viniferin (**18**): UV (MeOH) λ_max_ (log ε) 306 (4.49) nm; ^1^H NMR (DMSO-*d*_6_, 600 MHz) δ 3.77 (6H, s, CH_3_O-11, CH_3_O-13), 6.28 (1H, t, *J* = 2.2 Hz, H-12′), 6.32 (2H, d, *J* = 2.2 Hz, H-10′, H-14′), 6.38 (1H, t, *J* = 2.2 Hz, H-12), 6.80 (2H, d, *J* = 8.6 Hz, H-3′, H-5′), 6.80 (2H, d, *J* = 2.2 Hz, H-10, H-14), 7.11 (1H, d, *J* = 16.4 Hz, H-8), 7.41 (1H, d, *J* = 16.4 Hz, H-7), 7.47 (2H, d, *J* = 8.6 Hz, H-2′, H-6′), 7.56 (1H, d, *J* = 1.6 Hz, H-2), 7.61 (1H, d, *J* = 8.5 Hz, H-5), 7.63 (1H, dd, *J* = 8.5, 1.6 Hz, H-6), 9.42 (2H, s, 11′OH, 13′OH), 9.87 (1H, s, 4′OH); ^13^C NMR (DMSO-*d*_6_, 151 MHz) δ 55.2 (CH_3_O-11, CH_3_O-13), 99.9 (C-12), 102.1 (C-12′), 104.3 (C-10, C-14), 107.4 (C-10′, C-14′), 111.3 (C-5), 115.3 (C-8′), 115.6 (C-3′, C-5′), 117.8 (C-2), 120.7 (C-1′), 122.8 (C-6), 127.3 (C-8), 128.3 (C-2′, C-6′), 129.3 (C-7), 130.4 (C-3), 132.4 (C-1), 133.7 (C-9′), 139.4 (C-9), 150.9 (C-7′), 152.8 (C-4), 158.1 (C-4′), 159.0 (C-11′, C-13′), 160.6 (C-11, C-13); HR-ESI/MS analysis: *m/z* 479.1489 [M-H]^−^, (calcd for C_30_H_23_O_6_^−^, 479.1500, ∆ = 2.3 ppm). MS/MS spectrum: CCMSLIB00010129249. SMILES: OC1=CC=C(C(O2)=C(C3=CC(O)=CC(O)=C3)C4=C2C=CC(/C=C/C5=CC(OC)=CC(OC)=C5)=C4)C=C1.

11,11′,13,13′-tetra-*O*-methyl-dehydro-*trans*-δ-viniferin (**19**): UV (MeOH) λ_max_ (log ε) 307 (4.51) nm; ^1^H NMR (DMSO-*d*_6_, 600 MHz) δ 3.76 (6H, s, CH_3_O-11′, CH_3_O-13′), 3.77 (6H, s, CH_3_O-11, CH_3_O-13), 6.38 (1H, t, *J* = 2.3 Hz, H-12), 6.60 (3H, m, H-10′, H-12′, H-14′), 6.79 (2H, d, *J* = 8.7 Hz, H-3′, H-5′), 6.80 (2H, d, *J* = 2.3 Hz, H-10, H-14), 7.13 (1H, d, *J* = 16.4 Hz, H-8), 7.42 (1H, d, *J* = 16.4 Hz, H-7), 7.45 (2H, d, *J* = 8.7 Hz, H-2′, H-6′), 7.57 (1H, d, *J* = 1.6 Hz, H-2), 7.63 (1H, d, *J* = 8.6 Hz, H-5), 7.66 (1H, dd, *J* = 8.6, 1.6 Hz, H-6), 9.88 (1H, s, 4′OH); ^13^C NMR (DMSO-*d*_6_, 151 MHz) δ 55.2 (CH_3_O-11, CH_3_O-13), 55.3 (CH_3_O-11′, CH_3_O-13′), 99.7 (C-12′), 99.8 (C-12), 104.3 (C-10, C-14), 107.5 (C-10′, C-14′), 111.3 (C-5), 115.0 (C-8′), 115.6 (C-3′, C-5′), 117.9 (C-2), 120.5 (C-1′), 122.7 (C-6), 127.3 (C-8), 128.3 (C-2′, C-6′), 129.3 (C-7), 130.2 (C-3), 132.6 (C-1), 134.1 (C-9′), 139.4 (C-9), 151.3 (C-7′), 152.8 (C-4), 158.2 (C-4′), 160.6 (C-11, C-13), 161.0 (C-11′, C-13′); HR-ESI/MS analysis: *m/z* 509.1921 [M + H]^+^, (calcd for C_32_H_29_O_6_^+^, 509.1959 , ∆ = 7.2 ppm). MS/MS spectrum: CCMSLIB00010129250. SMILES: OC1=CC=C(C(O2)=C(C3=CC(OC)=CC(OC)=C3)C4=C2C=CC(/C=C/C5=CC(OC)=CC(OC)=C5)=C4)C=C1.

14-bromo-*trans*-*δ*-viniferin (**20**): UV (MeOH) λ_max_ (log ε) 229 (sh) (4.52), 288 (sh) (4.09), 313 (4.26), 332 (4.24) nm; ^1^H NMR (DMSO-*d*_6_, 600 MHz) δ 4.47 (1H, d, *J* = 7.8 Hz, H-8′), 5.41 (1H, d, *J* = 7.8 Hz, H-7′), 6.04 (2H, d, *J* = 2.0 Hz, H-10′, H-14′), 6.10 (1H, t, *J* = 2.0 Hz, H-12′), 6.38 (1H, d, *J* = 2.6 Hz, H-12), 6.61 (1H, d, *J* = 2.6 Hz, H-10), 6.76 (2H, d, *J* = 8.5 Hz, H-3′, H-5′), 6.92 (1H, d, *J* = 8.3 Hz, H-5), 7.01 (1H, d, *J* = 16.2 Hz, H-7), 7.14 (1H, d, *J* = 16.2 Hz, H-8), 7.18 (2H, d, *J* = 8.5 Hz, H-2′, H-6′), 7.20 (1H, s, H-2), 7.43 (1H, d, *J* = 8.3 Hz, H-6), 9.22 (2H, s, 11′OH), 9.45 (1H, s, 11OH), 9.53 (1H, s, 4′OH), 10.00 (1H, s, 13OH); ^13^C NMR (DMSO-*d*_6_, 151 MHz) δ 55.5 (C-8′), 100.9 (C-14), 101.3 (C-12′), 102.5 (C-12), 104.2 (C-10), 105.9 (C-10′, C-14′), 109.5 (C-5), 115.3 (C-3′, C-5′), 123.1 (C-2), 124.7 (C-8), 127.8 (C-2′, C-6′), 128.0 (C-6), 130.0 (C-1), 130.3 (C-1′), 130.8 (C-7), 131.4 (C-3), 137.9 (C-9), 143.7 (C-9′), 154.9 (C-13), 157.1 (C-11), 157.5 (C-4′), 158.6 (C-11′, C-13′), 159.3 (C-4); HR-ESI/MS analysis: *m/z* 531.0446 [M-H]^−^, (calcd for C_28_H_20_BrO_6_^−^, 531.0443, ∆ = 0.4 ppm). MS/MS spectrum: CCMSLIB00010129251. SMILES: OC1=CC=C([C@H](O2)[C@H](C3=CC(O)=CC(O)=C3)C4=C2C=CC(/C=C/C5=C(Br)C(O)=CC(O)=C5)=C4)C=C1.

14,14′-dibromo-*trans*-*δ*-viniferin (**21**): UV (MeOH) λ_max_ (log ε) 230 (sh) (4.59), 288 (sh) (4.10), 313 (4.25) nm; ^1^H NMR (DMSO-*d*_6_, 600 MHz) δ 5.05 (1H, brs, H-8′), 5.46 (1H, brs, H-7′), 5.96 (1H, d, *J* = 2.7 Hz, H-10′), 6.36 (1H, d, *J* = 2.7 Hz, H-12′), 6.38 (1H, d, *J* = 2.7 Hz, H-12), 6.61 (1H, d, *J* = 2.7 Hz, H-10), 6.73 (2H, d, *J* = 8.4 Hz, H-3′, H-5′), 6.95 (1H, d, *J* = 8.3 Hz, H-5), 7.00 (1H, d, *J* = 16.0 Hz, H-7), 7.15 (1H, d, *J* = 16.0 Hz, H-8), 7.18 (2H, d, *J* = 8.4 Hz, H-2′, H-6′), 7.27 (1H, s, H-2), 7.46 (2H, d, *J* = 8.3 Hz, H-6), 9.45 (2H, s, 11OH), 9.46 (1H, s, 11′OH), 9.51 (1H, s, 4′OH), 10.00 (1H, s, 13OH), 10.12 (1H, s, 13′OH); ^13^C NMR (DMSO-*d*_6_, 151 MHz) δ 54.2 (C-8′), 91.9 (C-7′), 100.8 (C-14′), 100.9 (C-14), 102.3 (C-12′), 102.5 (C-12), 104.2 (C-10), 106.7 (C-10′), 109.7 (C-5), 115.2 (C-3′, C-5′), 123.1 (C-2), 124.9 (C-8), 127.6 (C-2′, C-6′), 128.1 (C-6), 130.7 (C-7), 130.8 (C-1′), 130.9 (C-1), 138.0 (C-9), 154.9 (C-13), 157.0 (C-11), 157.5 (C-11′), 157.5 (C-4′), 159.4 (C-4); HR-ESI/MS analysis: *m/z* 608.9565 [M-H]^−^, (calcd for C_28_H_19_Br_2_O_6_^−^, 608.9548, ∆ = 2.8 ppm). MS/MS spectrum: CCMSLIB00010129252. SMILES: OC1=CC=C([C@H](O2)[C@H](C3=C(Br)C(O)=CC(O)=C3)C4=C2C=CC(/C=C/C5=C(Br)C(O)=CC(O)=C5)=C4)C=C1.

14-chloro-*trans*-*δ*-viniferin (**22**): UV (MeOH) λ_max_ (log ε) 229 (sh) (4.52), 289 (sh) (4.10), 314 (4.29), 333 (4.28) nm; ^1^H NMR (DMSO-*d*_6_, 600 MHz) δ 4.46 (1H, d, *J* = 7.8 Hz, H-8′), 5.41 (1H, d, *J* = 7.8 Hz, H-7′), 6.04 (2H, d, *J* = 2.2 Hz, H-10′, H-14′), 6.10 (1H, d, *J* = 2.2 Hz, H-12′), 6.38 (1H, d, *J* = 2.7 Hz, H-12), 6.61 (1H, d, *J* = 2.7 Hz, H-10), 6.76 (2H, d, *J* = 8.4 Hz, H-3′, H-5′), 6.91 (1H, d, *J* = 8.2 Hz, H-5), 7.05 (1H, d, *J* = 16.4 Hz, H-7), 7.16 (1H, d, *J* = 16.4 Hz, H-8), 7.18 (2H, d, *J* = 8.4 Hz, H-2′, H-6′), 7.22 (1H, s, H-2), 7.44 (1H, dd, *J* = 8.2, 1.8 Hz, H-6), 9.22 (2H, s, 11′OH, 13′OH), 9.41 (1H, s, 11OH), 9.54 (1H, s, 4′OH), 9.91 (1H, s, 13OH); ^13^C NMR (DMSO-*d*_6_, 151 MHz) δ 55.5 (C-8′), 92.6 (C-7′), 101.3 (C-12′), 102.7 (C-12), 103.6 (C-10), 105.9 (C-10′, C-14′), 109.5 (C-5), 109.6 (C-14), 115.3 (C-3′, C-5′), 121.9 (C-8), 123.1 (C-2), 127.8 (C-2′, C-6′), 128.0 (C-6), 130.0 (C-1), 130.3 (C-1′), 130.7 (C-7), 131.4 (C-3), 136.2 (C-9), 143.7 (C-9′), 153.9 (C-13), 156.3 (C-11), 157.5 (C-4′), 158.6 (C-11′, C-13′), 159.3 (C-4); HR-ESI/MS analysis: *m/z* 487.0949 [M-H]^−^, (calcd for C_28_H_20_ClO_6_^−^, 487.0948, ∆ = 0.1 ppm). MS/MS spectrum: CCMSLIB00010129259. SMILES: OC1=CC=C([C@H](O2)[C@H](C3=CC(O)=CC(O)=C3)C4=C2C=CC(/C=C/C5=C(Cl)C(O)=CC(O)=C5)=C4)C=C1.

14,14′-dichloro-*trans*-*δ*-viniferin (**23**): UV (MeOH) λ_max_ (log ε) 229 (sh) (4.51), 287 (sh) (4.09), 313 (4.22), 332 (4.19) nm; ^1^H NMR (DMSO-*d*_6_, 600 MHz) δ 5.00 (1H, d, *J* = 5.9 Hz, H-8′), 5.47 (1H, d, *J* = 5.9 Hz, H-7′), 5.94 (1H, d, *J* = 2.7 Hz, H-10′), 6.36 (1H, d, *J* = 2.7 Hz, H-12′), 6.38 (1H, d, *J* = 2.7 Hz, H-12), 6.61 (1H, d, *J* = 2.7 Hz, H-10), 6.74 (2H, d, *J* = 8.6 Hz, H-3′, H-5′), 6.94 (1H, d, *J* = 8.3 Hz, H-5), 7.05 (1H, d, *J* = 16.2 Hz, H-7), 7.18 (3H, m, H-2′, H-6′, H-8), 7.28 (1H, s, H-2), 7.47 (1H, dd, *J* = 8.3, 1.9 Hz, H-6), 9.41 (1H, s, 11OH), 9.42 (1H, s, 11′OH), 9.51 (1H, s, 4′OH), 9.91 (1H, s, 13OH), 10.03 (1H, s, 13′OH); ^13^C NMR (DMSO-*d*_6_, 151 MHz) δ 91.6 (C-7′), 102.4 (C-12′), 102.7 (C-12), 103.6 (C-10), 109.6 (C-5), 109.7 (C-14), 109.9 (C-14′), 115.2 (C-3′, C-5′), 122.1 (C-8), 123.3 (C-2), 127.6 (C-2′, C-6′), 128.2 (C-6), 130.2 (C-1), 130.5 (C-3), 130.7 (C-7), 130.8 (C-9′), 136.3 (C-9), 153.9 (C-13), 154.0 (C-13′), 156.3 (C-11), 156.7 (C-11′), 157.5 (C-4′), 159.5 (C-4); HR-ESI/MS analysis: *m/z* 521.0564 [M-H]^−^, (calcd for C_28_H_19_Cl_2_O_6_^−^, 521.0559, ∆ = 1.0 ppm). MS/MS spectrum: CCMSLIB00010129258. SMILES: OC1=CC=C([C@H](O2)[C@H](C3=C(Cl)C(O)=CC(O)=C3)C4=C2C=CC(/C=C/C5=C(Cl)C(O)=CC(O)=C5)=C4)C=C1.

14-iodo-*trans*-*δ*-viniferin (**24**): UV (MeOH) λ_max_ (log ε) 229 (sh) (4.96), 288 (sh) (4.53), 313 (4.68), 332 (4.65) nm; ^1^H NMR (DMSO-*d*_6_, 600 MHz) δ 4.48 (1H, d, *J* = 7.8 Hz, H-8′), 5.41 (1H, d, *J* = 7.8 Hz, H-7′), 6.04 (2H, d, *J* = 2.2 Hz, H-10′, H-14′), 6.10 (1H, t, *J* = 2.2 Hz, H-12′), 6.35 (1H, d, *J* = 2.6 Hz, H-12), 6.60 (1H, d, *J* = 2.6 Hz, H-10), 6.76 (2H, d, *J* = 8.5 Hz, H-3′, H-5′), 6.92 (1H, d, *J* = 16.0 Hz, H-7), 6.93 (1H, d, *J* = 8.1 Hz, H-5), 7.07 (1H, d, *J* = 16.0 Hz, H-8), 7.18 (3H, m, H-2, H-2′, H-6′), 7.43 (1H, dd, *J* = 8.1, 1.9 Hz, H-6), 9.21 (1H, s), 9.21 (2H, s, 11′OH, 13′OH), 9.47 (1H, s, 11OH), 9.53 (1H, s, 4′OH), 10.14 (1H, s, 13OH); ^13^C NMR (DMSO-*d*_6_, 151 MHz) δ 55.5 (C-8′), 78.4 (C-14), 92.5 (C-7′), 101.2 (C-12′), 101.3 (C-12), 104.4 (C-10), 105.8 (C-10′, C-14′), 109.4 (C-5), 115.1 (C-3′, C-5′), 123.0 (C-2), 127.8 (C-6, C-2′, C-6′), 129.8 (C-1, C-8), 130.3 (C-1′), 130.6 (C-7), 131.2 (C-3), 141.1 (C-9), 143.5 (C-9′), 157.5 (C-4′), 158.2 (C-11), 158.6 (C-11′, C-13′), 159.3 (C-4); HR-ESI/MS analysis: *m/z* 579.0314 [M-H]^−^, (calcd for C_28_H_20_IO_6_^−^, 579.0305, ∆ = 1.6 ppm). MS/MS spectrum: CCMSLIB00010129268. SMILES: OC1=CC=C([C@H](O2)[C@H](C3=CC(O)=CC(O)=C3)C4=C2C=CC(/C=C/C5=C(I)C(O)=CC(O)=C5)=C4)C=C1.

12-iodo-*trans*-*δ*-viniferin (**25**): UV (MeOH) λ_max_ (log ε) 229 (sh) (4.56), 288 (sh) (4.08), 313 (4.27), 336 (4.29), 353 (sh) (4.05) nm; ^1^H NMR (DMSO-*d*_6_, 600 MHz) δ 4.44 (1H, d, *J* = 7.7 Hz, H-8′), 5.39 (1H, d, *J* = 7.7 Hz, H-7′), 6.03 (2H, d, *J* = 2.2 Hz, H-10′, H-14′), 6.10 (1H, t, *J* = 2.2 Hz, H-12′), 6.51 (2H, s, H-10, H-14), 6.76 (3H, d, *J* = 8.6 Hz, H-3′, H-5′), 6.86 (1H, d, *J* = 16.3 Hz, H-8), 6.90 (1H, d, *J* = 8.3 Hz, H-5), 6.96 (1H, d, *J* = 16.3 Hz, H-7), 7.18 (2H, d, *J* = 8.6 Hz, H-2′, H-6′), 7.27 (1H, s, H-2), 7.44 (1H, dd, *J* = 8.3, 1.9 Hz, H-6), 9.21 (2H, s, 11′OH, 13′OH), 9.53 (2H, s, 4′OH), 10.06 (2H, s, 11OH, 13OH); ^13^C NMR (DMSO-*d*_6_, 151 MHz) δ 55.7 (C-8′), 74.1 (C-12), 92.6 (C-7′), 101.3 (C-12′), 103.7 (C-10, C-14), 105.9 (C-10′, C-14′), 109.4 (C-5), 115.3 (C-3′, C-5′), 123.1 (C-2), 125.6 (C-8), 127.8 (C-2′, C-6′), 128.0 (C-6), 128.4 (C-7), 130.1 (C-1), 130.4 (C-1′), 131.1 (C-3), 138.6 (C-9), 143.7 (C-9′), 157.5 (C-4′), 158.0 (C-11, C-13), 158.7 (C-11′, C-13′), 159.1 (C-4); HR-ESI/MS analysis: *m/z* 579.0315 [M–H]^−^, (calcd for C_28_H_20_IO_6_^−^, 579.0305, ∆ = 1.8 ppm). MS/MS spectrum: CCMSLIB00010129269. SMILES: OC1=CC=C([C@H](O2)[C@H](C3=CC(O)=CC(O)=C3)C4=C2C=CC(/C=C/C5=CC(O)=C(I)C(O)=C5)=C4)C=C1.

14-bromo-11′,13′-di-*O*-methyl-*trans*-*δ*-viniferin (**26**): UV (MeOH) λ_max_ (log ε) 229 (sh) (4.49), 285 (sh) (4.07), 313 (4.28), 332 (4.26) nm; ^1^H NMR (DMSO-*d*_6_, 600 MHz) δ 3.70 (6H, s, CH_3_O-11′, CH_3_O-13′), 4.62 (1H, d, *J* = 8.0 Hz, H-8′), 5.58 (1H, d, *J* = 8.0 Hz, H-7′), 6.37 (3H, m, H-10′, H-12, 14′), 6.43 (1H, t, *J* = 2.3 Hz, H-12′), 6.61 (1H, d, *J* = 2.6 Hz, H-10), 6.76 (2H, d, *J* = 8.5 Hz, H-3′, H-5′), 6.93 (1H, d, *J* = 8.3 Hz, H-5), 7.00 (1H, d, *J* = 16.1 Hz, H-7), 7.13 (1H, d, *J* = 16.1 Hz, H-8), 7.20 (3H, m, H-2, H-2′, H-6′), 7.44 (1H, dd, *J* = 8.3, 1.9 Hz, H-6), 9.45 (1H, s, 11OH), 9.53 (1H, s, 4′OH), 10.00 (1H, s, 13OH); ^13^C NMR (DMSO-*d*_6_, 151 MHz) δ 55.2 (CH_3_O-11′, CH_3_O-13′), 55.5 (C-8′), 92.0 (C-7′), 98.6 (C-12′), 100.8 (C-14), 102.5 (C-12), 104.2 (C-10), 106.1 (C-10′, C-14′), 109.6 (C-5), 115.3 (C-3′, C-5′), 123.0 (C-2), 124.7 (C-8), 127.9 (C-2′, C-6′), 128.0 (C-6), 130.1 (C-1, C-1′), 130.8 (C-7), 131.4 (C-3), 137.9 (C-9), 143.7 (C-9′), 154.9 (C-13), 157.1 (C-11), 157.6 (C-4′), 159.1 (C-4), 160.7 (C-11′, C-13′); HR-ESI/MS analysis: *m/z* 559.0764 [M-H]^−^, (calcd for C_30_H_24_BrO_6_^−^, 559.0756, ∆ = 1.4 ppm). MS/MS spectrum: CCMSLIB00010129254. SMILES: OC1=CC=C([C@H](O2)[C@H](C3=CC(OC)=CC(OC)=C3)C4=C2C=CC(/C=C/C5=C(Br)C(O)=CC(O)=C5)=C4)C=C1.

10,14-dibromo-11′,13′-di-*O*-methyl-*trans*-*δ*-viniferin (**27**): UV (MeOH) λ_max_ (log ε) 229 (sh) (4.62), 288 (4.23), 309 (4.20) nm; ^1^H NMR (DMSO-*d*_6_, 600 MHz) δ 3.70 (6H, s, CH_3_O-11′, CH_3_O-13′), 4.60 (1H, d, *J* = 7.8 Hz, H-8′), 5.60 (1H, d, *J* = 7.8 Hz, H-7′), 6.38 (2H, d, *J* = 2.3 Hz, H-10′, H-14′), 6.42 (1H, t, *J* = 2.3 Hz, H-12′), 6.63 (1H, s, H-12), 6.68 (1H, d, *J* = 16.5 Hz, H-7), 6.76 (2H, d, *J* = 8.6 Hz, H-3′, H-5′), 6.76 (1H, d, *J* = 16.5 Hz, H-8), 6.93 (1H, d, *J* = 8.2 Hz, H-5), 7.18 (1H, s, H-2), 7.20 (2H, d, *J* = 8.6 Hz, H-2′, H-6′), 7.44 (1H, dd, *J* = 8.2, 1.9 Hz, H-6), 9.53 (1H, s, 4′OH), 10.26 (2H, s, 11OH, 13OH); ^13^C NMR (DMSO-*d*_6_, 151 MHz) δ 55.0 (CH_3_O-11′, CH_3_O-13′), 55.5 (C-8′), 91.9 (C-7′), 98.4 (C-12′), 100.6 (C-10, C-14), 102.0 (C-12), 106.0 (C-10′, C-14′), 109.5 (C-5), 115.1 (C-3′, C-5′), 122.9 (C-2), 125.2 (C-8), 127.6 (C-6), 127.8 (C-2′, C-6′), 129.6 (C-1), 130.1 (C-1′), 131.2 (C-3), 135.5 (C-7), 138.8 (C-9), 143.8 (C-9′), 153.9 (C-11, C-13), 157.5 (C-4′), 159.1 (C-4), 160.6 (C-11′, C-13′); HR-ESI/MS analysis: *m/z* 636.9877 [M-H]^−^, (calcd for C_30_H_23_Br_2_O_6_^−^, 636.9861, ∆ = 2.4 ppm). MS/MS spectrum: CCMSLIB00010129253. SMILES: OC1=CC=C([C@H](O2)[C@H](C3=CC(OC)=CC(OC)=C3)C4=C2C=CC(/C=C/C5=C(Br)C(O)=CC(O)=C5Br)=C4)C=C1.

14-chloro-11′,13′-di-*O*-methyl-*trans*-*δ*-viniferin (**28**): UV (MeOH) λ_max_ (log ε) 228 (sh) (4.58), 286 (sh) (4.16), 314 (4.39), 330 (4.37) nm; ^1^H NMR (DMSO-*d*_6_, 600 MHz) δ 3.70 (6H, s, CH_3_O-11′, CH_3_O-13′), 4.61 (1H, d, *J* = 8.1 Hz, H-8′), 5.59 (1H, d, *J* = 8.1 Hz, H-7′), 6.37 (3H, m, H-10′, H-12, H-14′), 6.43 (1H, t, *J* = 2.3 Hz, H-12′), 6.61 (1H, d, *J* = 2.6 Hz, H-10), 6.76 (2H, d, *J* = 8.6 Hz, H-3′, H-5′), 6.93 (1H, d, *J* = 8.3 Hz, H-5), 7.04 (1H, d, *J* = 16.2 Hz, H-7), 7.15 (1H, d, *J* = 16.2 Hz, H-8), 7.20 (3H, m, H-2, H-2′, H-6′), 7.45 (1H, dd, *J* = 8.3, 1.9 Hz, H-6), 9.41 (1H, s, 11OH), 9.53 (1H, s, 4′OH), 9.91 (1H, s, 13OH); ^13^C NMR (DMSO-*d*_6_, 151 MHz) δ 55.1 (CH_3_O-11′, CH_3_O-13′), 55.5 (C-8′), 92.0 (C-7′), 98.6 (C-12′), 102.7 (C-12), 103.6 (C-10), 106.1 (C-10′, C-14′), 109.6 (C-5), 109.6 (C-14), 115.3 (C-3′, C-5′), 121.9 (C-8), 123.0 (C-2), 127.8 (C-2′, C-6′), 128.0 (C-6), 130.1 (C-1′, C-3), 130.7 (C-7), 131.4 (C-1), 136.3 (C-9), 143.8 (C-9′), 153.9 (C-13), 156.3 (C-11), 157.6 (C-4′), 159.1 (C-4), 160.7 (C-11′, C-13′); HR-ESI/MS analysis: *m/z* 515.1262 [M-H]^−^, (calcd for C_30_H_24_ClO_6_^−^, 515.1261, ∆ = 0.1 ppm). MS/MS spectrum: CCMSLIB00010129261. SMILES: OC1=CC=C([C@H](O2)[C@H](C3=CC(OC)=CC(OC)=C3)C4=C2C=CC(/C=C/C5=C(Cl)C(O)=CC(O)=C5)=C4)C=C1.

12-chloro-11′,13′-di-*O*-methyl-*trans*-*δ*-viniferin (**29**): UV (MeOH) λ_max_ (log ε) 228 (sh) (4.46), 286 (sh) (4.03), 313 (4.29), 333 (4.27), 350 (sh) (4.04) nm; ^1^H NMR (DMSO-*d*_6_, 600 MHz) δ 3.70 (6H, s, CH_3_O-11′, CH_3_O-13′), 4.58 (1H, d, *J* = 8.0 Hz, H-8′), 5.56 (1H, d, *J* = 8.0 Hz, H-7′), 6.36 (2H, d, *J* = 2.3 Hz, H-10′, H-14′), 6.43 (1H, t, *J* = 2.3 Hz, H-12′), 6.58 (2H, s, H-10, H-14), 6.76 (2H, d, *J* = 8.6 Hz, H-3′, H-5′), 6.84 (1H, d, *J* = 16.4 Hz, H-8), 6.91 (1H, d, *J* = 8.3 Hz, H-5), 6.93 (1H, d, *J* = 15.4 Hz, H-7), 7.19 (2H, d, *J* = 8.6 Hz, H-2′, H-6′), 7.24 (1H, d, *J* = 1.3 Hz, H-2), 7.44 (1H, dd, *J* = 8.3, 1.3 Hz, H-6), 9.53 (1H, s, 4′OH), 9.87 (2H, s, 11OH, 13OH); ^13^C NMR (DMSO-*d*_6_, 151 MHz) δ 55.0 (CH_3_O-11′, CH_3_O-13′), 55.5 (C-8′), 91.9 (C-7′), 98.4 (C-12′), 104.8 (C-10, C-14), 106.2 (C-12, C-10′, C-14′), 109.4 (C-5), 115.1 (C-3′, C-5′), 122.7 (C-2), 125.5 (C-8), 127.8 (C-2′, C-6′), 128.0 (C-6), 128.1 (C-7), 130.1 (C-1′), 131.0 (C-3), 136.4 (C-9), 143.8 (C-9′), 154.1 (C-11, C-13), 157.5 (C-4′), 158.8 (C-4), 160.6 (C-11′, C-13′); HR-ESI/MS analysis: *m/z* 515.1263 [M-H]^−^, (calcd for C_30_H_24_ClO_6_^−^, 515.1261, ∆ = 0.3 ppm). MS/MS spectrum: CCMSLIB00010129260. SMILES: OC1=CC=C([C@H](O2)[C@H](C3=CC(OC)=CC(OC)=C3)C4=C2C=CC(/C=C/C5=CC(O)=C(Cl)C(O)=C5)=C4)C=C1.

10,14-dichloro-11′,13′-di-*O*-methyl-*trans*-*δ*-viniferin (**30**): UV (MeOH) λ_max_ (log ε) 229 (sh) (4.49), 285 (sh) (4.13), 300 (4.19) nm; ^1^H NMR (DMSO-*d*_6_, 600 MHz) δ 3.70 (6H, s, CH_3_O-11′, CH_3_O-13′), 4.60 (1H, d, *J* = 7.8 Hz, H-8′), 5.60 (1H, d, *J* = 7.8 Hz, H-7′), 6.38 (2H, d, *J* = 2.3 Hz, H-10′, H-14′), 6.42 (1H, t, *J* = 2.3 Hz, H-12′), 6.61 (1H, s, H-12), 6.76 (2H, d, *J* = 8.5 Hz, H-3′, H-5′), 6.83 (1H, d, *J* = 16.6 Hz, H-8), 6.90 (1H, d, *J* = 16.6 Hz, H-7), 6.94 (1H, d, *J* = 8.3 Hz, H-5), 7.19 (3H, m, H-2, H-2′, H-6′), 7.45 (1H, dd, *J* = 8.3, 1.9 Hz, H-6), 9.53 (1H, s, 4′OH), 10.15 (2H, s, 11OH, 13OH); ^13^C NMR (DMSO-*d*_6_, 151 MHz) δ 55.1 (CH_3_O-11′, CH_3_O-13′), 55.6 (C-8′), 91.9 (C-7′), 98.6 (C-12′), 102.5 (C-12), 106.1 (C-10′, C-14′), 109.6 (C-5), 110.2 (C-10, C-14), 115.3 (C-3′, C-5′), 120.8 (C-8), 123.0 (C-2), 127.8 (C-2′, C-6′), 127.8 (C-6), 129.7 (C-1), 130.2 (C-1′), 131.4 (C-3), 135.4 (C-9), 135.9 (C-7), 143.9 (C-9′), 152.4 (C-11, C-13), 157.6 (C-4′), 159.2 (C-4), 160.7 (C-11′, C-13′); HR-ESI/MS analysis: *m/z* 549.0877 [M-H]^−^, (calcd for C_30_H_23_Cl_2_O_6_^−^, 549.0872, ∆ = 1.0 ppm). MS/MS spectrum: CCMSLIB00010129262. SMILES: OC1=CC=C([C@H](O2)[C@H](C3=CC(OC)=CC(OC)=C3)C4=C2C=CC(/C=C/C5=C(Cl)C(O)=CC(O)=C5Cl)=C4)C=C1.

14-iodo-11′,13′-di-*O*-methyl-*trans*-*δ*-viniferin (**31**): UV (MeOH) λ_max_ (log ε) 229 (sh) (4.63), 287 (sh) (4.15), 313 (4.31), 335 (4.28) nm; ^1^H NMR (DMSO-*d*_6_, 600 MHz) δ 3.70 (6H, s, CH_3_O-11′, CH_3_O-13′), 4.63 (1H, d, *J* = 8.1 Hz, H-8′), 5.58 (1H, d, *J* = 8.1 Hz, H-7′), 6.35 (1H, d, *J* = 2.6 Hz, H-12), 6.37 (2H, d, *J* = 2.3 Hz, H-10′, H-14′), 6.43 (1H, t, *J* = 2.3 Hz, H-12′), 6.59 (1H, d, *J* = 2.6 Hz, H-10), 6.76 (2H, d, *J* = 8.6 Hz, H-3′, H-5′), 6.92 (1H, d, *J* = 16.0 Hz, H-7), 6.94 (1H, d, *J* = 8.3 Hz, H-5), 7.06 (1H, d, *J* = 16.0 Hz, H-8), 7.18 (1H, d, *J* = 1.9 Hz, H-2), 7.20 (2H, d, *J* = 8.6 Hz, H-2′, H-6′), 7.44 (1H, dd, *J* = 8.3, 1.9 Hz, H-6), 9.47 (1H, s, 11OH), 9.53 (1H, s, 4′OH), 10.14 (1H, s, 13OH); ^13^C NMR (DMSO-*d*_6_, 151 MHz) δ 55.0 (CH_3_O-11′, CH_3_O-13′), 55.5 (C-8′), 78.4 (C-14), 91.9 (C-7′), 98.6 (C-12′), 101.3 (C-12), 104.4 (C-10), 106.0 (C-10′, C-14′), 109.5 (C-5), 115.1 (C-3′, C-5′), 122.9 (C-2), 127.8 (C-6, C-2′, C-6′), 129.9 (C-8), 130.1 (C-1), 130.6 (C-7), 131.2 (C-3), 141.1 (C-9), 143.5 (C-9′), 157.3 (C-13), 157.5 (C-4′), 158.2 (C-11), 159.1 (C-4), 160.6 (C-11′, C-13′); HR-ESI/MS analysis: *m/z* 607.0632 [M-H]^−^, (calcd for C_30_H_24_IO_6_^−^, 607.0618, ∆ = 2.4 ppm). MS/MS spectrum: CCMSLIB00010129270. SMILES: OC1=CC=C([C@H](O2)[C@H](C3=CC(OC)=CC(OC)=C3)C4=C2C=CC(/C=C/C5=C(I)C(O)=CC(O)=C5)=C4)C=C1.

12-iodo-11′,13′-di-*O*-methyl-*trans*-*δ*-viniferin (**32**): UV (MeOH) λ_max_ (log ε) 229 (sh) (4.54), 287 (sh) (4.10), 313 (4.40), 337 (4.43), 352 (sh) (4.23) nm; ^1^H NMR (DMSO-*d*_6_, 600 MHz) δ 3.70 (6H, s, CH_3_O-11′, CH_3_O-13′), 4.58 (1H, d, *J* = 8.0 Hz, H-7′), 5.56 (1H, d, *J* = 8.0 Hz, H-8′), 6.36 (2H, d, *J* = 2.2 Hz, H-10′, H-14′), 6.43 (1H, t, *J* = 2.2 Hz, H-12′), 6.50 (2H, s, H-10, H-14), 6.76 (2H, d, *J* = 8.6 Hz, H-3′, H-5′), 6.85 (1H, d, *J* = 16.2 Hz, H-8), 6.91 (1H, d, *J* = 8.3 Hz, H-5), 6.95 (1H, d, *J* = 16.2 Hz, H-7), 7.19 (2H, d, *J* = 8.6 Hz, H-2′, H-6′), 7.25 (1H, s, H-2), 7.45 (1H, dd, *J* = 8.3, 1.9 Hz, H-6), 9.53 (1H, s, 4′OH), 10.06 (2H, s, 11OH, 13OH); ^13^C NMR (DMSO-*d*_6_, 151 MHz) δ 55.1 (CH_3_O-11′, CH_3_O-13′), 55.7 (C-8′), 74.1 (C-12), 92.0 (C-7′), 98.5 (C-12′), 103.7 (C-10, C-14), 106.1 (C-10′, C-14′), 109.5 (C-5), 115.3 (C-3′, C-5′), 122.9 (C-2), 125.6 (C-8), 127.8 (C-2′, C-6′), 128.0 (C-6), 128.3 (C-7), 130.2 (C-1, C-1′), 131.2 (C-3), 138.6 (C-9), 143.8 (C-9′), 157.6 (C-4′), 158.0 (C-11, C-13), 158.9 (C-4), 160.7 (C-11′, C-13′); HR-ESI/MS analysis: *m/z* 607.0626 [M-H]^−^, (calcd for C_30_H_24_IO_6_^−^, 607.0618, ∆ = 1.4 ppm). MS/MS spectrum: CCMSLIB00010129271. SMILES: OC1=CC=C([C@H](O2)[C@H](C3=CC(OC)=CC(OC)=C3)C4=C2C=CC(/C=C/C5=CC(O)=C(I)C(O)=C5)=C4)C=C1.

14,14′-dibromo-11,13-di-*O*-methyl-*trans*-*δ*-viniferin (**33**): UV (MeOH) λ_max_ (log ε) 229 (sh) (4.62), 288 (sh) (4.23), 314 (4.39), 332 (4.36) nm; ^1^H NMR (DMSO-*d*_6_, 600 MHz) δ 3.82 (3H, s, CH_3_O-11), 3.83 (3H, s, CH_3_O-13), 5.08 (1H, brs, H-8′), 5.49 (1H, brs, H-7′), 5.99 (1H, brs, H-10′), 6.37 (1H, d, *J* = 2.7 Hz, H-12′), 6.57 (1H, d, *J* = 2.7 Hz, H-12), 6.74 (2H, d, *J* = 8.5 Hz, H-3′, H-5′), 6.95 (1H, d, *J* = 2.7 Hz, H-10), 6.97 (1H, d, *J* = 8.4 Hz, H-5), 7.19 (2H, d, *J* = 8.5 Hz, H-2′, H-6′), 7.24 (2H, m, H-7, H-8), 7.26 (1H, d, *J* = 1.9 Hz, H-2), 7.52 (1H, dd, *J* = 8.4, 1.9 Hz, H-6), 9.47 (1H, s, 11′OH), 9.52 (1H, s, 4′OH), 10.13 (1H, s, 13′OH); ^13^C NMR (DMSO-*d*_6_, 151 MHz) δ 54.1 (C-8′), 55.5 (CH_3_O-11) 56.2 (CH_3_O-13), 91.9 (C-7′), 98.9 (C-12), 101.1 (C-14′), 102.3 (C-12′), 103.6 (C-14), 106.7 (C-10′) 109.7 (C-5, C-10), 115.1 (C-3′, C-5′), 123.7 (C-2), 123.9 (C-8), 127.6 (C-2′, C-6′), 127.8 (C-6), 130.3 (C-1), 130.5 (C-1′), 130.9 (C-3), 131.9 (C-7), 155.0 (C-13′), 156.4 (C-13), 157.3 (C-11′), 157.5 (C-4′), 159.3 (C-11), 159.5 (C-4); HR-ESI/MS analysis: *m/z* 636.9877 [M-H]^−^, (calcd for C_30_H_23_Br_2_O_6_^−^, 636.9861, ∆ = 2.4 ppm). MS/MS spectrum: CCMSLIB00010129255. SMILES: OC(C=C1)=CC=C1[C@@H](O2)[C@@H](C3=C(Br)C(O)=CC(O)=C3)C4=C2C=CC(/C=C/C5=C(Br)C(OC)=CC(OC)=C5)=C4.

14′-chloro-11,13-di-*O*-methyl-*trans*-*δ*-viniferin (**34**): UV (MeOH) λ_max_ (log ε) 228 (sh) (4.46), 288 (sh) (4.10), 313 (4.29), 332 (4.24) nm; ^1^H NMR (DMSO-*d*_6_, 600 MHz) δ 3.75 (6H, s, CH_3_O-11, CH_3_O-13), 5.01 (1H, brs, H-8′), 5.48 (1H, brs, H-7′), 5.99 (1H, d, *J* = 2.7 Hz, H-10′), 6.35 (1H, t, *J* = 2.2 Hz, H-12), 6.37 (1H, d, *J* = 2.7 Hz, H-12′), 6.72 (2H, d, *J* = 2.2 Hz, H-10, H-14), 6.74 (2H, d, *J* = 8.6 Hz, H-3′, H-5′), 6.93 (1H, d, *J* = 8.3 Hz, H-5), 6.98 (1H, d, *J* = 16.4 Hz, H-8), 7.18 (2H, d, *J* = 8.6 Hz, H-2′, H-6′), 7.22 (1H, d, *J* = 16.4 Hz, H-7), 7.27 (1H, s, H-2), 7.46 (1H, dd, *J* = 8.3, 1.9 Hz, H-6), 9.43 (1H, s, 11′OH), 9.51 (1H, s, 4′OH), 10.02 (1H, s, 13′OH); ^13^C NMR (DMSO-*d*_6_, 151 MHz) δ 55.1 (CH_3_O-11, CH_3_O-13), 91.8 (C-7′), 99.5 (C-12), 102.5 (C-12′), 104.1 (C-10, C-14), 106.8 (C-10′), 109.5 (C-5), 110.1 (C-14′), 115.2 (C-3′, C-5′), 122.9 (C-2), 125.9 (C-8), 127.6 (C-2′, C-6′), 127.9 (C-6), 128.7 (C-7), 130.4 (C-3), 130.6 (C-1), 130.7 (C-1′), 139.5 (C-9), 153.9 (C-13′), 156.7 (C-11′), 157.5 (C-4′), 159.2 (C-4), 160.6 (C-11, C-13); HR-ESI/MS analysis: *m/z* 515.1268 [M-H]^−^, (calcd for C_30_H_24_ClO_6_^−^, 515.1261, ∆ = 1.3 ppm). MS/MS spectrum: CCMSLIB00010129265. SMILES: OC(C=C1)=CC=C1[C@@H](O2)[C@@H](C3=C(Cl)C(O)=CC(O)=C3)C4=C2C=CC(/C=C/C5=CC(OC)=CC(OC)=C5)=C4.

14-chloro-11,13-di-*O*-methyl-*trans*-*δ*-viniferin (**35**): UV (MeOH) λ_max_ (log ε) 229 (sh) (4.71), 287 (sh) (4.31), 313 (4.53), 332 (4.51) nm; ^1^H NMR (DMSO-*d*_6_, 600 MHz) δ 3.82 (3H, s, CH_3_O-11), 3.83 (3H, s, CH_3_O-13), 4.48 (1H, d, *J* = 8.0 Hz, H-8′), 5.43 (1H, d, *J* = 8.0 Hz, H-7′), 6.05 (2H, d, *J* = 2.2 Hz, H-10′, H-14′), 6.10 (1H, t, *J* = 2.2 Hz, H-12′), 6.60 (1H, d, *J* = 2.6 Hz, H-12), 6.76 (2H, d, *J* = 8.6 Hz, H-3′, H-5′), 6.93 (1H, d, *J* = 8.3 Hz, H-5), 6.96 (1H, d, *J* = 2.6 Hz, H-10), 7.19 (2H, d, *J* = 8.6 Hz, H-2′, H-6′), 7.22 (1H, d, *J* = 16.2 Hz, H-8), 7.23 (1H, s, H-2), 7.31 (1H, d, *J* = 16.2 Hz, H-7), 7.50 (1H, dd, *J* = 8.3, 1.9 Hz, H-6), 9.22 (2H, s, 11′OH, 13′OH), 9.54 (1H, s, 4′OH); ^13^C NMR (DMSO-*d*_6_, 151 MHz) δ 55.6 (C-8′, CH_3_O-11), 56.2 (CH_3_O-13), 92.7 (C-7′), 99.1 (C-12), 101.3 (C-12′), 101.8 (C-10), 106.0 (C-10′, C-14′), 109.6 (C-5), 112.3 (C-14), 115.3 (C-3′, C-5′), 121.0 (C-8), 123.7 (C-2), 127.7 (C-6), 127.9 (C-2′, C-6′), 129.9 (C-1), 130.2 (C-1′), 131.5 (C-3), 132.0 (C-7), 136.4 (C-9), 143.6 (C-9′), 155.6 (C-13), 157.6 (C-4′), 158.7 (C-11, C-11′, C-13′), 159.4 (C-4); HR-ESI/MS analysis: *m/z* 515.1263 [M-H]^−^, (calcd for C_30_H_24_ClO_6_^−^, 515.1261, ∆ = 0.3 ppm). MS/MS spectrum: CCMSLIB00010129266. SMILES: OC(C=C1)=CC=C1[C@@H](O2)[C@@H](C3=CC(O)=CC(O)=C3)C4=C2C=CC(/C=C/C5=C(Cl)C(OC)=CC(OC)=C5)=C4.

12-chloro-11,13-di-*O*-methyl-*trans*-*δ*-viniferin (**36**): UV (MeOH) λ_max_ (log ε) 227 (sh) (4.78), 288 (sh) (4.29), 313 (4.53), 335 (4.52), 352 (sh) (4.27) nm; ^1^H NMR (DMSO-*d*_6_, 600 MHz) δ 3.86 (6H, s, CH_3_O-11, CH_3_O-13), 4.48 (1H, d, *J* = 8.5 Hz, H-8′), 5.42 (1H, d, *J* = 8.5 Hz, H-7′), 6.05 (2H, d, *J* = 2.1 Hz, H-10′, H-14′), 6.11 (1H, t, *J* = 2.1 Hz, H-12′), 6.76 (2H, d, *J* = 8.6 Hz, H-3′, H-5′), 6.92 (1H, d, *J* = 8.2 Hz, H-5), 6.98 (2H, s, H-10, H-14), 7.02 (1H, d, *J* = 16.4 Hz, H-8), 7.20 (2H, d, *J* = 8.6 Hz, H-2′, H-6′), 7.24 (1H, s, H-2), 7.33 (1H, d, *J* = 16.4 Hz, H-7), 7.45 (1H, dd, *J* = 8.2, 1.9 Hz, H-6), 9.22 (2H, s, 11′OH, 13′OH), 9.54 (1H, s, 4′OH); ^13^C NMR (DMSO-*d*_6_, 151 MHz) δ 55.5 (C-8′), 56.1 (CH_3_O-11, CH_3_O-13), 92.6 (C-7′), 101.3 (C-12′), 102.8 (C-10, C-14), 106.0 (C-10′, C-14′), 107.6 (C-12), 109.3 (C-5), 115.1 (C-3′, C-5′), 122.8 (C-2), 125.2 (C-8), 127.6 (C-6), 128.0 (C-2′, C-6′), 129.4 (C-7), 129.8 (C-1′), 130.1 (C-1), 131.4 (C-3), 137.3 (C-9), 143.3 (C-9′), 155.5 (C-11, C-13), 157.7 (C-4′), 158.6 (C-11′, C-13′), 159.1 (C-4); HR-ESI/MS analysis: *m/z* 515.1265 [M-H]^−^, (calcd for C_30_H_24_ClO_6_^−^, 515.1261, ∆ = 0.7 ppm). MS/MS spectrum: CCMSLIB00010129263. SMILES: OC(C=C1)=CC=C1[C@@H](O2)[C@@H](C3=CC(O)=CC(O)=C3)C4=C2C=CC(/C=C/C5=CC(OC)=C(Cl)C(OC)=C5)=C4.

14,14′-dichloro-11,13-di-*O*-methyl-*trans*-*δ*-viniferin (**37**): UV (MeOH) λ_max_ (log ε) 229 (sh) (4.59), 313 (4.40), 333 (4.38) nm; ^1^H NMR (DMSO-*d*_6_, 600 MHz) δ 3.82 (3H, s, CH_3_O-11), 3.83 (3H, s, CH_3_O-13), 5.02 (1H, brs, H-8′), 5.50 (1H, brs, H-7′), 5.97 (1H, d, *J* = 2.7 Hz, H-10′), 6.37 (1H, d, *J* = 2.7 Hz, H-12′), 6.60 (1H, d, *J* = 2.7 Hz, H-12), 6.74 (2H, d, *J* = 8.5 Hz, H-3′, H-5′), 6.96 (2H, m, H-5, H-10), 7.19 (2H, d, *J* = 8.5 Hz, H-2′, H-6′), 7.24 (1H, d, *J* = 16.3 Hz, H-8), 7.28 (2H, m, H-2, H-7), 7.53 (1H, dd, *J* = 8.4, 1.9 Hz, H-6), 9.43 (1H, s, 11′OH), 9.52 (1H, s, 4′OH), 10.03 (1H, s, 13′OH); ^13^C NMR (DMSO-*d*_6_, 151 MHz) δ 55.6 (CH_3_O-11), 56.2 (CH_3_O-13), 99.1 (C-12), 101.9 (C-10), 102.5 (C-12′), 109.7 (C-5), 110.0 (C-14′), 112.3 (C-14), 115.2 (C-3′, C-5′), 121.2 (C-8), 123.8 (C-2), 127.6 (C-2′, C-6′), 127.9 (C-6), 130.1 (C-1), 130.7 (C-1′, C-3), 131.9 (C-7), 136.4 (C-9), 154.0 (C-13′), 155.6 (C-13), 156.7 (C-11′), 157.5 (C-4′), 158.7 (C-11), 159.6 (C-4); HR-ESI/MS analysis: *m/z* 549.0881 [M-H]^−^, (calcd for C_30_H_23_Cl_2_O_6_^−^, 549.0872, ∆ = 1.7 ppm). MS/MS spectrum: CCMSLIB00010129264. SMILES: OC(C=C1)=CC=C1[C@@H](O2)[C@@H](C3=C(Cl)C(O)=CC(O)=C3)C4=C2C=CC(/C=C/C5=C(Cl)C(OC)=CC(OC)=C5)=C4.

14′-iodo-11,13-di-*O*-methyl-*trans*-*δ*-viniferin (**38**): UV (MeOH) λ_max_ (log ε) 230 (sh) (4.45), 288 (sh) (4.07), 313 (4.24), 334 (4.18) nm; ^1^H NMR (DMSO-*d*_6_, 600 MHz) δ 3.75 (6H, s, CH_3_O-11, CH_3_O-13), 5.10 (1H, d, *J* = 6.7 Hz, H-8′), 5.44 (1H, d, *J* = 6.7 Hz, H-7′), 6.02 (1H, d, *J* = 2.6 Hz, H-12′ or H-14′), 6.34 (1H, d, *J* = 2.6 Hz, H-14′ or H-12′), 6.35 (1H, t, *J* = 2.3 Hz, H-12), 6.73 (2H, d, *J* = 2.3 Hz, H-10, H-14), 6.74 (2H, d, *J* = 8.6 Hz, H-3′, H-5′), 6.94 (1H, d, *J* = 8.2 Hz, H-5), 6.96 (1H, d, *J* = 16.4 Hz, H-8), 7.18 (2H, d, *J* = 8.6 Hz, H-2′, H-6′), 7.21 (1H, d, *J* = 16.4 Hz, H-7), 7.23 (1H, s, H-2), 7.47 (1H, d, *J* = 8.2 Hz, H-6), 9.47 (1H, s, 11′OH), 9.50 (1H, s, 4′OH), 10.24 (1H, s, 13′OH); ^13^C NMR (DMSO-*d*_6_, 151 MHz) δ 55.0 (CH_3_O-11, CH_3_O-13), 58.9 (C-8′), 79.3 (C-14′), 92.5 (C-7′), 99.5 (C-12), 101.3 (C-10′ or C-12′), 104.1 (C-10, C-14), 106.9 (C-12′ or C-10′), 109.5 (C-5), 115.1 (C-3′, C-5′), 122.7 (C-2), 125.9 (C-8), 127.6 (C-6, C-2′, C-6′), 128.7 (C-7), 130.3 (C-1′), 139.5 (C-9), 157.5 (C-4′, C-13′), 158.6 (C-11′), 158.8 (C-4), 160.4 (C-11, C-13); HR-ESI/MS analysis: *m/z* 607.0627 [M-H]^−^, (calcd for C_30_H_24_IO_6_^−^, 607.0618, ∆ = 1.5 ppm). MS/MS spectrum: CCMSLIB00010129273. SMILES: OC(C=C1)=CC=C1[C@@H](O2)[C@@H](C3=C(I)C(O)=CC(O)=C3)C4=C2C=CC(/C=C/C5=CC(OC)=CC(OC)=C5)=C4.

12′-iodo-11,13-di-*O*-methyl-*trans*-*δ*-viniferin (**39**): UV (MeOH) λ_max_ (log ε) 218 (sh) (4.74), 310 (4.45), 332 (4.41) nm; ^1^H NMR (DMSO-*d*_6_, 600 MHz) δ 3.75 (6H, s, CH_3_O-11, CH_3_O-13), 4.49 (1H, d, *J* = 8.5 Hz, H-8′), 5.35 (1H, d, *J* = 8.5 Hz, H-7′), 6.19 (2H, s, H-10′, H-14′), 6.35 (1H, t, *J* = 2.2 Hz, H-12), 6.73 (2H, d, *J* = 2.2 Hz, H-10, H-14), 6.77 (2H, d, *J* = 8.6 Hz, H-3′, H-5′), 6.92 (1H, d, *J* = 8.3 Hz, H-5), 6.98 (1H, d, *J* = 16.4 Hz, H-8), 7.20 (2H, d, *J* = 8.6 Hz, H-2′, H-6′), 7.23 (1H, d, *J* = 16.4 Hz, H-7), 7.27 (1H, s, H-2), 7.45 (1H, dd, *J* = 8.3, 1.8 Hz, H-6), 9.55 (1H, s, 4′OH), 10.07 (2H, s, 11′OH, 13′OH); ^13^C NMR (DMSO-*d*_6_, 151 MHz) δ 55.1 (CH_3_O-11, CH_3_O-13), 55.6 (C-8′), 73.6 (C-12′), 92.9 (C-7′), 99.5 (C-12), 104.1 (C-10, C-14), 105.6 (C-10′, C-14′), 109.4 (C-5), 115.3 (C-3′, C-5′), 123.0 (C-2), 125.8 (C-8), 127.8 (C-6), 128.0 (C-2′, C-6′), 128.7 (C-7), 129.9 (C-1′), 130.4 (C-1), 131.0 (C-3), 139.5 (C-9), 143.0 (C-9′), 157.6 (C-4′), 158.1 (C-11′, C-13′), 159.1 (C-4), 160.6 (C-11, C-13); HR-ESI/MS analysis: *m/z* 607.0627 [M-H]^−^, (calcd for C_30_H_24_IO_6_^−^, 607.0618, ∆ = 1.5 ppm). MS/MS spectrum: CCMSLIB00010129272. SMILES: OC(C=C1)=CC=C1[C@@H](O2)[C@@H](C3=CC(O)=C(I)C(O)=C3)C4=C2C=CC(/C=C/C5=CC(OC)=CC(OC)=C5)=C4.

12-bromo-11,11′,13,13′-tetra-*O*-methyl-*trans*-*δ*-viniferin (**40**): UV (MeOH) λ_max_ (log ε) 229 (sh) (4.50), 286 (sh) (4.06), 313 (4.35), 336 (4.38), 354 (sh) (4.12) nm; ^1^H NMR (DMSO-*d*_6_, 600 MHz) δ 3.70 (6H, s, CH_3_O-11′, CH_3_O-13′), 3.86 (6H, s, CH_3_O-11, CH_3_O-13), 4.62 (1H, d, *J* = 8.8 Hz, H-8′), 5.60 (1H, d, *J* = 8.8 Hz, H-7′), 6.39 (2H, d, *J* = 2.3 Hz, H-10′, H-14′), 6.44 (1H, t, *J* = 2.3 Hz, H-12′), 6.76 (2H, d, *J* = 8.7 Hz, H-3′, H-5′), 6.94 (2H, s, H-10, H-14), 6.94 (1H, d, *J* = 8.3 Hz, H-5), 7.01 (1H, d, *J* = 16.4 Hz, H-8), 7.21 (3H, m, H-2, H-2′, H-6′), 7.35 (1H, d, *J* = 16.4 Hz, H-7), 7.47 (1H, dd, *J* = 8.3, 1.9 Hz, H-6), 9.54 (1H, s, 4′OH); ^13^C NMR (DMSO-*d*_6_, 151 MHz) δ 55.2 (CH_3_O-11′, CH_3_O-13′), 55.7 (C-8′), 56.3 (CH_3_O-11, CH_3_O-13), 92.1 (C-7′), 98.2 (C-12), 98.5 (C-12′), 102.9 (C-10, C-14), 106.4 (C-10′, C-14′), 109.6 (C-5), 115.3 (C-3′, C-5′), 122.9 (C-2), 125.2 (C-8), 127.7 (C-6), 128.0 (C-2′, C-6′), 129.4 (C-7), 129.9 (C-1′), 130.2 (C-1), 131.6 (C-3), 138.3 (C-9), 143.5 (C-9′), 156.5 (C-11, C-13), 157.6 (C-4′), 158.9 (C-4), 160.7 (C-11′, C-13′); HR-ESI/MS analysis: *m/z* 587.1072 [M-H]^−^, (calcd for C_32_H_28_BrO_6_^−^, 587.1069, ∆ = 0.6 ppm). MS/MS spectrum: CCMSLIB00010129257. SMILES: OC(C=C1)=CC=C1[C@@H](O2)[C@@H](C3=CC(OC)=CC(OC)=C3)C4=C2C=CC(/C=C/C5=CC(OC)=C(Br)C(OC)=C5)=C4.

14-bromo-11,11′,13,13′-tetra-*O*-methyl-*trans*-*δ*-viniferin (**41**): UV (MeOH) λ_max_ (log ε) 229 (sh) (4.47), 285 (sh) (4.00), 312 (4.24), 332 (4.23) nm; ^1^H NMR (DMSO-*d*_6_, 600 MHz) δ 3.70 (6H, s, CH_3_O-11′, CH_3_O-13′), 3.81 (3H, s, CH_3_O-11), 3.82 (3H, s, CH_3_O-13), 4.63 (1H, d, *J* = 8.3 Hz, H-8′), 5.61 (1H, d, *J* = 8.3 Hz, H-7′), 6.38 (2H, d, *J* = 2.3 Hz, H-10′, H-14′), 6.43 (1H, t, *J* = 2.3 Hz, H-12′), 6.57 (1H, d, *J* = 2.6 Hz, H-12), 6.76 (2H, d, *J* = 8.5 Hz, H-3′, H-5′), 6.95 (2H, m, H-5, H-10), 7.20 (4H, m, H-2, H-2′, H-6′, H-8), 7.26 (1H, d, *J* = 16.2 Hz, H-7), 7.50 (1H, dd, *J* = 8.4, 1.9 Hz, H-6), 9.54 (1H, s, 4′OH); ^13^C NMR (DMSO-*d*_6_, 151 MHz) δ 55.2 (CH_3_O-11′, CH_3_O-13′), 55.5 (C-8′), 55.6 (CH_3_O-11), 56.4 (CH_3_O-13), 92.1 (C-7′), 98.6 (C-12′), 99.1 (C-12), 102.5 (C-10), 103.6 (C-14), 106.2 (C-10′, C-14′), 109.7 (C-5), 115.3 (C-3′, C-5′), 123.5 (C-2), 123.9 (C-8), 127.6 (C-6), 127.9 (C-2′, C-6′), 129.9 (C-1′), 130.0 (C-1), 131.5 (C-3), 132.0 (C-7), 138.1 (C-9), 143.6 (C-9′), 156.4 (C-13), 157.6 (C-4′), 159.2 (C-4), 159.4 (C-11), 160.7 (C-11′, C-13′); HR-ESI/MS analysis: *m/z* 587.1069 [M-H]^−^, (calcd for C_32_H_28_BrO_6_^−^, 587.1069, ∆ = 0 ppm). MS/MS spectrum: CCMSLIB00010129256. SMILES: OC(C=C1)=CC=C1[C@@H](O2)[C@@H](C3=CC(OC)=CC(OC)=C3)C4=C2C=CC(/C=C/C5=C(Br)C(OC)=CC(OC)=C5)=C4.

14-chloro-11,11′,13,13′-tetra-*O*-methyl-*trans*-*δ*-viniferin (**42**): UV (MeOH) λ_max_ (log ε) 229 (sh) (4.63), 286 (sh) (4.20), 313 (4.45), 332 (4.44) nm; ^1^H NMR (DMSO-*d*_6_, 600 MHz) δ 3.70 (6H, s, CH_3_O-11′, CH_3_O-13′), 3.81 (3H, s, CH_3_O-11), 3.83 (3H, s, CH_3_O-13), 4.62 (1H, d, *J* = 8.3 Hz, H-8′), 5.61 (1H, d, *J* = 8.3 Hz, H-7′), 6.38 (2H, d, *J* = 2.3 Hz, H-10′, H-14′), 6.43 (1H, t, *J* = 2.3 Hz, H-12′), 6.59 (1H, d, *J* = 2.6 Hz, H-12), 6.76 (2H, d, *J* = 8.5 Hz, H-3′, H-5′), 6.95 (2H, m, H-5, H-10), 7.21 (4H, m, H-2, H-2′, H-6′, H-8), 7.30 (1H, d, *J* = 16.3 Hz, H-7), 7.51 (1H, dd, *J* = 8.4, 1.9 Hz, H-6), 9.54 (1H, s, 4′OH); ^13^C NMR (DMSO-*d*_6_, 151 MHz) δ 55.2 (CH_3_O-11′, CH_3_O-13′), 55.5 (C-8′), 55.6 (CH_3_O-11), 56.2 (CH_3_O-13), 92.1 (C-7′), 98.6 (C-12′), 99.2 (C-12), 101.8 (C-10), 106.2 (C-10′, C-14′), 109.7 (C-5), 112.3 (C-14), 115.3 (C-3′, C-5′), 121.1 (C-8), 123.6 (C-2), 127.7 (C-6), 127.9 (C-2′, C-6′), 130.0 (C-1), 130.0 (C-1′), 131.5 (C-3), 132.0 (C-7), 136.3 (C-9), 143.7 (C-9′), 155.6 (C-13), 157.6 (C-4′), 158.6 (C-11), 159.2 (C-4), 160.7 (C-11′, C-13′); HR-ESI/MS analysis: *m/z* 543.1568 [M-H]^−^, (calcd for C_32_H_28_ClO_6_^−^, 543.1574, ∆ = 1.2 ppm). MS/MS spectrum: CCMSLIB00010129267. SMILES: OC(C=C1)=CC=C1[C@@H](O2)[C@@H](C3=CC(OC)=CC(OC)=C3)C4=C2C=CC(/C=C/C5=C(Cl)C(OC)=CC(OC)=C5)=C4.

14-iodo-11,11′,13,13′-tetra-*O*-methyl-*trans*-*δ*-viniferin (**43**): UV (MeOH) λ_max_ (log ε) 287 (sh) (3.96), 312 (4.17), 335 (4.15) nm; ^1^H NMR (DMSO-*d*_6_, 600 MHz) δ 3.70 (6H, s, CH_3_O-11′, CH_3_O-13′), 3.81 (6H, s, CH_3_O-11, CH_3_O-13), 4.64 (1H, d, *J* = 8.4 Hz, H-8′), 5.61 (1H, d, *J* = 8.4 Hz, H-7′), 6.39 (2H, d, *J* = 2.3 Hz, H-10′, H-14′), 6.43 (1H, t, *J* = 2.3 Hz, H-12′), 6.50 (1H, d, *J* = 2.6 Hz, H-12), 6.76 (2H, d, *J* = 8.5 Hz, H-3′, H-5′), 6.92 (1H, d, *J* = 2.6 Hz, H-10), 6.96 (1H, d, *J* = 8.3 Hz, H-5), 7.15 (2H, m, H-7, H-8), 7.18 (1H, s, H-2), 7.21 (2H, d, *J* = 8.5 Hz, H-2′, H-6′), 7.49 (1H, dd, *J* = 8.3, 1.9 Hz, H-6), 9.54 (1H, s, 4′OH); ^13^C NMR (DMSO-*d*_6_, 151 MHz) δ 55.2 (CH_3_O-11′, CH_3_O-13′), 55.5 (C-8′), 56.5 (CH_3_O-11, CH_3_O-13), 81.9 (C-14), 92.0 (C-7′), 98.2 (C-12), 98.6 (C-12′), 103.0 (C-10), 106.2 (C-10′, C-14′), 109.7 (C-5), 115.3 (C-3′, C-5′), 123.5 (C-2), 127.4 (C-6), 127.9 (C-2′, C-6′), 129.4 (C-8), 129.9 (C-1), 130.0 (C-1′), 131.5 (C-3), 131.9 (C-7), 141.4 (C-9), 143.6 (C-9′), 157.6 (C-4′), 158.6 (C-13), 159.2 (C-4), 160.7 (C-11), 160.7 (C-11′, C-13′); HR-ESI/MS analysis: *m/z* 635.0920 [M-H]^−^, (calcd for C_32_H_28_IO_6_^−^, 635.0931, ∆ = 1.7 ppm). MS/MS spectrum: CCMSLIB00010129274. SMILES: OC(C=C1)=CC=C1[C@@H](O2)[C@@H](C3=CC(OC)=CC(OC)=C3)C4=C2C=CC(/C=C/C5=C(I)C(OC)=CC(OC)=C5)=C4.

12-iodo-11,11′,13,13′-tetra-*O*-methyl-*trans*-*δ*-viniferin (**44**): UV (MeOH) λ_max_ (log ε) 229 (sh) (4.56), 287 (sh) (4.10), 315 (4.45), 340 (4.52), 358 (sh) (4.28) nm; ^1^H NMR (DMSO-*d*_6_, 600 MHz) δ 3.70 (6H, s, CH_3_O-11′, CH_3_O-13′), 3.84 (6H, s, CH_3_O-11, CH_3_O-13), 4.62 (1H, d, *J* = 8.8 Hz, H-8′), 5.60 (1H, d, *J* = 8.8 Hz, H-7′), 6.39 (2H, d, *J* = 2.3 Hz, H-10′, H-14′), 6.44 (1H, t, *J* = 2.3 Hz, H-12′), 6.76 (2H, d, *J* = 8.5 Hz, H-3′, H-5′), 6.85 (2H, s, H-10, H-14), 6.94 (1H, d, *J* = 8.3 Hz, H-5), 7.02 (1H, d, *J* = 16.4 Hz, H-8), 7.21 (1H, d, *J* = 1.9 Hz, H-2), 7.21 (2H, d, *J* = 8.5 Hz, H-2′, H-6′), 7.37 (1H, d, *J* = 16.4 Hz, H-7), 7.47 (1H, dd, *J* = 8.3, 1.9 Hz, H-6), 9.53 (1H, s, 4′OH); ^13^C NMR (DMSO-*d*_6_, 151 MHz) δ 55.1 (CH_3_O-11′, CH_3_O-13′), 55.7 (C-8′), 56.5 (CH_3_O-11, CH_3_O-13), 75.6 (C-12), 92.1 (C-7′), 98.5 (C-12′), 102.4 (C-10, C-14), 106.4 (C-10′, C-14′), 109.5 (C-5), 115.2 (C-3′, C-5′), 122.9 (C-2), 125.2 (C-8), 127.7 (C-6), 127.9 (C-2′, C-6′), 129.4 (C-7), 129.9 (C-1′), 130.2 (C-1), 131.6 (C-3), 139.7 (C-9), 143.5 (C-9′), 157.6 (C-4′), 158.9 (C-4), 159.0 (C-11, C-13), 160.7 (C-11′, C-13′); HR-ESI/MS analysis: *m/z* 635.0923 [M-H]^−^, (calcd for C_32_H_28_IO_6_^−^, 635.0931, ∆ = 1.2 ppm). MS/MS spectrum: CCMSLIB00010129275. SMILES: OC(C=C1)=CC=C1[C@@H](O2)[C@@H](C3=CC(OC)=CC(OC)=C3)C4=C2C=CC(/C=C/C5=CC(OC)=C(I)C(OC)=C5)=C4.

### Minimum inhibitory concentration (MIC) determinations

MICs against *Staphylococcus aureus* strains Newman (MSSA), COL (MRSA) and a clinical isolate of *Staphylococcus epidermidis* were determined in Mueller–Hinton (MH) broth according to CLSI guidelines^35^ and were repeated at least three times.

### QSAR analysis

The set of compounds was first analyzed with the “R-group Analysis” tab from Maestro (Schrödinger Release 2021-1: Maestro, Schrödinger, LLC, New York, NY, 2021), considering the atomic number and aromaticity for atom equivalences and the bond order for bond equivalences. The resulting R groups analysis exclude the *cis* (**5**–**8**) and the benzofuran (**16**–**19**) compounds. We then created a dataset, coding the substituent for each compound and each R-group.

We then first analyzed the dataset with statistical tools consisting of the Smoothly Clipped Absolute Deviation (SCAD)—a modified version of the Lasso—^36^ and the high-dimensional BIC (HBIC)^37, 38^. We then built a linear model based on the variables previously found, where the response MIC was transformed into its log. The model with the smallest HBIC value was chosen, and the standard least squares estimator was computed on the selected variables. The R^2^ of this model is 0.84 and standard residual analysis suggested that there are no major deviations from the model assumptions.

### Supplementary Information

Supplementary Information.

## Data Availability

The raw data files of the NMR analysis are available at the following link: https://doi.org/10.26037/yareta:n2qfzsjylnb7pojbqiifrpukmm. The MS/MS spectrum of each isolated compound has its own accession number CCMSLIB00010129XXX on the Global Natural Product Social Molecular Networking (GNPS) (accessed via: https://gnps.ucsd.edu/ProteoSAFe/static/gnps-splash.jsp). The complete table with every compound generated and associated data (name, SMILE, molecular weight, MIC) can be found in supplementary Table S4 for reprocessing of the SAR analysis.
