# Supplementary material for: Generation of potent antibacterial compounds through enzymatic and chemical modifications of the trans-δ-viniferin scaffold
Source: Sci Rep. 2023 Sep 25;13:15986. doi: 10.1038/s41598-023-43000-5 (PMC10520035; doi:10.1038/s41598-023-43000-5)
Supplement: Supplementary file 1 — Supplementary Information. [file 41598_2023_43000_MOESM1_ESM.pdf]

Generation of potent antibacterial compounds through enzymatic and chemical modifications of the trans-δ-viniferin scaffold

Robin Huber,[1,2] Laurence Marcourt,[1,2] Margaux Héritier,[1,2] Alexandre Luscher,[3] Laurie Guebey,[3] Sylvain Schnee,[4] Emilie Michellod,[4] Stéphane Guerrier,[1,2,5] Jean-Luc Wolfender,[1,2] Leonardo Scapozza,[1,2] Thilo Köhler,[3] Katia Gindro,[4†*] and Emerson Ferreira Queiroz.[1,2†*]

[1] School of Pharmaceutical Sciences, University of Geneva, CMU, Geneva, Switzerland

[2] Institute of Pharmaceutical Sciences of Western Switzerland, University of Geneva, CMU, Geneva, Switzerland

[3] Department of Microbiology and Molecular Medicine, University of Geneva, Rue Michel-Servet 1, CH-1211 Genève 4, Switzerland

[4] Agroscope, Plant Protection Research Division, Mycology Group, Route de Duillier 50, P.O. Box 1012, 1260 Nyon, Switzerland

[5] University of Geneva, Geneva School of Economics and Management, Geneva 1205, Switzerland

[†]These authors have contributed equally to this work and share last authorship**,**

*Corresponding authors: Emerson Ferreira Queiroz (emerson.ferreira@unige.ch), Katia Gindro (katia.gindro@agroscope.admin.ch)

Supplementary Material

Supplementary methods 2

Benzofuran derivatives synthesis (compounds 16-19) 2

Bromination reactions (scale-up and isolation, compounds 20, 21, 26, 27, 33, 40 and 41) 6

Chlorination reactions (scale-up and isolation, compounds 22, 23, 28-30, 34-37 and 42) 7

Iodination reactions (scale-up and isolation, compounds 24, 25, 31, 32, 38, 39, 43, 44) 7

Supplementary Figures and Tables 9

NMR Data..24

Supplementary methods

Benzofuran derivatives synthesis (compounds 16-19)

To a solution of **1** (16.8 mg, 0.037 mmol, 1 eq) in DCM (5 mL) and DMSO (0.4 mL) were added Ac_2O (23 µL, 0.24 mmol, 6.6 eq) and TEA (76 µL, 0.54 mmol, 14.7 eq) and the mixture was stirred at room temperature overnight. Solvent was evaporated by rotary evaporation and the residue was solubilized in EtOAc (5 mL) and washed with H_2O (5 mL). The aqueous phase was extracted with EtOAc (2 × 5 mL). The combined organic layers were washed with water (10 mL), and dried under nitrogen (23.6 mg). The crude reaction mixture was analyzed by UHPLC-PDA-ELSD-MS. The purity of **1-Ac** was good enough to use it without purification for the next step. The crude reaction mixture containing **1-Ac** (22.6 mg, 0.034 mmol, 1 eq) was solubilized, under argon in dry toluene (10 mL) at room temperature. DDQ (77 mg, 0.34 mmol, 10 eq) was added and the mixture was heated at 80 °C. The reaction was monitored by UHPLC-PDA-ELSD-MS until consumption of the starting material (about 23h). The crude reaction mixture was directly purified by flash chromatography using a Scorpius C18e-HP column (125 × 28 mm i.d., 30 µm; BGB) at 25 ml/min, 40°C with H_2O (A) and MeOH (B) both containing 0.1% formic acid as solvents. A generic gradient was used (5 to 100% MeOH in 160 min). The targeted product **1-Ac-Ox** was collected (6.3 mg, t_R = 57 min). To a solution of **1-Ac-Ox** (5.2 mg, 0.0078 mmol, 1 eq) in MeOH (1 mL) at 0 °C was added KOH (4.4 mg, 0.078 mmol, 10 eq). The mixture was stirred for 2h at 0 °C, and then acidified with HCl 0.1 M until pH 1 was reached. MeOH was removed by rotary evaporation. 5 mL H_2O were added and extracted with EtOAc (3 x 10 mL). The combined organic layers were washed with water (10 mL), and dried under nitrogen, giving the targeted compound **16** (3.9 mg) without further purification.

1-Ac: ^{1}H NMR (DMSO-d_6, 600 MHz) δ 2.24 (6H, s), 2.27 (9H, s), 4.80 (1H, d, *J* = 7.3 Hz), 5.74 (1H, d, *J* = 7.3 Hz), 6.84 (1H, t, *J* = 2.1 Hz), 6.98 (3H, m), 7.02 (1H, d, *J* = 8.4 Hz), 7.04 (1H, d, *J* = 16.3 Hz), 7.15 (2H, d, *J* = 8.6 Hz), 7.22 (2H, d, *J* = 2.1 Hz), 7.27 (1H, s), 7.28 (1H, d, *J* = 16.3 Hz), 7.42 (2H, d, *J* = 8.6 Hz), 7.50 (1H, dd, *J* = 8.4, 1.8 Hz); HR-ESI/MS analysis: *m/z* 665.2024 $[M+H]^+$, (calcd for $C_{38}H_{33}O_{11}^+$, 665.2017, Δ = 1.0 ppm).

1-Ac-Ox: ^{1}H NMR (DMSO-d_6, 600 MHz) δ 2.28, 2.28, 2.29 (15H, 3 s), 6.88 (1H, t, *J* = 2.1 Hz), 7.15 (1H, t, *J* = 2.1 Hz), 7.20 (2H, d, *J* = 8.8 Hz), 7.24 (3H, m), 7.30 (2H, d, *J* = 2.1 Hz), 7.47 (1H, d, *J* = 16.3 Hz), 7.66 (3H, m), 7.73 (2H, d, *J* = 1.5 Hz); HR-ESI/MS analysis: *m/z* 663.1852 $[M+H]^+$, (calcd for $C_{38}H_{31}O_{11}^+$, 663.1861 , Δ = 1.3 ppm).

dehydro-*trans*-δ-viniferin (**16**): UV (MeOH) λ_{max} (log ε) 307 (4.34) nm; ^{1}H NMR (DMSO-d_6, 600 MHz) δ 6.14 (1H, t, *J* = 2.1 Hz, H-12), 6.27 (1H, t, *J* = 2.2 Hz, H-12'), 6.32 (2H, d, *J* = 2.2 Hz, H-10', H-14'), 6.44 (2H, d, *J* = 2.1 Hz, H-10, H-14), 6.79 (2H, d, *J* = 8.7 Hz, H-3', H-5'), 6.98 (1H, d, *J* = 16.3 Hz, H-8), 7.16 (1H, d, *J* = 16.3 Hz, H-7), 7.47 (2H, d, *J* = 8.7 Hz, H-2', H-6'), 7.53 (1H, s, H-2), 7.59 (2H, m, H-5, H-6), 9.22 (2H, s, 11OH, 13OH), 9.41 (2H, s, 11'OH, 13'OH), 9.86 (1H, s, 4'OH); ^{13}C NMR (DMSO, 151 MHz) δ 102.1 (C-12, C-12'), 104.6 (C-10, C-14), 107.3 (C-10', C-14'), 111.2 (C-5), 115.3 (C-8'), 115.6 (C-3', C-5'), 117.5 (C-2), 120.7 (C-1'), 123.0 (C-6), 127.9 (C-8), 128.2 (C-7),

128.3 (C-2', C-6'), 130.2 (C-3), 132.5 (C-1), 133.7 (C-9'), 139.0 (C-9), 150.9 (C-7'), 152.7 (C-4), 158.1 (C-4'), 158.5 (C-11, C-13), 159.0 (C-11', C-13'); HR-ESI/MS analysis: *m/z* 451.1175 [M - H]$^-$, (calcd for $C_{28}H_{19}O_6^-$, 451.1187 , Δ = 2.7 ppm). MS/MS spectrum: CCMSLIB00010129247. SMILES: OC1=CC=C(C(O2)=C(C3=CC(O)=CC(O)=C3)C4=C2C=CC(/C=C/C5=CC(O)=CC(O)=C5)=C4)C=C1.

To a solution of **2** (16.9 mg, 0.035 mmol, 1 eq) in DCM (5 mL) and DMSO (0.4 mL) were added Ac_2O (21.8 µL, 0.23 mmol, 6.6 eq) and TEA (72 µL, 0.51 mmol, 14.7 eq) and the mixture was stirred at room temperature overnight. Solvent was evaporated by rotary evaporation and the residue was solubilized in EtOAc (5 mL) and washed with H_2O (5 mL). The aqueous phase was extracted with EtOAc (2 × 5 mL). The combined organic layers were washed with water (10 mL), and dried under nitrogen (22.8 mg). The crude reaction mixture was analyzed by UHPLC-PDA-ELSD-MS. The purity of **2**-Ac was good enough to use it without purification for the next step. The crude reaction mixture containing **2-Ac** (22.8 mg, 0.0375 mmol, 1 eq) was solubilized, under argon in dry toluene (10 mL) at room temperature. DDQ (83 mg, 0.36 mmol, 10 eq) was added and the mixture was heated at 80 °C. The reaction was monitored by UHPLC-PDA-ELSD-MS until consumption of the starting material (about 16h). The crude reaction mixture was directly purified by flash chromatography using a Scorpius C18e-HP column (125 × 28 mm i.d., 30 µm; BGB) at 25 ml/min, 40°C with H_2O (A) and MeOH (B) both containing 0.1% formic acid as solvents. A generic gradient was used (5 to 100% MeOH in 160 min). The targeted product **2-Ac-Ox** was collected (8.8 mg, t_R = 59 min). To a solution of **2-Ac-Ox** (7.8 mg, 0.013 mmol, 1 eq) in MeOH (1 mL) at 0 °C was added KOH (7.2 mg, 0.128 mmol, 10 eq). The mixture was stirred for 2h at 0 °C, and then acidified with HCl 0.1 M until pH 1 was reached. MeOH was removed by rotary evaporation. 5 mL H_2O were added and extracted with EtOAc (3 x 10 mL). The combined organic layers were washed with water (10 mL), and dried under nitrogen, giving the targeted compound **17** (5.6 mg) without further purification.

2-Ac: ^{1}H NMR (DMSO-d_6, 600 MHz) δ 2.27 (9H, s), 3.71 (6H, s), 4.64 (1H, d, *J* = 7.9 Hz), 5.77 (1H, d, *J* = 7.9 Hz), 6.42 (2H, d, *J* = 2.2 Hz), 6.44 (1H, t, *J* = 2.2 Hz), 6.83 (1H, t, *J* = 2.1 Hz), 6.99 (1H, d, *J* = 8.3 Hz), 7.03 (1H, d, *J* = 16.4 Hz), 7.15 (2H, d, *J* = 8.6 Hz), 7.22 (2H, d, *J* = 2.1 Hz), 7.24 (1H, d, *J* = 1.6 Hz), 7.27 (1H, d, *J* = 16.5 Hz), 7.42 (2H, d, *J* = 8.6 Hz), 7.48 (1H, dd, *J* = 8.3, 1.6 Hz); HR-ESI/MS analysis: *m/z* 609.2130 [M+H]$^+$, (calcd for $C_{36}H_{33}O_9^+$, 609.2119 , Δ = 1.8 ppm).

2-Ac-Ox: ^{1}H NMR (DMSO-d_6, 600 MHz) δ 2.28 (3H, s), 2.28 (6H, s), 3.76 (6H, s), 6.62 (1H, t, *J* = 2.3 Hz), 6.64 (2H, d, *J* = 2.2 Hz), 6.88 (1H, t, *J* = 2.1 Hz), 7.22 (3H, m), 7.31 (2H, d, *J* = 2.1 Hz), 7.49 (1H, d, *J* = 16.3 Hz), 7.66 (3H, m), 7.72 (2H, m); HR-ESI/MS analysis: *m/z* 607.1956 [M+H]$^+$, (calcd for $C_{36}H_{31}O_9^+$, 607.1963 , Δ = 1.1 ppm).

11',13'-di-*O*-methyl-dehydro-*trans*-δ-viniferin (**17**): UV (MeOH) λ_{max} (log ε) 306 (4.51) nm; ^{1}H NMR (DMSO-d_6, 600 MHz) δ 3.76 (6H, s, CH_3O-11', CH_3O-13'), 6.15 (1H, t, *J* = 2.1 Hz, H-12), 6.44 (2H, d, *J* = 2.1 Hz, H-10, H-14), 6.59 (1H, t, *J* = 2.3 Hz, H-12'), 6.61 (2H, d, *J* = 2.3 Hz, H-10', H-14'), 6.79 (2H, d, *J* = 8.7 Hz, H-3', H-5'), 7.00 (1H, d, *J* = 16.3 Hz, H-8), 7.18 (1H, d, *J* = 16.3 Hz, H-7), 7.45 (2H, d, *J* = 8.7 Hz, H-2', H-6'), 7.57 (1H, d, *J* = 1.6 Hz, H-2), 7.61 (1H, d, *J* = 8.6 Hz, H-5), 7.63 (1H, dd, *J* = 8.6, 1.6 Hz, H-6), 9.22 (2H, s, 11OH, 13OH), 9.89 (1H, s, 4'OH); ^{13}C NMR (DMSO-d_6, 151

MHz) δ 55.3 (CH_3O-11', CH_3O-13'), 99.8 (C-12'), 102.1 (C-12), 104.7 (C-10, C-14), 107.4 (C-10', C-14'), 111.2 (C-5), 115.0 (C-8'), 115.6 (C-3', C-5'), 117.7 (C-2), 120.5 (C-1'), 122.9 (C-6), 127.9 (C-8), 128.3 (C-7), 128.4 (C-2', C-6'), 130.0 (C-3), 132.7 (C-1), 134.1 (C-9'), 139.0 (C-9), 151.3 (C-7'), 152.7 (C-4), 158.2 (C-4'), 158.5 (C-11, C-13), 161.0 (C-11', C-13'); HR-ESI/MS analysis: *m/z* 479.1490 $[M - H]^-$, (calcd for $C_{30}H_{23}O_6^-$, 479.1500 , Δ = 2.1 ppm). MS/MS spectrum: CCMSLIB00010129248. SMILES: OC1=CC=C(C(O2)=C(C3=CC(OC)=CC(OC)=C3)C4=C2C=CC(/C=C/C5=CC(O)=CC(O)=C5)=C4)C=C1.

To a solution of **3** (17.0 mg, 0.035 mmol, 1 eq) in DCM (5 mL) and DMSO (0.4 mL) were added Ac_2O (22 µL, 0.23 mmol, 6.6 eq) and TEA (72 µL, 0.51 mmol, 14.7 eq) and the mixture was stirred at room temperature overnight. Solvent was evaporated by rotary evaporation and the residue was solubilized in EtOAc (5 mL) and washed with H_2O (5 mL). The aqueous phase was extracted with EtOAc (2 × 5 mL). The combined organic layers were washed with water (10 mL), and dried under nitrogen (26.1 mg). The crude reaction mixture was analyzed by UHPLC-PDA-ELSD-MS. The purity was good enough to use it without purification for the next step. The crude reaction mixture containing **3-Ac** (25 mg, 0.041 mmol, 1 eq) was solubilized, under argon in dry toluene (10 mL) at room temperature. DDQ (93 mg, 0.41 mmol, 10 eq) was added and the mixture was heated at 80 °C. The reaction was monitored by UHPLC-PDA-ELSD-MS until consumption of the starting material (about 19h). The crude reaction mixture was directly purified by flash chromatography using a Scorpius C18e-HP column (125 × 28 mm i.d., 30 µm; BGB) at 25 ml/min, 40°C with H_2O (A) and MeOH (B) both containing 0.1% formic acid as solvents. A generic gradient was used (5 to 100% MeOH in 160 min). The targeted product **3-Ac-Ox** (10.7 mg, t_R = 67 min) was collected To a solution of **3-Ac-Ox** (9.7 mg, 0.016 mmol, 1 eq) in MeOH (1 mL) at 0 °C was added KOH (9.0 mg, 0.160 mmol, 10 eq). The mixture was stirred for 2h at 0 °C, and then acidified with HCl 0.1 M until pH 1 was reached. MeOH was removed by rotary evaporation. 5 mL H_2O were added and extracted with EtOAc (3 x 10 mL). The combined organic layers were washed with water (10 mL), and dried under nitrogen, giving the targeted compound **18** (7.2 mg) without further purification.

3-Ac: ^1H NMR (DMSO-d_6, 600 MHz) δ 2.24 (6H, s), 2.27 (3H, s), 3.74 (6H, s), 4.79 (1H, d, *J* = 7.4 Hz), 5.73 (1H, d, *J* = 7.4 Hz), 6.35 (1H, t, *J* = 2.2 Hz), 6.72 (2H, d, *J* = 2.2 Hz), 6.98 (4H, m), 7.01 (1H, d, *J* = 8.3 Hz), 7.15 (2H, d, *J* = 8.5 Hz), 7.25 (1H, d, *J* = 1.9 Hz), 7.41 (2H, d, *J* = 8.5 Hz), 7.50 (1H, dd, *J* = 8.3, 1.9 Hz); HR-ESI/MS analysis: *m/z* 609.2125 $[M+H]^+$, (calcd for $C_{36}H_{33}O_9^+$, 609.2119 , Δ = 1.0 ppm).

3-Ac-Ox: ^1H NMR (DMSO-d_6, 600 MHz) δ 2.28 (6H, s), 2.28 (3H, s), 3.77 (6H, s), 6.40 (1H, t, *J* = 2.2 Hz), 6.79 (2H, d, *J* = 2.2 Hz), 7.16 (2H, m), 7.21 (2H, d, *J* = 8.7 Hz), 7.25 (2H, d, *J* = 2.1 Hz), 7.42 (1H, d, *J* = 16.3 Hz), 7.64 (1H, s), 7.66 (2H, d, *J* = 8.7 Hz), 7.72 (2H, m); HR-ESI/MS analysis: *m/z* 607.1953 $[M+H]^+$, (calcd for $C_{36}H_{31}O_9^+$, 607.1963 , Δ = 1.6 ppm).

11,13-di-*O*-methyl-dehydro-*trans*-δ-viniferin (**18**): UV (MeOH) λ_{max} (log ε) 306 (4.49) nm; ^1H NMR (DMSO-d_6, 600 MHz) δ 3.77 (6H, s, CH_3O-11, CH_3O-13), 6.28 (1H, t, *J* = 2.2 Hz, H-12'), 6.32 (2H, d, *J* = 2.2 Hz, H-10', H-14'), 6.38 (1H, t, *J* = 2.2 Hz, H-12), 6.80 (2H, d, *J* = 8.6 Hz, H-3', H-5'), 6.80

(2H, d, J = 2.2 Hz, H-10, H-14), 7.11 (1H, d, J = 16.4 Hz, H-8), 7.41 (1H, d, J = 16.4 Hz, H-7), 7.47 (2H, d, J = 8.6 Hz, H-2', H-6'), 7.56 (1H, d, J = 1.6 Hz, H-2), 7.61 (1H, d, J = 8.5 Hz, H-5), 7.63 (1H, dd, J = 8.5, 1.6 Hz, H-6), 9.42 (2H, s, 11'OH, 13'OH), 9.87 (1H, s, 4'OH); ^{13}C NMR (DMSO-d_6, 151 MHz) δ 55.2 (CH_3O-11, CH_3O-13), 99.9 (C-12), 102.1 (C-12'), 104.3 (C-10, C-14), 107.4 (C-10', C-14'), 111.3 (C-5), 115.3 (C-8'), 115.6 (C-3', C-5'), 117.8 (C-2), 120.7 (C-1'), 122.8 (C-6), 127.3 (C-8), 128.3 (C-2', C-6'), 129.3 (C-7), 130.4 (C-3), 132.4 (C-1), 133.7 (C-9'), 139.4 (C-9), 150.9 (C-7'), 152.8 (C-4), 158.1 (C-4'), 159.0 (C-11', C-13'), 160.6 (C-11, C-13); HR-ESI/MS analysis: *m/z* 479.1489 [M - H]$^-$, (calcd for $C_{30}H_{23}O_6^-$, 479.1500, Δ = 2.3 ppm). MS/MS spectrum: CCMSLIB00010129249. SMILES: OC1=CC=C(C(O2)=C(C3=CC(O)=CC(O)=C3)C4=C2C=CC(/C=C/C5=CC(OC)=CC(OC)=C5)=C4)C=C1.

To a solution of **4** (20.0 mg, 0.04 mmol, 1 eq) in DCM (5 mL) and DMSO (0.4 mL) were added Ac_2O (5.5 µL, 0.06 mmol, 1.5 eq) and TEA (16.3 µL, 0.12 mmol, 3 eq) and the mixture was stirred at room temperature overnight. Solvent was evaporated by rotary evaporation and the residue was solubilized in EtOAc (5 mL) and washed with H_2O (5 mL). The aqueous phase was extracted with EtOAc (2 × 5 mL). The combined organic layers were washed with water (10 mL), and dried under nitrogen (27.8 mg). The crude reaction mixture was analyzed by UHPLC-PDA-ELSD-MS. The purity was good enough to use it without purification for the next step. The crude reaction mixture containing **4-Ac** (10 mg, 0.018 mmol, 1 eq) was solubilized, under argon in dry toluene (10 mL) at room temperature. DDQ (41 mg, 0.18 mmol, 10 eq) was added and the mixture was heated at 80 °C. The reaction was monitored by UHPLC-PDA-ELSD-MS until consumption of the starting material (about 23h). The crude reaction mixture was directly purified by flash chromatography using a Scorpius C18e-HP column (125 × 28 mm i.d., 30 µm; BGB) at 25 ml/min, 40°C with H_2O (A) and MeOH (B) both containing 0.1% formic acid as solvents. A generic gradient was used (5 to 100% MeOH in 160 min). The targeted product **4-Ac-Ox** was collected (5.3 mg, t_R = 64 min). To a solution of **4-Ac-Ox** (3.9 mg, 0.0071 mmol, 1 eq) in MeOH (1 mL) at 0 °C was added KOH (3.9 mg, 0.071 mmol, 10 eq). The mixture was stirred for 2h at 0 °C, and then acidified with HCl 0.1 M until pH 1 was reached. MeOH was removed by rotary evaporation. 5 mL H_2O were added and extracted with EtOAc (3 x 10 mL). The combined organic layers were washed with water (10 mL), and dried under nitrogen, giving the targeted compound **19** (3.4 mg) without further purification.

4-Ac: ^{1}H NMR (DMSO-d_6, 600 MHz) δ 2.27 (3H, s), 3.71 (6H, s), 3.74 (6H, s), 4.63 (1H, d, J = 8.0 Hz), 5.77 (1H, d, J = 8.0 Hz), 6.35 (1H, t, J = 2.3 Hz), 6.42 (2H, d, J = 2.2 Hz), 6.45 (1H, t, J = 2.2 Hz), 6.72 (2H, d, J = 2.3 Hz), 6.96 (1H, d, J = 16.3 Hz), 6.98 (1H, d, J = 8.3 Hz), 7.15 (2H, d, J = 8.7 Hz), 7.22 (1H, d, J = 1.7 Hz), 7.22 (1H, d, J = 16.3 Hz), 7.41 (2H, d, J = 8.7 Hz), 7.48 (1H, dd, J = 8.3); HR-ESI/MS analysis: *m/z* 553.2211 [M+H]$^+$, (calcd for $C_{34}H_{33}O_7^+$, 553.2221 , Δ = 1.8 ppm).

4-Ac-Ox: ^{1}H NMR (DMSO-d_6, 600 MHz) δ 2.28 (3H, s), 3.76 (6H, s), 3.77 (6H, s), 6.39 (1H, t, J = 2.3 Hz), 6.63 (3H, m), 6.81 (2H, d, J = 2.3 Hz), 7.15 (1H, d, J = 16.4 Hz), 7.21 (2H, d, J = 8.6 Hz), 7.44 (1H, d, J = 16.4 Hz), 7.61 (1H, d, J = 1.7 Hz), 7.65 (2H, d, J = 8.6 Hz), 7.69 (1H, d, J = 8.6 Hz),

7.72 (1H, dd, *J* = 8.6, 1.7 Hz); HR-ESI/MS analysis: *m/z* 551.2056 [M+H]$^+$, (calcd for $C_{34}H_{31}O_7^+$, 551.2064, Δ = 1.5 ppm).

11,11',13,13'-tetra-*O*-methyl-dehydro-*trans*-δ-viniferin (**19**): UV (MeOH) λ_{max} (log ε) 307 (4.51) nm; ^{1}H NMR (DMSO-d_6, 600 MHz) δ 3.76 (6H, s, CH_3O-11', CH_3O-13'), 3.77 (6H, s, CH_3O-11, CH_3O-13), 6.38 (1H, t, *J* = 2.3 Hz, H-12), 6.60 (3H, m, H-10', H-12', H-14'), 6.79 (2H, d, *J* = 8.7 Hz, H-3', H-5'), 6.80 (2H, d, *J* = 2.3 Hz, H-10, H-14), 7.13 (1H, d, *J* = 16.4 Hz, H-8), 7.42 (1H, d, *J* = 16.4 Hz, H-7), 7.45 (2H, d, *J* = 8.7 Hz, H-2', H-6'), 7.57 (1H, d, *J* = 1.6 Hz, H-2), 7.63 (1H, d, *J* = 8.6 Hz, H-5), 7.66 (1H, dd, *J* = 8.6, 1.6 Hz, H-6), 9.88 (1H, s, 4'OH); ^{13}C NMR (DMSO-d_6, 151 MHz) δ 55.2 (CH_3O-11, CH_3O-13), 55.3 (CH_3O-11', CH_3O-13'), 99.7 (C-12'), 99.8 (C-12), 104.3 (C-10, C-14), 107.5 (C-10', C-14'), 111.3 (C-5), 115.0 (C-8'), 115.6 (C-3', C-5'), 117.9 (C-2), 120.5 (C-1'), 122.7 (C-6), 127.3 (C-8), 128.3 (C-2', C-6'), 129.3 (C-7), 130.2 (C-3), 132.6 (C-1), 134.1 (C-9'), 139.4 (C-9), 151.3 (C-7'), 152.8 (C-4), 158.2 (C-4'), 160.6 (C-11, C-13), 161.0 (C-11', C-13'); HR-ESI/MS analysis: *m/z* 509.1921 [M + H]$^+$, (calcd for $C_{32}H_{29}O_6^+$, 509.1959 , Δ = 7.2 ppm). MS/MS spectrum: CCMSLIB00010129250. SMILES: OC1=CC=C(C(O2)=C(C3=CC(OC)=CC(OC)=C3)C4=C2C=CC(/C=C/C5=CC(OC)=CC(OC)=C5)=C4)C=C1.

Bromination reactions (scale-up and isolation, compounds 20, 21, 26, 27, 33, 40 and 41)
10 mg **1**, **2**, **3** or **4** were solubilized in 2.6 mL of a MeCN:H_2O 50:50 mixture in a 10 mL round-bottom flask. The mixture was placed at 40 °C and stirred. 1 eq. of NaBr was added, followed by 2 mL of CH_3COOH and 21 eq. of H_2O_2 added dropwise. After 4h, 8 mL of EtOAc and 8 mL of H_2O were added. The organic phase was recovered and the aqueous was extracted with 2 x 3 mL of EtOAc. The combined organic layers were dried and analyzed by UHPLC-PDA-ELSD-MS. The crude reaction mixtures obtained after the bromination reactions were fractionated using semi-preparative reverse-phase chromatography. Two different stationary phases were used: C_{18} and phenyl. The same procedure was applied to every reaction crude mixture: the separation was first optimized at the analytical level on a HP 1260 Agilent High-Performance liquid chromatography equipped with a photodiode array detector (HPLC-PDA) (Agilent technologies, Santa Clara, CA, USA). The injections were performed on a XBridge C_{18} column (250 × 4.6 mm i.d., 5 µm; Waters, Milford, MA, USA) or a Zorbax XDB-Phenyl column (250 × 4.6 mm i.d., 5 µm; Agilent, Santa-Clara, CA, USA) at 1 mL/min, with H_2O (A) and MeCN (B) both containing 0.1% formic acid as solvents. Once a good separation was achieved, the optimized conditions were geometrically transferred to the semi-preparative scale by using the same stationary phase (see Table S3, for each chromatographic condition). The semi-preparative injections were performed on a Shimadzu system equipped with an LC-20 A module pumps, an SPD-20 A UV/VIS, a 7725I Rheodyne® valve, and an FRC-40 fraction collector (Shimadzu, Kyoto, Japan). The injections were performed on a XBridge C_{18} column (250 mm × 10 mm i.d., 5 µm; Waters, Milford, MA, USA) or an Eclipse XDB-Phenyl column (250 × 9.4 mm i.d., 5 µm; Agilent technologies, Santa Clara, CA, USA) at 4.7 mL/min (C_{18}) and 4 mL/min (phenyl), with H_2O (A) and MeCN (B) both containing 0.1% formic acid as solvents. The UV detection was set at 320 nm. The mixtures were injected on the semi-preparative HPLC column using a dry load (Queiroz et al., 2019). Fractions were collected, analyzed by UHPLC-PDA-ELSD-MS and combined according to their composition, affording compounds **20** (1.8 mg, 16% yield t_R = 19.0 min), **21** (0.6 mg, 5% yield,

t_R = 28.5 min), **26** (2.6 mg, 24% yield, t_R = 25 min), **27** (0.2 mg, 2% yield, t_R = 32 min), **33** (0.4 mg, 3% yield, t_R = 40 min), **40** (0.3 mg, 2% yield, t_R = 17.5 min), and **41** (2.7 mg, 21% yield, t_R = 19 min).

Chlorination reactions (scale-up and isolation, compounds 22, 23, 28-30, 34-37 and 42)
10 mg of **1**, **2**, **3** or **4** were solubilized in 2.6 mL of a MeCN:H_2O 50:50 mixture in a 10 mL round-bottom flask. The mixture was placed at 40 °C and stirred. 100 eq. of NaCl were added, followed by 2 mL of CH_3COOH and 21 eq. of H_2O_2 added dropwise. After 6h30, 8 mL of EtOAc and 8 mL of H_2O were added. The organic phase was recovered and the aqueous was extracted with 2 x 3 mL of EtOAc. The combined organic layers were dried and analyzed by UHPLC-PDA-ELSD-MS. The crude reaction mixtures obtained after the chlorination reactions were fractionated using semi-preparative reverse-phase chromatography on phenyl phase. The same procedure was applied to every reaction crude mixture: the separation was first optimized at the analytical level on a HP 1260 Agilent High-Performance liquid chromatography equipped with a photodiode array detector (HPLC-PDA) (Agilent technologies, Santa Clara, CA, USA). The injections were performed on a Zorbax XDB-Phenyl column (250 × 4.6 mm i.d., 5 µm; Agilent, Santa-Clara, CA, USA) at 1 mL/min, with H_2O (A) and MeCN (B) both containing 0.1% formic acid as solvents. Once a good separation was achieved, the optimized conditions were geometrically transferred to the semi-preparative scale by using the same stationary phase (see Table S3, for each chromatographic condition). The semi-preparative injections were performed on a Shimadzu system equipped with an LC-20 A module pumps, an SPD-20 A UV/VIS, a 7725I Rheodyne® valve, and an FRC-40 fraction collector (Shimadzu, Kyoto, Japan). The injections were performed on an Eclipse XDB-Phenyl column (250 × 9.4 mm i.d., 5 µm; Agilent technologies, Santa Clara, CA, USA) at 4 mL/min, with H_2O (A) and MeCN (B) both containing 0.1% formic acid as solvents. The UV detection was set at 320 nm. The mixtures were injected on the semi-preparative HPLC column using a dry load (Queiroz et al., 2019). Fractions were collected, analyzed by UHPLC-PDA-ELSD-MS and combined according to their composition, affording compounds **22** (3.1 mg, 28% yield, t_R = 39 min), **23** (0.6 mg, 5% yield, t_R = 55 min), **28** (3.7 mg, 34% yield, t_R = 36 min), **29** (0.5 mg, 5% yield, t_R = 39.5 min), **30** (0.7 mg, 6% yield, t_R = 47 min), **36** (0.2 mg, 2% yield, t_R = 29.5 min), **37** (1.1 mg, 8% yield, t_R = 33 min), and **42** (2.4 mg, 27% yield, t_R = 33.5 min).

In the case of compound **3** chlorination, the region between 25 and 28 min was collected together because the peaks were not sufficiently separated. Optimization on C_{18} or phenyl stationary phases did not allow their separation. A pentafluorophenyl (PFP) HPLC column (ACE C_{18}-PFP 250 x 4.6 mm i.d., 5 µm, Avantor, Radnor Township, PA, USA) was also tested, but gave similar results (data not shown). This fraction was further purified using a FCV-12AH Shimadzu recycling valve on the Shimadzu system described above. Isocratic conditions at 50% MeCN were used on an Eclipse XDB-Phenyl column (250 × 9.4 mm i.d., 5 µm; Agilent technologies, Santa Clara, CA, USA) at 4 mL/min. Peaks were collected after 14 rounds in the column allowing the isolation of **34** (0.8 mg, 6% yield, t_R = 267 min) and **35** (0.8 mg, 6% yield, t_R = 274 min).

Iodination reactions (scale-up and isolation, compounds 24, 25, 31, 32, 38, 39, 43, 44)
10 mg of **1**, **2**, **3** or **4** were solubilized in 2.6 mL of a MeCN:H_2O 50:50 mixture in a 10 mL round-bottom flask. The mixture was placed at 40 °C and stirred. 0.5 eq. of NaI were added, followed by 2

mL of CH_3COOH and 21 eq. of H_2O_2 added dropwise. After 2h30, 8 mL of EtOAc and 8 mL of H_2O were added. The organic phase was recovered and the aqueous was extracted with 2 x 3 mL of EtOAc. The combined organic layers were dried and analyzed by UHPLC-PDA-ELSD-MS. The crude reaction mixtures obtained after the iodination reactions were fractionated using semi-preparative reverse-phase chromatography on phenyl phase. The same procedure was applied to every reaction crude mixture: the separation was first optimized at the analytical level on a HP 1260 Agilent High-Performance liquid chromatography equipped with a photodiode array detector (HPLC-PDA) (Agilent technologies, Santa Clara, CA, USA). The injections were performed on a Zorbax XDB-Phenyl column (250 × 4.6 mm i.d., 5 µm; Agilent, Santa-Clara, CA, USA) at 1 mL/min, with H_2O (A) and MeCN (B) both containing 0.1% formic acid as solvents. Once a good separation was achieved, the optimized conditions were geometrically transferred to the semi-preparative scale by using the same stationary phase (see Table S3, for each chromatographic condition). The semi-preparative injections were performed on a Shimadzu system equipped with an LC-20 A module pumps, an SPD-20 A UV/VIS, a 7725I Rheodyne® valve, and an FRC-40 fraction collector (Shimadzu, Kyoto, Japan). The injections were performed on an Eclipse XDB-Phenyl column (250 × 9.4 mm i.d., 5 µm; Agilent technologies, Santa Clara, CA, USA) at 4 mL/min, with H_2O (A) and MeCN (B) both containing 0.1% formic acid as solvents. The UV detection was set at 320 nm. The mixtures were injected on the semi-preparative HPLC column using a dry load (Queiroz et al., 2019). Fractions were collected, analyzed by UHPLC-PDA-ELSD-MS and combined according to their composition, affording compounds **24** (0.1 mg, 1% yield, t_R = 30 min), **25** (2.2 mg, 14% yield, t_R = 32.5 min), **31** (0.4 mg, 3% yield, t_R = 28.5 min), **32** (3.5 mg, 24% yield, t_R = 33 min), **38** (0.3 mg, 2% yield, t_R = 32 min), **39** (3.5 mg, 21% yield, t_R = 39.5 min), **43** (1.5 mg, 15% yield, t_R = 23 min), and **44** (1.1 mg, 11% yield, t_R = 26 min).

Supplementary Figures and Tables

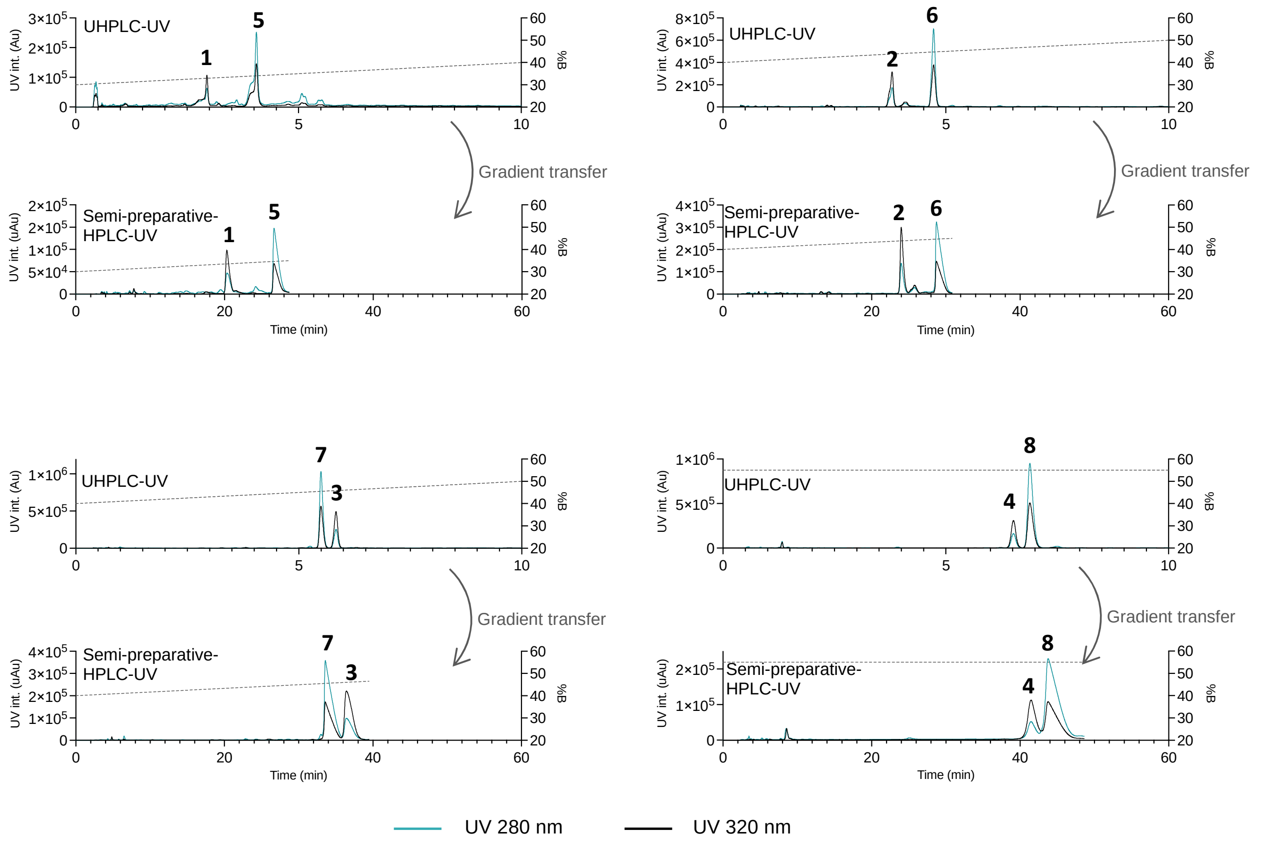

Figure S1. Isolation of *cis* isomers of *trans*-δ-viniferin derivatives. UHPLC-UV optimization (C_{18} 100 x 2.1 mm i.d., 1.7 µm) and transfer to semi-preparative HPLC-UV (C_{18} 250 x 19 mm i.d., 5 µm) for mixtures of compounds **1** and **5**; **2** and **6**; **3** and **7**; **4** and **8**. Differences in absorption between 280 and 320 nm allow the identification of *cis* and *trans* isomers.

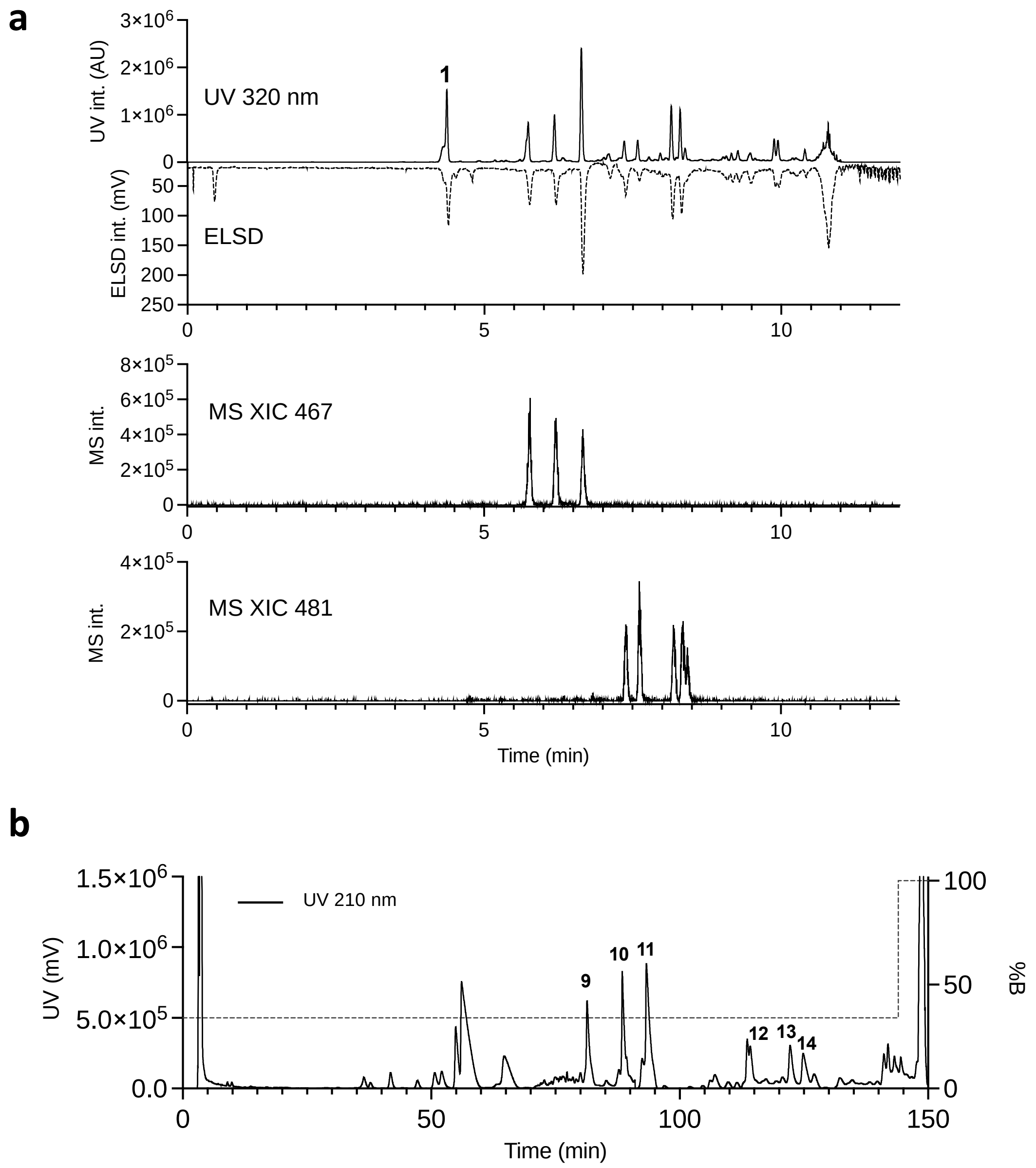

Figure S2. Synthesis and isolation of *O*-methylated derivatives of *trans*-δ-viniferin. (**a**) Reaction monitoring on UHPLC-PDA-ELSD-MS (C_{18} 100 x 2.1 mm i.d., 1.7 µm, gradient from 20 to 100% MeCN in 10 min). Extracted ion chromatograms (XIC) allow easy localisation of mono- (*m/z* 467) and di- (*m/z* 481) *O*-methylated derivatives. (**b**) Semi-preparative HPLC-UV separation (C_{18} 250 x 19 mm i.d., 5 µm) using isocratic conditions (34% MeCN).

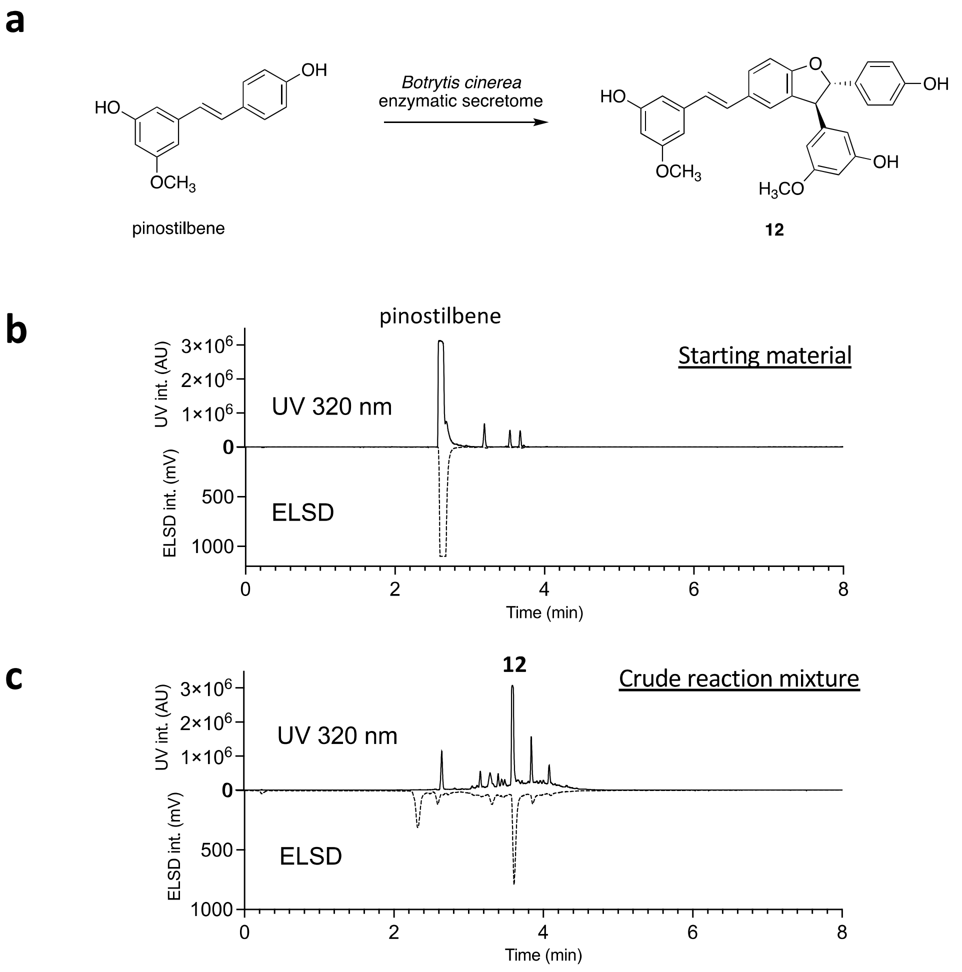

Figure S3. Synthesis of compound 12 using a chemoenzymatic approach. (**a**) Reaction scheme. (**b**) UHPLC-PDA-ELSD (C_{18} 50 x 2.1 mm i.d., 1.7 µm) analysis of the starting material pinostilbene. (**c**) Analysis of the crude reaction mixture using the same instrument. Compound **12** is the main product, as shown by the ELSD analysis.

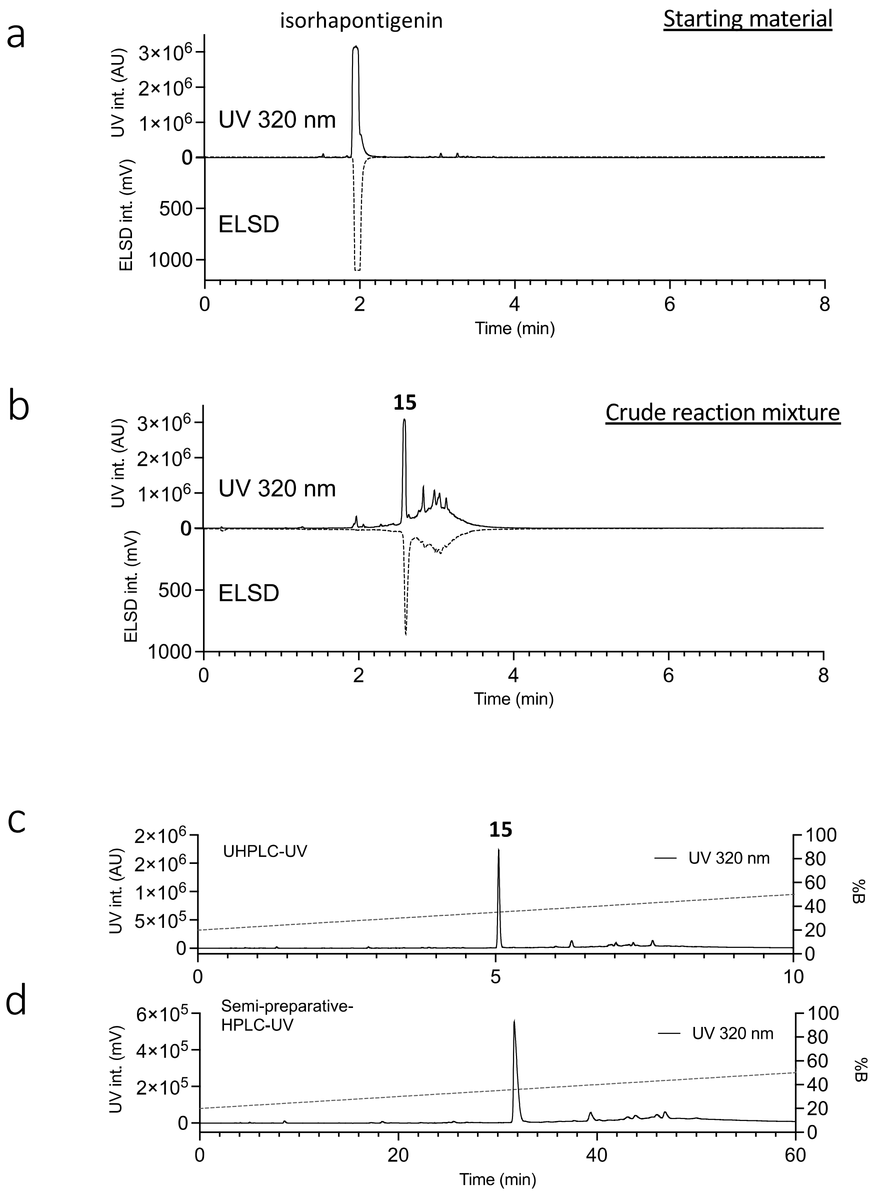

Figure S4. Synthesis of compound 15 by a chemoenzymatic approach. (**a**) UHPLC-PDA-ELSD (C_{18} 50 x 2.1 mm i.d., 1.7 µm) analysis of the starting material isorhapontigenin. (**b**) Analysis of the crude reaction mixture using the same instrument. Compound **15** is the main product, as shown by the ELSD analysis. (**c**) UHPLC-UV optimization (C_{18} 100 x 2.1 mm i.d., 1.7 µ) and (**d**) Semi-preparative HPLC-UV run (C_{18} 250 x 19 mm i.d., 5 µm).

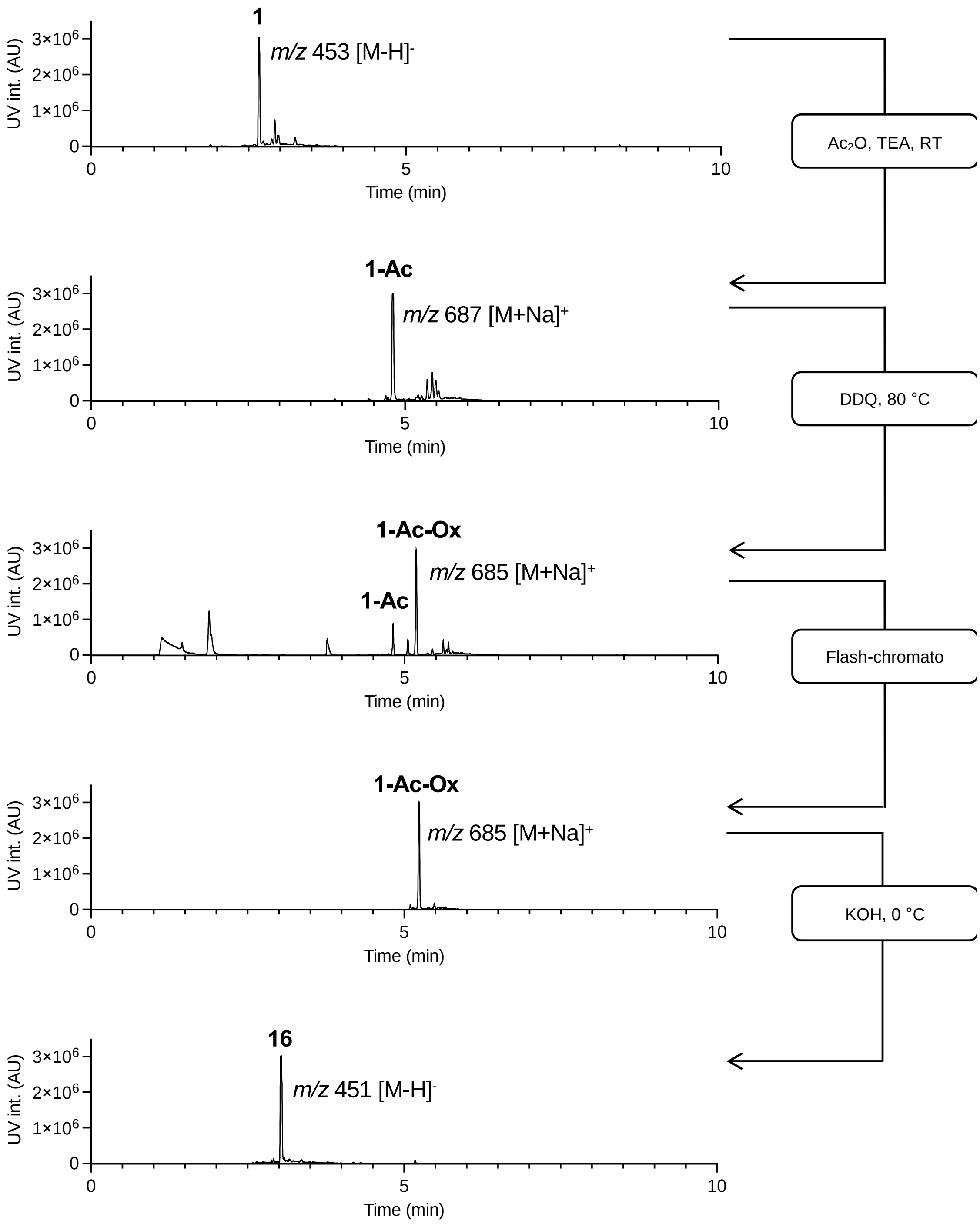

Figure S5. UHPLC (C_{18} 50 x 2.1 mm i.d., 1.7 µm) monitoring of the different steps in the synthesis of compound **16**. Detailed reaction conditions are given in the material and methods section.

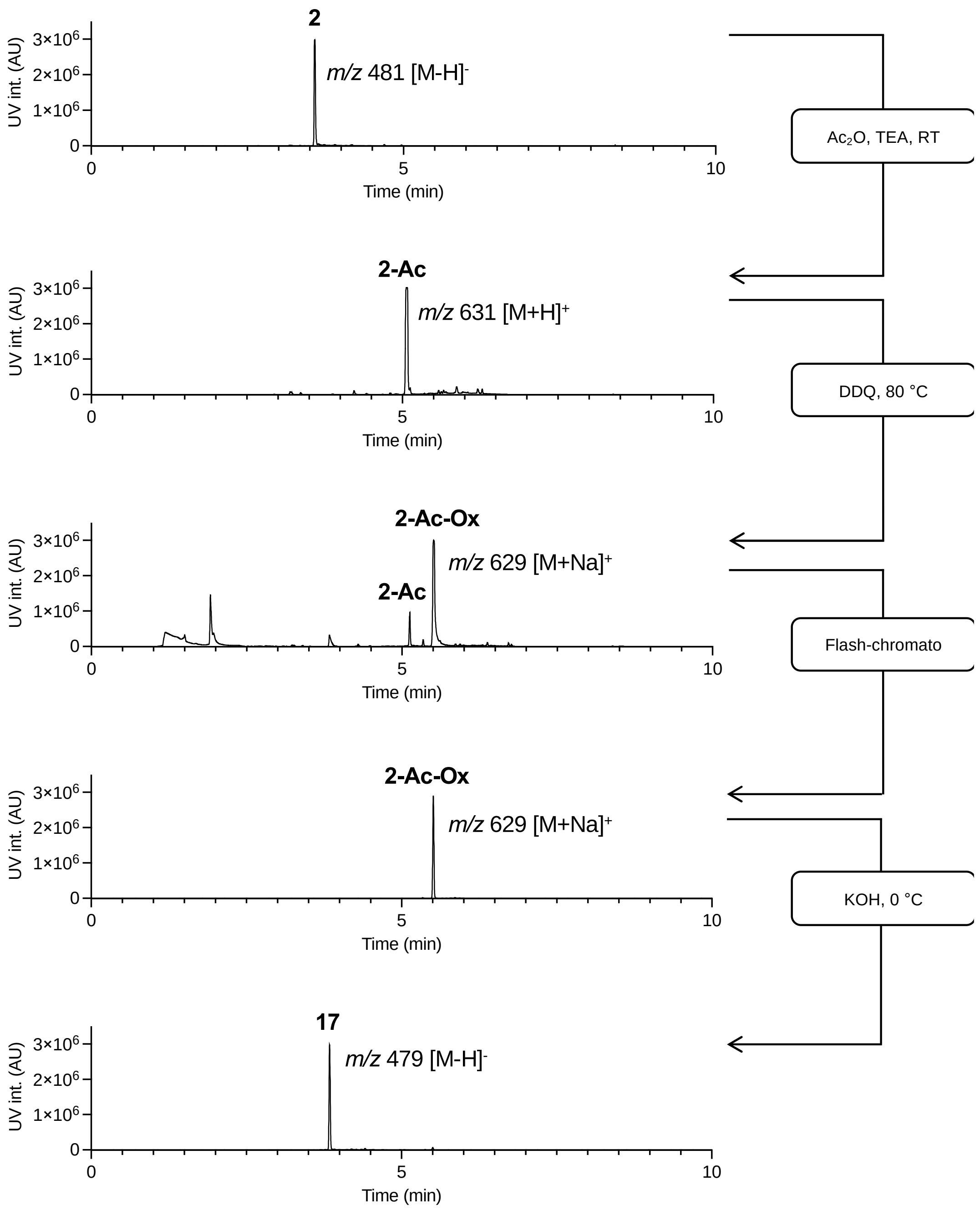

Figure S6. UHPLC (C_{18} 50 x 2.1 mm i.d., 1.7 µm) monitoring of the different steps in the synthesis of compound **17**. Detailed reaction conditions are given in the material and methods section.

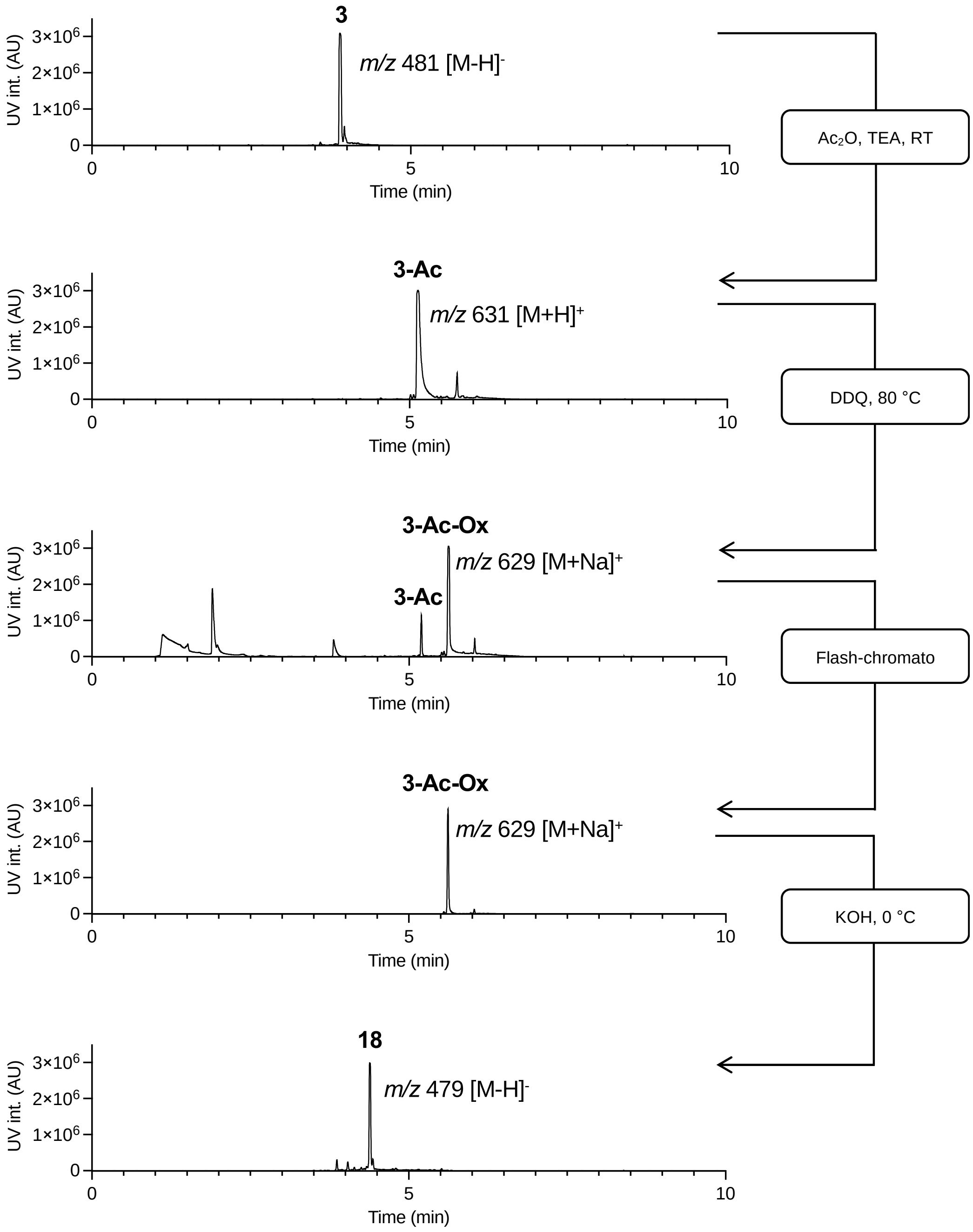

Figure S7. UHPLC (C_{18} 50 x 2.1 mm i.d., 1.7 µm) monitoring of the different steps in the synthesis of compound **18**. Detailed reaction conditions are given in the material and methods section.

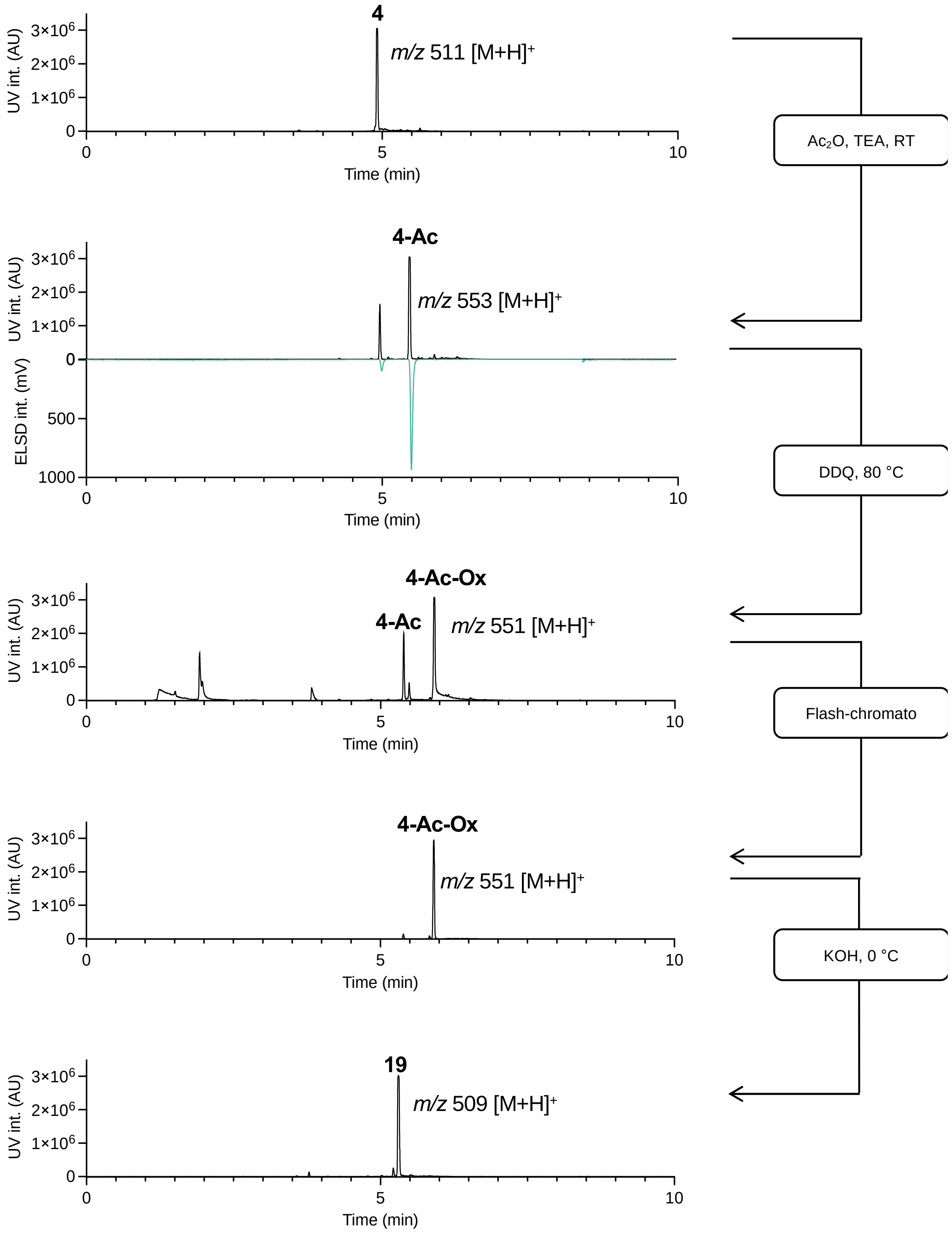

Figure S8. UHPLC (C_{18} 50 x 2.1 mm i.d., 1.7 µm) monitoring of the different steps in the synthesis of compound **19**. Detailed reaction conditions are given in the material and methods section. ELSD detection is shown for the acetylated compound **4-Ac** to highlight that it is the main product, as the UV absorption at 320 mm appears to show an almost 2:3 ratio with the starting compound **4**.

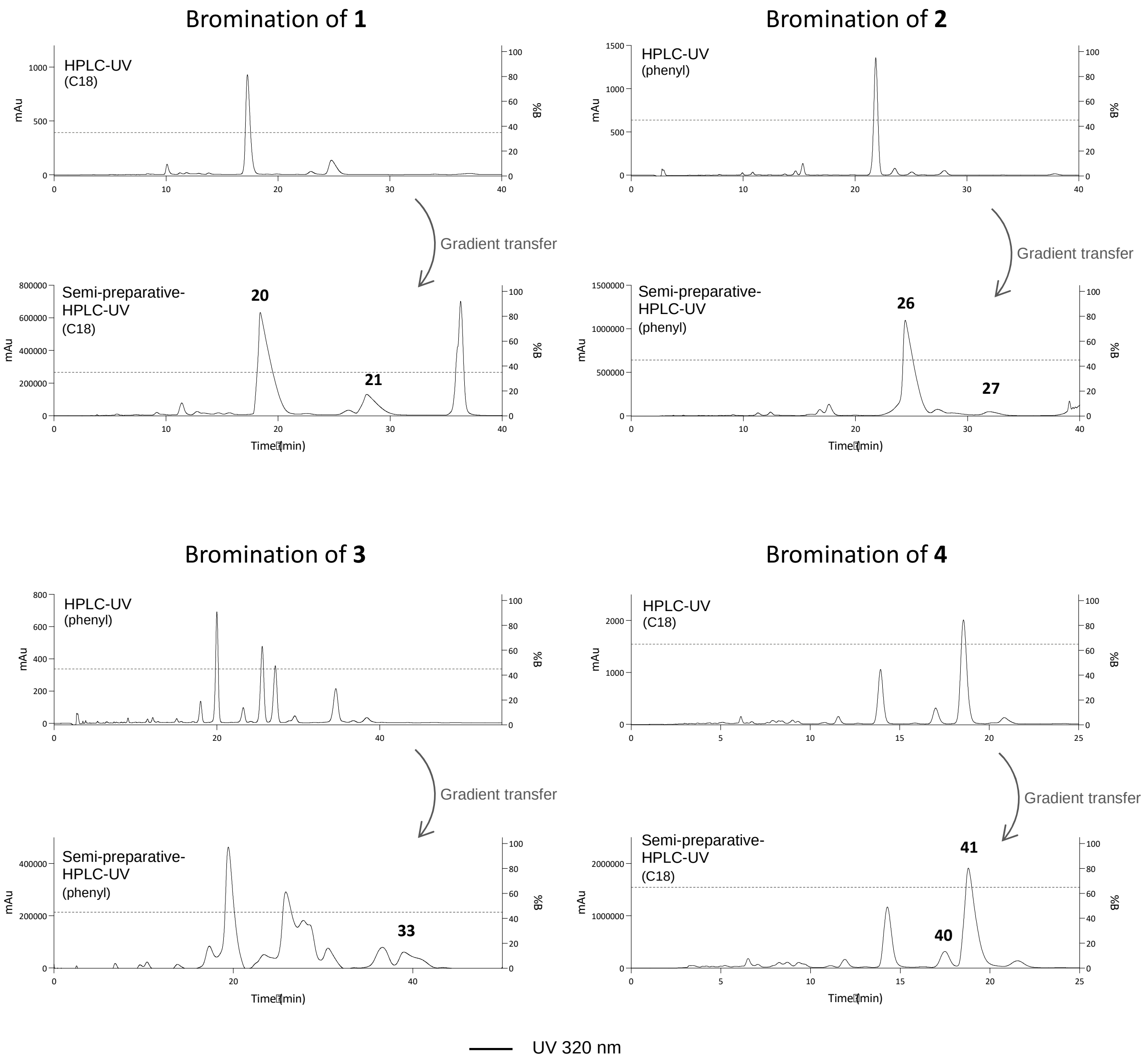

Figure S9. Isolation of brominated derivatives of *trans*-δ-viniferin derivatives 1-4. For each starting compound, the top chromatogram corresponds to HPLC-UV optimization (C_{18} or phenyl, 250 x 4.6 mm i.d., 5 µm) and the bottom chromatogram is the transfer to semi-preparative HPLC-UV (C_{18} or phenyl, ~250 x 10 mm i.d., 5 µm).

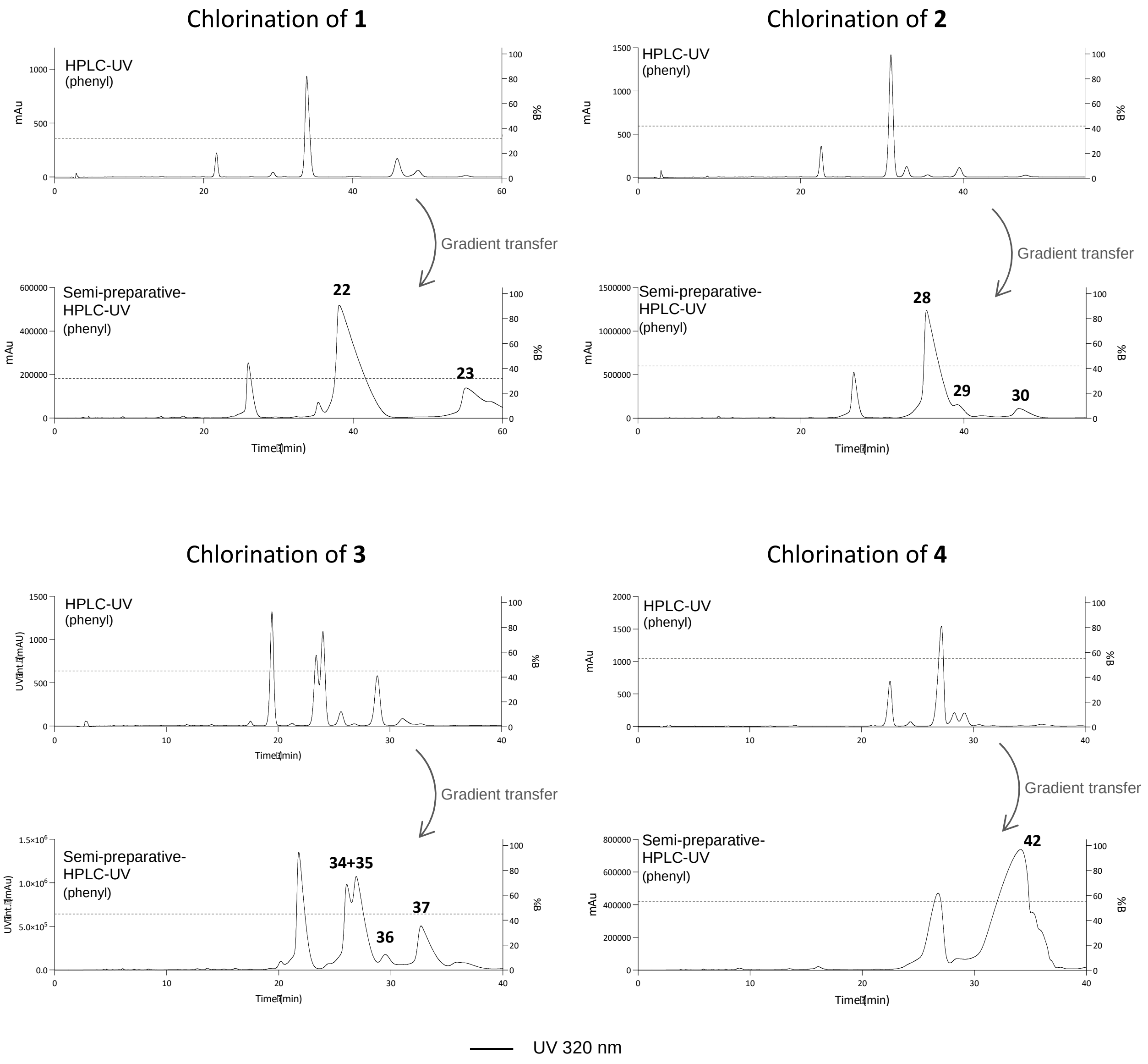

Figure S10. Isolation of chlorinated derivatives of *trans*-δ-viniferin derivatives 1-4. For each starting compound, the top chromatogram corresponds to HPLC-UV optimization (Phenyl 250 x 4.6 mm i.d., 5 µm) and the bottom chromatogram is the transfer to semi-preparative HPLC-UV (Phenyl, 250 x 9.4 mm i.d., 5 µm). Separation of compounds **34** and **35** is shown in Figure 6.

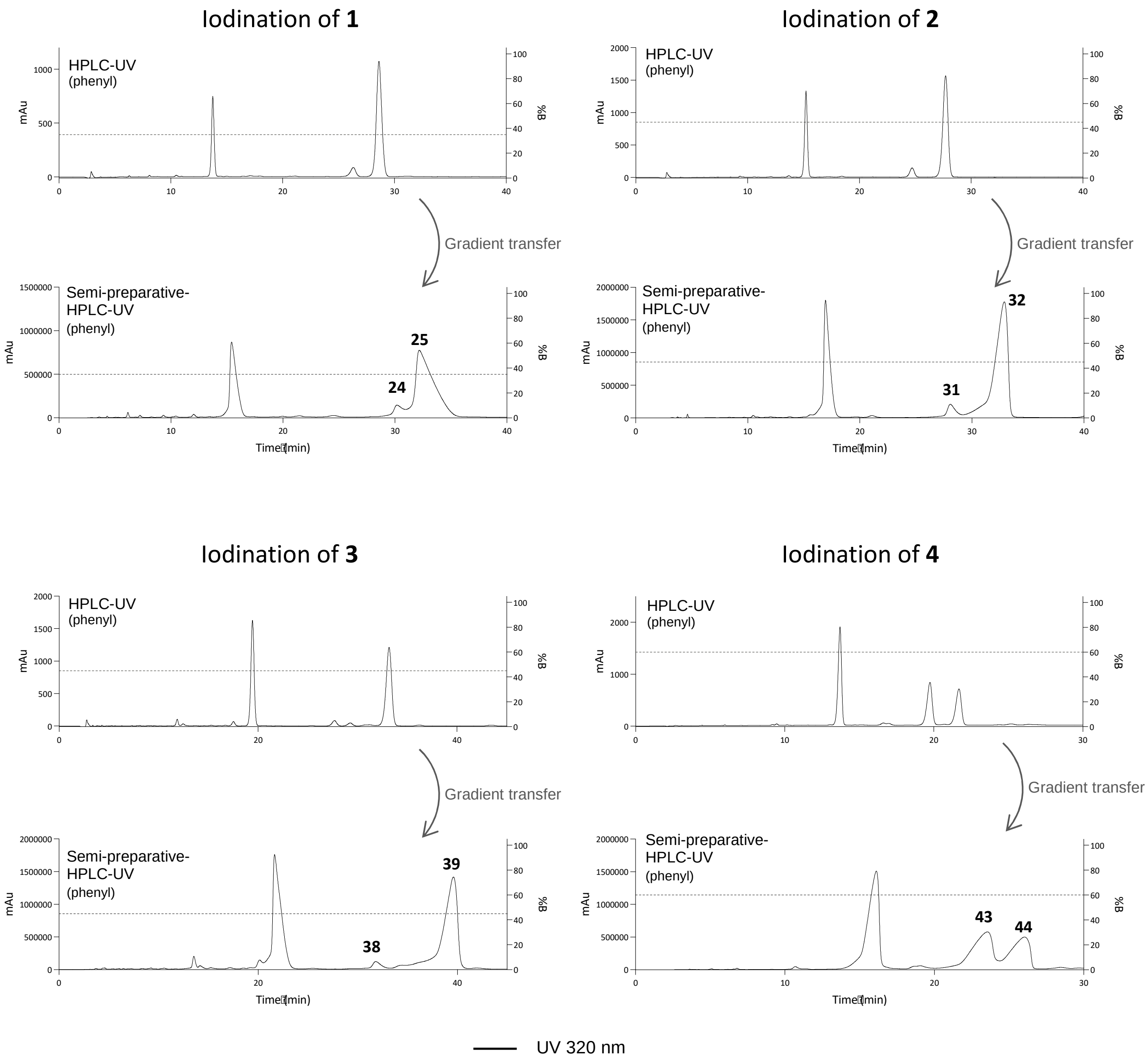

Figure S11. Isolation of iodinated derivatives of *trans*-δ-viniferin derivatives 1-4. For each starting compound, the top chromatogram corresponds to HPLC-UV optimization (Phenyl 250 x 4.6 mm i.d., 5 µm) and the bottom chromatogram is the transfer to semi-preparative HPLC-UV (Phenyl, 250 x 9.4 mm i.d., 5 µm).

Table S1. Details of the linear model.

Variable	Estimated coefficient	Standard error	p-value	Significance
Intercept	2.29	0.10	< 2e-16	***
A OCH_3/CH_3	-1.13	0.22	3.66E-06	***
$R_{6,7}$ OCH_3	1.53	0.35	6.96E-05	***
A Cl/Cl	0.49	0.35	0.174844	
A H/Br	0.69	0.19	0.000688	***
A H/I	0.42	0.22	0.060158	·
R_{10} Cl	0.83	0.35	0.022283	*
A OCH_3/CH_3:C OCH_3/CH_3	2.73	0.25	4.29E-15	***
C OCH_3/CH_3:R_{10} I	0.56	0.26	0.038294	*
A OCH_3/CH_3:R_9 Br	2.31	0.44	2.47E-06	***
A OCH_3/CH_3:R_9 Cl	-0.86	0.34	0.015543	*
A H/Cl:R_9 Cl	0.79	0.29	0.007996	**

The symbols ***, **, * and · correspond to p-values in the following intervals [0, 0.1%), [0.1%, 1%), [1%, 5%) and [5%, 10%), respectively

Table S2. Chromatographic conditions for the separation of the mixtures of *trans*/*cis* isomers of *trans*-δ-viniferin derivatives.

**Mixture**	**UHPLC** (100 x 2.1 mm i.d., 1.7 µm)				**Semi-preparative HPLC** (250 x 19 mm i.d., 5 µm)			
	Flow-rate (mL/min)	%A	%B	Time (min)	Flow-rate (mL/min)	%A	%B	Time (min)
**1** + **5**	0.5	70	30	0	17	70	30	0
		60	40	10		60	40	60
		0	100	10.2		0	100	61
		0	100	11.7		0	100	70
**2** + **6** and **3** + **7**	0.5	60	40	0	17	60	40	0
		50	50	10		50	50	60
		0	100	10.2		0	100	61
		0	100	11.7		0	100	70
**4** + **8**	0.5	45	55	0.0	17	45	55	0
		45	55	10.0		45	55	60
		0	100	10.2		0	100	61
		0	100	11.7		0	100	70

Table S3. Chromatographic conditions (stationary phase, percentage of MeCN) for each separation of halogenation reactions.

Starting material / halogenation reaction	Br	Cl	I
**1**	C_{18}, ISO 35% MeCN	Phenyl, ISO 32% MeCN	Phenyl, ISO 35% MeCN
**2**	Phenyl, ISO 45% MeCN	Phenyl, ISO 42% MeCN	Phenyl, ISO 45% MeCN
**3**	Phenyl, ISO 45% MeCN	Phenyl, ISO 45% MeCN	Phenyl, ISO 45% MeCN
**4**	C_{18}, ISO 65% MeCN	Phenyl, ISO 55% MeCN	Phenyl, ISO 60% MeCN

Table S4. Complete table with every compound generated and associated data (name, SMILE, molecular weight, MIC).

Number	Name	SMILES	MW (g/mol)	Type	MIC *S. aureus* NEWMAN (MSSA) (µM)
1	trans-δ-viniferin	OC1=CC=C([C@H](O2)[C@H](C3=CC(O)=CC(O)=C3)C4=C2C=CC(/C=C/C5=CC(O)=CC(O)=C5)=C4)C=C1	454	Viniferin	16-32
2	11',13'-di-O-methyl-trans-δ-viniferin	OC1=CC=C([C@H](O2)[C@H](C3=CC(OC)=CC(OC)=C3)C4=C2C=CC(/C=C/C5=CC(O)=CC(O)=C5)=C4)C=C1	482	Viniferin	4
3	11,13-di-O-methyl-trans-δ-viniferin	OC(C=C1)=CC=C1[C@@H](O2)[C@@H](C3=CC(O)=CC(O)=C3)C4=C2C=CC(/C=C/C5=CC(OC)=CC(OC)=C5)=C4	482	Viniferin	4
4	11,11',13,13'-tetra-O-methyl-trans-δ-viniferin	OC(C=C1)=CC=C1[C@@H](O2)[C@@H](C3=CC(OC)=CC(OC)=C3)C4=C2C=CC(/C=C/C5=CC(OC)=CC(OC)=C5)=C4	510	Viniferin	>64
5	cis-δ-viniferin	OC(C=C1)=CC=C1[C@@H](O2)[C@@H](C3=CC(O)=CC(O)=C3)C4=C2C=CC(/C=C\C5=CC(O)=CC(O)=C5)=C4	454	*cis* form	32-64
6	11',13'-di-O-methyl-cis-δ-viniferin	OC(C=C1)=CC=C1[C@@H](O2)[C@@H](C3=CC(OC)=CC(OC)=C3)C4=C2C=CC(/C=C\C5=CC(O)=CC(O)=C5)=C4	482	*cis* form	8-16
7	11,13-di-O-methyl-cis-δ-viniferin	OC(C=C1)=CC=C1[C@@H](O2)[C@@H](C3=CC(O)=CC(O)=C3)C4=C2C=CC(/C=C\C5=CC(OC)=CC(OC)=C5)=C4	482	*cis* form	8-16
8	11,11',13,13'-tetra-O-methyl-cis-δ-viniferin	OC(C=C1)=CC=C1[C@@H](O2)[C@@H](C3=CC(OC)=CC(OC)=C3)C4=C2C=CC(/C=C\C5=CC(OC)=CC(OC)=C5)=C4	510	*cis* form	>64
9	11'-O-methyl-trans-δ-viniferin	OC(C=C1)=CC=C1[C@@H](O2)[C@@H](C3=CC(O)=CC(OC)=C3)C4=C2C=CC(/C=C/C5=CC(O)=CC(O)=C5)=C4	468	*O*-methyl derivative	8-16
10	11-O-methyl-trans-δ-viniferin	OC(C=C1)=CC=C1[C@@H](O2)[C@@H](C3=CC(O)=CC(O)=C3)C4=C2C=CC(/C=C/C5=CC(O)=CC(OC)=C5)=C4	468	*O*-methyl derivative	8-16
11	4'-O-methyl-trans-δ-viniferin	OC1=CC([C@H]([C@@H](C2=CC=C(OC)C=C2)O3)C4=C3C=CC(/C=C/C5=CC(O)=CC(O)=C5)=C4)=CC(O)=C1	468	*O*-methyl derivative	8
12	11,11'-di-O-methyl-trans-δ-viniferin	OC(C=C1)=CC=C1[C@@H](O2)[C@@H](C3=CC(O)=CC(OC)=C3)C4=C2C=CC(/C=C/C5=CC(OC)=CC(O)=C5)=C4	482	*O*-methyl derivative	8
13	4',11'-di-O-methyl-trans-δ-viniferin	OC1=CC(OC)=CC([C@H]([C@@H](C2=CC=C(OC)C=C2)O3)C4=C3C=CC(/C=C/C5=CC(O)=CC(O)=C5)=C4)=C1	482	*O*-methyl derivative	8
14	4',11-di-O-methyl-trans-δ-viniferin	OC1=CC([C@H]([C@@H](C2=CC=C(OC)C=C2)O3)C4=C3C=CC(/C=C/C5=CC(OC)=CC(O)=C5)=C4)=CC(O)=C1	482	*O*-methyl derivative	8
15	5,5'-dimethoxy-trans-δ-viniferin	OC(C=C1)=C(OC)C=C1[C@@H](O2)[C@@H](C3=CC(O)=CC(O)=C3)C4=C2C(OC)=CC(/C=C/C5=CC(O)=CC(O)=C5)=C4	514	Other dimer	32-64
16	dehydro-trans-δ-viniferin	OC1=CC=C(C(O2)=C(C3=CC(O)=CC(O)=C3)C4=C2C=CC(/C=C/C5=CC(O)=CC(O)=C5)=C4)C=C1	452	Benzofuran derivative	8
17	11',13'-di-O-methyl-dehydro-trans-δ-viniferin	OC1=CC=C(C(O2)=C(C3=CC(OC)=CC(OC)=C3)C4=C2C=CC(/C=C/C5=CC(O)=CC(O)=C5)=C4)C=C1	480	Benzofuran derivative	4-8
18	11,13-di-O-methyl-dehydro-trans-δ-viniferin	OC1=CC=C(C(O2)=C(C3=CC(O)=CC(O)=C3)C4=C2C=CC(/C=C/C5=CC(OC)=CC(OC)=C5)=C4)C=C1	480	Benzofuran derivative	8
19	11,11',13,13'-tetra-O-methyl-dehydro-trans-δ-viniferin	OC1=CC=C(C(O2)=C(C3=CC(OC)=CC(OC)=C3)C4=C2C=CC(/C=C/C5=CC(OC)=CC(OC)=C5)=C4)C=C1	508	Benzofuran derivative	>64
20	14-bromo-trans-δ-viniferin	OC1=CC=C([C@H](O2)[C@H](C3=CC(O)=CC(O)=C3)C4=C2C=CC(/C=C/C5=C(Br)C(O)=CC(O)=C5)=C4)C=C1	533	Halogenated derivative	16-32

21	14,14'-dibromo-trans-δ-viniferin	OC1=CC=C([C@H](O2)[C@H](C3=C(Br)C(O)=CC(O)=C3)C4=C2C=CC(/C=C/C5=C(Br)C(O)=CC(O)=C5)=C4)C=C1	612	Halogenated derivative	16-32
22	14-chloro-trans-δ-viniferin	OC1=CC=C([C@H](O2)[C@H](C3=CC(O)=CC(O)=C3)C4=C2C=CC(/C=C/C5=C(Cl)C(O)=CC(O)=C5)=C4)C=C1	488	Halogenated derivative	16-32
23	14,14'-dichloro-trans-δ-viniferin	OC1=CC=C([C@H](O2)[C@H](C3=C(Cl)C(O)=CC(O)=C3)C4=C2C=CC(/C=C/C5=C(Cl)C(O)=CC(O)=C5)=C4)C=C1	523	Halogenated derivative	16-32
24	14-iodo-trans-δ-viniferin	OC1=CC=C([C@H](O2)[C@H](C3=CC(O)=CC(O)=C3)C4=C2C=CC(/C=C/C5=C(I)C(O)=CC(O)=C5)=C4)C=C1	580	Halogenated derivative	16-32
25	12-iodo-trans-δ-viniferin	OC1=CC=C([C@H](O2)[C@H](C3=CC(O)=CC(O)=C3)C4=C2C=CC(/C=C/C5=CC(O)=C(I)C(O)=C5)=C4)C=C1	580	Halogenated derivative	4-8
26	14-bromo-11',13'-di-O-methyl-trans-δ-viniferin	OC1=CC=C([C@H](O2)[C@H](C3=CC(OC)=CC(OC)=C3)C4=C2C=CC(/C=C/C5=C(Br)C(O)=CC(O)=C5)=C4)C=C1	561	Halogenated derivative	16-32
27	10,14-dibromo-11',13'-di-O-methyl-trans-δ-viniferin	OC1=CC=C([C@H](O2)[C@H](C3=CC(OC)=CC(OC)=C3)C4=C2C=CC(/C=C/C5=C(Br)C(O)=CC(O)=C5Br)=C4)C=C1	640	Halogenated derivative	NA
28	14-chloro-11',13'-di-O-methyl-trans-δ-viniferin	OC1=CC=C([C@H](O2)[C@H](C3=CC(OC)=CC(OC)=C3)C4=C2C=CC(/C=C/C5=C(Cl)C(O)=CC(O)=C5)=C4)C=C1	516	Halogenated derivative	4-8
29	12-chloro-11',13'-di-O-methyl-trans-δ-viniferin	OC1=CC=C([C@H](O2)[C@H](C3=CC(OC)=CC(OC)=C3)C4=C2C=CC(/C=C/C5=CC(O)=C(Cl)C(O)=C5)=C4)C=C1	516	Halogenated derivative	16-32
30	10,14-dichloro-11',13'-di-O-methyl-trans-δ-viniferin	OC1=CC=C([C@H](O2)[C@H](C3=CC(OC)=CC(OC)=C3)C4=C2C=CC(/C=C/C5=C(Cl)C(O)=CC(O)=C5Cl)=C4)C=C1	551	Halogenated derivative	16
31	14-iodo-11',13'-di-O-methyl-trans-δ-viniferin	OC1=CC=C([C@H](O2)[C@H](C3=CC(OC)=CC(OC)=C3)C4=C2C=CC(/C=C/C5=C(I)C(O)=CC(O)=C5)=C4)C=C1	608	Halogenated derivative	8-16
32	12-iodo-11',13'-di-O-methyl-trans-δ-viniferin	OC1=CC=C([C@H](O2)[C@H](C3=CC(OC)=CC(OC)=C3)C4=C2C=CC(/C=C/C5=CC(O)=C(I)C(O)=C5)=C4)C=C1	608	Halogenated derivative	16-32
33	14,14'-dibromo-11,13-di-O-methyl-trans-δ-viniferin	OC(C=C1)=CC=C1[C@@H](O2)[C@@H](C3=C(Br)C(O)=CC(O)=C3)C4=C2C=CC(/C=C/C5=C(Br)C(OC)=CC(OC)=C5)=C4	640	Halogenated derivative	>64
34	14'-chloro-11,13-di-O-methyl-trans-δ-viniferin	OC(C=C1)=CC=C1[C@@H](O2)[C@@H](C3=C(Cl)C(O)=CC(O)=C3)C4=C2C=CC(/C=C/C5=CC(OC)=CC(OC)=C5)=C4	516	Halogenated derivative	1-2
35	14-chloro-11,13-di-O-methyl-trans-δ-viniferin	OC(C=C1)=CC=C1[C@@H](O2)[C@@H](C3=CC(O)=CC(O)=C3)C4=C2C=CC(/C=C/C5=C(Cl)C(OC)=CC(OC)=C5)=C4	516	Halogenated derivative	2
36	12-chloro-11,13-di-O-methyl-trans-δ-viniferin	OC(C=C1)=CC=C1[C@@H](O2)[C@@H](C3=CC(O)=CC(O)=C3)C4=C2C=CC(/C=C/C5=CC(OC)=C(Cl)C(OC)=C5)=C4	516	Halogenated derivative	NA
37	14,14'-dichloro-11,13-di-O-methyl-trans-δ-viniferin	OC(C=C1)=CC=C1[C@@H](O2)[C@@H](C3=C(Cl)C(O)=CC(O)=C3)C4=C2C=CC(/C=C/C5=C(Cl)C(OC)=CC(OC)=C5)=C4	551	Halogenated derivative	2-4
38	14'-iodo-11,13-di-O-methyl-trans-δ-viniferin	OC(C=C1)=CC=C1[C@@H](O2)[C@@H](C3=C(I)C(O)=CC(O)=C3)C4=C2C=CC(/C=C/C5=CC(OC)=CC(OC)=C5)=C4	608	Halogenated derivative	NA
39	12'-iodo-11,13-di-O-methyl-trans-δ-viniferin	OC(C=C1)=CC=C1[C@@H](O2)[C@@H](C3=CC(O)=C(I)C(O)=C3)C4=C2C=CC(/C=C/C5=CC(OC)=CC(OC)=C5)=C4	608	Halogenated derivative	4
40	12-bromo-11,11',13,13'-tetra-O-methyl-trans-δ-viniferin	OC(C=C1)=CC=C1[C@@H](O2)[C@@H](C3=CC(OC)=CC(OC)=C3)C4=C2C=CC(/C=C/C5=CC(OC)=C(Br)C(OC)=C5)=C4	589	Halogenated derivative	>64
41	14-bromo-11,11',13,13'-tetra-O-methyl-trans-δ-viniferin	OC(C=C1)=CC=C1[C@@H](O2)[C@@H](C3=CC(OC)=CC(OC)=C3)C4=C2C=CC(/C=C/C5=C(Br)C(OC)=CC(OC)=C5)=C4	589	Halogenated derivative	>64
42	14-chloro-11,11',13,13'-tetra-O-methyl-trans-δ-viniferin	OC(C=C1)=CC=C1[C@@H](O2)[C@@H](C3=CC(OC)=CC(OC)=C3)C4=C2C=CC(/C=C/C5=C(Cl)C(OC)=CC(OC)=C5)=C4	545	Halogenated derivative	>64
43	14-iodo-11,11',13,13'-tetra-O-methyl-trans-δ-viniferin	OC(C=C1)=CC=C1[C@@H](O2)[C@@H](C3=CC(OC)=CC(OC)=C3)C4=C2C=CC(/C=C/C5=C(I)C(OC)=CC(OC)=C5)=C4	636	Halogenated derivative	>64
44	12-iodo-11,11',13,13'-tetra-O-methyl-trans-δ-viniferin	OC(C=C1)=CC=C1[C@@H](O2)[C@@H](C3=CC(OC)=CC(OC)=C3)C4=C2C=CC(/C=C/C5=CC(OC)=C(I)C(OC)=C5)=C4	636	Halogenated derivative	>64

NMR Data

Pages 25 to 126: ^{1}H, COSY, ^{13}C, HSQC, HMBC and ROESY spectra of compounds **5** to **44**

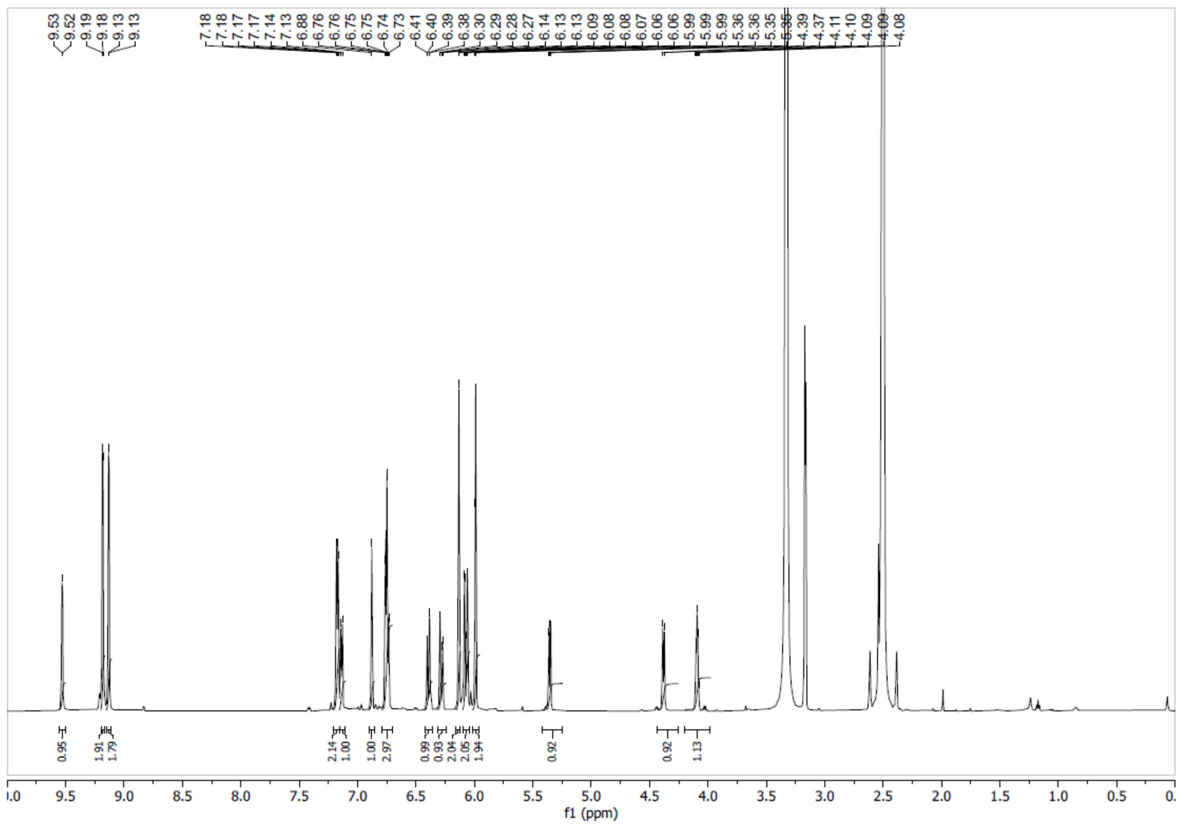

^{1}H NMR spectrum of compound **5** in DMSO-d_6 at 600 MHz

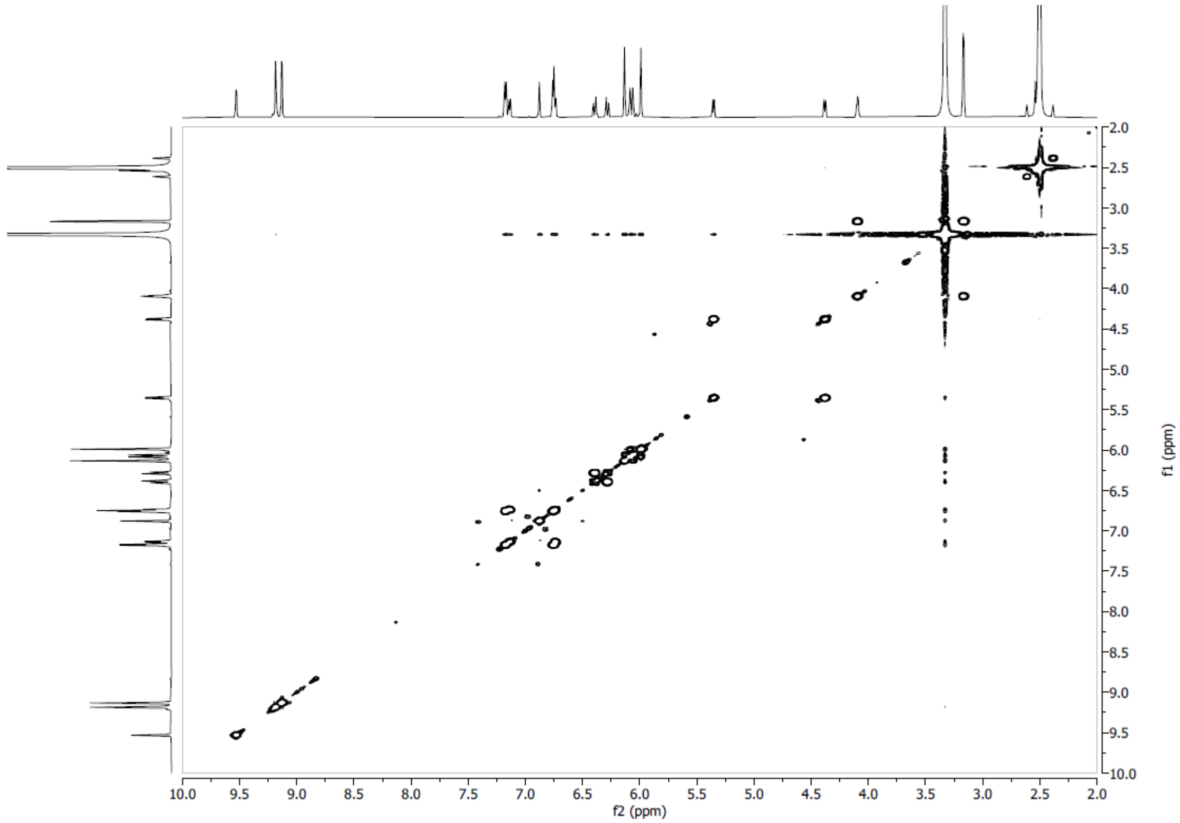

COSY NMR spectrum of compound **5** in DMSO-d_6

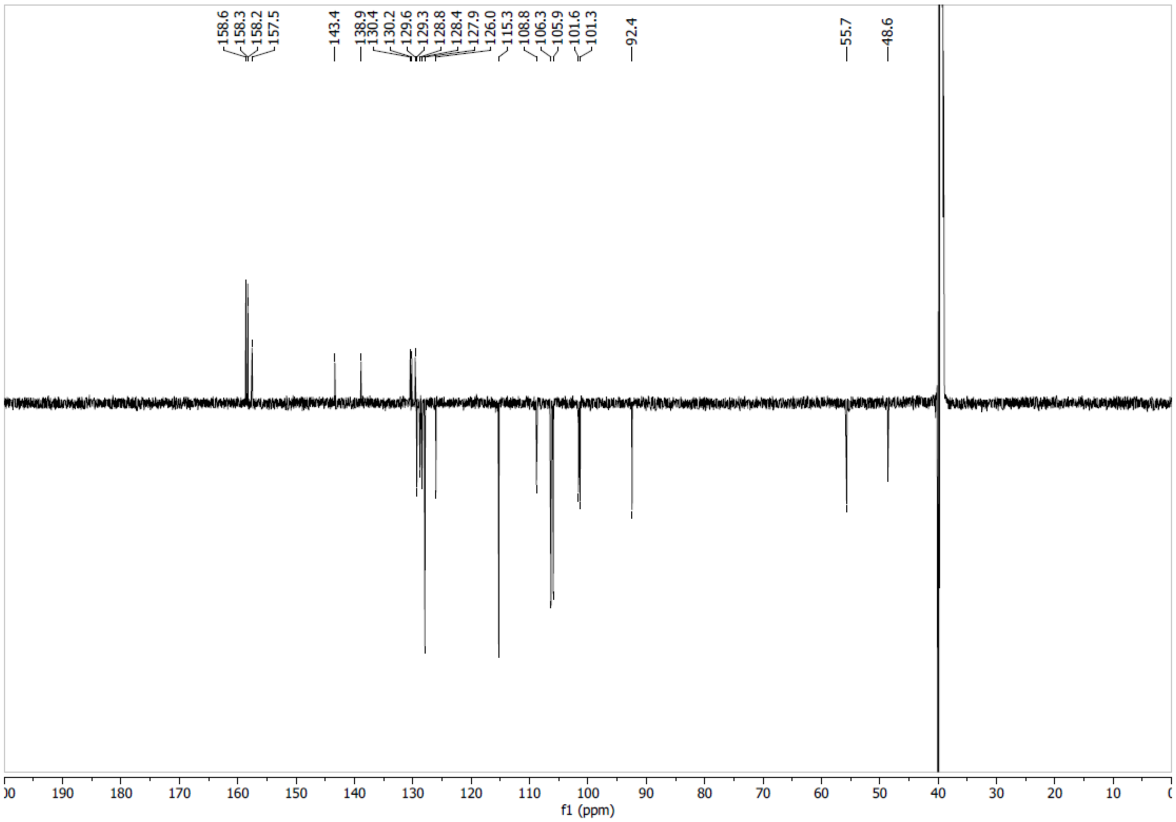

^{13}C-DEPTQ NMR spectrum of compound **5** in DMSO-d_6 at 151 MHz

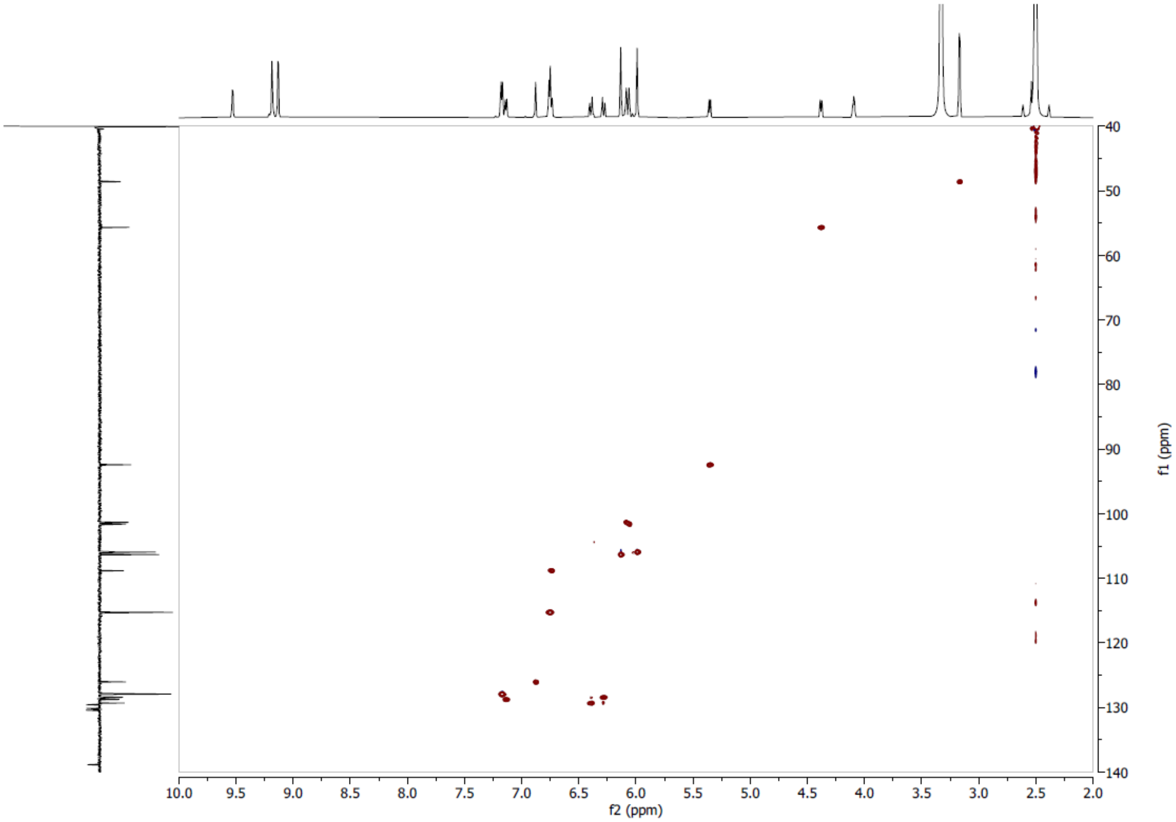

Edited HSQC NMR spectrum of compound **5** in DMSO-d_6

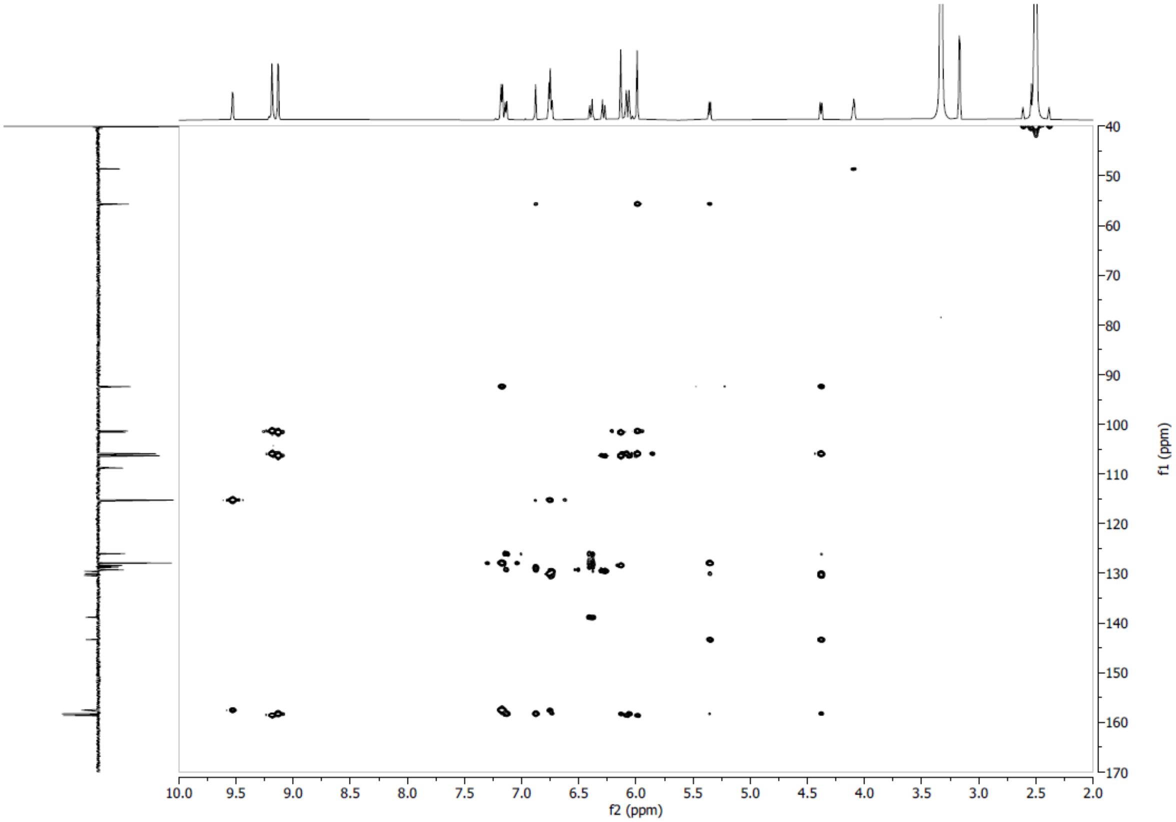

HMBC NMR spectrum of compound **5** in DMSO-d_6

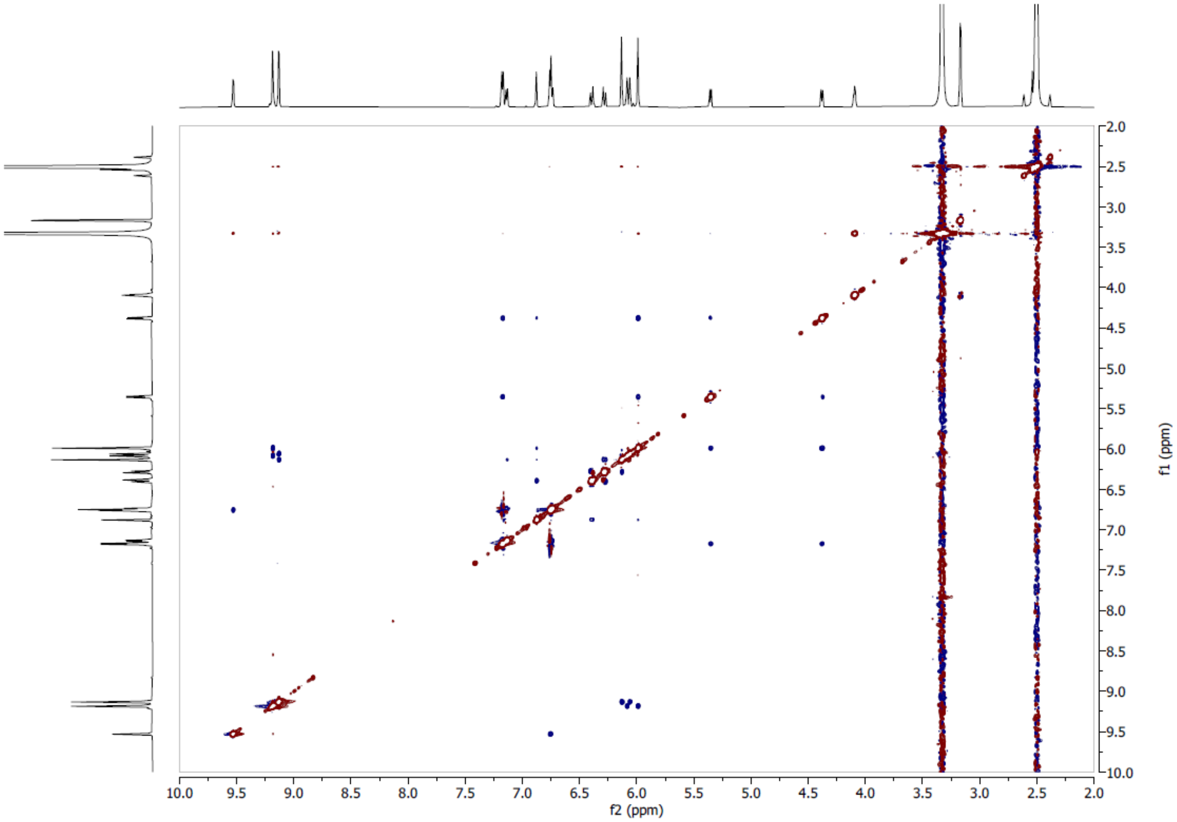

ROESY NMR spectrum of compound **5** in DMSO-d_6

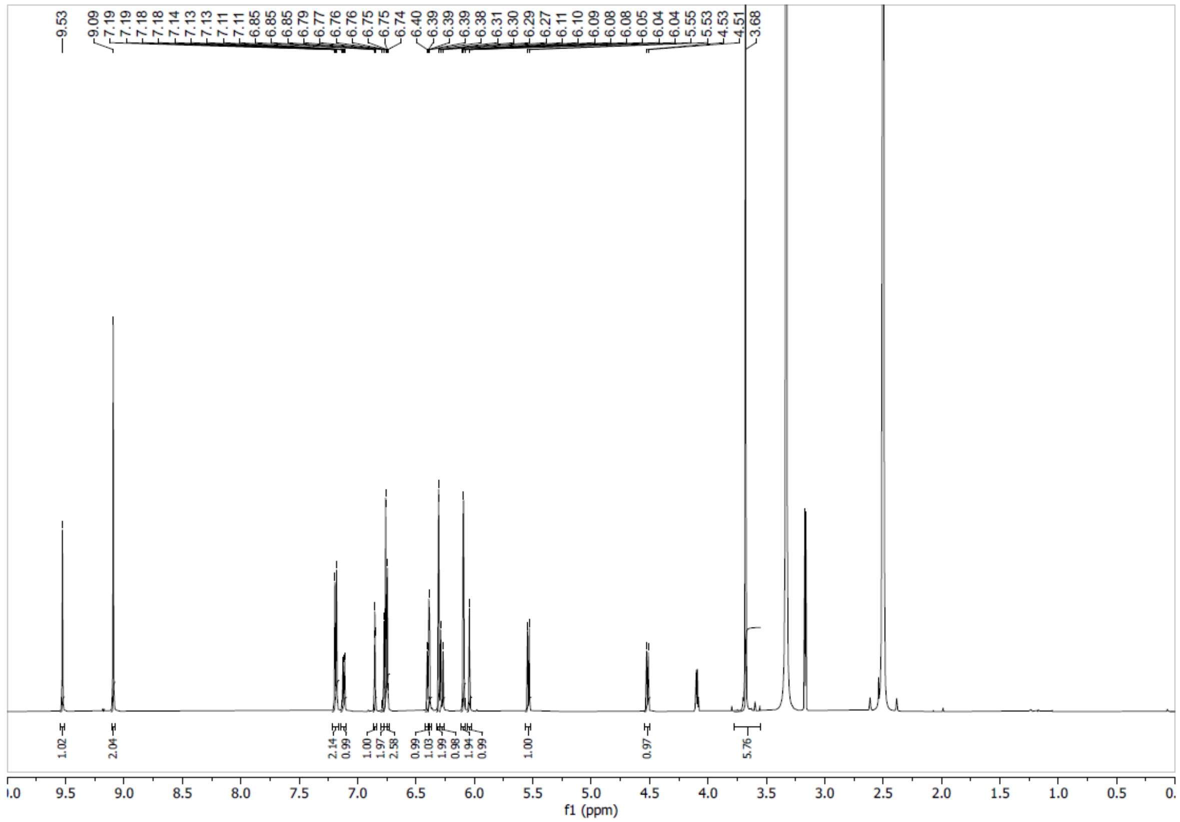

^{1}H NMR spectrum of compound **6** in DMSO-d_6 at 600 MHz

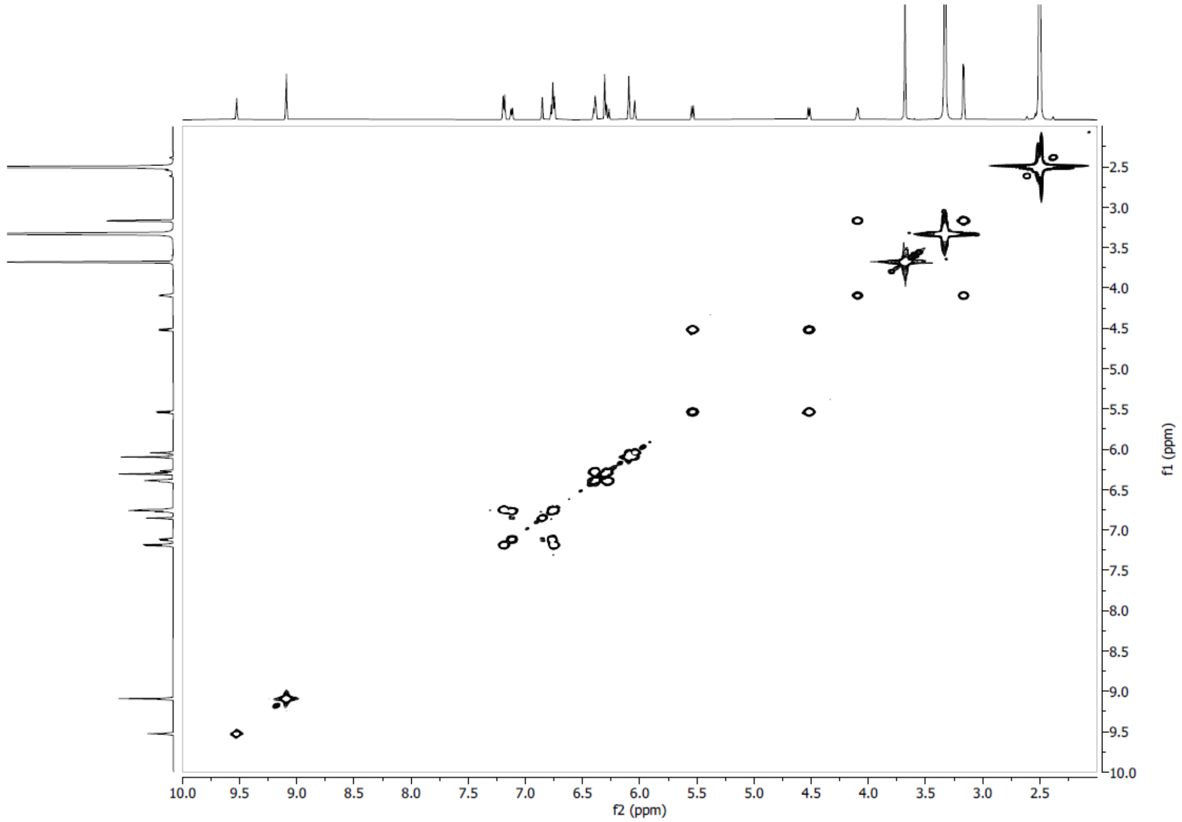

COSY NMR spectrum of compound **6** in DMSO-d_6

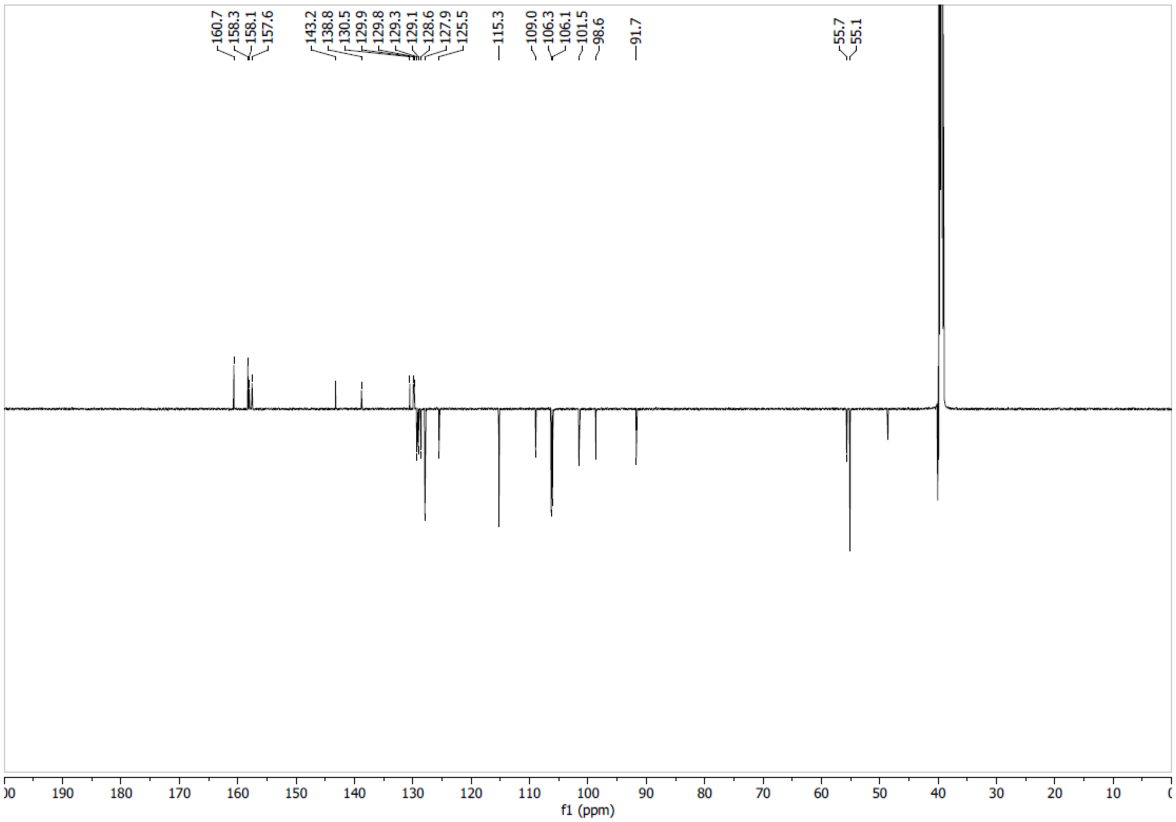

^{13}C-DEPTQ NMR spectrum of compound **6** in DMSO-d_6 at 151 MHz

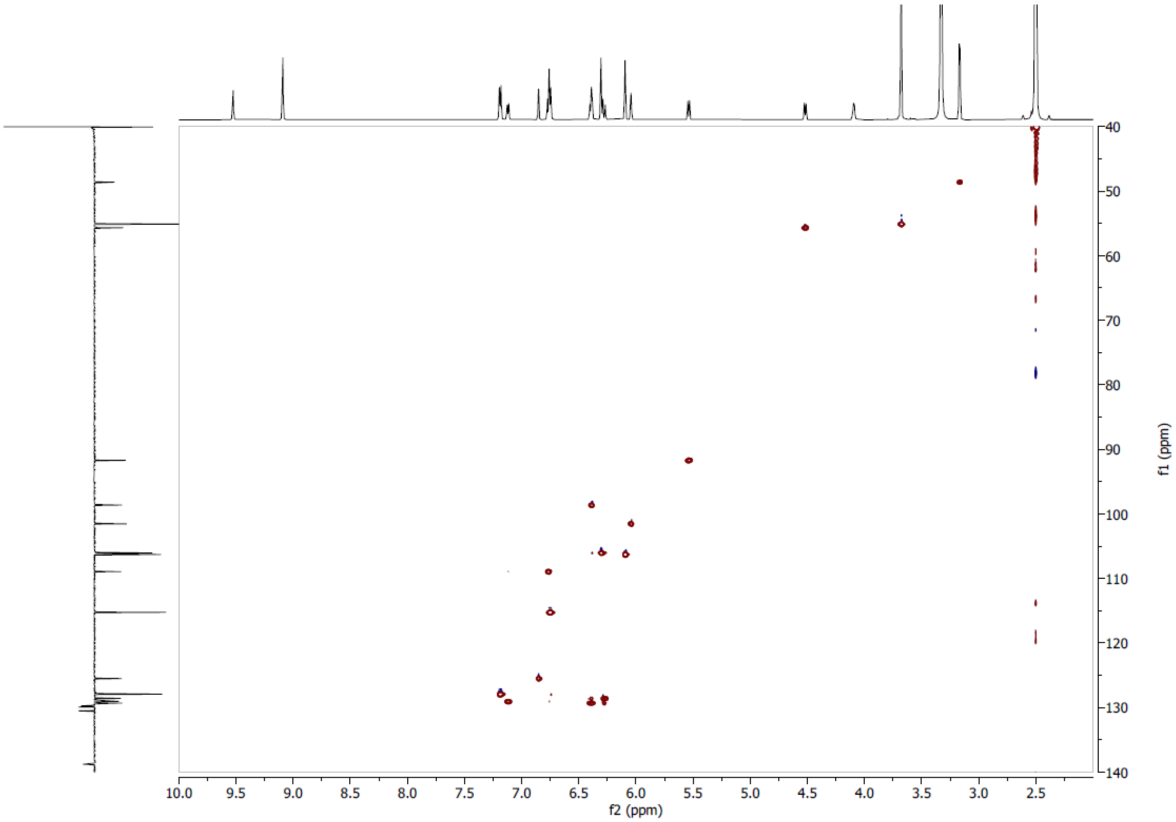

Edited HSQC NMR spectrum of compound **6** in DMSO-d_6

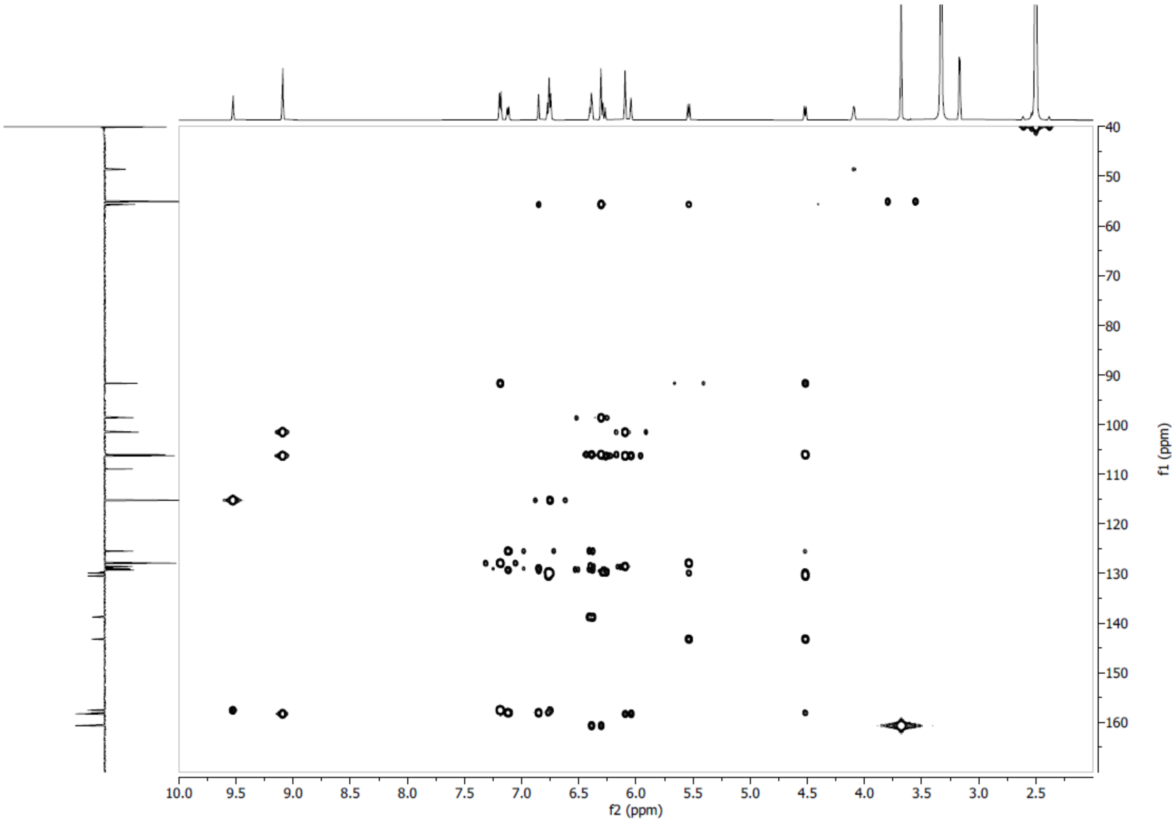

HMBC NMR spectrum of compound **6** in DMSO-d_6

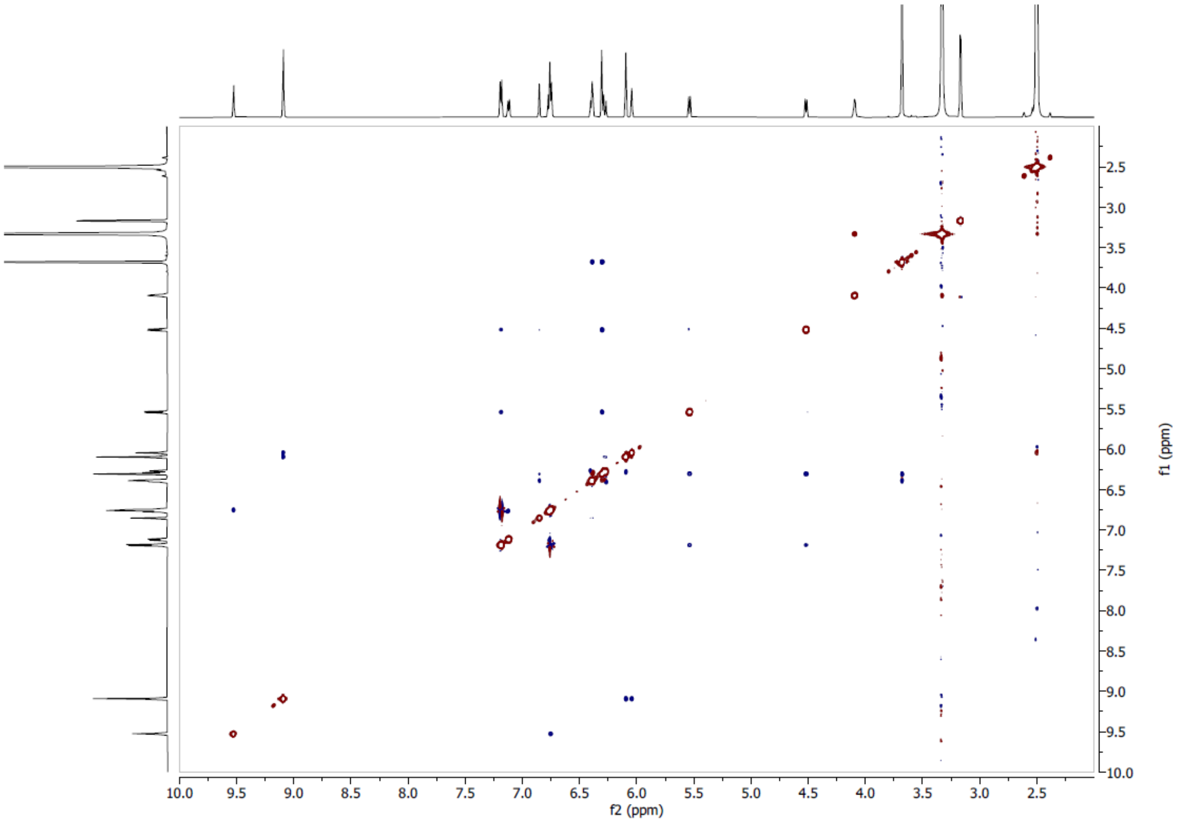

ROESY NMR spectrum of compound **6** in DMSO-d_6

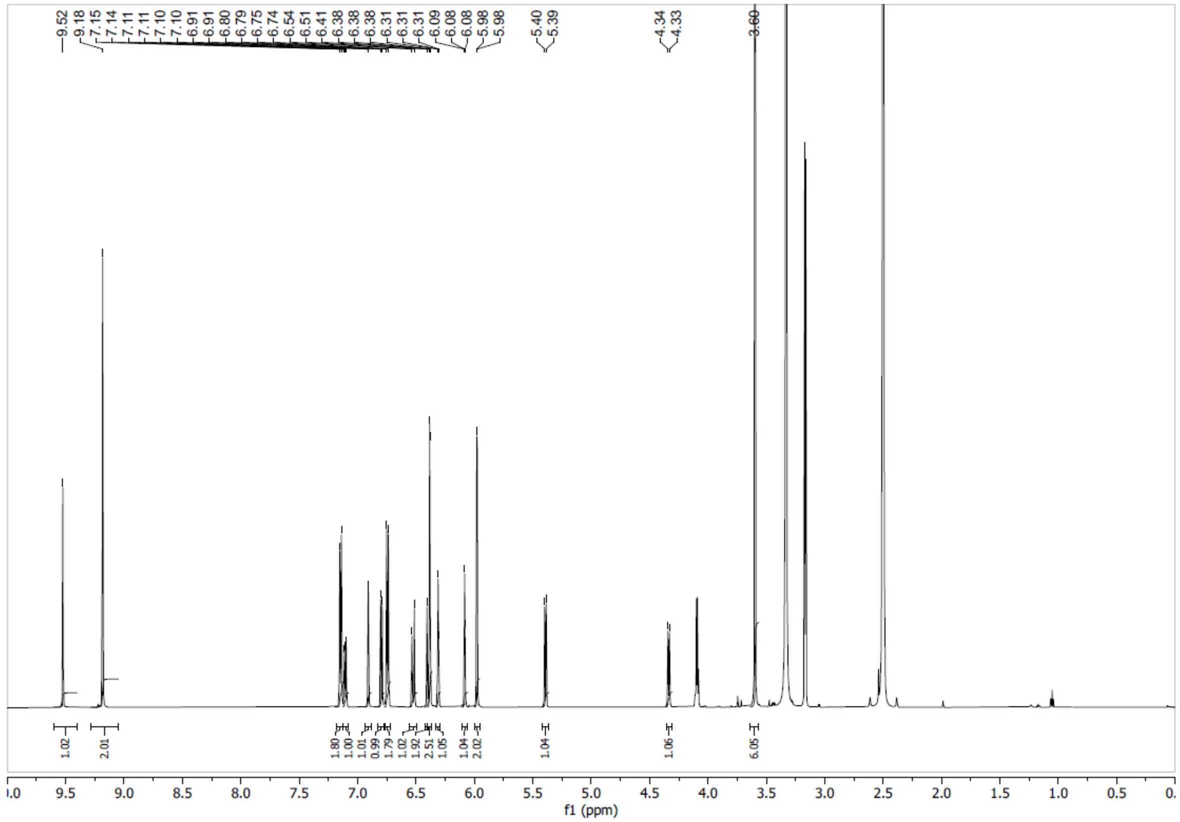

^{1}H NMR spectrum of compound **7** in DMSO-d_6 at 600 MHz

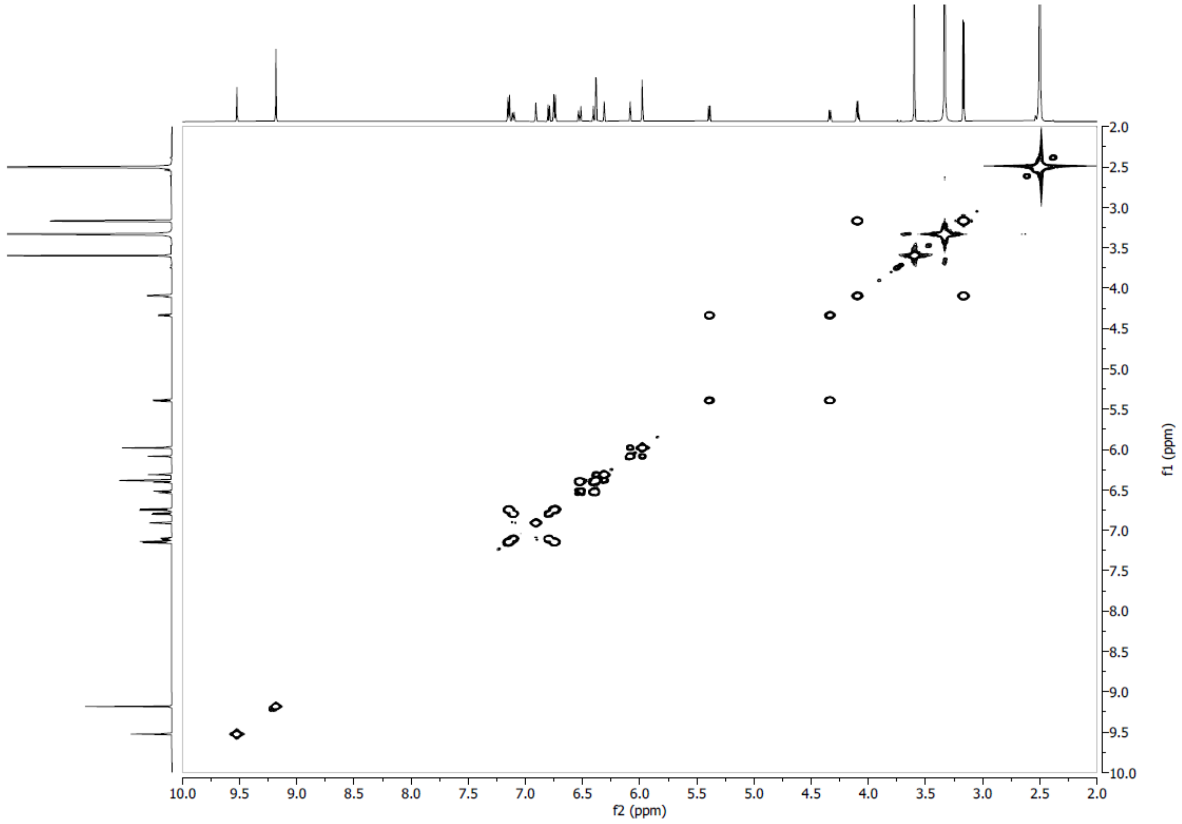

COSY NMR spectrum of compound **7** in DMSO-d_6

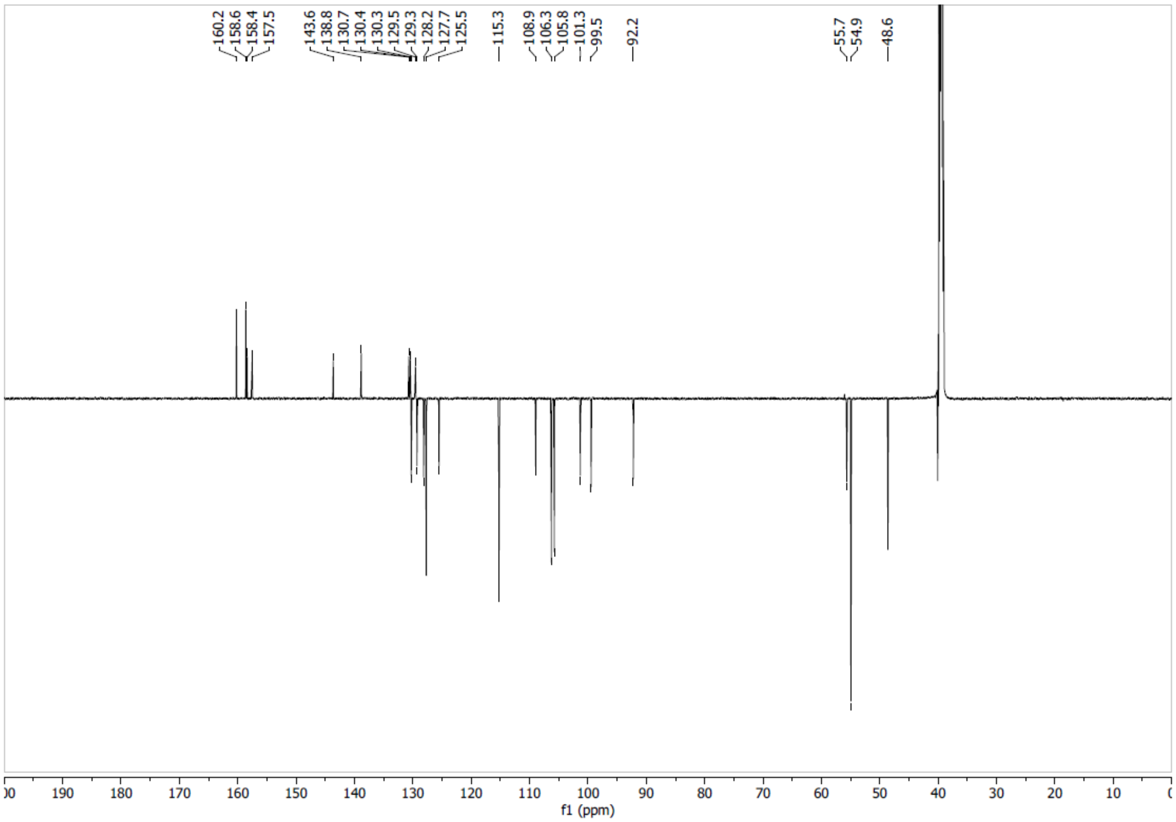

^{13}C-DEPTQ NMR spectrum of compound **7** in DMSO-d_6 at 151 MHz

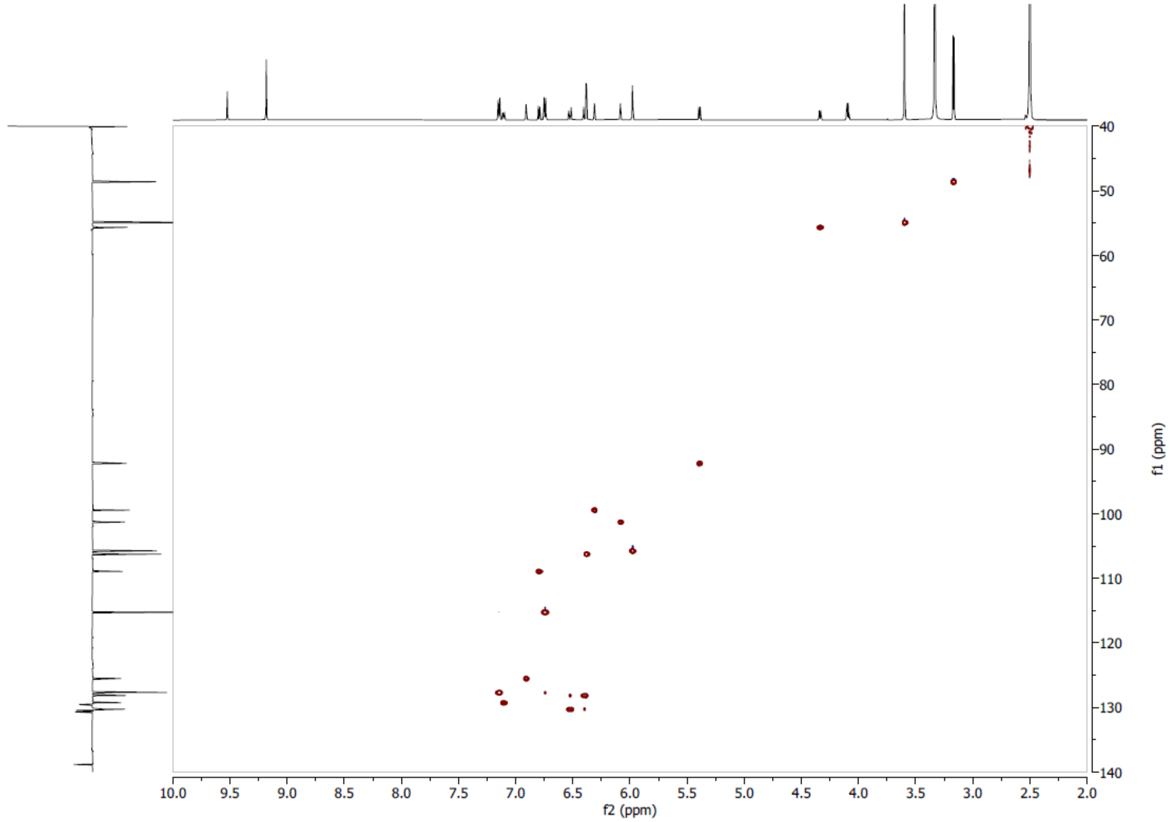

Edited HSQC NMR spectrum of compound **7** in DMSO-d_6

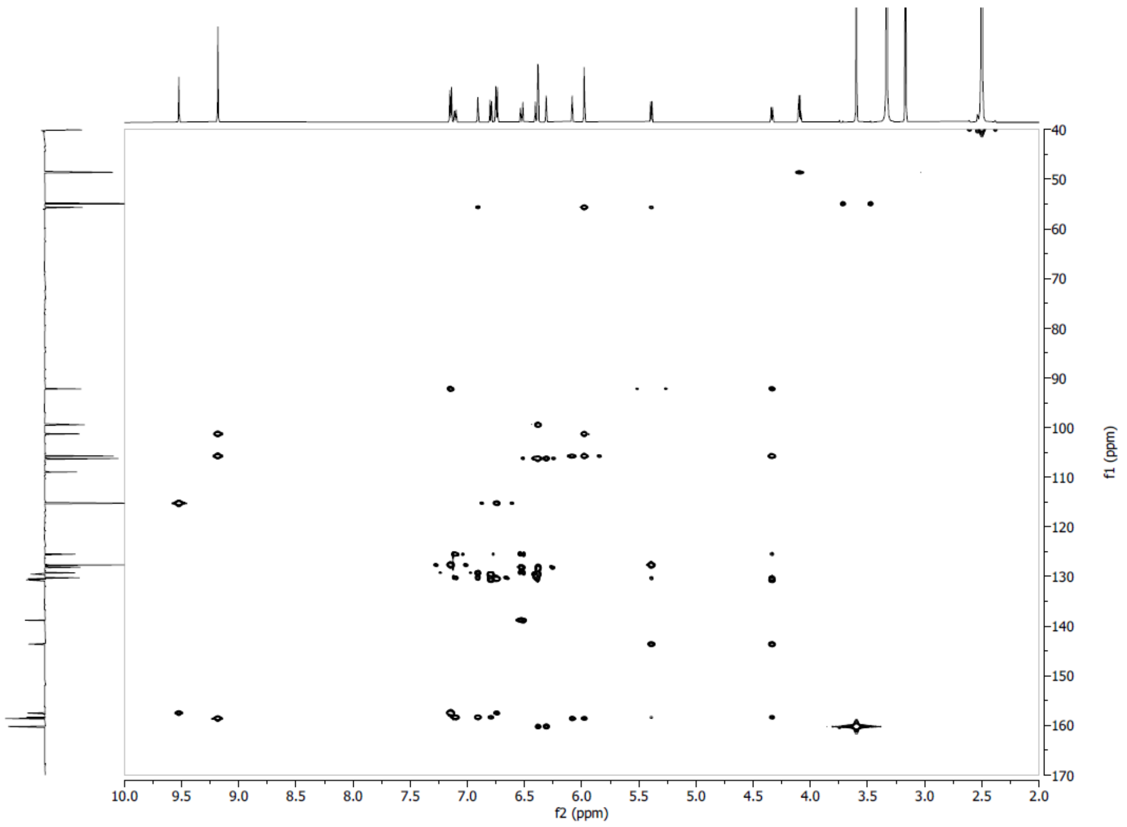

HMBC NMR spectrum of compound **7** in DMSO-*d*6

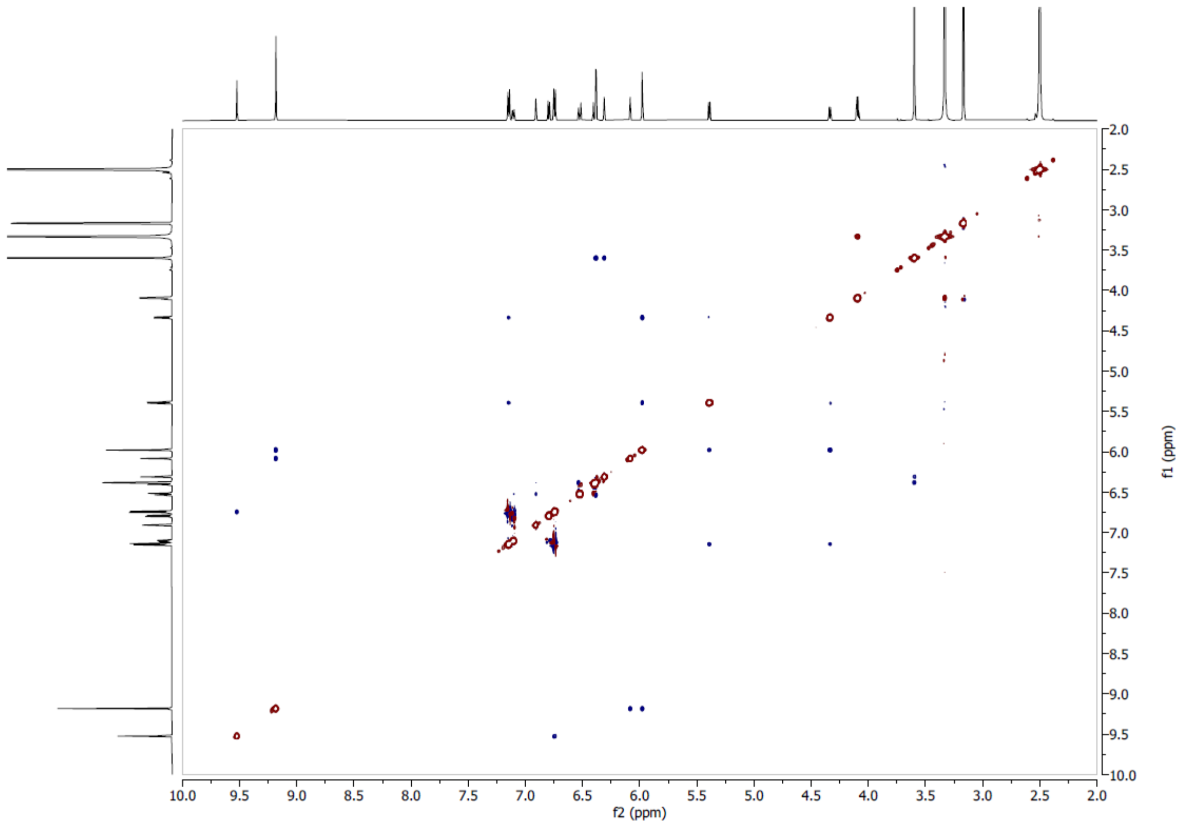

ROESY NMR spectrum of compound **7** in DMSO-*d*6

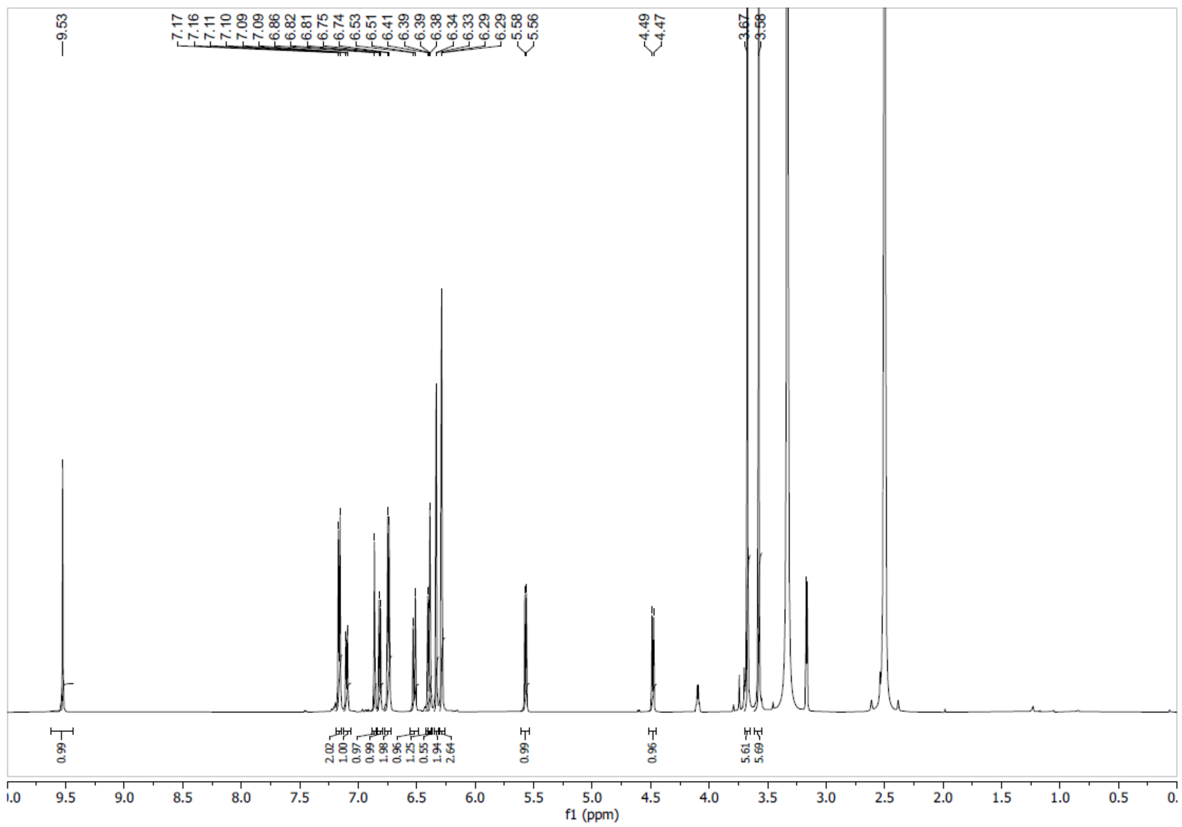

^{1}H NMR spectrum of compound **8** in DMSO-d_6 at 600 MHz

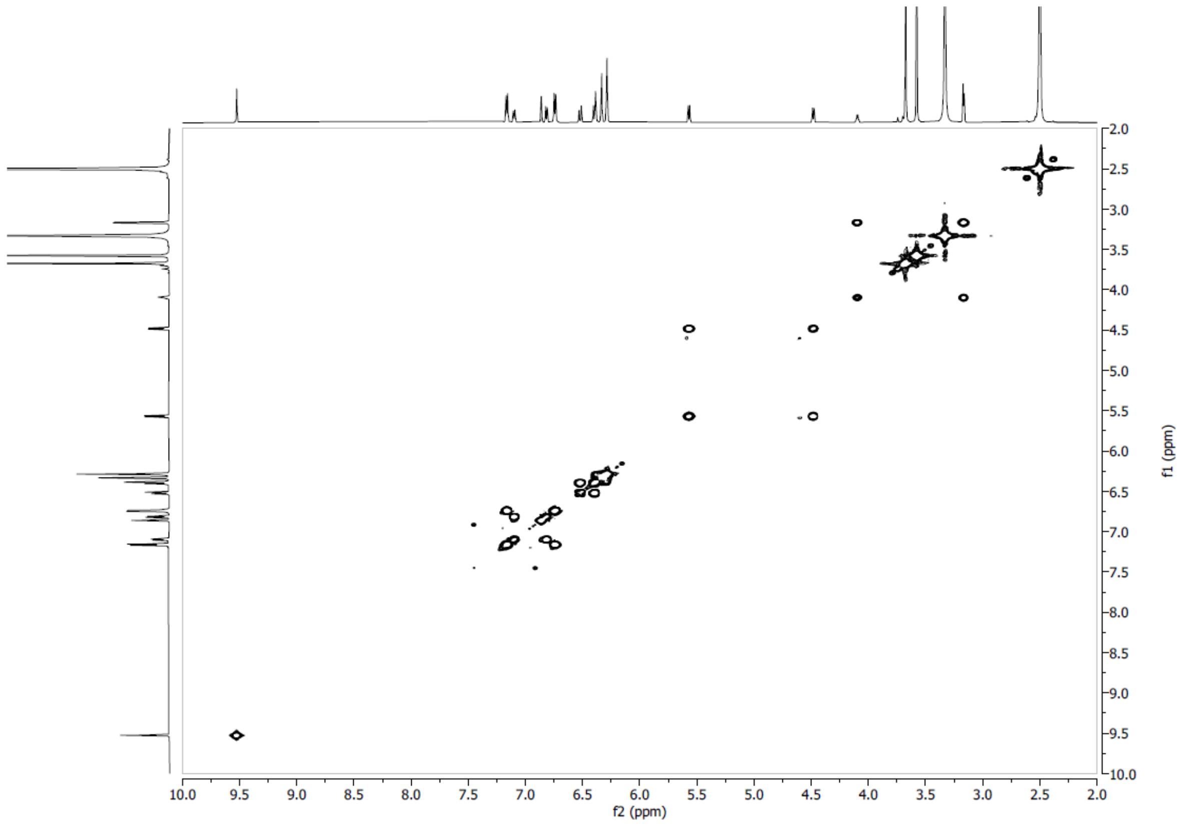

COSY NMR spectrum of compound **8** in DMSO-d_6

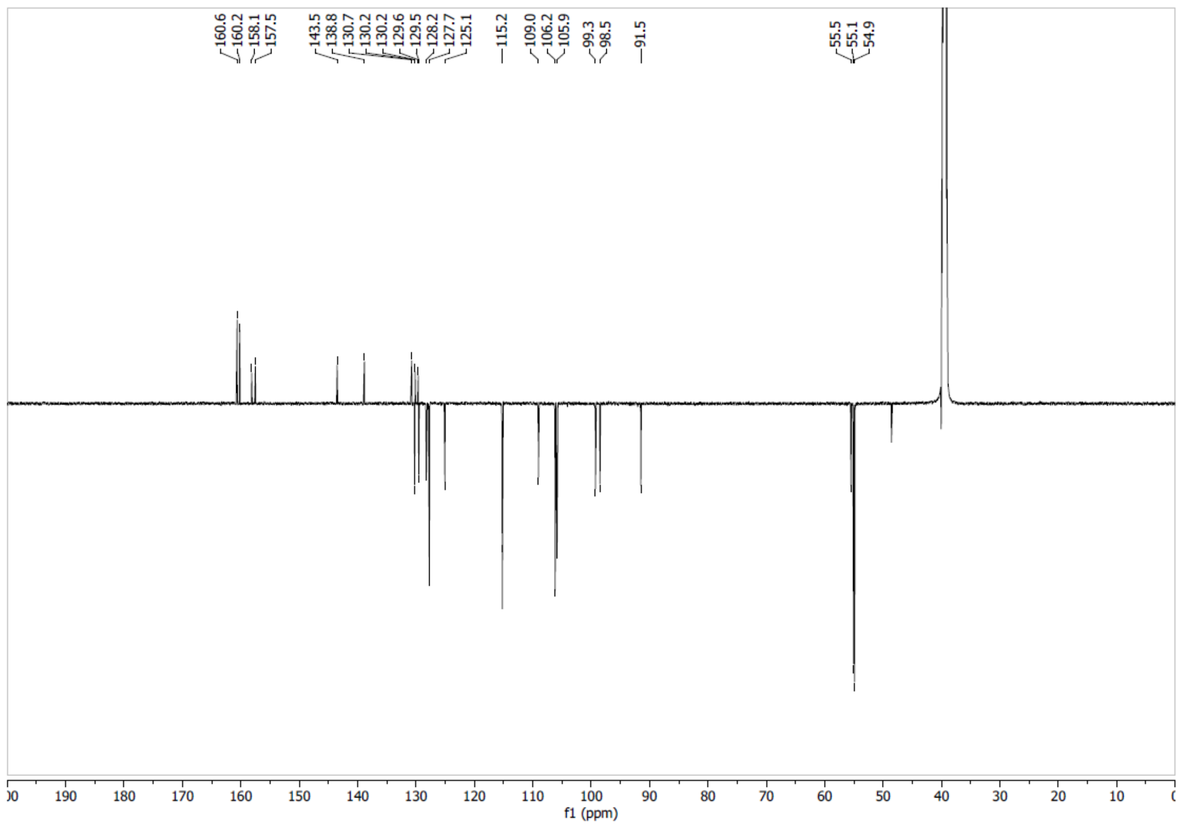

^{13}C-DEPTQ NMR spectrum of compound **8** in DMSO-d_6 at 151 MHz

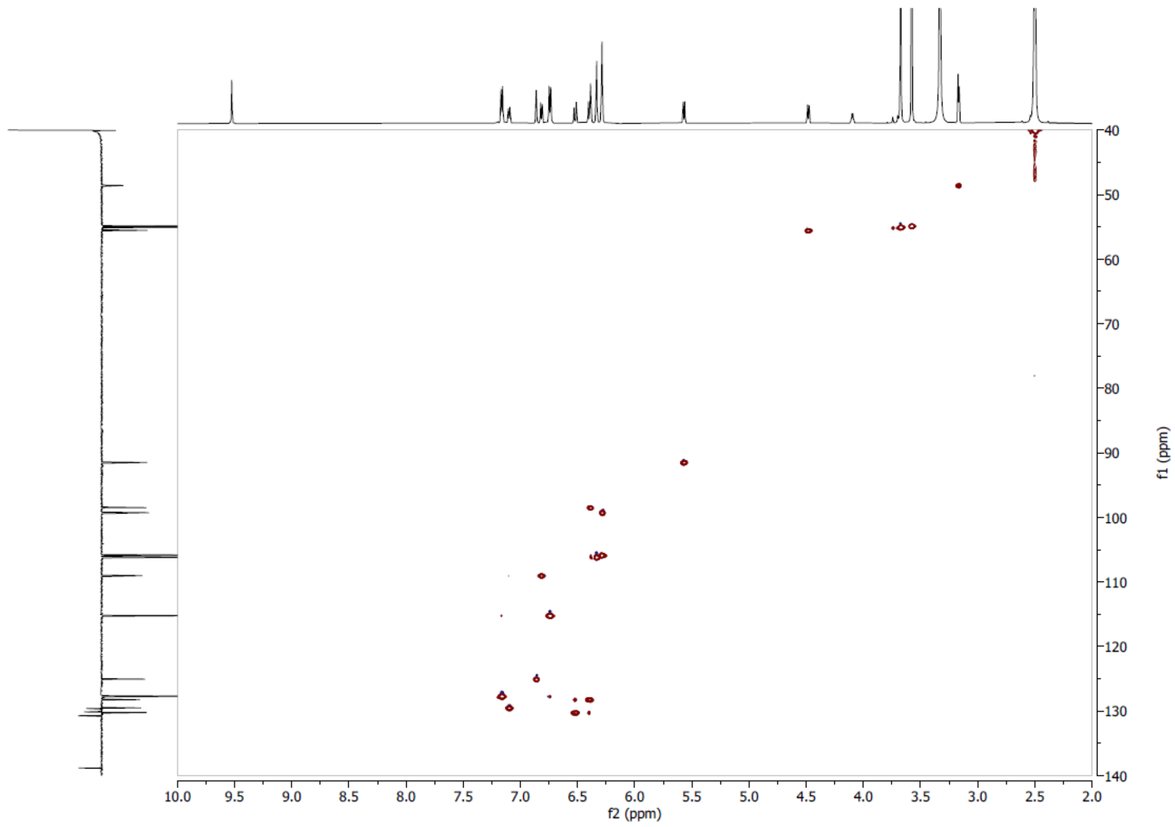

Edited HSQC NMR spectrum of compound **8** in DMSO-d_6

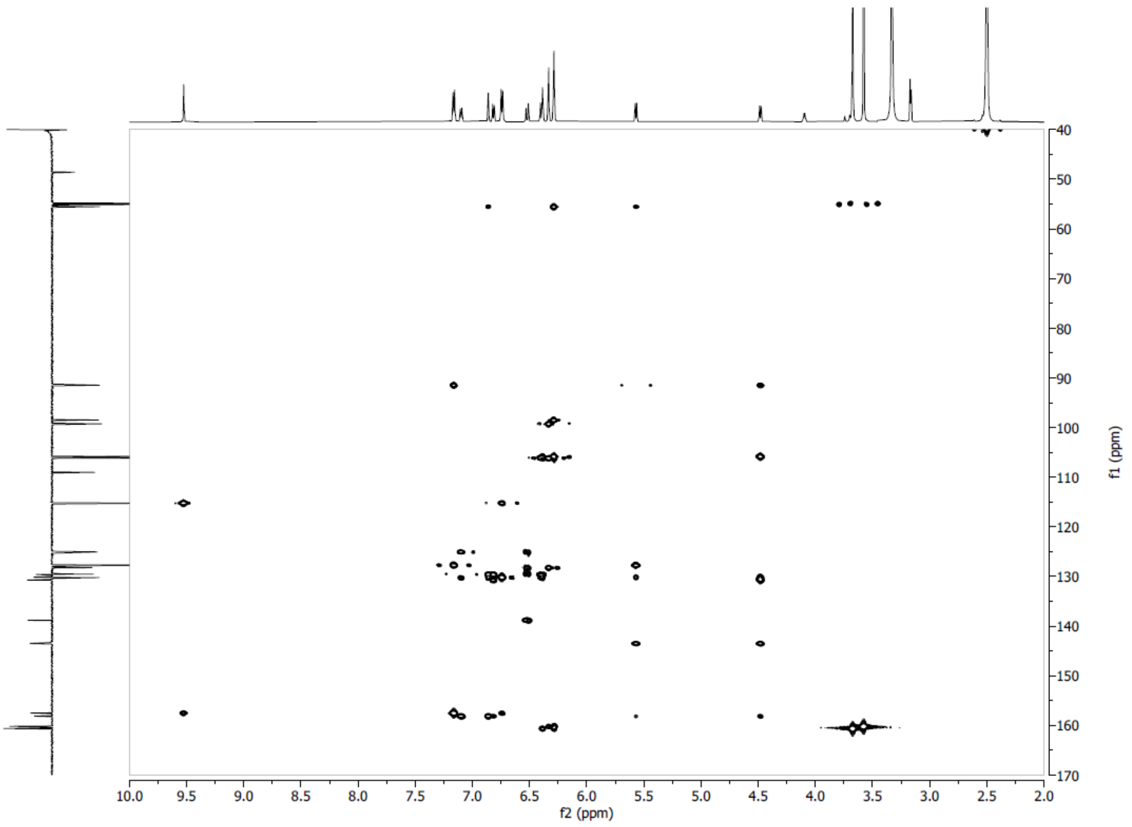

HMBC NMR spectrum of compound **8** in DMSO-d_6

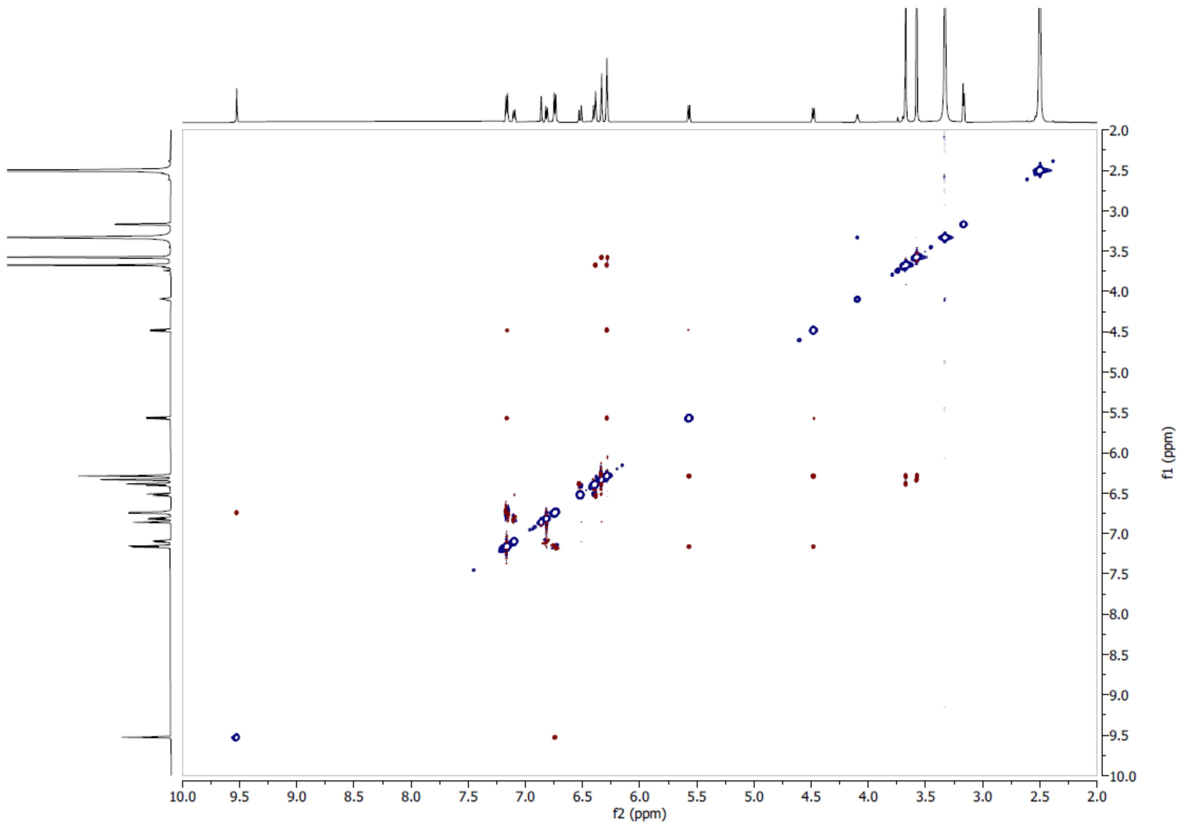

ROESY NMR spectrum of compound **8** in DMSO-d_6

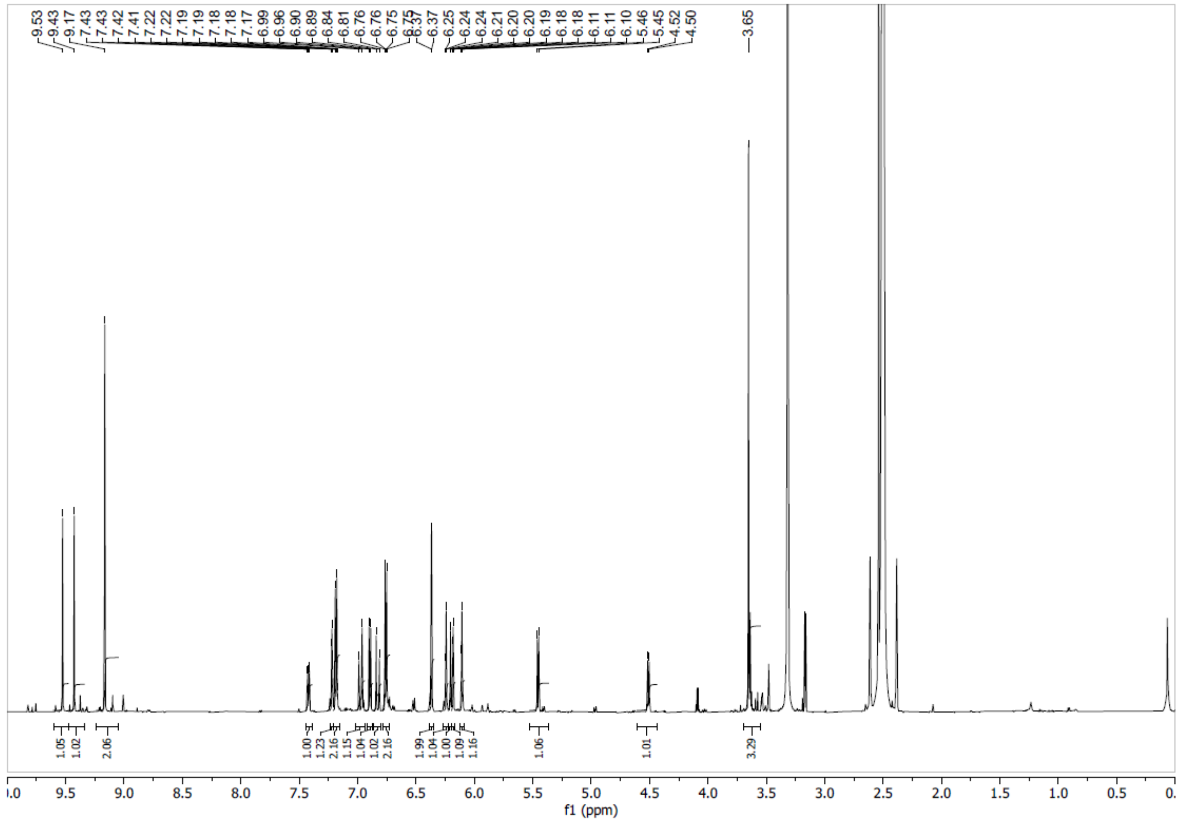

^{1}H NMR spectrum of compound **9** in DMSO-d_6 at 600 MHz

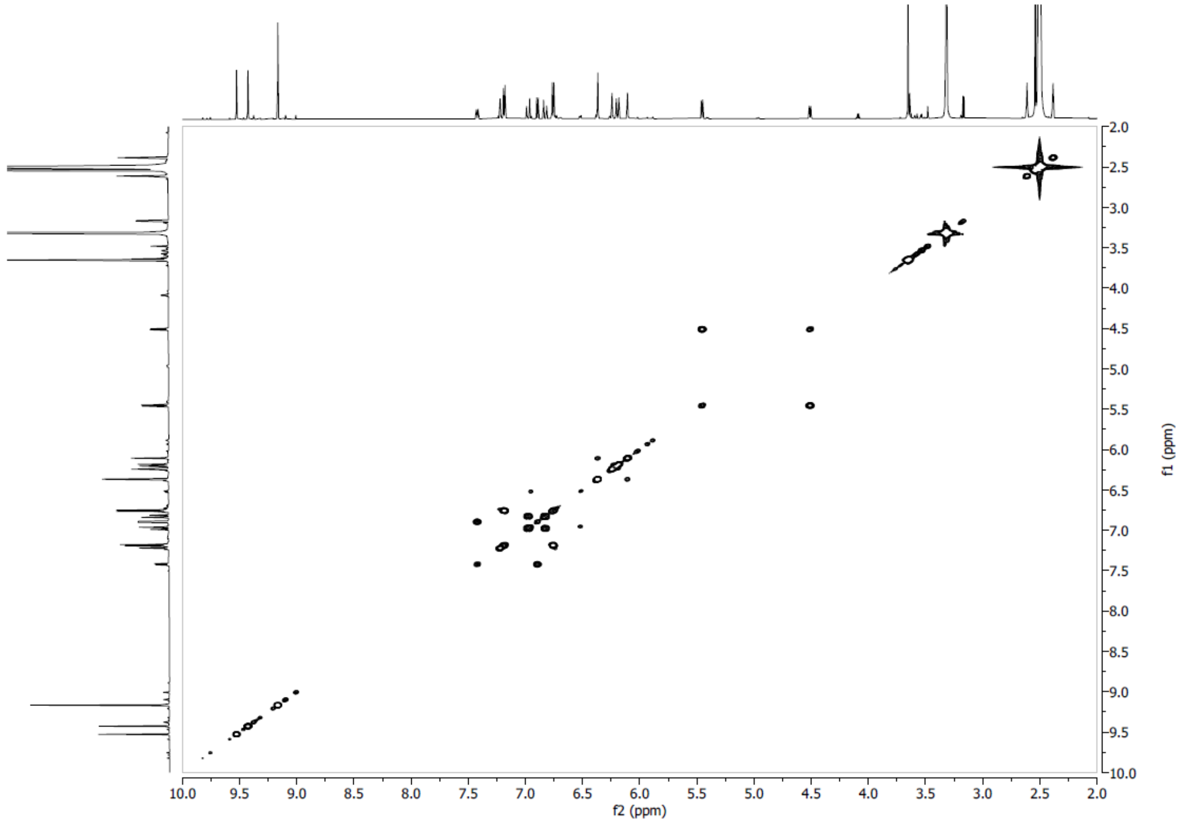

COSY NMR spectrum of compound **9** in DMSO-d_6

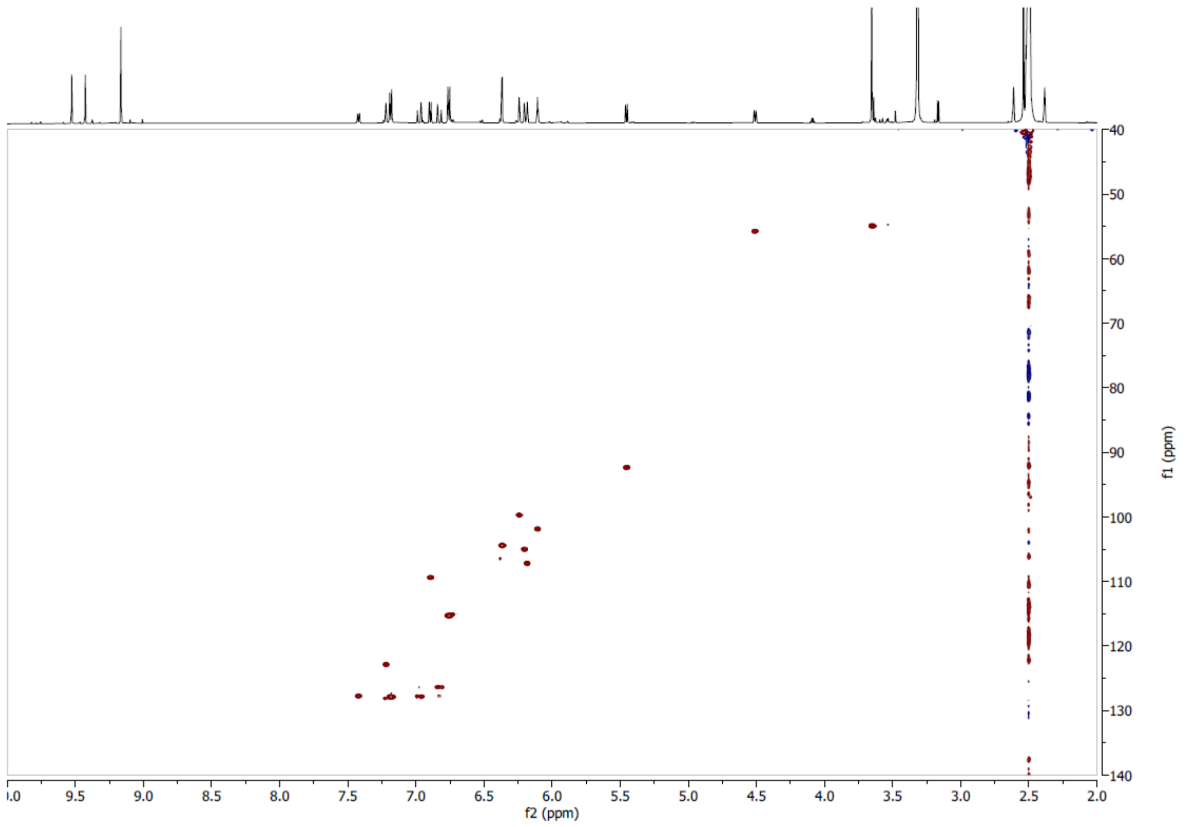

Edited HSQC NMR spectrum of compound **9** in DMSO-d_6

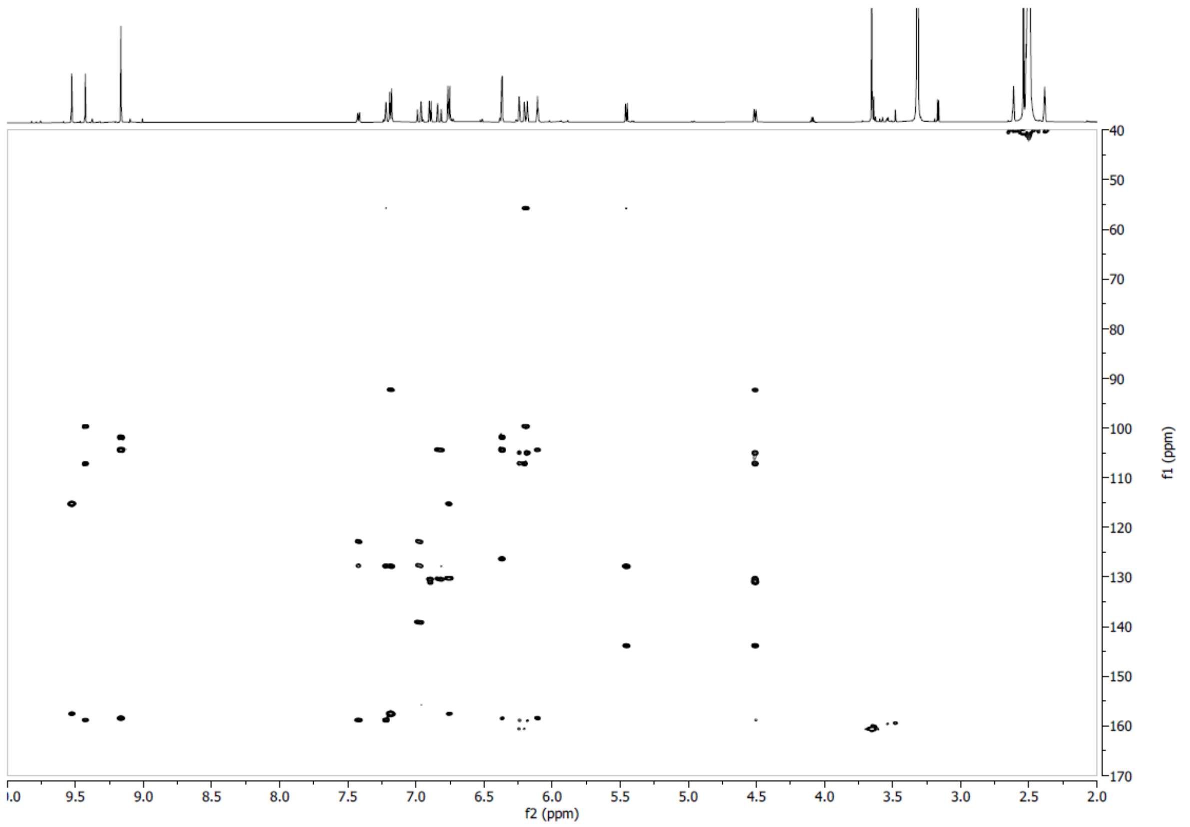

HMBC NMR spectrum of compound **9** in DMSO-d_6

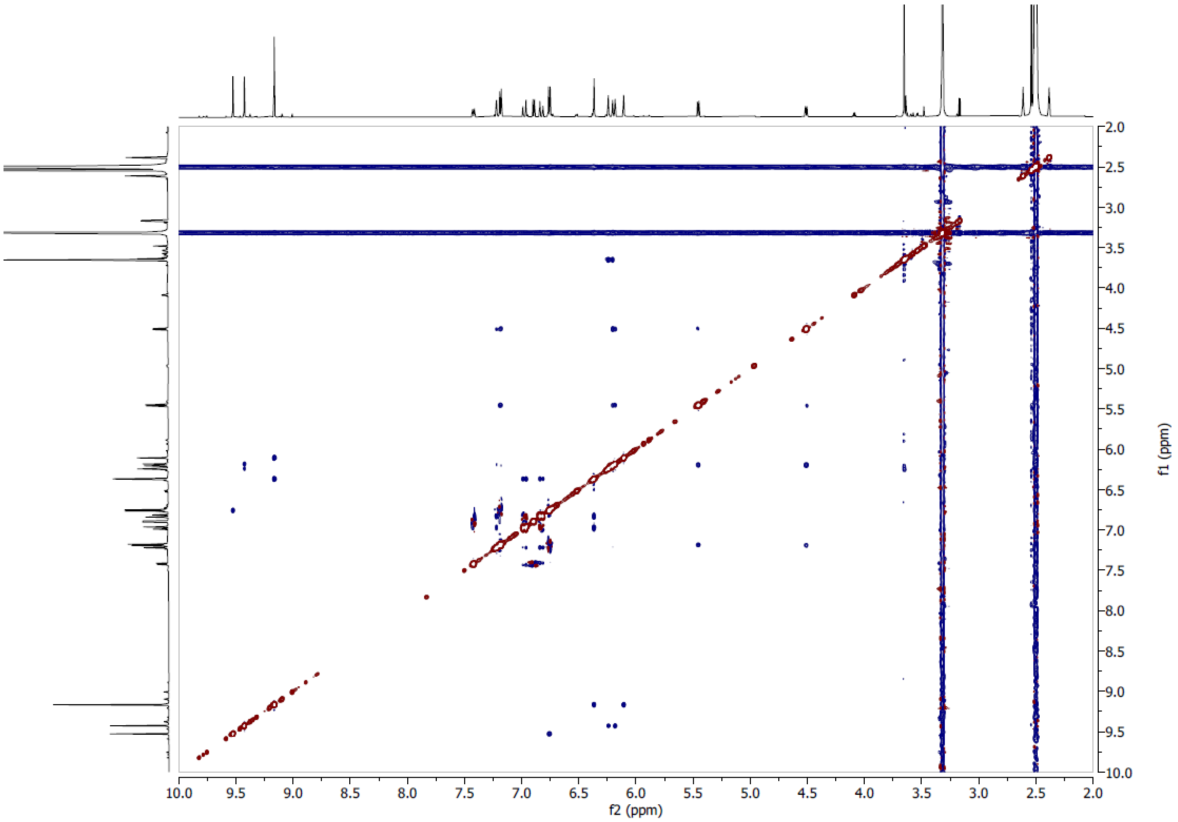

ROESY NMR spectrum of compound **9** in DMSO-d_6

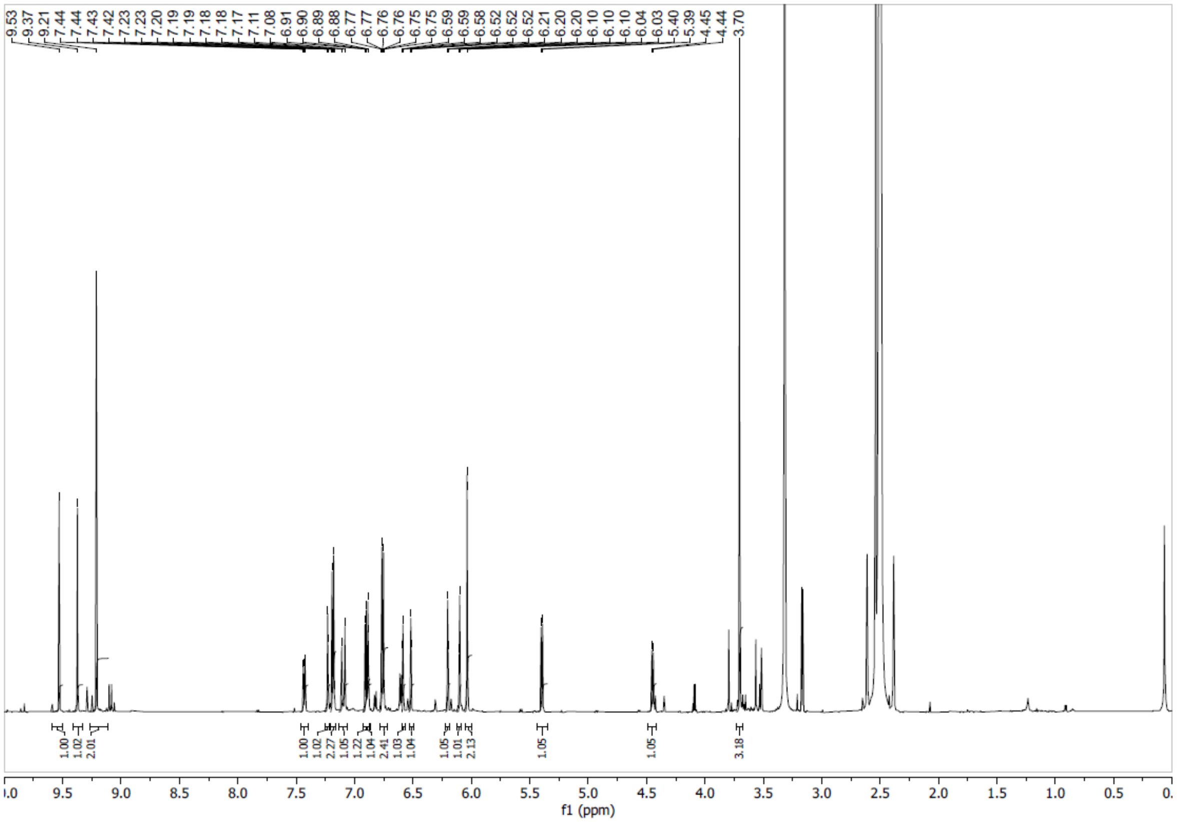

^{1}H NMR spectrum of compound **10** in DMSO-d_6 at 600 MHz

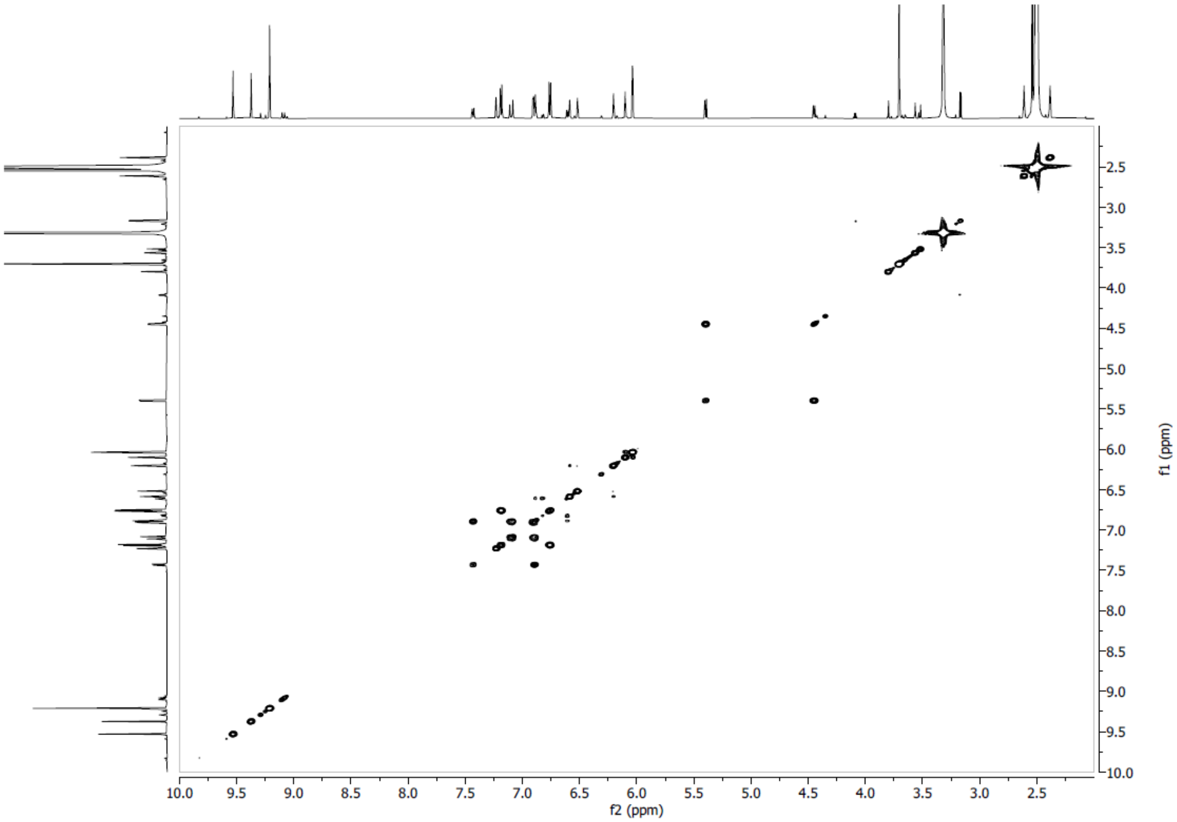

COSY NMR spectrum of compound **10** in DMSO-d_6

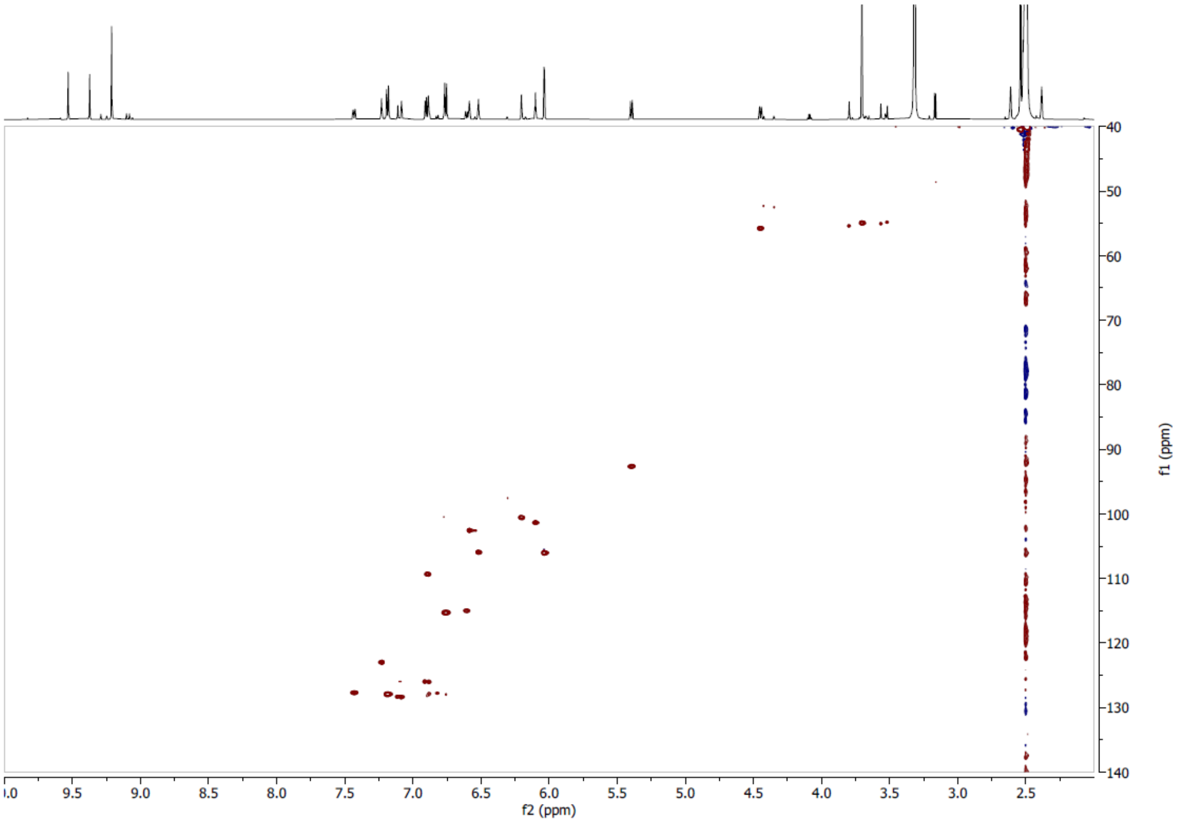

Edited HSQC NMR spectrum of compound **10** in DMSO-d_6

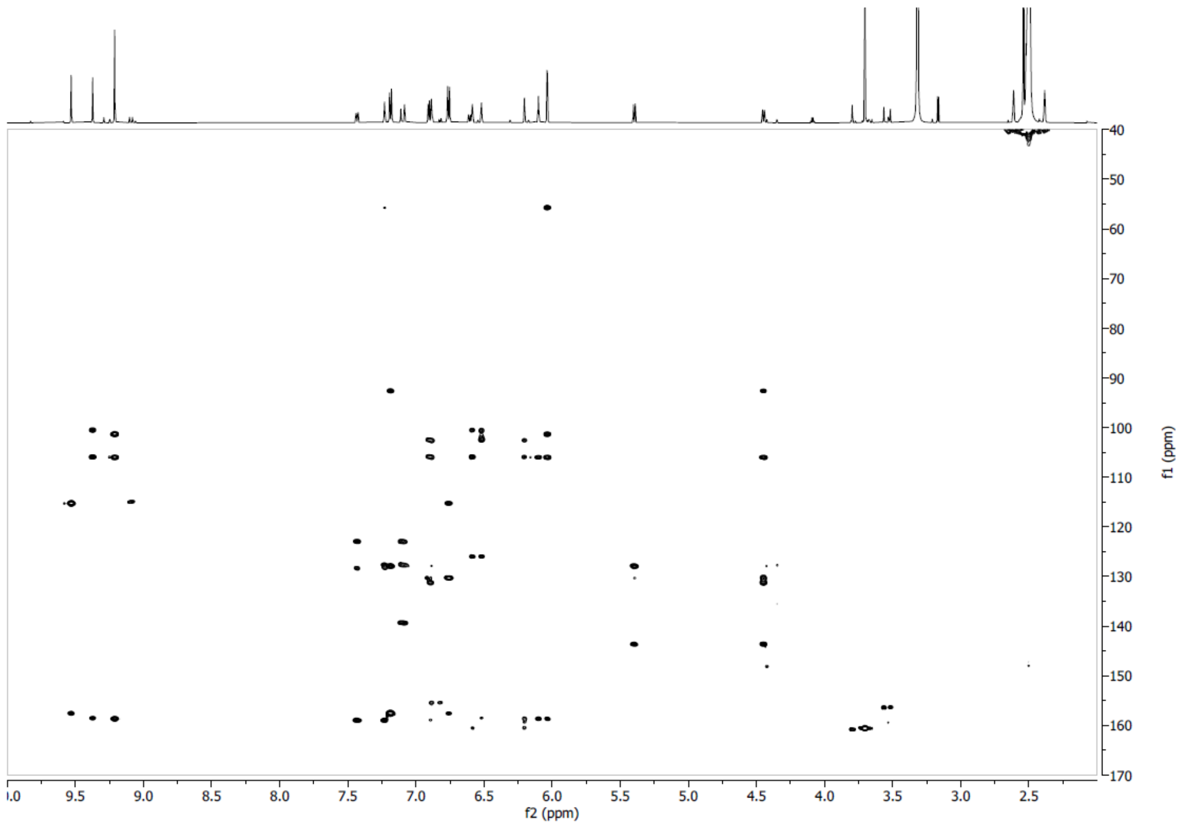

HMBC NMR spectrum of compound **10** in DMSO-d_6

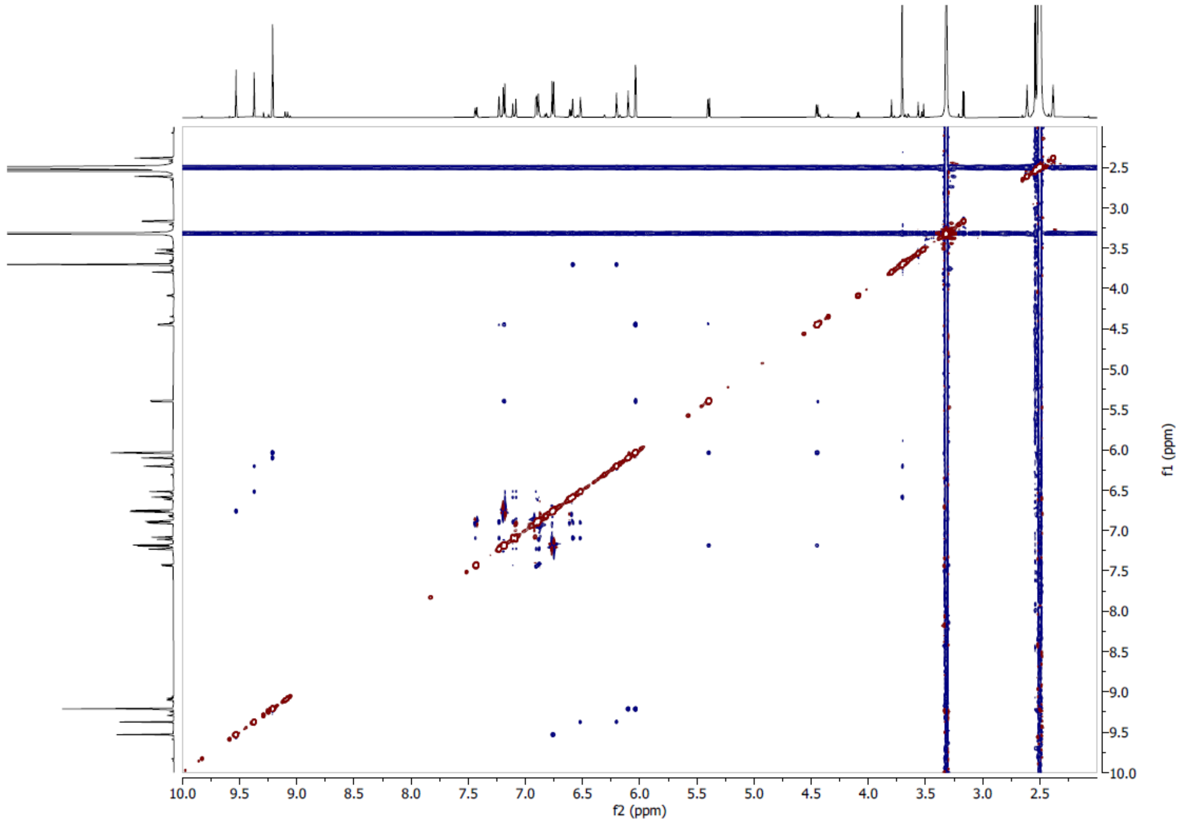

ROESY NMR spectrum of compound **10** in DMSO-d_6

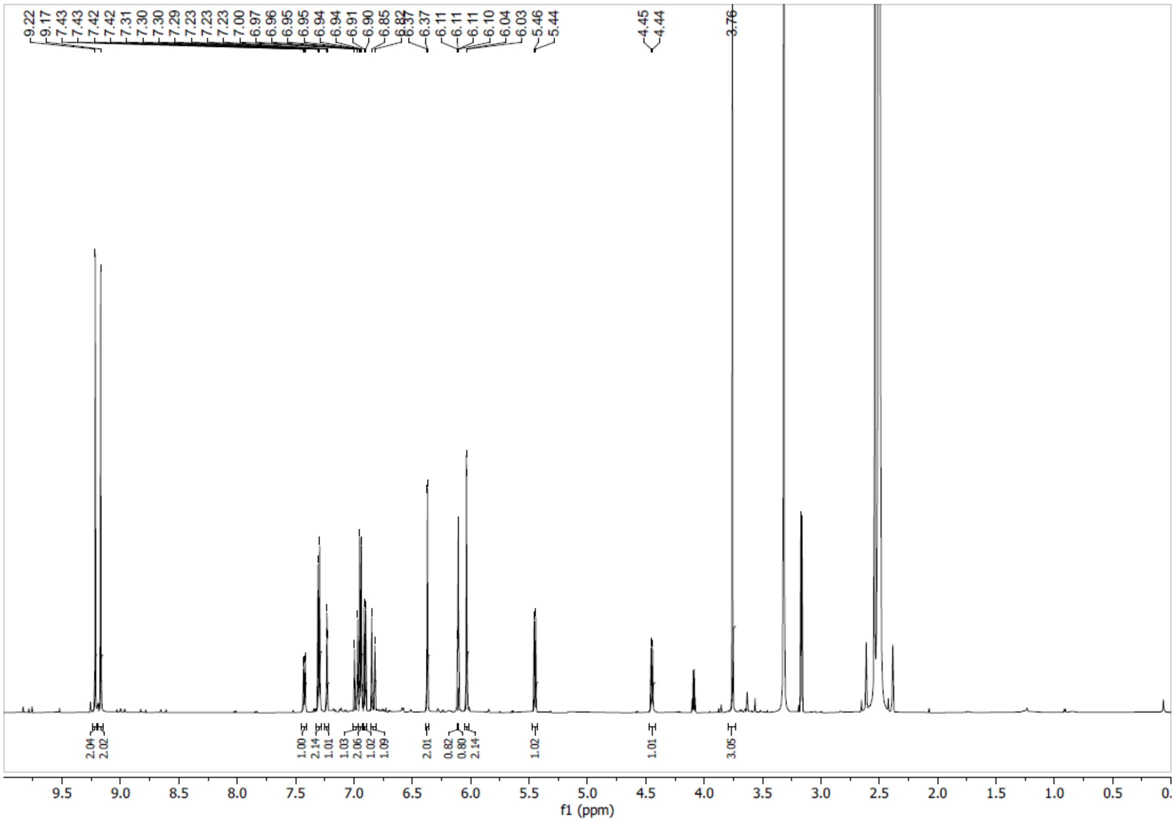

^{1}H NMR spectrum of compound **11** in DMSO-d_6 at 600 MHz

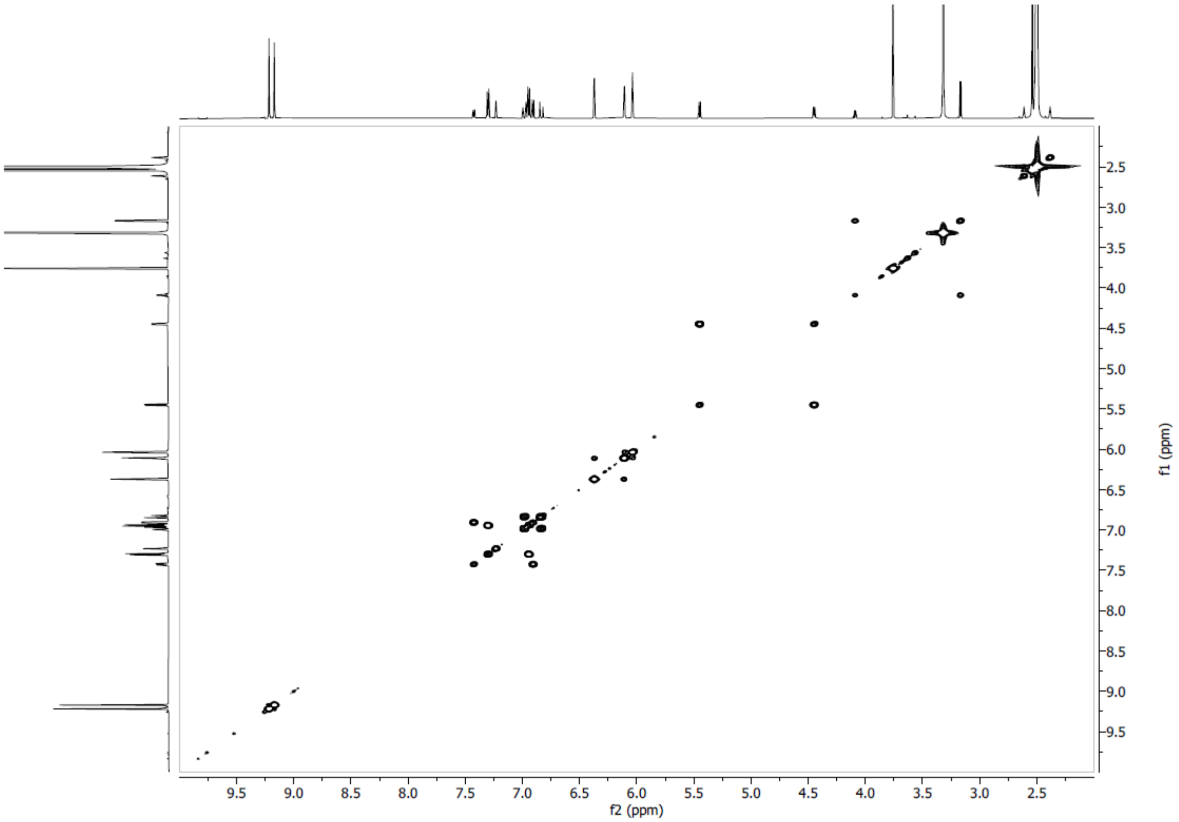

COSY NMR spectrum of compound **11** in DMSO-d_6

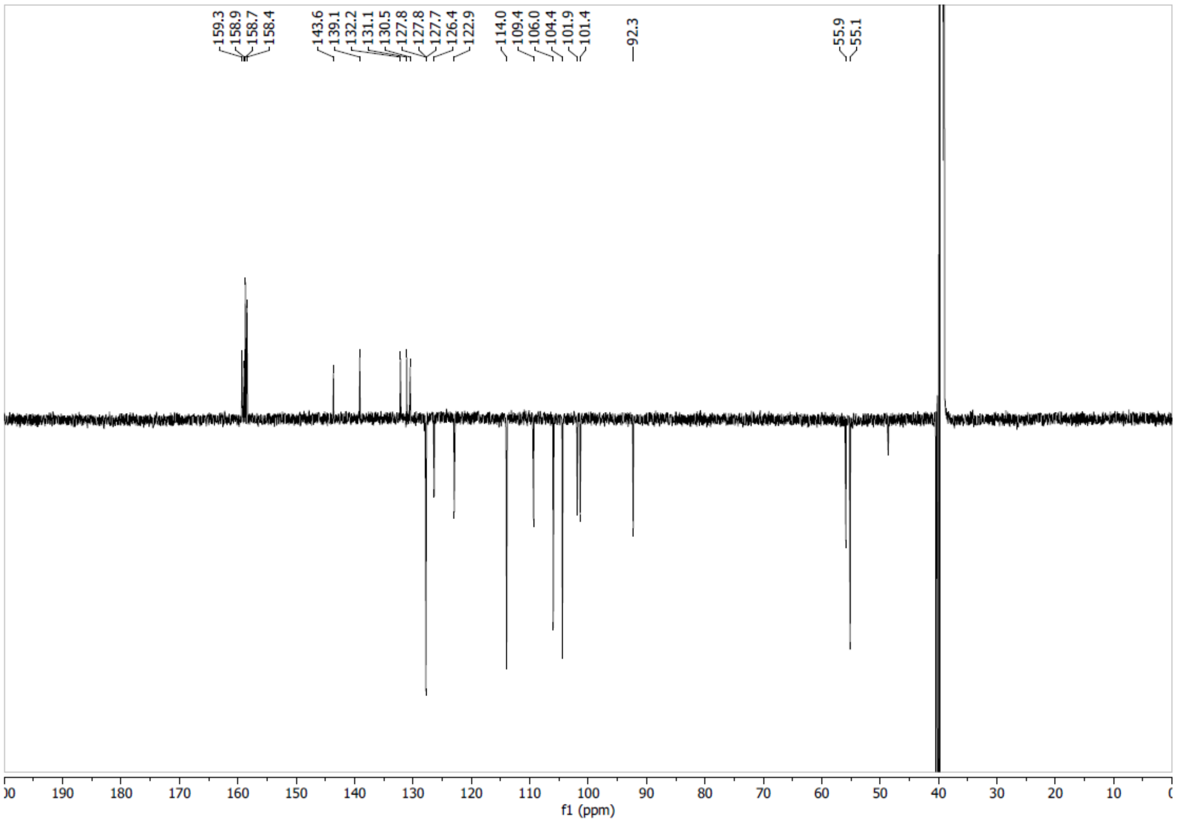

^{13}C-DEPTQ NMR spectrum of compound **11** in DMSO-d_6 at 151 MHz

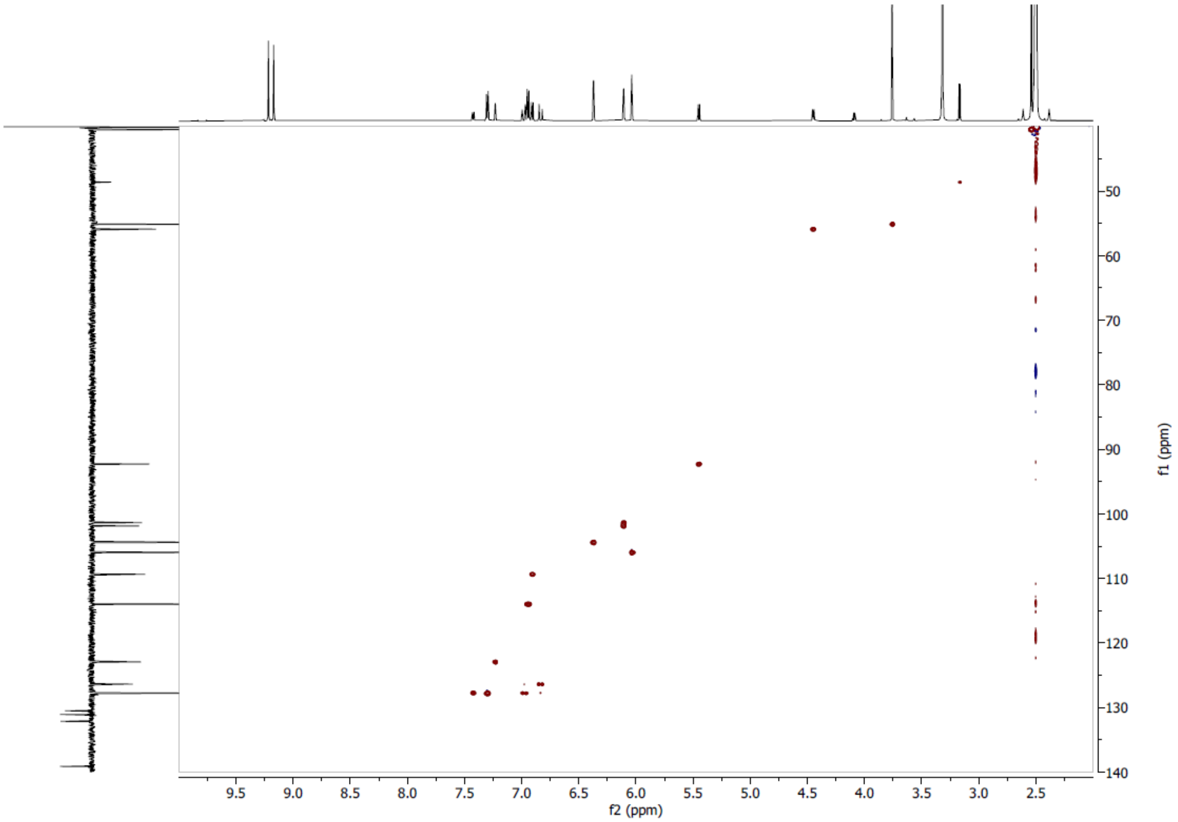

Edited HSQC NMR spectrum of compound **11** in DMSO-d_6

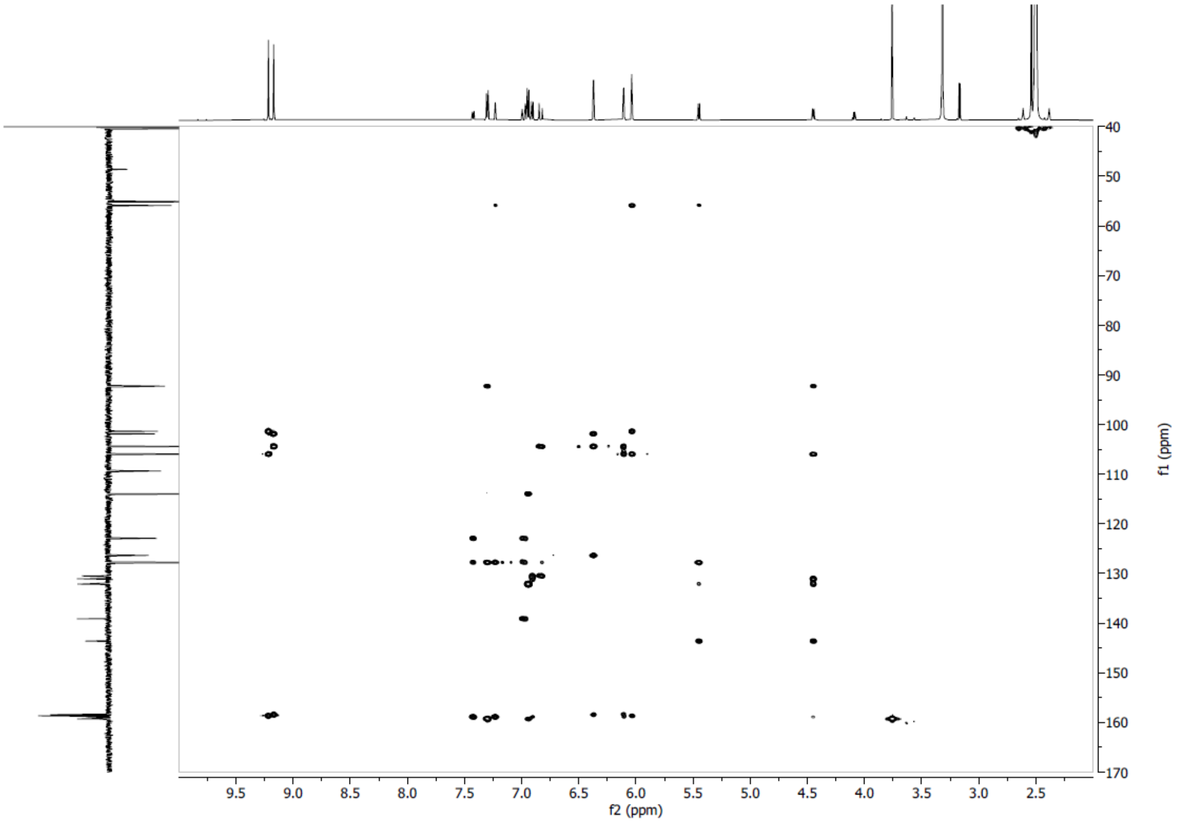

HMBC NMR spectrum of compound **11** in DMSO-d_6

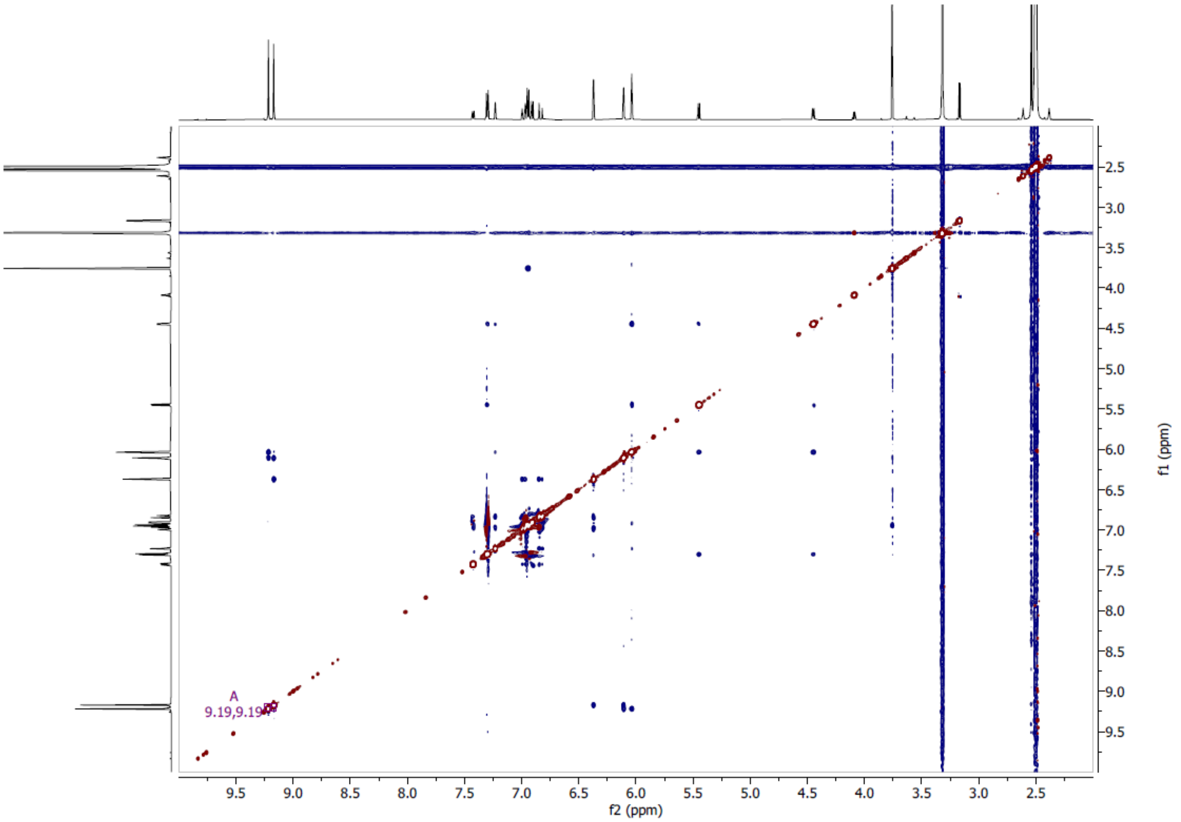

ROESY NMR spectrum of compound **11** in DMSO-d_6

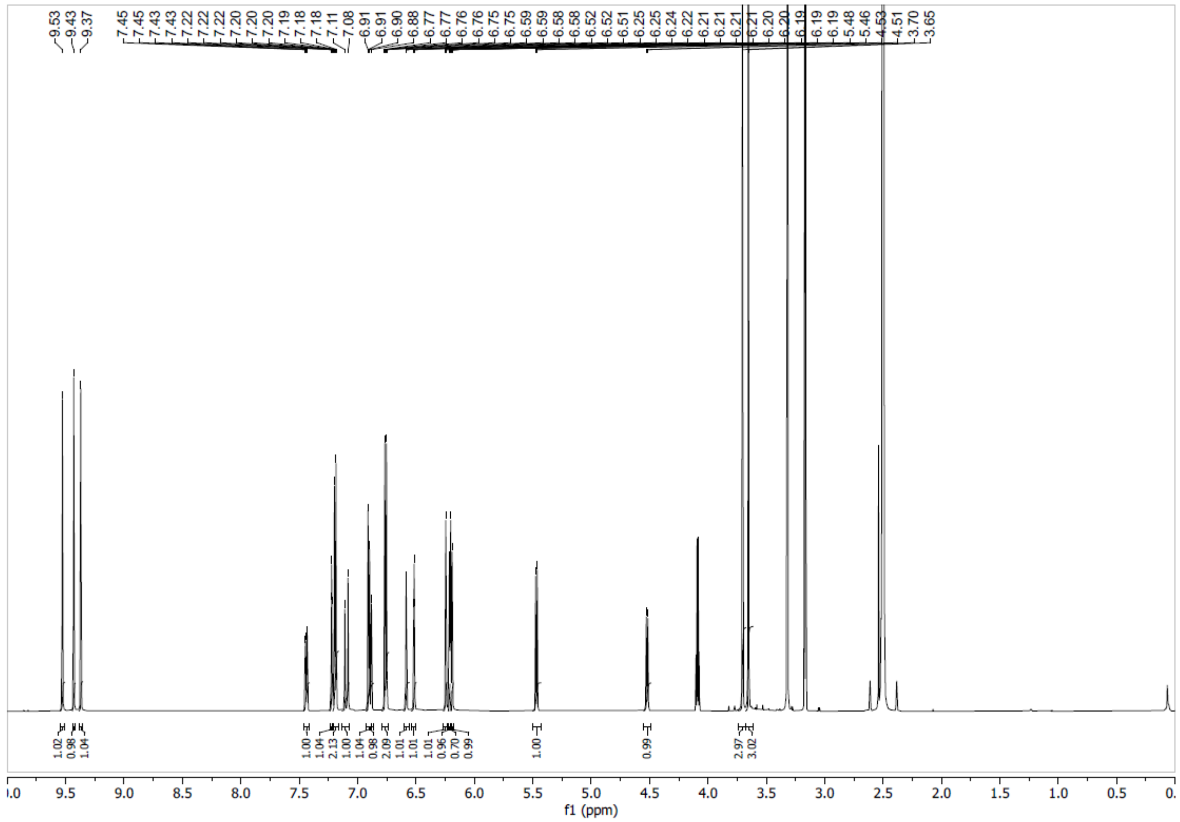

^{1}H NMR spectrum of compound **12** in DMSO-d_6 at 600 MHz

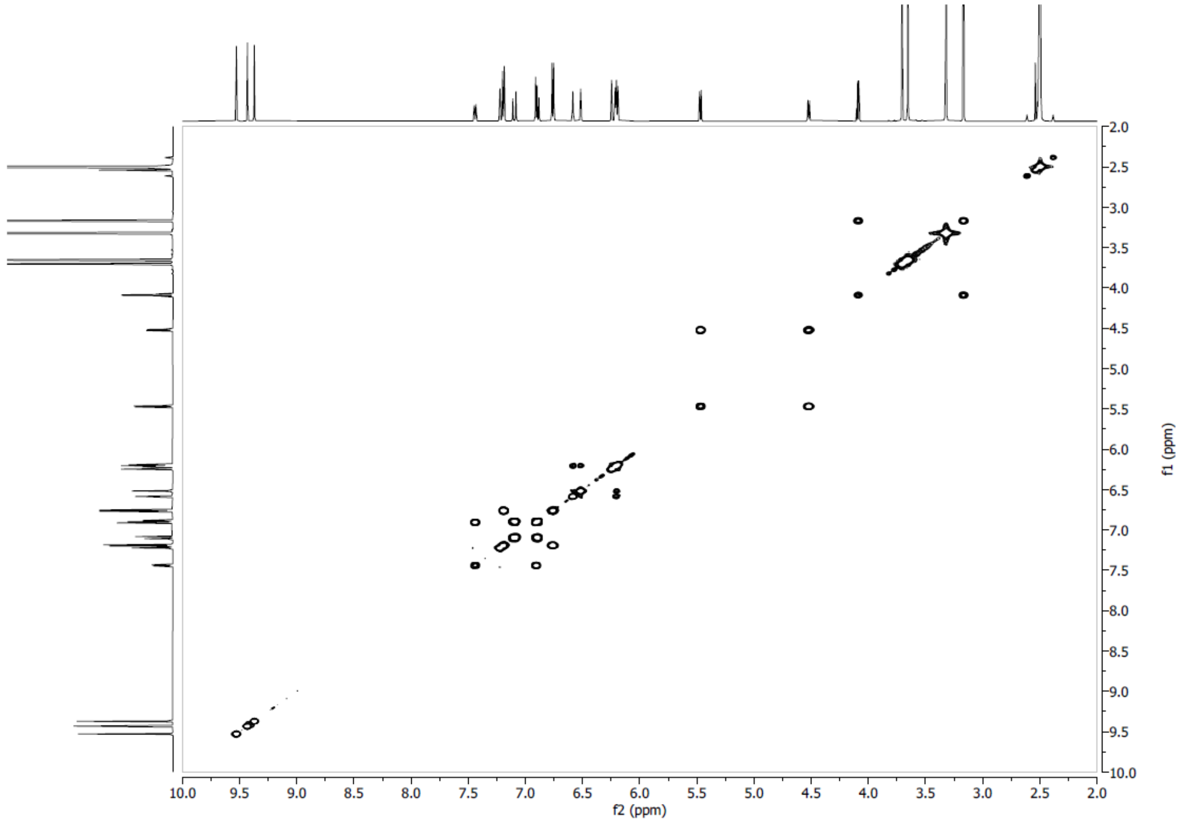

COSY NMR spectrum of compound **12** in DMSO-d_6

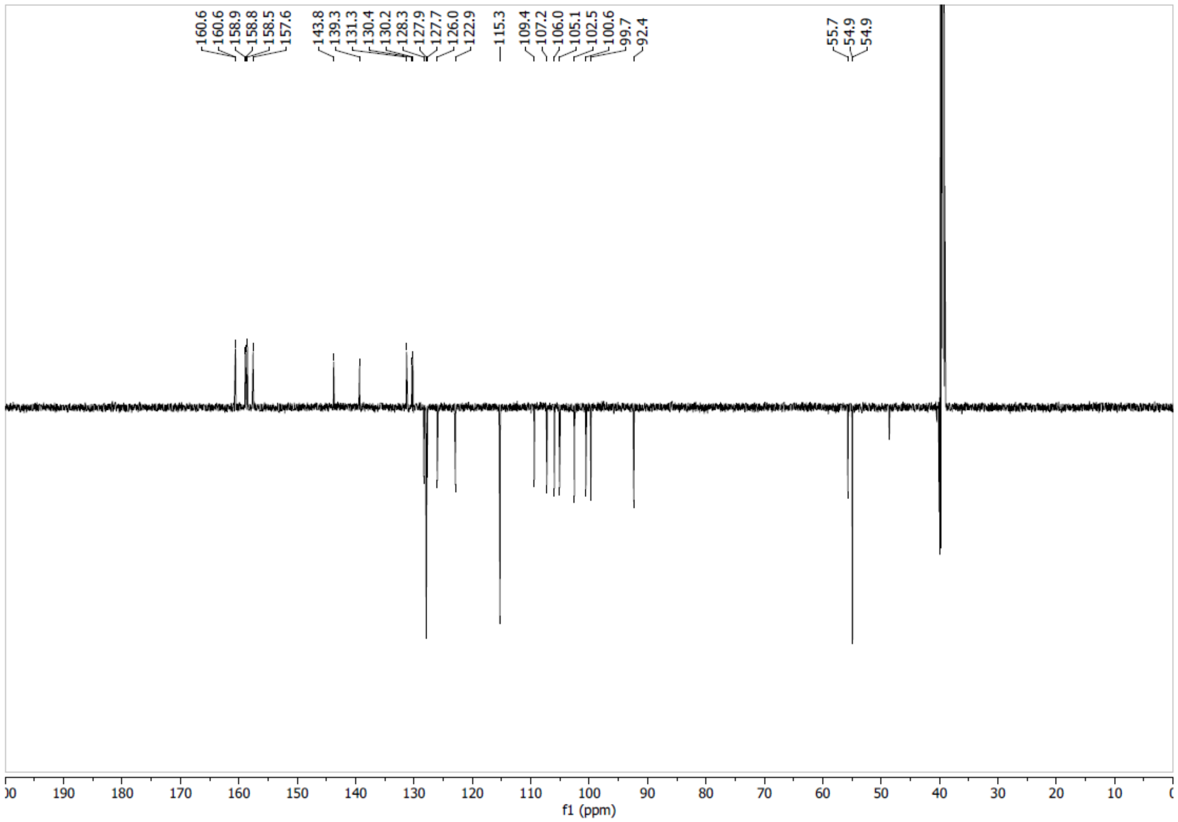

^{13}C-DEPTQ NMR spectrum of compound **12** in DMSO-d_6 at 151 MHz

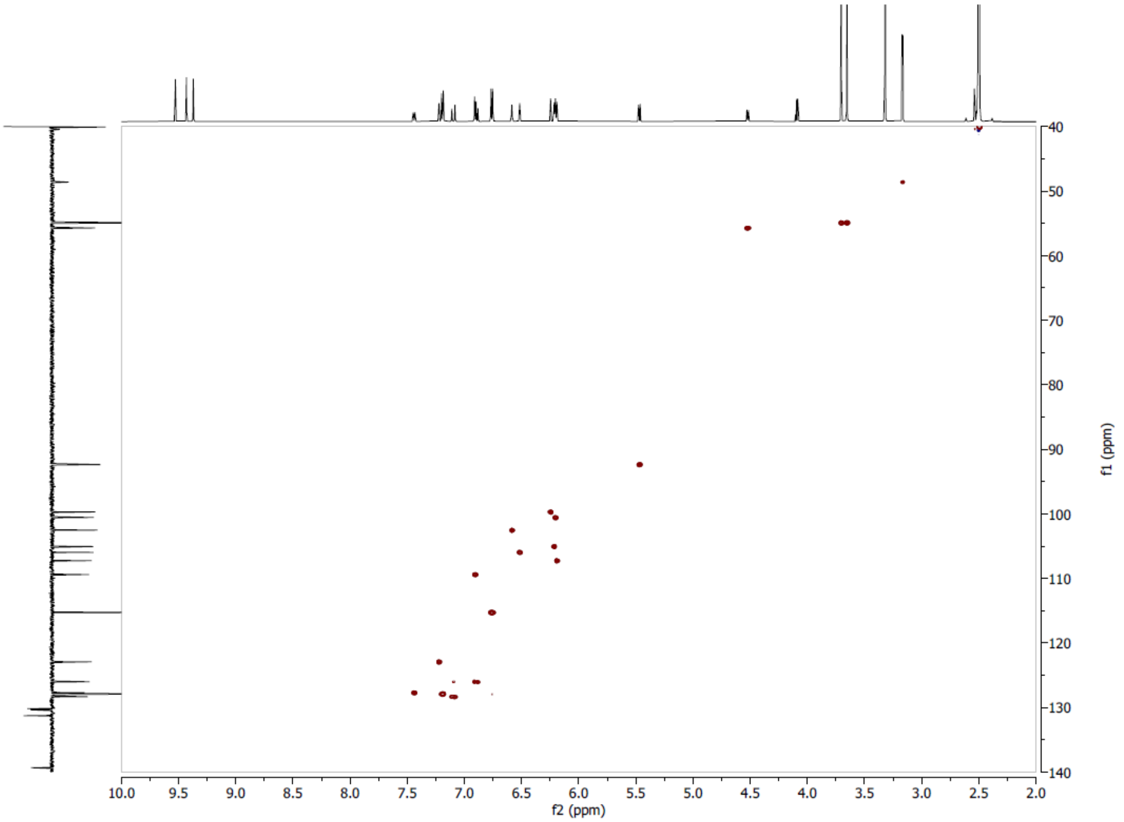

Edited HSQC NMR spectrum of compound **12** in DMSO-d_6

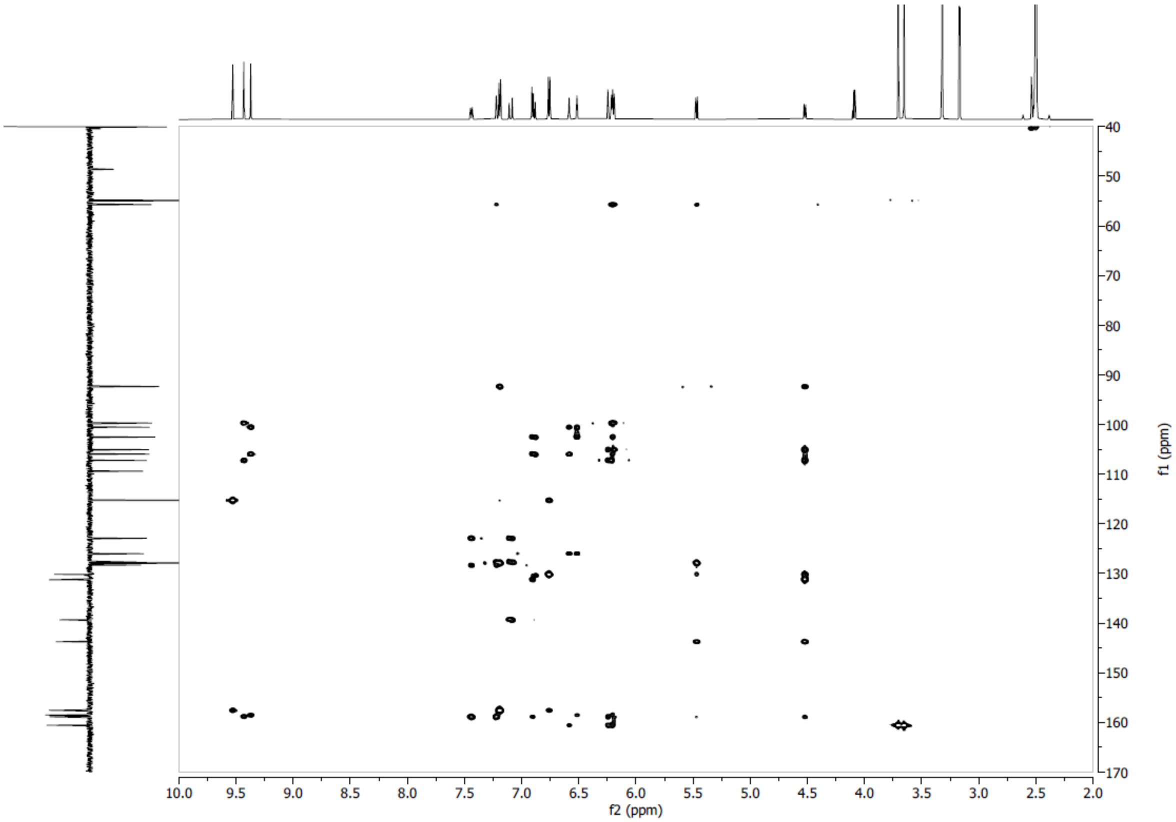

HMBC NMR spectrum of compound **12** in DMSO-d_6

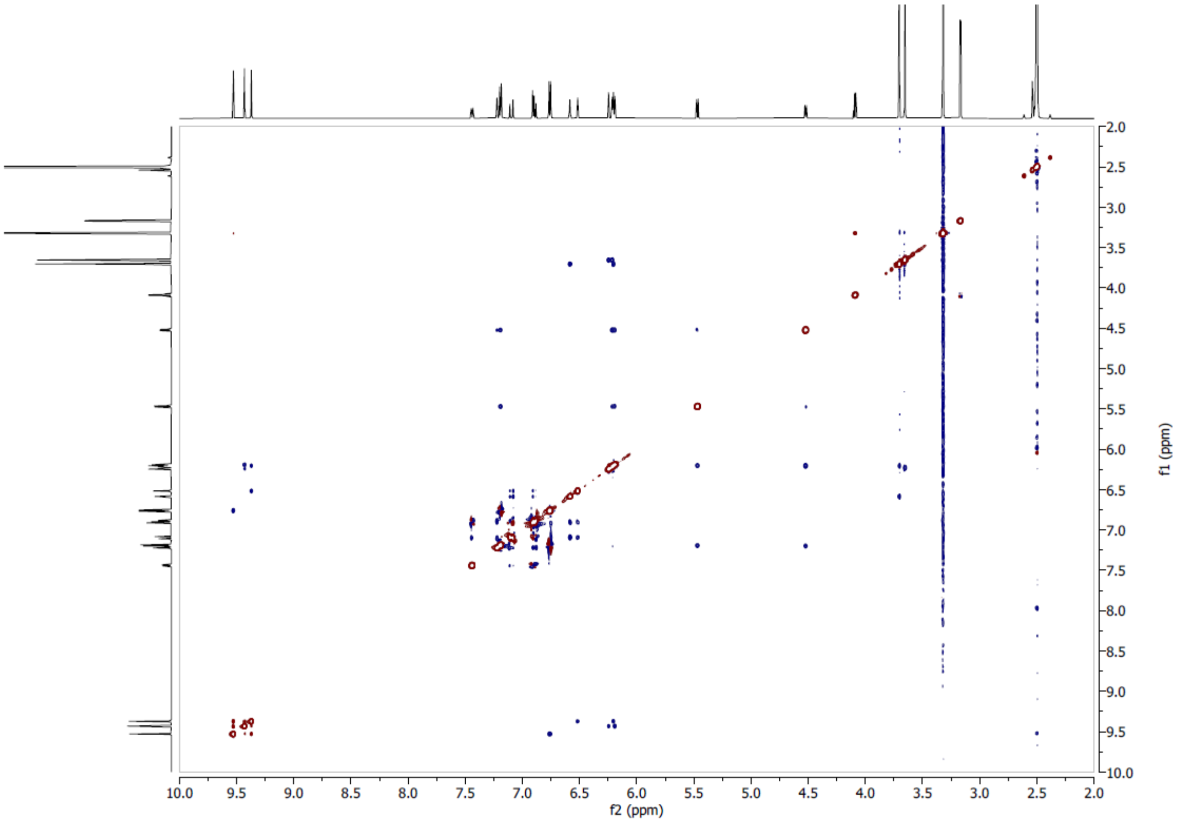

ROESY NMR spectrum of compound **12** in DMSO-d_6

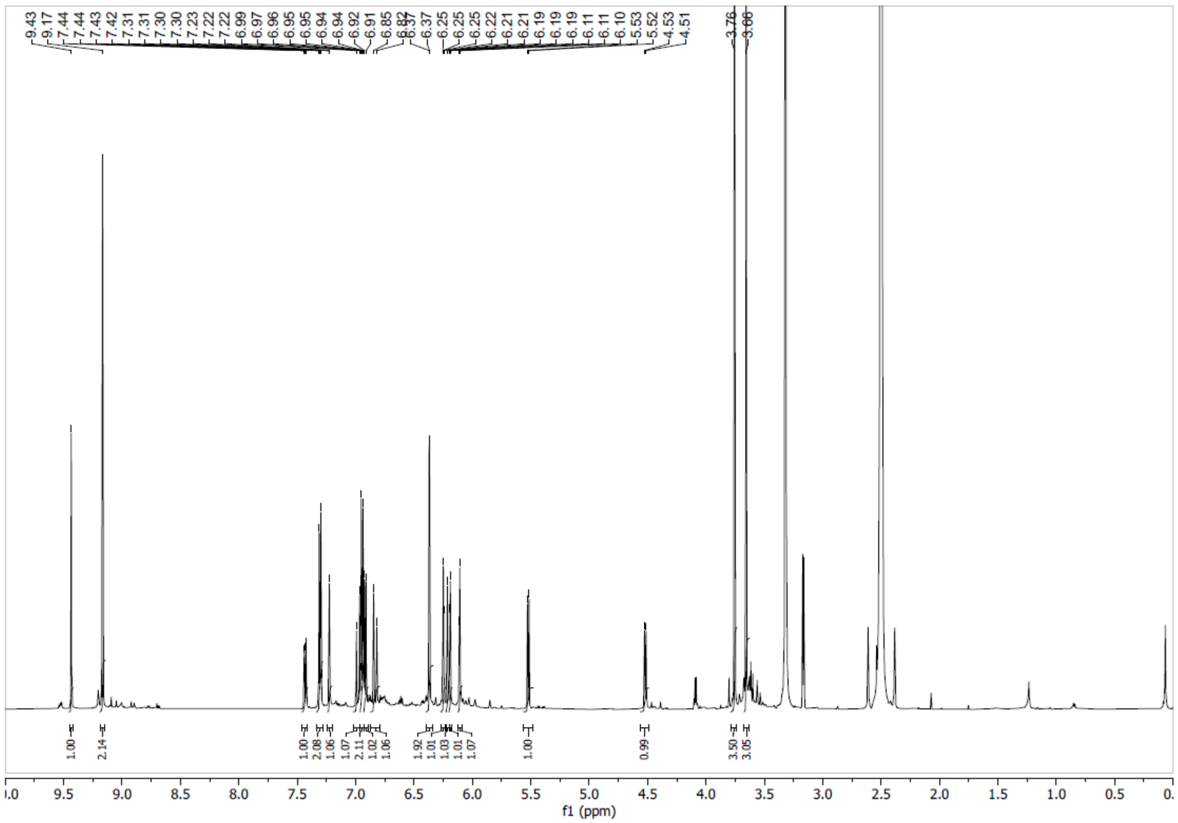

^{1}H NMR spectrum of compound **13** in DMSO-d_6 at 600 MHz

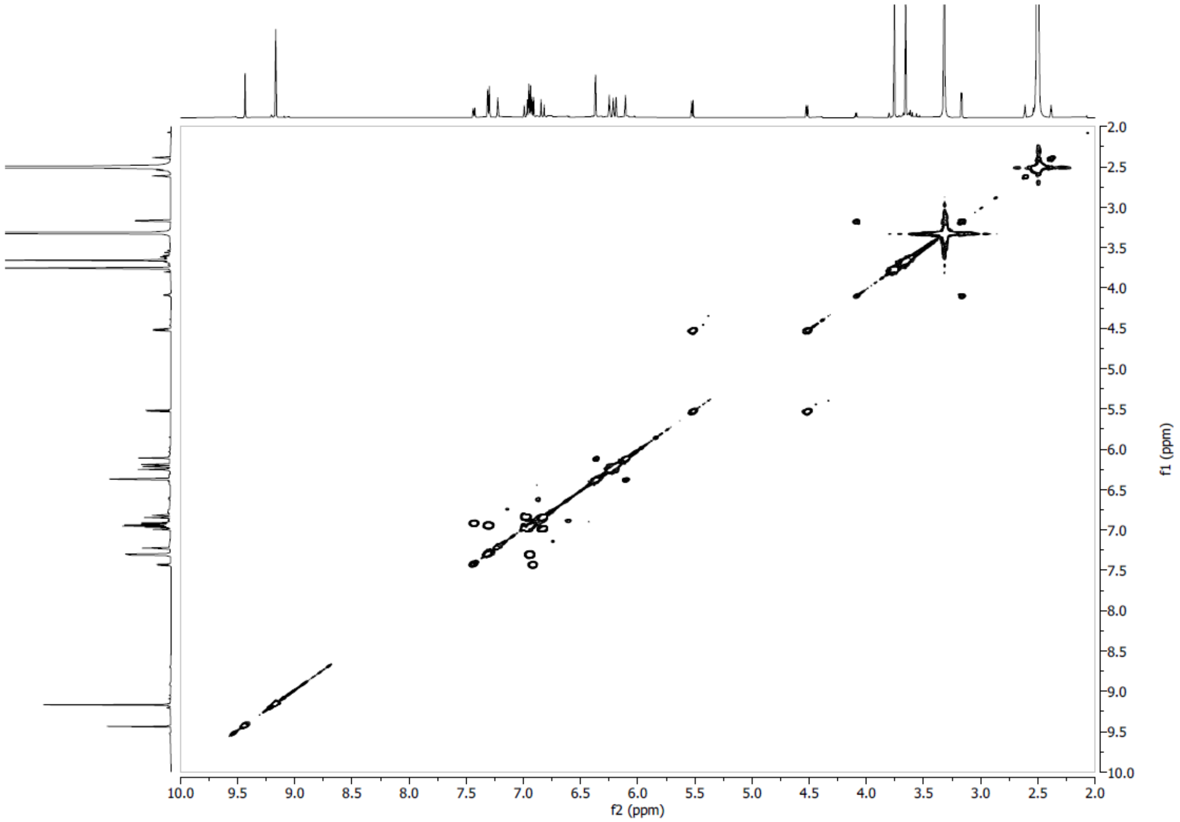

COSY NMR spectrum of compound **13** in DMSO-d_6

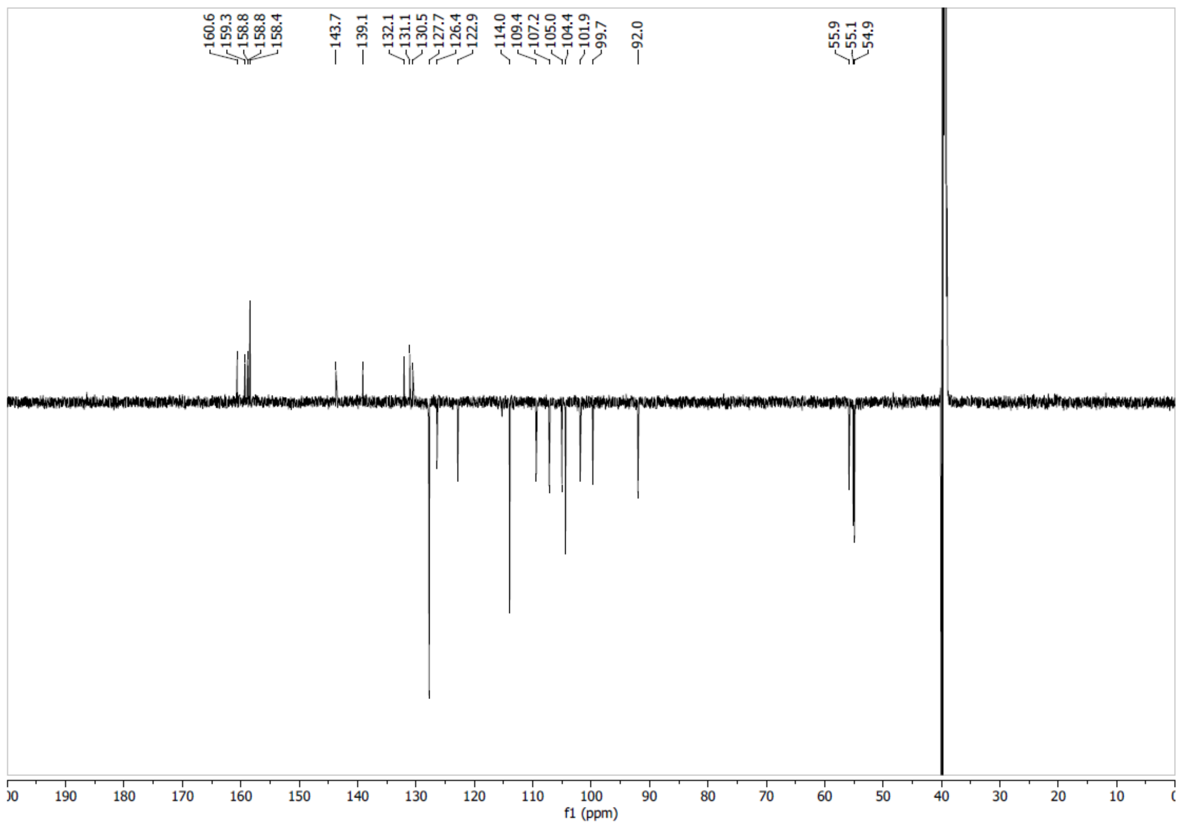

^{13}C-DEPTQ NMR spectrum of compound **13** in DMSO-d_6 at 151 MHz

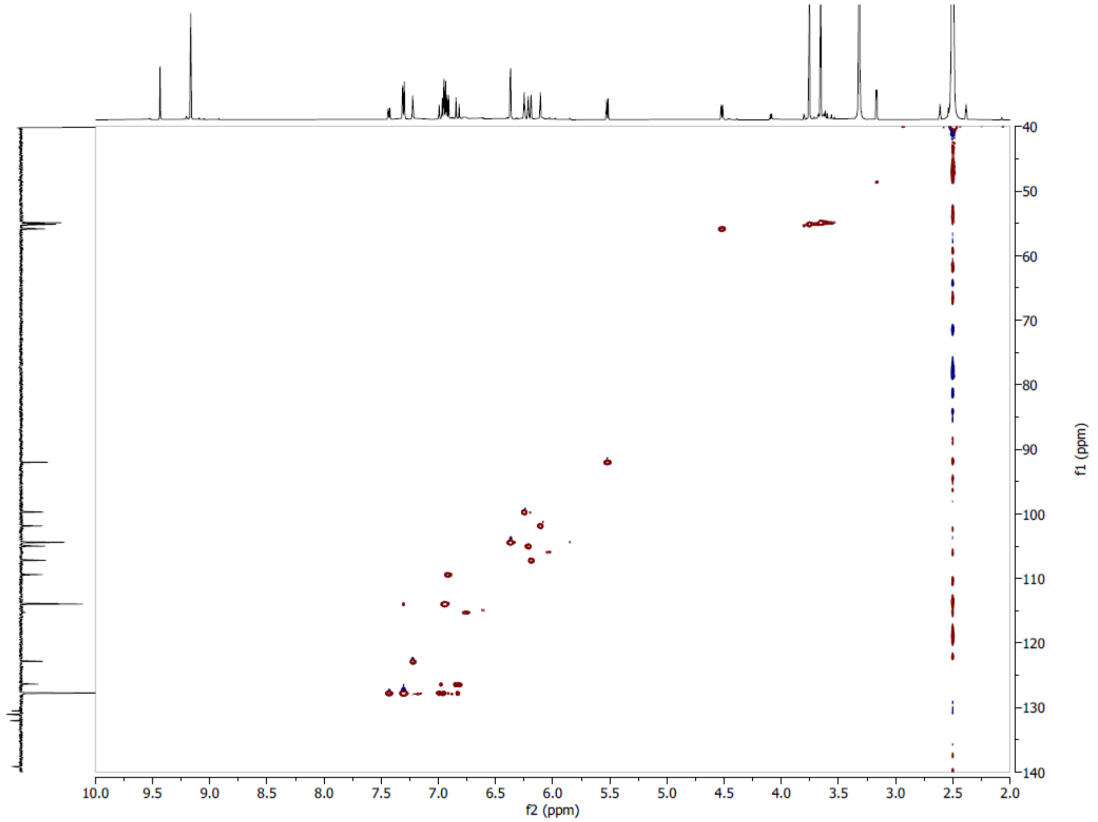

Edited HSQC NMR spectrum of compound **13** in DMSO-d_6

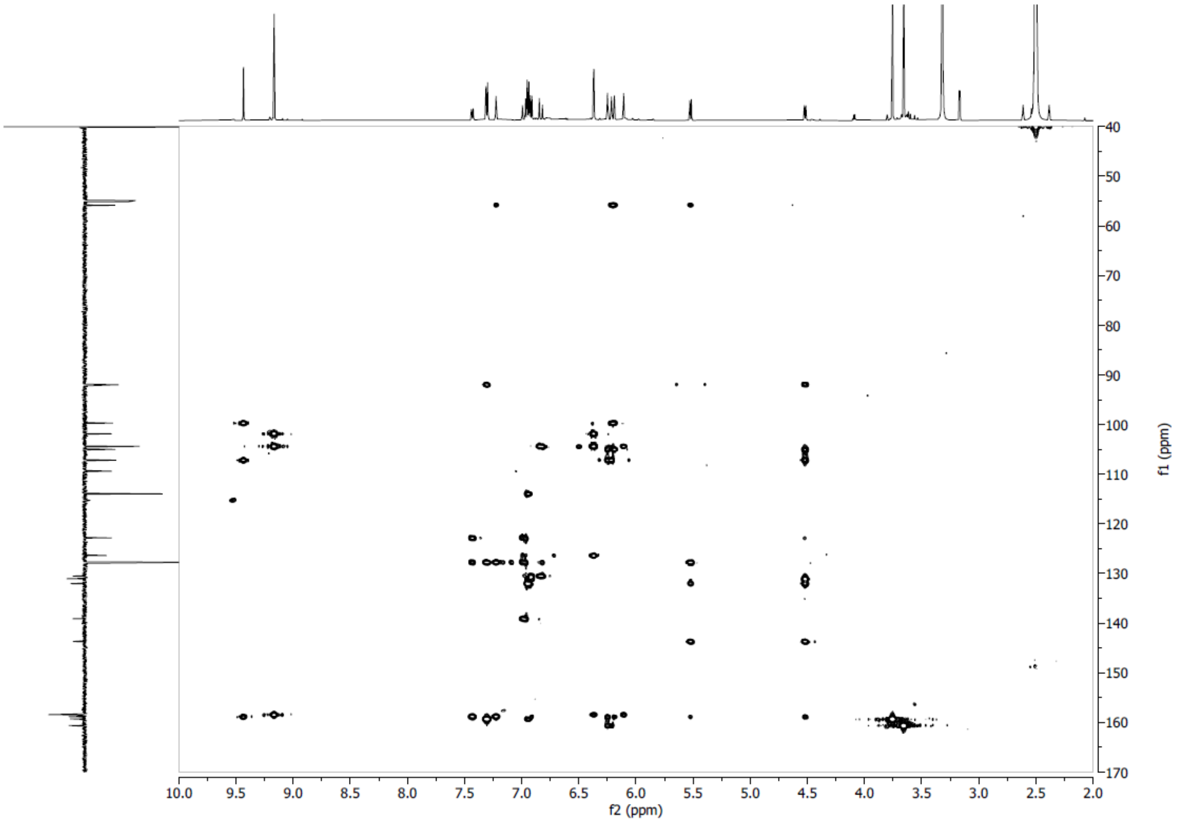

HMBC NMR spectrum of compound **13** in DMSO-d_6

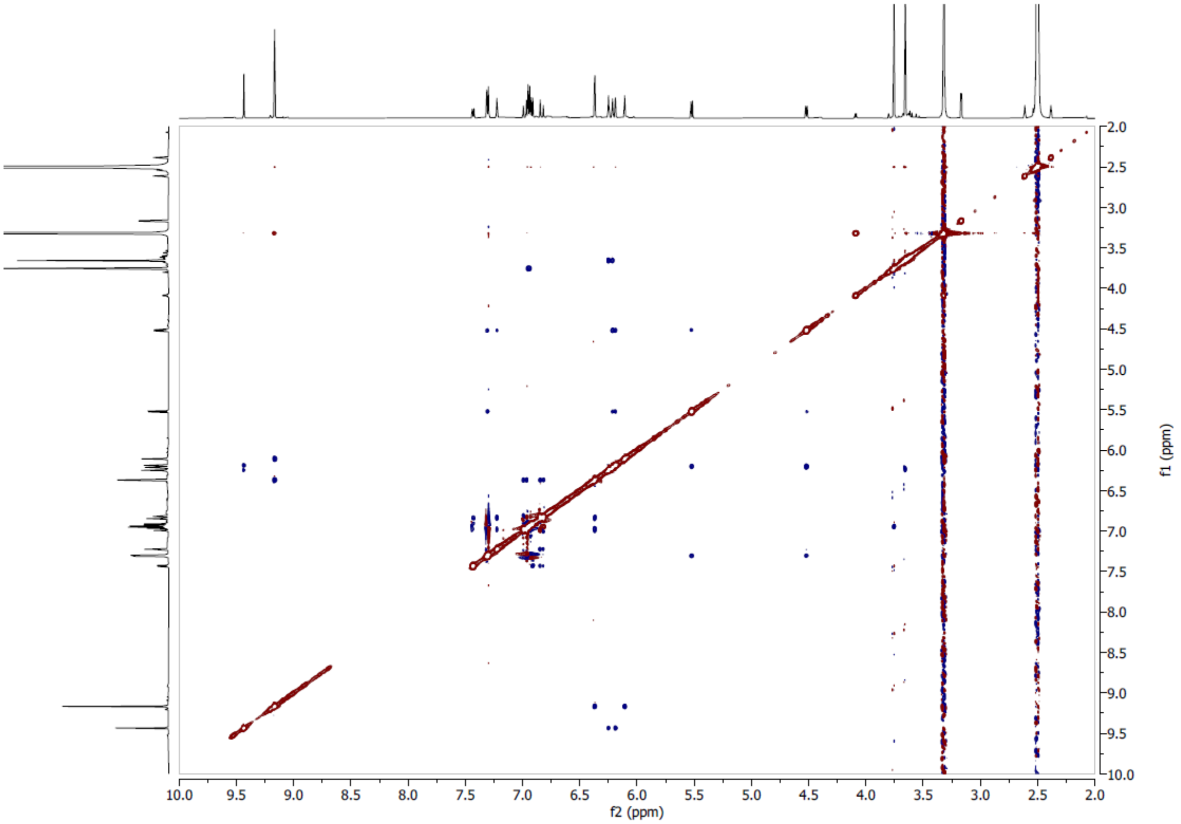

ROESY NMR spectrum of compound **13** in DMSO-d_6

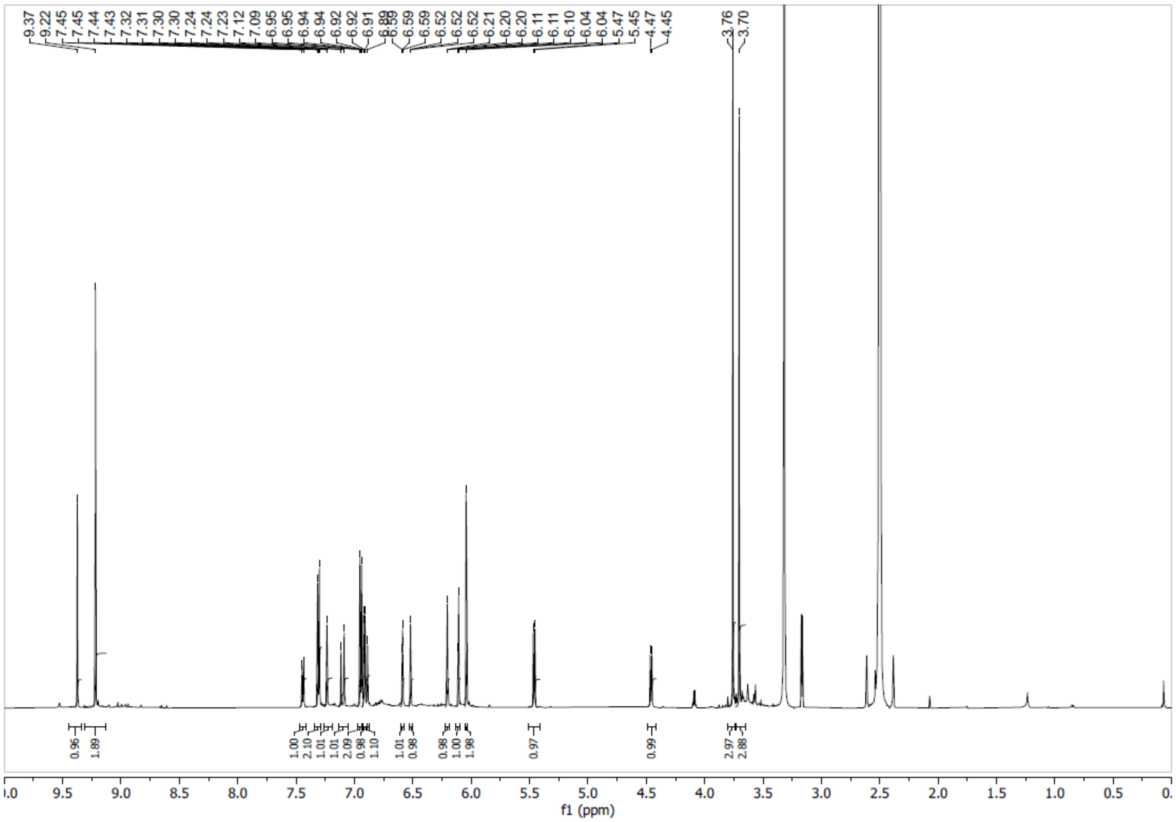

^{1}H NMR spectrum of compound **14** in DMSO-d_6 at 600 MHz

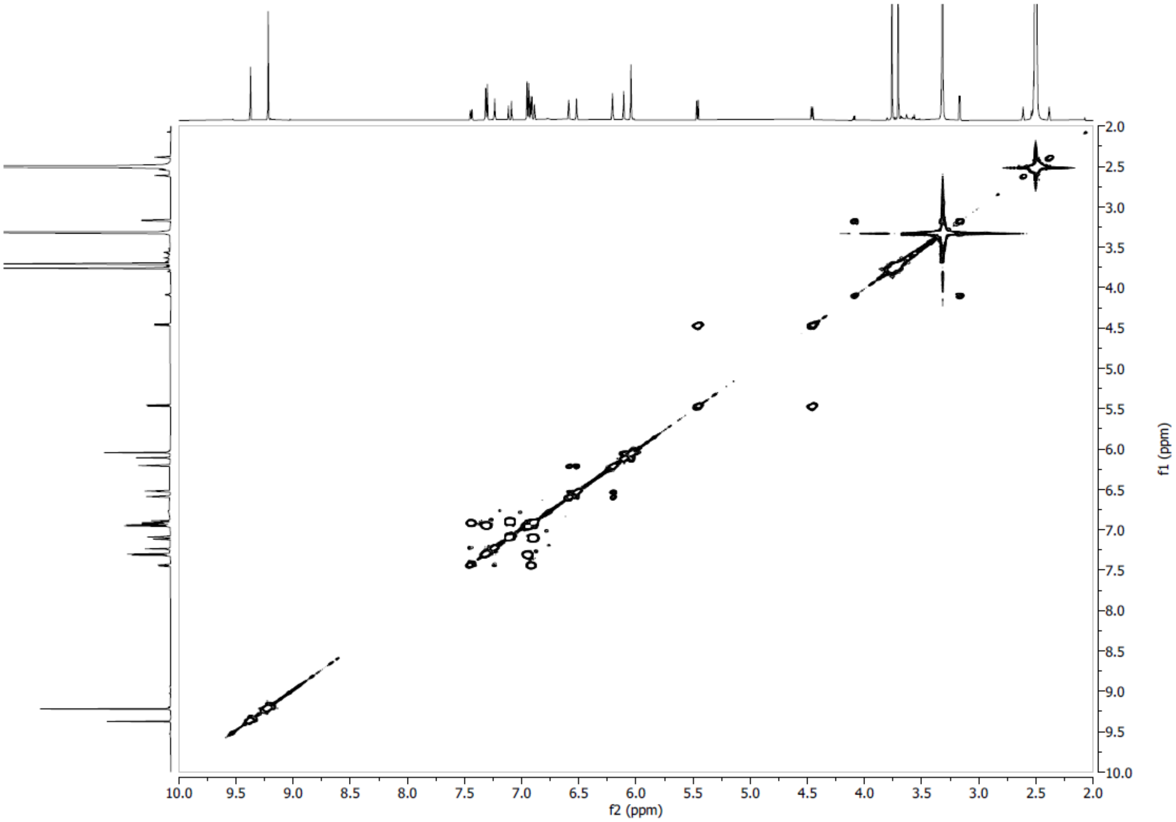

COSY NMR spectrum of compound **14** in DMSO-d_6

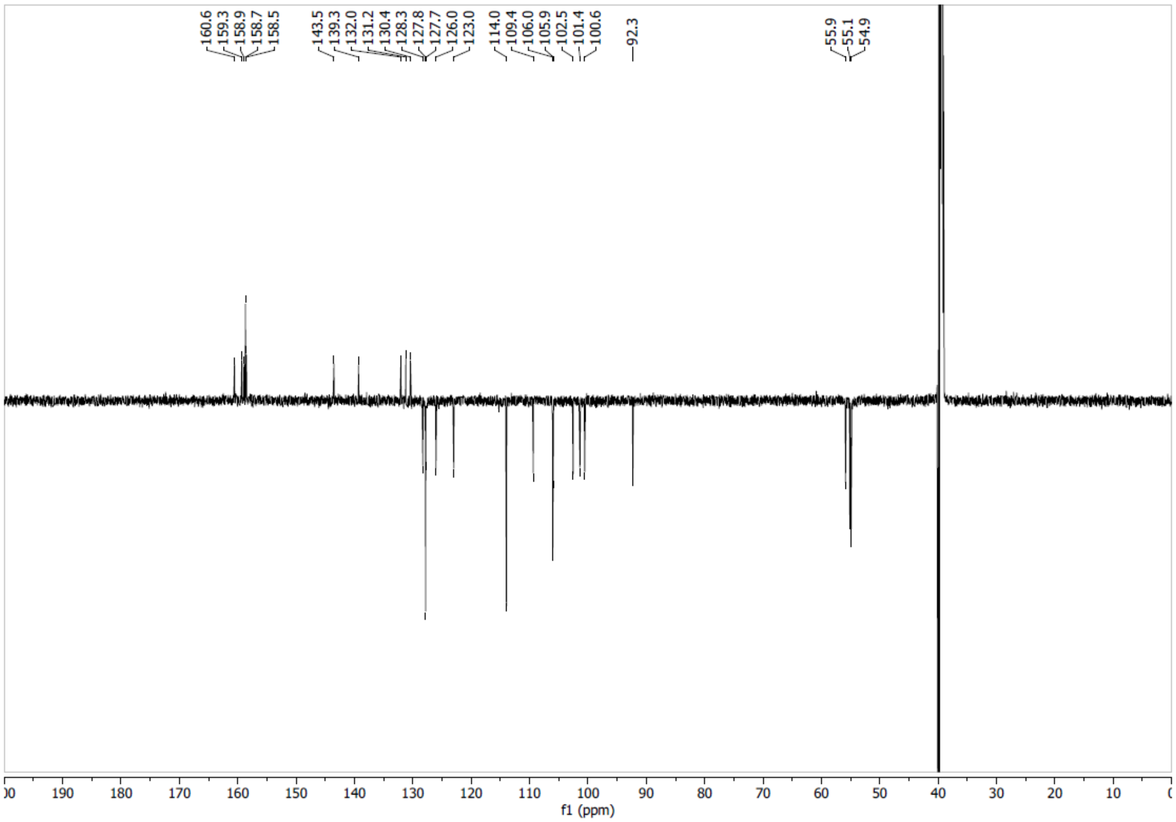

^{13}C-DEPTQ NMR spectrum of compound **14** in DMSO-d_6 at 151 MHz

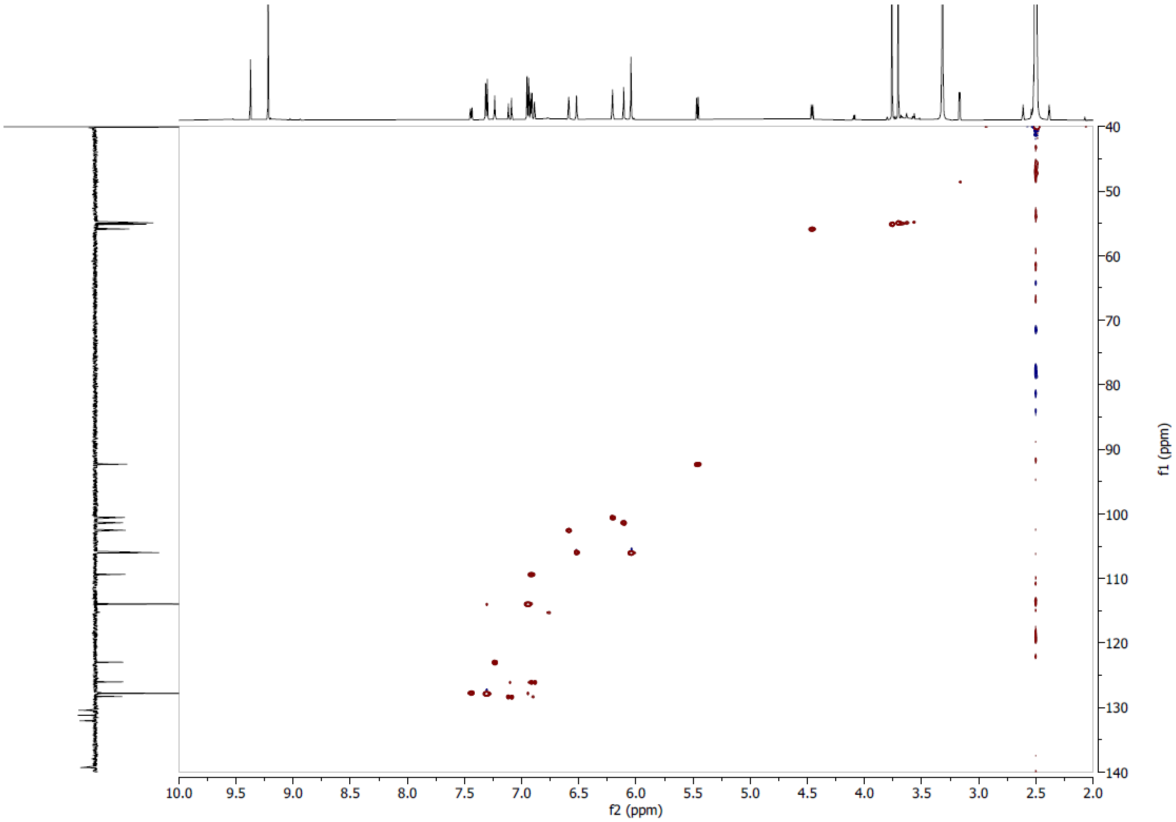

Edited HSQC NMR spectrum of compound **14** in DMSO-d_6

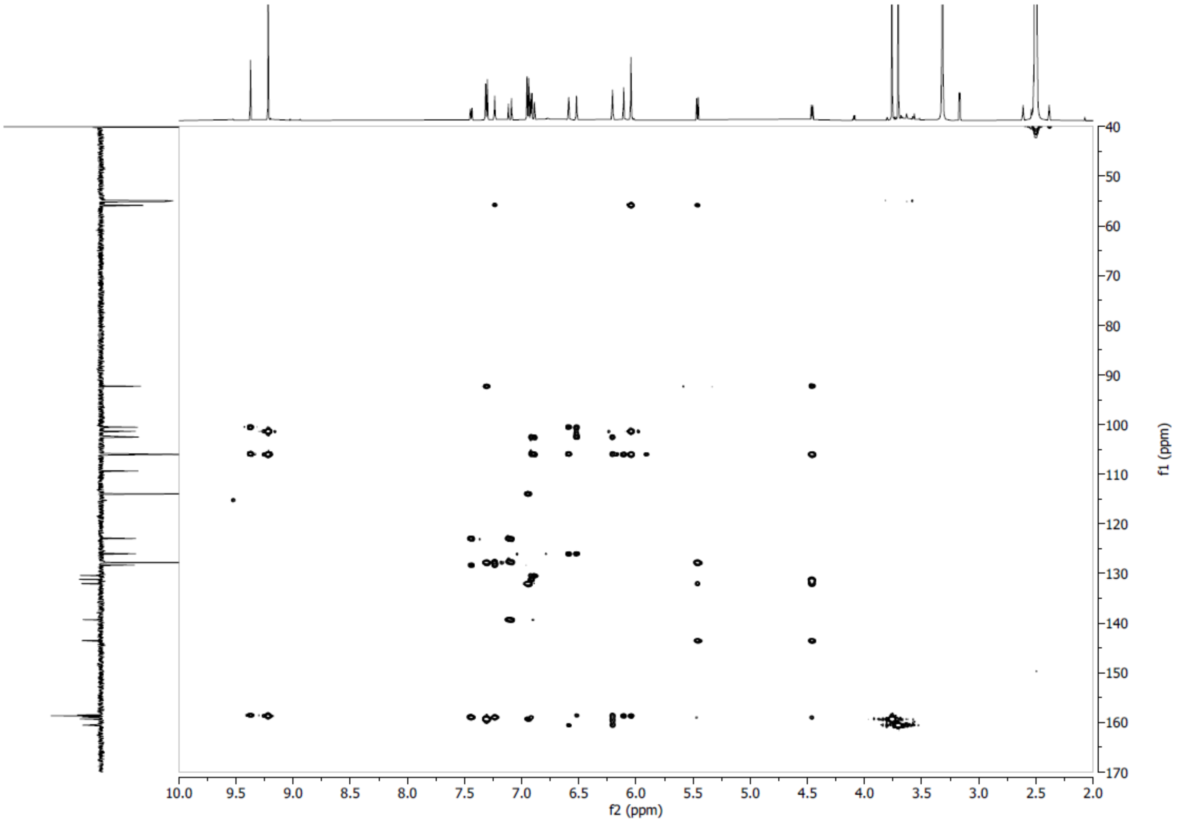

HMBC NMR spectrum of compound **14** in DMSO-d_6

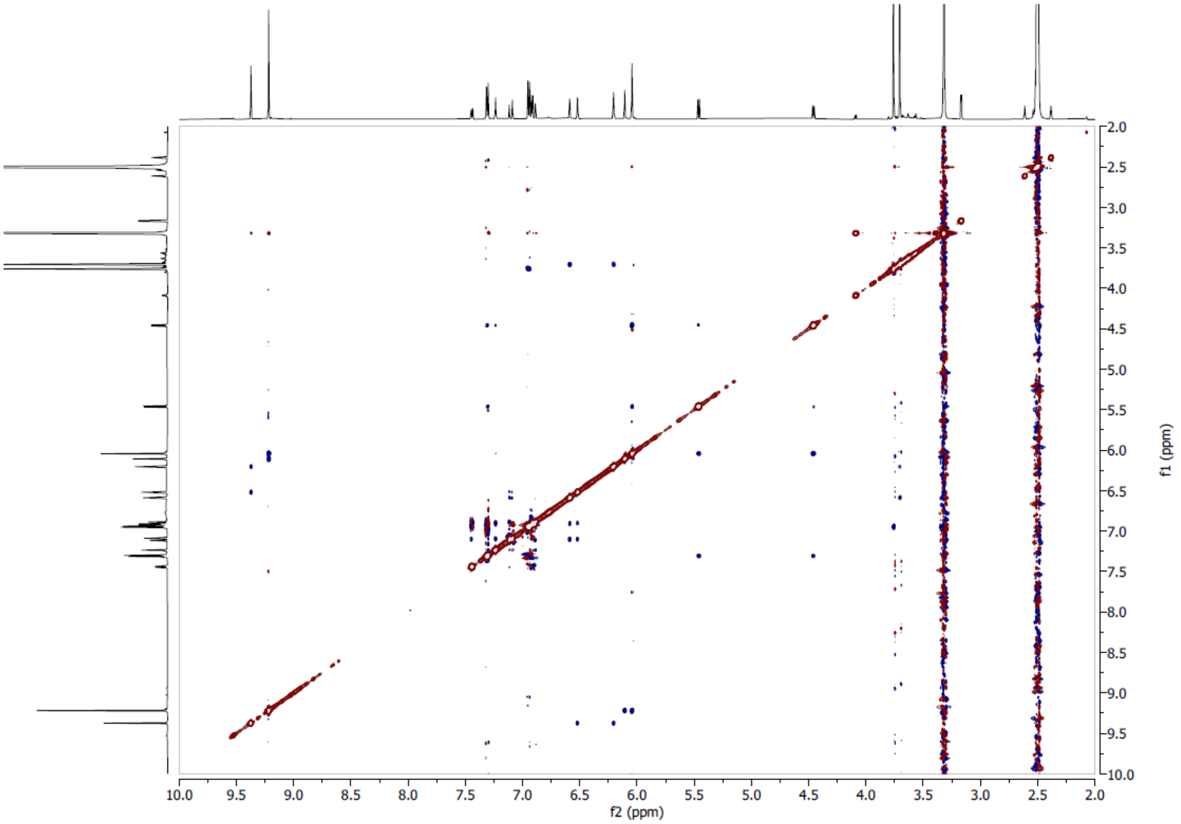

ROESY NMR spectrum of compound **14** in DMSO-d_6

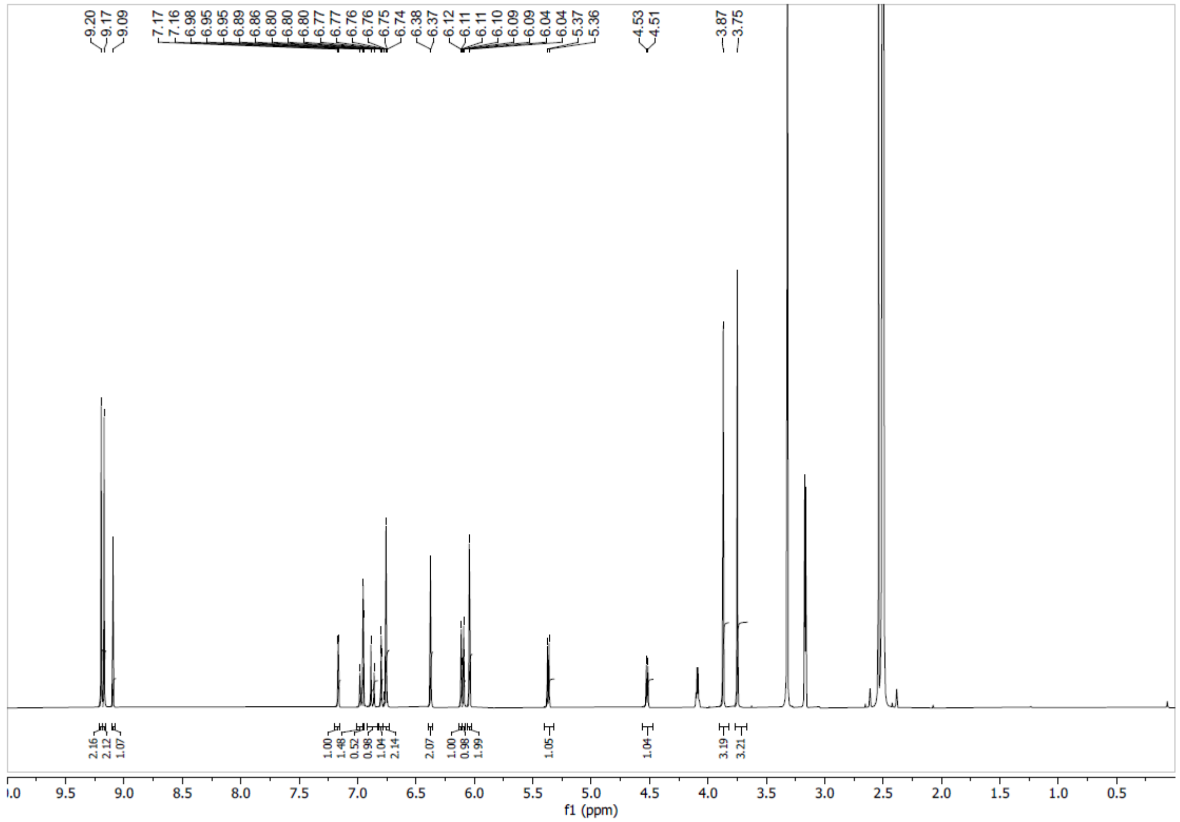

^{1}H NMR spectrum of compound **15** in DMSO-d_6 at 600 MHz

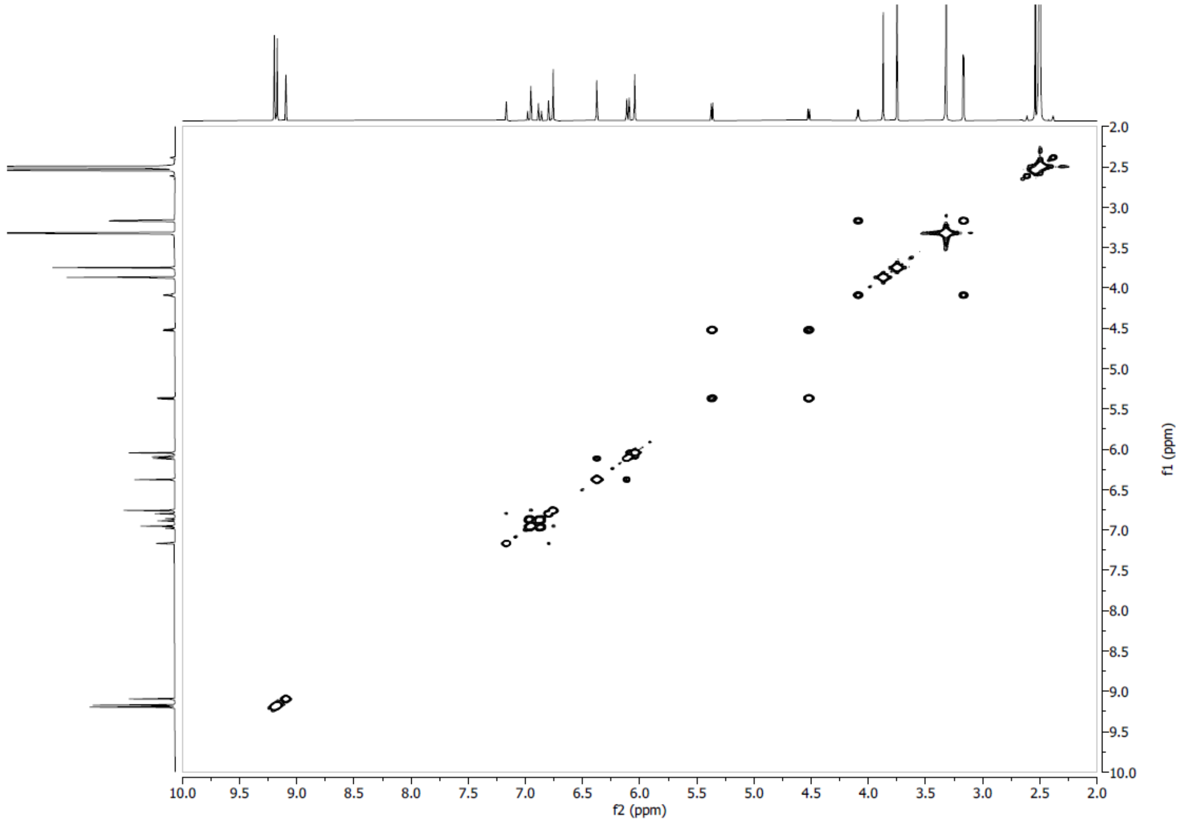

COSY NMR spectrum of compound **15** in DMSO-d_6

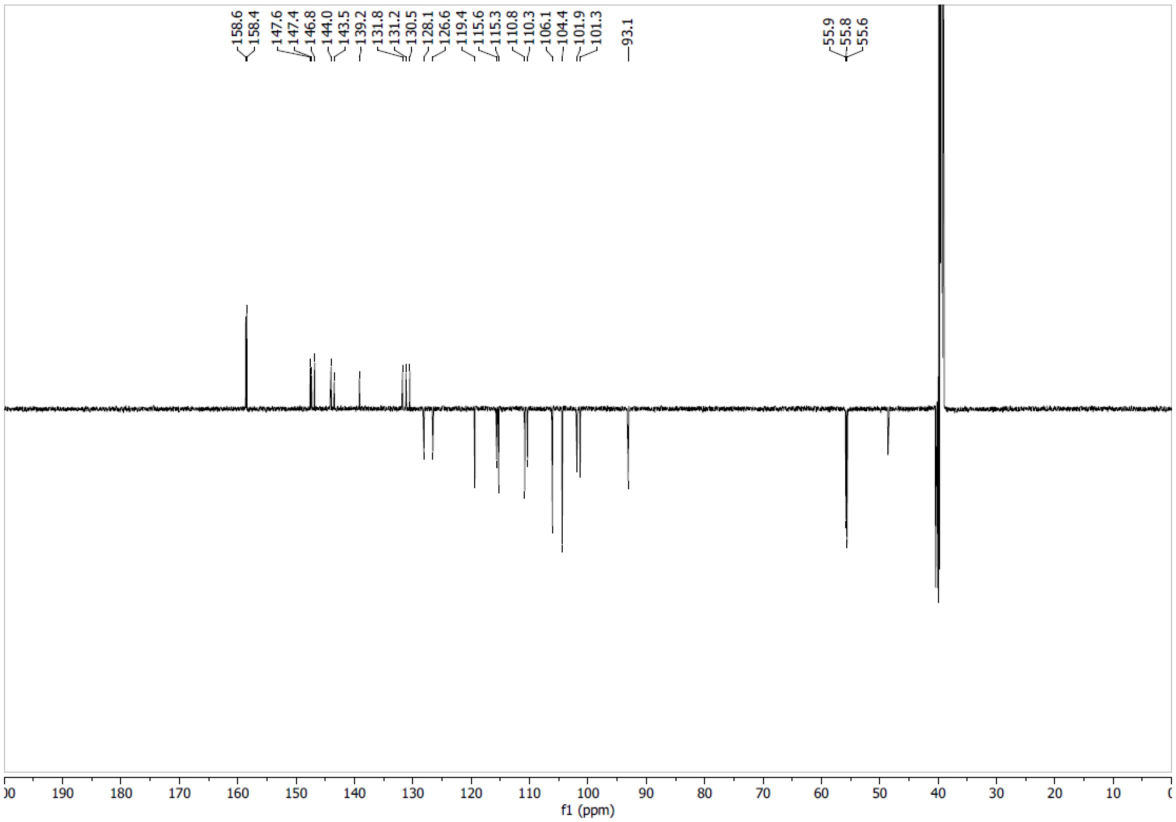

^{13}C-DEPTQ NMR spectrum of compound **15** in DMSO-d_6 at 151 MHz

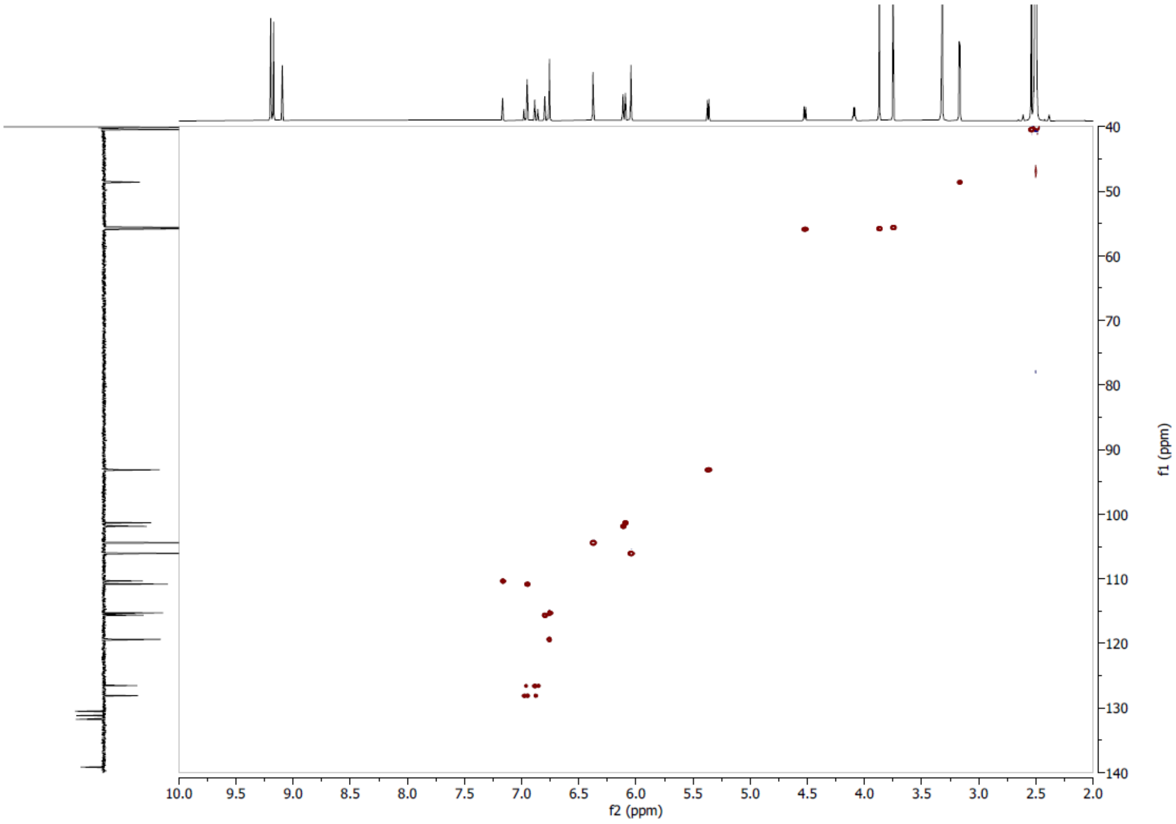

Edited HSQC NMR spectrum of compound **15** in DMSO-d_6

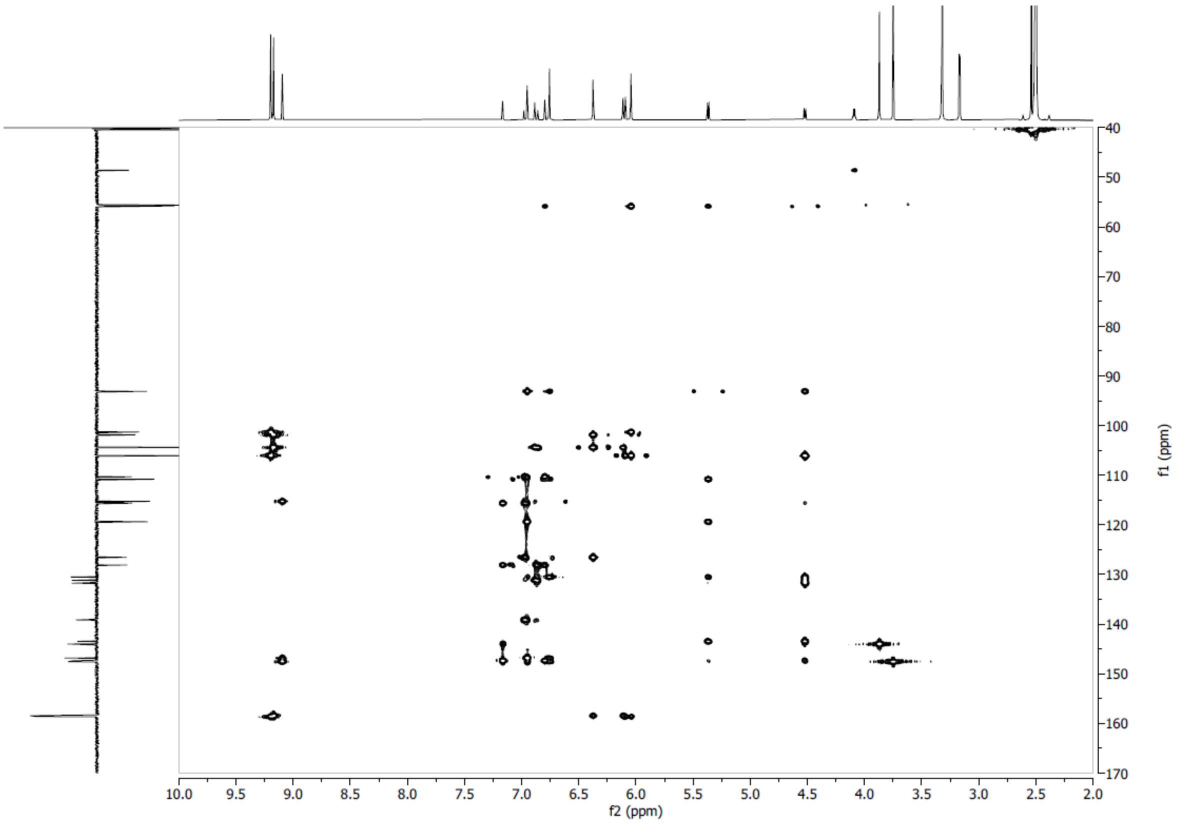

HMBC NMR spectrum of compound **15** in DMSO-d_6

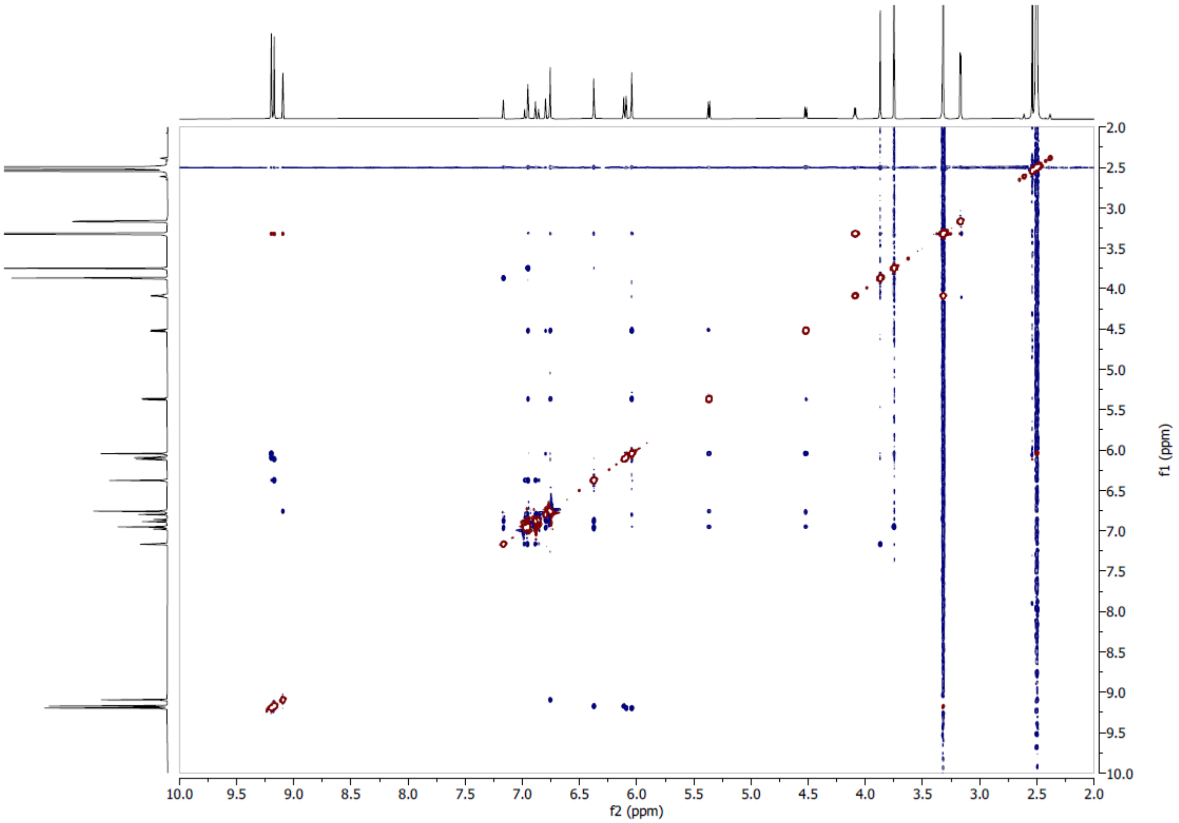

ROESY NMR spectrum of compound **15** in DMSO-d_6

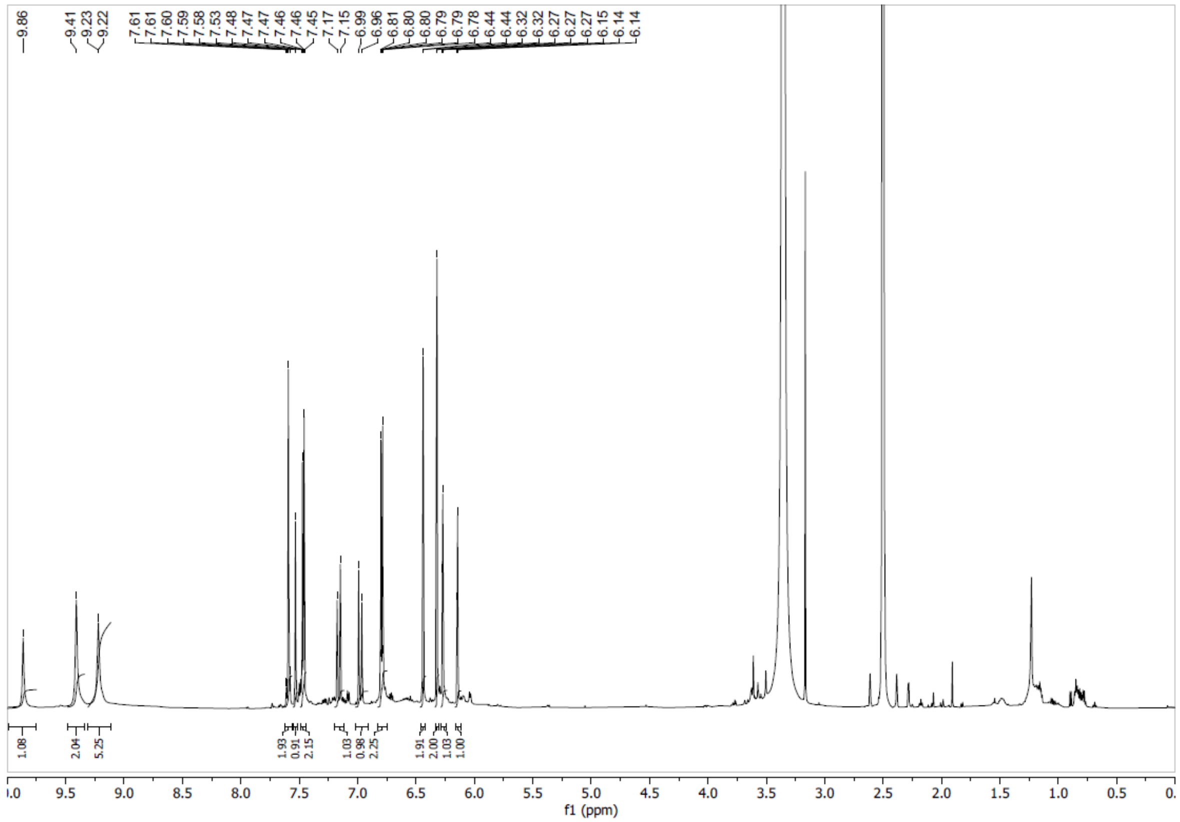

^{1}H NMR spectrum of compound **16** in DMSO-d_6 at 600 MHz

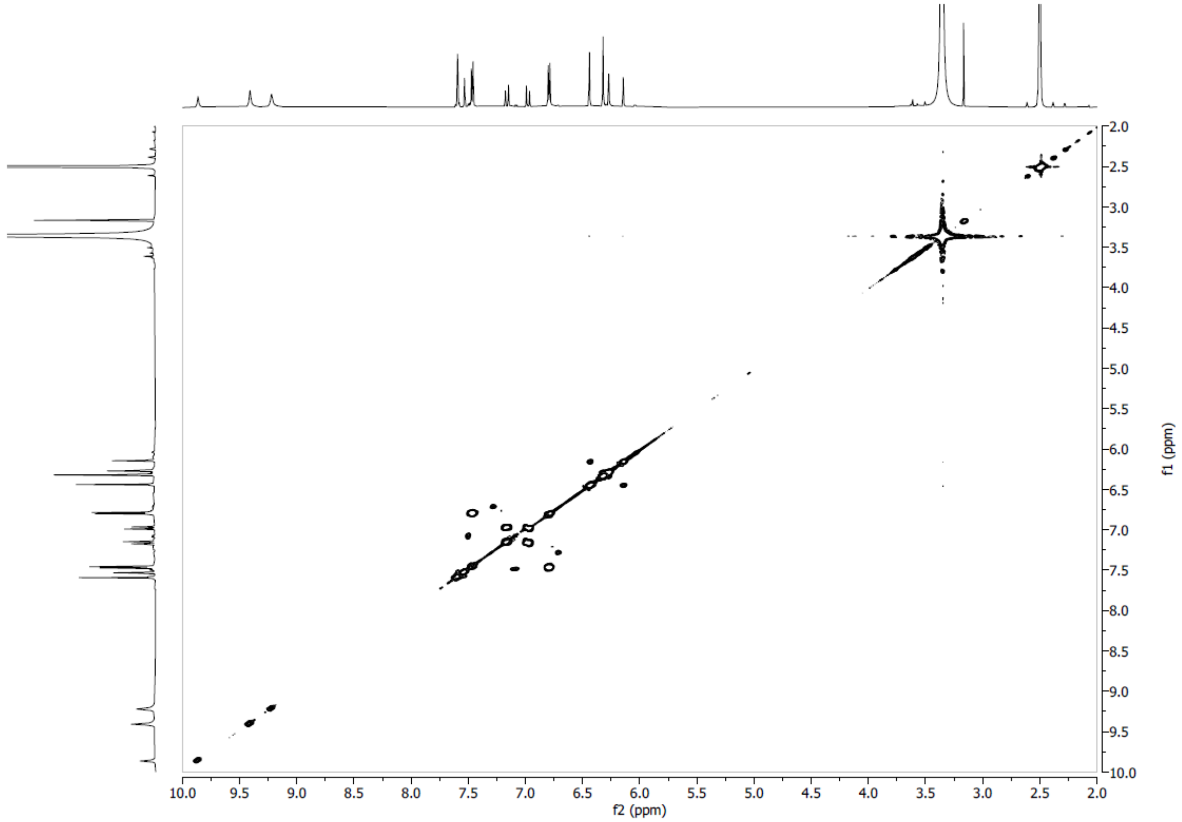

COSY NMR spectrum of compound **16** in DMSO-d_6

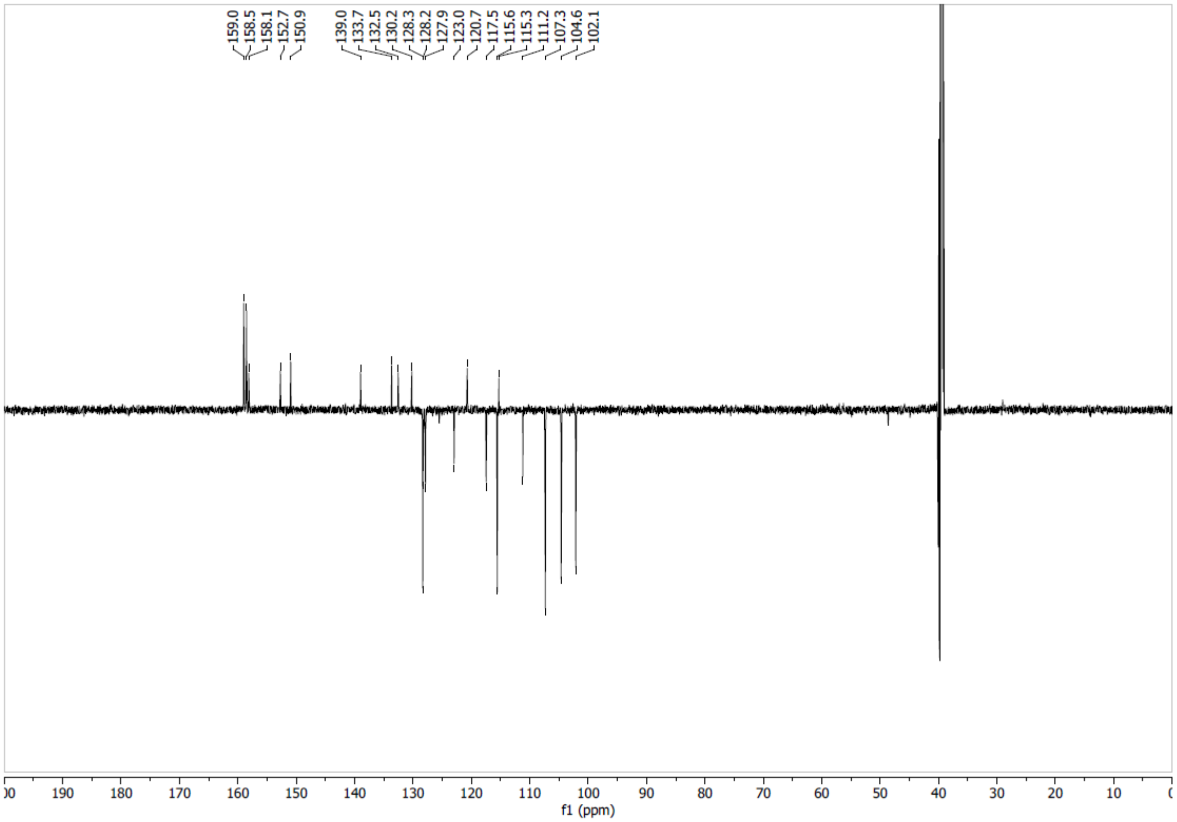

^{13}C-DEPTQ NMR spectrum of compound **16** in DMSO-d_6 at 151 MHz

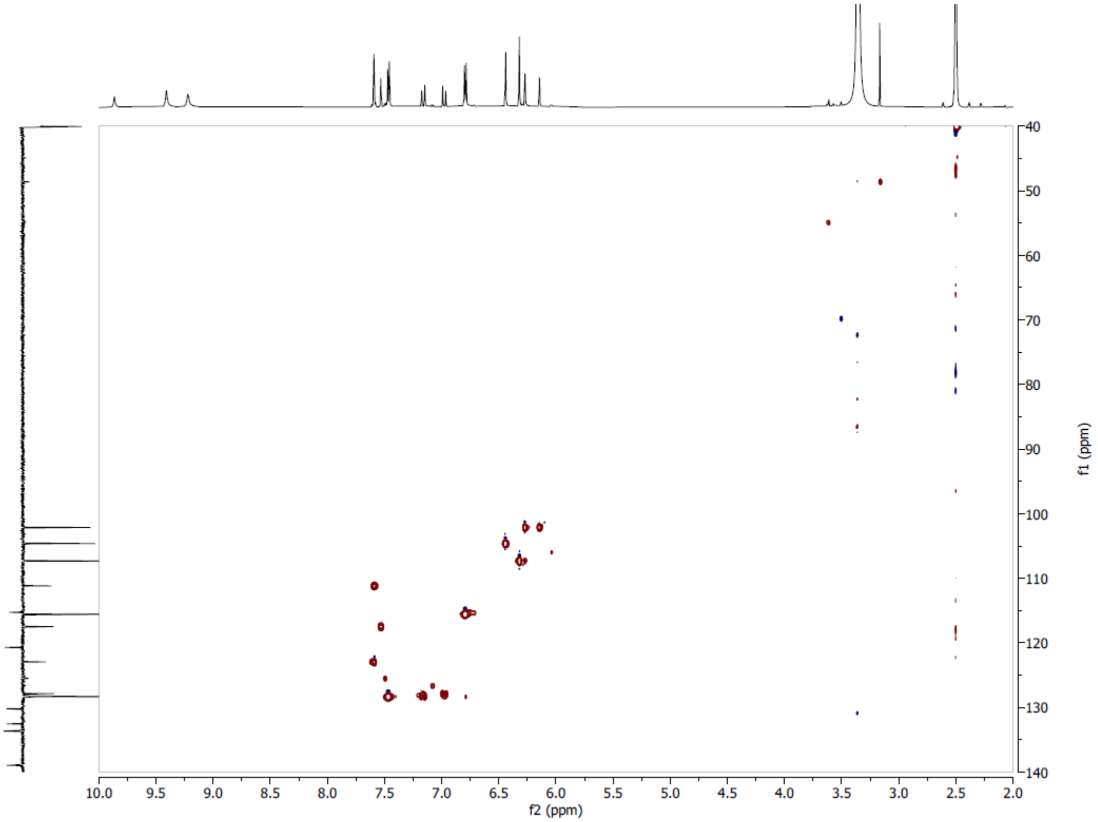

Edited HSQC NMR spectrum of compound **16** in DMSO-d_6

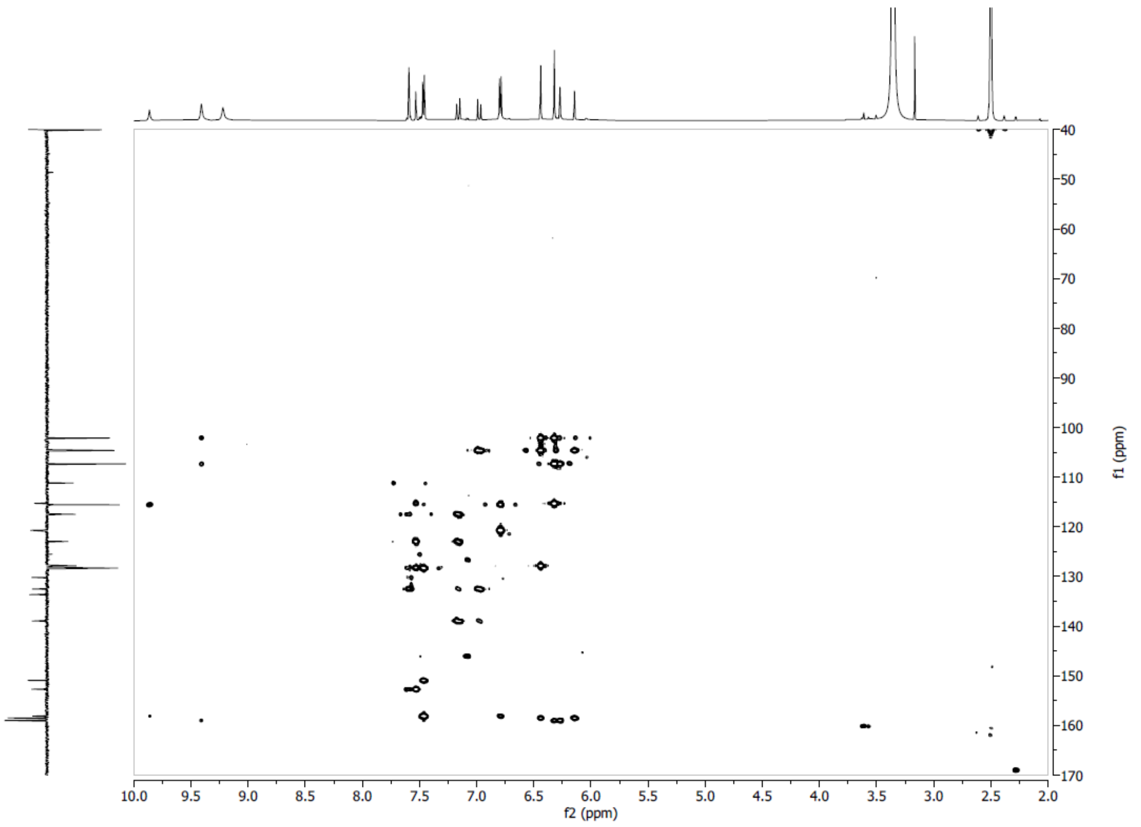

HMBC NMR spectrum of compound **16** in DMSO-d_6

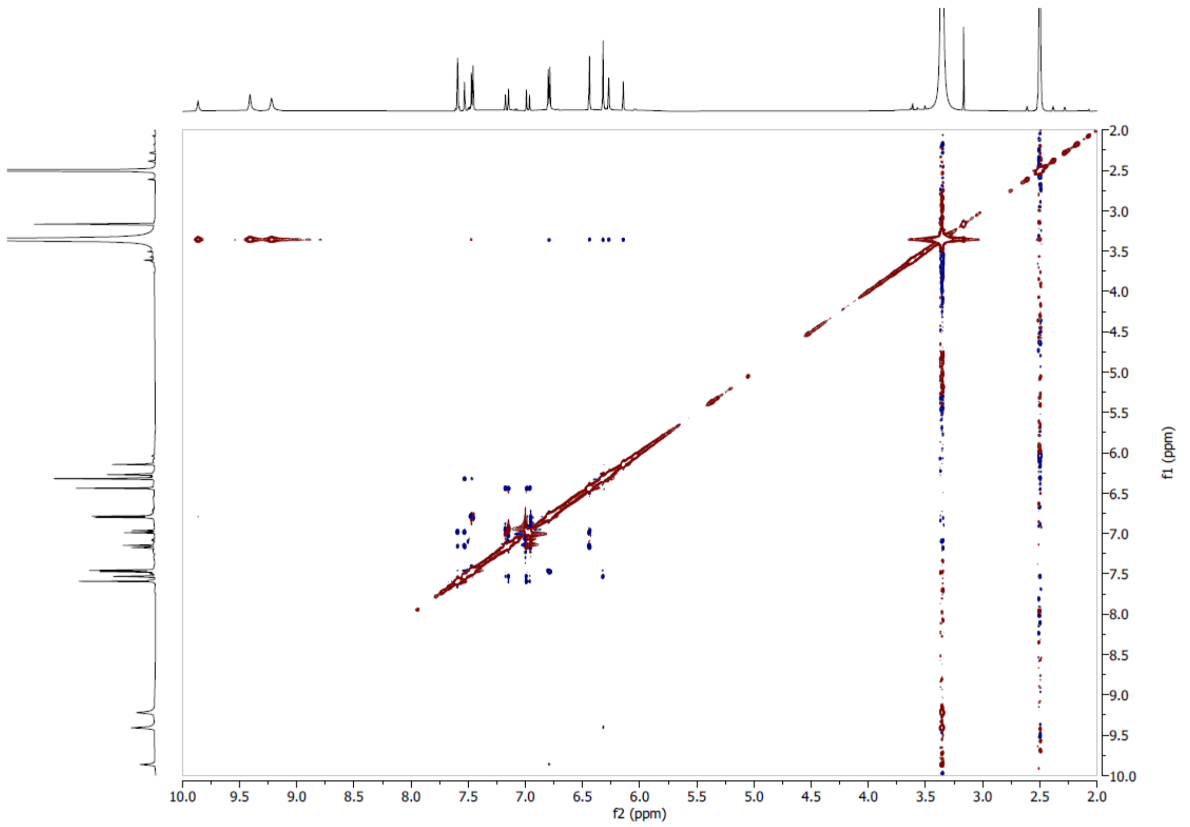

ROESY NMR spectrum of compound **16** in DMSO-d_6

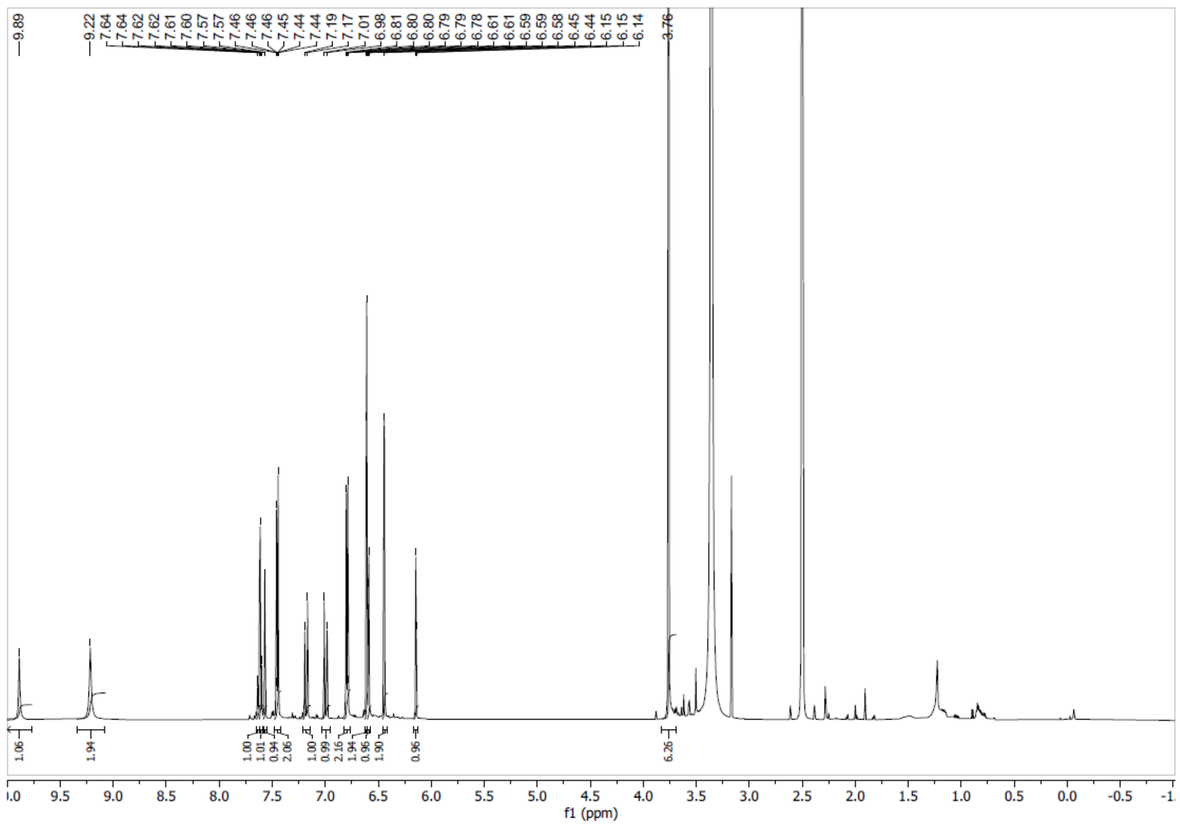

^{1}H NMR spectrum of compound **17** in DMSO-d_6 at 600 MHz

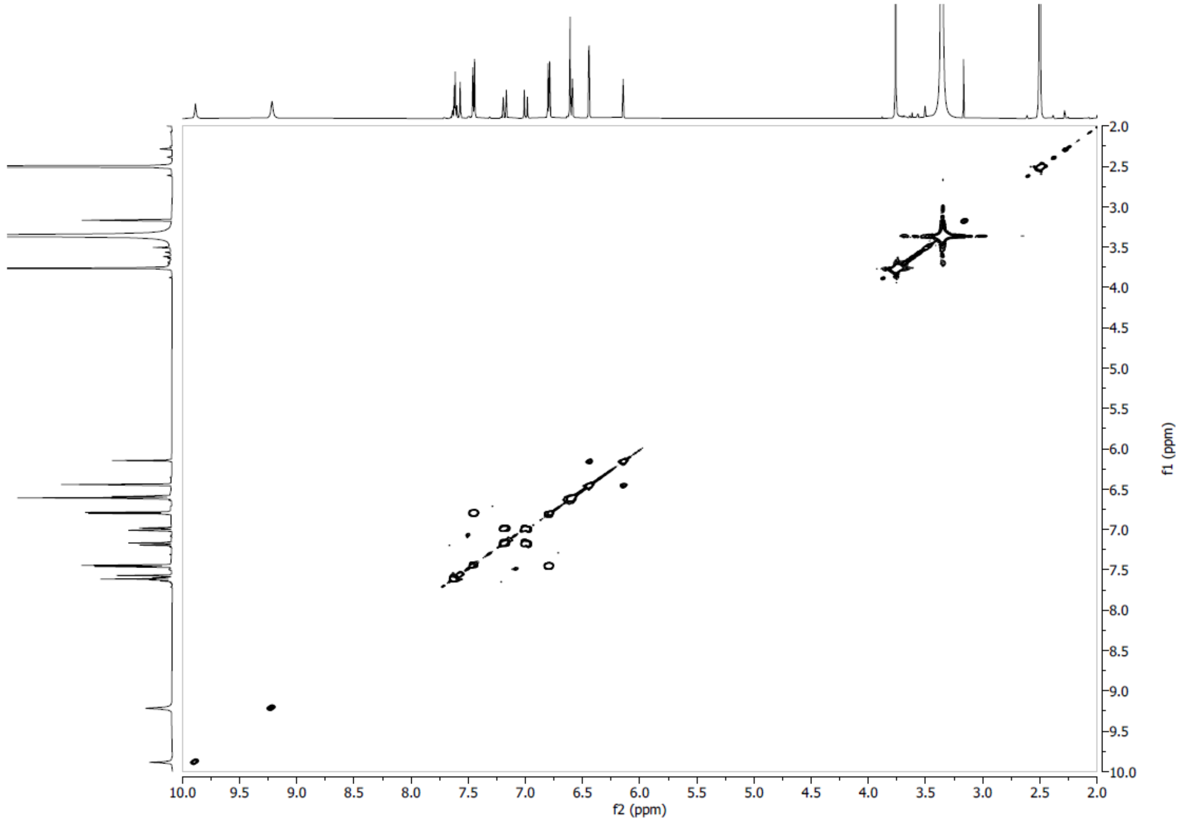

COSY NMR spectrum of compound **17** in DMSO-d_6

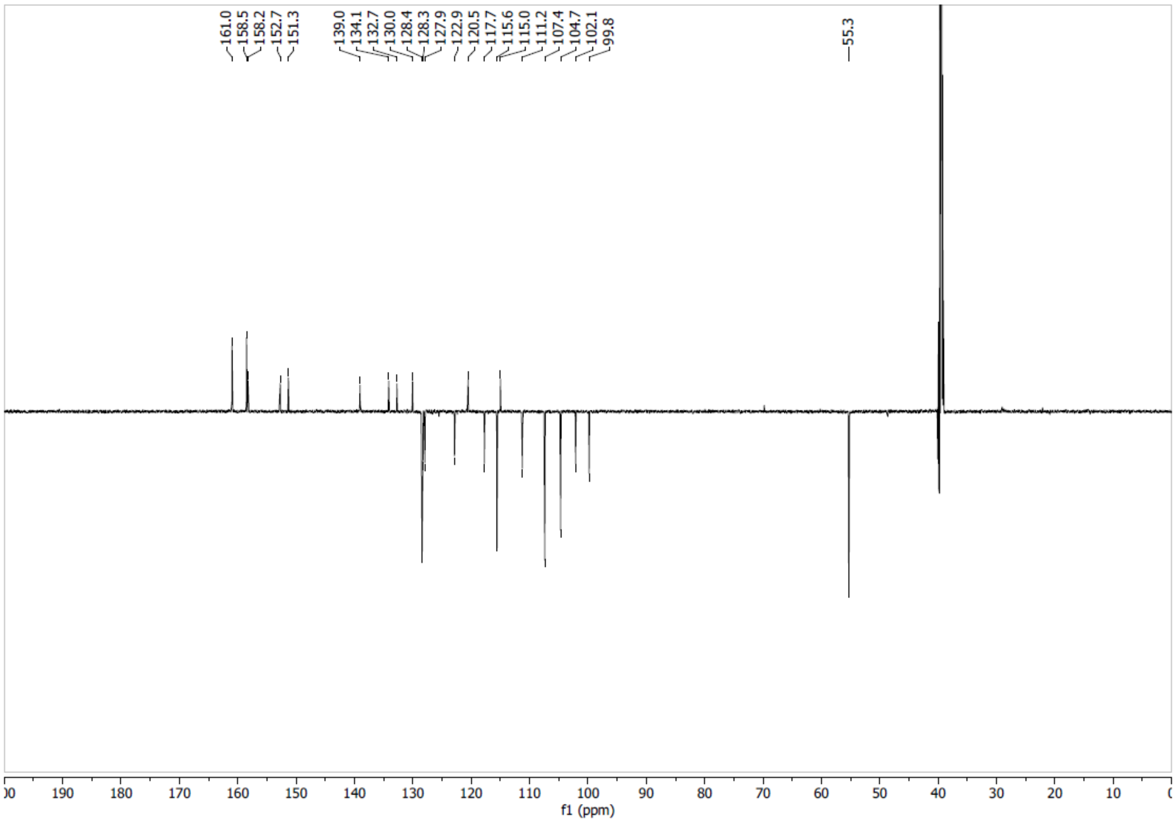

^{13}C-DEPTQ NMR spectrum of compound **17** in DMSO-d_6 at 151 MHz

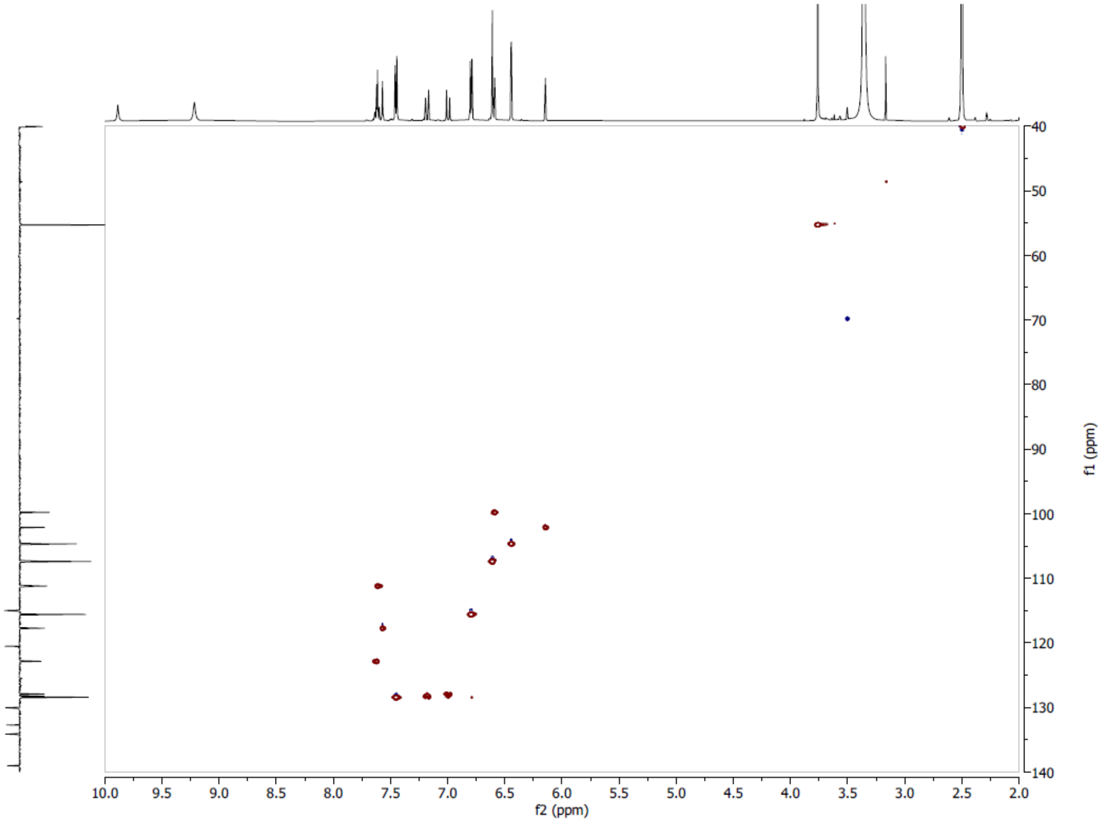

Edited HSQC NMR spectrum of compound **17** in DMSO-d_6

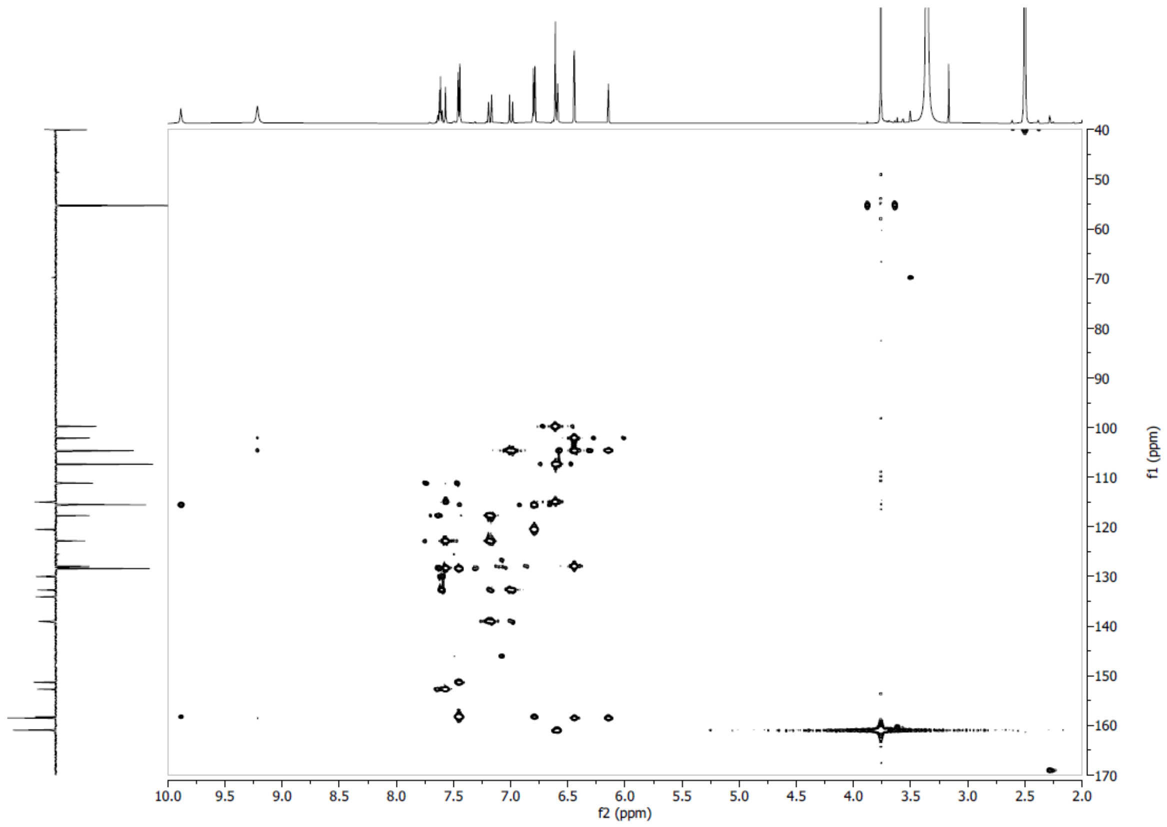

HMBC NMR spectrum of compound **17** in DMSO-d_6

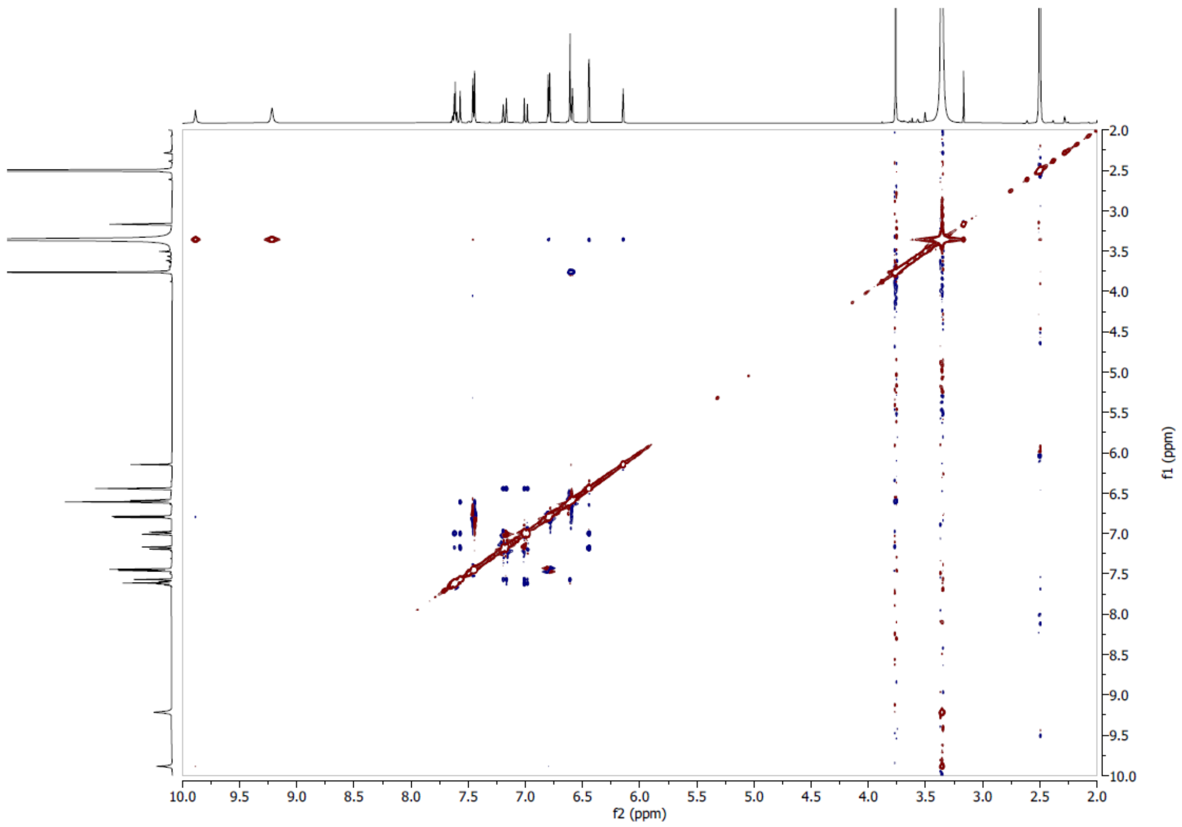

ROESY NMR spectrum of compound **17** in DMSO-d_6

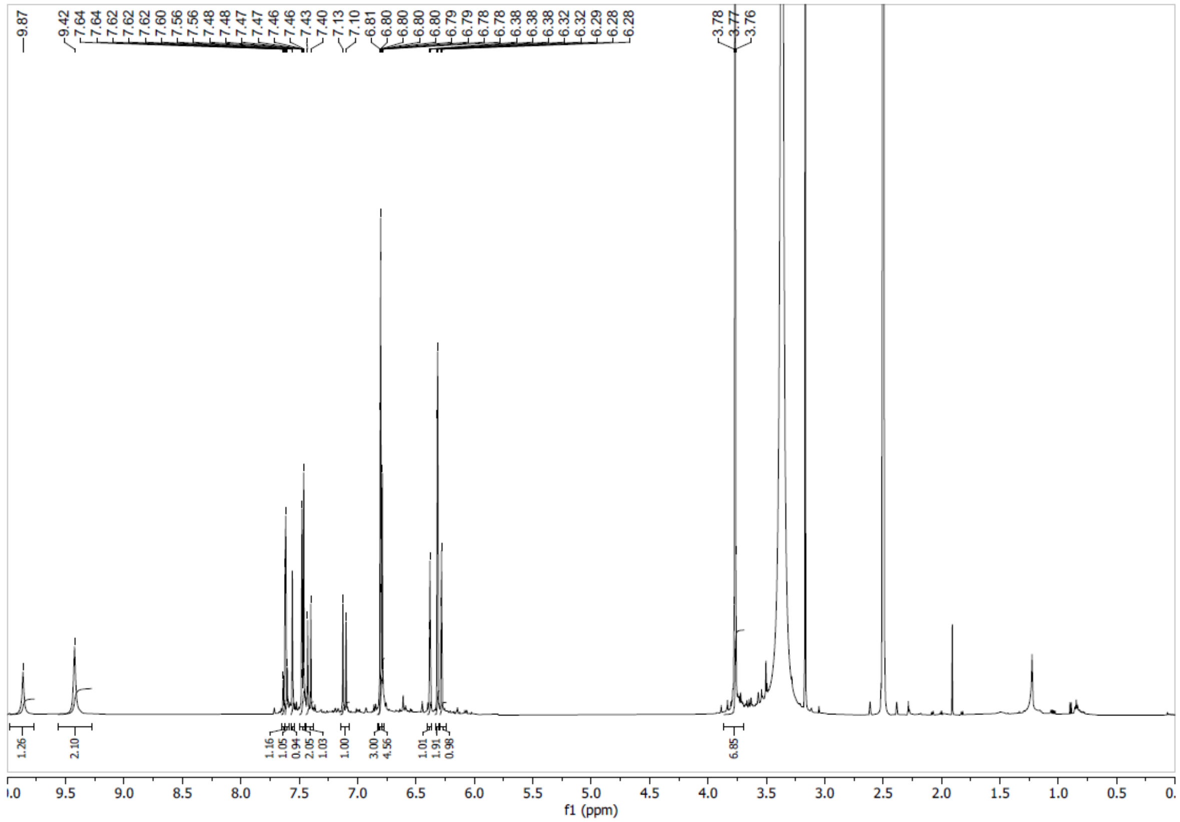

^{1}H NMR spectrum of compound **18** in DMSO-d_6 at 600 MHz

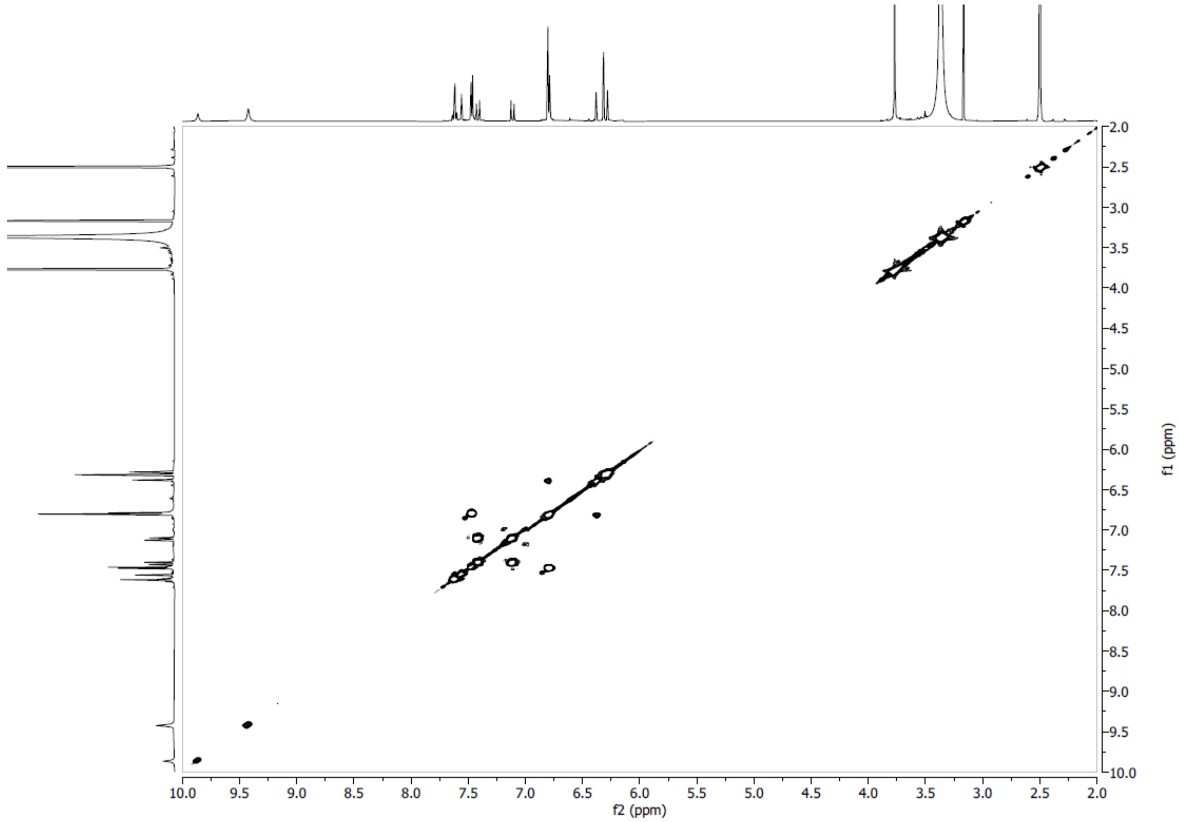

COSY NMR spectrum of compound **18** in DMSO-d_6

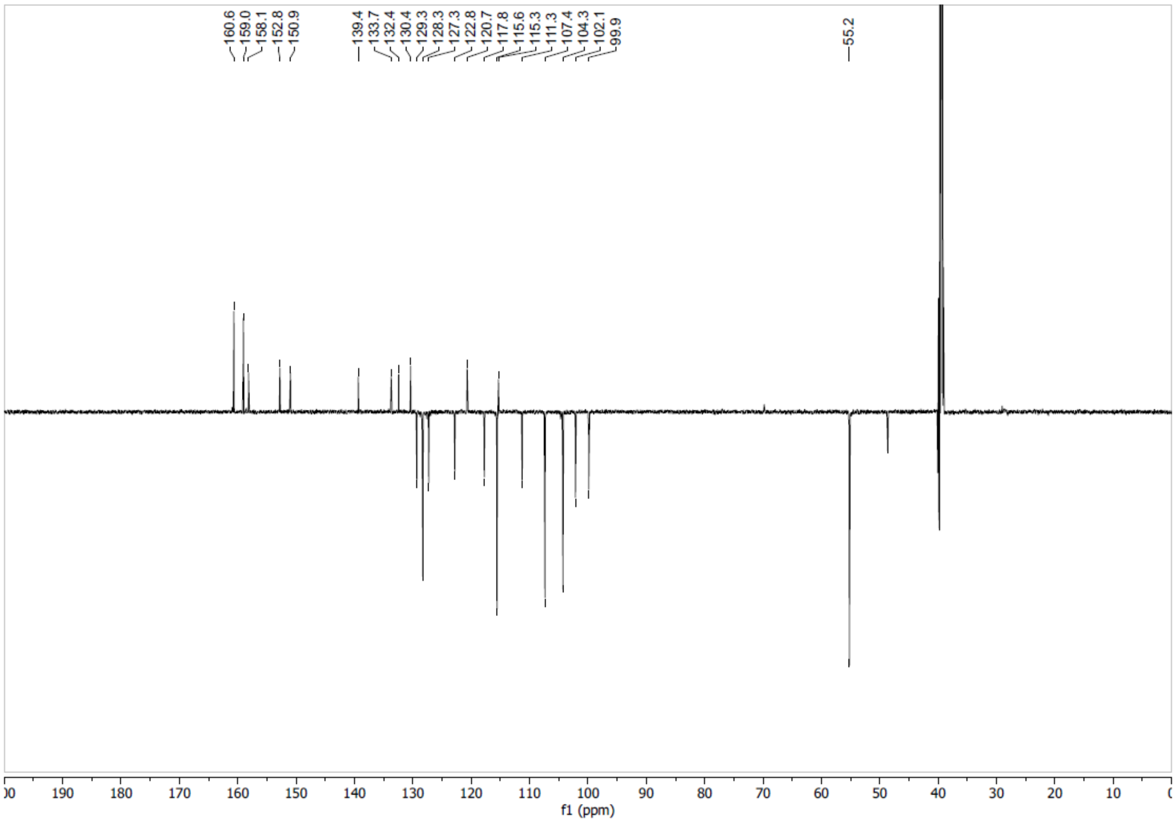

^{13}C-DEPTQ NMR spectrum of compound **18** in DMSO-d_6 at 151 MHz

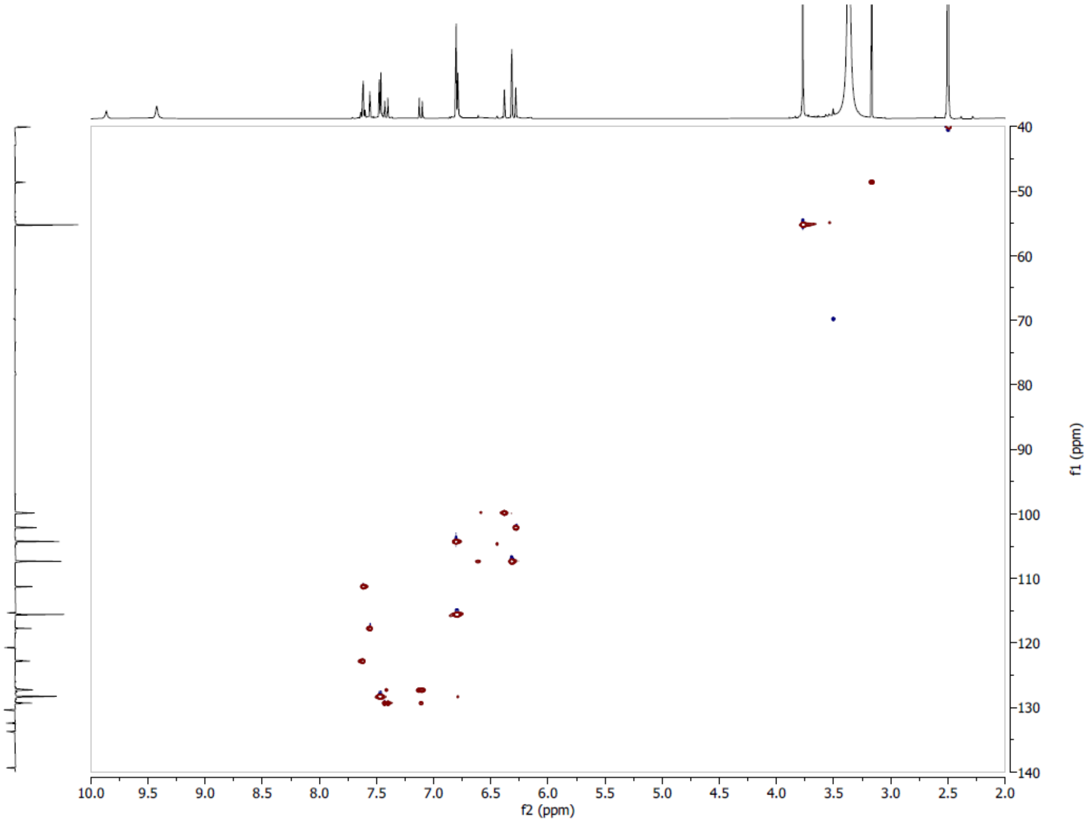

Edited HSQC NMR spectrum of compound **18** in DMSO-d_6

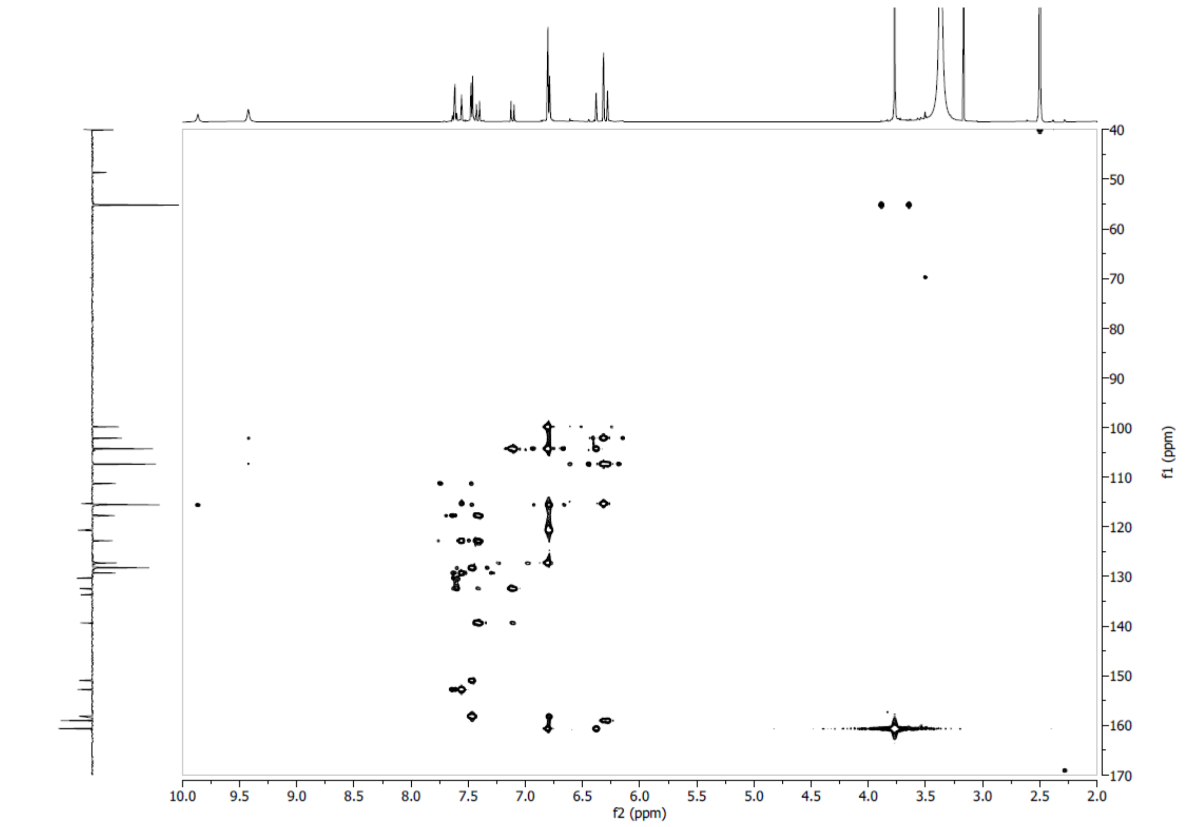

HMBC NMR spectrum of compound **18** in DMSO-d_6

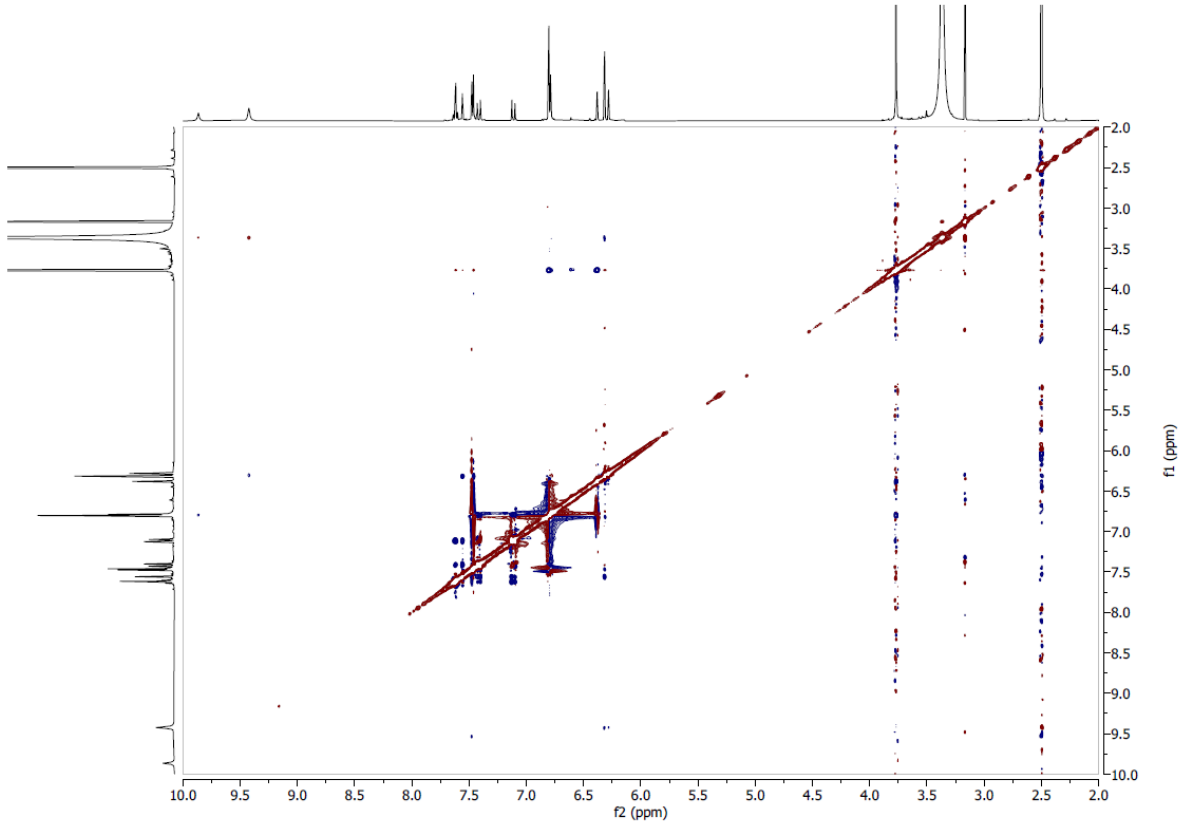

ROESY NMR spectrum of compound **18** in DMSO-d_6

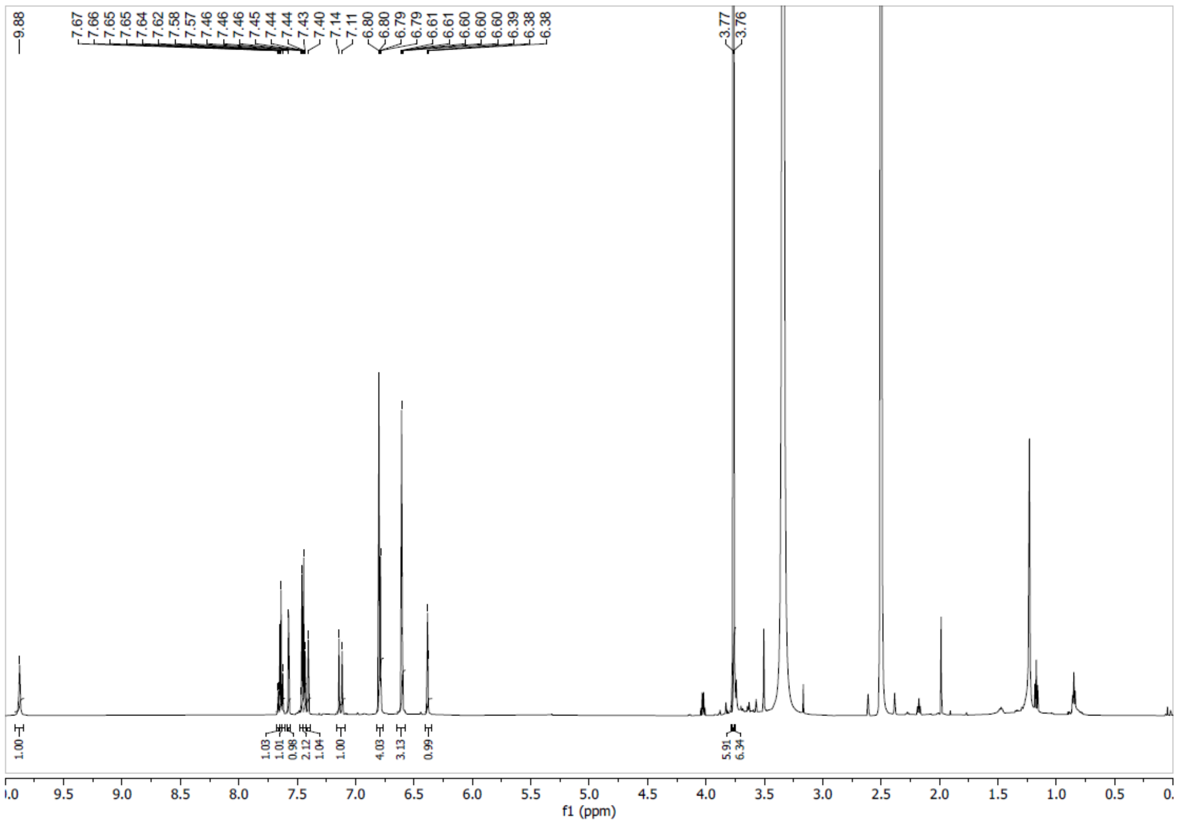

^{1}H NMR spectrum of compound **19** in DMSO-d_6 at 600 MHz

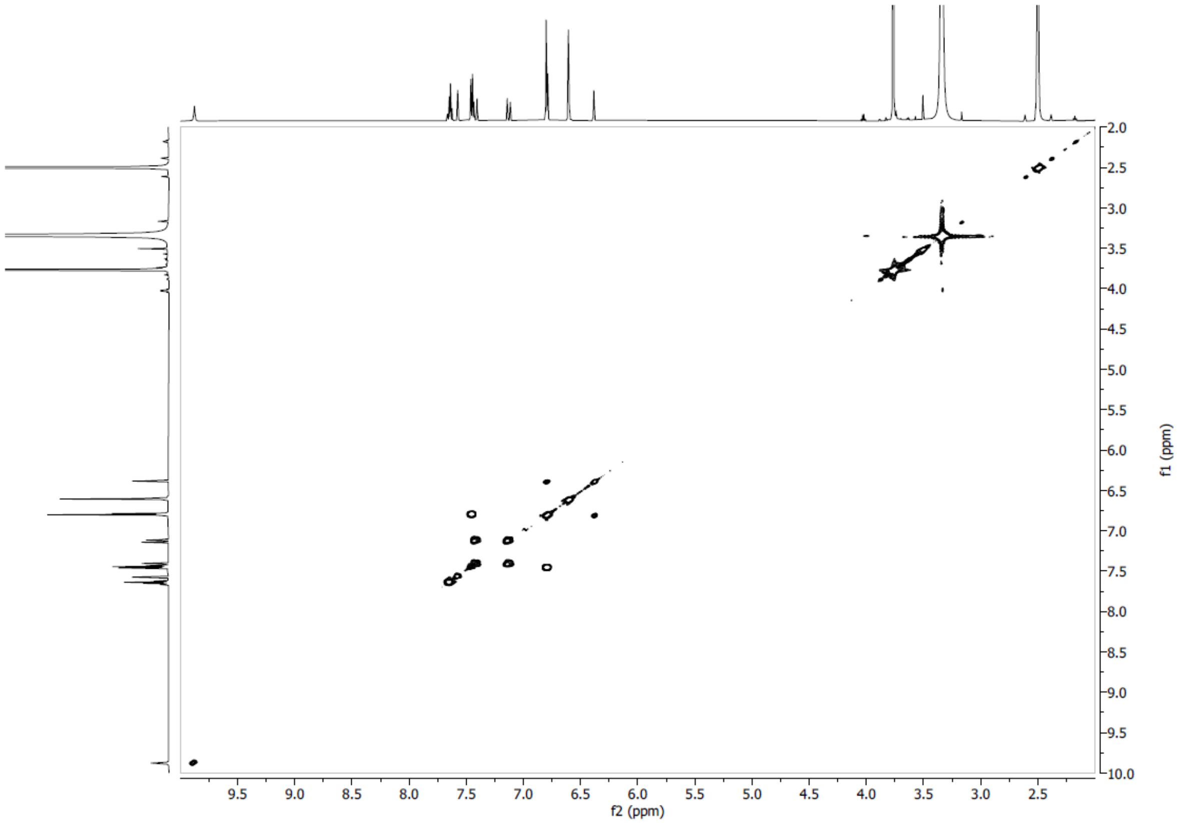

COSY NMR spectrum of compound **19** in DMSO-d_6

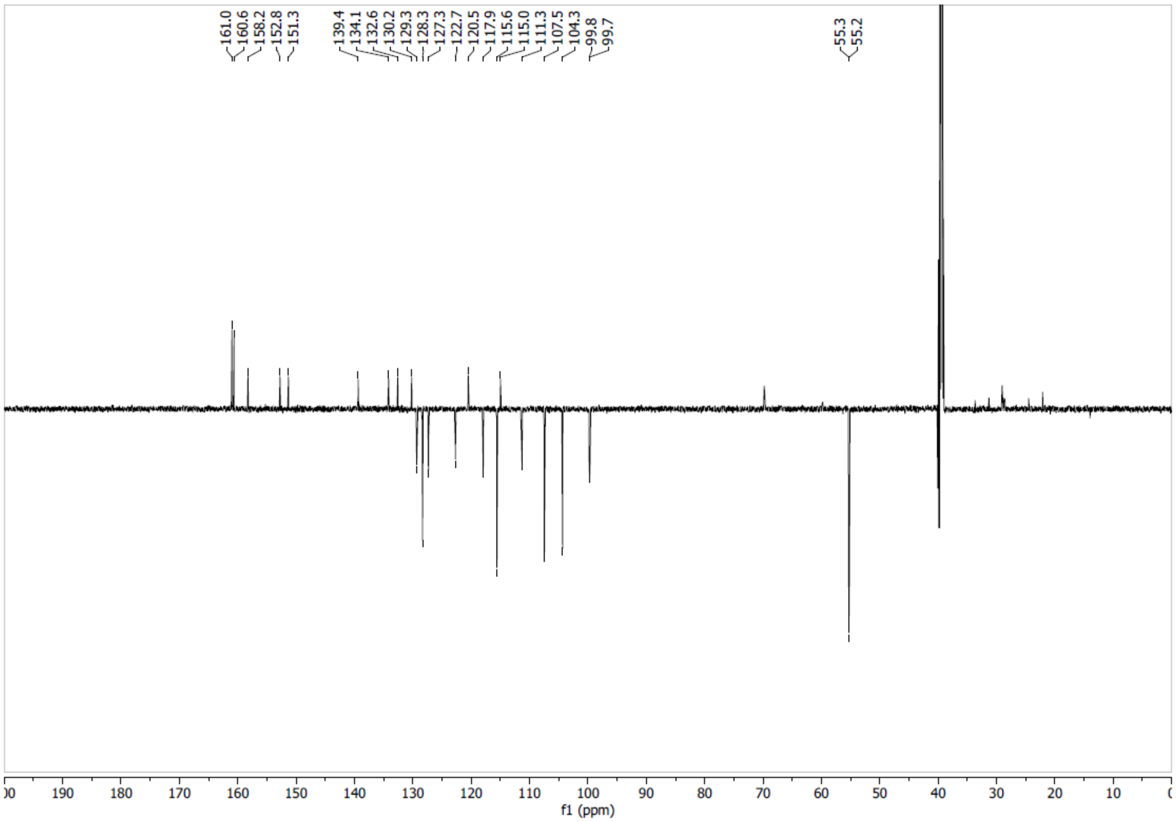

^{13}C-DEPTQ NMR spectrum of compound **19** in DMSO-d_6 at 151 MHz

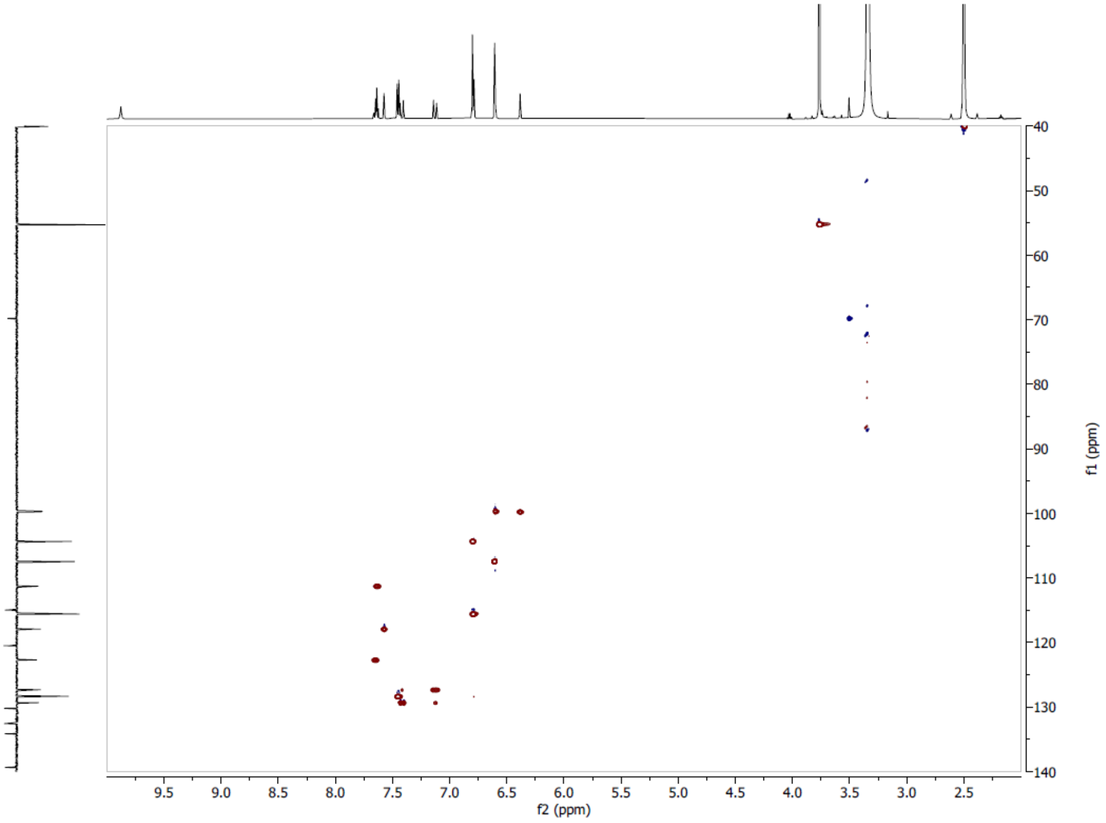

Edited HSQC NMR spectrum of compound **19** in DMSO-d_6

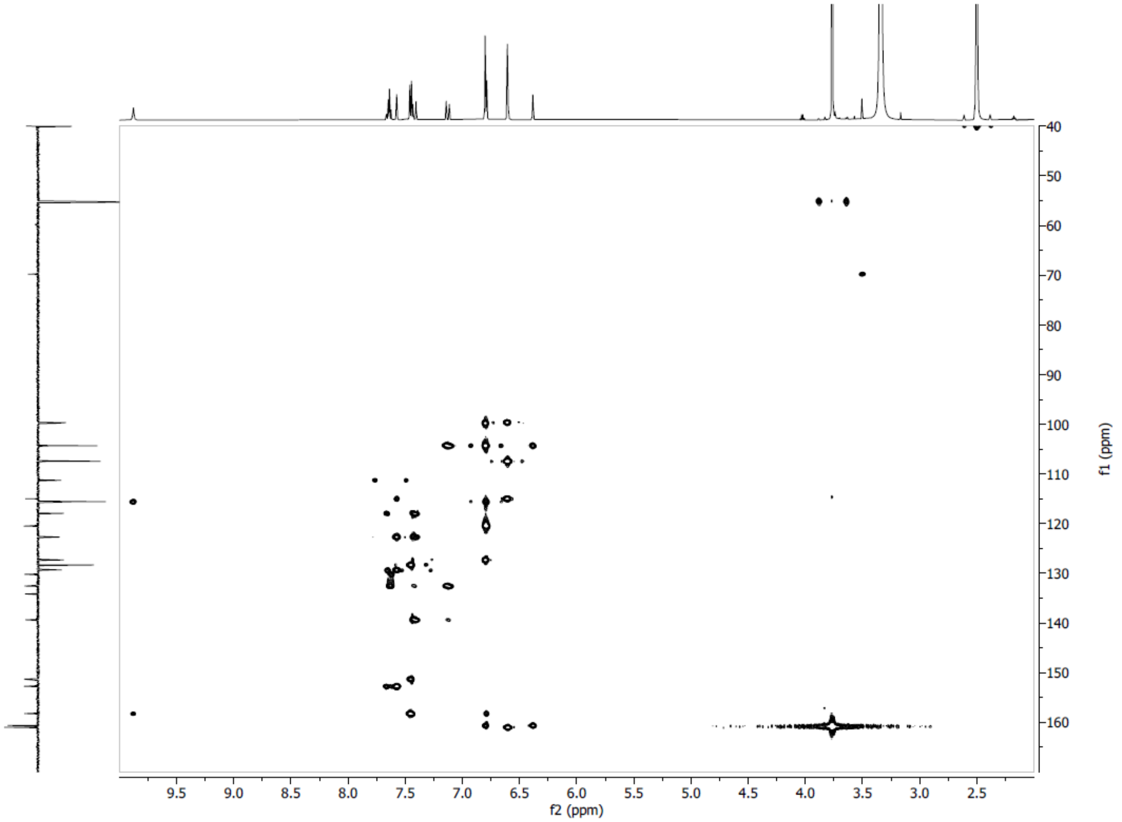

HMBC NMR spectrum of compound **19** in DMSO-*d*6

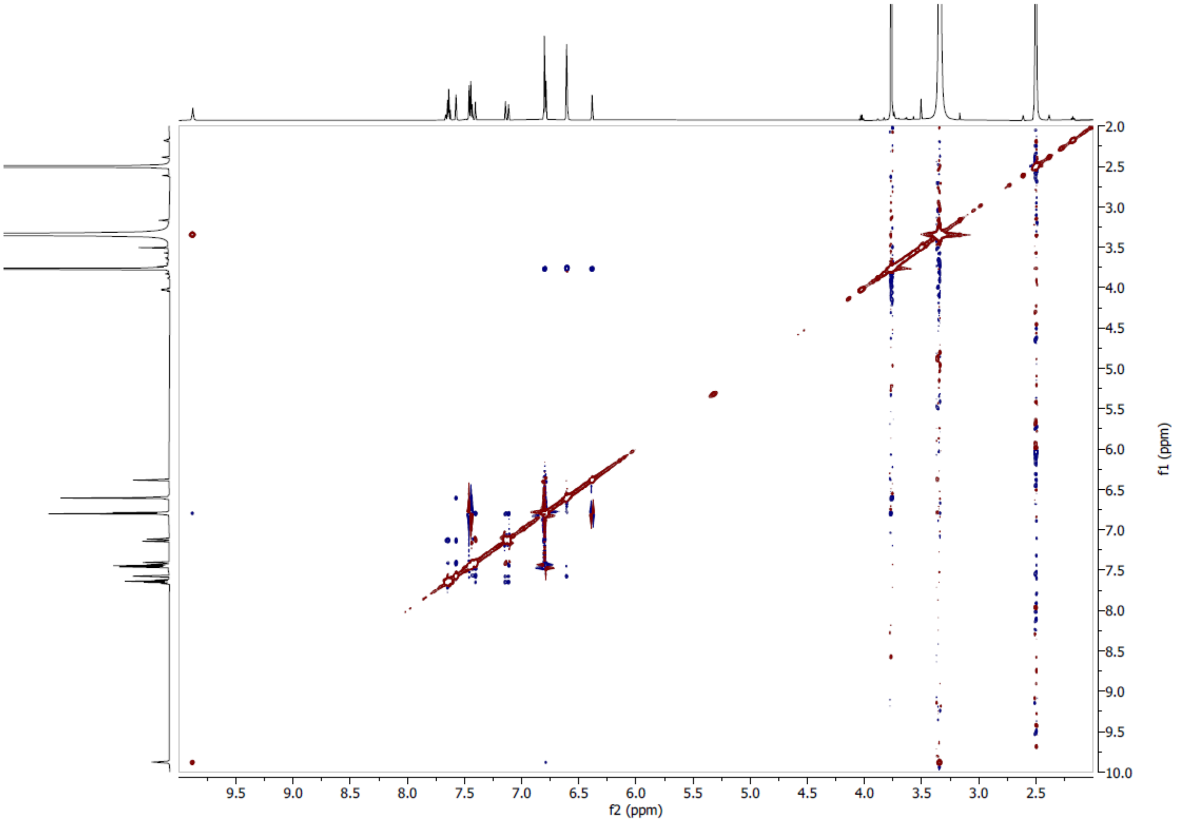

ROESY NMR spectrum of compound **19** in DMSO-*d*6

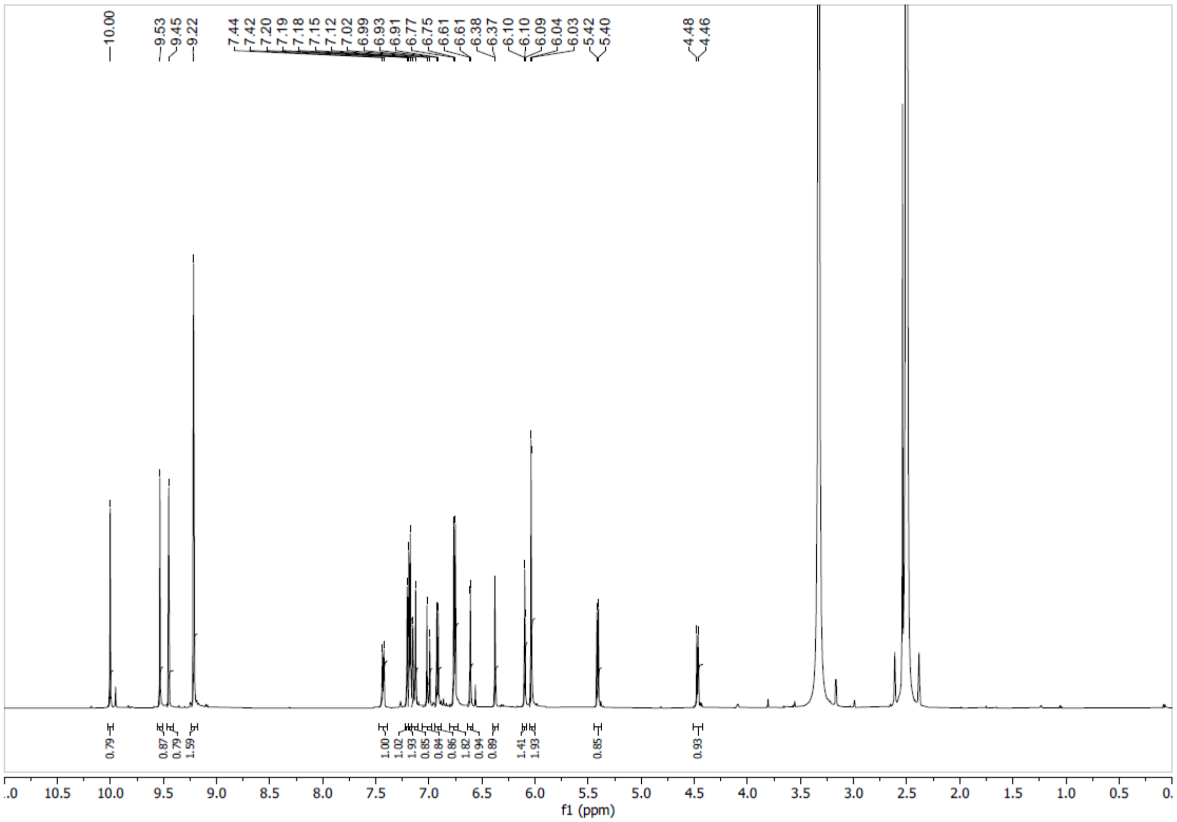

^{1}H NMR spectrum of compound **20** in DMSO-d_6 at 600 MHz

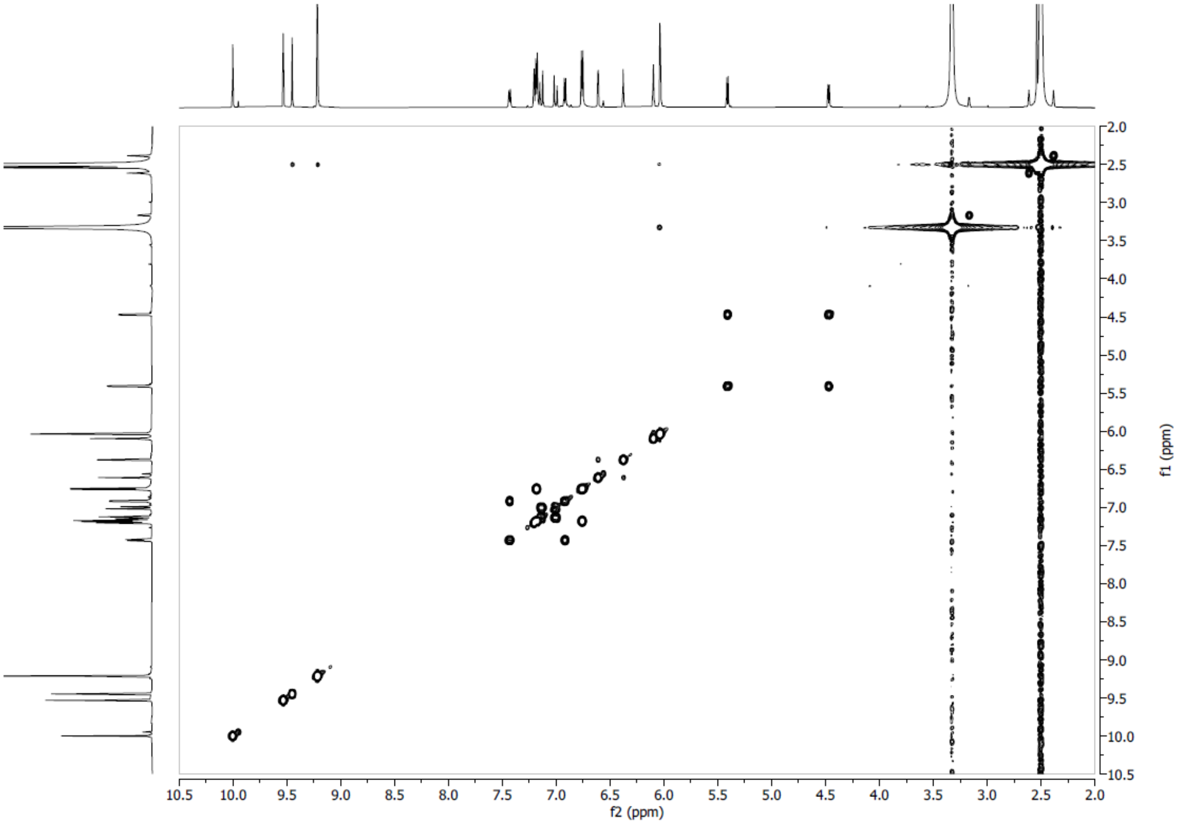

COSY NMR spectrum of compound **20** in DMSO-d_6

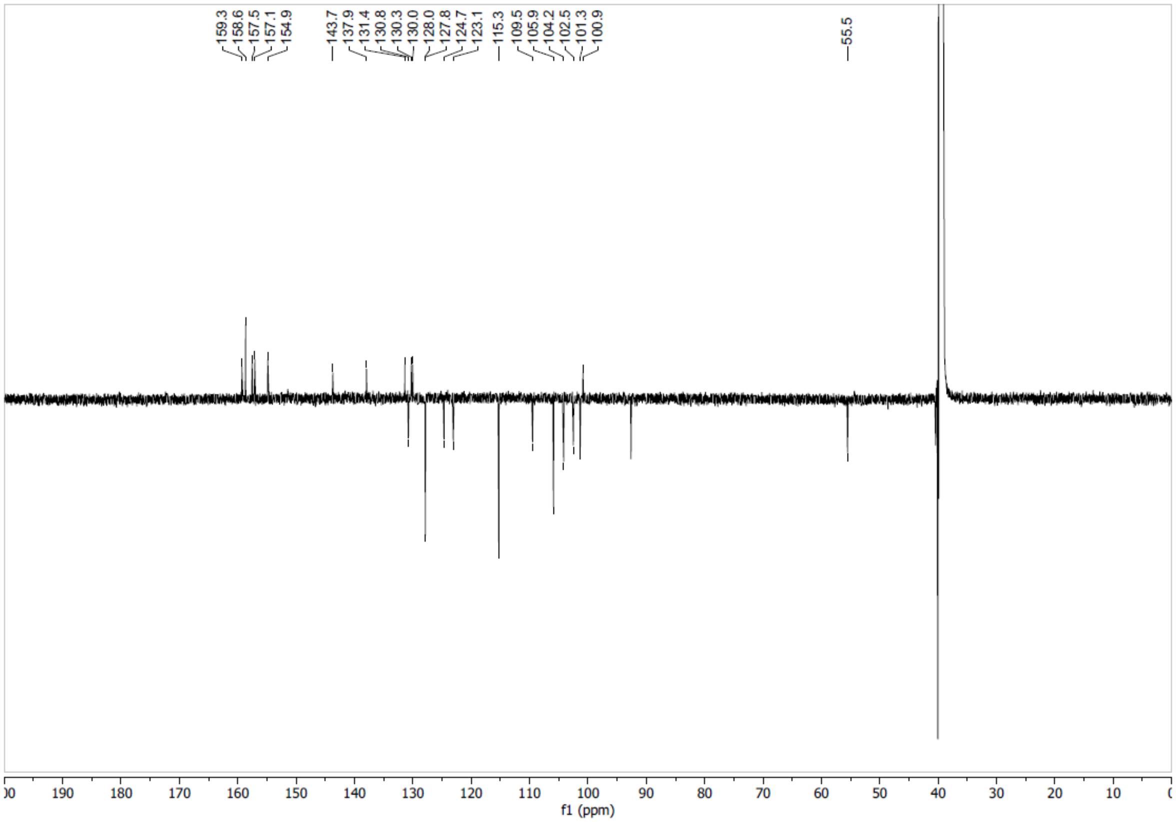

^{13}C-DEPTQ NMR spectrum of compound **20** in DMSO-d_6 at 151 MHz

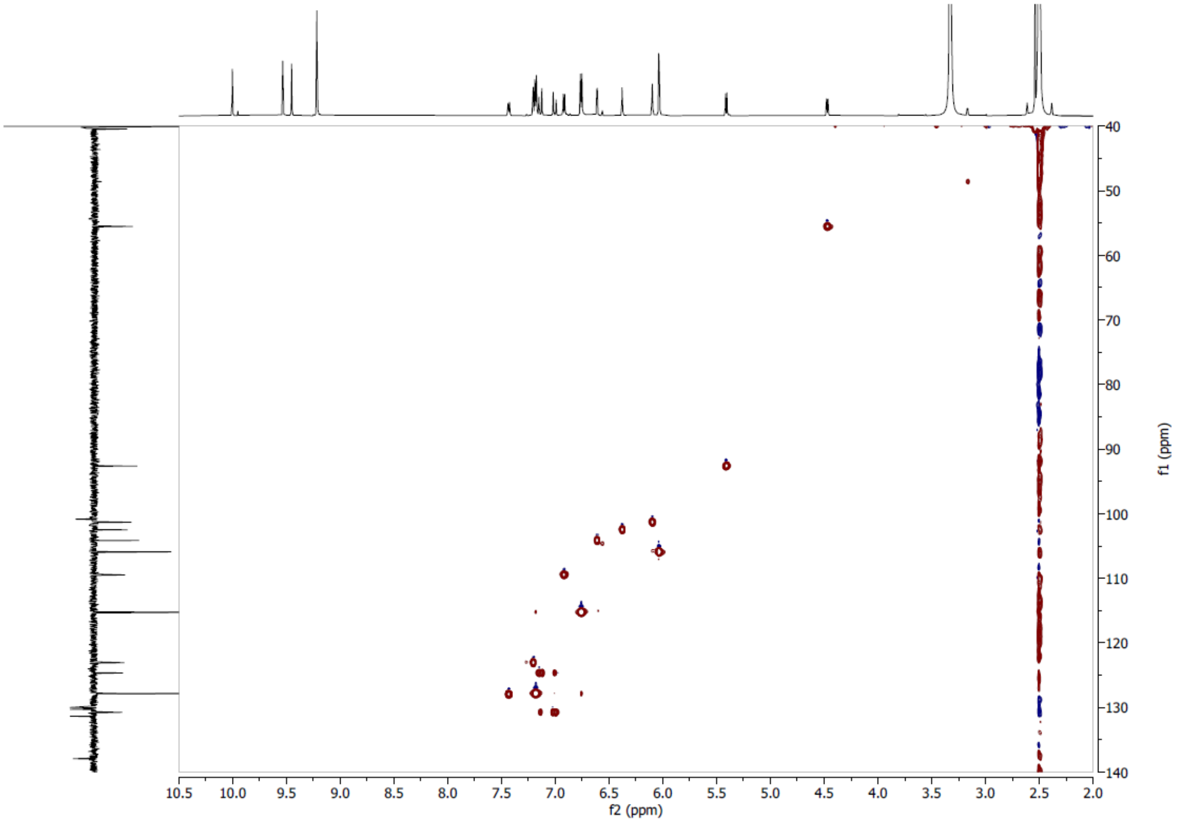

Edited HSQC NMR spectrum of compound **20** in DMSO-d_6

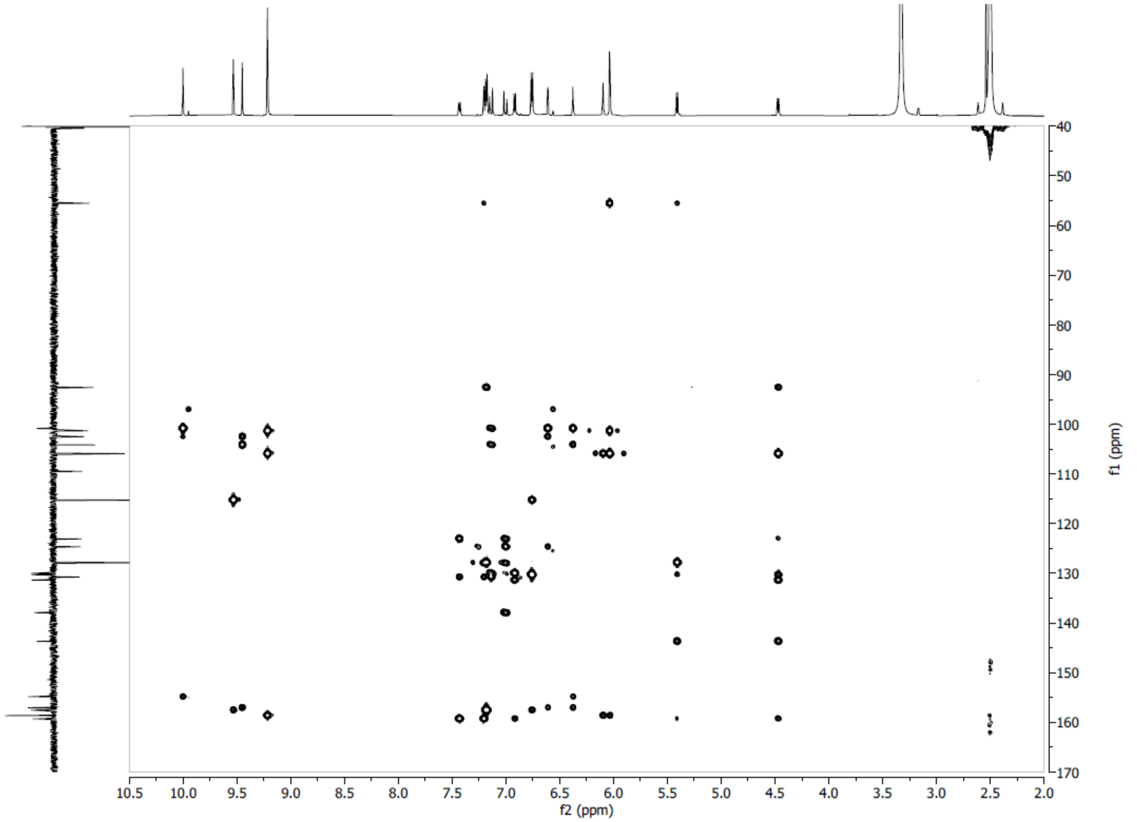

HMBC NMR spectrum of compound **20** in DMSO-d_6

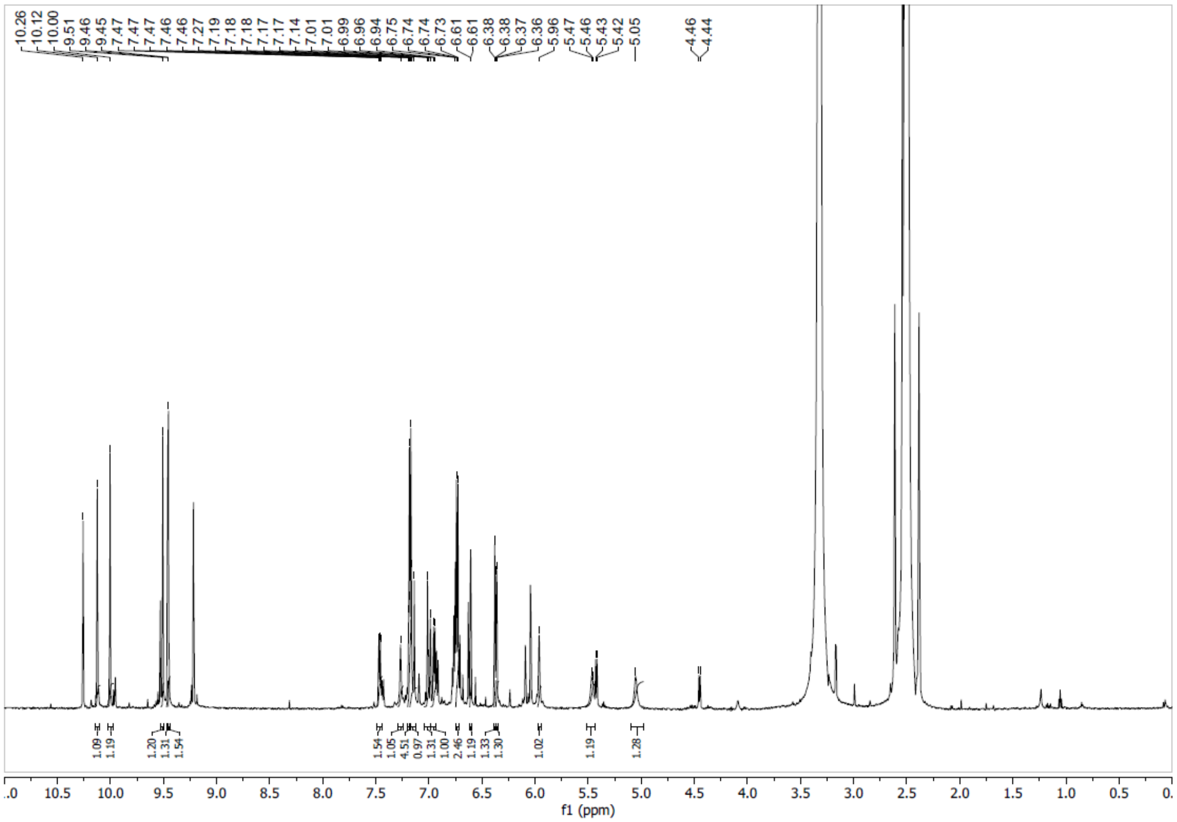

^{1}H NMR spectrum of compound **21** in DMSO-d_6 at 600 MHz

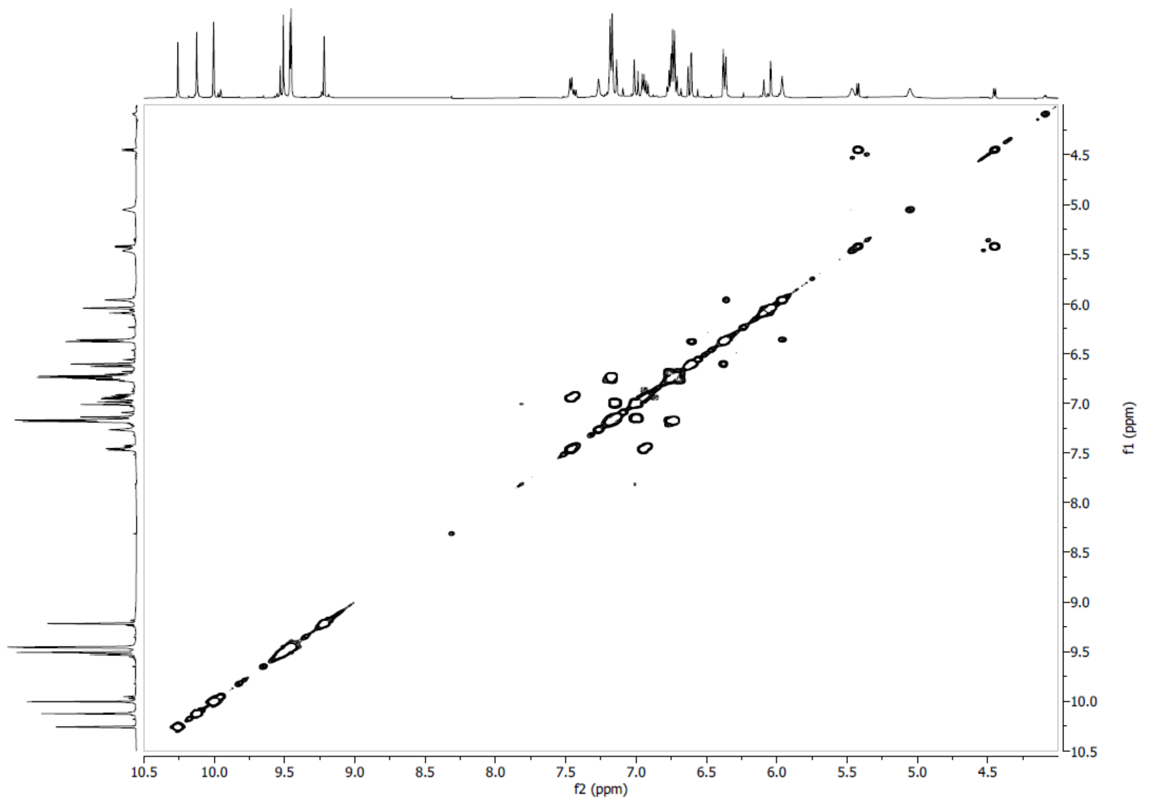

COSY NMR spectrum of compound **21** in DMSO-d_6

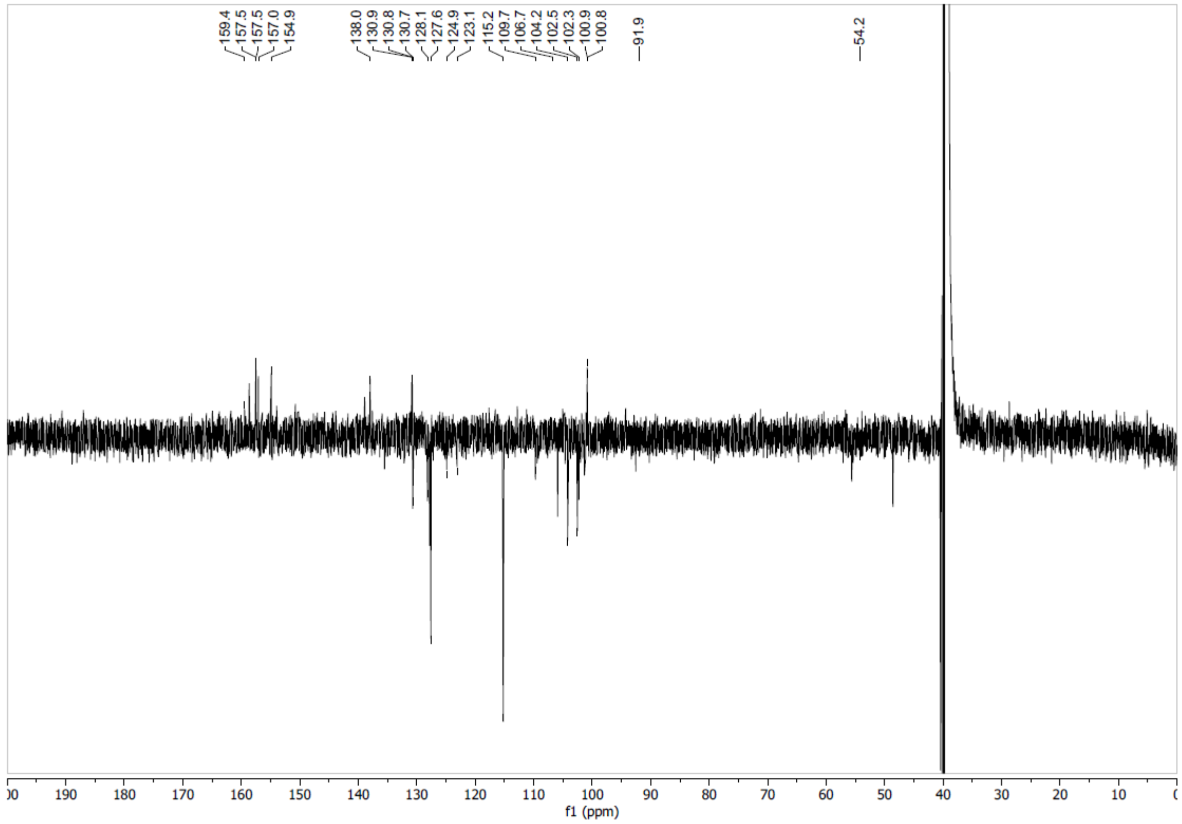

^{13}C-DEPTQ NMR spectrum of compound **21** in DMSO-d_6 at 151 MHz

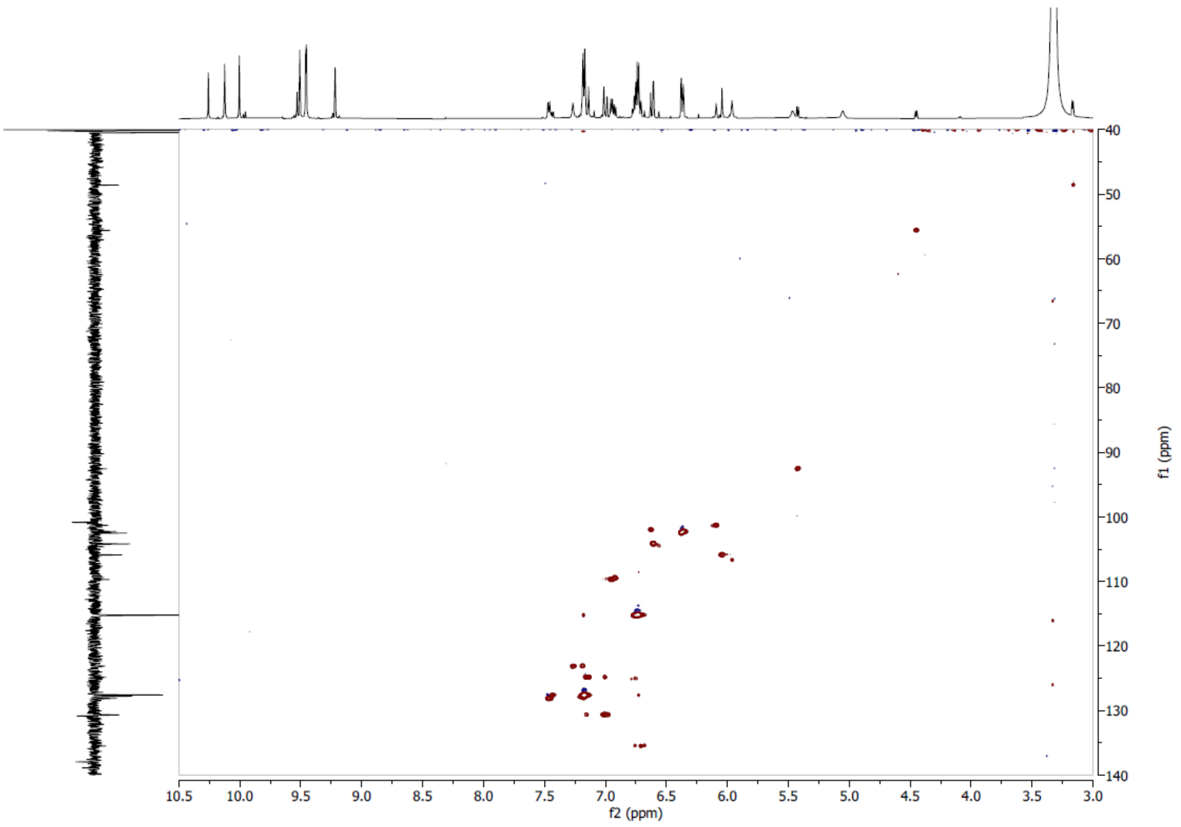

Edited HSQC NMR spectrum of compound **21** in DMSO-d_6

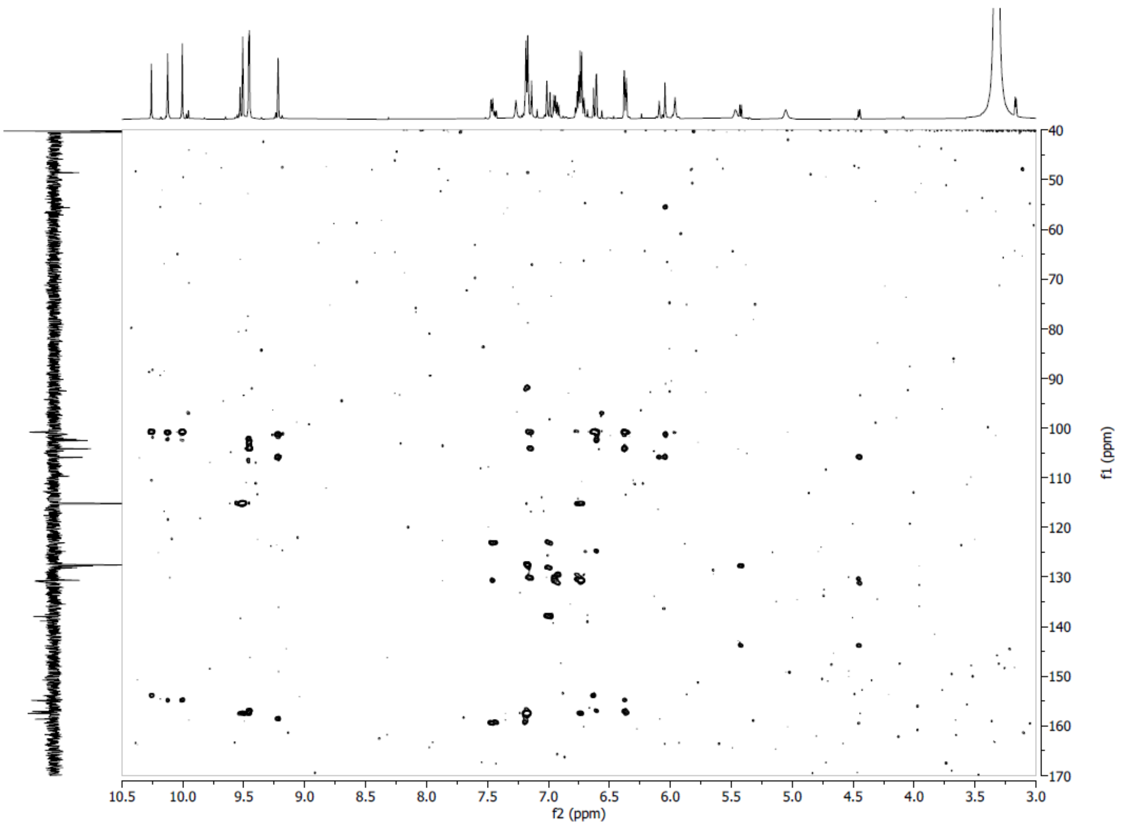

HMBC NMR spectrum of compound **21** in DMSO-d_6

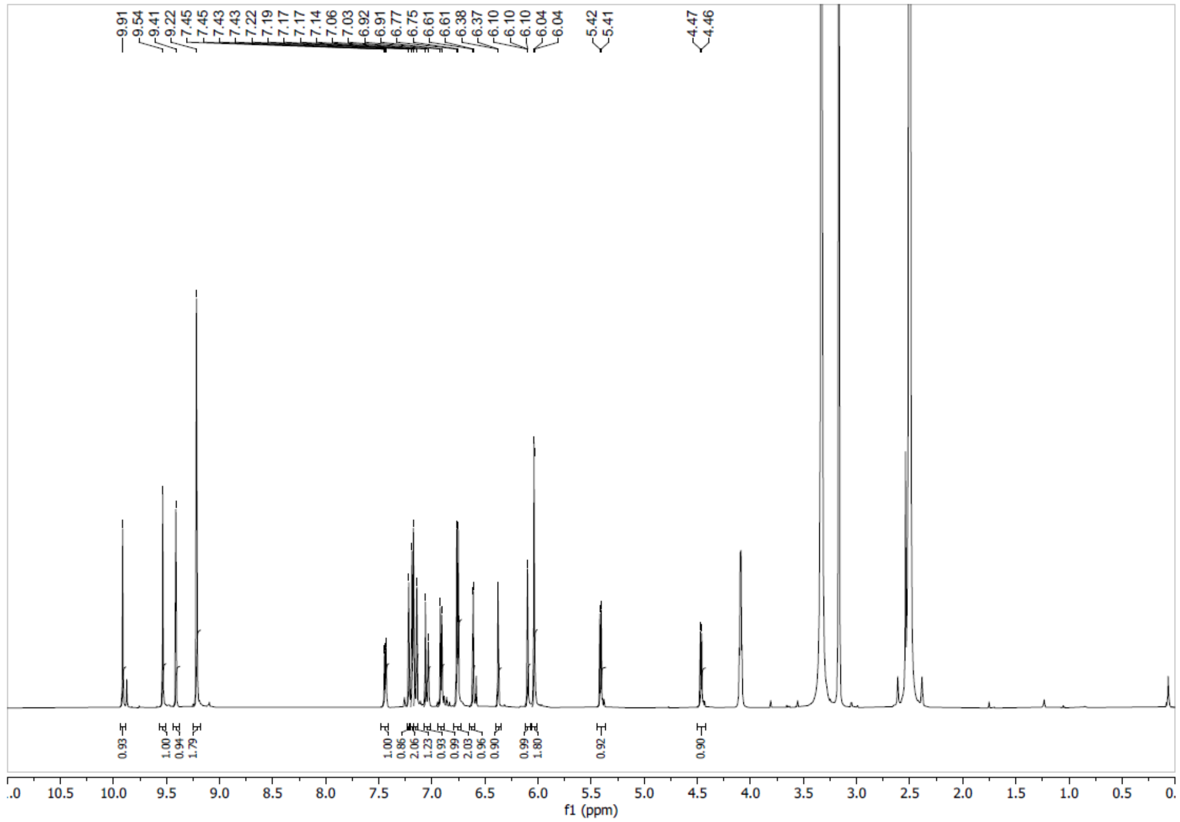

^{1}H NMR spectrum of compound **22** in DMSO-d_6 at 600 MHz

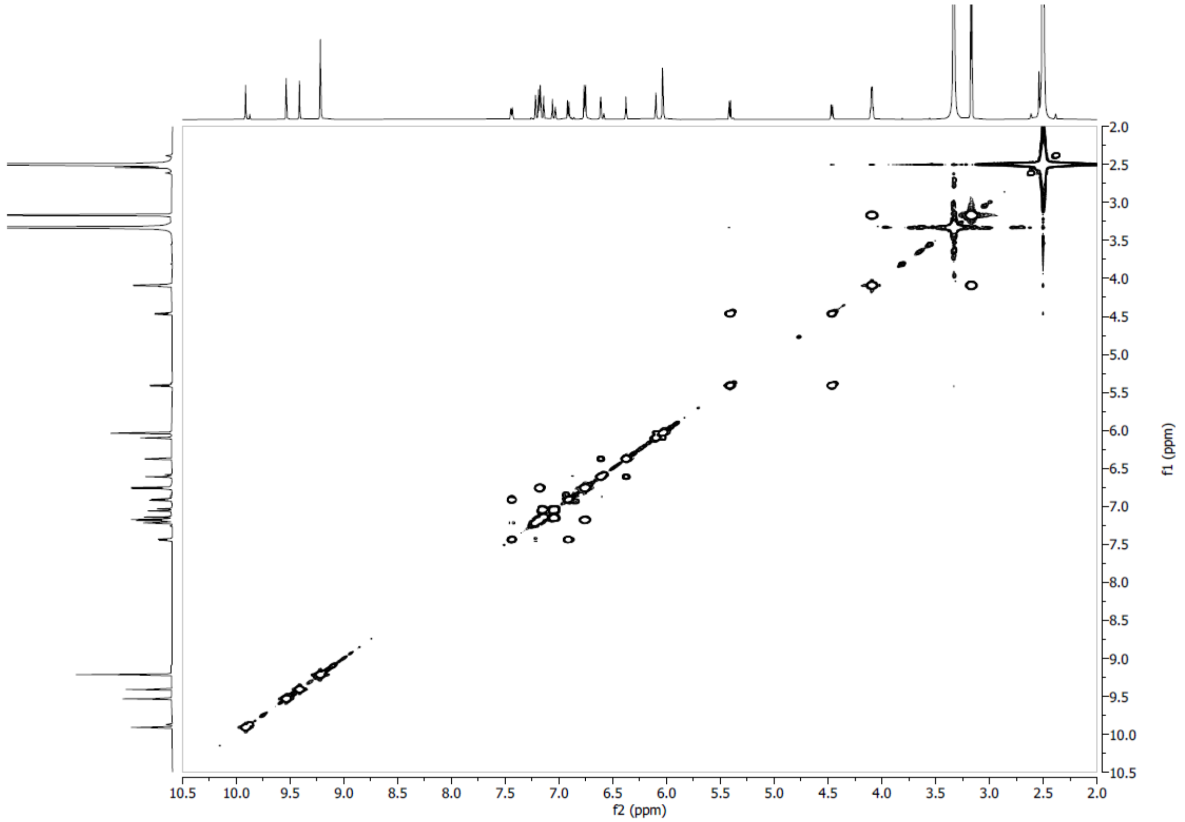

COSY NMR spectrum of compound **22** in DMSO-d_6

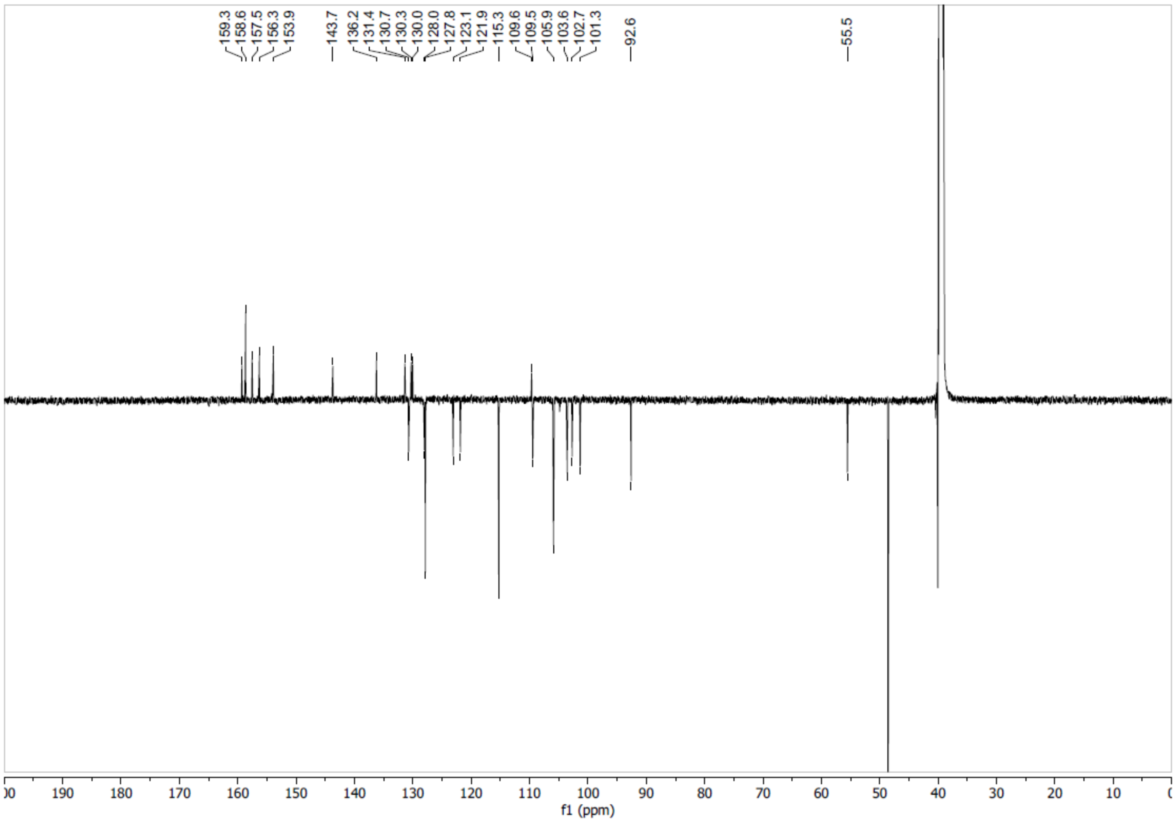

^{13}C-DEPTQ NMR spectrum of compound **22** in DMSO-d_6 at 151 MHz

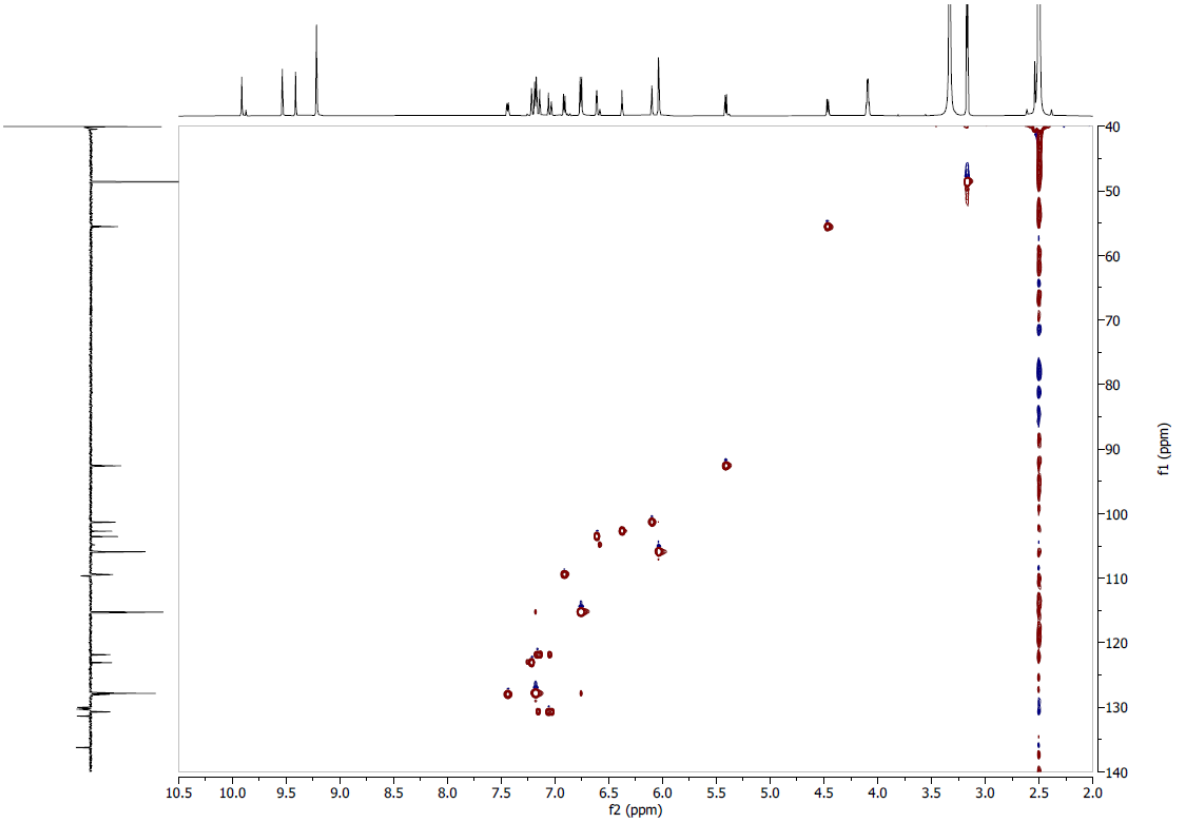

Edited HSQC NMR spectrum of compound **22** in DMSO-d_6

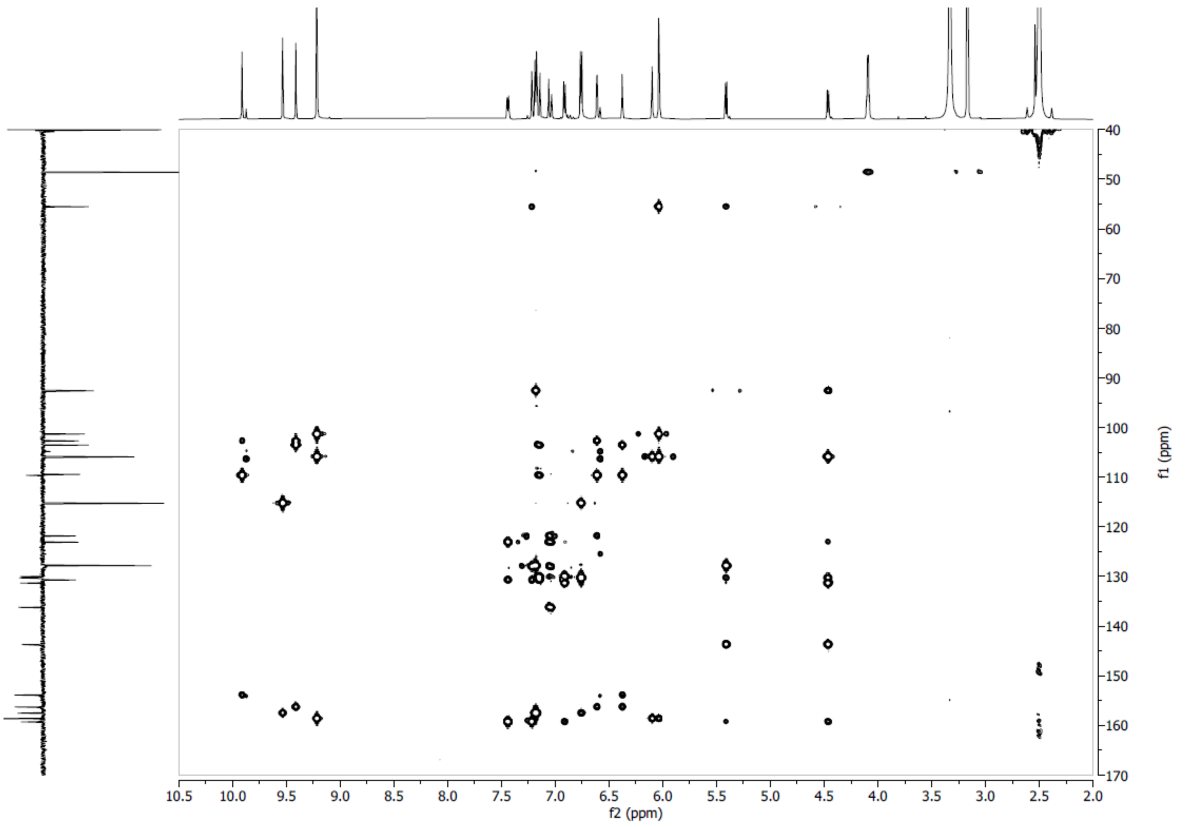

HMBC NMR spectrum of compound **22** in DMSO-d_6

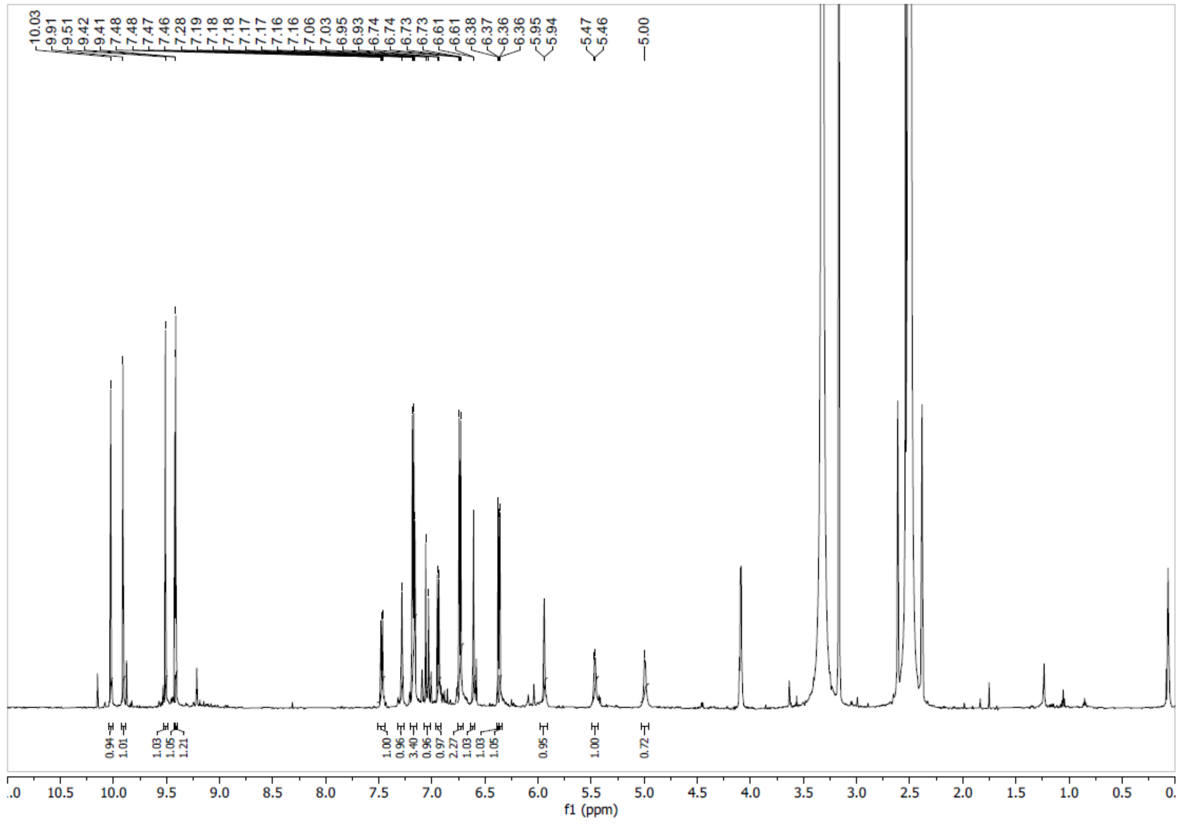

^{1}H NMR spectrum of compound **23** in DMSO-d_6 at 600 MHz

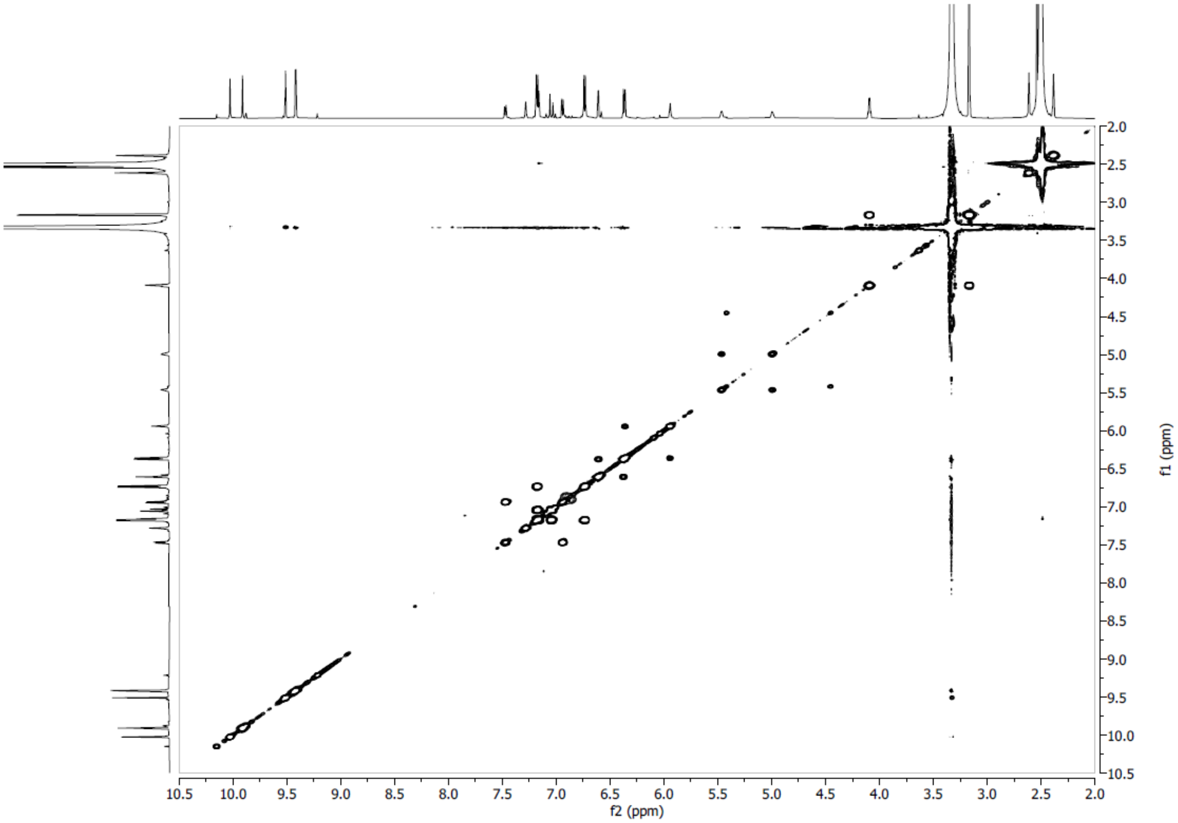

COSY NMR spectrum of compound **23** in DMSO-d_6

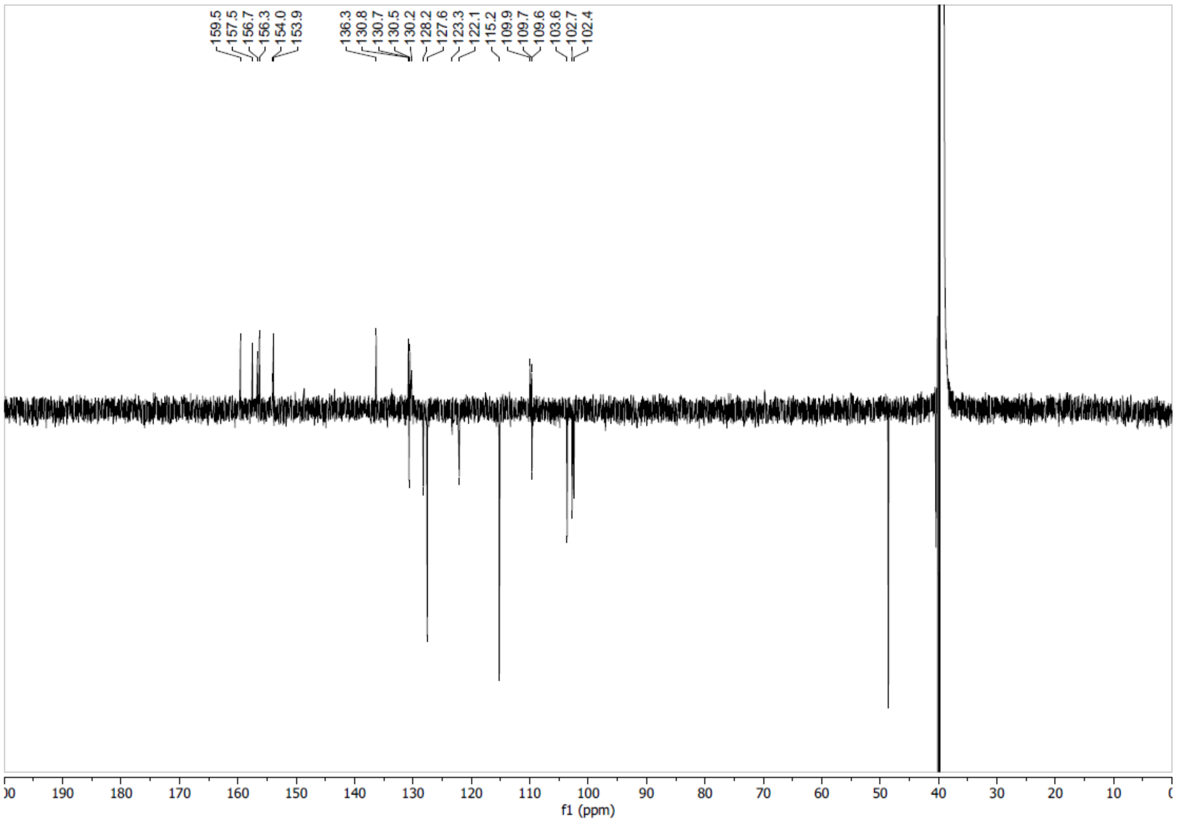

^{13}C-DEPTQ NMR spectrum of compound **23** in DMSO-d_6 at 151 MHz

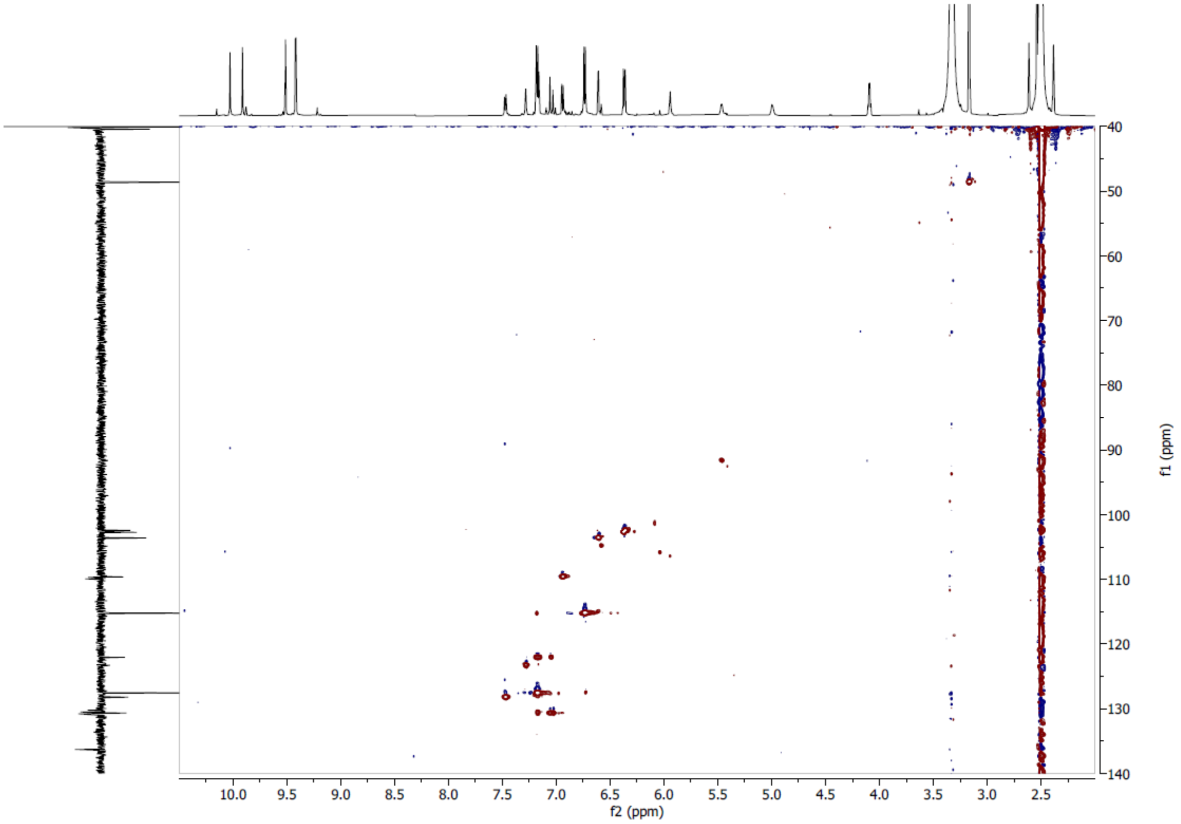

Edited HSQC NMR spectrum of compound **23** in DMSO-d_6

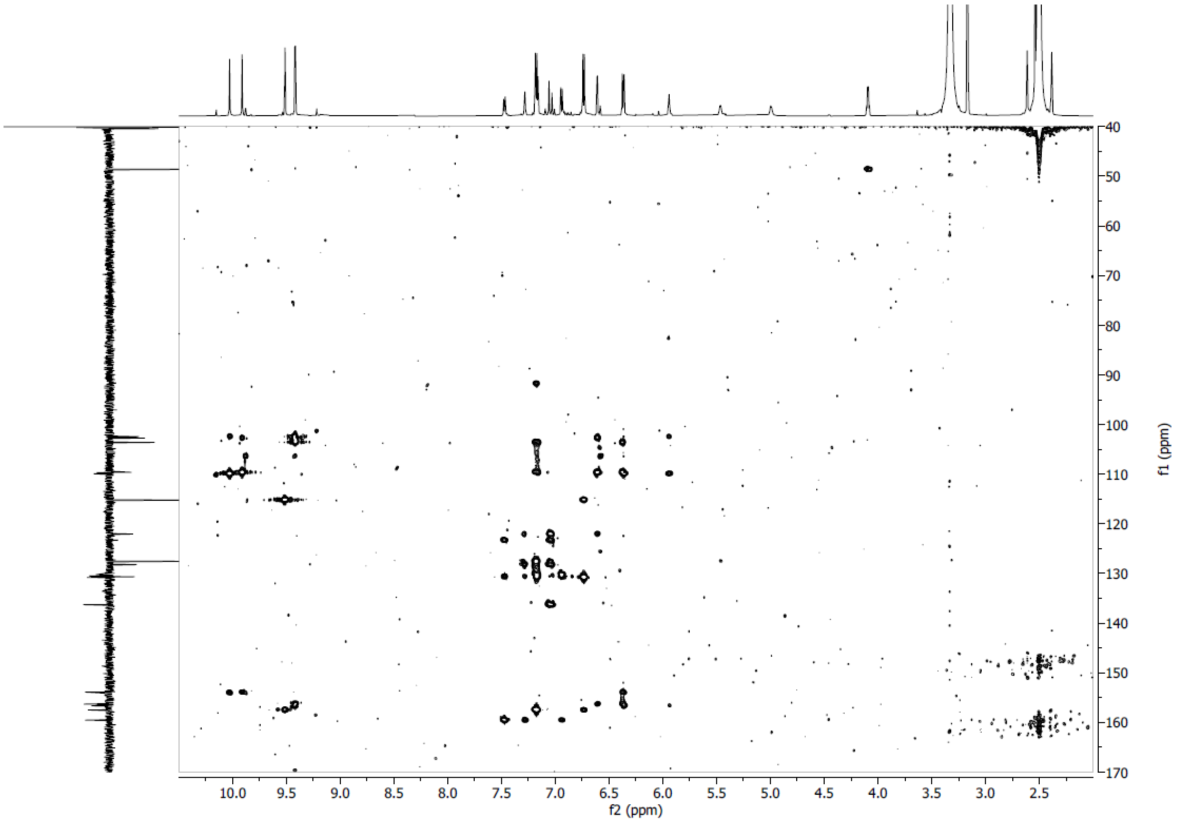

HMBC NMR spectrum of compound **23** in DMSO-d_6

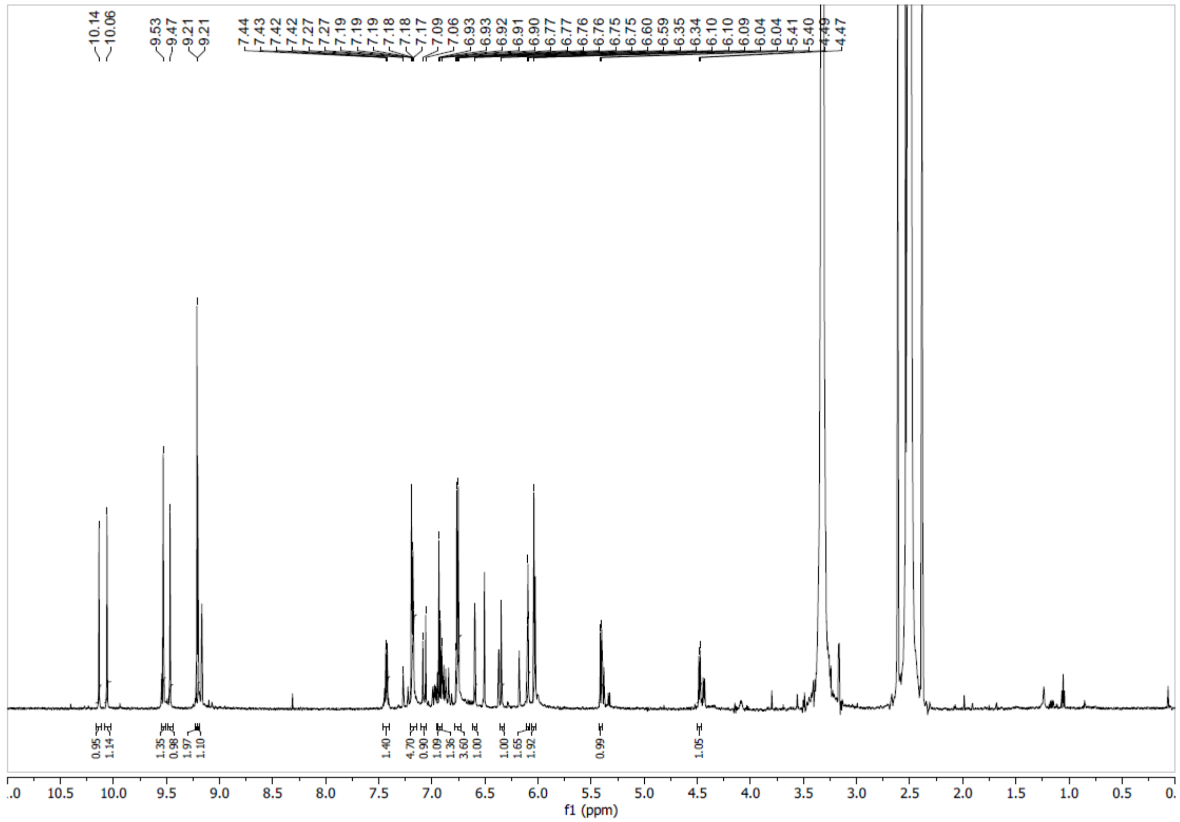

^{1}H NMR spectrum of compound **24** in DMSO-d_6 at 600 MHz

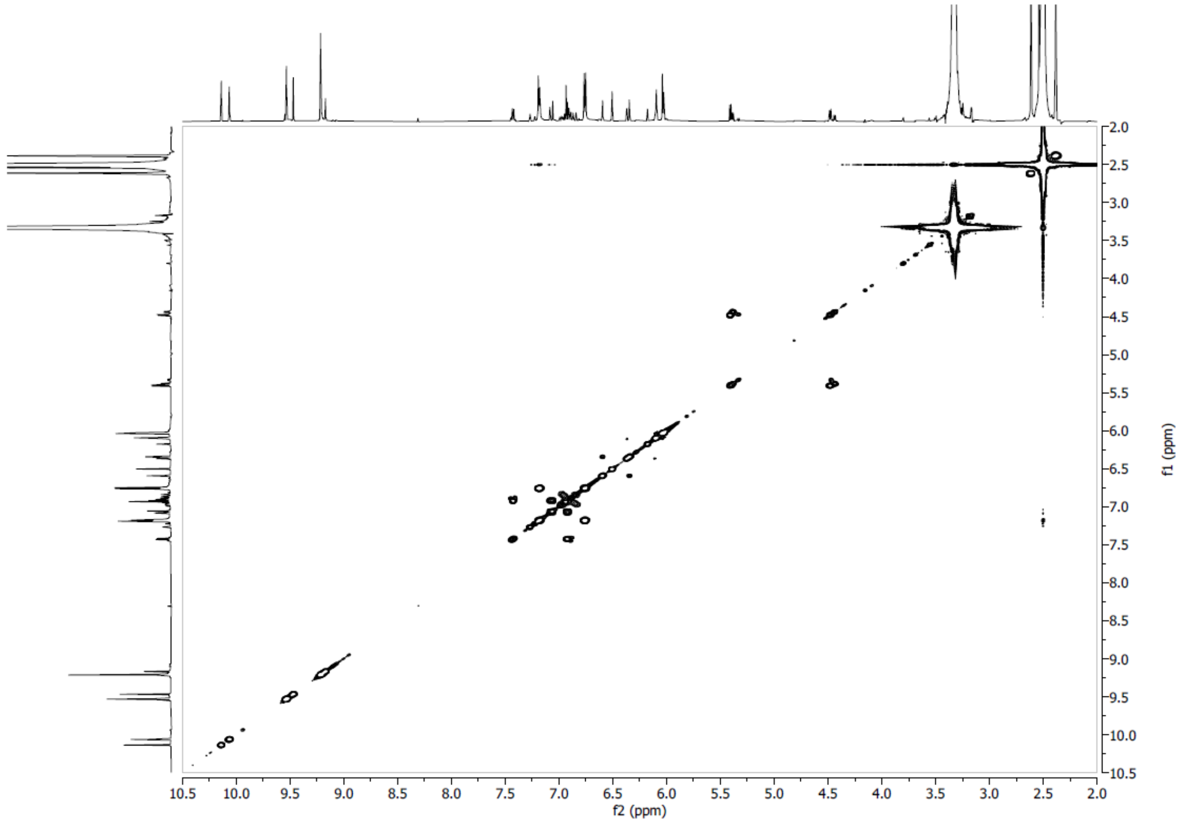

COSY NMR spectrum of compound **24** in DMSO-d_6

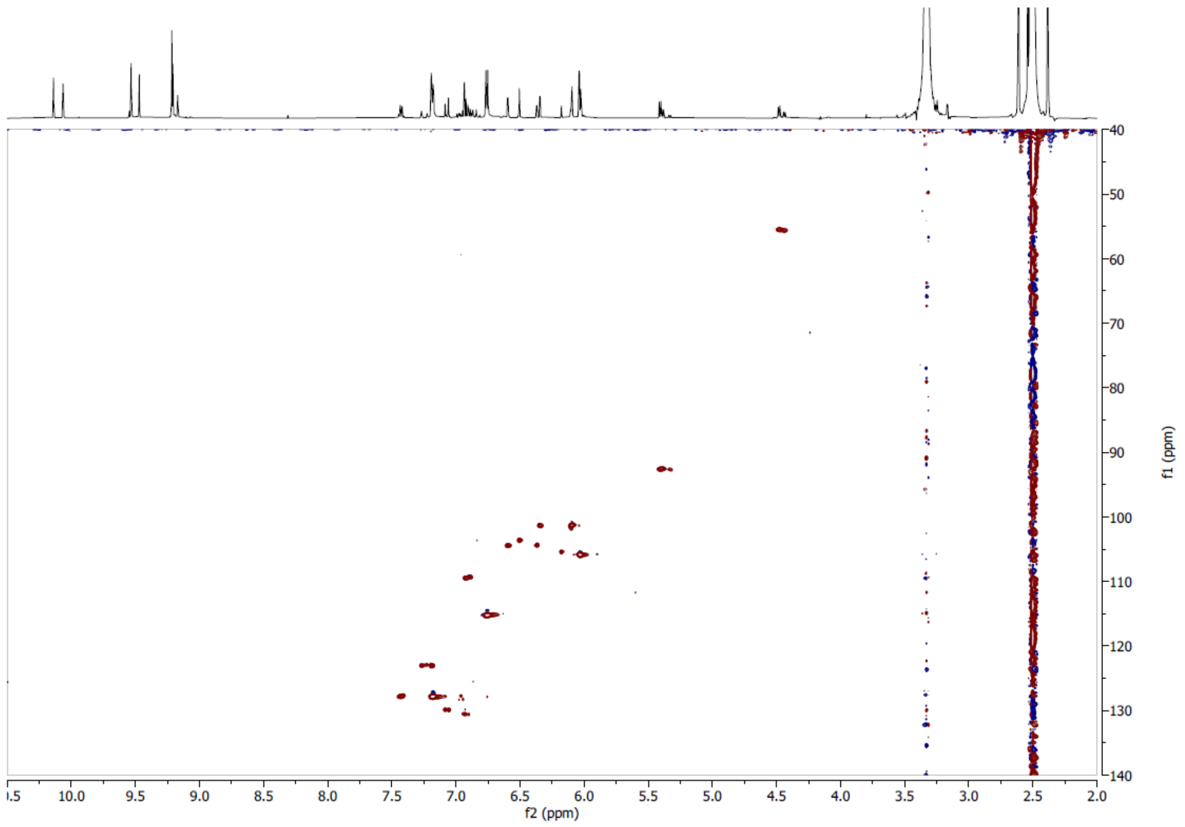

Edited HSQC NMR spectrum of compound **24** in DMSO-d_6

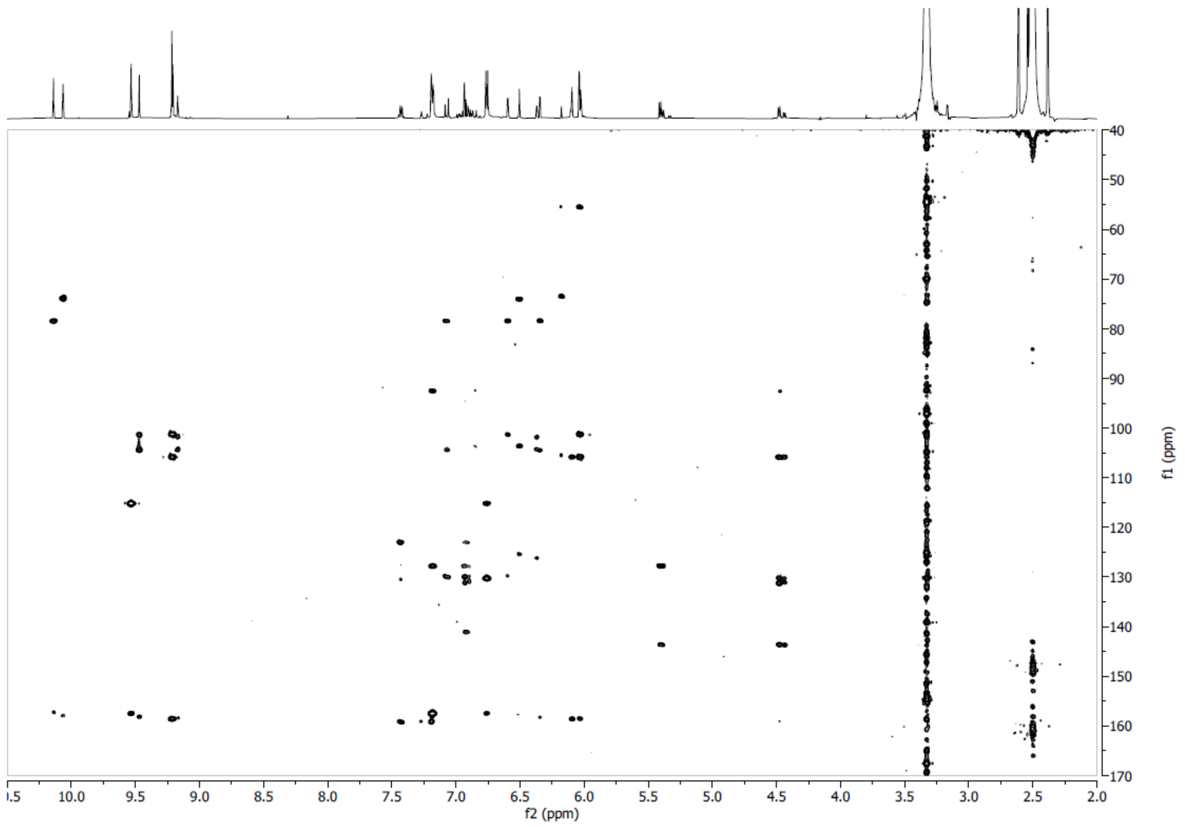

HMBC NMR spectrum of compound **24** in DMSO-d_6

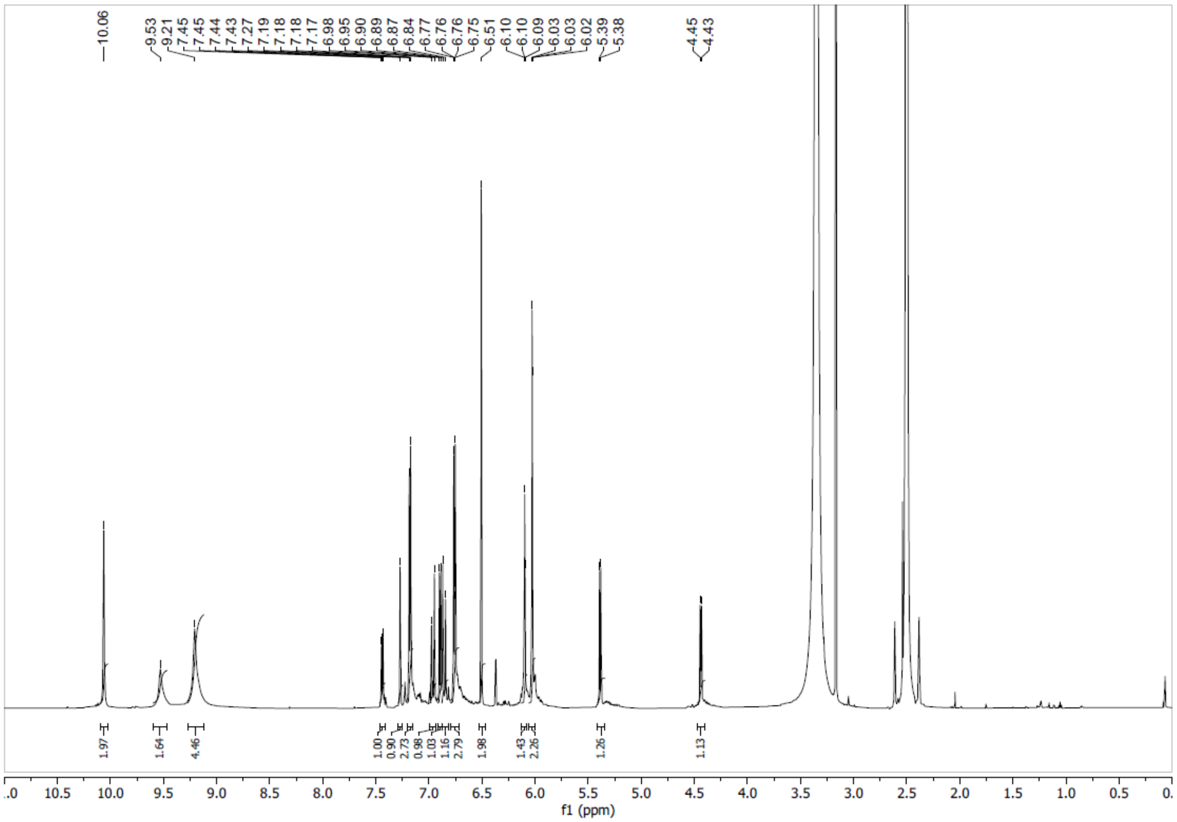

^{1}H NMR spectrum of compound **25** in DMSO-d_6 at 600 MHz

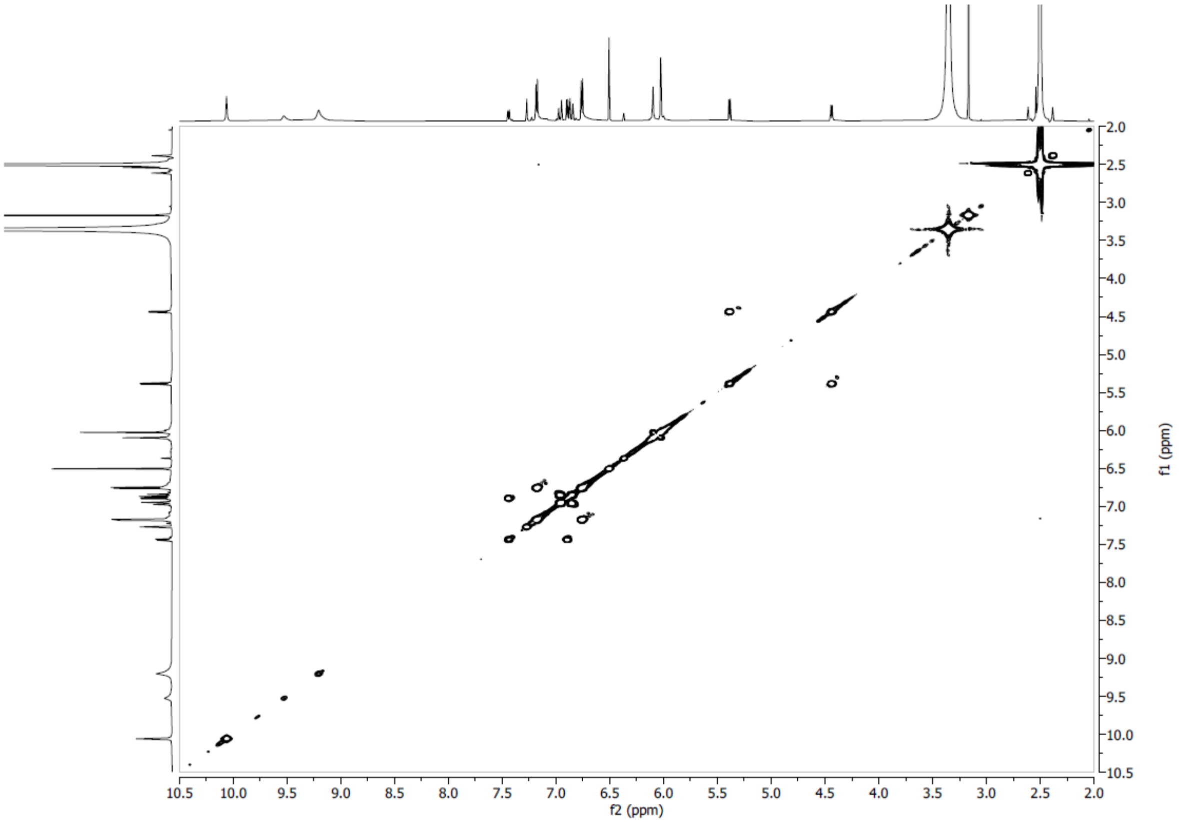

COSY NMR spectrum of compound **25** in DMSO-d_6

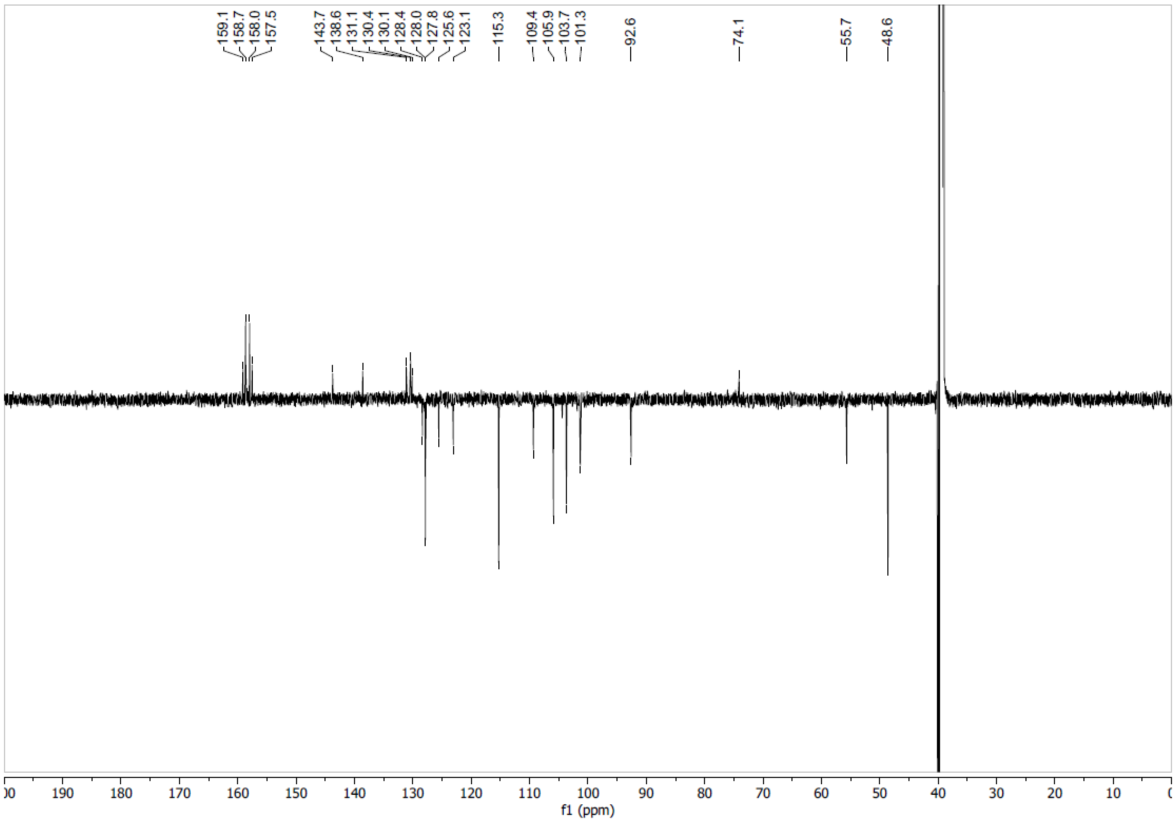

^{13}C-DEPTQ NMR spectrum of compound **25** in DMSO-d_6 at 151 MHz

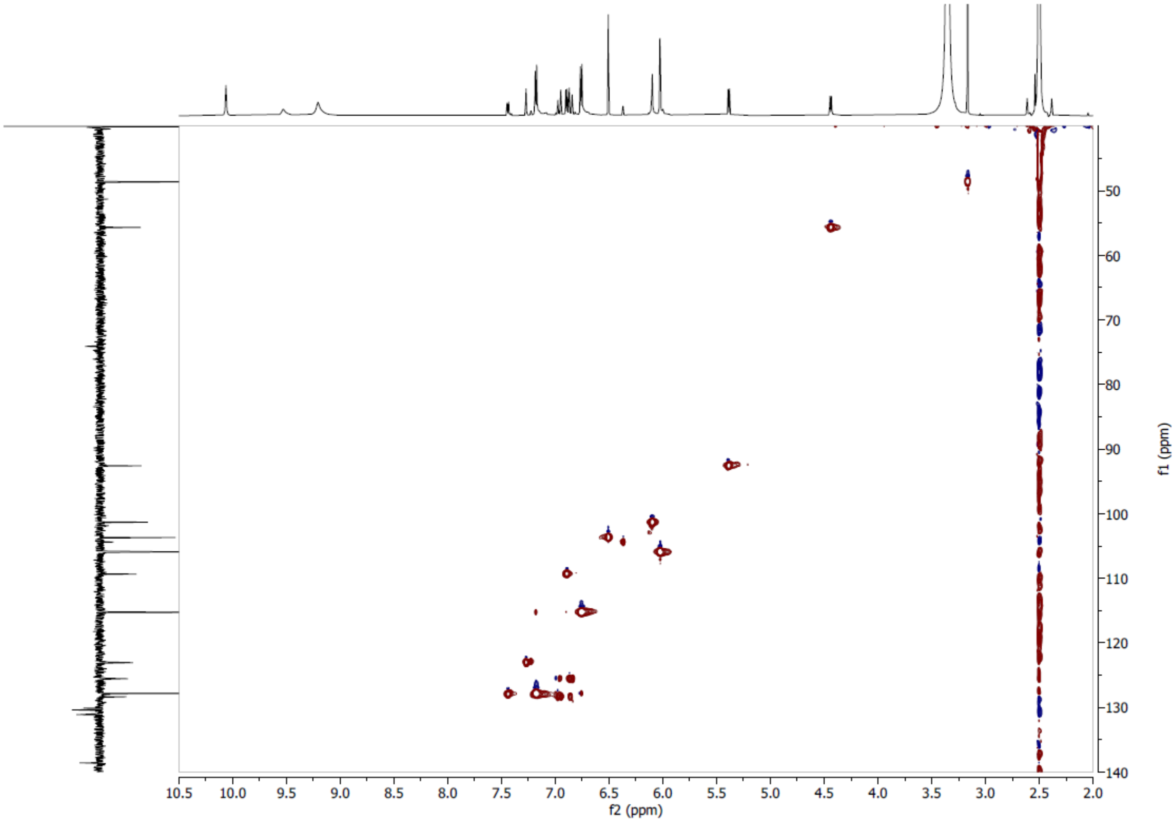

Edited HSQC NMR spectrum of compound **25** in DMSO-d_6

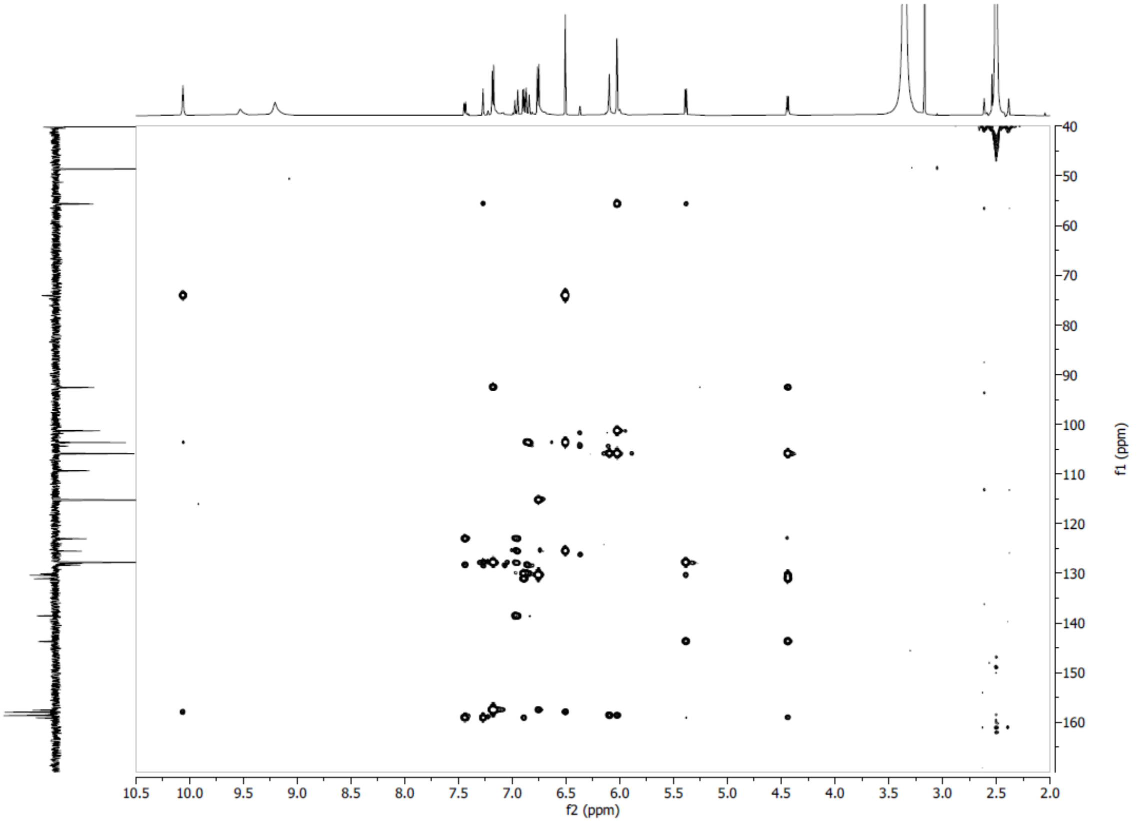

HMBC NMR spectrum of compound **25** in DMSO-d_6

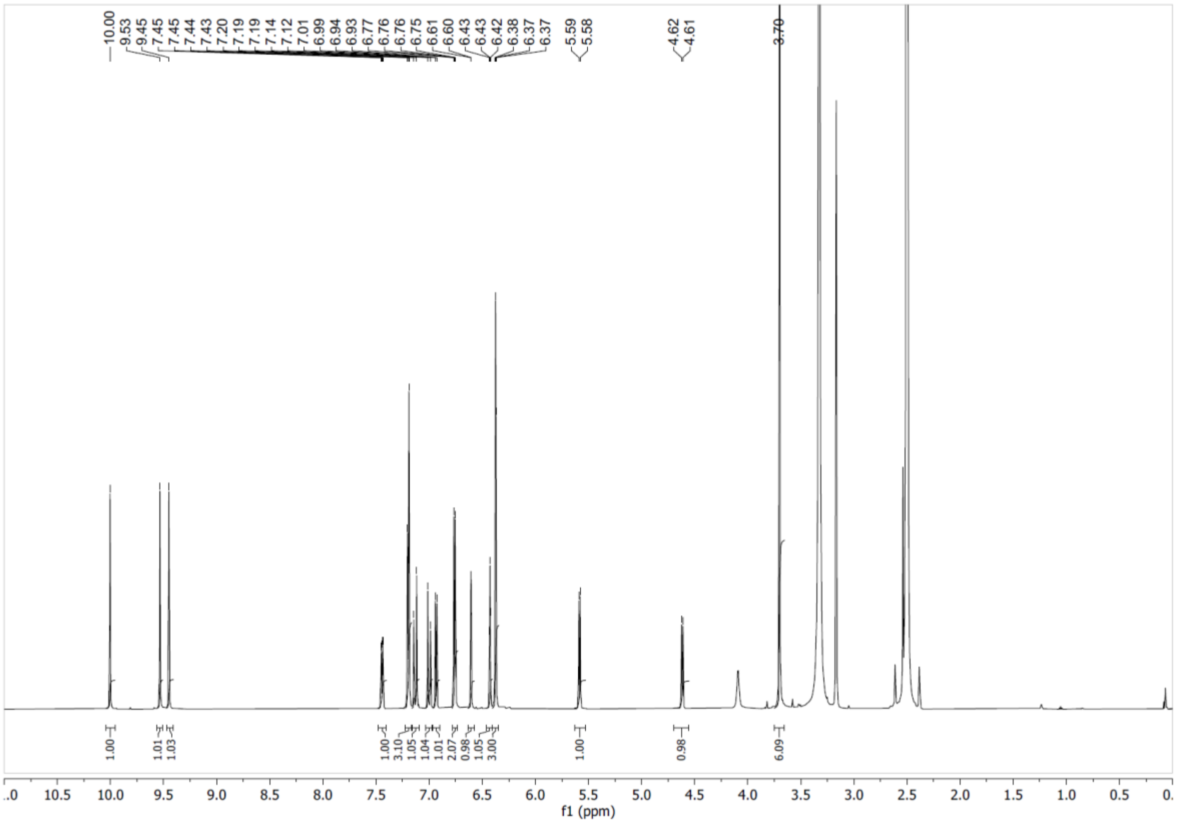

^{1}H NMR spectrum of compound **26** in DMSO-d_6 at 600 MHz

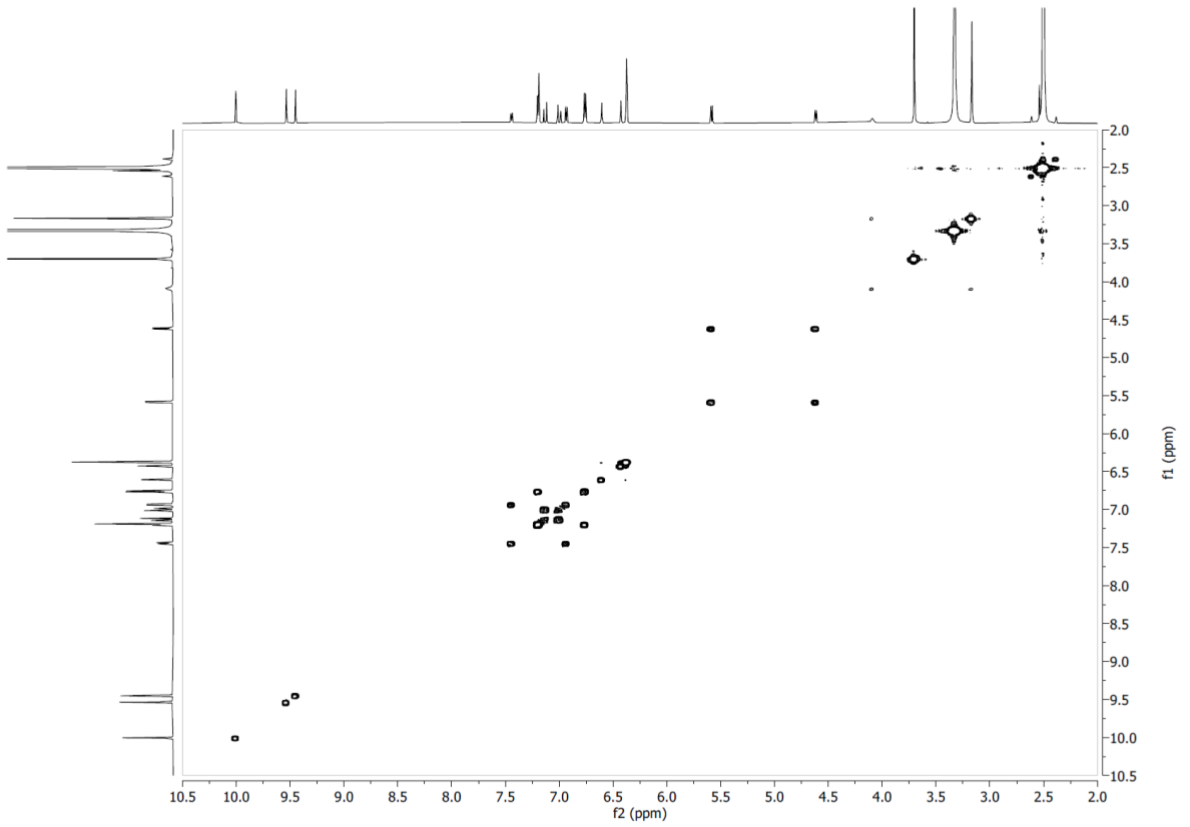

COSY NMR spectrum of compound **26** in DMSO-d_6

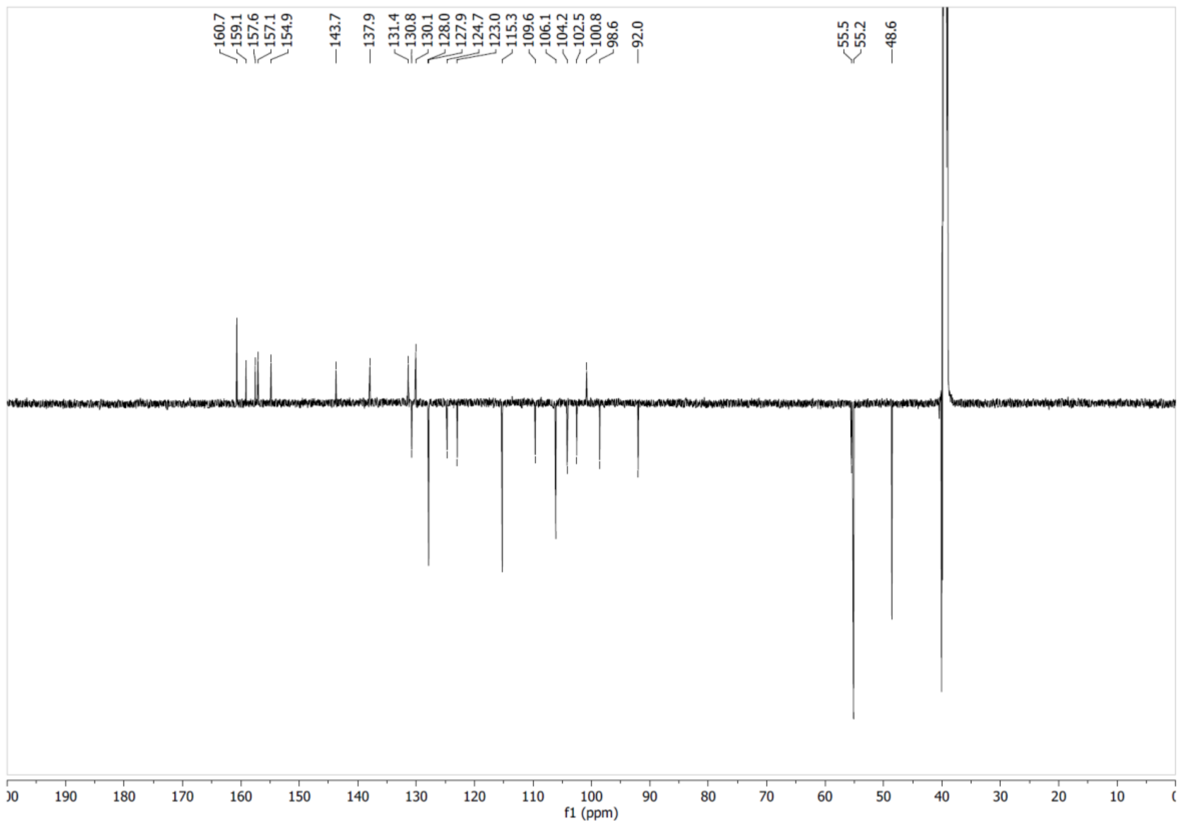

^{13}C-DEPTQ NMR spectrum of compound **26** in DMSO-d_6 at 151 MHz

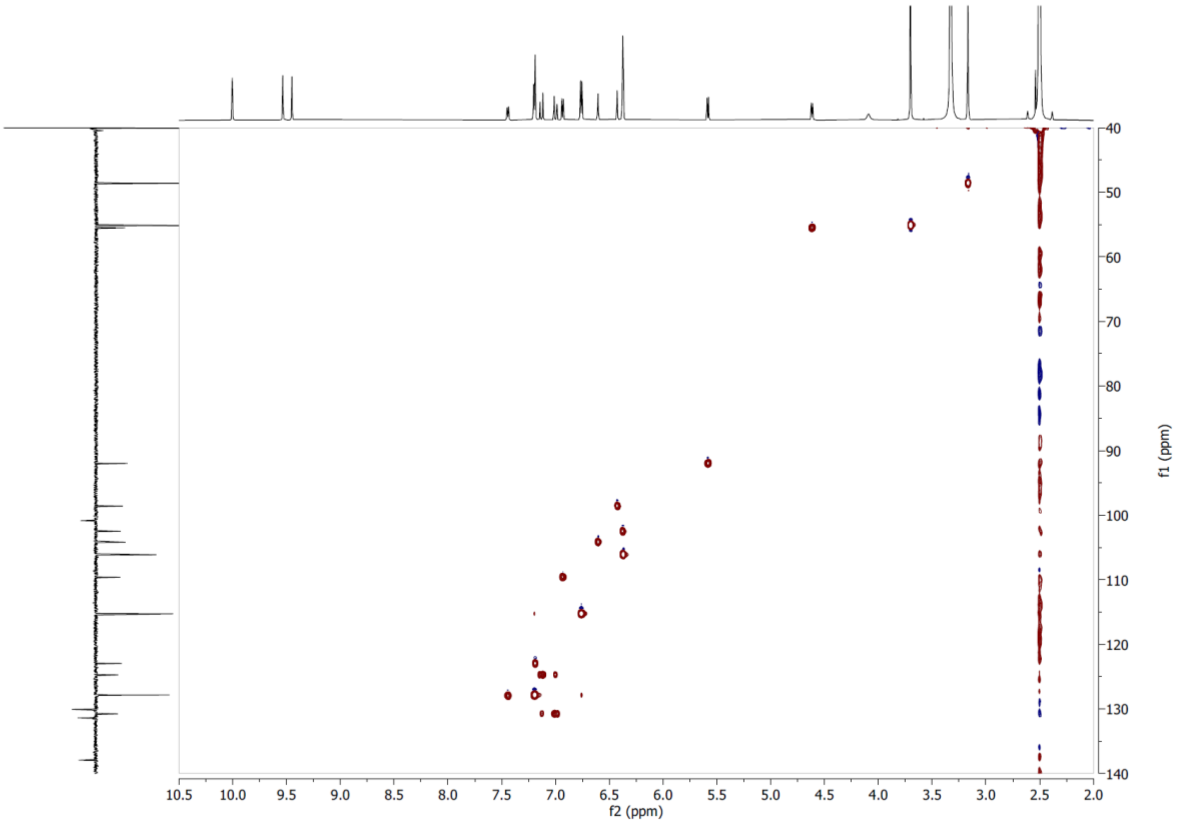

Edited HSQC NMR spectrum of compound **26** in DMSO-d_6

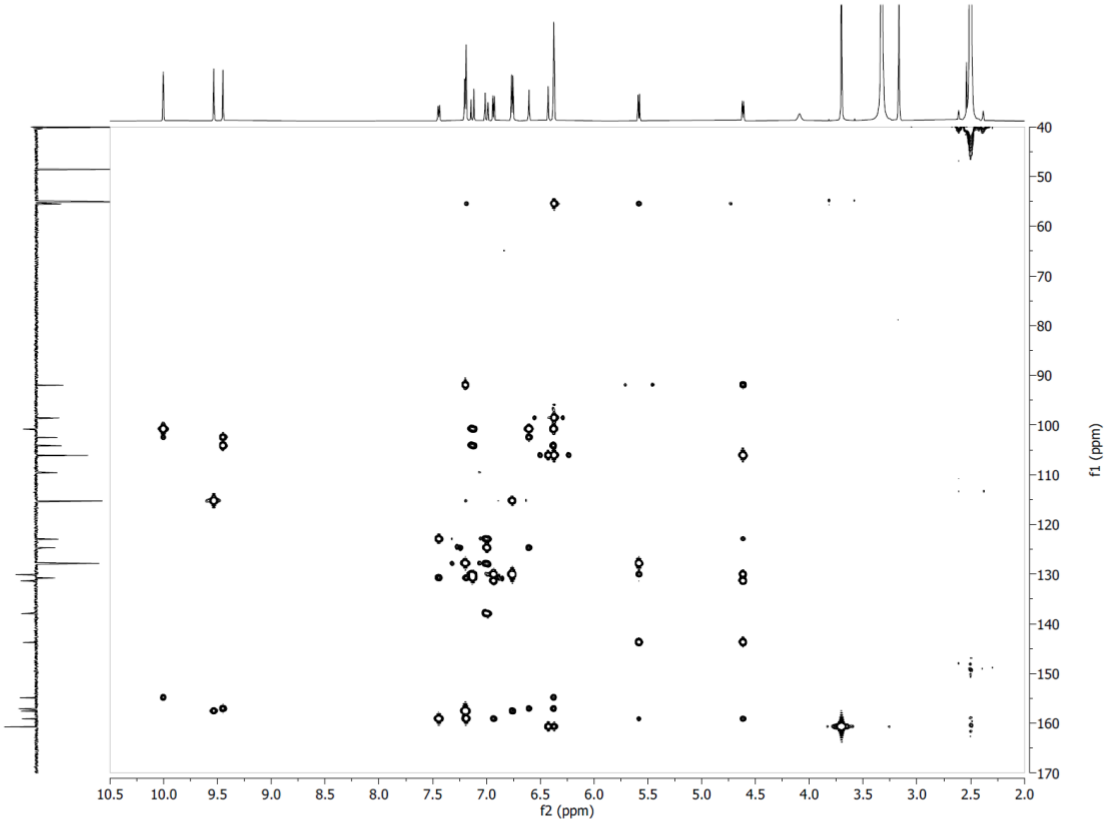

HMBC NMR spectrum of compound **26** in DMSO-d_6

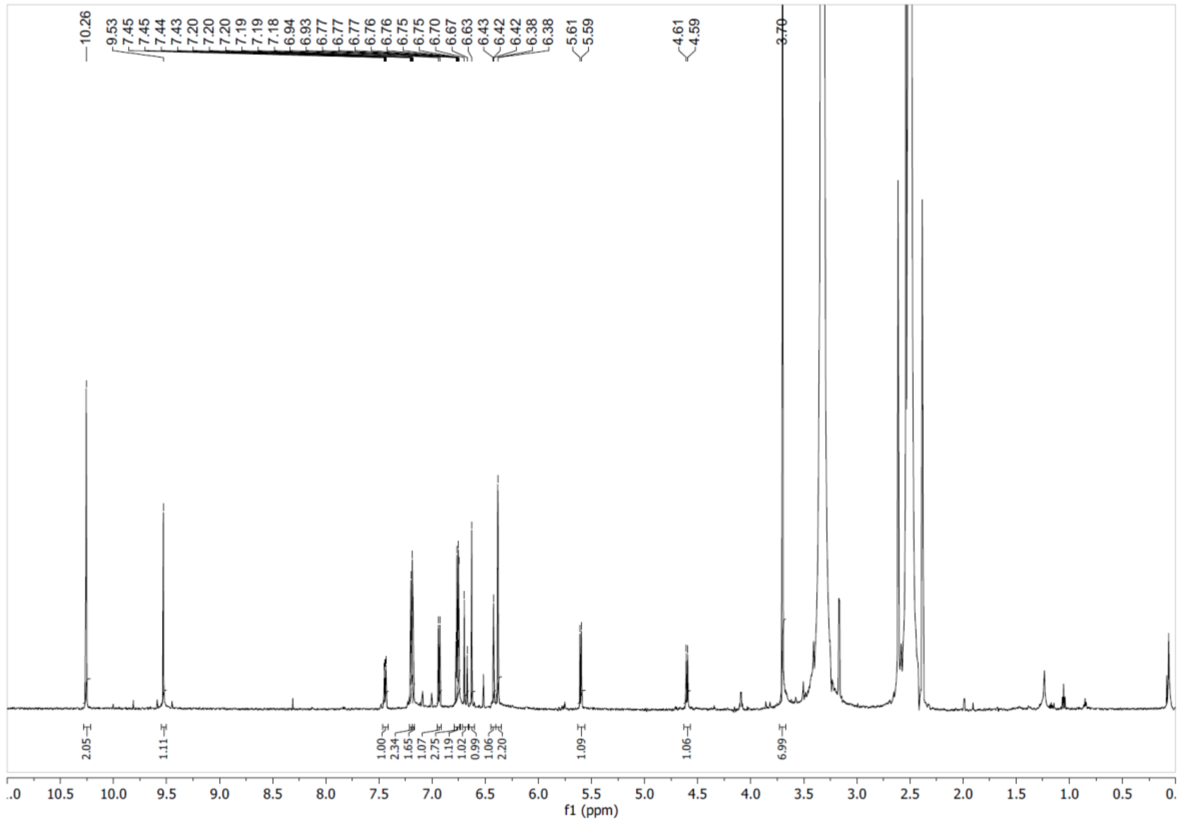

^{1}H NMR spectrum of compound **27** in DMSO-d_6 at 600 MHz

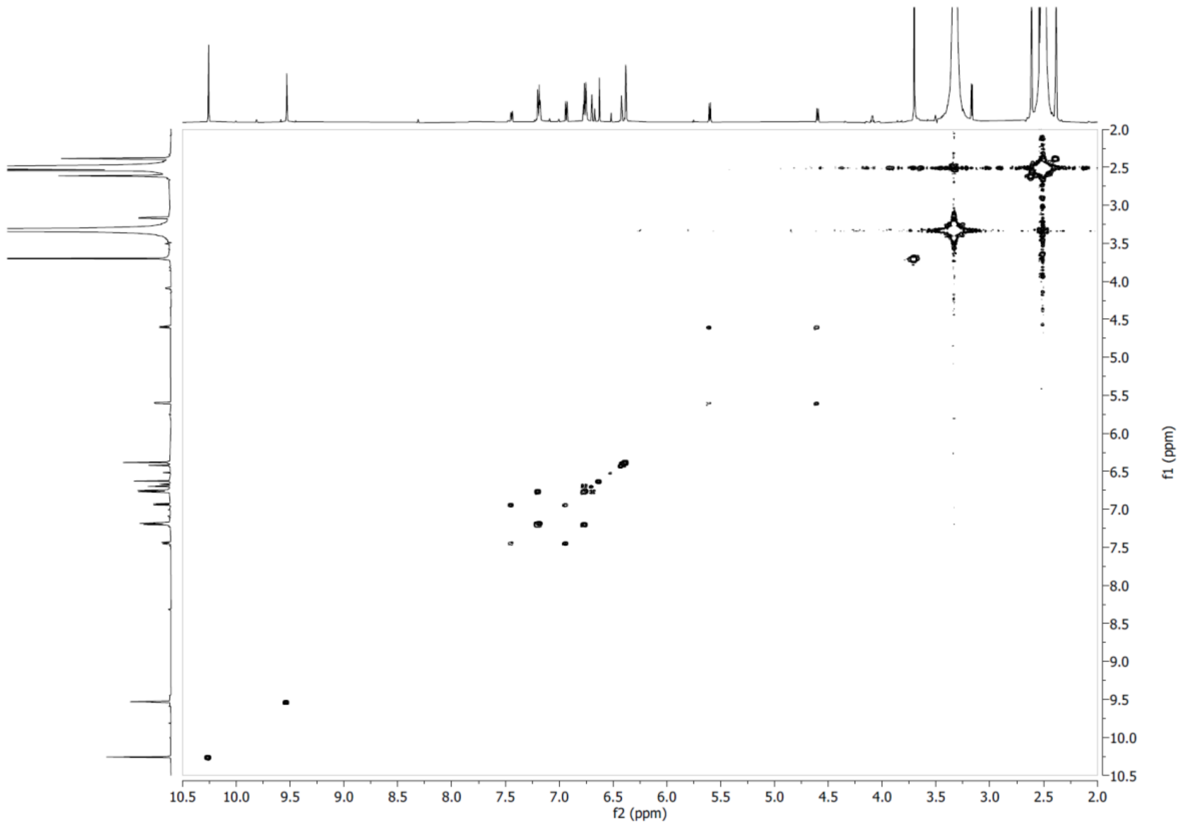

COSY NMR spectrum of compound **27** in DMSO-d_6

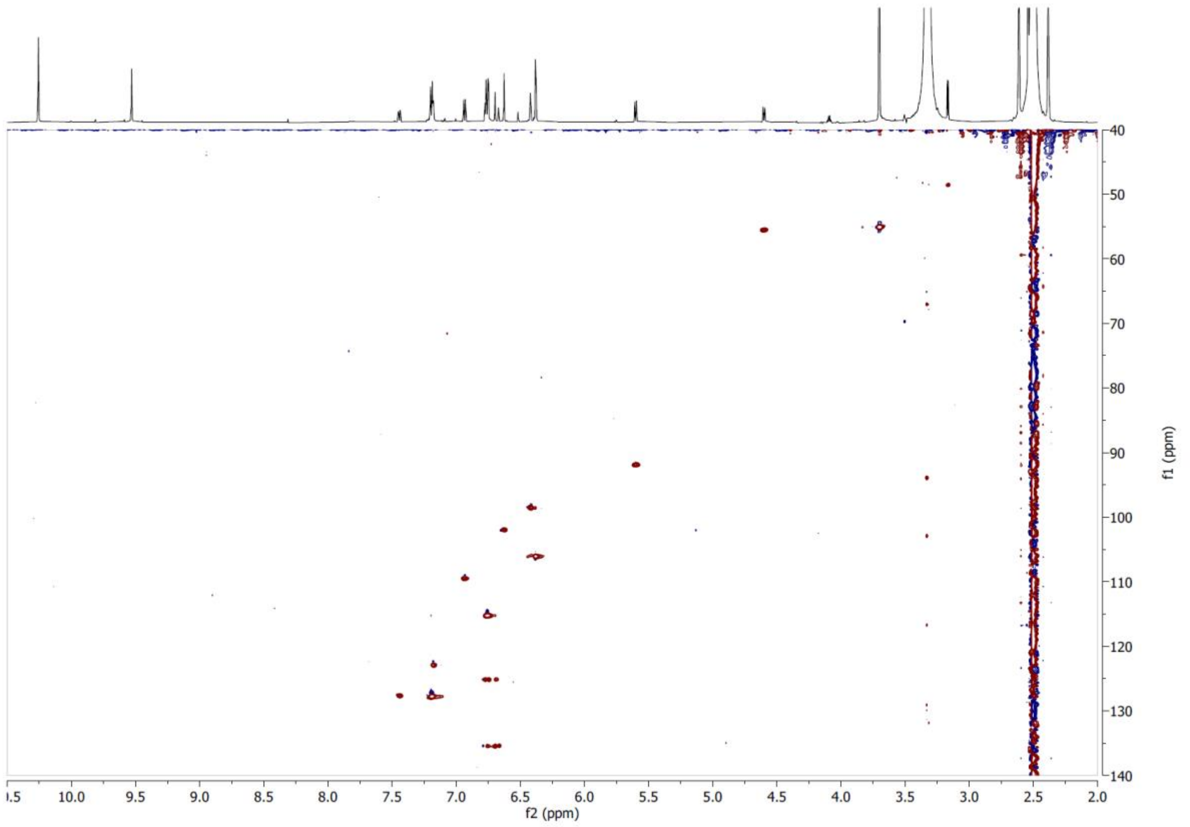

Edited HSQC NMR spectrum of compound **27** in DMSO-d_6

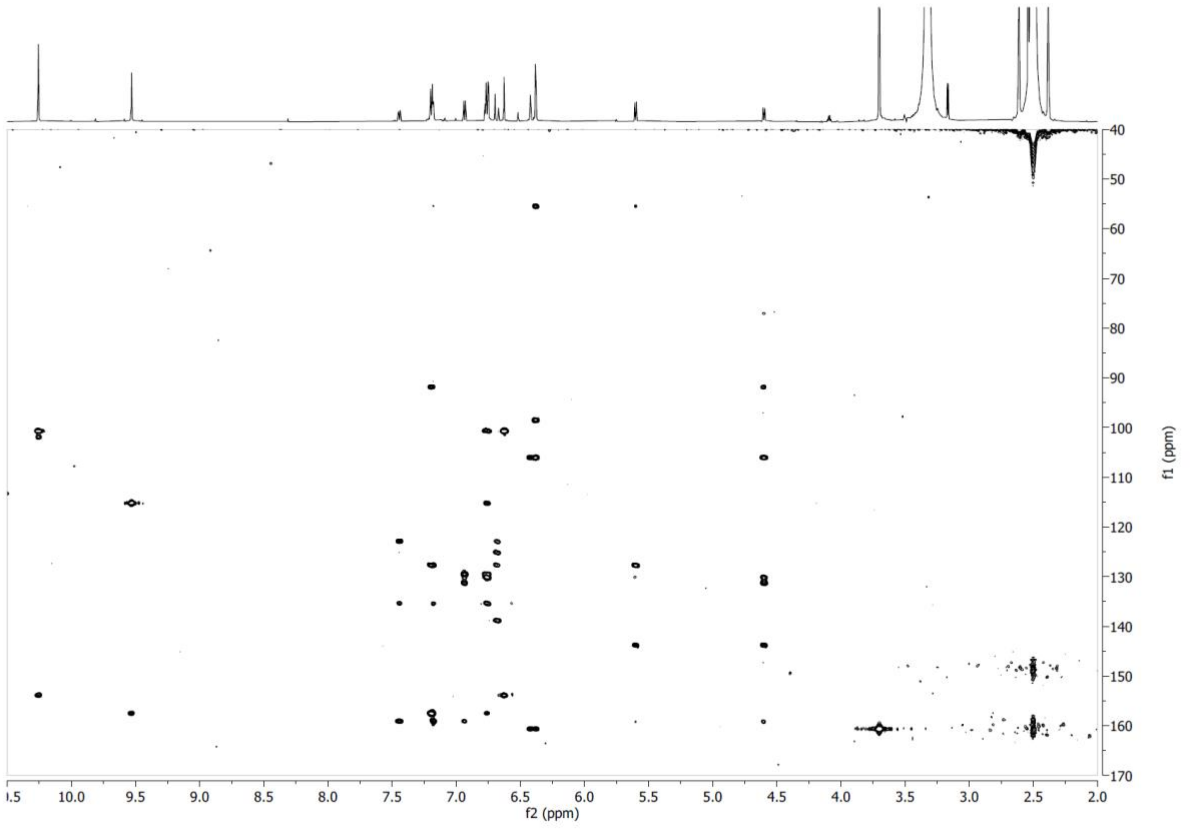

HMBC NMR spectrum of compound **27** in DMSO-d_6

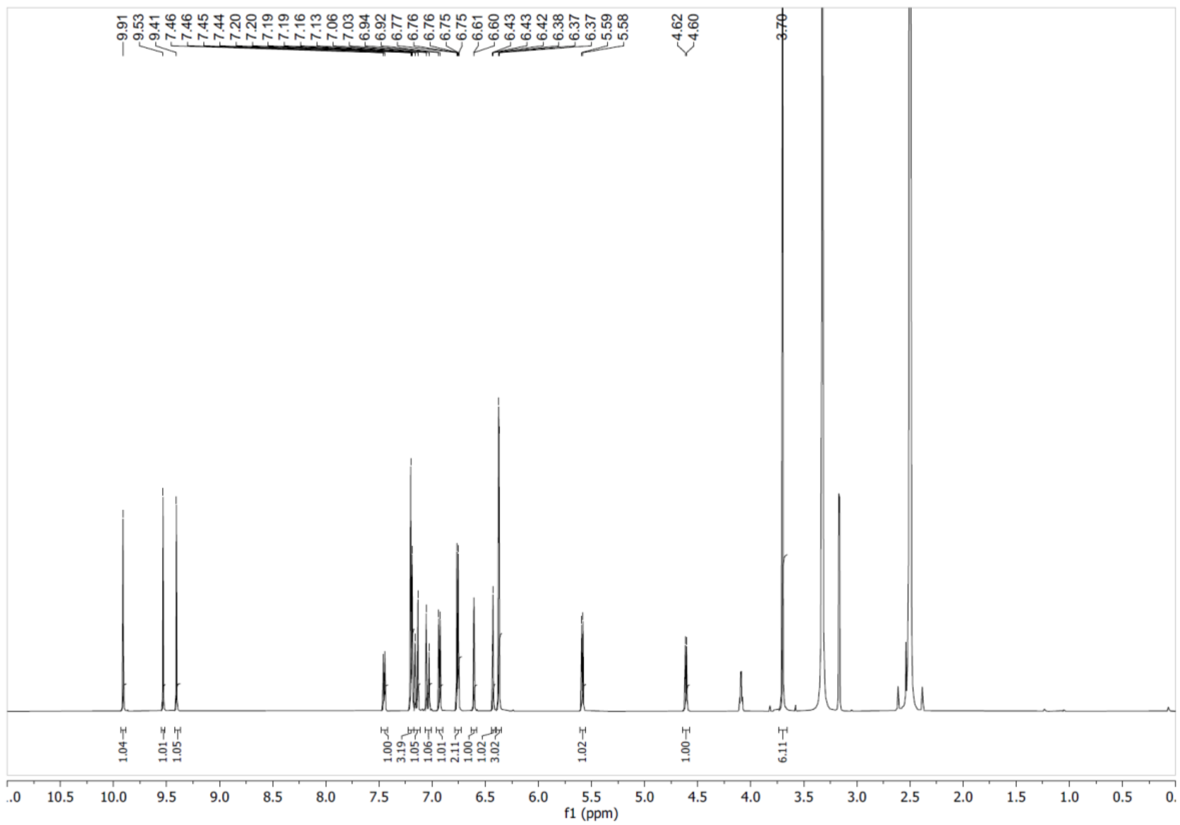

^{1}H NMR spectrum of compound **28** in DMSO-d_6 at 600 MHz

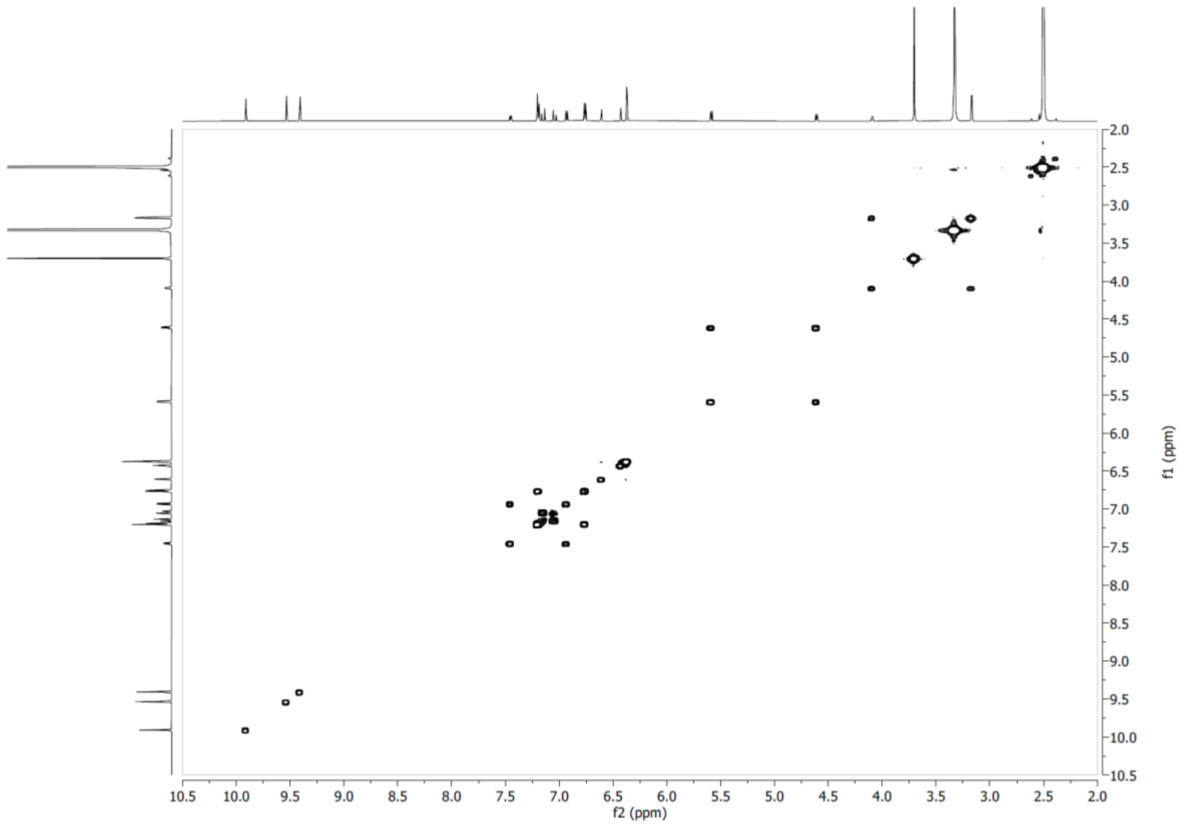

COSY NMR spectrum of compound **28** in DMSO-d_6

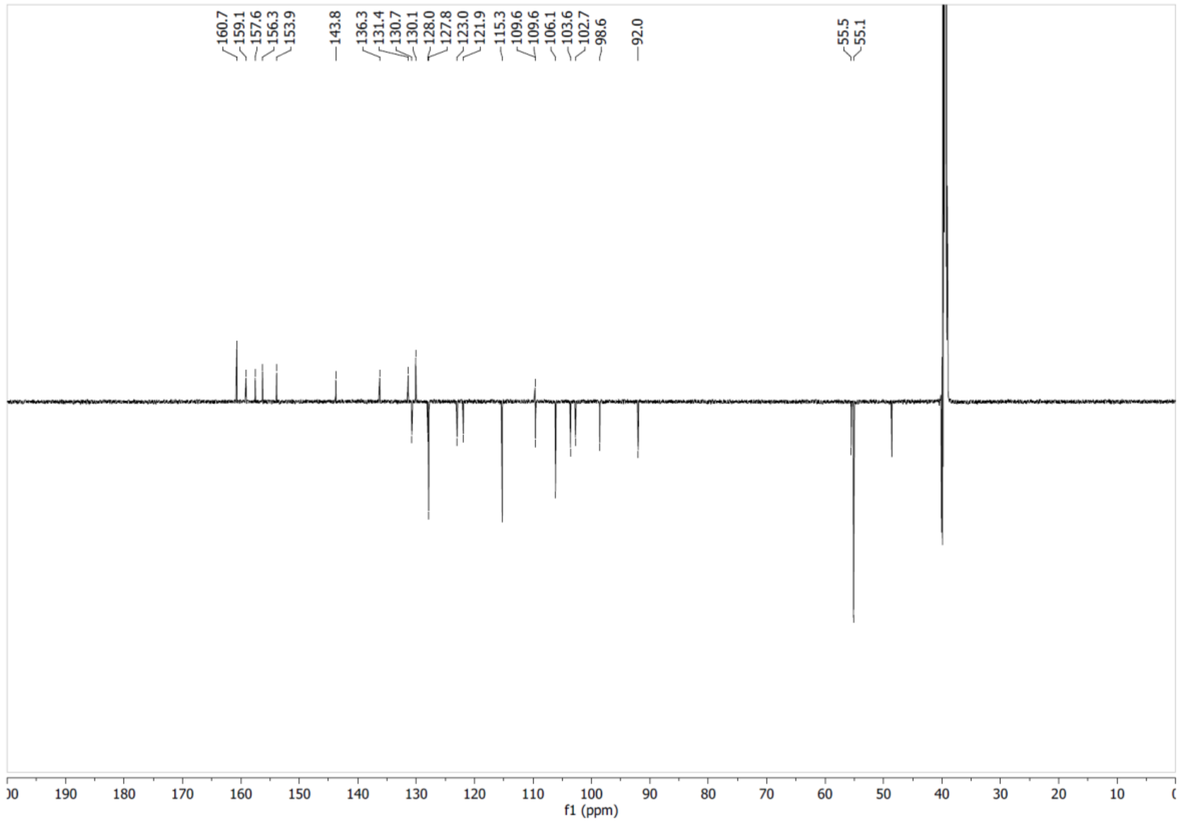

^{13}C-DEPTQ NMR spectrum of compound **28** in DMSO-d_6 at 151 MHz

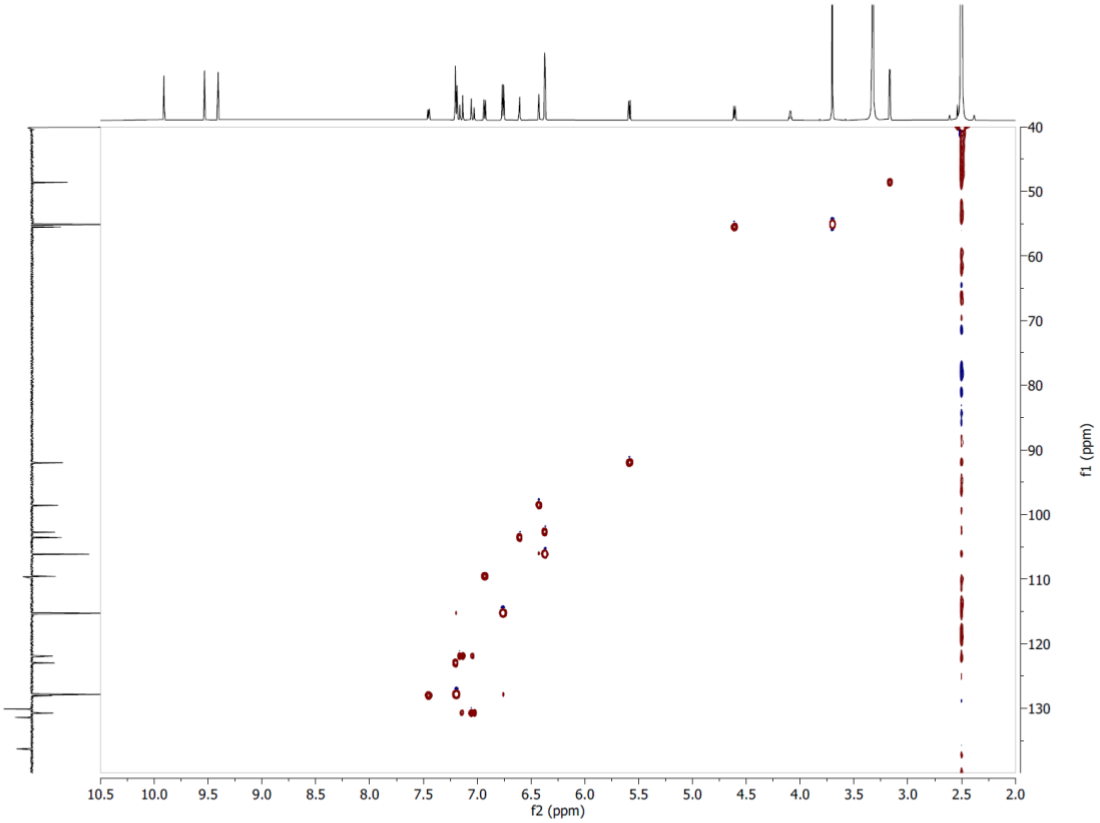

Edited HSQC NMR spectrum of compound **28** in DMSO-d_6

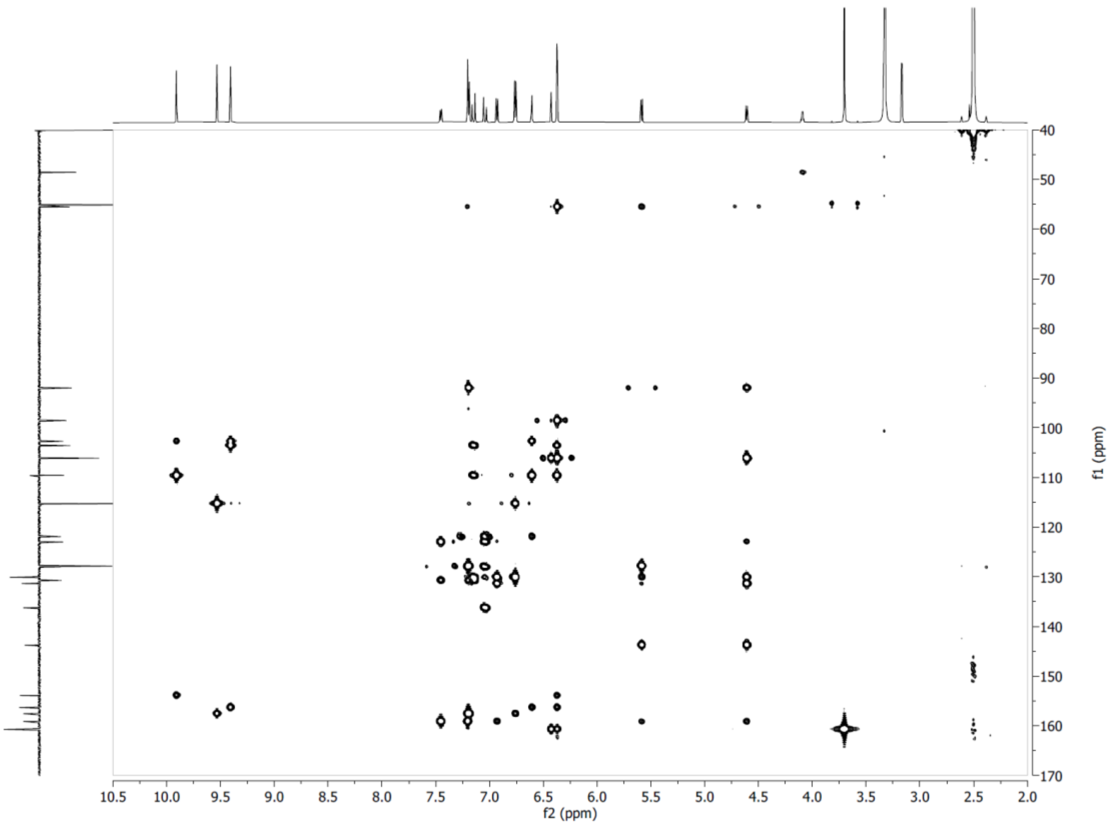

HMBC NMR spectrum of compound **28** in DMSO-d_6

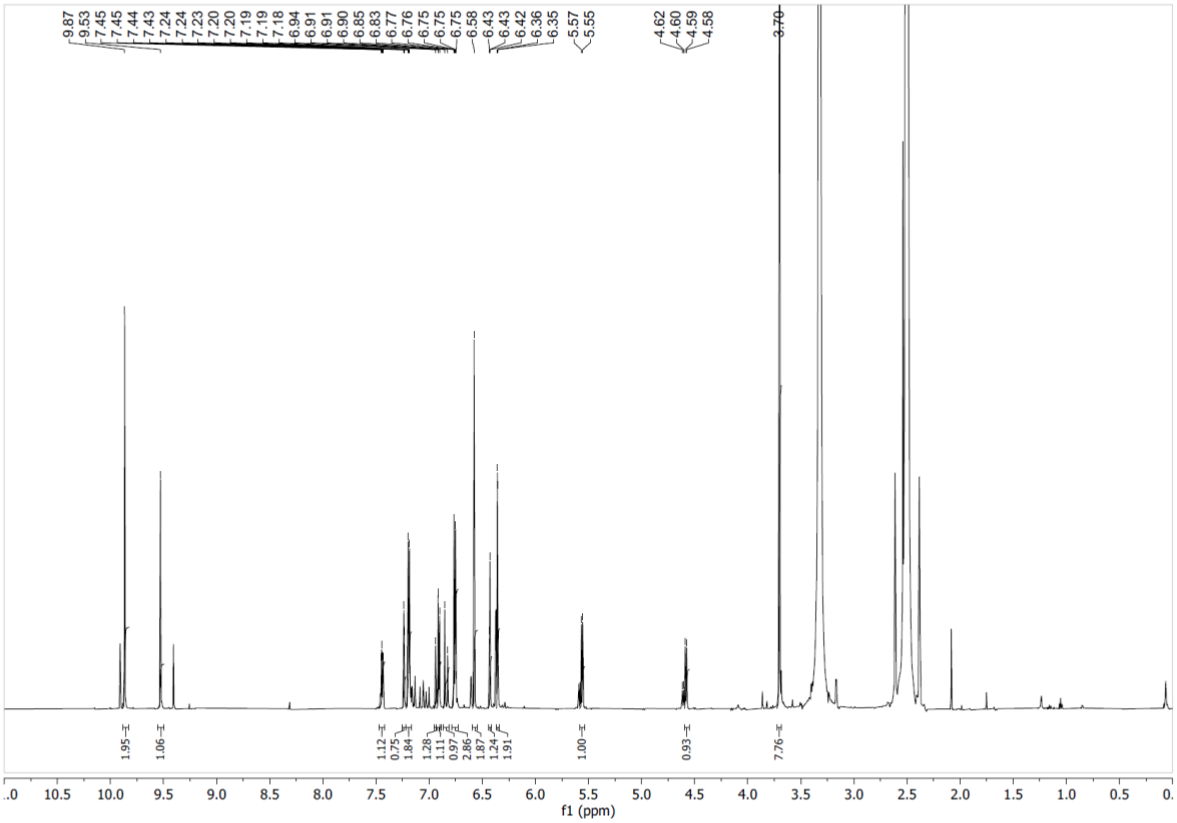

^{1}H NMR spectrum of compound **29** in DMSO-d_6 at 600 MHz

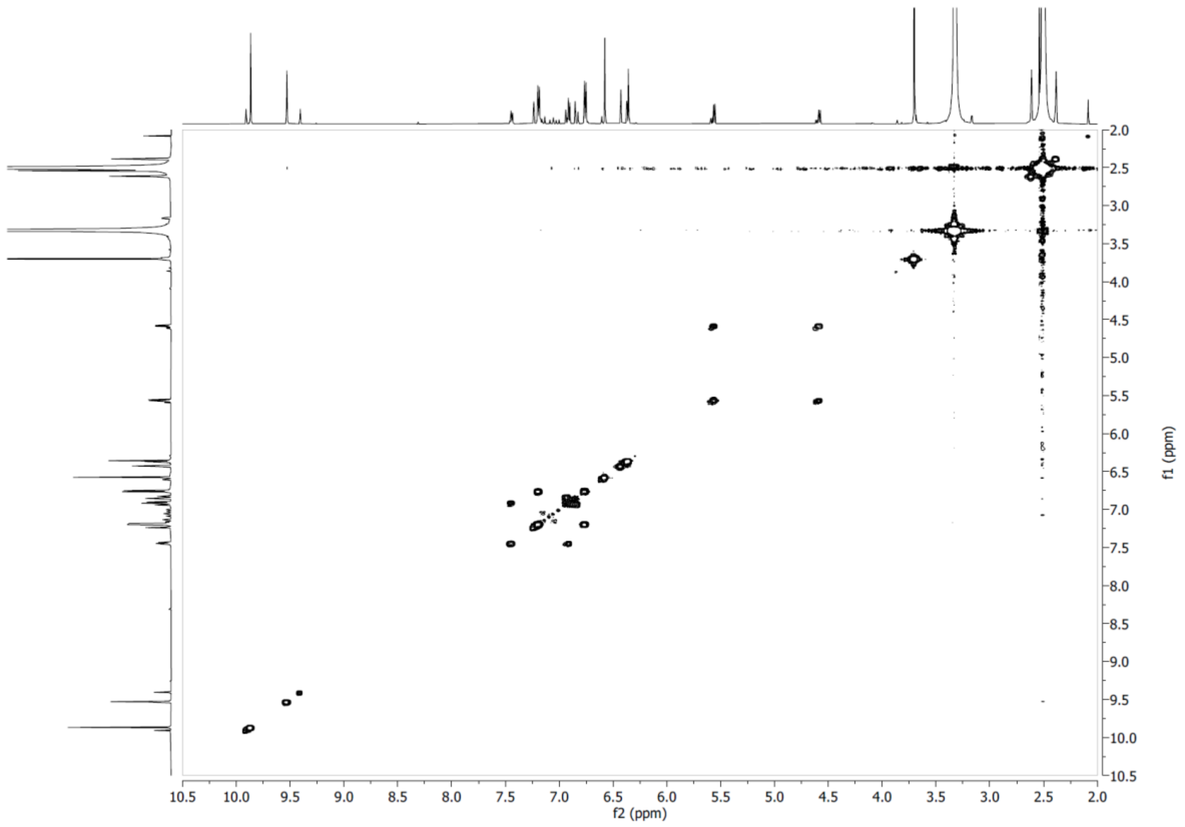

COSY NMR spectrum of compound **29** in DMSO-d_6

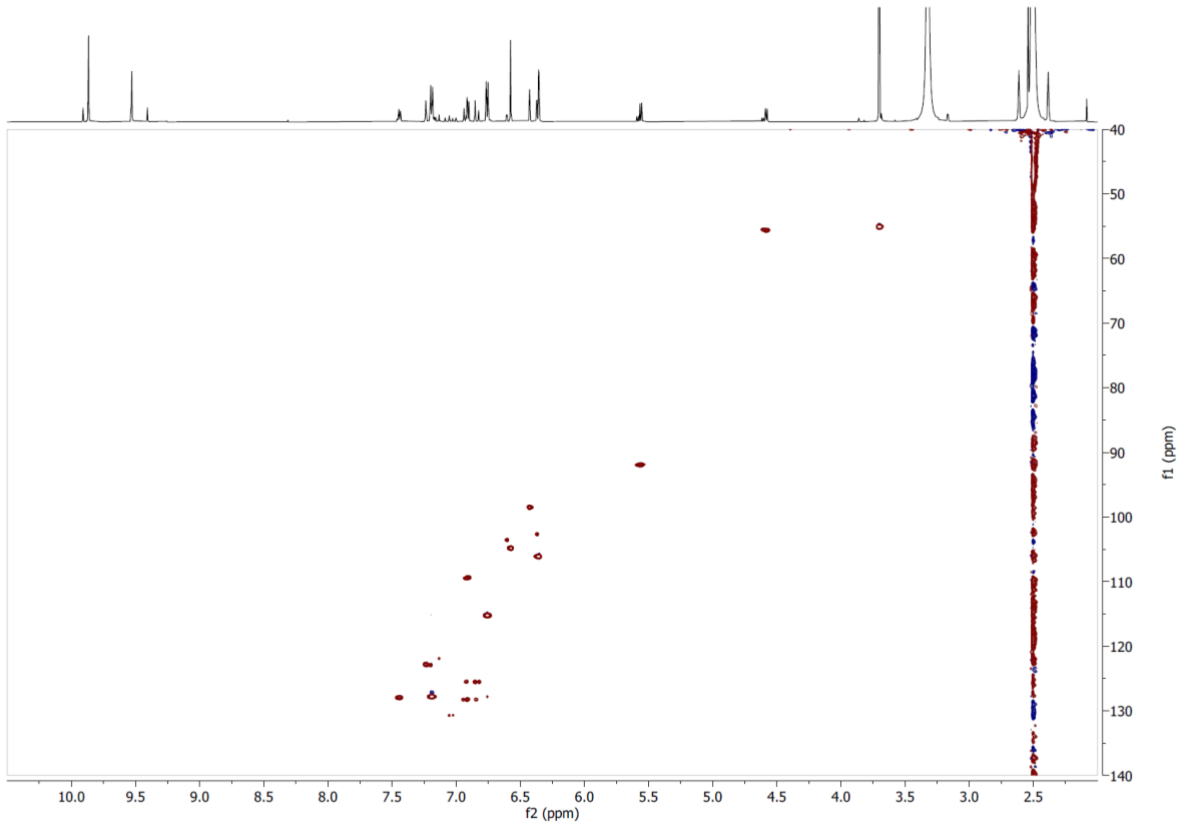

Edited HSQC NMR spectrum of compound **29** in DMSO-d_6

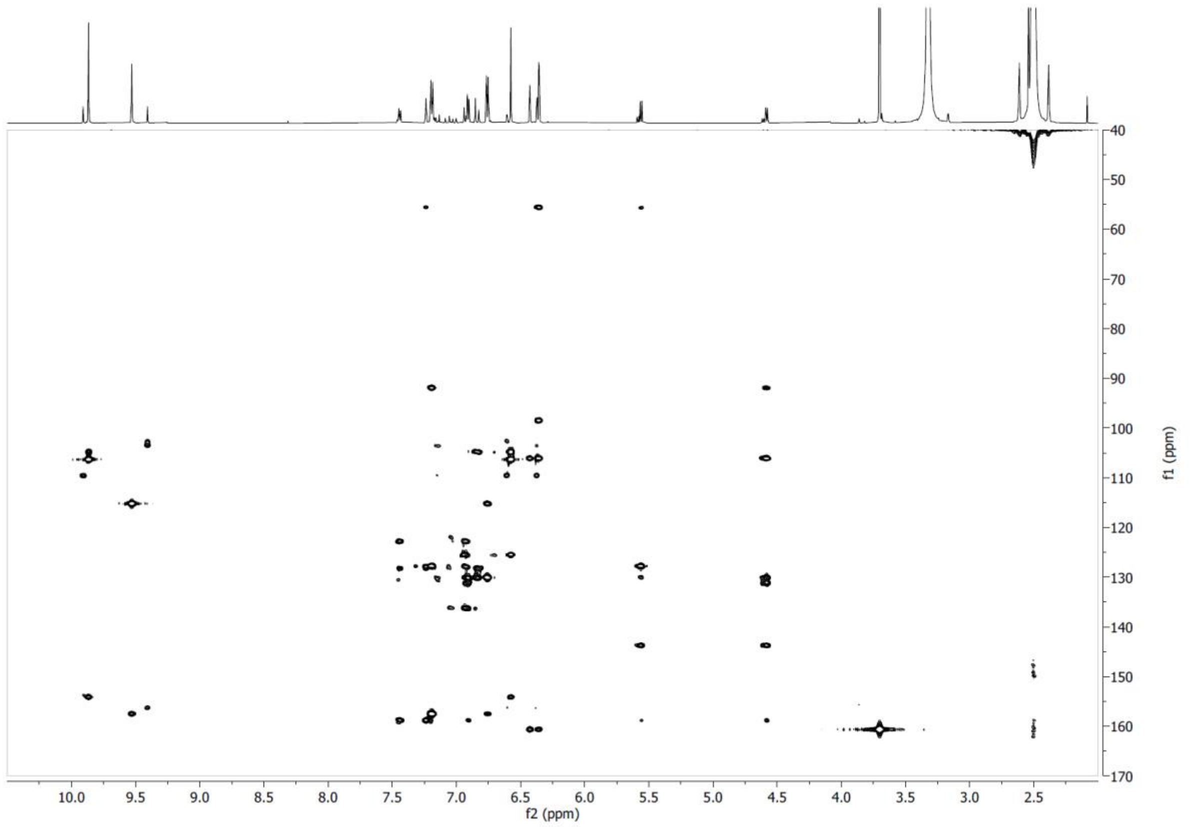

HMBC NMR spectrum of compound **29** in DMSO-d_6

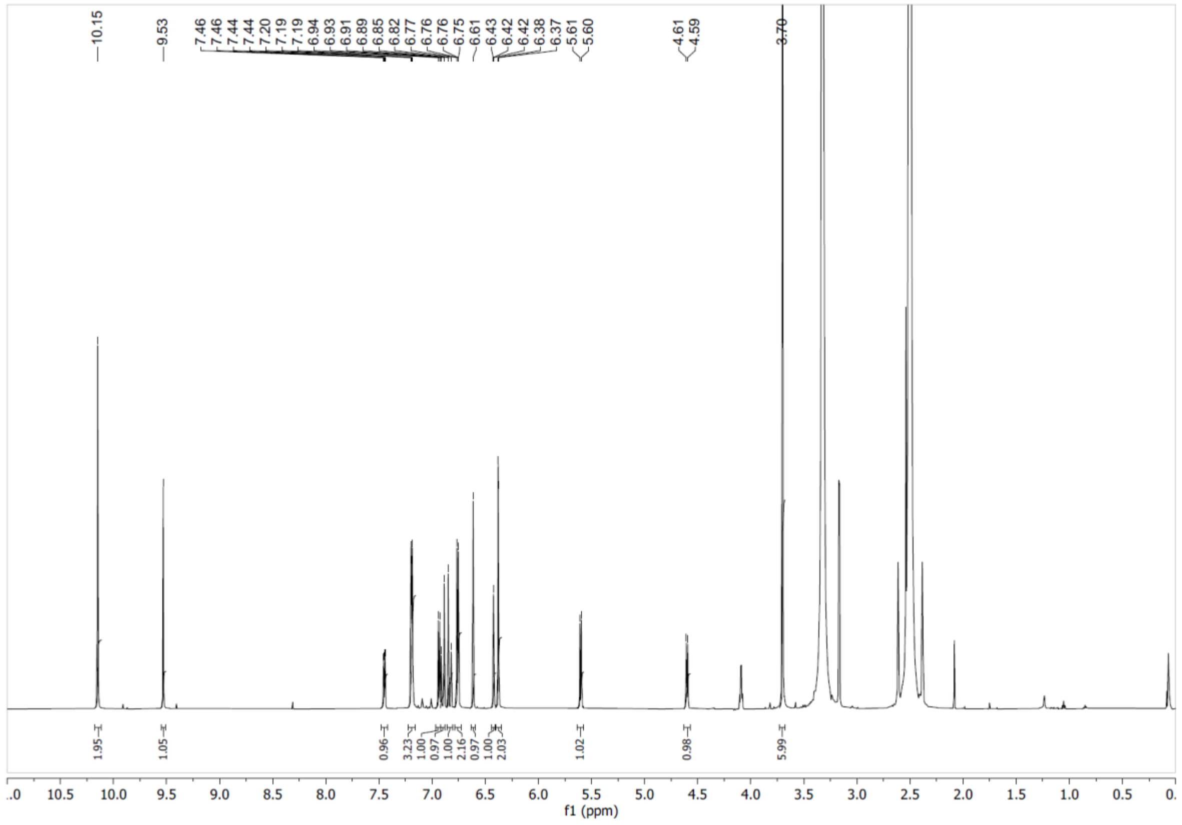

^{1}H NMR spectrum of compound **30** in DMSO-d_6 at 600 MHz

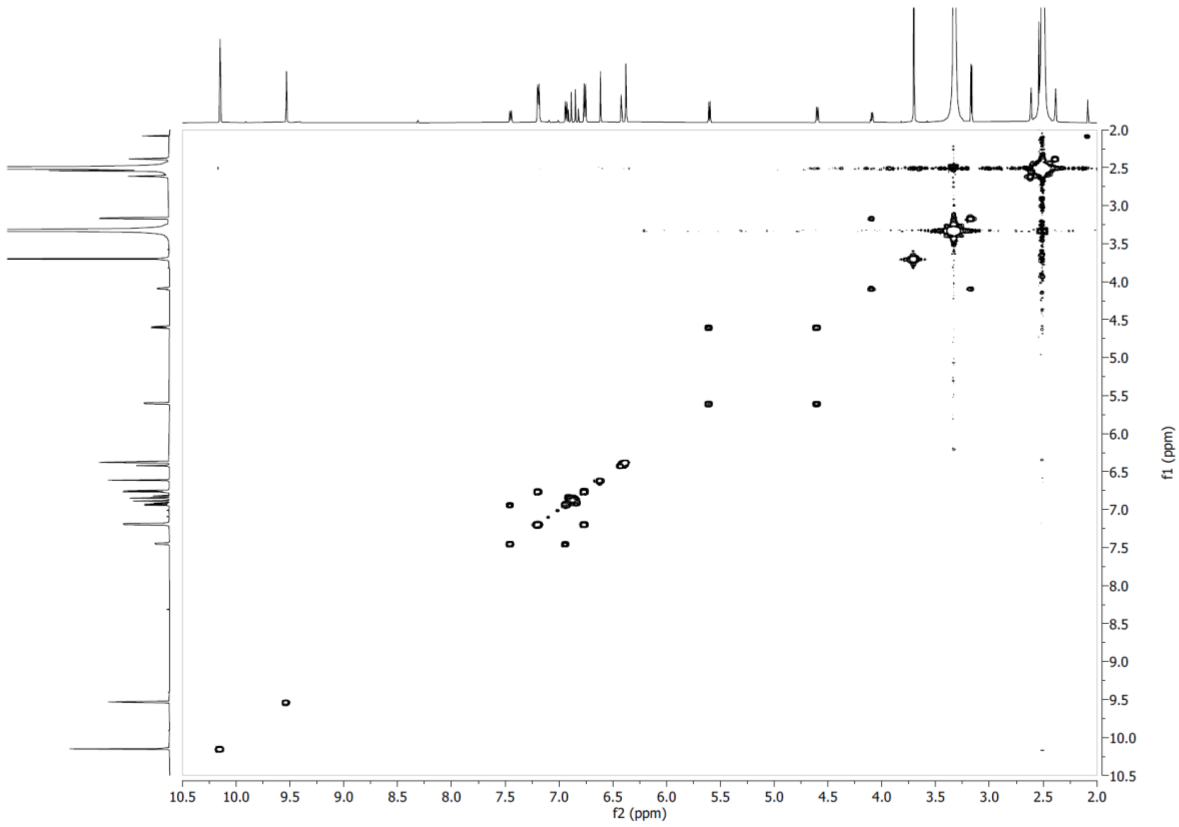

COSY NMR spectrum of compound **30** in DMSO-d_6

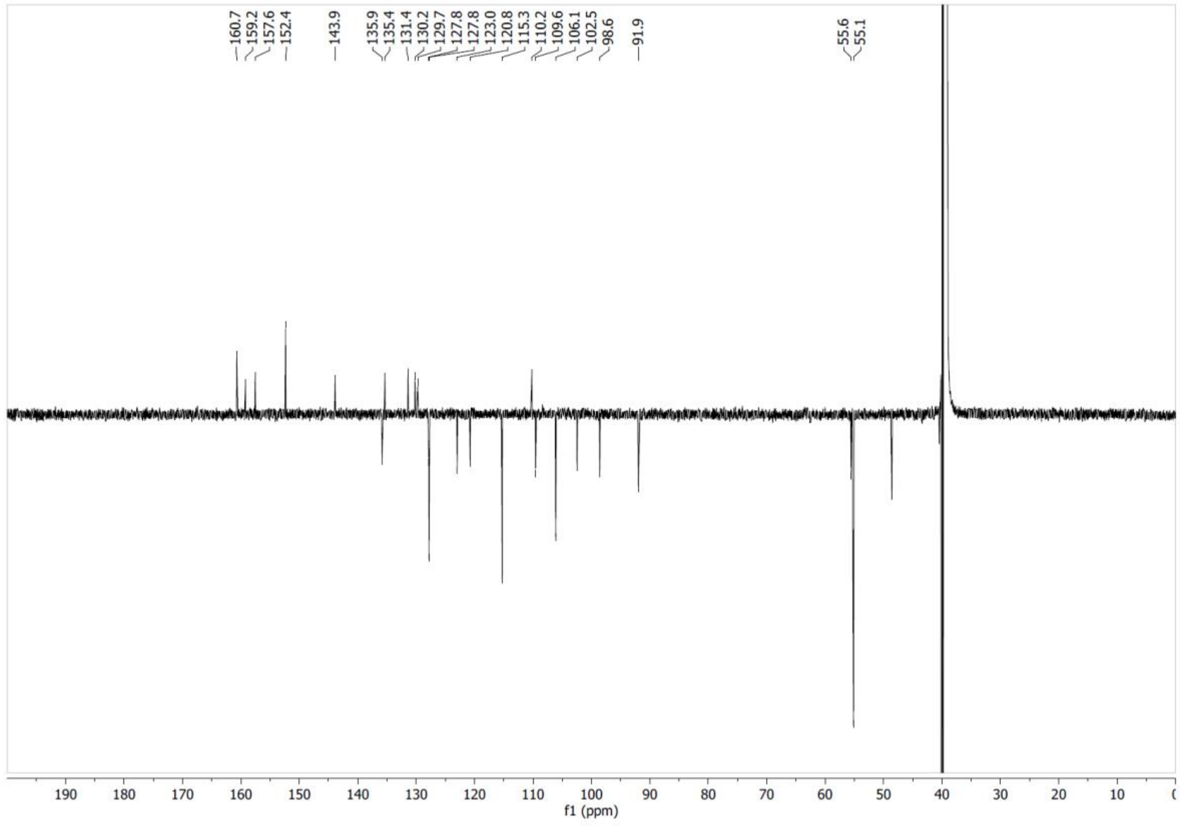

^{13}C-DEPTQ NMR spectrum of compound **30** in DMSO-d_6 at 151 MHz

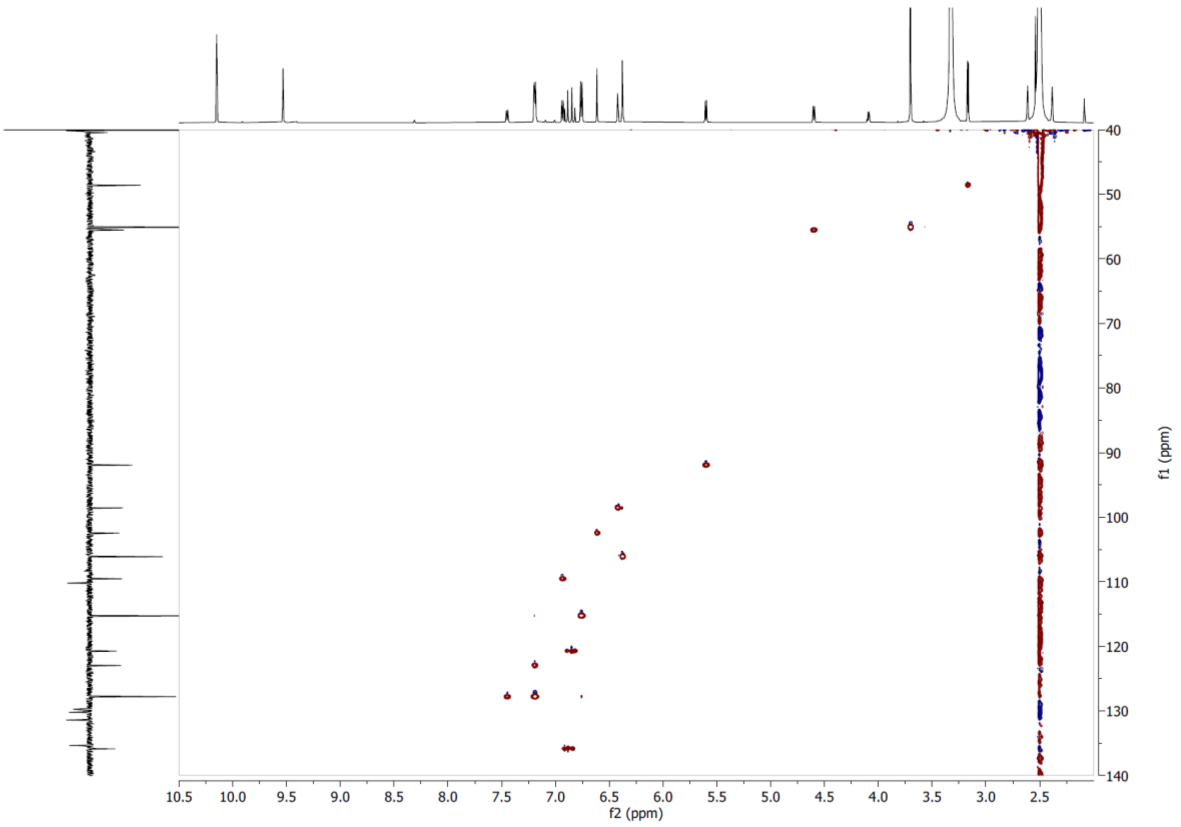

Edited HSQC NMR spectrum of compound **30** in DMSO-d_6

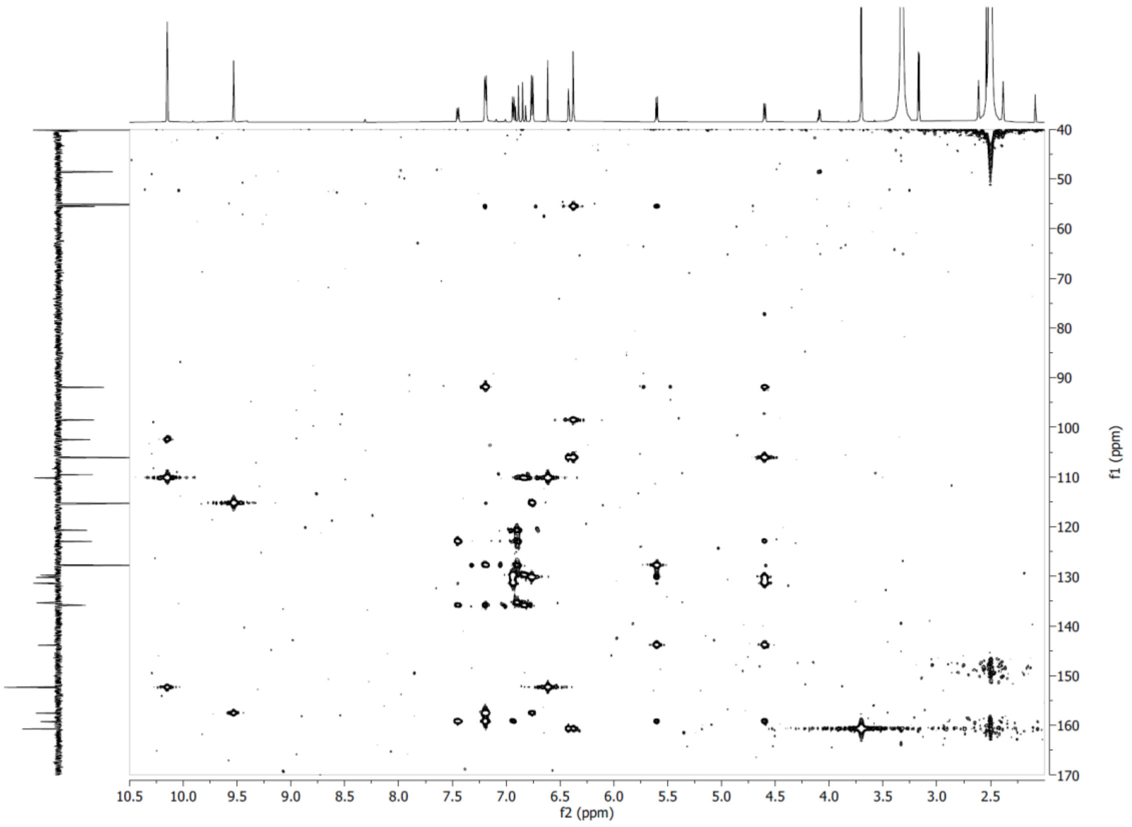

HMBC NMR spectrum of compound **30** in DMSO-d_6

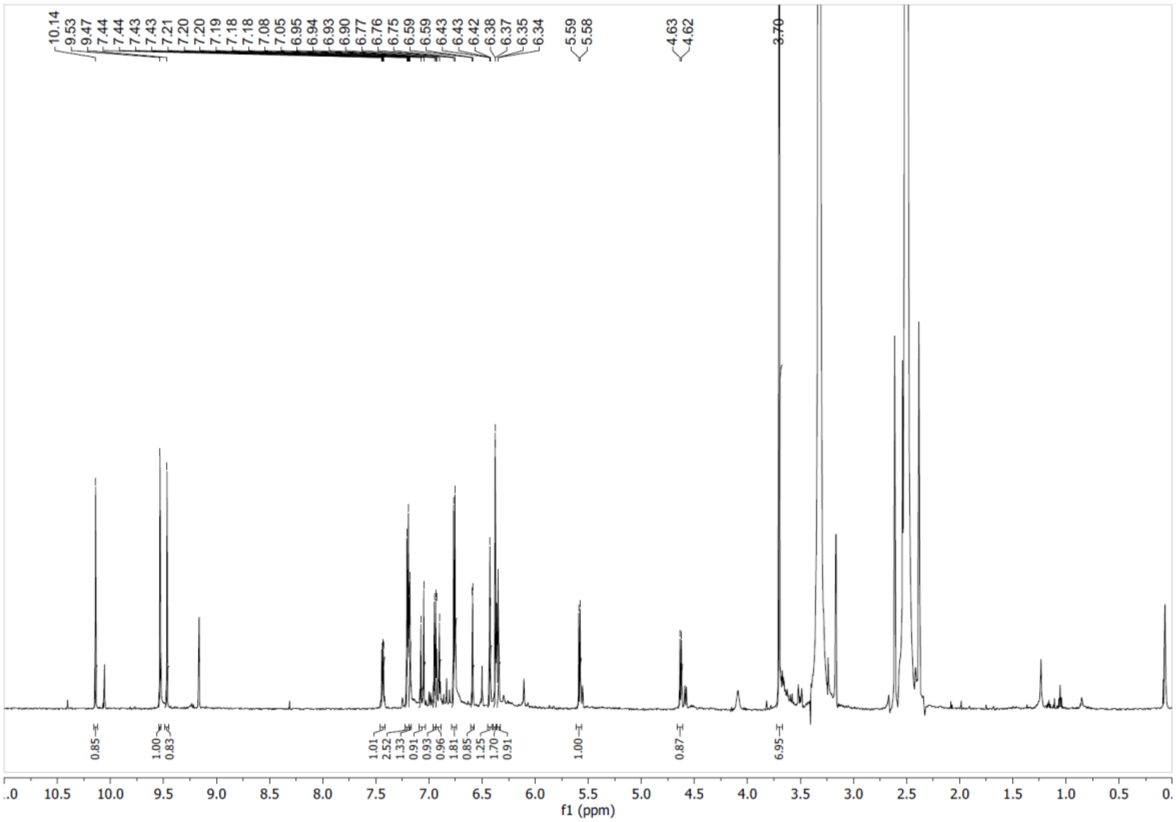

^{1}H NMR spectrum of compound **31** in DMSO-d_6 at 600 MHz

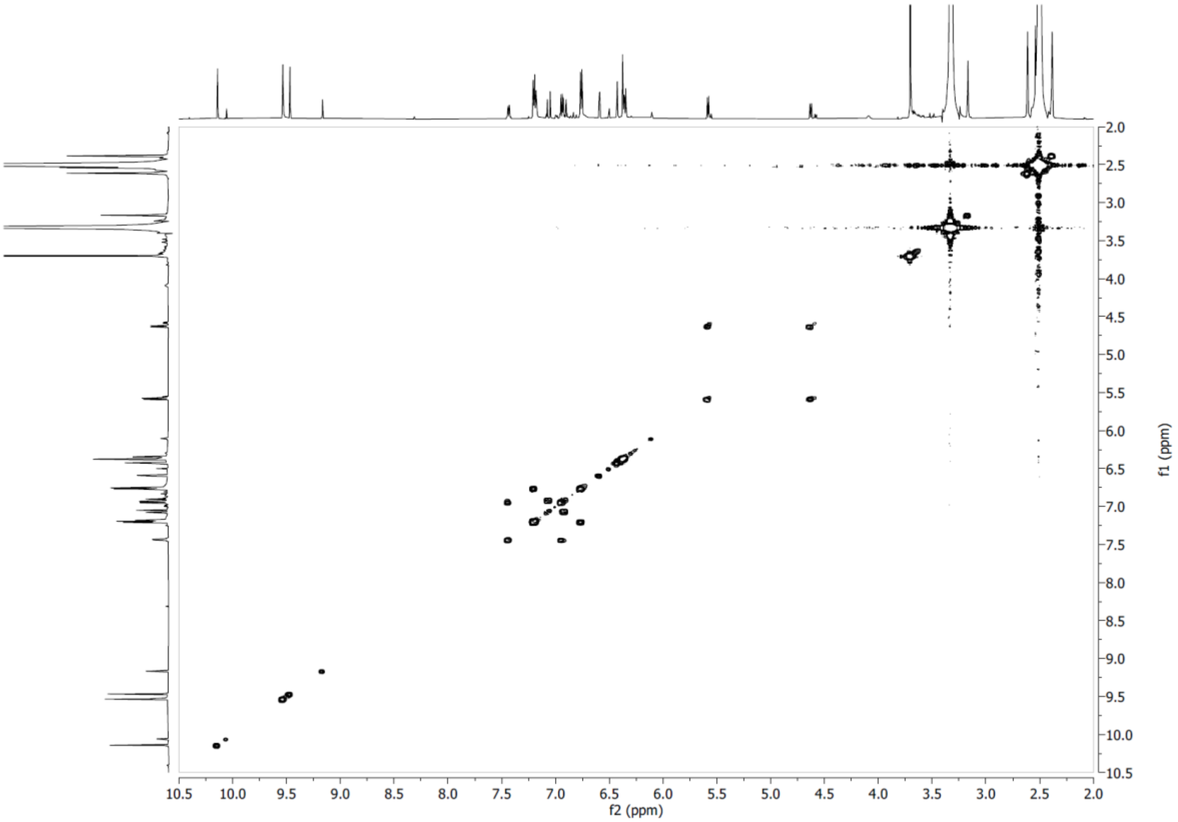

COSY NMR spectrum of compound **31** in DMSO-d_6

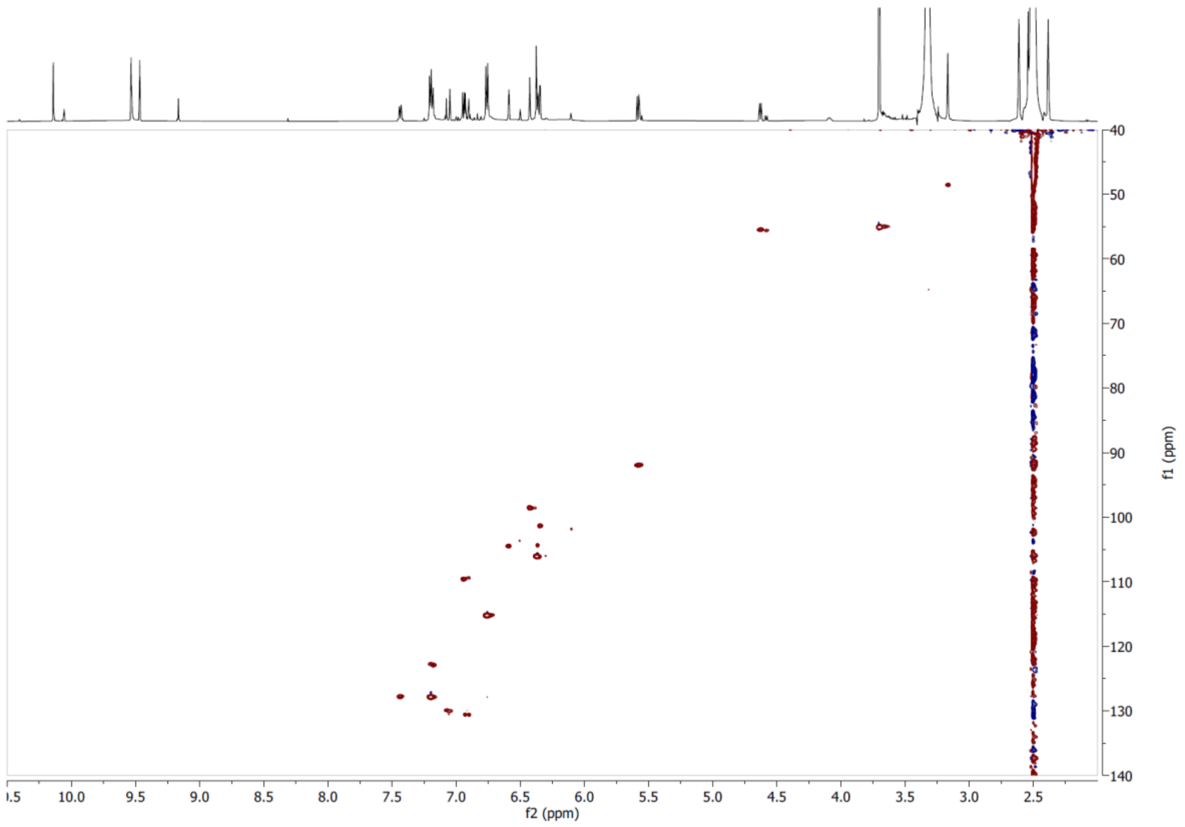

Edited HSQC NMR spectrum of compound **31** in DMSO-d_6

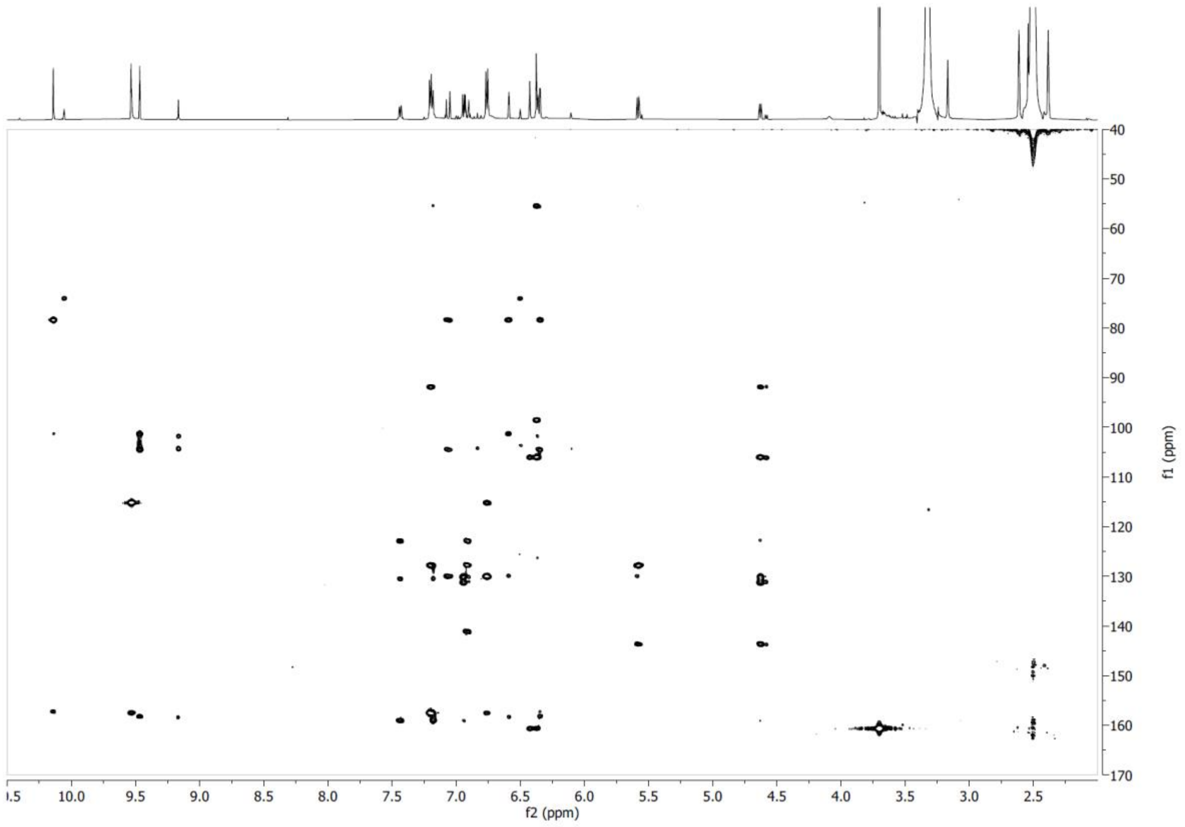

HMBC NMR spectrum of compound **31** in DMSO-d_6

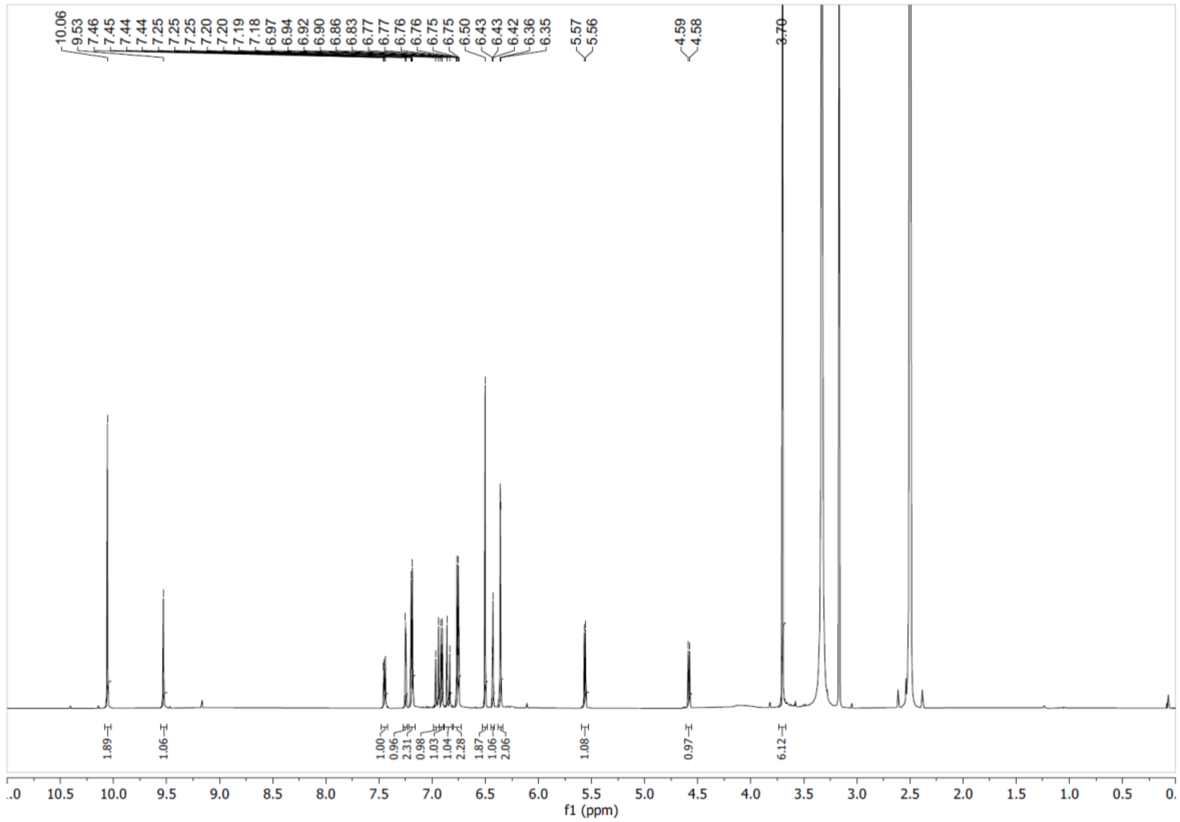

^{1}H NMR spectrum of compound **32** in DMSO-d_6 at 600 MHz

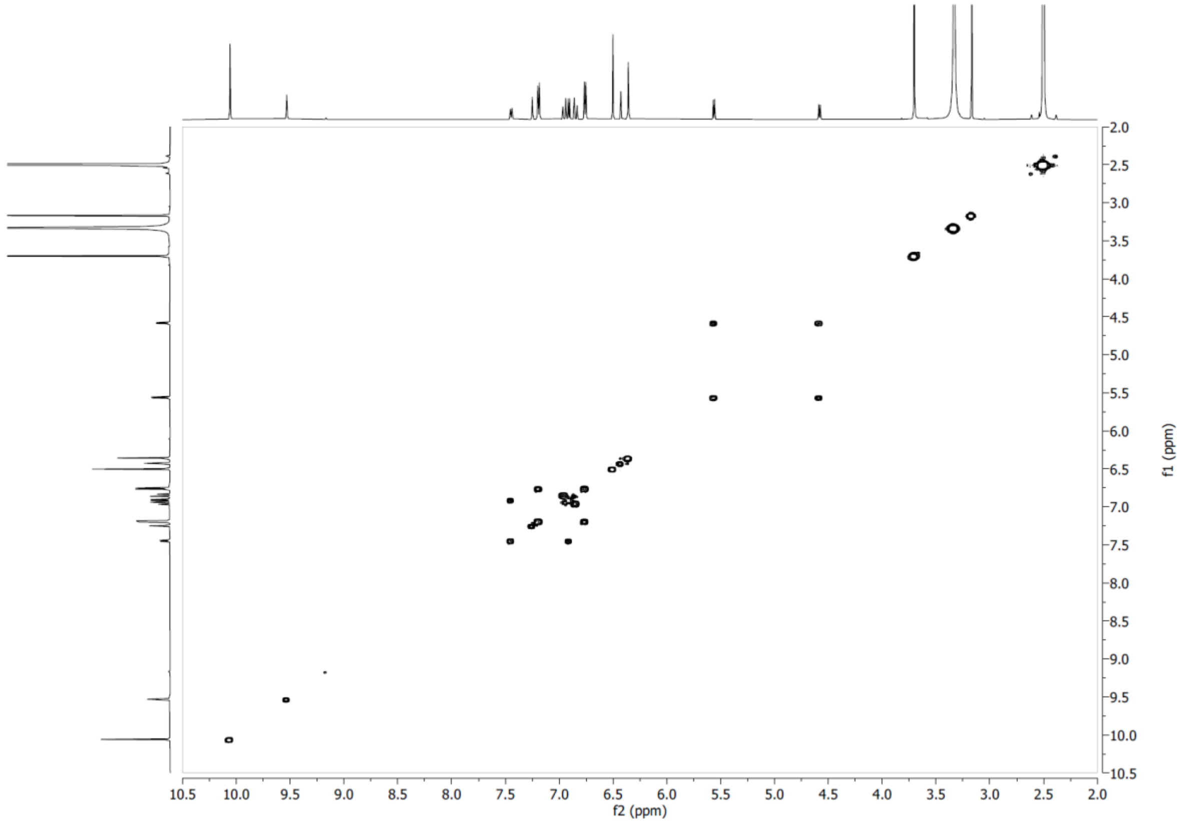

COSY NMR spectrum of compound **32** in DMSO-d_6

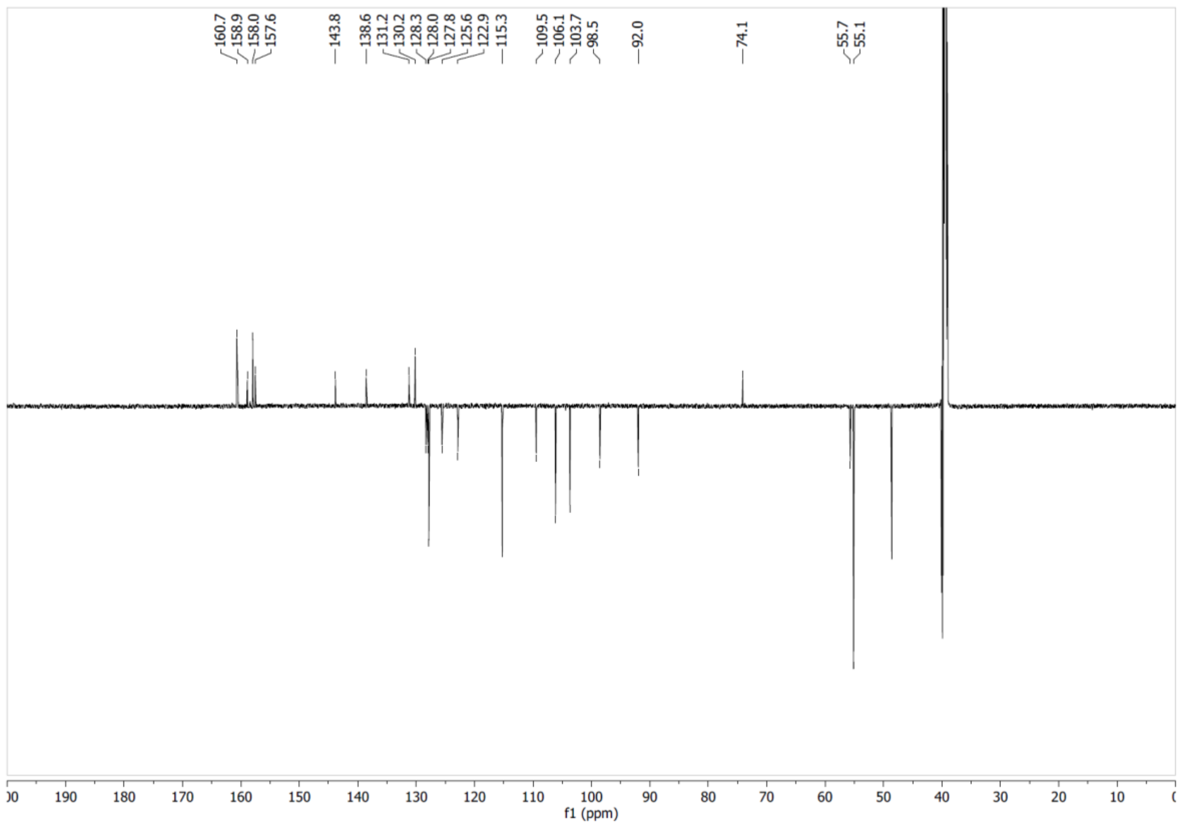

^{13}C-DEPTQ NMR spectrum of compound **32** in DMSO-d_6 at 151 MHz

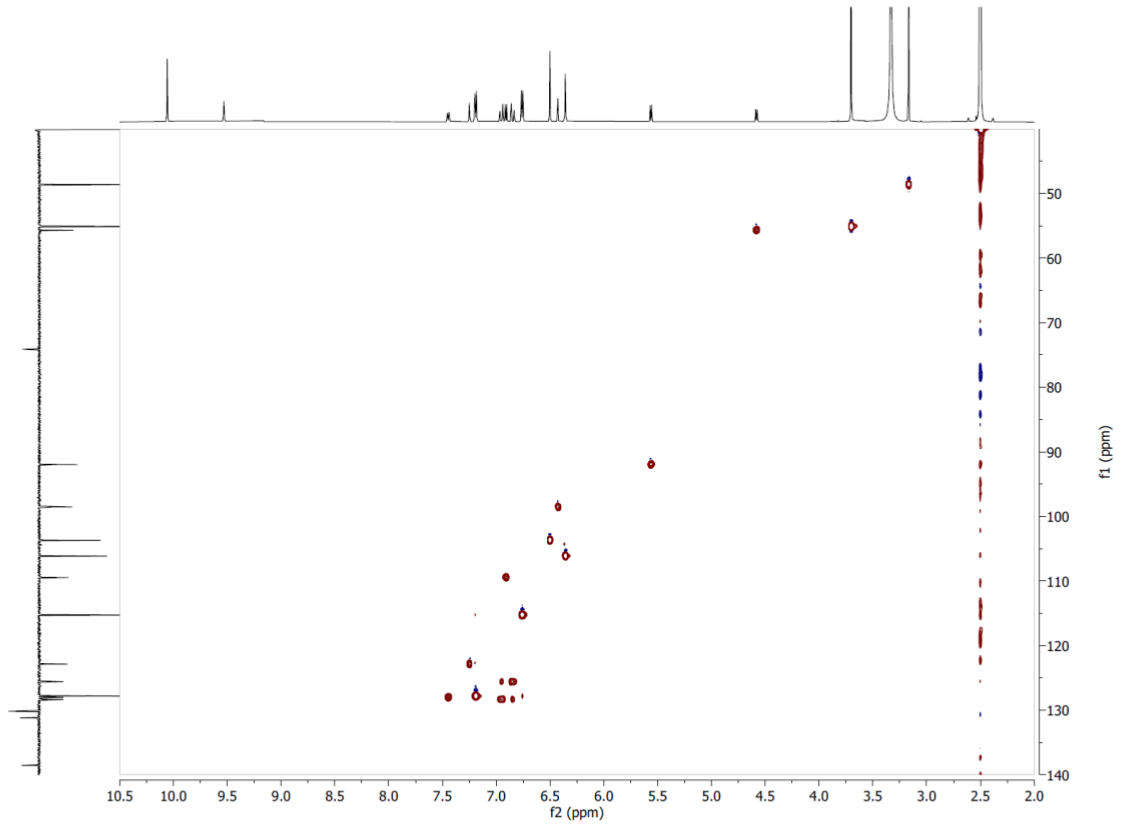

Edited HSQC NMR spectrum of compound **32** in DMSO-d_6

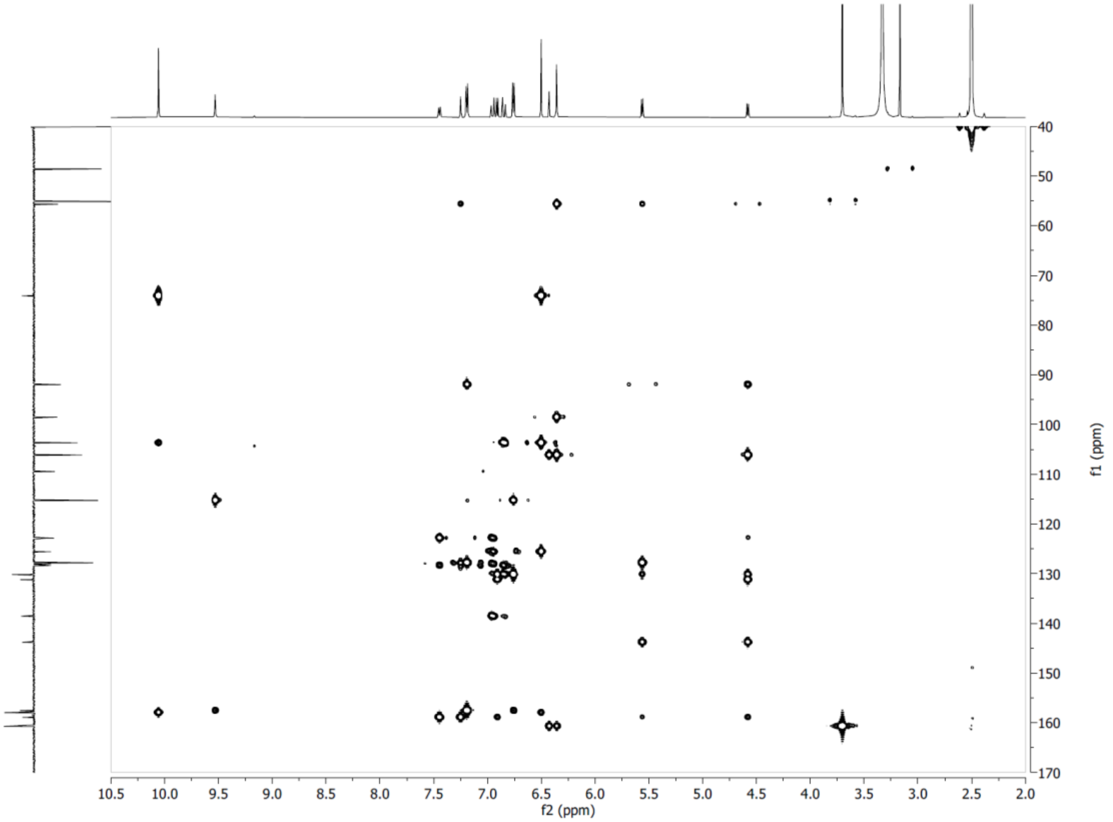

HMBC NMR spectrum of compound **32** in DMSO-d_6

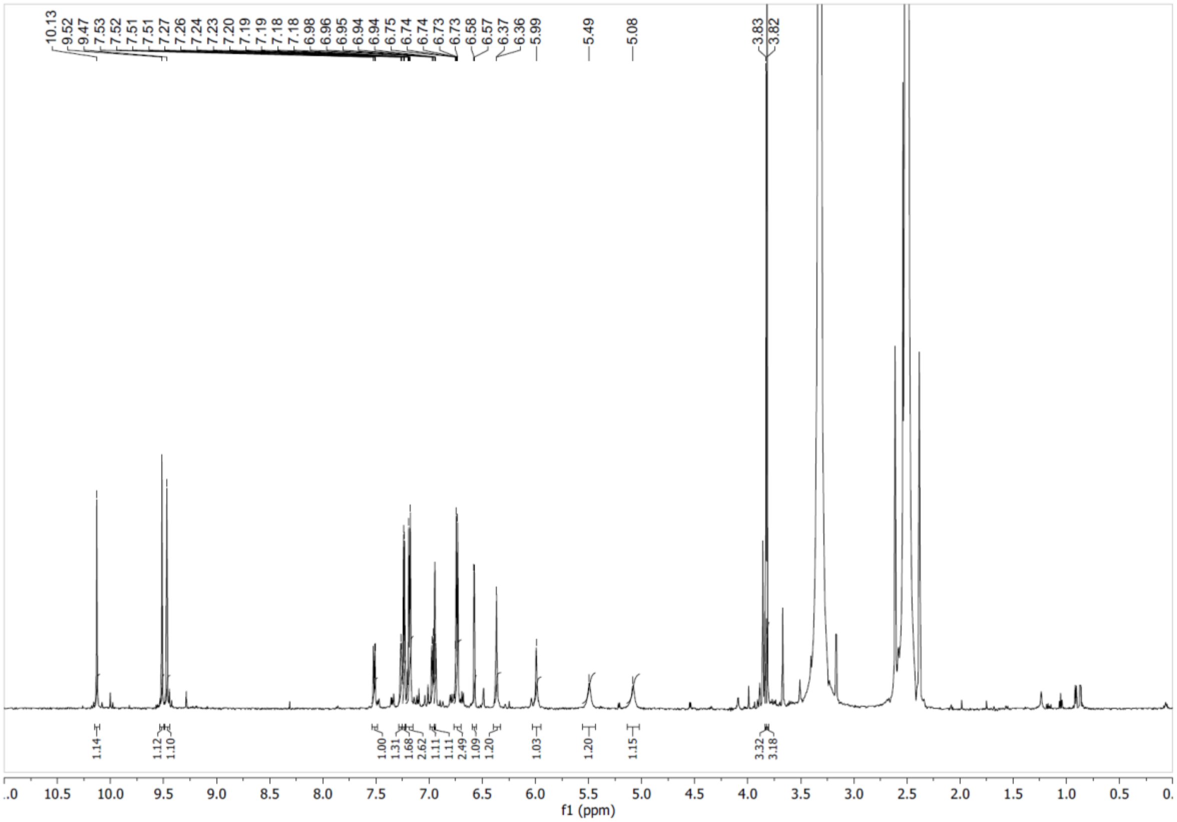

^{1}H NMR spectrum of compound **33** in DMSO-d_6 at 600 MHz

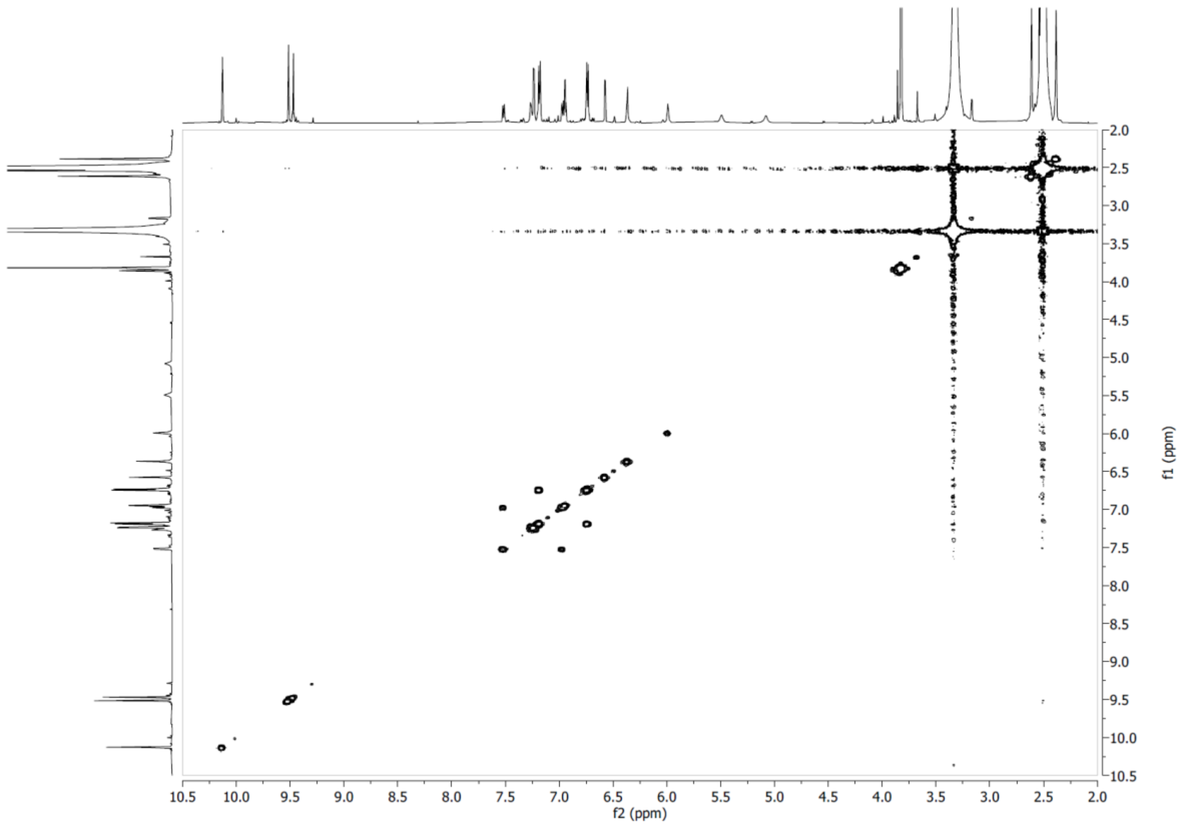

COSY NMR spectrum of compound **33** in DMSO-*d*6

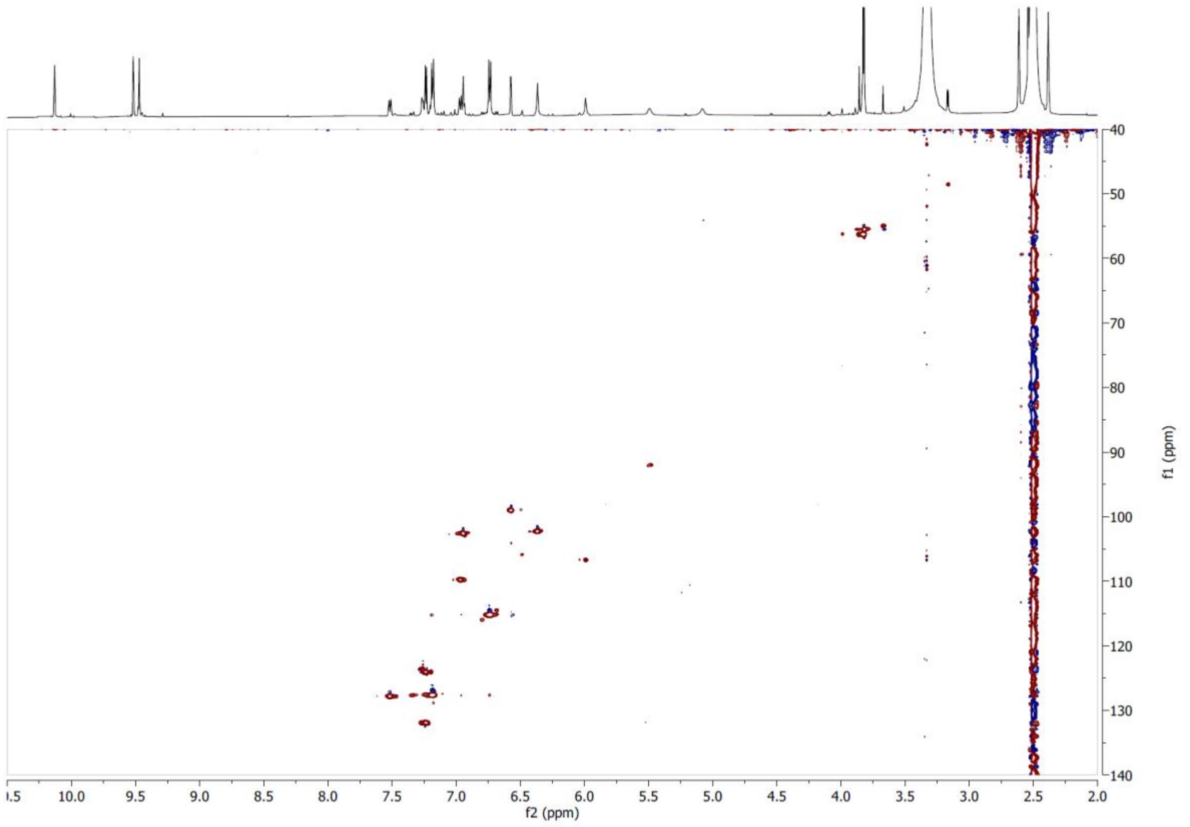

Edited HSQC NMR spectrum of compound **33** in DMSO-*d*6

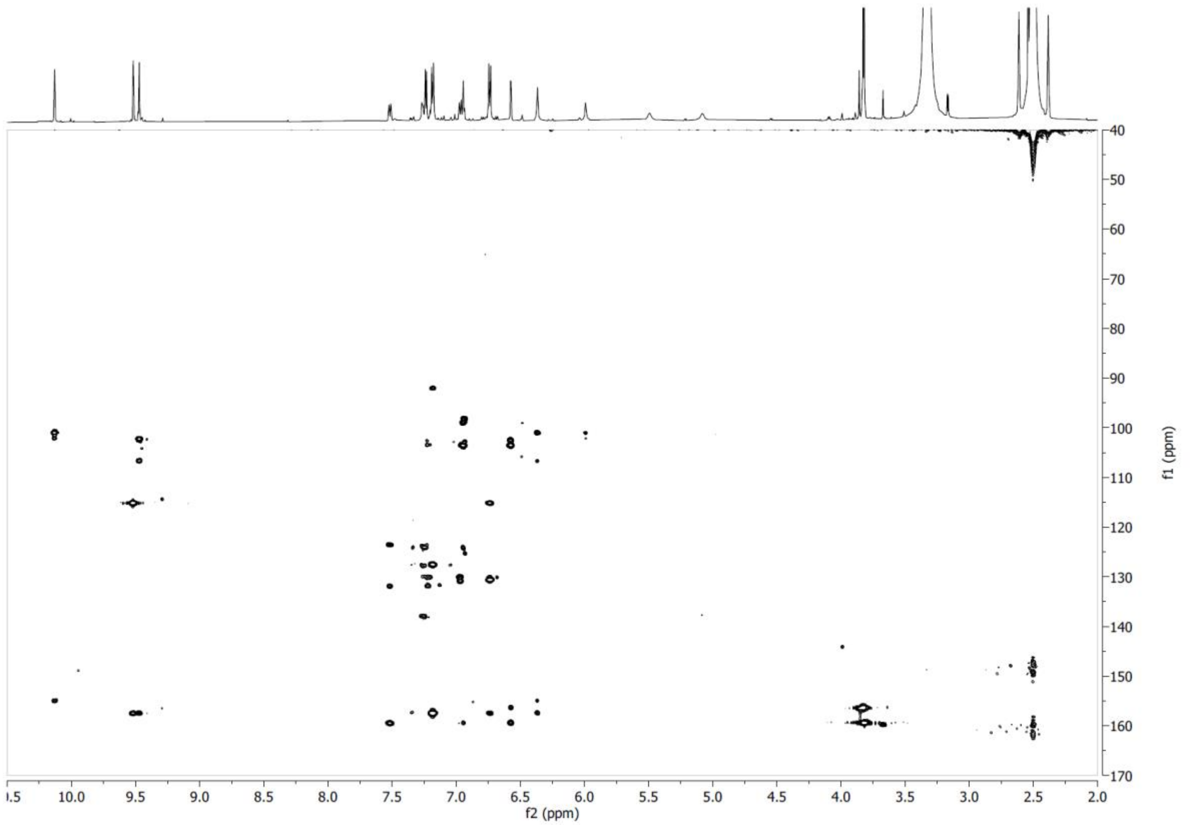

HMBC NMR spectrum of compound **33** in DMSO-d_6

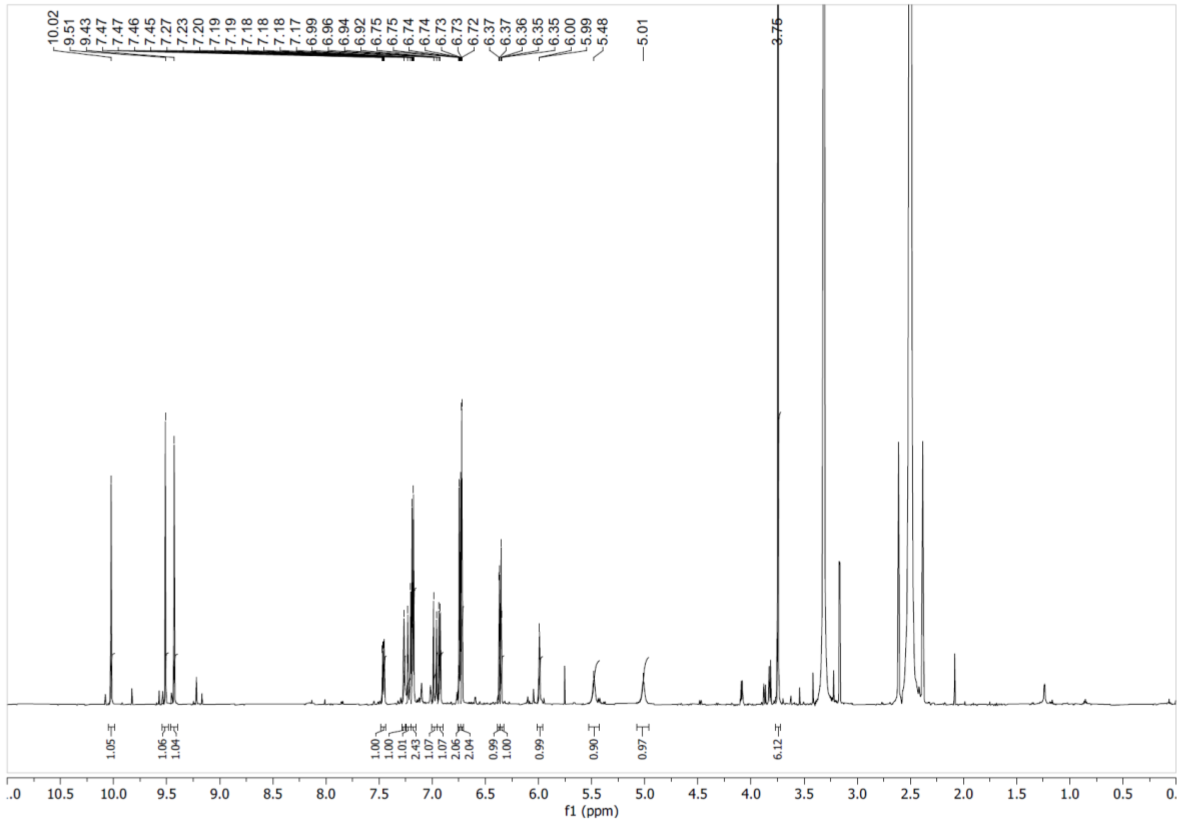

^{1}H NMR spectrum of compound **34** in DMSO-d_6 at 600 MHz

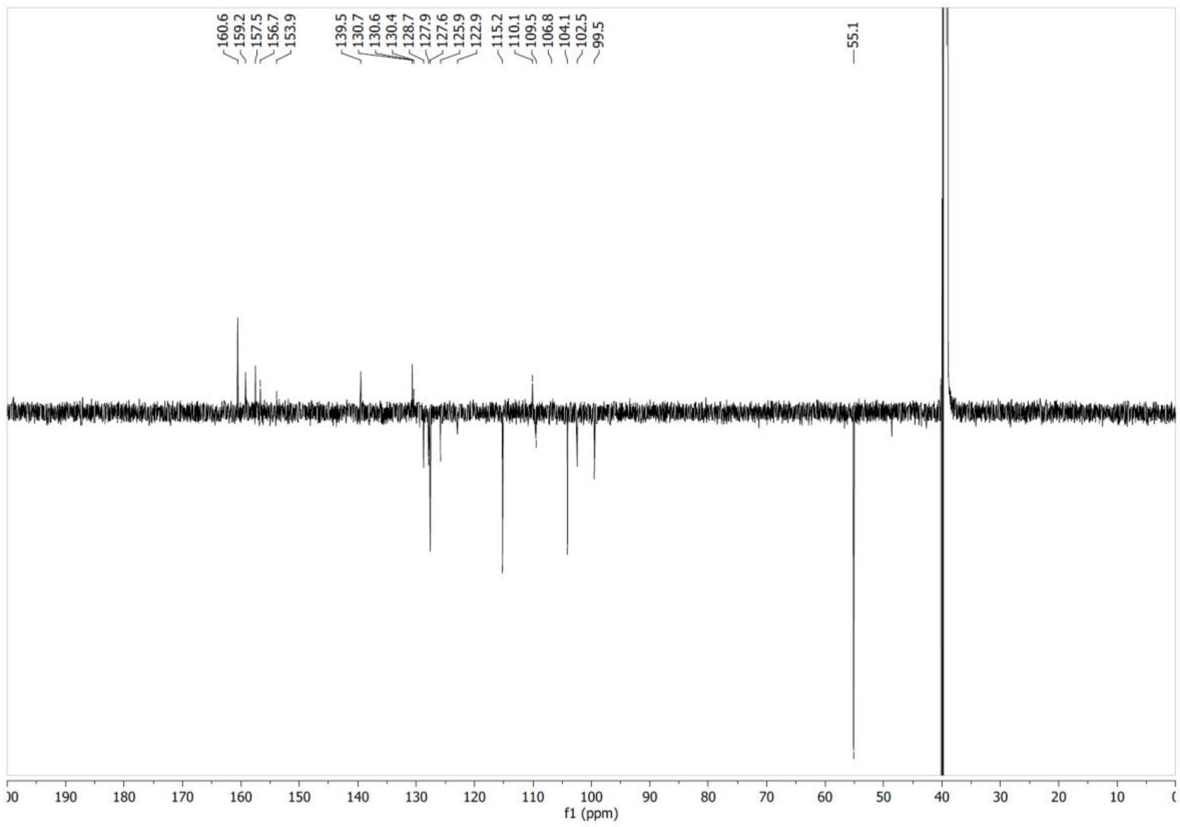

^{13}C-DEPTQ NMR spectrum of compound **34** in DMSO-d_6 at 151 MHz

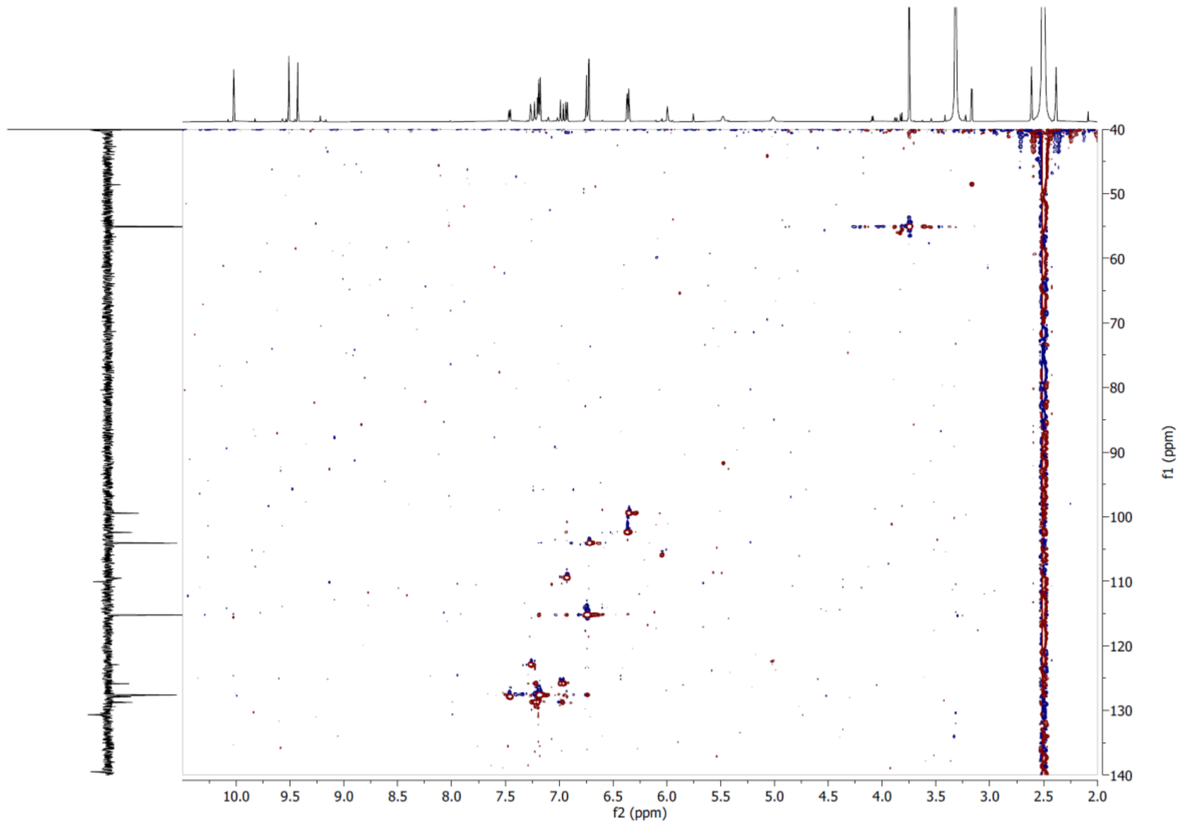

Edited HSQC NMR spectrum of compound **34** in DMSO-d_6

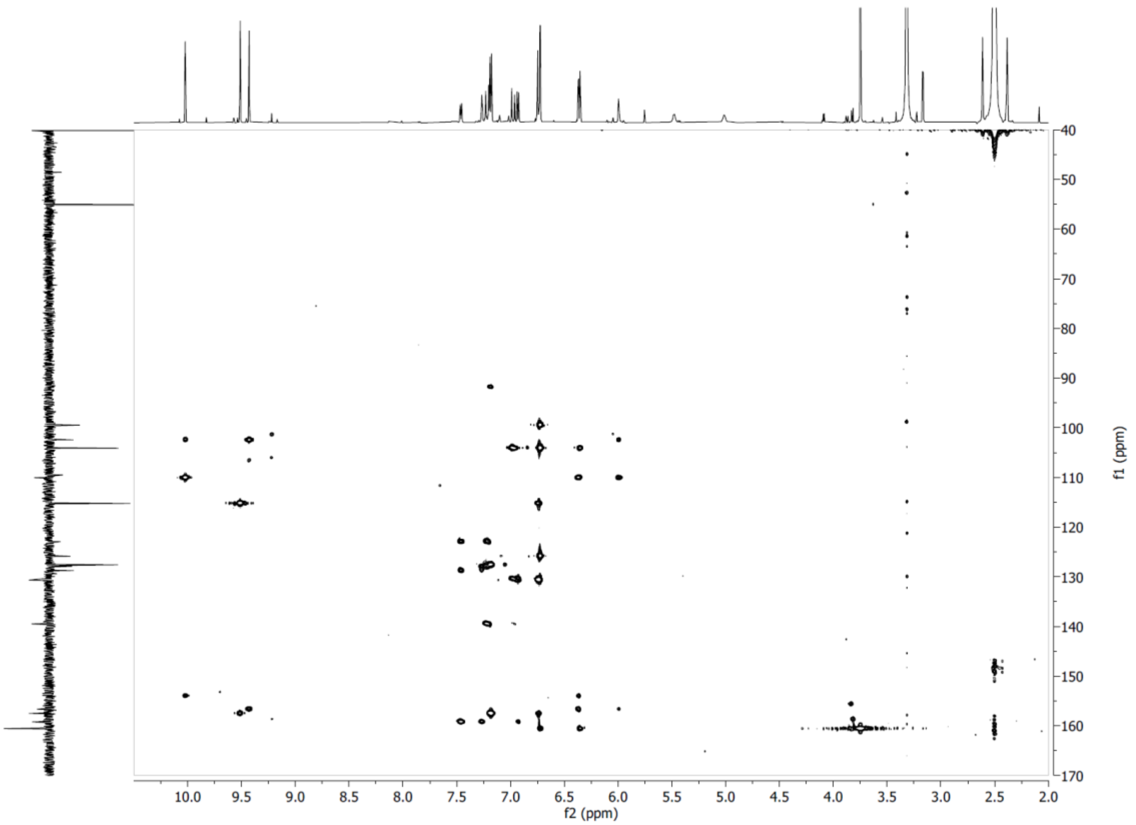

HMBC NMR spectrum of compound **34** in DMSO-d_6

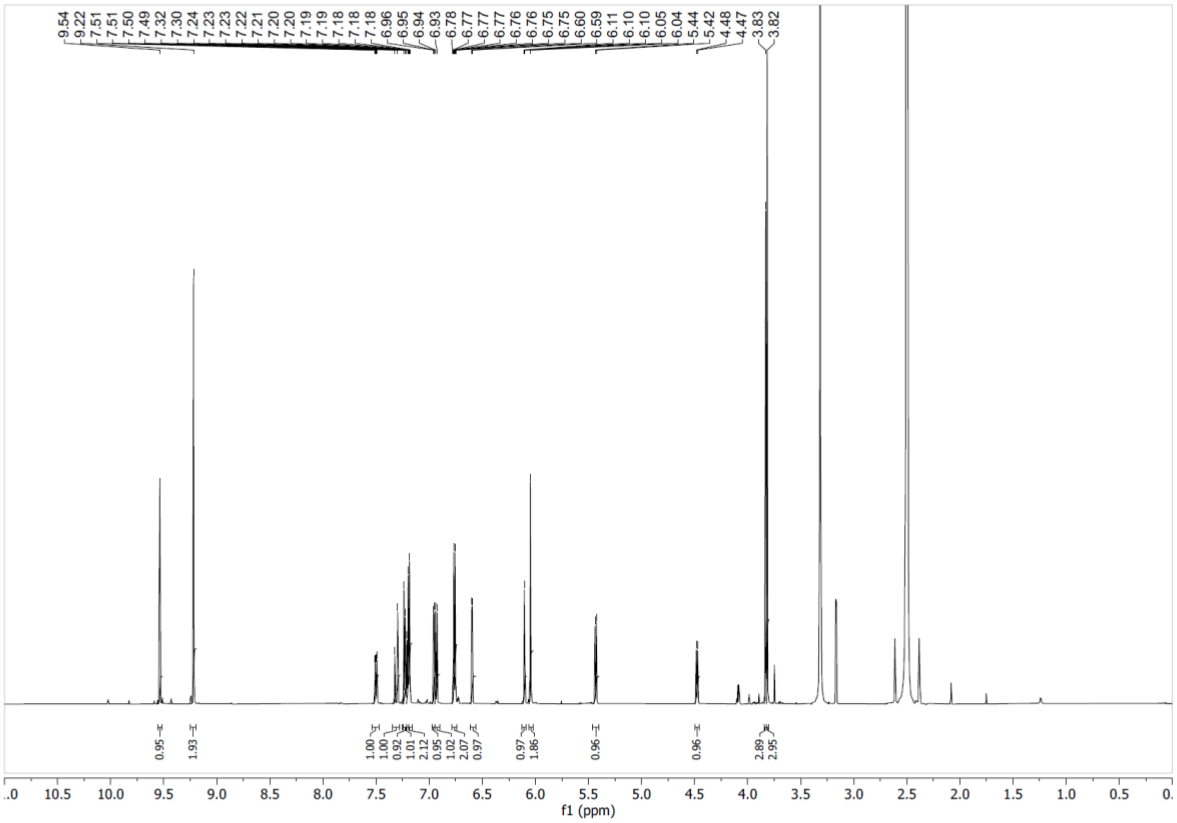

^{1}H NMR spectrum of compound **35** in DMSO-d_6 at 600 MHz

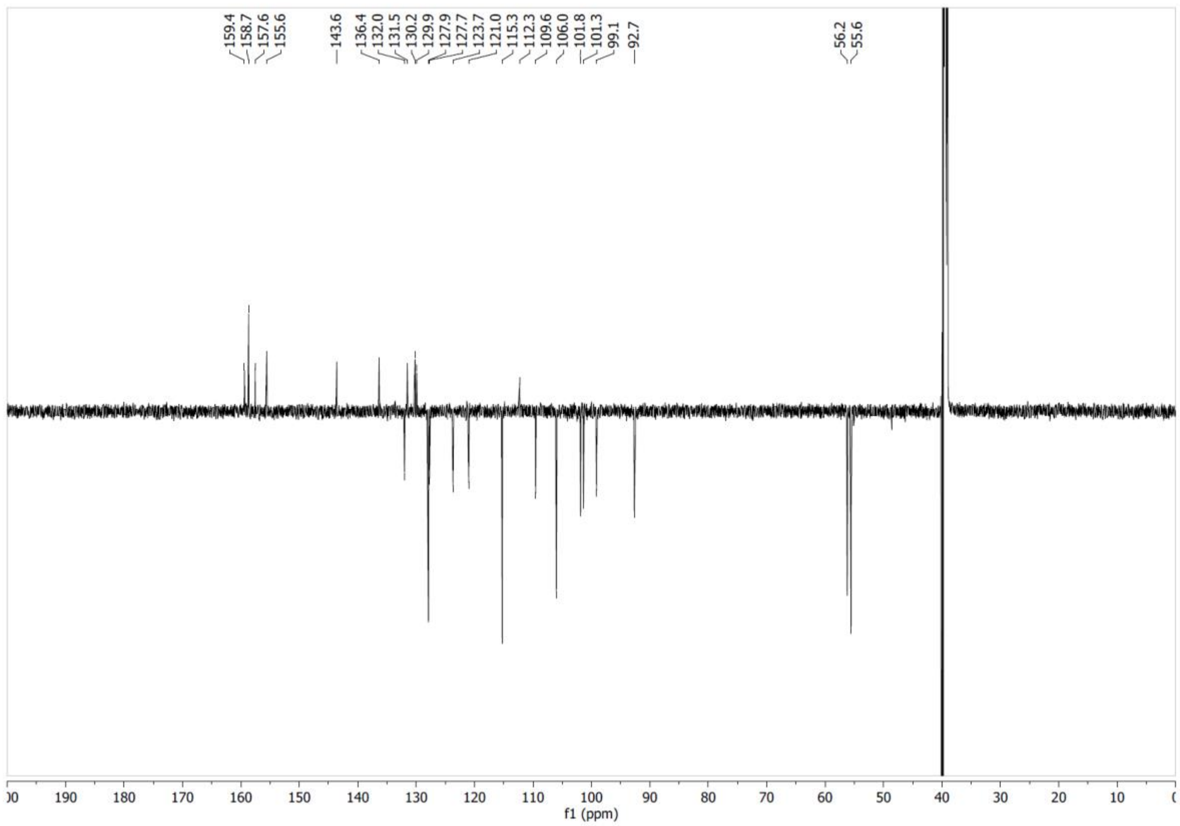

^{13}C-DEPTQ NMR spectrum of compound **35** in DMSO-d_6 at 151 MHz

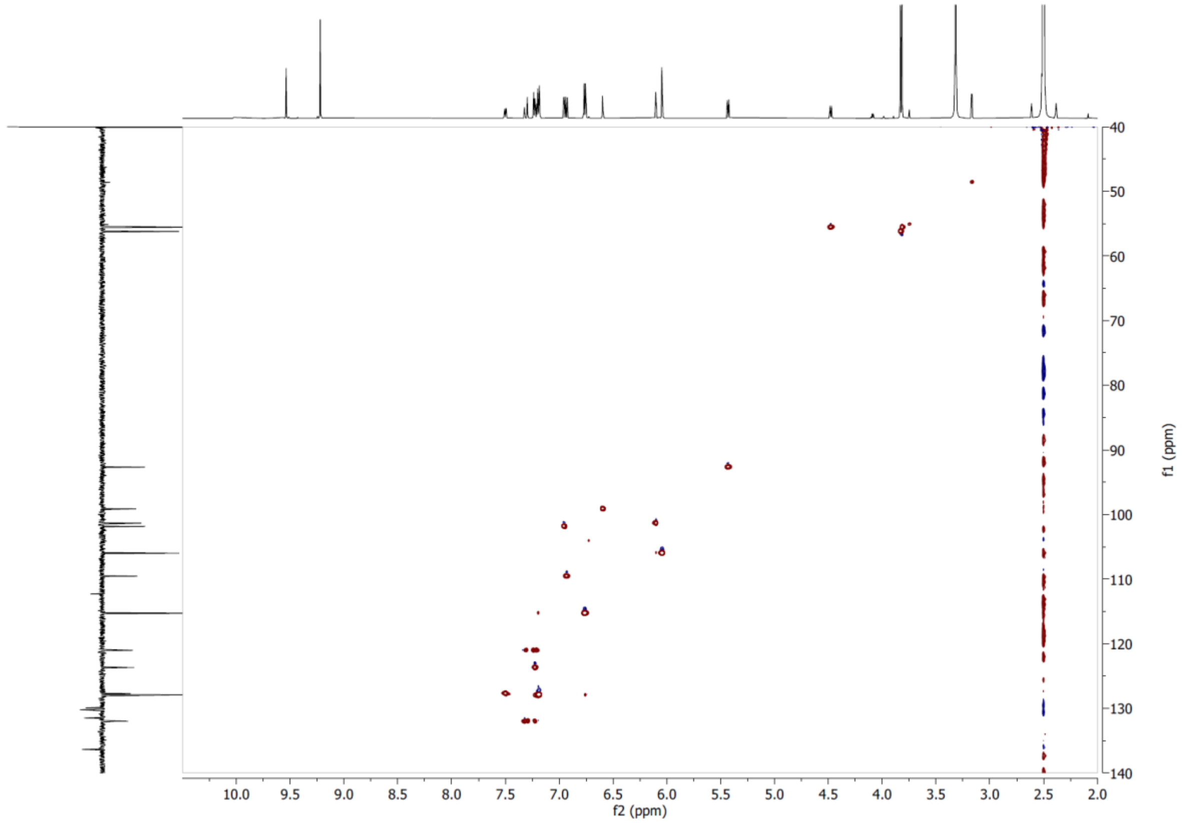

Edited HSQC NMR spectrum of compound **35** in DMSO-d_6

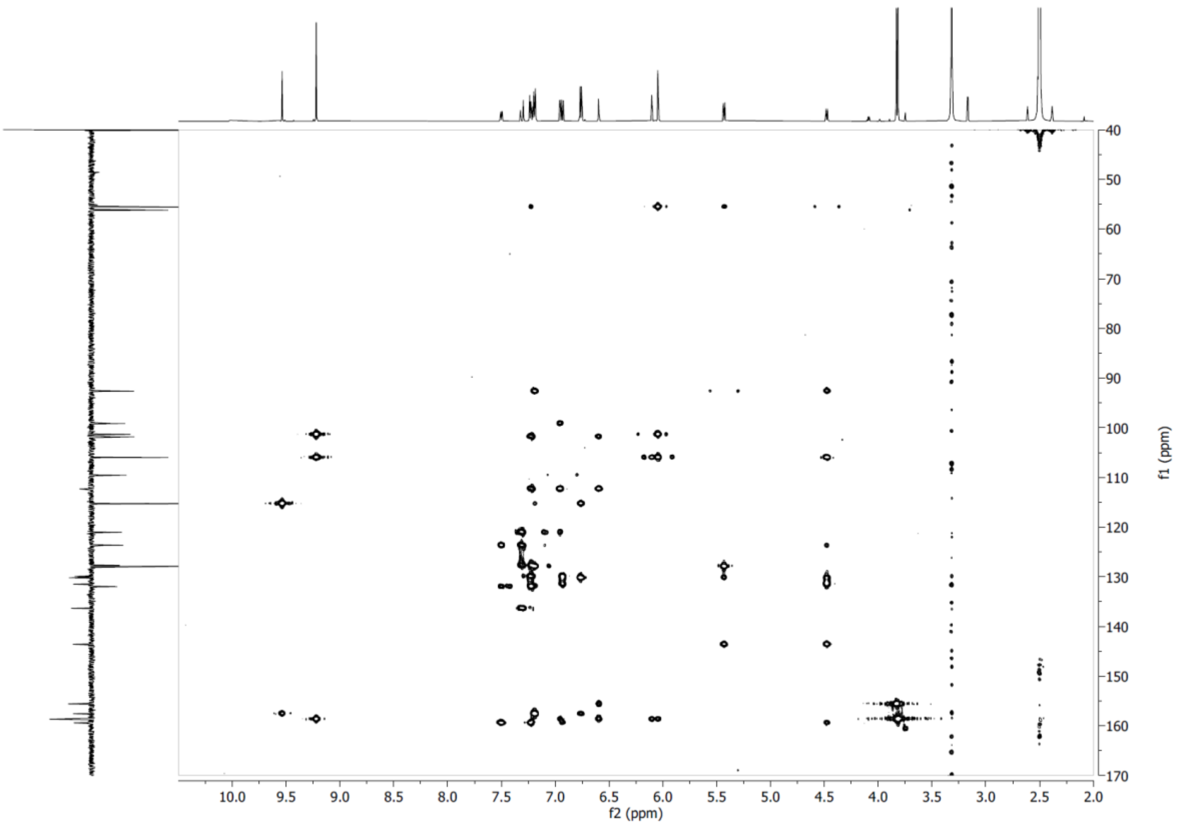

HMBC NMR spectrum of compound **35** in DMSO-d_6

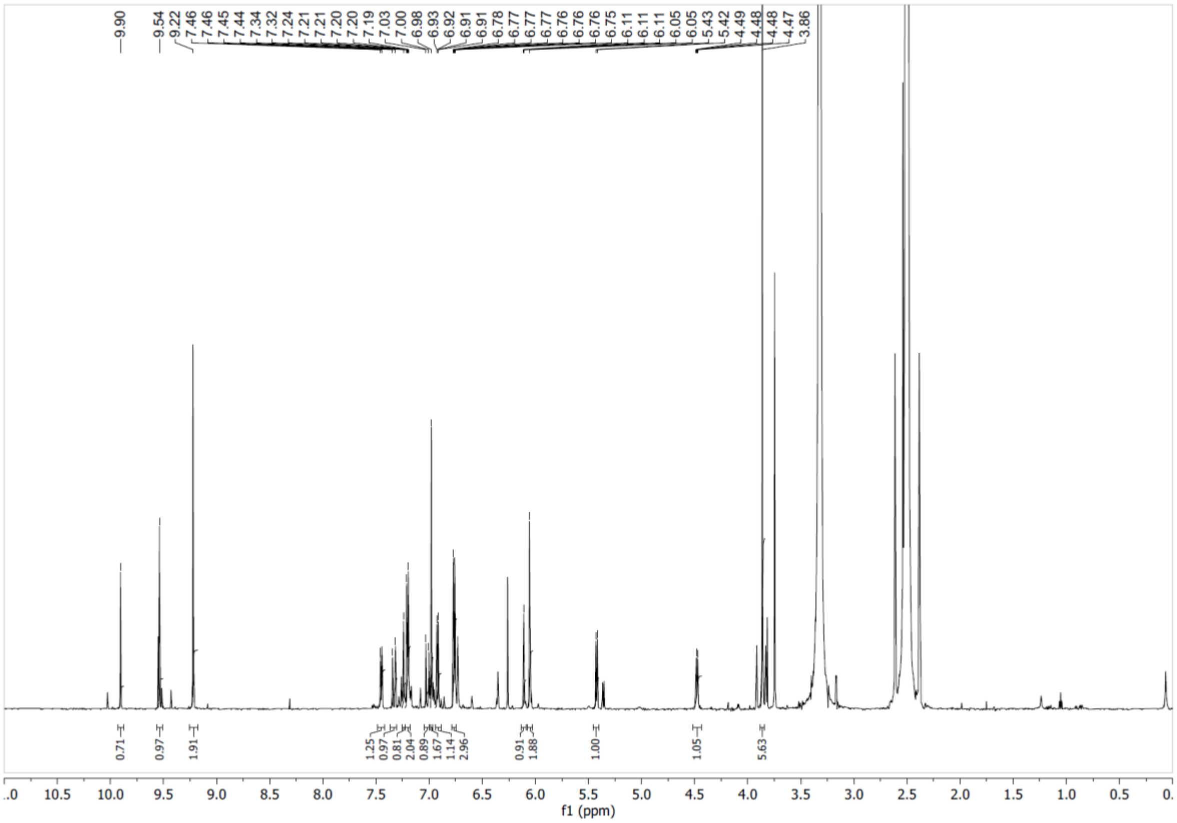

^{1}H NMR spectrum of compound **36** in DMSO-d_6 at 600 MHz

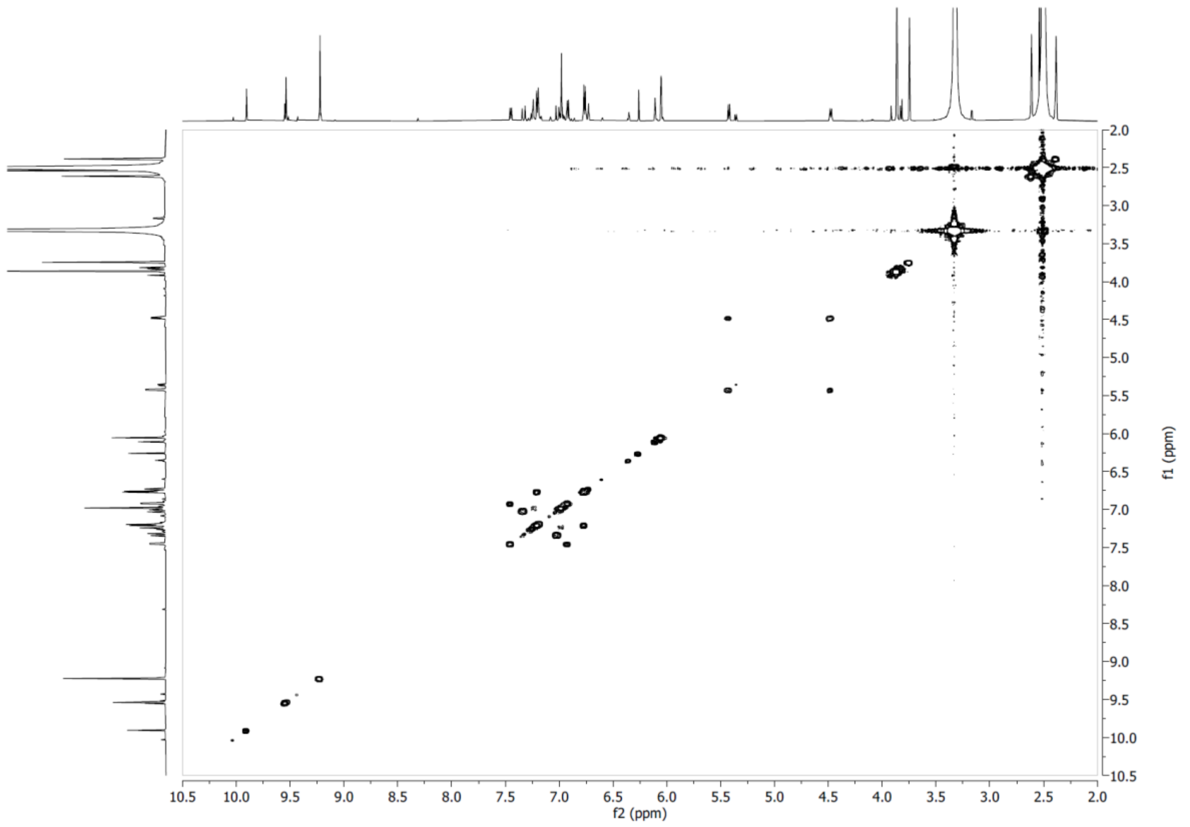

COSY NMR spectrum of compound **36** in DMSO-d_6

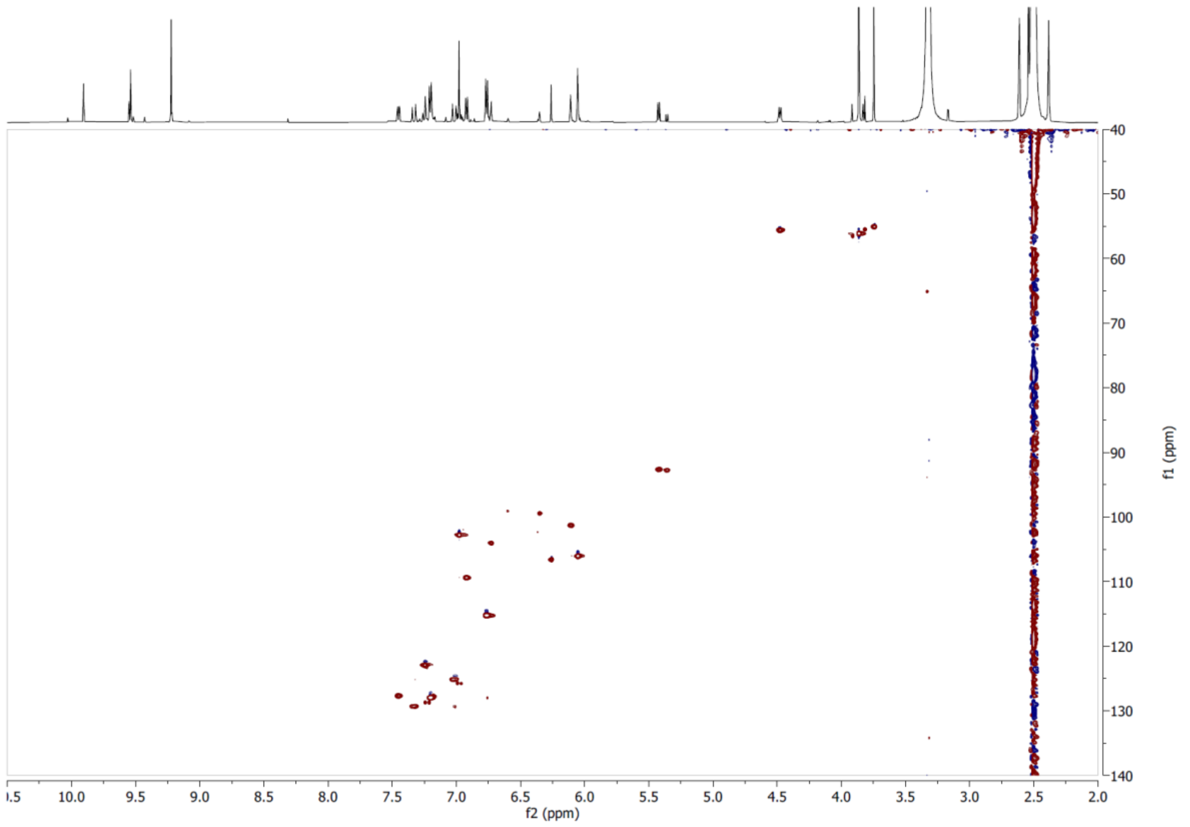

Edited HSQC NMR spectrum of compound **36** in DMSO-d_6

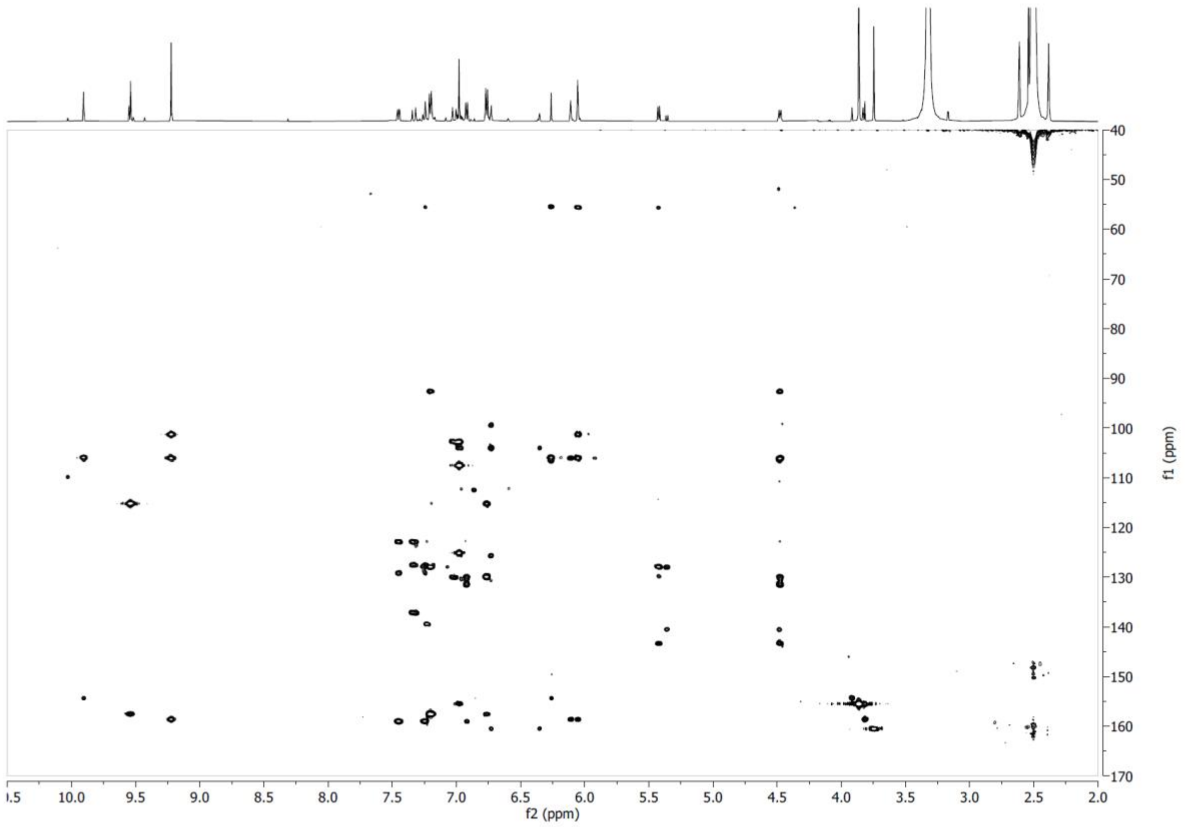

HMBC NMR spectrum of compound **36** in DMSO-d_6

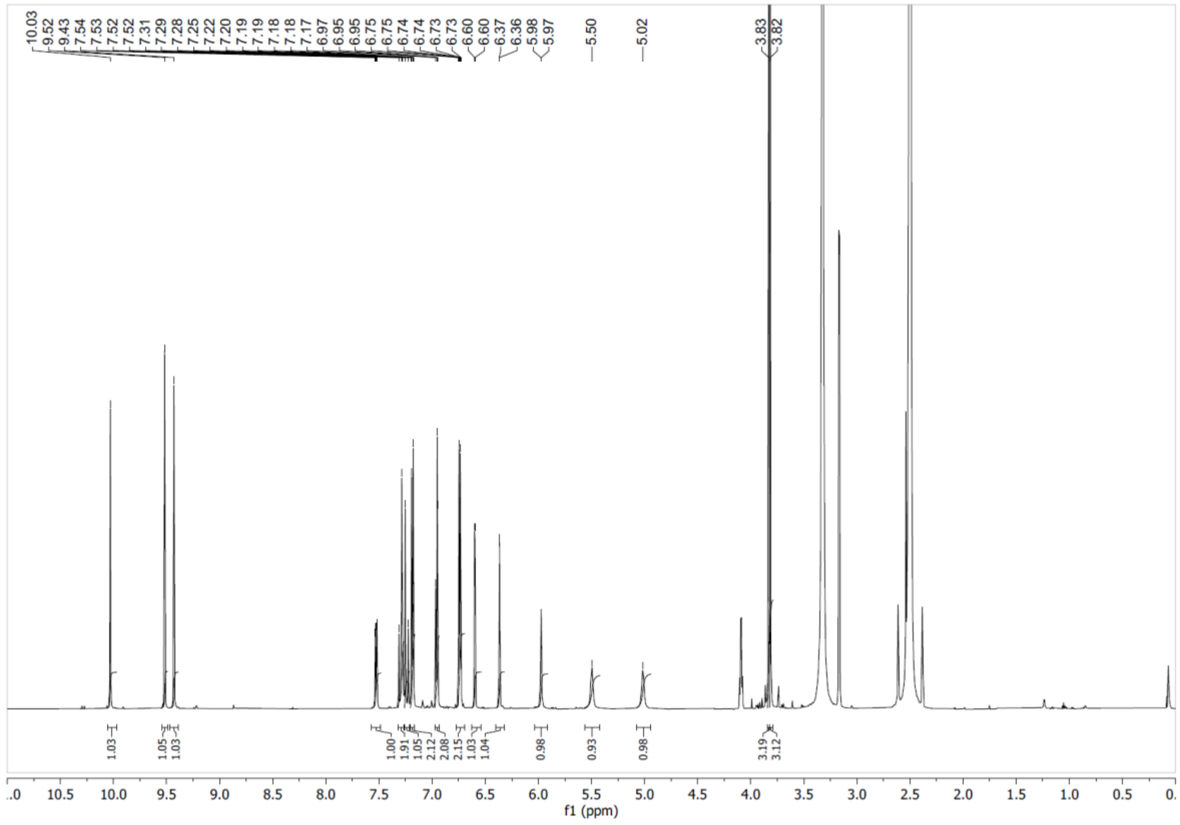

^{1}H NMR spectrum of compound **37** in DMSO-d_6 at 600 MHz

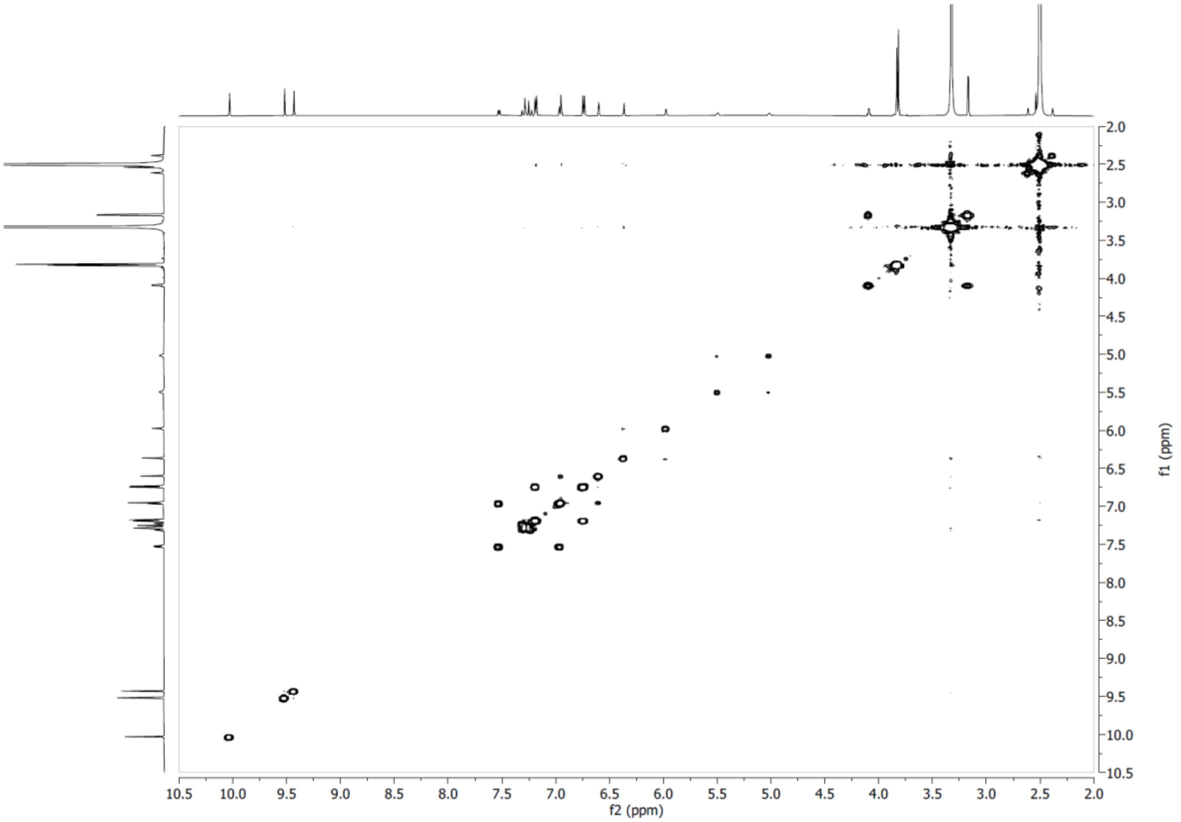

COSY NMR spectrum of compound **37** in DMSO-d_6

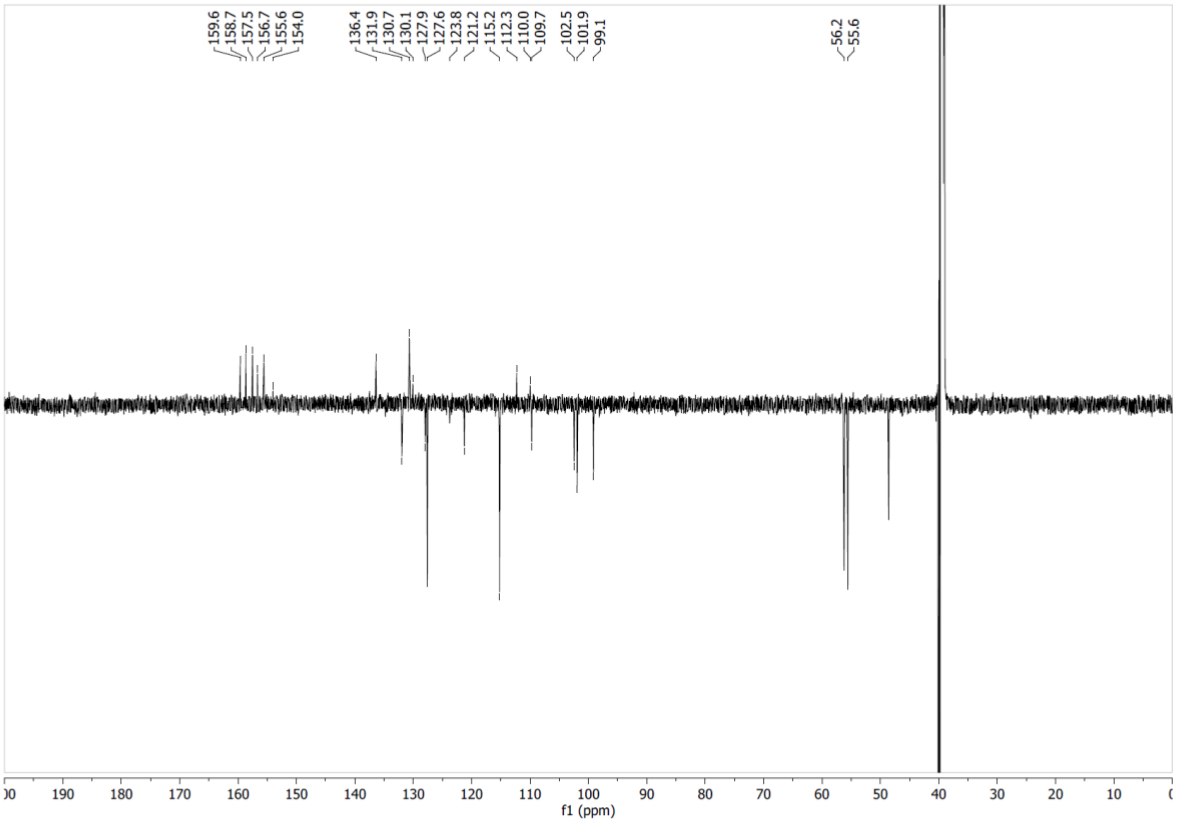

^{13}C-DEPTQ NMR spectrum of compound **37** in DMSO-d_6 at 151 MHz

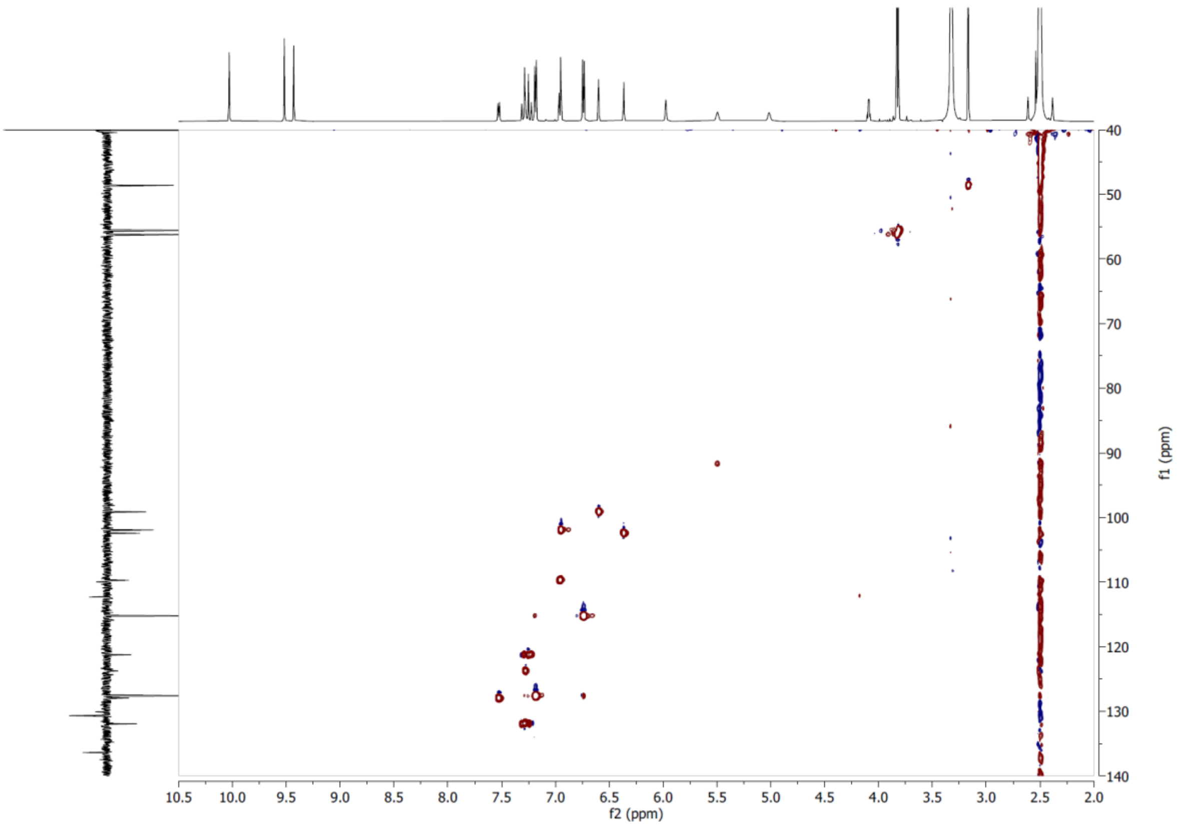

Edited HSQC NMR spectrum of compound **37** in DMSO-d_6

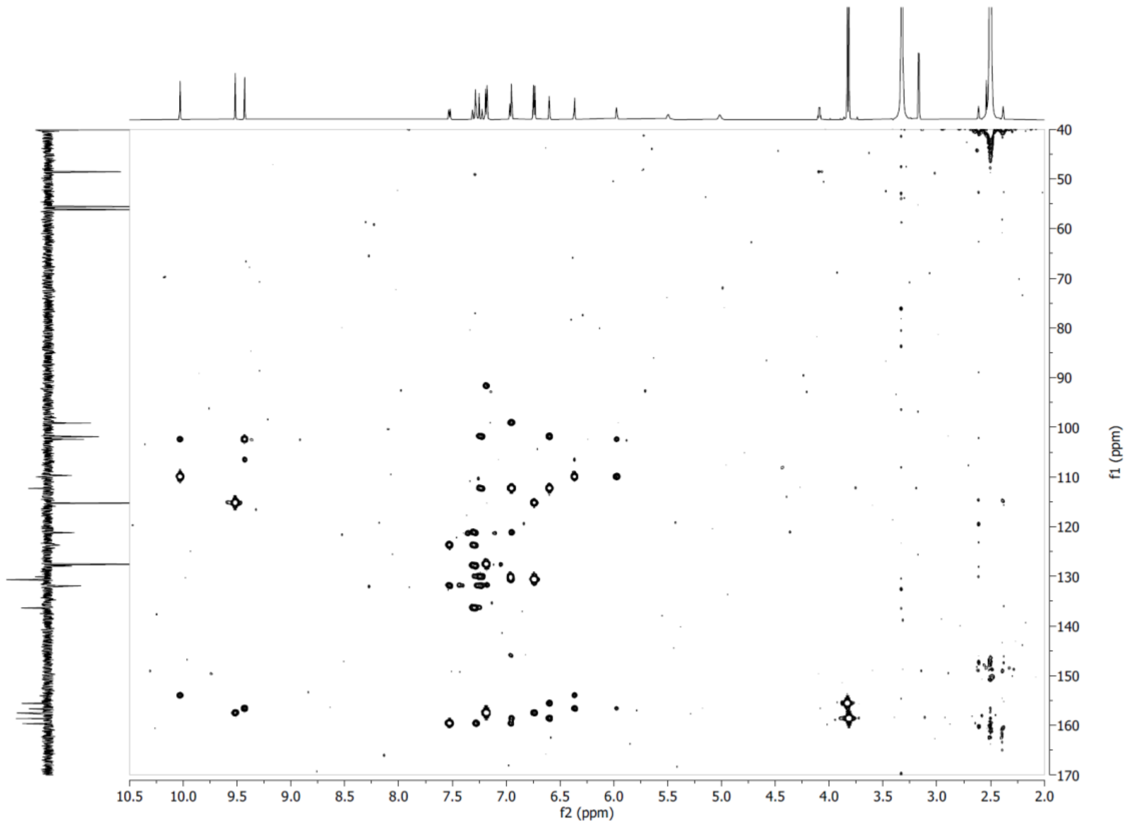

HMBC NMR spectrum of compound **37** in DMSO-d_6

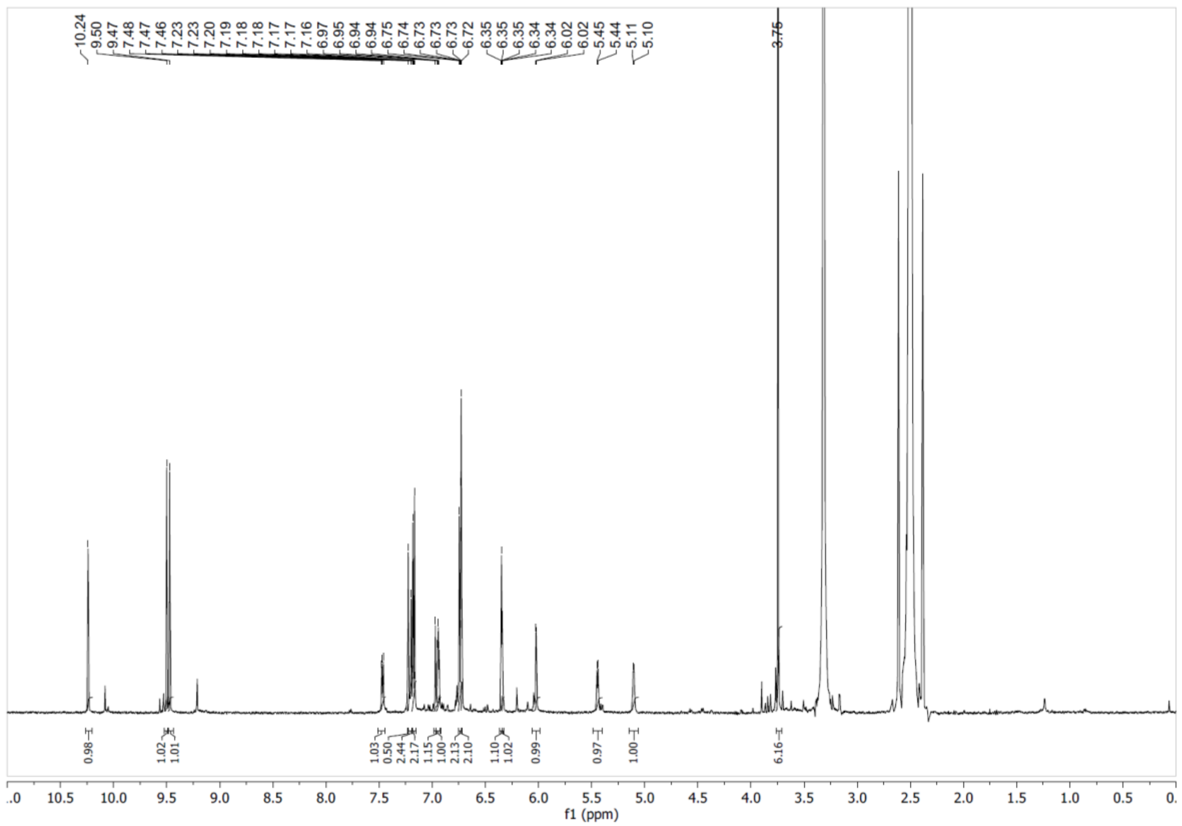

^{1}H NMR spectrum of compound **38** in DMSO-d_6 at 600 MHz

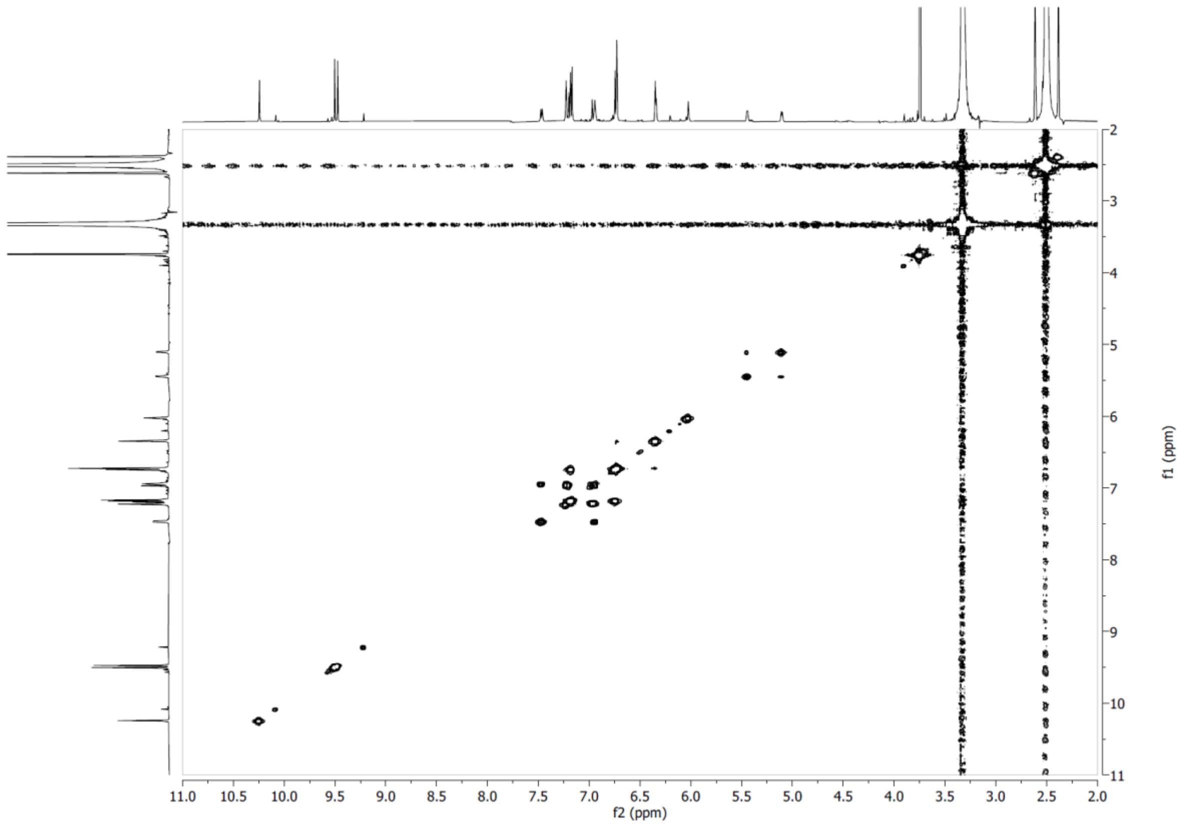

COSY NMR spectrum of compound **38** in DMSO-d_6

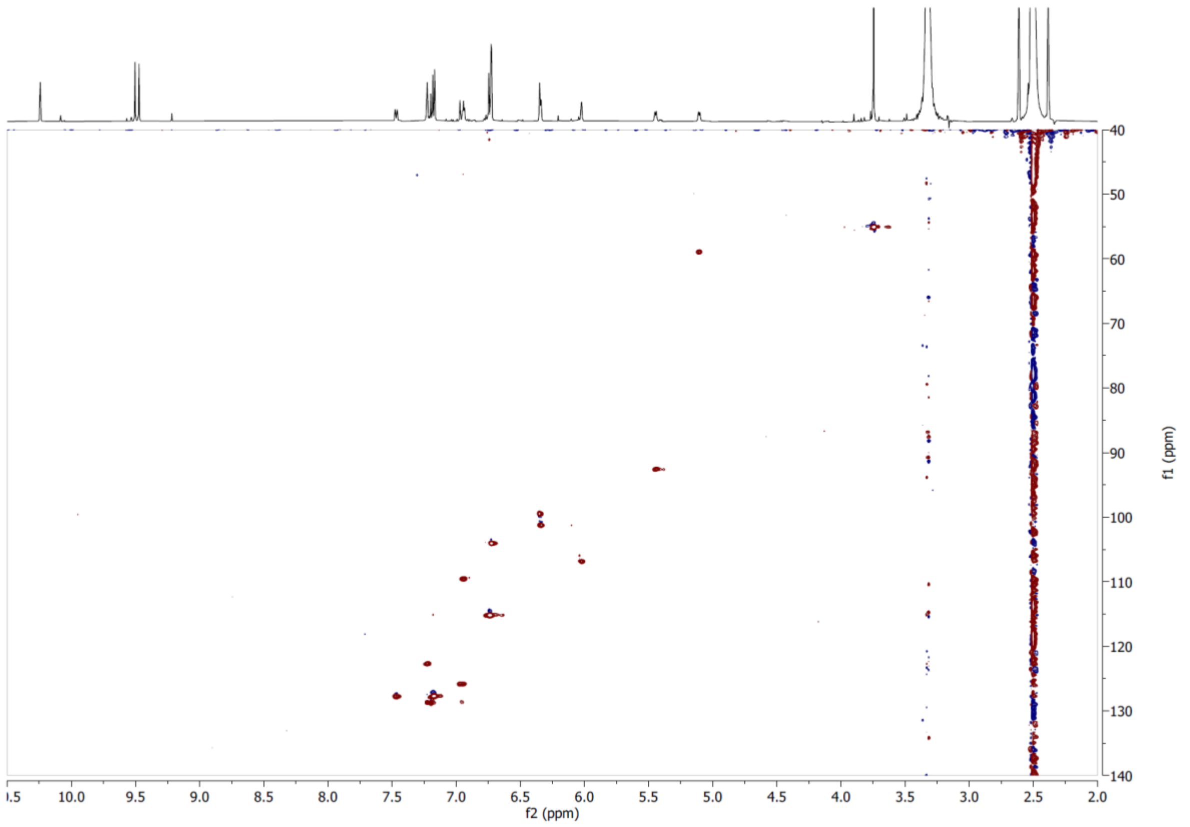

Edited HSQC NMR spectrum of compound **38** in DMSO-d_6

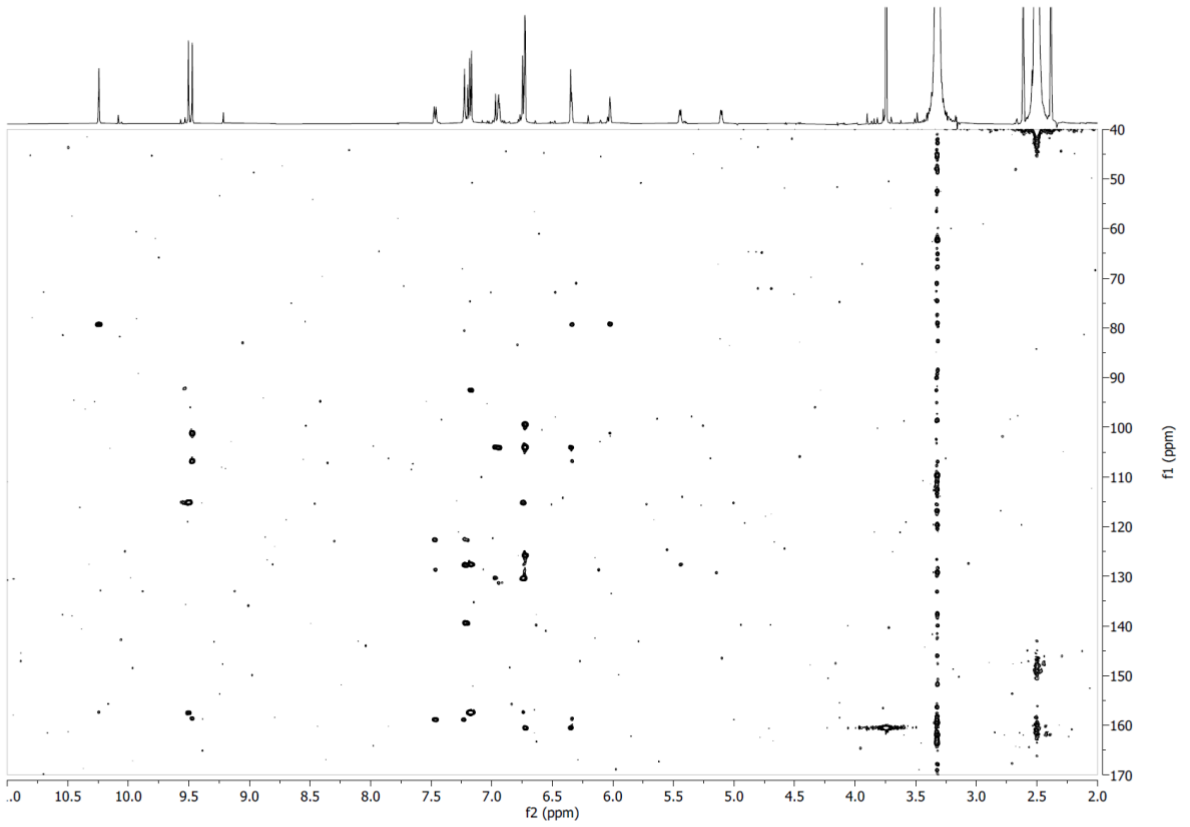

HMBC NMR spectrum of compound **38** in DMSO-d_6

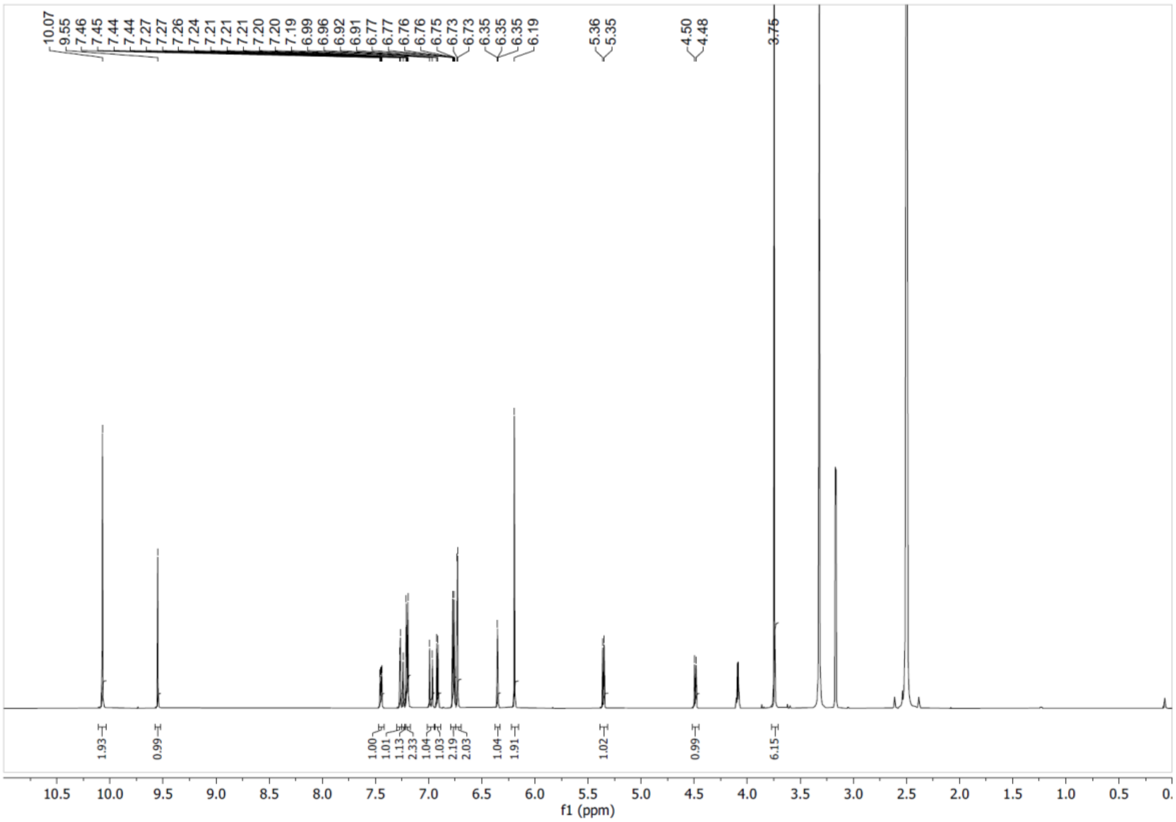

^{1}H NMR spectrum of compound **39** in DMSO-d_6 at 600 MHz

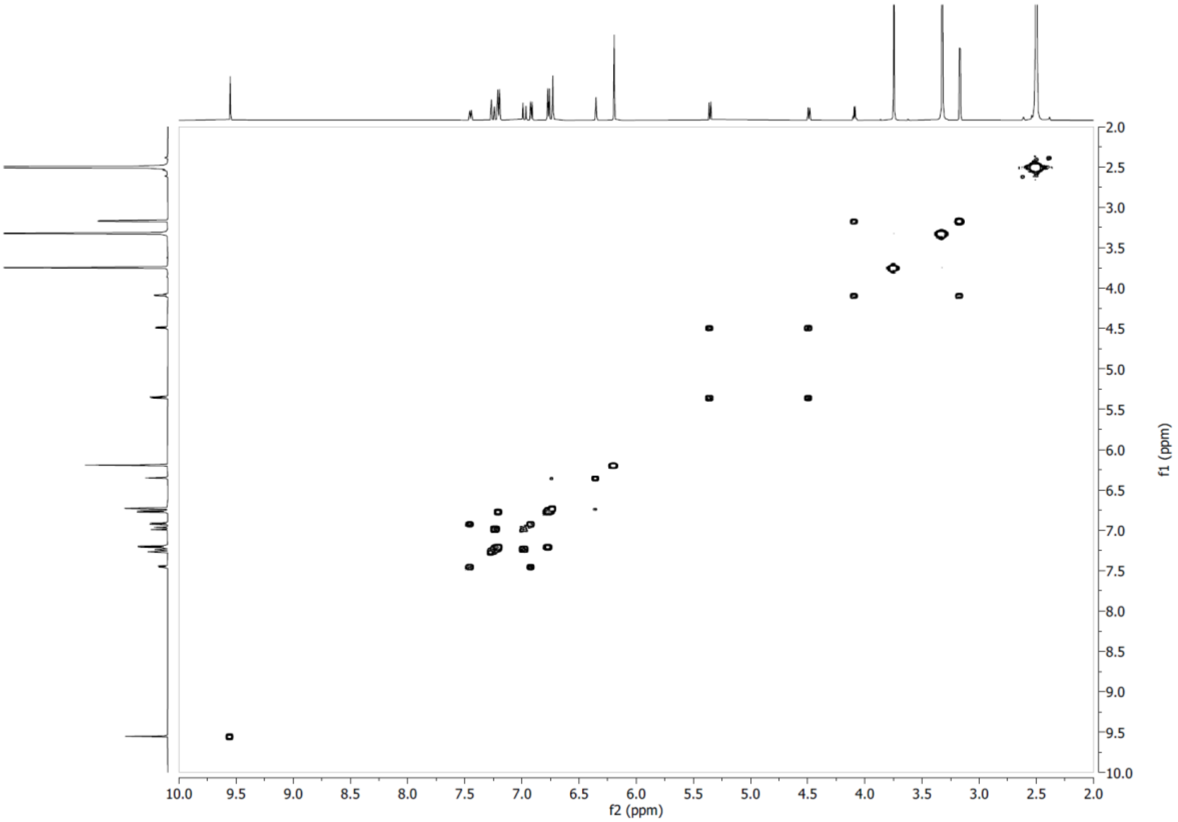

COSY NMR spectrum of compound **39** in DMSO-d_6

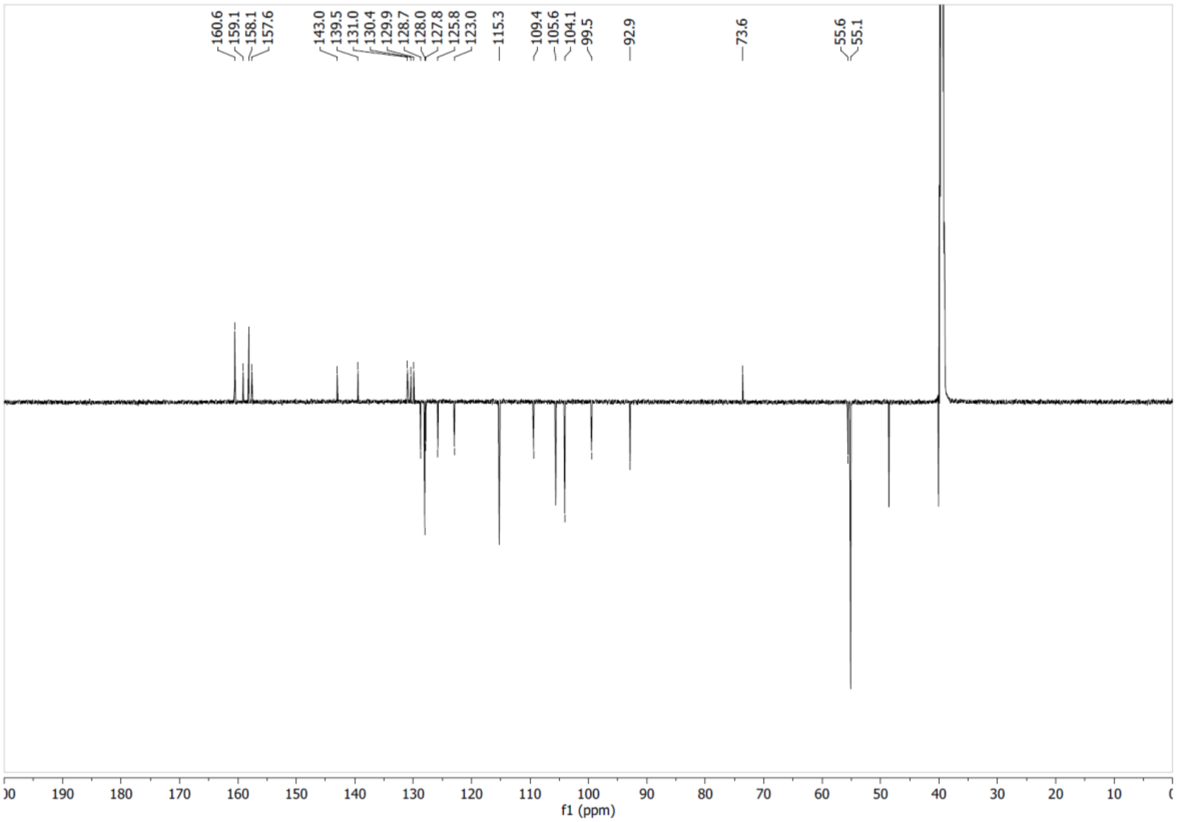

^{13}C-DEPTQ NMR spectrum of compound **39** in DMSO-d_6 at 151 MHz

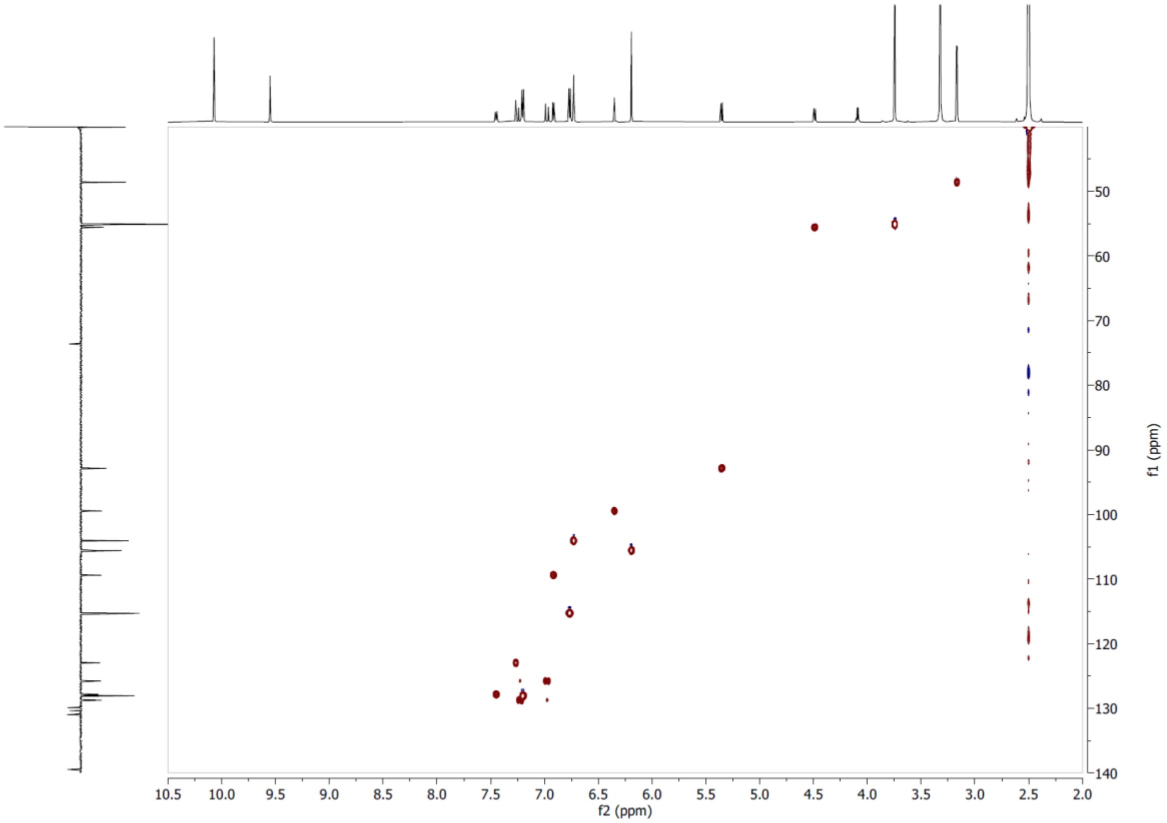

Edited HSQC NMR spectrum of compound **39** in DMSO-d_6

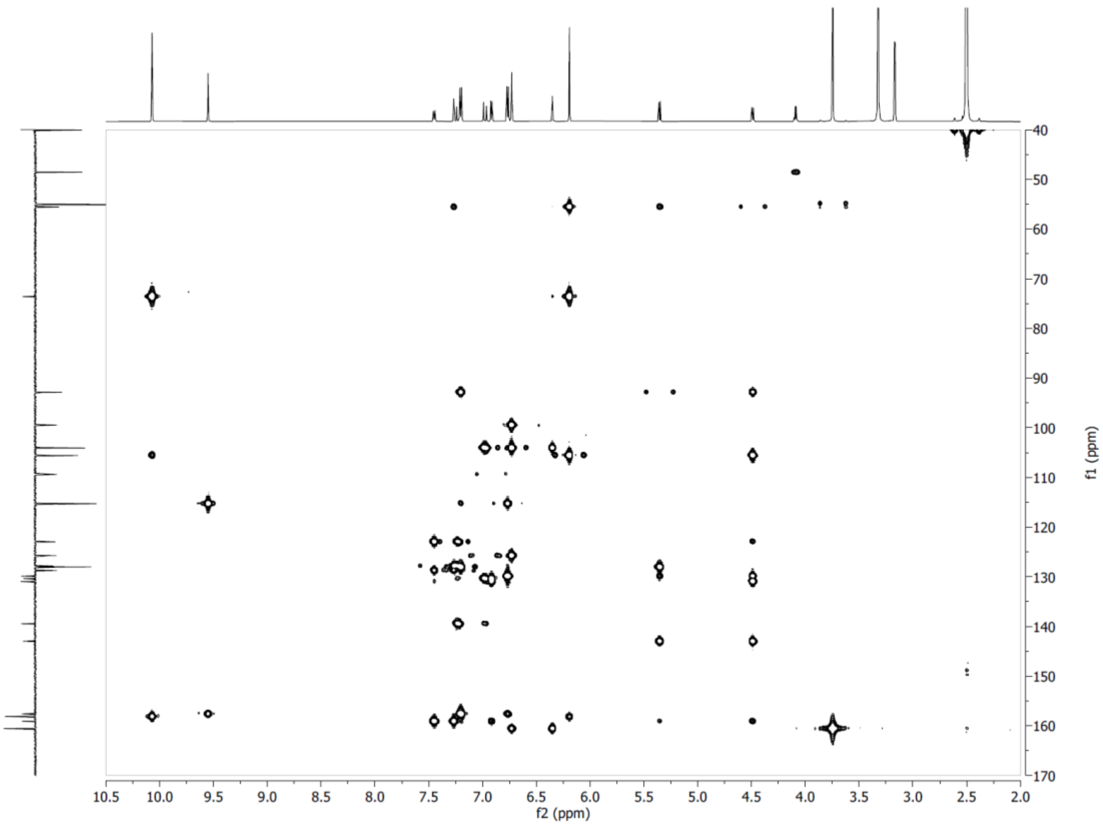

HMBC NMR spectrum of compound **39** in DMSO-d_6

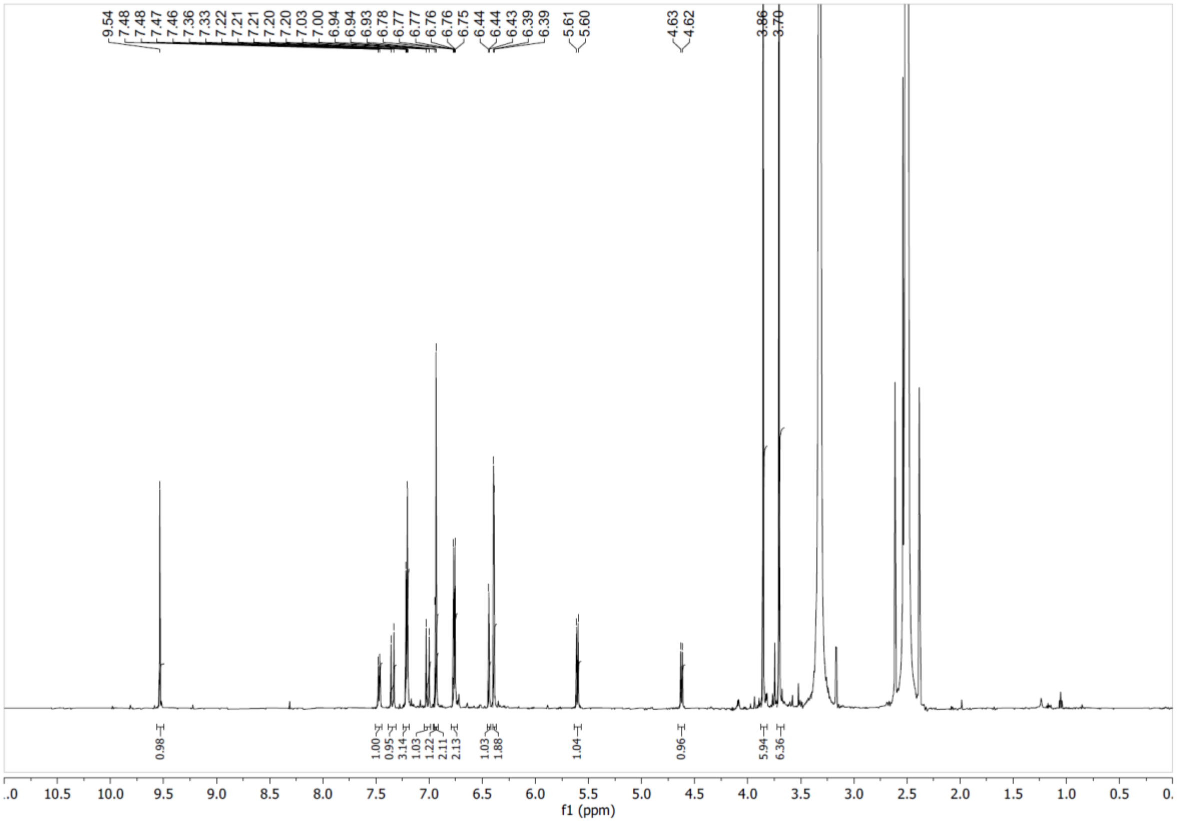

^{1}H NMR spectrum of compound **40** in DMSO-d_6 at 600 MHz

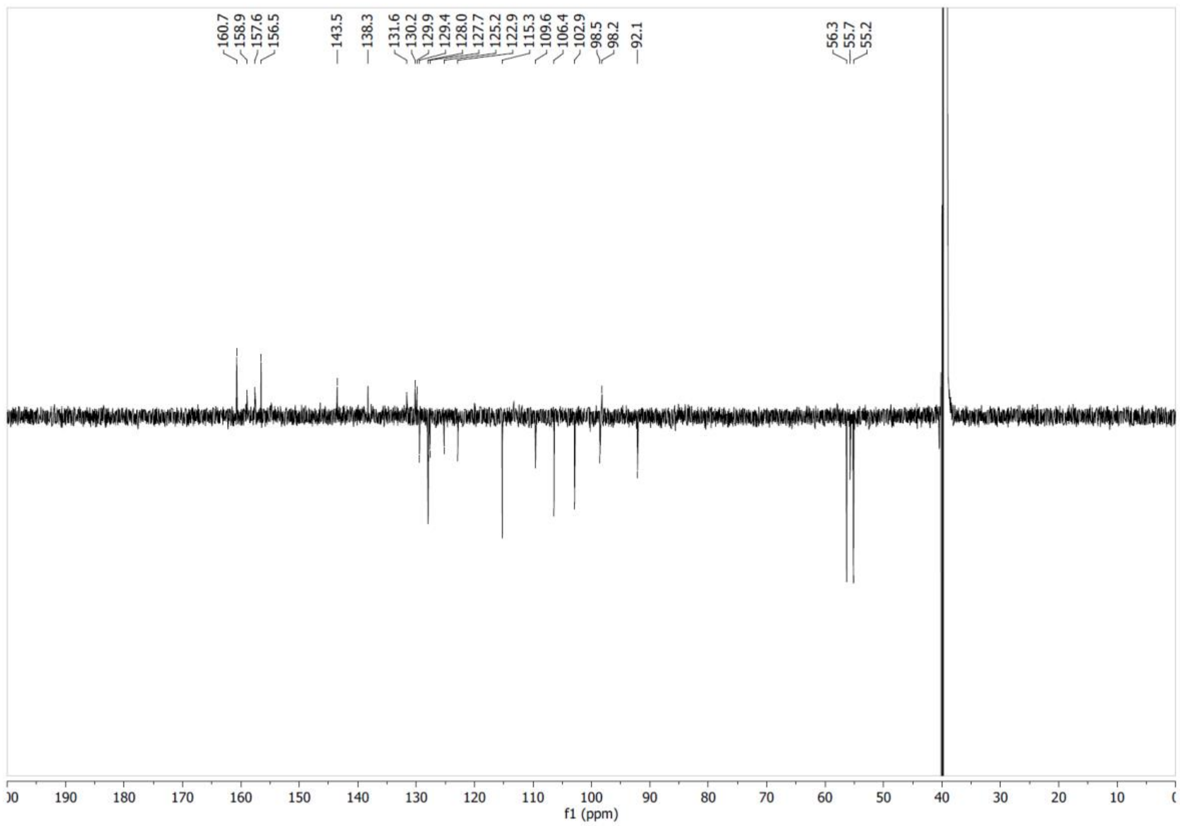

^{13}C-DEPTQ NMR spectrum of compound **40** in DMSO-d_6 at 151 MHz

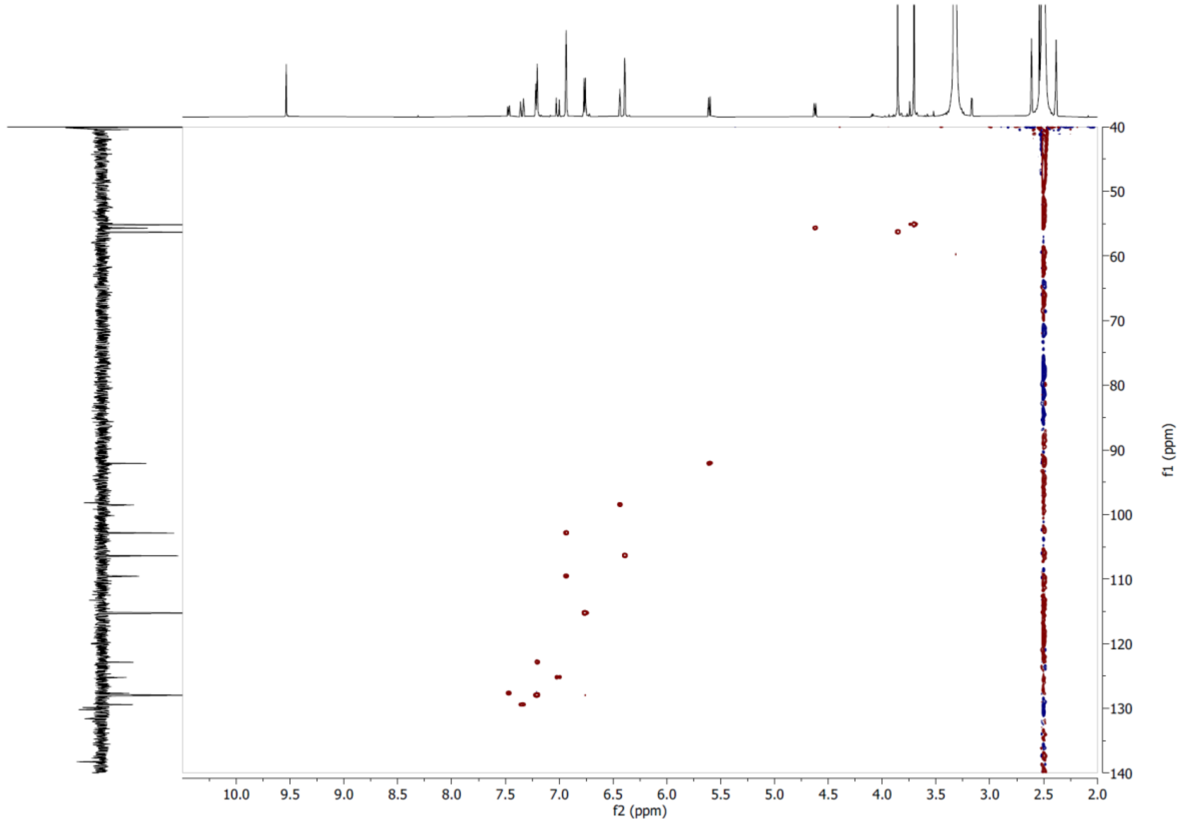

Edited HSQC NMR spectrum of compound **40** in DMSO-d_6

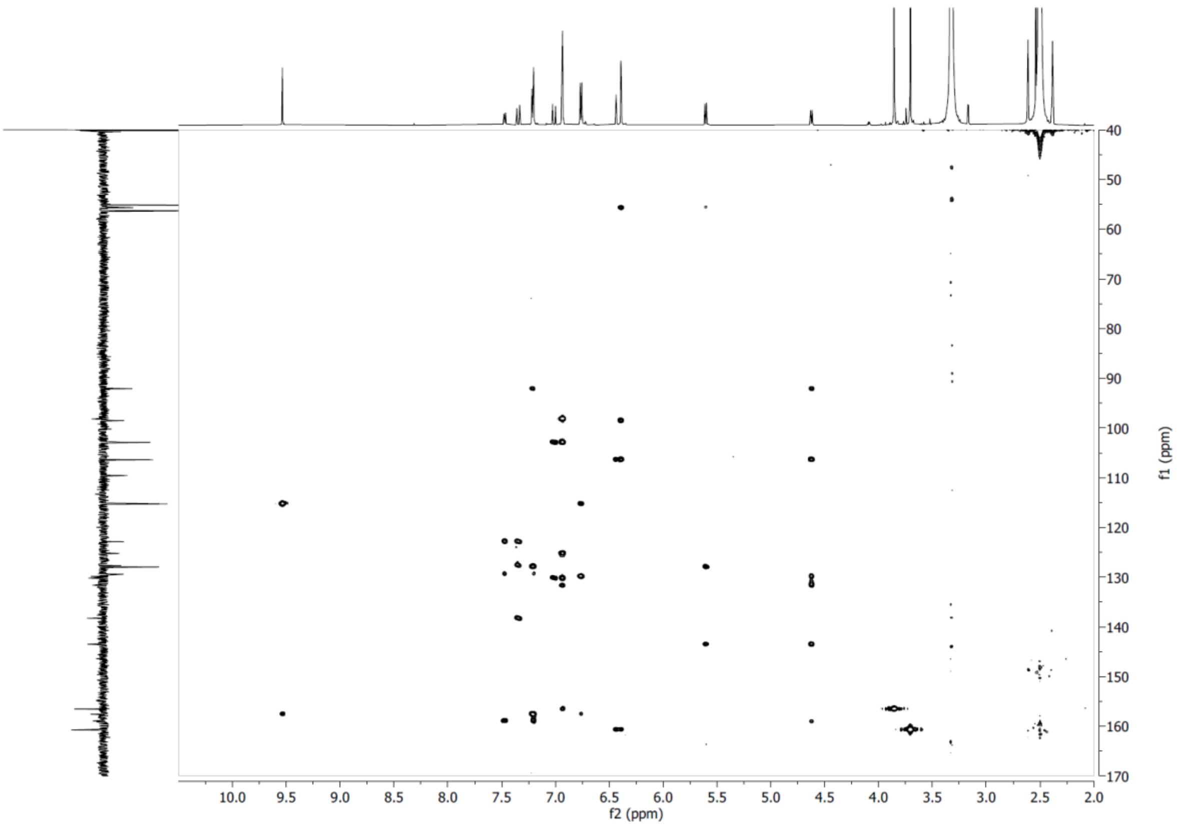

HMBC NMR spectrum of compound **40** in DMSO-d_6

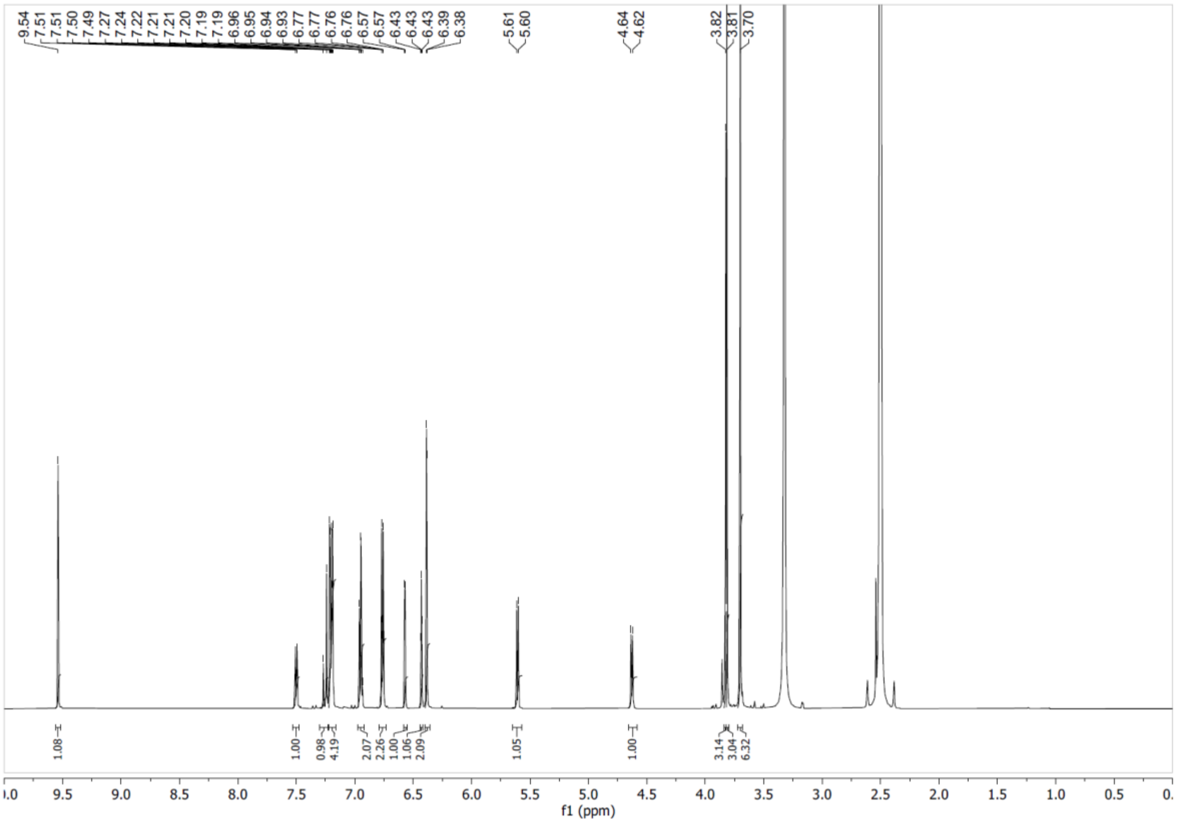

^{1}H NMR spectrum of compound **41** in DMSO-d_6 at 600 MHz

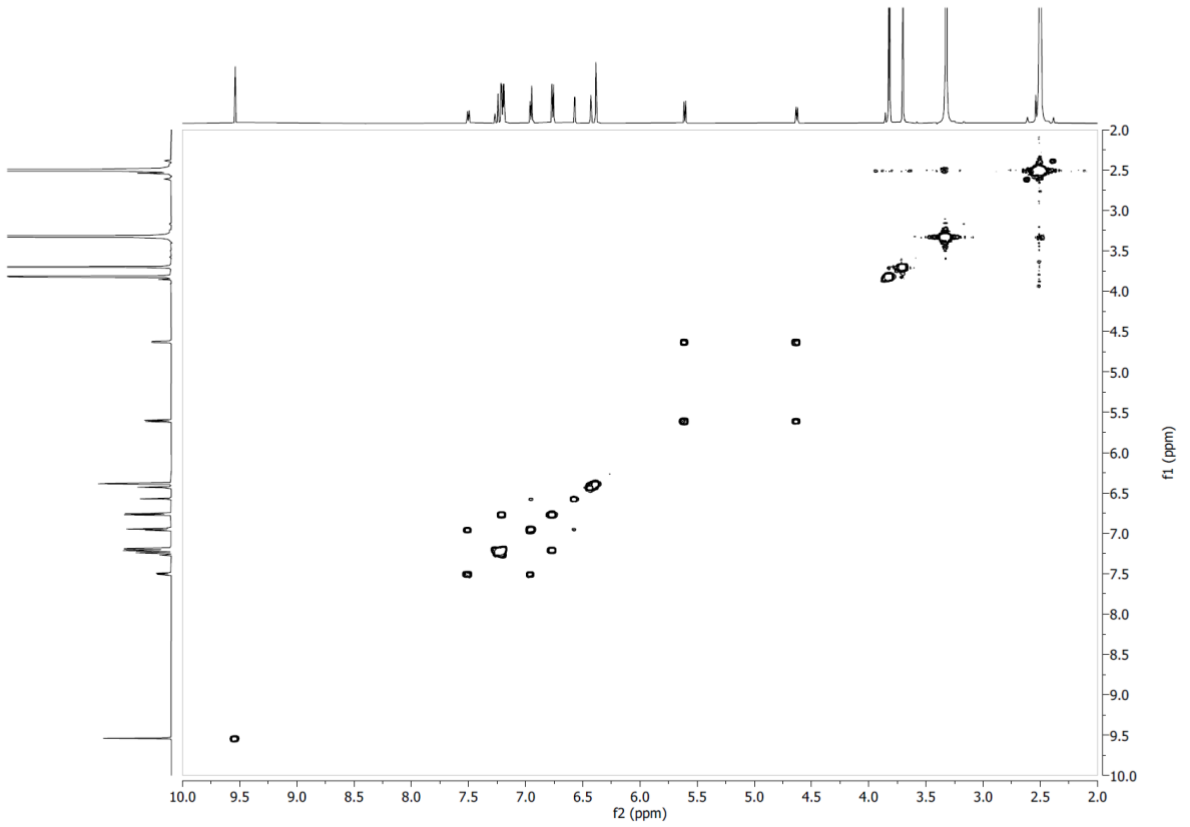

COSY NMR spectrum of compound **41** in DMSO-d_6

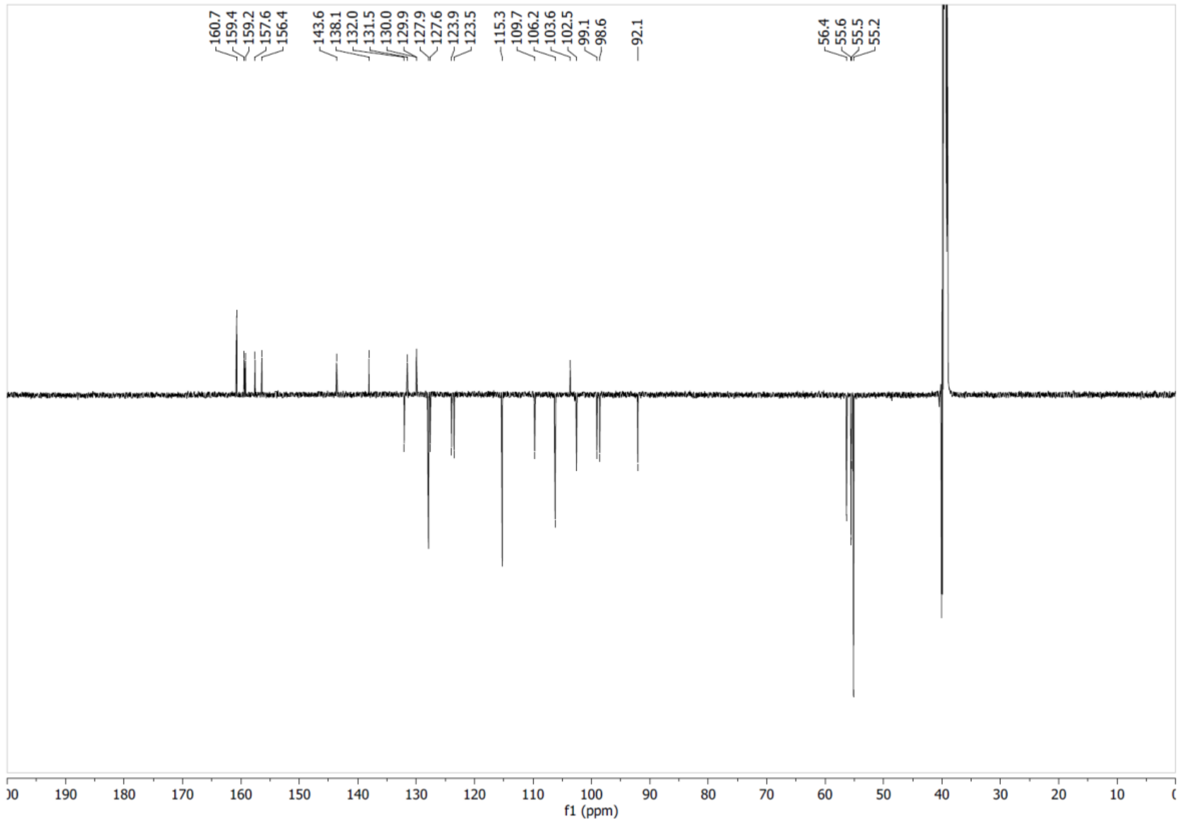

^{13}C-DEPTQ NMR spectrum of compound **41** in DMSO-d_6 at 151 MHz

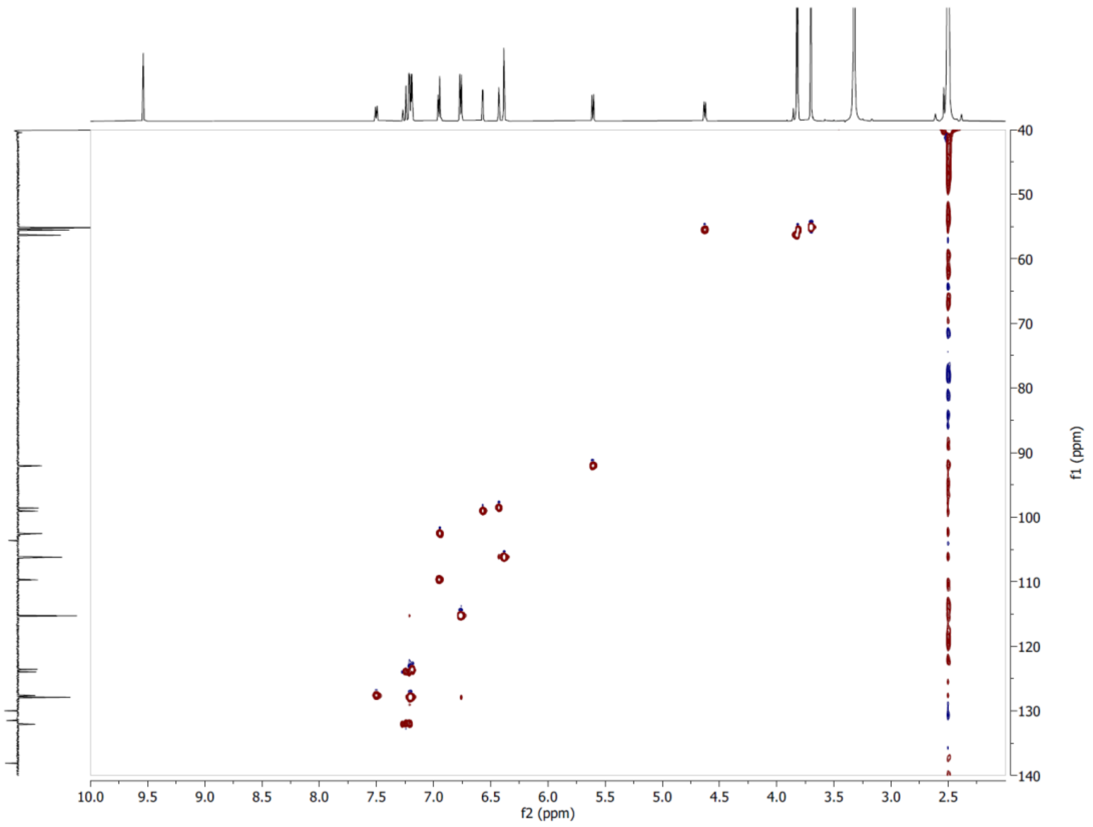

Edited HSQC NMR spectrum of compound **41** in DMSO-d_6

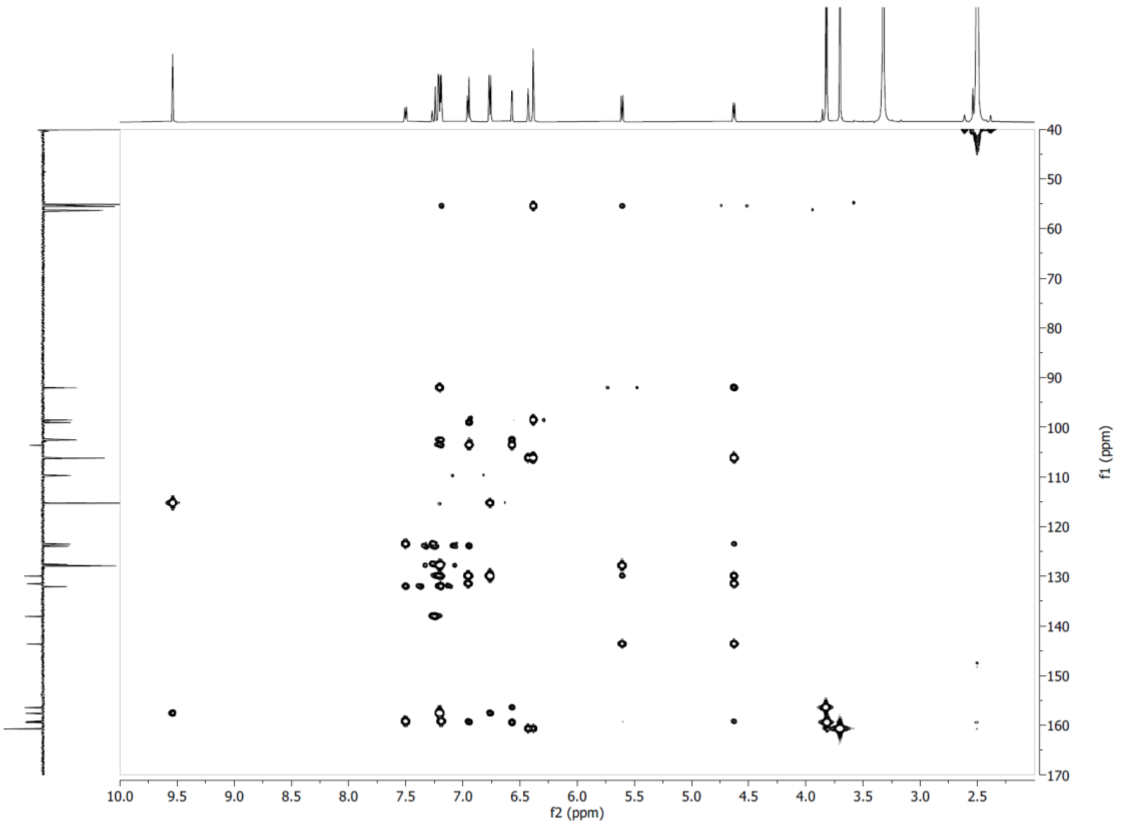

HMBC NMR spectrum of compound **41** in DMSO-d_6

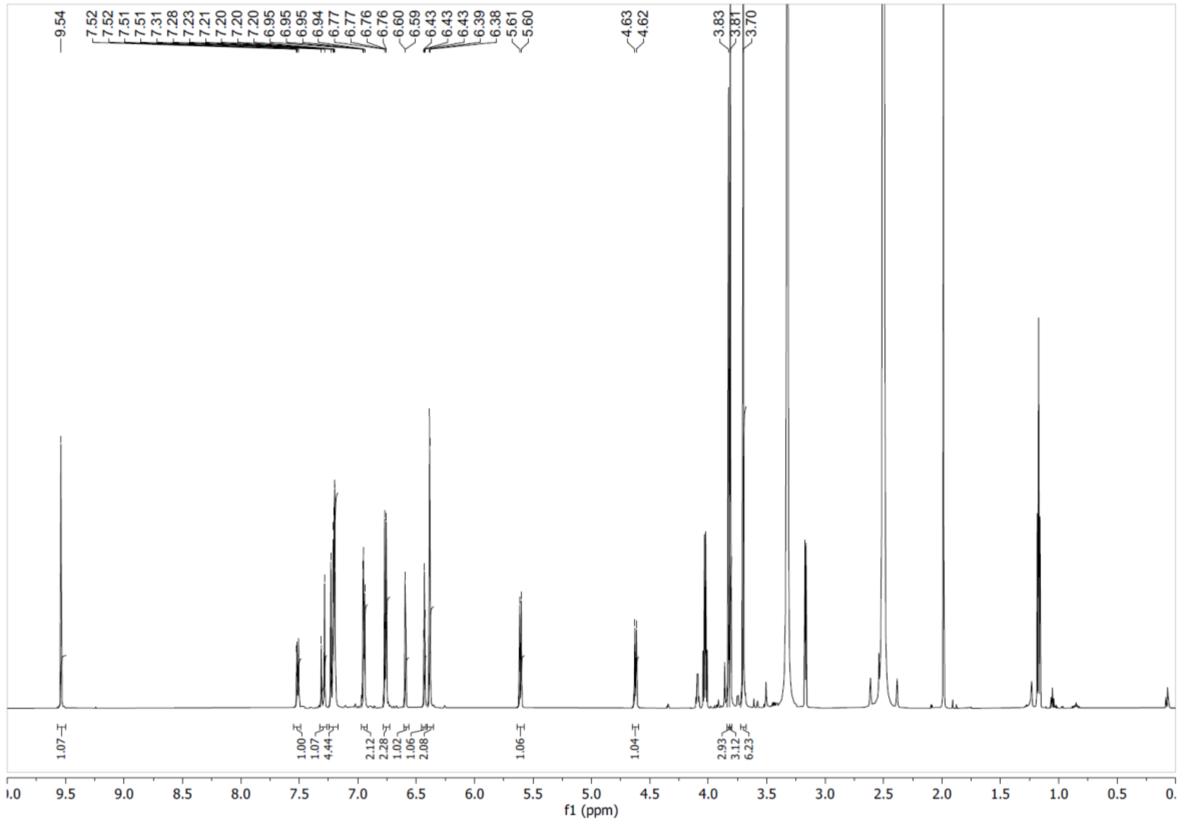

^{1}H NMR spectrum of compound **42** in DMSO-d_6 at 600 MHz

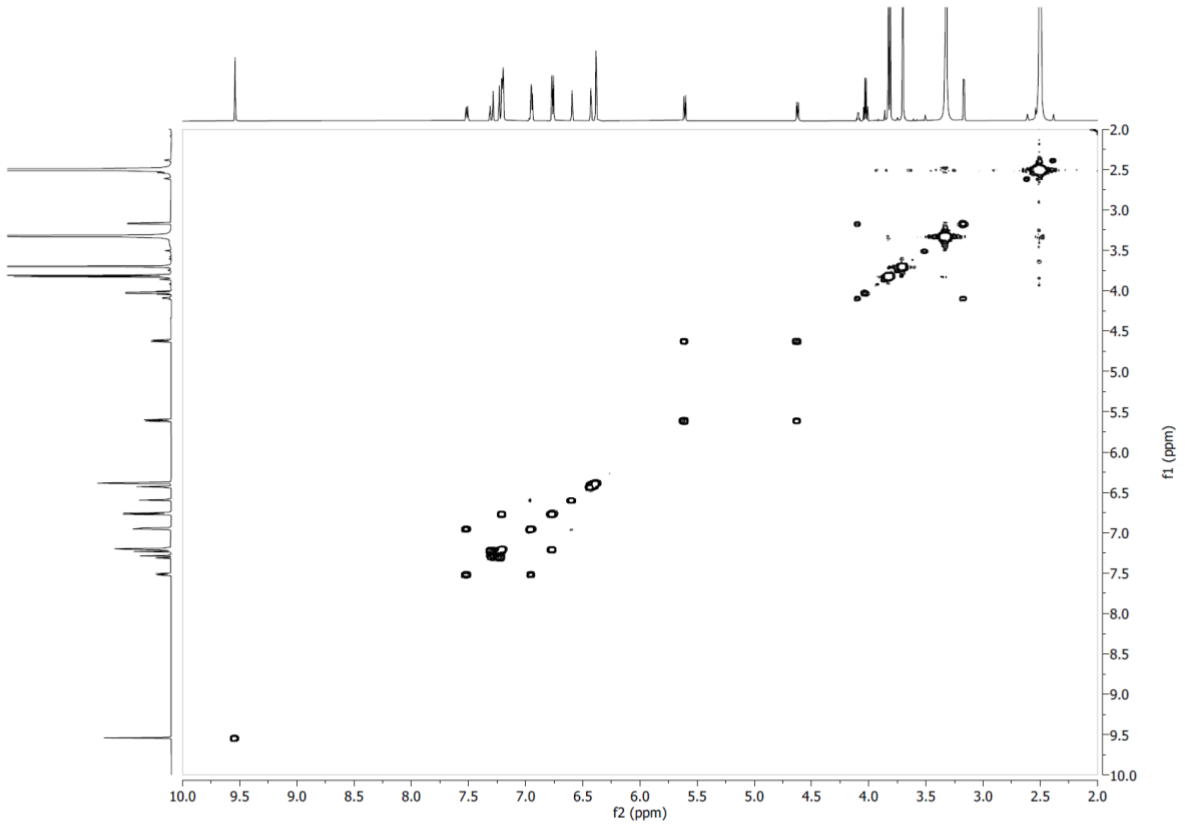

COSY NMR spectrum of compound **42** in DMSO-d_6

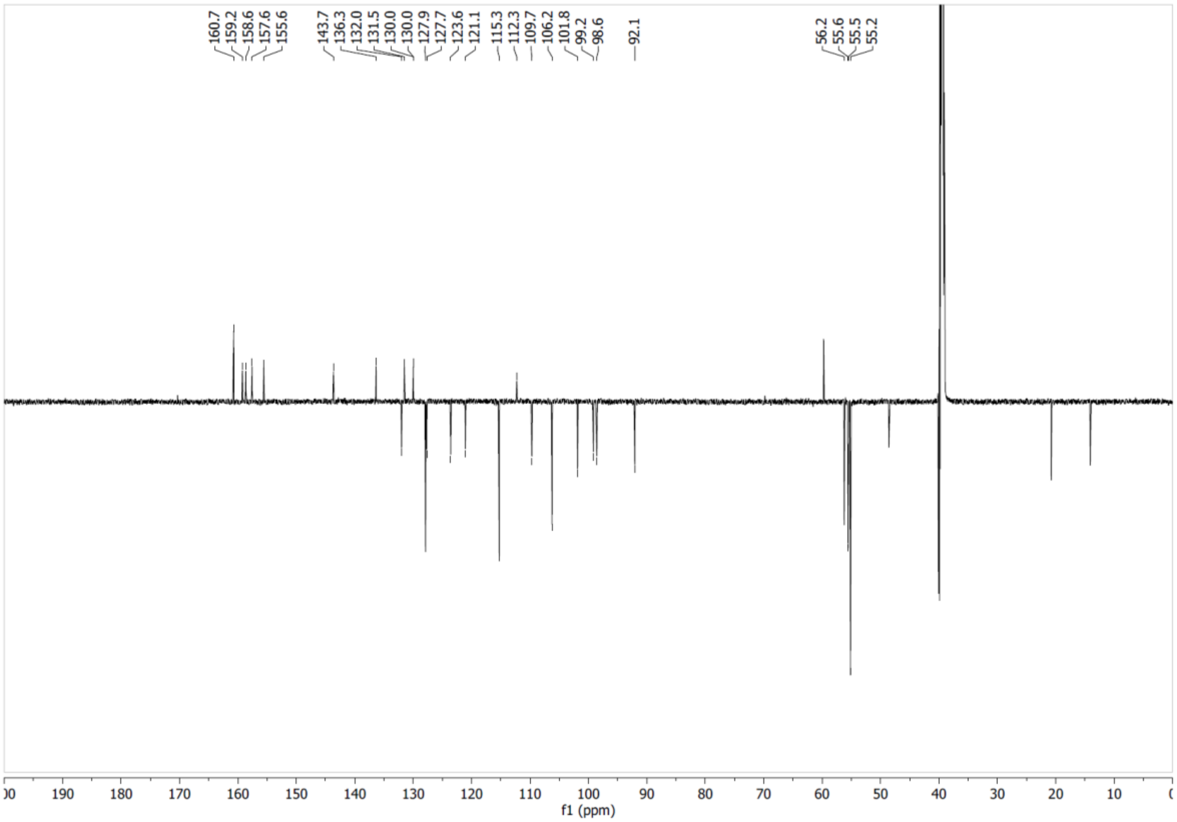

^{13}C-DEPTQ NMR spectrum of compound **42** in DMSO-d_6 at 151 MHz

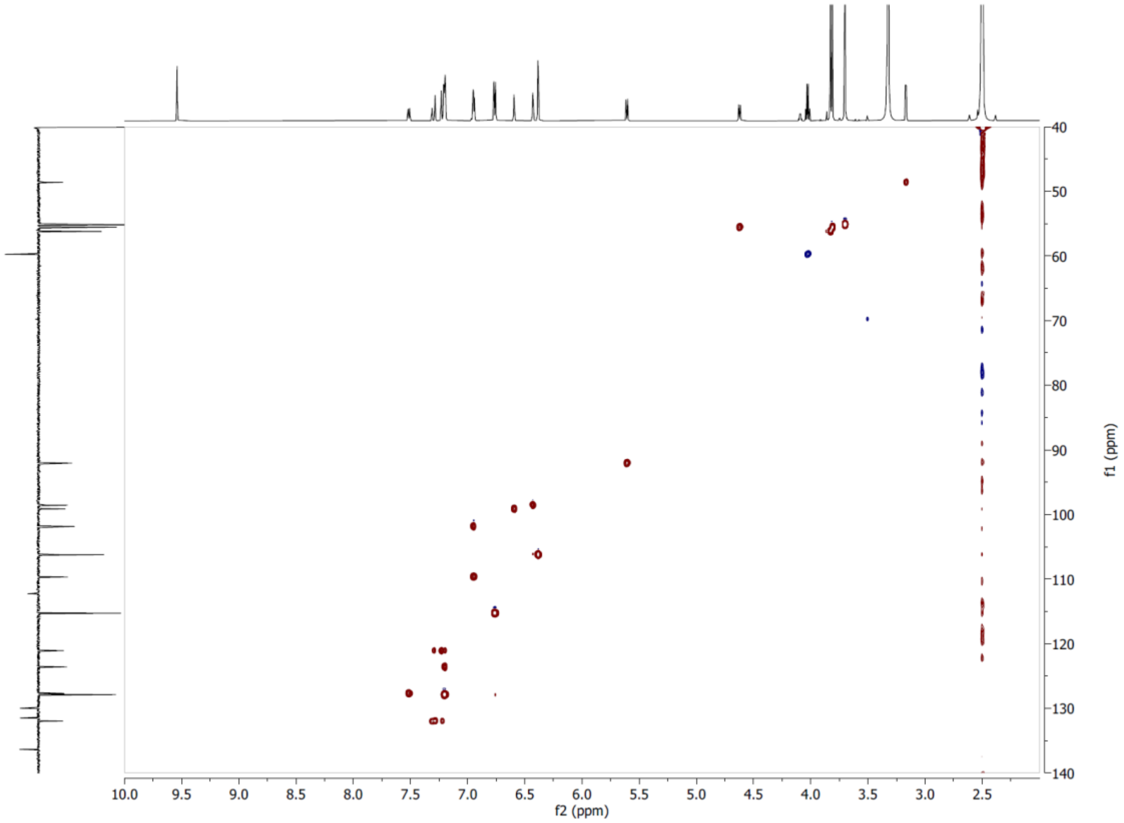

Edited HSQC NMR spectrum of compound **42** in DMSO-d_6

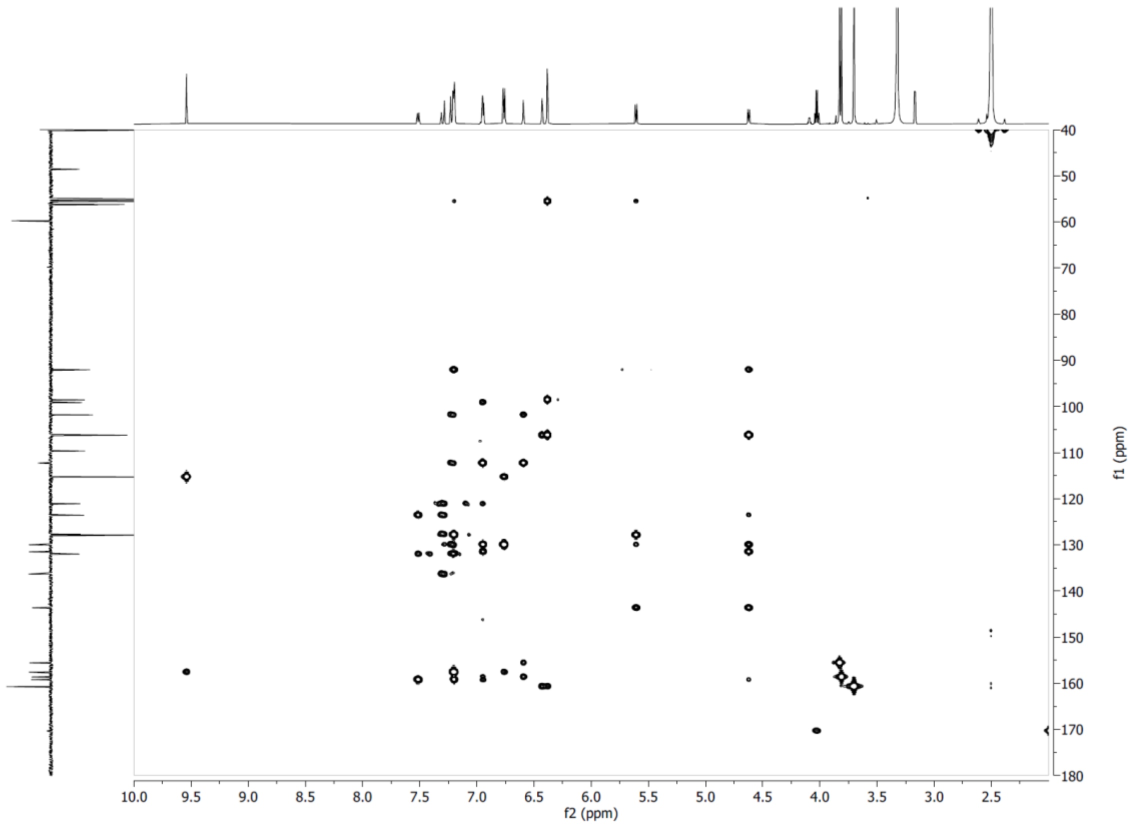

HMBC NMR spectrum of compound **42** in DMSO-d_6

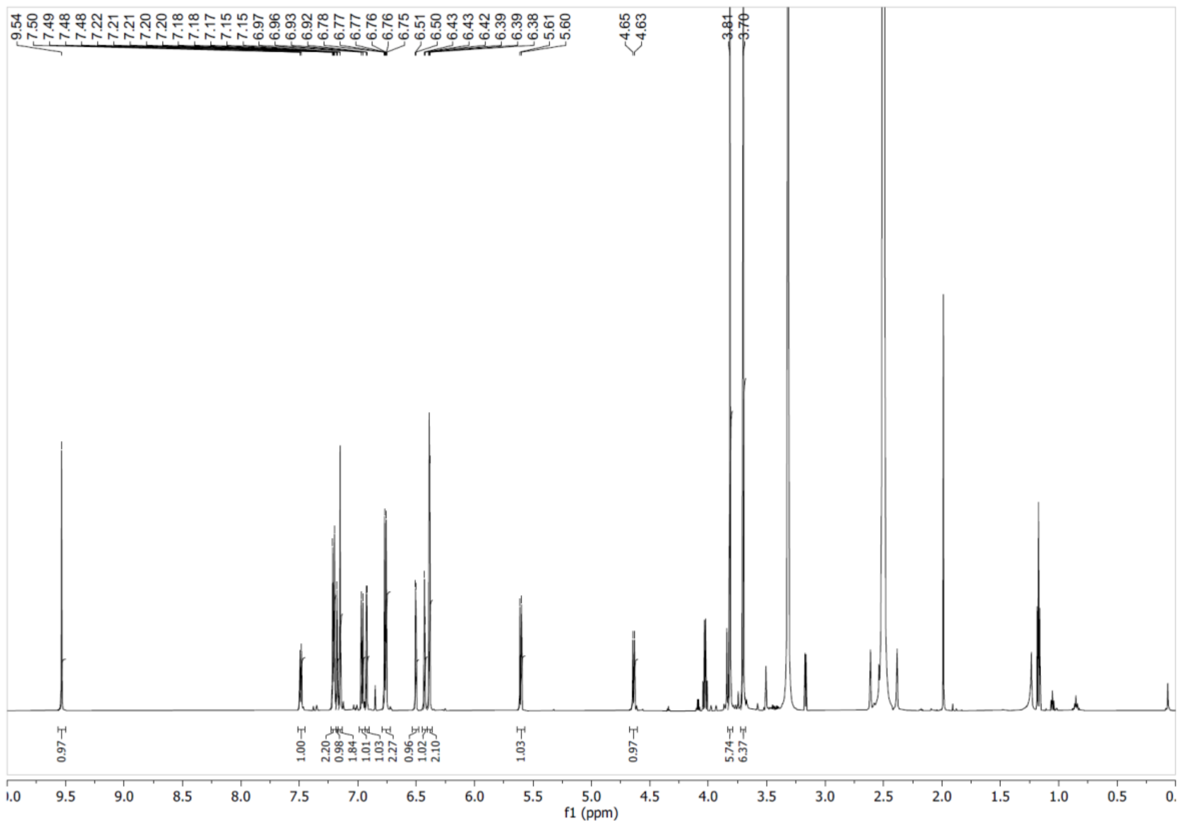

^{1}H NMR spectrum of compound **43** in DMSO-d_6 at 600 MHz

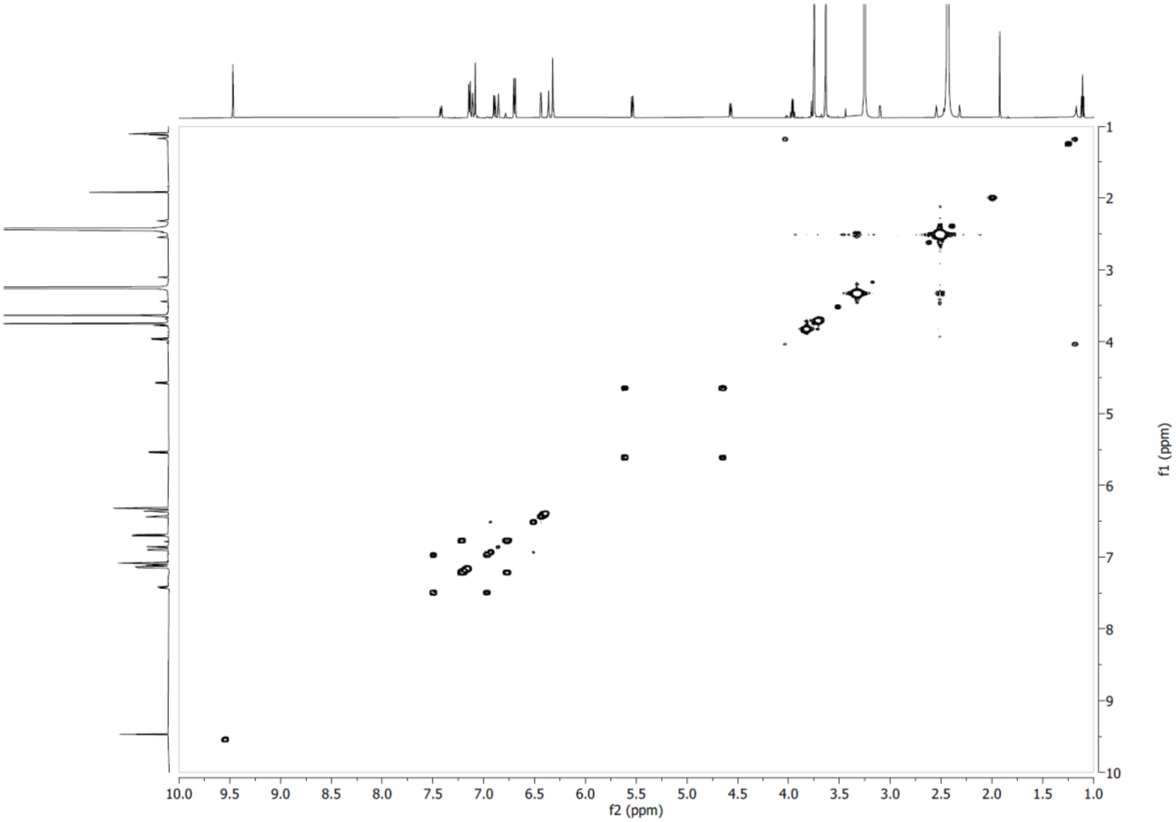

COSY NMR spectrum of compound **43** in DMSO-d_6

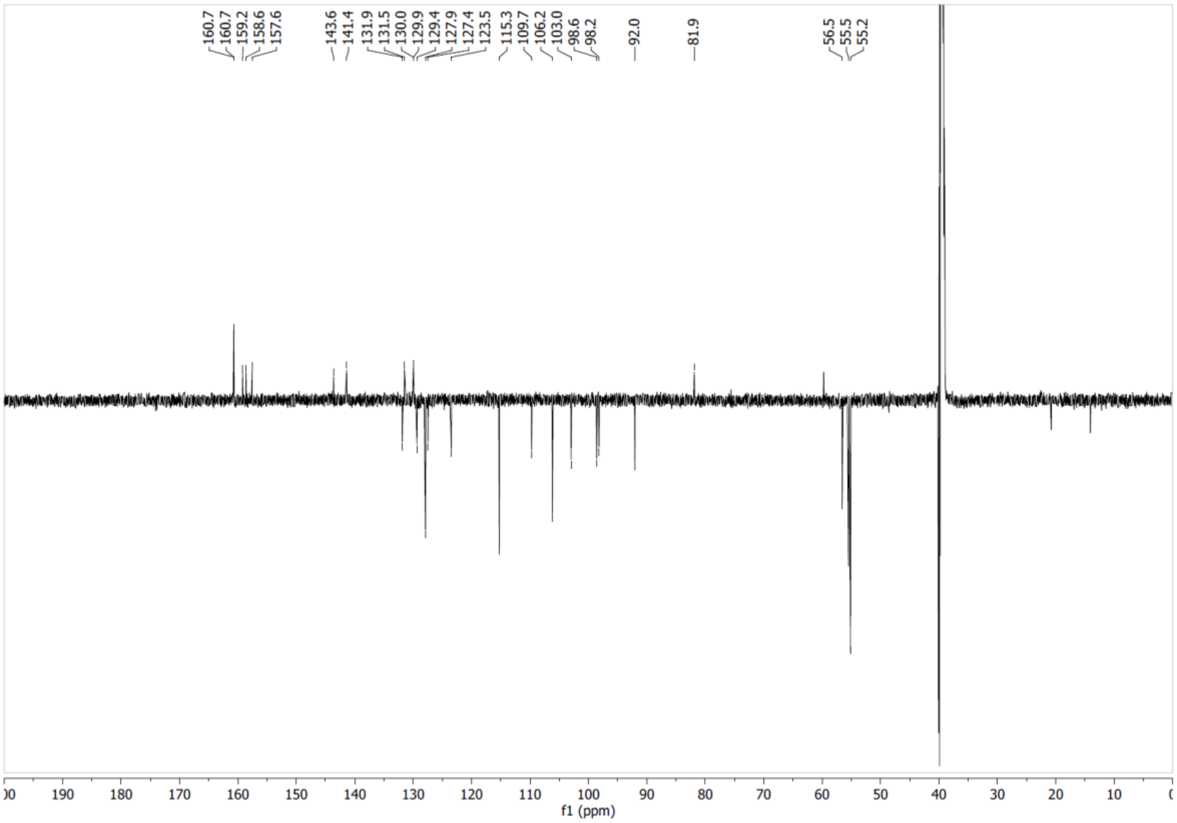

^{13}C-DEPTQ NMR spectrum of compound **43** in DMSO-d_6 at 151 MHz

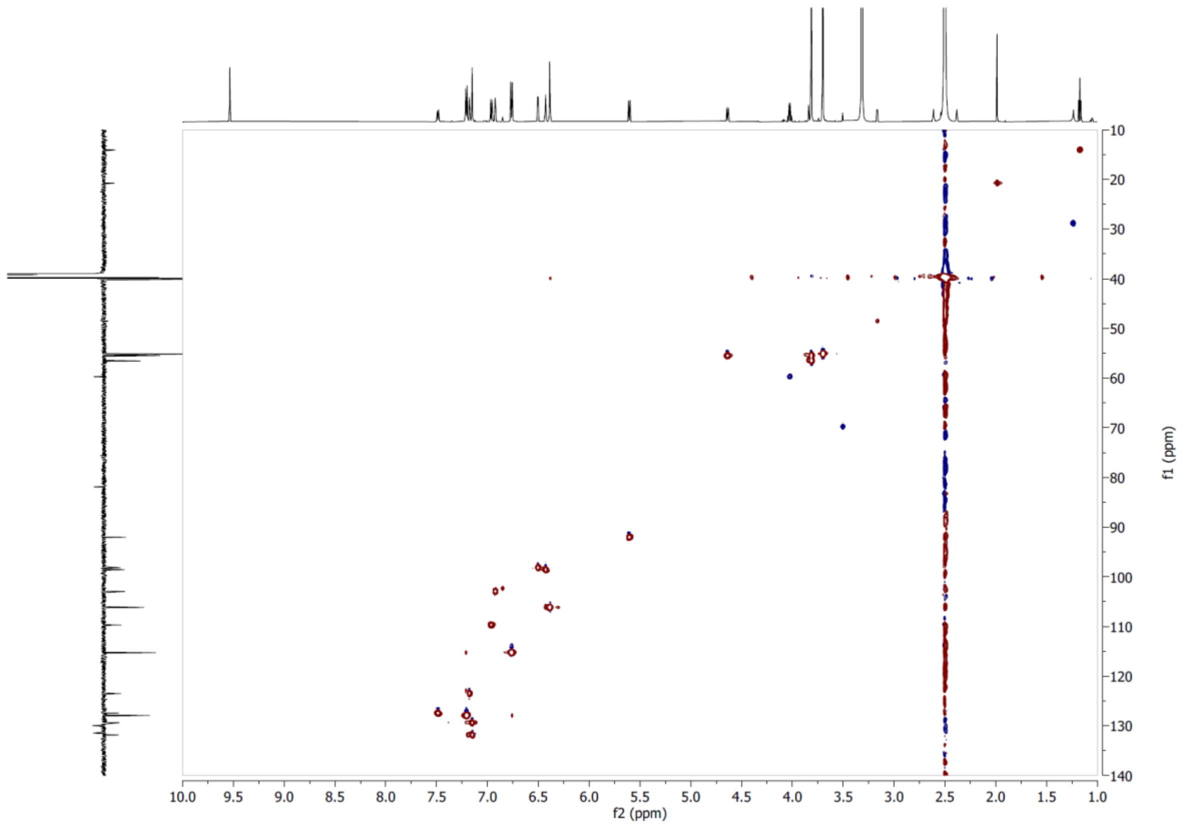

Edited HSQC NMR spectrum of compound **43** in DMSO-d_6

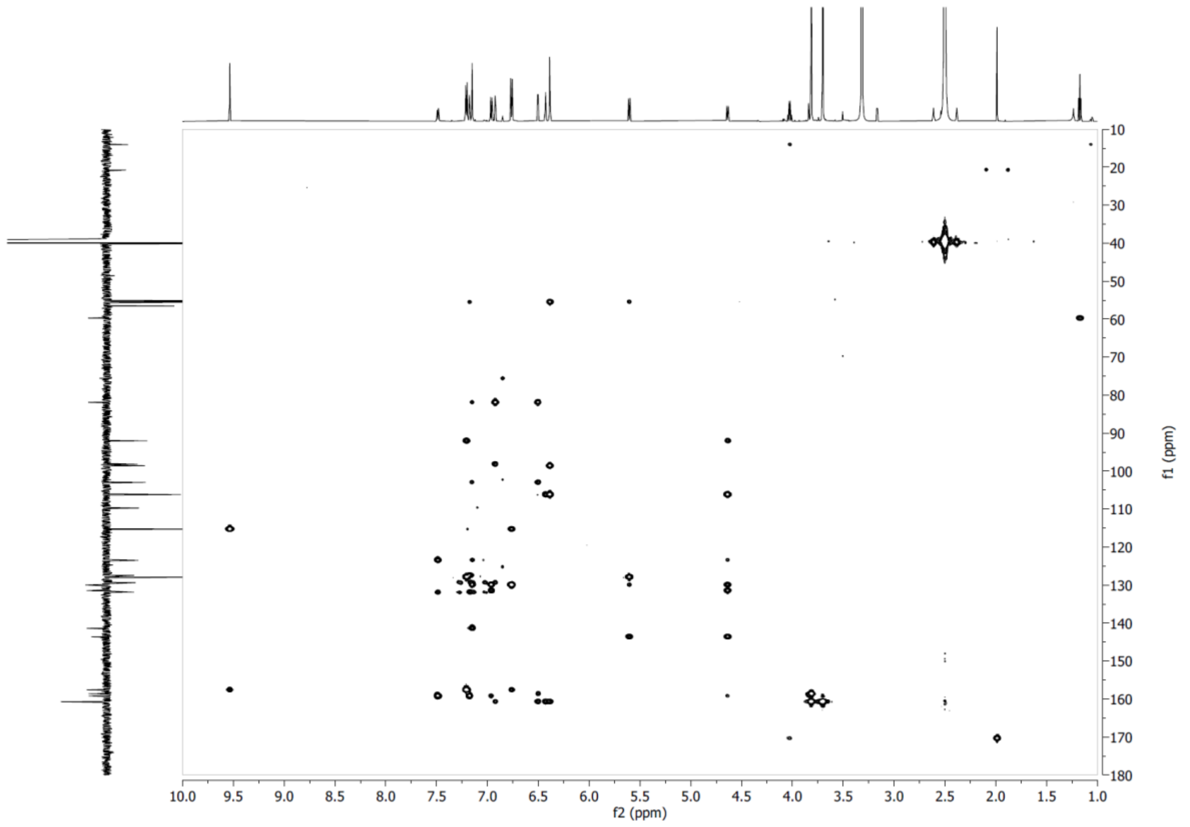

HMBC NMR spectrum of compound **43** in DMSO-d_6

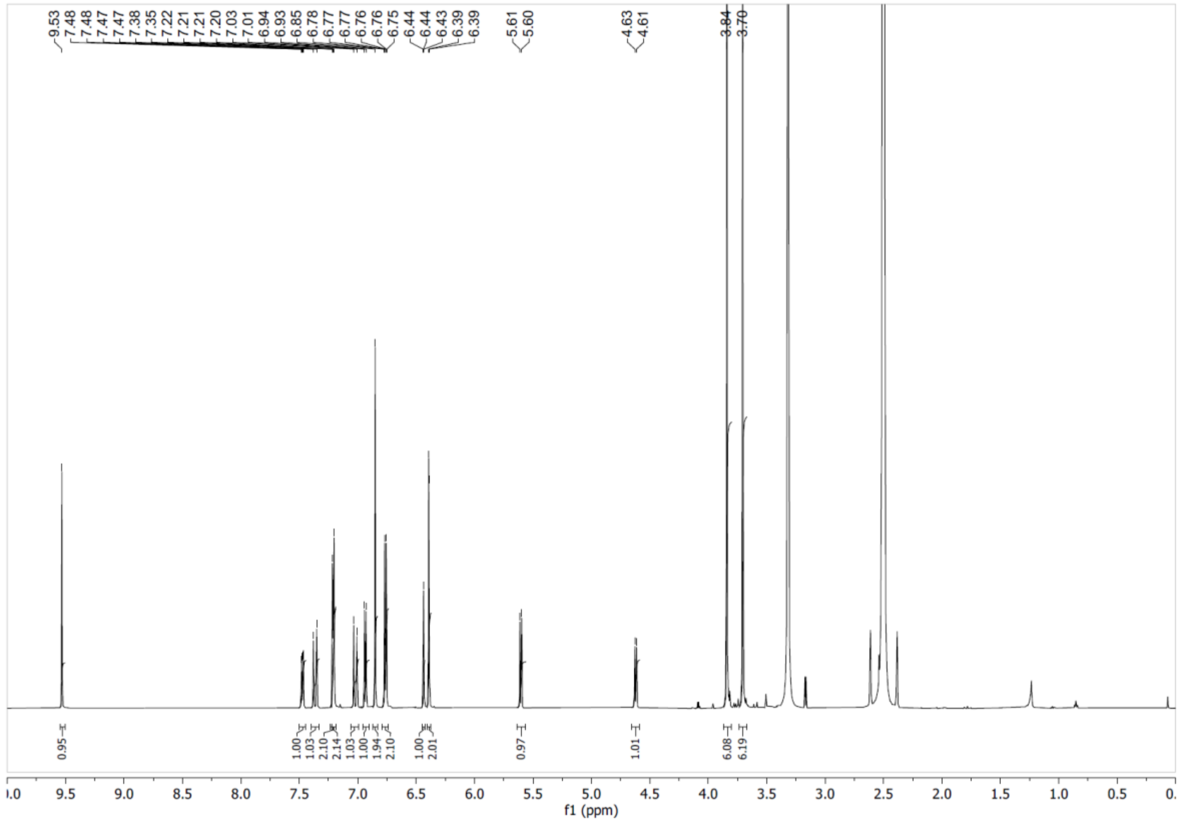

^{1}H NMR spectrum of compound **44** in DMSO-d_6 at 600 MHz

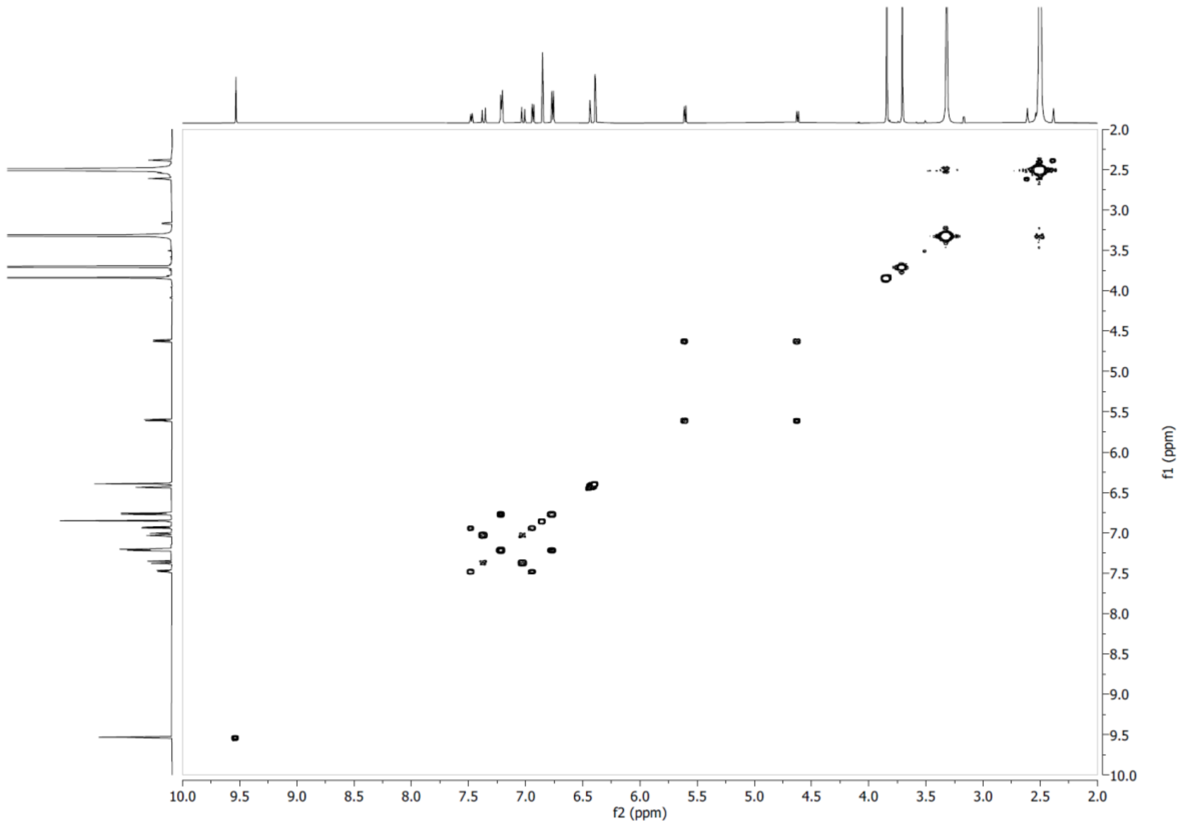

COSY NMR spectrum of compound **44** in DMSO-d_6

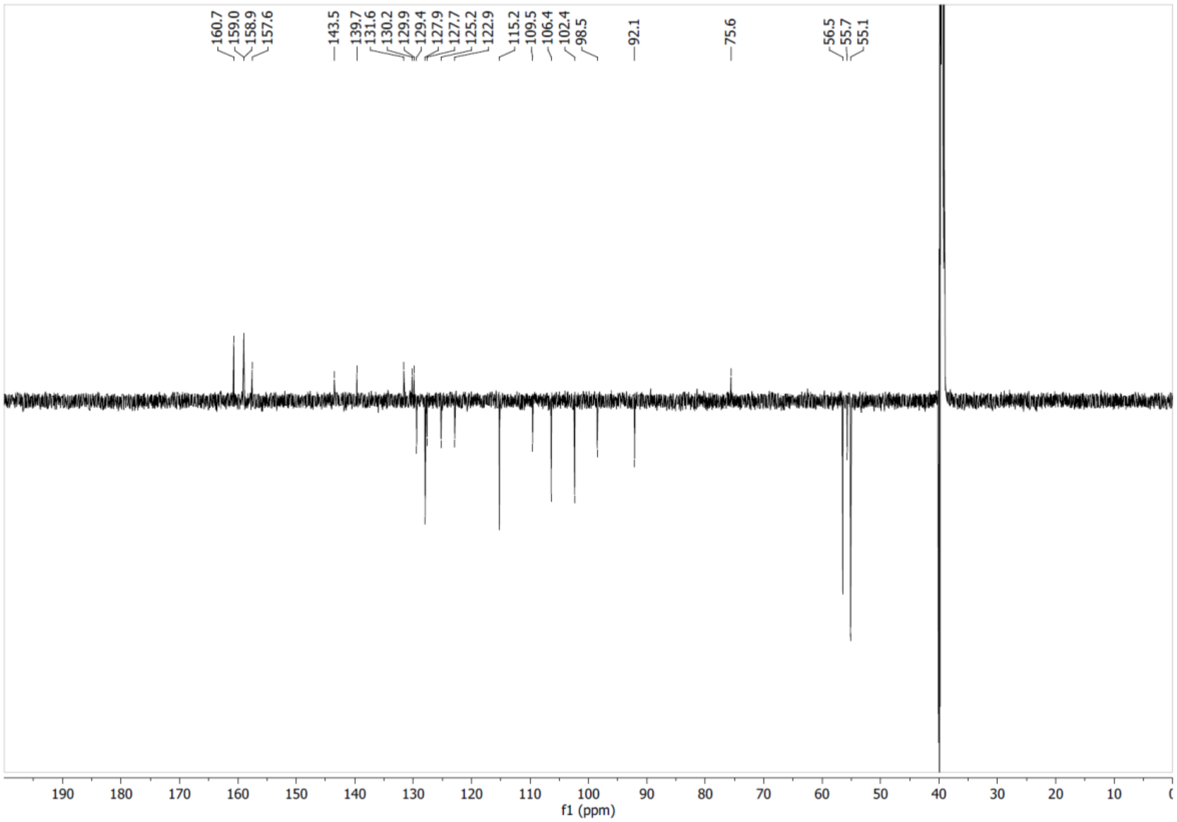

^{13}C-DEPTQ NMR spectrum of compound **44** in DMSO-d_6 at 151 MHz

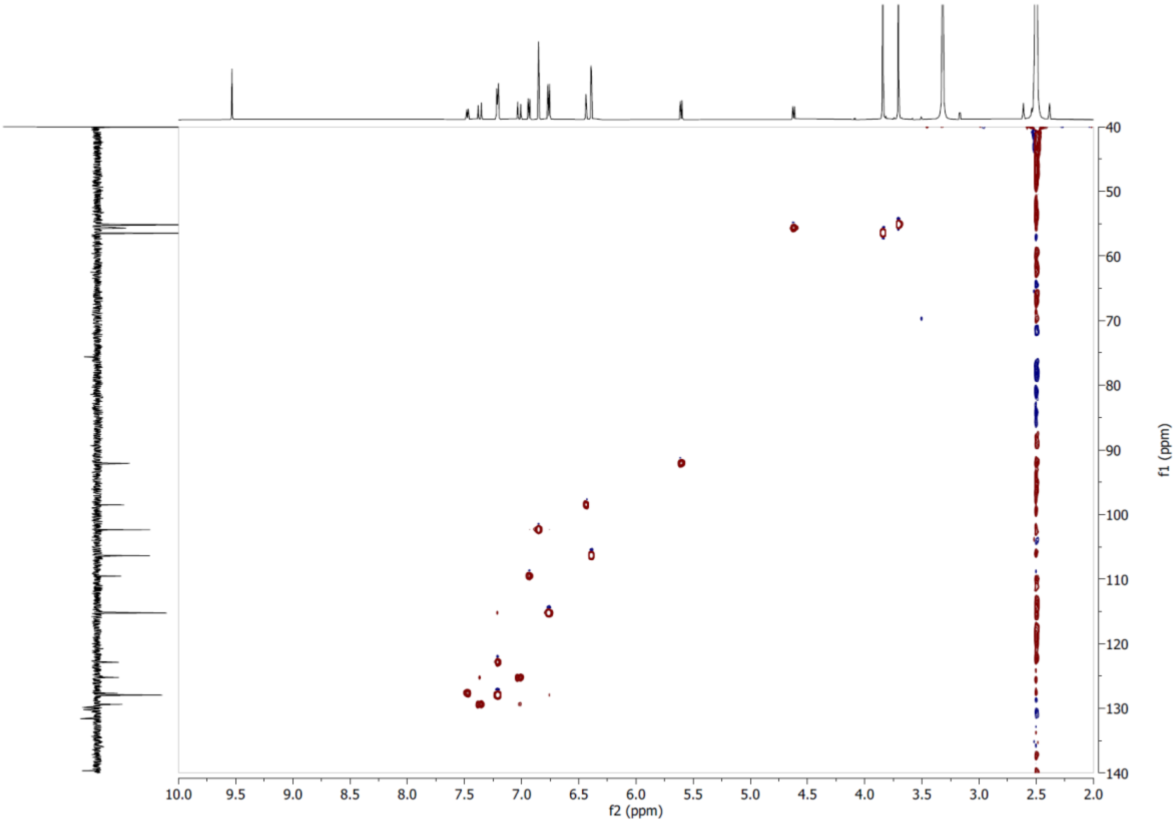

Edited HSQC NMR spectrum of compound **44** in DMSO-d_6

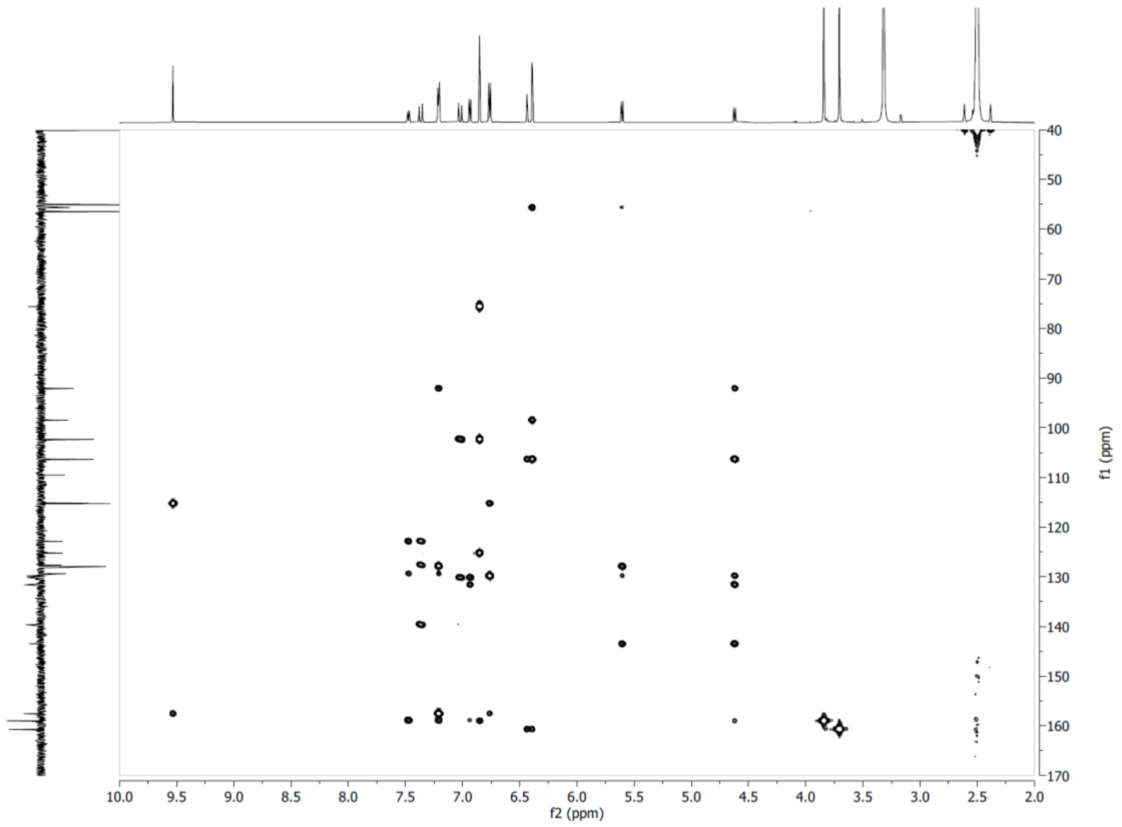

HMBC NMR spectrum of compound **44** in DMSO-d_6
